# The global epidemiology of injecting drug use, HIV, viral hepatitis and tuberculosis among people who are incarcerated: a multistage systematic review^☆^

**DOI:** 10.1016/j.drugpo.2025.105062

**Published:** 2026-02-05

**Authors:** Louisa Degenhardt, Matthew Hickman, Frederick L. Altice, Jason Grebely, Sophia Taylor, Michelle Lynch, Aleksa Kamenjaš, Jack Marsden, Lucy T. Tran, Paige Webb, Olivia Price, Christel Macdonald, Filipa Alves da Costa, Justin Berk, Anja Busse, Evan Cunningham, Colleen Daniels, Behzad Hajarizadeh, Linda Montanari, Luis Royuela, Keith Sabin, Jack Stone, Annette Verster, Peter Vickerman, Michael Farrell, Thomas Santo

**Affiliations:** a National Drug and Alcohol Research Centre (NDARC), University of New South Wales Sydney (UNSW Sydney), Sydney, Australia; b Population Health Sciences, Bristol Medical School, University of Bristol, Bristol, United Kingdom; c Yale University, New Haven, United States; d Kirby Institute, University of New South Wales Sydney (UNSW Sydney), Sydney, Australia; e University of Lisbon, Lisbon, Portugal; f Brown University, Providence, United States; g Department of Mental Health, Brain Health and Substance Use, World Health Organization (WHO), Geneva, Switzerland; h European Union Drugs Agency, Lisbon, Portugal; i Joint United Nations Programme on HIV/AIDS (UNAIDS), Geneva, Switzerland; j Department of Global HIV, Viral Hepatitis and STI Programmes (HSS), World Health Organization (WHO), Geneva, Switzerland; k Médecins Sans Frontières (MSF) Access Asia Pacific, Kuala Lumpur, Malaysia

**Keywords:** Incarceration, Criminal justice settings, Injecting drug use, HIV, Hepatitis, Tuberculosis, Epidemiology, Prevalence

## Abstract

**Background:**

This global systematic review assesses the prevalence of injecting drug use (IDU) and key infectious diseases (HIV, hepatitis C virus [HCV], tuberculosis and hepatitis B virus [HBV]) among people who are incarcerated.

**Methods:**

We conducted a systematic search of peer-reviewed (Medline, Embase, PsycINFO), internet, and grey literature databases, from January 2000 through 2nd June 2025 and engaged international experts and relevant agencies liaising with key agencies focused on incarcerated populations (WHO, UNODC, UNAIDS and EUDA). Data on study methods, size of incarcerated populations and demographic characteristics, and prevalence of IDU, HIV, HCV, HBV and tuberculosis among incarcerated populations were extracted. Meta-analyses pooled data where multiple estimates were available for a country; regional and global estimates were calculated, weighted by incarcerated population size. We present overall country, regional and global prevalence estimates for each variable examined, stratified by sex. We then estimated the ratio of IDU, HIV, HCV, HBV and tuberculosis prevalence among incarcerated populations compared to the general population.

**Results:**

Of 75,755 screened documents, 2,968 were eligible for data extraction. There are approximately 11,322,000 people aged 15–64 years incarcerated globally with their incarceration rate being 221 per 100,000 (29 per 100,000 among females and 404 per 100,000 among males). Substantial variation in rates across countries and regions were observed with the highest regional rate being in North America. Globally, we estimate that 11·9% of people who are incarcerated have ever injected drugs (1,348,000; 95%CI 1,061,500–1,687,000), 51·4 times higher than the general population. We estimate that 3·7% (95%CI 2·5–5·4) of people who are incarcerated globally are living with HIV (25.1· times higher than the general population); 11·7% (95%CI 7·7–17·1) have current HCV infection (15·6 times higher); 4·4% (95%CI 2·4–7·7) have current HBV infection (2·2 times higher) and 2·5% (95%CI 1·5–3·8) have active tuberculosis (45·3 times higher than the general population). There is substantial variation geographically and among females and males.

**Conclusion:**

The substantial concentration of people with multiple risks and comorbidities requires improved strategies to screen, evaluate, treat and prevent these adverse consequences, which is crucial for global control efforts.

**Funding:**

Australian National Health and Medical Research Council.

## Introduction

Incarcerated populations worldwide bear a disproportionately high risk for infectious diseases including HIV, tuberculosis and viral hepatitis (HCV and HBV) (Altice et al., 2016; Bosworth et al., 2022; Dolan et al., 2016). Moreover, people who inject drugs (PWID) are at increased risk of both being incarcerated having been infected with these infections and being at risk of exposure during incarceration (Altice et al., 2016; Bosworth et al., 2022; Dolan et al., 2016). In the absence of decarceration and decriminalisation efforts, “carceral environments” (defined here as jails, prisons, and correctional facilities, explicitly excluding immigration or compulsory detention centres) become pivotal venues for preventing, diagnosing, and treating globally prevalent infectious diseases.

There is a lack of robust evidence on population estimates of the health problems in people who are incarcerated, causing major barriers for national, regional, and global policymakers in evaluating progress towards UN Sustainable Development Goals (United Nations, 2025). A 2016 review examined HIV, HCV, HBV and tuberculosis in incarcerated populations (Dolan et al., 2016), with an update of this review among key populations published in 2018 (Wirtz et al., 2018), a 2018 review examined injecting drug use (Moazen et al., 2018), and reviews of tuberculosis prevalence among people who are incarcerated have been undertaken more recently (Martinez et al., 2023; Mera et al., 2023; Placeres et al., 2023). In every instance, the search scope was limited, often restricted to peer-reviewed literature, and failed to produce population size estimates, sex-disaggregated pooled estimates, or comparative risk assessments against the general population.

We undertook a multistage global systematic review of peer-reviewed and grey literature to estimate the prevalence and numbers of people with injecting drug use, HIV, and current HCV, HBV and tuberculosis infections among people who are incarcerated, including examining these among both females and males. We estimated prevalence and population sizes at country, regional and global levels and compared prevalence in people who are incarcerated to the general population.

## Methods

### Search strategy and selection criteria

We conducted a systematic review using methods consistent with previous global reviews (Degenhardt et al., 2017; Mathers et al., 2008; Nelson et al., 2011) and in accordance with PRISMA (Moher et al., 2009) and GATHER (Stevens et al., 2025) guidelines (Appendix 1). The review protocol was registered on PROSPERO (CRD42023425532). Searches were conducted in several stages with no limitations on languages. Searches and contacts with country experts and key agencies continued until December 2024.

We searched electronic peer-reviewed literature databases (Medline, EMBASE, PsycINFO, Web of Science, and CINAHL) using a comprehensive set of search terms developed in consultation with a specialist drug and alcohol librarian (Appendix 2). This included a set of terms for incarceration settings, injecting drug use, blood-borne viruses and tuberculosis, and harm reduction interventions for people who use drugs (Appendix 2). Searches were conducted March 7th-10th 2023, and were limited to those published from January 1st, 2000 onwards. An updated search was done on June 2nd 2025, and was limited to reports published between March 10th 2023 and June 2nd 2025 (Appendix 2). Systematic reviews were hand-searched for relevant original papers or reports within them.

Grey literature and online databases identified as sources of papers or reports on people who are incarcerated were systematically searched using their own search functions or Google Advanced Search (Appendix 2). These sources included websites of drug surveillance systems, regional harm-reduction networks, criminal justice-related websites, and country-specific ministries/departments of health, justice or interior. National and region-specific websites were searched in both English and the national language of the country, with translations facilitated by Google Translate (see Appendix 3 for sites; see also Ottaviano et al., 2023).

We searched key documents published by relevant international agencies, including UN Office on Drugs and Crime’s (UNODC) World Drug Reports (United Nations Office on Drugs & Crime, 2022), Harm Reduction International’s Global State of Harm Reduction reports (Harm Reduction International, 2022,^2023^), reports from the European Union Drug Agency (EUDA) (European Union Drug Agency, 2024a, 2024b), World Health Organization (WHO), UNAIDS and The Global Fund to Fight AIDS, Tuberculosis and Malaria. We contacted members of these organisations directly when additional information was required and liaised with those agencies until completion of the review.

Data were also requested from experts in November 2023, via an email distribution process and social media. This comprised initial emails sent to key experts and organisations, and posts on Twitter and Facebook, with requests to circulate to broader networks (Appendix 4). Searches and contacts with country experts and key agencies continued until December 2024.

### Screening and data extraction

An EndNote 20 library was created to catalogue papers and reports, with duplicates removed. We had members proficient in reading English, French, Serbo-Croatian, Portuguese, and Spanish; other languages were read via Google Translate or the Microsoft Word 365 translate function. Initial screening of title and abstract was done by two independent reviewers (ST, LD, ML, SO, AK, PW, BY, BC, MB, JG, TS, RJ, OP, BH, EBC) with discrepancies resolved via consensus with at least one other reviewer. Papers were included at title and abstract or full-text stage if they met a defined set of criteria for inclusion in the review (Appendix 5). Full-text review was also independently undertaken by two reviewers (AK, ST, BY, JM, ML, LD, JW, NO, MB, TS, BC, EL, OP or CM). Papers and reports were excluded if they met any of the following: samples sizes fewer than 40; cohort studies without baseline data; case control studies; non-original works (e.g., reviews or editorials); papers with insufficient methodological details; or samples of subpopulations (e.g., only people with HIV) (Appendix 5). A classification system was used for grading studies included in the review according to the extent to which the study sample was representative of the entire country’s incarcerated population (Appendix 6).

Data from eligible studies were extracted into purpose-built databases using Microsoft Access and REDCap at city, sub-national, or country level and double-checked for accuracy. Countries and regional groupings were based upon those used by UNAIDS, WHO and UNODC (Colledge-Frisby et al., 2023; Degenhardt et al., 2017, 2023; Larney et al., 2017).

Data on the estimates of the size of incarcerated populations were obtained from the most recent data reported in the World Prison Brief (WPB) (https://www.prisonstudies.org/). Data were unavailable for some countries from the WPB, including incarcerated population estimates (Eritrea and Palestine) and ratios of females and males (Eritrea, Palestine, Cuba and Uzbekistan). Estimates of females and males for these countries were imputed from the population-weighted average female-to-male prisoner ratio in the relevant regions. We used data from the UN Population Division to obtain general population numbers aged 15–64 years, using country population data produced for 2023.

To estimate the potentially elevated rates of IDU and infectious diseases among incarcerated populations (people, females and males separately) compared to the general population, we used data on general population prevalence from the sources below: Injecting drug use prevalence (Degenhardt et al., 2023); prevalence of current HCV infection (RNA positive) (Grebely et al., 2018); UNAIDS data on HIV among the general population (van Schalkwyk et al., 2024); WHO data on active tuberculosis infection among the general population (World Health Organization, 2024b), and WHO data on current HBV infections (HBsAg detectable) among the general population (World Health Organization, 2024a).

### Analysis of prevalence of IDU, blood-borne viruses, and tuberculosis

We used an approach consistent with the methods used in earlier reviews of people who inject drugs in the community (Degenhardt et al., 2017; Mathers et al., 2008; Nelson et al., 2011). Eligible data on the prevalence of injecting drug use, HIV antibody, HBsAg, HCV antibody, HCV RNA, and tuberculosis were extracted and, where multiple estimates were available, pooled for each country via random-effects meta-analyses in Stata (version 14 Stata Corporation, 2016, command: *metaprop)* (decision rules regarding selection of estimates are shown in the text box and Appendix 7). Metaprop allows meta-analyses of proportions for binomial data. The confidence intervals (CIs) were computed using an exact method (the Clopper-Pearson interval method) based on the binomial distribution (Clopper & Pearson, 1934; Puza & O’neill, 2006). In cases where CIs estimates fell outside the 0–100 % range, the double arcsine transformation method was used (command: *ftt*), because it is the preferred method for addressing the problem of variance instability in addition to the CI range problem. Current HCV infection was estimated using HCV RNA prevalence in countries where these data were available. If HCV RNA data were unavailable, HCV antibody prevalence data were used to estimate current HCV infection, assuming a 25 % clearance proportion as reported previously (Grebely et al., 2018). Tuberculosis infection was based on people whose sputum culture or GeneXpert was positive, or whose clinical presentation was consistent with TB and they responded to treatment.

Based on these extracted data, country-level prevalence estimates were calculated. The prevalence and estimated number of people with lifetime injecting drug use, and people living with HIV, HCV, tuberculosis and HBV were estimated by multiplying our prevalence or proportion estimates with the country, regional, and global population sizes of people who are incarcerated at country, regional and global levels.

## Text box about here

### Regional and global estimates

Regional and global estimates were produced using their respective country-level data. Countries where estimates for both females and males were not available had an imputed country-level prevalence estimate calculated using the methods stated in Appendix 8. Where data on females and males were located (which was often the case since most facilities are limited to females or males only), the overall country-level prevalence estimate was derived by aggregating relevant data for females and males. Following the collation of country-level estimates of incarceration and the prevalence of IDU and infectious diseases, regional and global estimates were derived. Region-specific, carceral population-weighted estimates were made using all the observed estimates and 95 % CIs of estimates in each country within that region, consistent with our previous reviews. Regional estimates were then used to estimate the global prevalence. Full details on methods of estimating regional and global numbers are in Appendix 8.

We estimated the prevalence and absolute numbers of incarcerated individuals with a history of injecting drug use, HIV, HCV, tuberculosis, and HBV by applying our prevalence rates to population sizes at the country, regional, and global levels.

### Sensitivity analysis

A sensitivity analysis was undertaken to examine the potential effect of using only more recent data (and thereby providing some evidence about potential shifts over time in prevalence of injecting drug use and infectious disease among incarcerated people, if indeed data had been collected over an extended period within a country). In that analysis, only data from the most recent five years within a country for each indicator were included in the pooled estimates of regional and global prevalence of injecting drug use and infectious diseases. The results of that sensitivity analysis are presented in Appendix 10.

### Risk of bias

We applied the Joanna Briggs Institute critical appraisal checklist for prevalence studies (Munn et al., 2020) to each included study. Each item was scored Yes (1), No (0), or Unclear (9) using the operational rules specified in Appendix 14 (Table 14.1). The results of this grading for each study included for each indicator are presented in Appendices 15-16.

### Role of funding source

The funders had no role in the design, conduct, analysis or interpretation of findings.

## Results

**Fig. 1.**
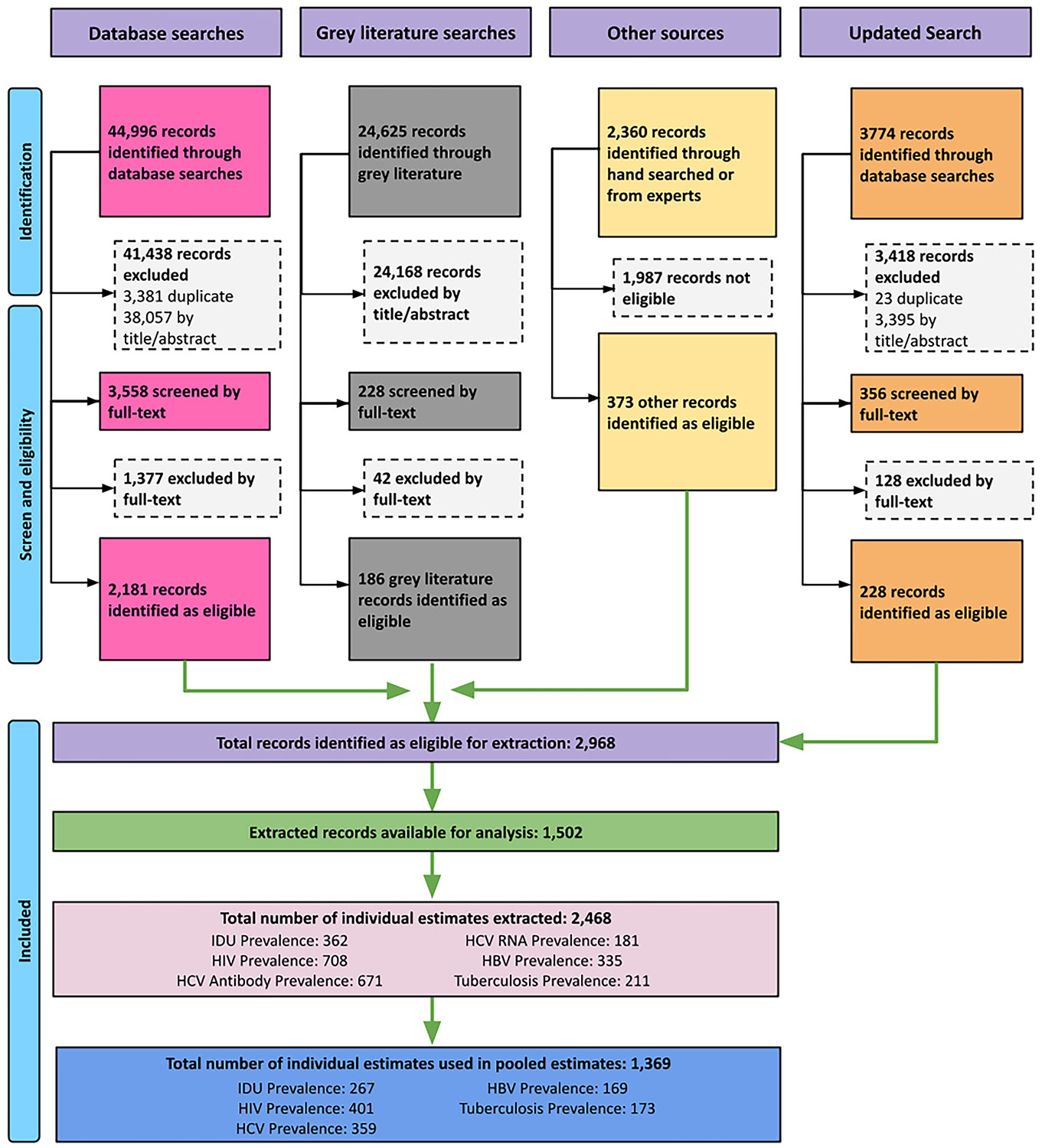
Flowchart.

We screened 75,755 papers or reports published between 2000 and 2025, with 2,968 eligible for extraction (Fig. 1). The total number of eligible estimates extracted was 2468, including 362 for IDU, 708 for HIV, 671 for HCV antibody, 181 for HCV RNA, 335 for HBV, and 211 for tuberculosis prevalence. The number of estimates included in pooled analyses (after applying our decision rules) was 1,369: 267 for IDU, 401 for HIV, 359 for HCV, 169 for HBV and 173 for tuberculosis prevalence (Fig. 1). Fig. 1 displays further details on the record and estimate selection process. Appendix 11 provides details of every eligible study located in this review, including which pooled estimates each study contributed data to (Appendix 12 shows studies excluded at full text screening stage).

### Incarceration rates and population numbers

**Fig. 2.**
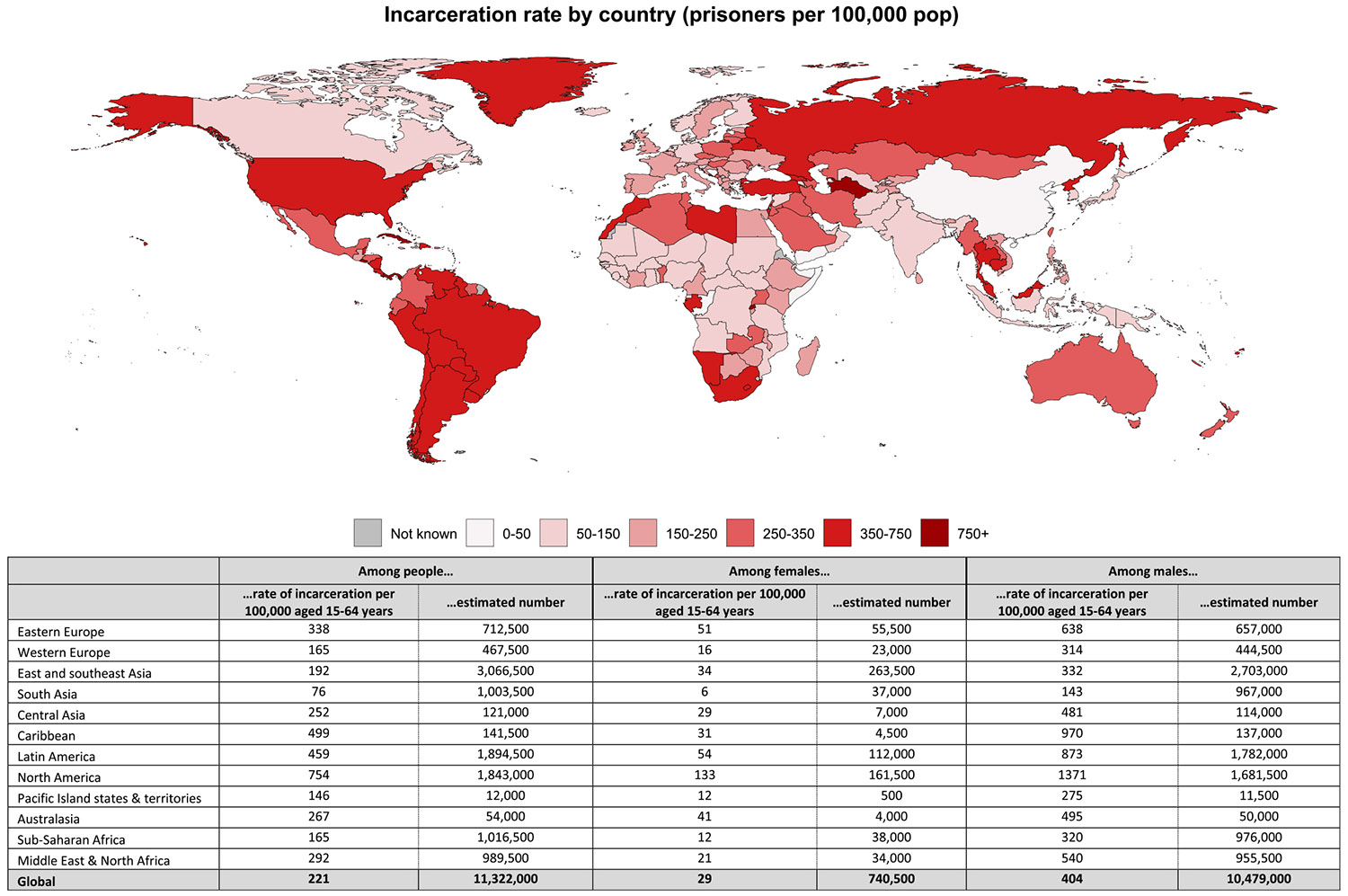
Estimates of the rate and number of people who are incarcerated per 100,000 people aged 15-64 years. Notes: Country level data that informed these regional and global estimates were sourced from the World Prison Brief, collated by the Institute for Crime and Justice Policy Research at Burbeck University. These contain the most recent available prison population estimates located by the WPB team. See: https://www.prisonstudies.org/world-prison-brief-data. Note that we used the country estimates to make rates for 15–64 years (not the total country population) so our rates differ from the World Prison Brief estimates.

Overall, there are an estimated 11,322,000 people aged 15–64 years incarcerated globally (a rate of 221 per 100,000), with the weighted global incarceration rate being 29 per 100,000 among females and 404 per 100,000 among men (Fig. 2). Globally, the rate of incarceration varies considerably by region and between countries – with the highest rates for men and females in North America and the lowest rates in South Asia. At the country level, the highest number of people incarcerated was in the United States, which had the 21st highest rate of incarceration. The highest rates of incarceration were recorded in Turkmenistan, Tonga, American Samoa, Rwanda, Cuba and El Salvador (see Table 2 for incarceration rates by country).

### Lifetime injecting drug use among people in carceral settings

**Fig. 3:**
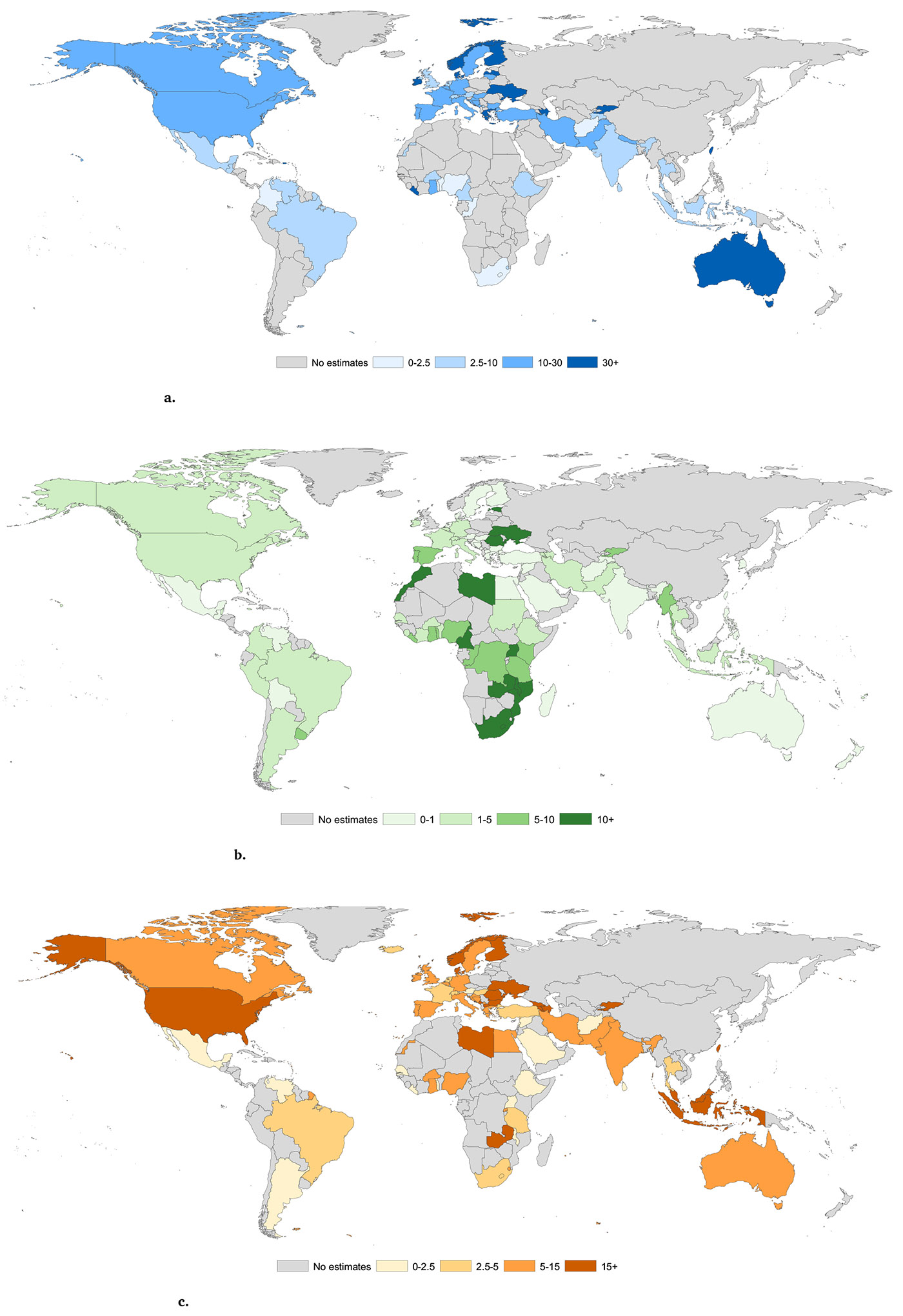

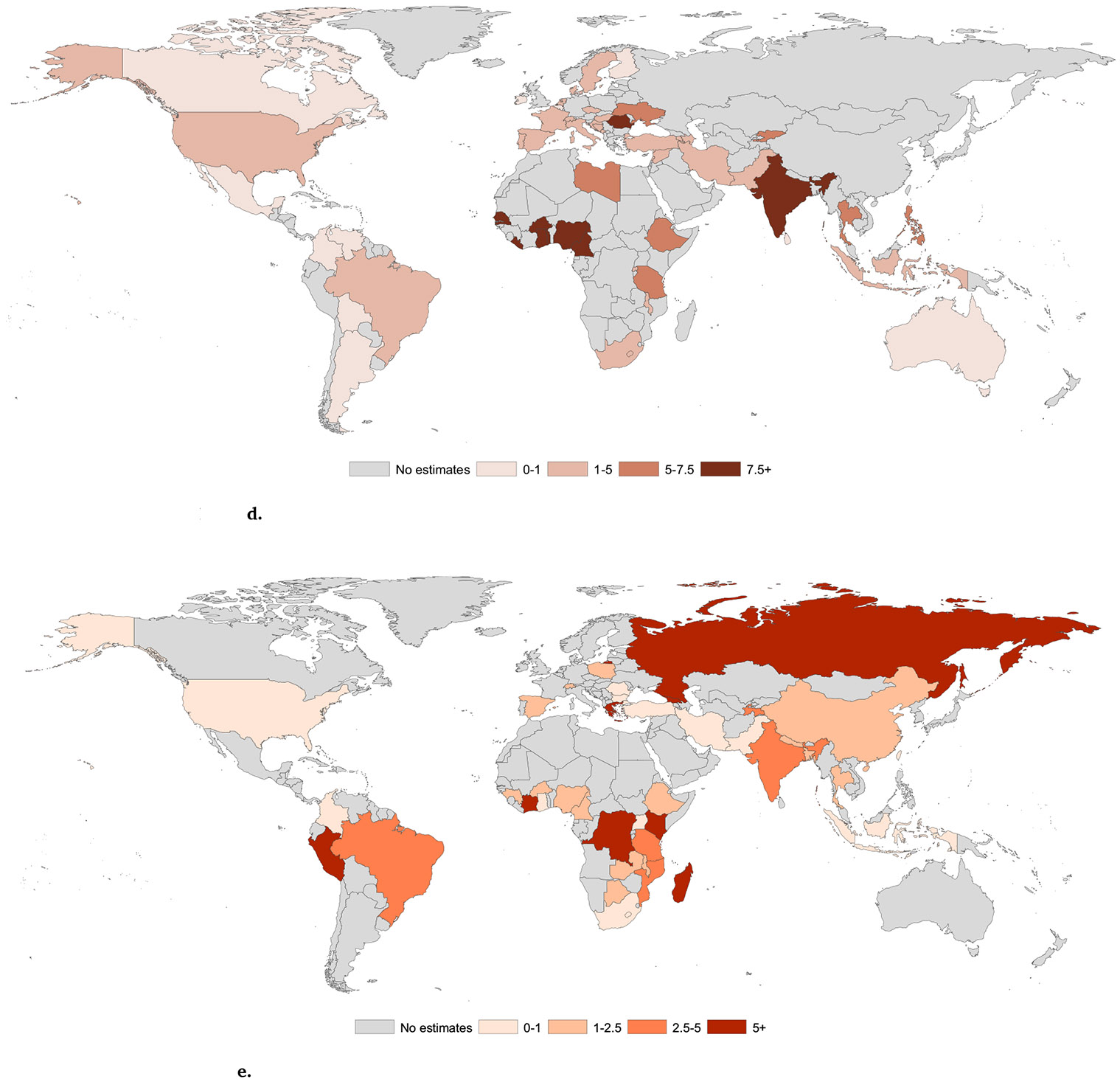
a. Prevalence (%) of lifetime injecting drug use among people who are incarcerated by country. **Fig. 3b.** Prevalence (%) of HIV among people who are incarcerated by country. **Fig. 3c.** Prevalence (%) of current HCV infection among people who are incarcerated by country. **Fig. 3d.** Prevalence (%) of HbsAg infection among people who are incarcerated by country. **Fig. 3e.** Prevalence (%) of active tuberculosis among people who are incarcerated by country.

Data on the prevalence of lifetime IDU among people in carceral settings were available in 62 countries and territories, covering 54·4 % of the world’s incarcerated population. Globally, we estimate that 1,348,000 (95 %CI 1,061,500-1,687,000) people who are incarcerated aged 15–64 years have ever injected drugs, amounting to 11·9 % (95 % CI 9·4–14·9) of all people incarcerated, translating to a prevalence that is approximately 51 times higher than in the general community (Table 1). Regionally, the lifetime prevalence of IDU in incarcerated populations varied substantially, from 3·0 % (95 %CI 2·1–4·2) in Sub-Saharan Africa to 51·0 % (95 %CI 47·0–54·9) in Australasia (country-level prevalence varied even more; these estimates are presented in Appendix 9). The ratio of IDU prevalence among people in carceral settings compared to the general population varied from approximately 10-fold higher in North America to over 190-fold higher in Western Europe (Table 1; country-level estimates are provided in Table 2 and shown in Fig. 3; Appendix 9 reports country-level data and sources in detail, and Appendix 15-16 **report** study level characteristics and risk of bias for each study included in country estimates for each indicator). The proportion of incarcerated females with a history of IDU also varied substantially (Table 1), with Australasia having the highest prevalence: 68·5 % (95 %CI 63·1–73·8), and lowest levels in the Middle East & North Africa (1·7 % [95 %CI 1·5–1·9]) and Sub-Saharan Africa (0·3 % [95 %CI 0·2–0·4]). The regional variation in lifetime IDU prevalence was similar for incarcerated males, with Australasia (49·5 % [95 %CI 45·6–53·3]) being considerably higher than the next highest region of Eastern Europe (33·4 % [95 %CI 29·2–37·8]); both Latin America (6·1 % [95 % CI 3·1–10·3]) and Sub-Saharan Africa (3·1 % [95 %CI 2·2–4·3]) had relatively low prevalence (Table 1).

### HIV, HCV, HBV and tuberculosis

Data from 92 countries, representing 65.6 % of the global incarcerated population, reveal that approximately 420,000 (95 %CI 281,000–612,500) incarcerated individuals, or 3·7 % (95 %CI 2·5–5·4), are people with HIV (the number of estimates informing our estimates by region are shown in the Table 2). This prevalence is 25·1 times higher than that in the general population. Notably, HIV prevalence globally (Table 1) is two-fold higher among females in carceral settings at 7·0 % (95 %CI 4·7–10·3) compared to males at 3·5 % (95 %CI 2·3–5·1). Regional variations show the lowest prevalence in Australasia at 0·7 % (95 %CI 0·3–1·4) and the highest in Eastern Europe at 16·6 % (95 %CI 13·7–19·6) and Sub-Saharan Africa at 9·6 % (95 %CI 6·2–14·1). Detailed country-level estimates are provided in Table 2 and Fig. 3 illustrates these findings (Appendix 9 reports country-level data and sources in detail, and Appendix 15-16 **report** study level characteristics and risk of bias for each study included in country estimates for each indicator). The disparity between HIV prevalence in incarcerated populations and the general population is most pronounced in Eastern Europe at 249 times higher, followed by the Middle East and North Africa at 59-fold higher, with the lowest ratios in Sub-Saharan Africa and the Caribbean at 2·9 and 2·6-fold higher, respectively.

Data from 67 countries, representing 58.6 % of the world’s incarcerated population, estimate that 1,321,500 (95 %CI 869,000–1,934,000) incarcerated people have current HCV infection. This amounts to 11·7 % (95 %CI 7·7–17·1) of all people who are incarcerated aged 15–64 years, an estimated 15·6-fold increase relative to the general global population (Table 1). Global prevalence of current HCV infection was similar for incarcerated females 11·8 % (95 %CI 7·8–17·3) compared to males 11·7 % (95 %CI 7·7–17·1). The highest regional prevalence for current HCV infection in carceral settings was seen in Eastern Europe 38·1 % (95 %CI 24·8–54·2) and North America 15·1 % (95 %CI 13·1–17·2). Data gaps exist, however, with no data from the Caribbean or the Pacific Islands & Territories (Table 1). East and Southeast Asia had the highest ratio of current HCV infection in carceral settings compared to the general population (25·5-fold higher), followed by South Asia (21·9-fold higher; Table 1). Fig. 3 shows country level data and Table 2 reports country-level estimates; Appendix 9 reports country-level data and sources in detail, and Appendix 15-16 **report** study level characteristics and risk of bias for each study included in country estimates for each indicator).

Data from 54 countries, covering 56·4 % of the world’s incarcerated population, indicate that 492,500 people (95 %CI 267,500–872,000), or 4·4 % (95 %CI 2·4–7·7), of all incarcerated individuals aged 15–64 years have current HBV infection, which is 2·2-fold higher than the general population (Table 1). The global prevalence of current HBV infection was similar in incarcerated females (3·4 % [95 %CI 1·8–6·0]) to that in incarcerated males (4·4 % [95 %CI 2·4– 7·8]). The regional prevalence of HBV infection varied greatly: Australasia (0·5 % [95 % CI 0·3–1·1]) and Latin America (0·8 % [95 %CI 0·2–2·0]) had much lower estimates than East and South-East Asia (6·1 % [95 %CI 4·3–8·4]), South Asia (6·1 [95 %CI 1·2–15·4]) and Sub-Saharan Africa (8·3 % [95 %CI 6·4–10·4]); country level data are reported in Fig. 3 and Table 2 (Appendix 9 reports country-level data and sources in detail, and Appendix 15-16 report study level characteristics and risk of bias for each study included in country estimates for each indicator). The ratio of HBV infection in carceral settings compared to that of the general population was highest in North America (8·2-fold higher) and Eastern Europe (3·8-fold higher), with people incarcerated in Australasia and Latin America having a lower risk than the general population (at 0·3 and 0.6 times, respectively; Table 1; Appendix 9 shows regional and country level ratios).

Data from 42 countries, representing 69·4 % of the world’s incarcerated population, indicate that 278,500 people (95 %CI 168,500–434,500), or 2·5 % (95 %CI 1·5–3·8), of all incarcerated individuals aged 15–64 years have active tuberculosis. This prevalence is 45·3 times higher than that in the global general population (Table 1). The global prevalence of active tuberculosis was almost 3-fold higher in incarcerated males (2·6 % [95 %CI 1·6–4·0]) compared to females (0·9 % [95 %CI 0·5–1·3]). Latin America (5·4 % [95 %CI 3·1- 8·4]), Eastern Europe (4·7 % [95 %CI 3·8–5·6]) and Sub-Saharan Africa (4·0 [95 %CI 1·8–7·2]) were the regions with the highest active tuberculosis prevalence. The Middle East and North Africa (1·6 % [95 %CI 0·9–2·5]), East and South-East Asia (1·4 % [95 %CI 0·8–2·1]), and North America (less than 0.01 %) (0·0 % [95 %CI 0·0–0·0]) reported the lowest active tuberculosis prevalence among incarcerated people (country level data are reported in Fig. 3 and Table 2; Appendix 9 reports country-level data and sources in detail, and Appendix 15-16 report study level characteristics and risk of bias for each study included in country estimates for each indicator). The ratio of active tuberculosis in carceral settings compared to that of the general population was considerably higher in Western Europe (502·4 times the prevalence) and Latin America (112·6 times the prevalence) than other regions (Appendix 9 and Table 1 show regional and country level ratios).

### Sensitivity analyses

We conducted sensitivity analyses examining the potential impact of restricting data to the most recent data for each country. In these analyses, we only included estimates from the most recent five years of data from within each country, for people, females and males. The regional estimates resulting from this approach are presented in Appendix 10. These estimates, although based on far fewer data points, were consistent with the estimates reported in the main analyses.

### Risk of bias

As noted earlier, each study was graded according to a range of methodological characteristics that permitted assessment of risk of bias against the JBI Prevalence Risk of Bias appraisal tool (see Appendix 14). No study providing data for any indicator was rated nine (out of a score of nine, i.e. for every study, on at least one measure out of nine, there was some feature that reflected a methodological characteristic that increased potential risk of bias in the study findings). For each indicator (injecting drug use, HIV, HCV, HBV and tuberculosis), the modal risk of bias score was five, indicating that there were most commonly four areas where a risk of bias existed in studies. Fewer than ten studies received a score of 8 across any indicator.

Across all indicators, the most common characteristics introducing potential bias were a) the nature of the sampling of participants (which were almost never census or representative samples of the entire incarcerated population being studied); b) poor sample coverage (recruitment into the study not offered to every eligible person); c) inadequate description of the study settings and participants (defined as reporting timeframe of recruitment, setting, age of sample and sex of participants); and d) details and adequacy of response rate (response rate both stated and considered high uptake). Appendix Tables 16.1 to 16.5 show the grades for every included study reported by indicator and country.

## Discussion

The incarceration epidemic involves an estimated 11.3 million people aged 15–64 incarcerated globally, with 30 to 70 million cycling through these settings annually, with the largest populations in East and South East Asia, Latin America and North America. There was clear variation in rates of incarceration, with the highest rate being in North America.

Overall, we estimate that one in nine people (11·9 %) who are incarcerated globally have injected drugs, which is over 50 times higher than in the general population. There was substantial variation across regions both in prevalence in incarcerated populations, from 3·0 % (Sub Saharan Africa) to 51·0 % (Australasia), and in IDU prevalence compared to the general population (Degenhardt et al., 2023). Crucial here is the magnitude of the elevated prevalence of infectious diseases in carceral settings relative to the general population, particularly for active tuberculosis (45·3-fold higher prevalence), HIV (25·1-fold higher) and HCV (15·6-fold higher). This review also estimated, for the first time, prevalence estimates for both females and males and compared prevalence within carceral settings to that of the general population.

Despite substantial geographic variation in the prevalence of all indicators examined, there remains a consistently elevated prevalence of infectious diseases and IDU in carceral settings across all regions compared to the general population, except for HBV. This trend was seen in both females and males, though regional differences exist in the relative prevalence between incarcerated females and males. For instance, Australasia and North America report a significantly higher prevalence of IDU among incarcerated females than males, whereas other regions generally exhibit lower prevalence rates among females.

This study provides the first systematic evidence across the globe showing the elevated prevalence of infectious disease in carceral populations, confirming that they are a critical target group in eliminating infectious disease. We estimated population sizes by country, region and globally, and also identified where incarcerated populations have a particularly elevated risk compared to the general population. This provides guidance at all levels on where and to what extent additional interventions within carceral settings are needed for countries to meet WHO targets to reduce morbidity and mortality due to tuberculosis, HIV, HCV and HBV as public health problems (World Health Organization, 2015, 2016, 2022, 2023). This can guide the extent to which the provision of prevention, harm reduction and treatment services are needed in carceral settings across countries, regions and the globe. We summarise the extent to which these services are currently being provided in carceral settings elsewhere (Santo et al., n.d.).

The scope of this review is far wider (incorporating peer-reviewed and grey literature and extensive interactions with key agencies focused on incarcerated populations) than previous reviews (Cords et al., 2021; Dolan et al., 2016). Our prevalence estimates are based on a much more rigorous and robust synthesis of the available evidence up until 2025. For example, the number of eligible data sources increased 10-fold compared to a previous global review (Dolan et al., 2016) (from 299 (Dolan et al., 2016) to 2968 in our review). We have generated the first estimates among females and males, and comparisons against the general population.

In this analysis we did not estimate the incidence of tuberculosis, as did a previous review (Cords et al., 2021), though we increased the number of studies of tuberculosis prevalence included in our review by 63 %. Our analysis revealed a global tuberculosis prevalence among incarcerated individuals of 2·5 %, which is more than double the previous estimate of 1·0 % (Cords et al., 2021). Moreover, the disparity in tuberculosis prevalence between incarcerated populations and the general population is 45-fold higher, far exceeding the earlier estimated of a 10-fold difference in tuberculosis incidence between prison and community (Cords et al., 2021).

## Limitations

Despite the strengths of this review, several limitations should be acknowledged particularly of the evidence. First, data gaps in some countries, particularly the Pacific Island States and Territories, introduce some uncertainty in estimates. Although we imputed data for these regions to generate global estimates, regional-level imputed estimates were not presented.

Second, estimates of IDU may be conservative due to potential under-reporting, as some individuals may have been reluctant to disclose prior risk-taking, even in surveys conducted by researchers independent of carceral authorities. This may mean that the prevalence of injecting drug use among people who are incarcerated is even higher than the recorded estimates suggest.

Third, population estimates for incarcerated individuals were derived from the World Prison Brief (https://www.prisonstudies.org/), the most authoritative source available. This is the most comprehensive source of data and the sources of those estimates are reported. Country information is updated on a monthly basis, using data that are most commonly obtained from governmental sources, but we could not independently verify them. Nonetheless the clear reporting of these data with reference to the source, the nature of the data (often a census count reported by the national government or similar body) mean that these are the best resource of such global statistics that we have been able to locate.

Fourth, data on population-wide disease screening for HIV, HCV, tuberculosis and HBV were limited. Most prevalence estimates were based on studies from select (often a single) facilities rather than nationally representative surveys, with only six studies using a census-based sampling approach in a carceral setting, and only one study utilising a fully national census approach. As within-country prevalence estimates may vary (Bose, 2018; Spaulding et al., 2023; Varan et al., 2014), there is a need for more focus on national screening and reporting to inform policy and practice. Indeed, in highlighting the limited epidemiological data on prevalence, this review has made somewhat more explicit the likely more common approach of reliance upon data about infectious disease as obtained from targeted screening and notifications data. In that sense, notifications data that may be more commonly available in carceral settings are likely to reflect not just variations in epidemiology of disease prevalence, but also varied screening algorithms used in these settings. As a result, in many instances epidemiological understanding of prevalence will reflect under-notification in routine programmatic data, meaning that policy in carceral settings may be being based on a biased understanding of the extent of the public health challenges of these diseases in the carceral system and highlights the importance of routine screening for infectious diseases. Having said that, we note that in some settings, for pragmatic reasons countries may not conduct studies of some infectious diseases (example.g. in countries where hepatitis B vaccination is universally offered in carceral settings such as Scotland, HBsAg may no longer be routinely measured in prevalence surveys).

Fifth, variation in tuberculosis case definitions and screening methods (e.g., reliance on chest radiographs versus clinical diagnosis) may have led to inconsistencies in reported tuberculosis prevalence. Some studies may have excluded individuals with diagnosed tuberculosis who were quarantined, potentially leading to underestimation of cases, while differences in defining "probable" versus "confirmed" tuberculosis cases may have contributed to variability in reported rates. Furthermore, there may be a potential overestimation of tuberculosis prevalence due to inclusion of outbreak papers in prevalence calculations.

Sixth, the use of convenience samples, without clear sampling frames and where it was a decision of incarcerated individuals to participant, may have affected the prevalence estimates observed in those studies. These types of studies might underestimate injecting drug use and infectious diseases (as those who are at risk may not participate) or, by contrast, could potentially overestimate population-level injecting drug use and infectious diseases (as those who might not feel that they are at risk might not participate) estimates. These concerns are shared with many community-level studies of the same behaviours that use the same convenience sampling approach.

This is the first complete review of this topic that incorporates peer-reviewed and grey literature, involved extensive interactions with key agencies focused on incarcerated populations. We have sought to include all available studies, but may have missed some. To address this as much as possible, we liaised directly with WHO, EUDA, UNODC and UNAIDS staff, and with many researchers across our global networks. We encourage feedback at global.reviews@unsw.edu.au, including enquiries from those interested in collaboration.

## Public health and policy implications

The extraordinary high burden of HIV, tuberculosis, and viral hepatitis in carceral settings identified here poses a significant challenge to global disease elimination efforts. The risk of within-prison disease transmission, compounded by poor access to screening and treatment, not only affects incarcerated individuals, but also drives infection rates in the broader population. Addressing this crisis requires an urgent public health response to expand evidence-based screening, prevention, and treatment interventions within prisons, and ensuring continuity of care both for transition into incarceration (e.g. ensuring that drug dependence or infectious disease treatment is continued following incarceration) *and* post-release.

The United Nations’ Mandela Rules mandate that individuals in prison should receive equivalent healthcare to that available in the community (United Nations Office on Drugs & Crime, 2015). Our review, alongside our companion paper on intervention coverage (Santo et al., n.d.) underscores the need to adopt and extend reach throughout countries for routine evidence-based screening, treatment and prevention for each condition. Routine HIV, HCV, HBV, and tuberculosis screening, alongside HBV vaccination and harm reduction programs, should be standard practice (World Health Organization, 2012). Interventions such as opioid agonist treatment (OAT) and needle and syringe programmes (NSPs) for people who inject drugs, which have been proven to reduce disease transmission (Degenhardt et al., 2019; Gowing et al., 2011; Platt et al., 2018) and improve continuity of care within prison and after release, must be implemented widely in correctional settings and maintained after release to reduce broader population transmission risks (Degenhardt et al., 2019; Macdonald et al., 2024).

Prisons function as high-risk environments that amplify infectious disease transmission. Multiple mathematical models showing that the cumulative risk of exposure during incarceration is associated with much greater levels of infection risk (Altice et al., 2016; Liu et al., 2024; Stone et al., 2021, 2017). Conversely, effective case-finding and treatment in prison and linkage to care upon release are cost-effective (Shih et al., 2024; Ward et al., 2022) and can have disproportionate benefits in terms of reducing infectious disease transmission in the community. Expanding treatment access in prisons has the potential to reduce community transmission, helping countries meet WHO elimination targets for HIV, tuberculosis, and viral hepatitis (Girardin et al., 2019; Godin et al., 2021; Martin et al., 2020; Stone et al., 2023), and inform and improve progress in countries' SDG targets (Tavoschi et al., 2024; Winkelman et al., 2022; World Health Organization, 2023). Addressing the incarcerated population’s health needs is not just a prison health issue—it is a population-wide public health imperative.

One striking limitation that our review revealed is that insufficient data come from public health surveillance or programmatic sources. This highlights a lack of integration between carceral healthcare systems and national health systems. Although additional research can refine strategies, there is already sufficient data to justify immediate action. Instead of conducting further observational studies, resources should be directed toward evidence-informed intervention implementation and scaling up proven strategies in prisons to reduce transmission and improve health outcomes.

Given the elevated health risks and exposures in prison, there is a pressing need to re-evaluate criminal laws and sentencing policies, particularly for people who use/inject drugs, who are disproportionately incarcerated for minor, nonviolent offences. Incarceration-driven public health crises could be mitigated through decriminalisation strategies and alternatives to detention, such as evidence-based drug treatment and diversion programs that keep people out of prison and within community healthcare systems. Decarceration strategies have been shown to yield substantial cost savings, which could be reinvested into scaling up prevention and treatment interventions in both prison and community settings. Shifting resources away from punitive approaches toward evidence-based public health responses will reduce health inequities, limit disease spread and strengthen national progress toward Sustainable Development Goals (SDGs) (United Nations, 2025).

The incarcerated population is a critical but often neglected group in global disease elimination efforts. Without urgent action, the high burden of infectious diseases in prisons will continue to undermine public health progress worldwide. Governments and international organisations must prioritise: 1) Routine screening, prevention, and treatment of HIV, HCV, HBV, and tuberculosis in carceral settings; 2) Services to prevent drug-related harm and treat drug dependence, including OAT and NSPs; 3) Stronger linkage-to-care programs to ensure continuity of treatment post-release; 4) Reform of the criminal legal system to reduce unnecessary incarceration, particularly for people who use drugs; and 5) Integration of prison health with national health systems to ensure equivalent healthcare for all. These strategies will not only protect the health of incarcerated individuals but also yield broader community benefits, strengthening national and global efforts to end HIV, tuberculosis and viral hepatitis as public health threats.

## Supplementary Material

9

11

3

10

8

6

2

7

5

4

1

Supplementary material associated with this article can be found, in the online version, at doi:10.1016/j.drugpo.2025.105062.

## Figures and Tables

**Table 1:** Regional and global estimates of the prevalence and number of people who are incarcerated who are living with HIV, have current HCV or HBV infection, have active tuberculosis and have injected drugs.

		Lifetime injecting drug use (IDU)							
	Incarc. per 100K people^1^	No. estimates	% (CI) among people	Estimated no. people who are incarcerated who have injected drugs (CI)	Ratio of IDU % among incarcerated people relative to gen. pop IDU %	% (CI) among females	Estimated no. females who are incarcerated who have injected drugs (CI)	% (CI) among males	Estimated no. males who are incarcerated who have injected drugs (CI)
Eastern Europe	338	13	32·3 (28·2, 36·6)	230,000 (201,000, 260,500)	63·0	19·3 (16·9, 21·9)	10,500 (9,500, 12,000)	33·4 (29·2, 37·8)	219,500 (191,500, 248,500)
Western Europe	165	80	23·8 (17·0, 32·1)	111,500 (79,500, 150,000)	192·8	26·3 (19·5, 34·3)	6,000 (4,500, 8,000)	23·7 (16·9, 32·0)	105,500 (75,000, 142,000)
East and southeast Asia	192	10	9·5 (8·3, 10·8)	291,500 (255,000, 331,500)	54·5	8·6 (7·5, 9·7)	23,500 (20,500, 26,500)	9·6 (8·4, 10·9)	268,500 (234,500, 305,000)
South Asia	76	38	7·6 (4·9, 11·2)	77,000 (49,000, 112,500)	44·2	1·8 (1·1, 2·6)	500 (500, 1,000)	7·9 (5·0, 11·5)	76,000 (48,500, 111,500)
Central Asia	252	3	19·8 (11·4, 28·4)	24,000 (14,000, 34,500)	31·0	2·1 (1·2, 3·0)	<500 (<500, <500)	20·9 (12·0, 29·9)	24,000 (13,500, 34,000)
Caribbean	499	1	12·7 (10·2, 15·7)	18,000 (14,500, 22,000)	··	10·1 (8·0, 12·6)	500 (500, 500)	12·8 (10·2, 15·8)	17,500 (14,000, 21,500)
Latin America	459	28	6·2 (3·1, 10·5)	118,500 (59,000, 199,500)	44·8	8·7 (4·1, 15·0)	10,000 (4,500, 17,000)	6·1 (3·1, 10·3)	108,500 (54,500, 183,000)
North America	754	49	13·4 (10·3, 16·8)	246,500 (190,500, 308,500)	9·6	20·6 (15·9, 25·8)	33,500 (25,500, 41,500)	12·7 (9·8, 15·9)	213,500 (165,000, 267,000)
Pacific Islands & territories	146	0	··	··	··	··	··	··	··
Australasia	267	23	51·0 (47·0, 54·9)	27,500 (25,500, 29,500)	84·9	68·5 (63·1, 73·8)	3,000 (2,500, 3,000)	49·5 (45·6, 53·3)	24,500 (22,500, 26,500)
Sub-Saharan Africa	165	16	3·0 (2·1, 4·2)	31,000 (22,000, 43,000)	39·6	0·3 (0·2, 0·4)	<500 (<500, <500)	3·1 (2·2, 4·3)	30,500 (22,000, 42,500)
Middle East and North Africa	292	6	15·9 (14·2, 17·8)	158,000 (140,000, 176,500)	77·7	1·7 (1·5, 1·9)	500 (500, 500)	16·5 (14·6, 18·4)	157,000 (139,500, 176,000)
**Global**	**221**	**267**	**11·9 (9·4, 14·9)**	**1,348,000 (1,061,500, 1,687,000)**	**51·4**	**10·5 (8·3, 13·1)**	**78,500 (62,000, 98,500)**	**12·0 (9·5, 15·0)**	**1,269,000 (999,500, 1,589,000)**
		HIV							
	Incarc. per 100K people^1^	No. estimates	% (CI) among people	Estimated no. people who are incarcerated living with HIV (CI)	Ratio of HIV % among incarcerated people relative to gen. pop HIV %	% (CI) among females	Estimated no. females who are incarcerated living with HIV (CI)	% (CI) among males	Estimated no. males who are incarcerated living with HIV (CI)
Eastern Europe	338	18	16·6 (13·7, 19·6)	118,500 (98,000, 140,000)	249·2	30·2 (24·9, 35·7)	17,000 (14,000, 20,000)	15·5 (12·8, 18·3)	102,000 (84,000, 120,000)
Western Europe	165	85	2·3 (0·9, 4·3)	10,500 (4,500, 20,500)	15·8	5·7 (2·3, 11·2)	1,500 (500, 2,500)	2·1 (0·9, 4·0)	9,500 (4,000, 17,500)
East and southeast Asia	192	14	2·1 (1·4, 3·2)	64,000 (43,500, 98,000)	3·1	6·5 (4·3, 10·4)	17,500 (12,000, 28,500)	1·7 (1·1, 2·5)	46,000 (31,500, 70,000)
South Asia	76	31	1·2 (0·7, 1·9)	12,500 (7,000, 19,500)	8·4	0·2 (0·1, 0·4)	<500 (<500, <500)	1·3 (0·7, 2·0)	12,500 (7,000, 19,000)
Central Asia	252	2	3·2 (2·2, 4·4)	4,000 (2,500, 5,500)	11·0	0·3 (0·2, 0·5)	<500 (<500, <500)	3·4 (2·3, 4·6)	4,000 (2,500, 5,500)
Caribbean	499	7	3·1 (1·7, 5·0)	4,500 (2,500, 7,000)	2·6	0·3 (0·2, 0·5)	<500 (<500, <500)	3·2 (1·8, 5·1)	4,500 (2,500, 7,000)
Latin America	459	59	2·3 (1·3, 3·7)	44,000 (24,500, 70,000)	4·4	3·5 (1·9, 5·6)	4,000 (2,000, 6,500)	2·2 (1·2, 3·6)	40,000 (22,000, 63,500)
North America	754	88	1·6 (1·3, 2·0)	30,000 (24,500, 36,500)	4·4	2·6 (2·1, 3·1)	4,000 (3,500, 5,000)	1·5 (1·3, 1·9)	26,000 (21,000, 31,500)
Pacific Islands & territories	146	1	3·2 (2·0, 5·0)	500 (<500, 500)	3·5	6·1 (4·1, 9·0)	<500 (<500, <500)	3·1 (1·9, 4·8)	500 (<500, 500)
Australasia	267	2	0·7 (0·3, 1·4)	500 (<500, 1,000)	6·6	0·6 (0·1, 3·8)	<500 (<500, <500)	0·7 (0·3, 1·4)	500 (<500, 500)
Sub-Saharan Africa	165	83	9·6 (6·2, 14·1)	97,500 (63,500, 143,500)	2·8	16·1 (10·4, 23·6)	6,000 (4,000, 9,000)	9·3 (6·1, 13·7)	91,500 (59,500, 134,500)
Middle East and North Africa	292	11	3·4 (1·0, 7·2)	33,500 (10,500, 71,500)	57·5	0·3 (0·1, 0·6)	<500 (<500, <500)	3·5 (1·1, 7·5)	33,500 (10,500, 71,500)
**Global**	**221**	**401**	**3·7 (2·5, 5·4)**	**420,000 (281,000, 612,500)**	**25·1**	**7·0 (4·7, 10·3)**	**52,500 (35,500, 77,000)**	**3·5 (2·3, 5·1)**	**367,000 (245,500, 535,500)**
		Current HCV infection							
	Incarc. per 100K people^1^	No. estimates	% (CI) among people	Estimated no. people who are incarcerated with current HCV infection (CI)	Ratio of HCV % among incarcerated people relative to gen. pop HCV %	% (CI) among females	Estimated no. females who are incarcerated with current HCV infection (CI)	% (CI) among males	Estimated no. males who are incarcerated with current HCV infection (CI)
Eastern Europe	338	22	38·1 (24·8, 54·2)	271,500 (176,500, 386,000)	10·9	13·3 (8·6, 18·9)	7,500 (5,000, 10,500)	40·2 (26·2, 57·2)	264,500 (172,000, 375,500)
Western Europe	165	130	7·9 (5·0, 11·7)	37,000 (23,500, 54,500)	19·0	8·5 (5·3, 12·7)	2,000 (1,000, 3,000)	7·8 (5·0, 11·6)	35,000 (22,000, 51,500)
East and southeast Asia	192	7	14·0 (7·1, 24·3)	431,000 (217,500, 745,500)	25·5	1·5 (0·8, 2·5)	4,000 (2,000, 7,000)	15·3 (7·7, 26·4)	427,000 (215,500, 738,500)
South Asia	76	40	8·3 (6·7, 10·4)	83,500 (67,500, 104,500)	21·9	3·0 (2·3, 4·1)	1,000 (1,000, 1,500)	8·5 (6·9, 10·7)	82,000 (66,500, 103,000)
Central Asia*	252	1	13·0 (8·9, 18·3)	15,500 (11,000, 22,000)	··	11·5 (7·6, 16·9)	1,000 (500, 1,000)	13·0 (9·0, 18·4)	15,000 (10,500, 21,000)
Caribbean	499	0	··	··	··	··	··	··	··
Latin America	459	52	4·0 (2·0, 6·8)	76,000 (37,500, 128,500)	5·5	3·3 (1·6, 5·7)	3,500 (2,000, 6,500)	4·1 (2·0, 6·8)	72,000 (35,500, 122,000)
North America	754	63	15·1 (13·1, 17·2)	277,500 (241,000, 316,000)	17·7	21·1 (18·3, 24·0)	34,000 (29,500, 39,000)	14·5 (12·6, 16·5)	243,500 (211,500, 277,500)
Pacific Islands & territories	146	0	·	·	·	·	·	·	·
Australasia	267	1	8·0 (6·4, 9·9)	4,500 (3,500, 5,500)	9·3	2·5 (0·9, 6·6)	<500 (<500, 500)	8·2 (6·6, 10·2)	4,000 (3,500, 5,000)
Sub-Saharan Africa	165	30	5·3 (3·8, 7·3)	53,500 (39,000, 74,000)	6·6	4·1 (2·4, 6·8)	1,500 (1,000, 2,500)	5·3 (3·9, 7·3)	52,000 (38,000, 71,500)
Middle East and North Africa	292	13	5·5 (4·1, 7·2)	54,000 (41,000, 71,000)	6·0	22·9 (17·0, 30·5)	8,000 (6,000, 10,500)	4·9 (3·7, 6·4)	46,500 (35,000, 61,000)
**Global**	**221**	**359**	**11·7 (7·7, 17·1)**	**1,321,500 (869,000, 1,934,000)**	**15·6**	**11·8 (7·8, 17·3)**	**88,500 (58,000, 129,500)**	**11·7 (7·7, 17·1)**	**1,233,000 (811,000, 1,804,500)**
		Current HBV infection							
	Incarc. per 100K people^1^	No. estimates	% (CI) among people	Estimated no. people who are incarcerated with current HBV infection (CI)	Ratio of HBV % among incarcerated people relative to gen. pop HBV %	% (CI) among females	Estimated no. females who are incarcerated with current HBV infection (CI)	% (CI) among males	Estimated no. males who are incarcerated with current HBV infection (CI)
Eastern Europe	338	11	5·3 (3·1, 8·1)	38,000 (22,000, 58,000)	3·8	2·9 (1·6, 4·5)	1,500 (1,000, 2,500)	5·5 (3·2, 8·4)	36,500 (21,000, 55,500)
Western Europe	165	41	1·7 (0·5, 3·6)	8,000 (2,500, 17,000)	2·0	4·8 (1·7, 10·4)	1,000 (500, 2,500)	1·5 (0·5, 3·3)	6,500 (2,000, 14,500)
East and southeast Asia	192	9	6·1 (4·3, 8·4)	188,000 (130,500, 257,000)	1·4	7·1 (4·9, 9·7)	19,500 (13,500, 26,500)	6·0 (4·2, 8·3)	169,000 (117,500, 231,000)
South Asia	76	24	6·1 (1·2, 15·4)	61,000 (12,000, 154,500)	2·6	1·3 (0·4, 2·8)	500 (<500, 1,000)	6·3 (1·2, 15·9)	60,500 (12,000, 153,500)
Central Asia	252	2	4·5 (2·4, 7·8)	5,500 (3,000, 9,500)	3·2	3·5 (1·8, 6·2)	<500 (<500, 500)	4·5 (2·5, 7·9)	5,000 (3,000, 9,000)
Caribbean	499	0	··	··	··	··	··	··	··
Latin America	459	30	0·8 (0·2, 2·0)	14,500 (4,000, 37,500)	0·6	0·6 (0·2, 1·6)	500 (<500, 2,000)	0·8 (0·2, 2·0)	13,500 (3,500, 36,000)
North America	754	9	2·9 (0·3, 9·1)	53,000 (4,500, 167,000)	8·2	0·6 (0·1, 1·9)	1,000 (<500, 3,000)	3·1 (0·3, 9·8)	52,000 (4,500, 164,000)
Pacific Islands & territories	146	0	··	··	··	··	··	··	··
Australasia	267	1	0·5 (0·3, 1·1)	500 (<500, 500)	0·3	0·4 (0·2, 2·5)	<500 (<500, <500)	0·5 (0·2, 1·1)	500 (<500, 500)
Sub-Saharan Africa	165	34	8·3 (6·4, 10·4)	84,500 (65,500, 106,000)	1·9	4·2 (3·0, 6·1)	1,500 (1,000, 2,500)	8·4 (6·6, 10·6)	83,000 (64,500, 104,000)
Middle East and North Africa	292	8	3·4 (2·0, 5·3)	34,000 (19,500, 52,500)	1·4	0·0 (0·0, 0·0)	<500 (<500, <500)	3·5 (2·1, 5·5)	34,000 (19,500, 52,500)
**Global**	**221**	**169**	**4·4 (2·4, 7·7)**	**492,500 (267,500, 872,000)**	**2·2**	**3·4 (1·8, 6·0)**	**25,500 (13,500, 45,000)**	**4·4 (2·4, 7·8)**	**467,500 (254,000, 826,500)**
		Active tuberculosis							
	Incarc. per 100K people^1^	No. estimates	% (CI) among people	Estimated no. people who are incarcerated with active tuberculosis (CI)	Ratio of TB % among incarcerated people relative to gen. pop TB %	% (CI) among females	Estimated no. females who are incarcerated with active tuberculosis (CI)	% (CI) among males	Estimated no. males who are incarcerated with active tuberculosis (CI)
Eastern Europe	338	6	4·7 (3·8, 5·6)	33,000 (27,500, 39,500)	85·1	0·3 (0·3, 0·6)	<500 (<500, 500)	5·0 (4·2, 6·0)	33,000 (27,500, 39,500)
Western Europe	165	5	3·0 (1·4, 6·7)	14,000 (6,500, 31,500)	502·4	5 (2·5, 10·4)	1,000 (500, 2,500)	2·9 (1·4, 6·5)	13,000 (6,000, 29,000)
East and southeast Asia	192	13	1·4 (0·8, 2·1)	41,500 (24,000, 65,500)	13·5	0·8 (0·5, 1·1)	2,000 (1,500, 3,000)	1·4 (0·8, 2·2)	39,500 (22,500, 62,500)
South Asia	76	21	2·3 (1·9, 2·7)	23,000 (19,500, 27,000)	8·6	0·6 (0·5, 0·7)	<500 (<500, <500)	2·4 (2·0, 2·8)	23,000 (19,500, 27,000)
Central Asia	252	1	2·7 (1·7, 4·0)	3,000 (2,000, 5,000)	72·8	0·8 (0·5, 1·3)	<500 (<500, <500)	2·8 (1·8, 4·2)	3,000 (2,000, 5,000)
Caribbean	499	0	··	··	··	··	··	··	··
Latin America	459	42	5·4 (3·1, 8·4)	101,500 (58,500, 159,000)	112·6	0·1 (0·1, 0·2)	<500 (<500, <500)	5·7 (3·3, 8·9)	101,500 (58,500, 159,000)
North America	754	6	0·0 (0·0, 0·0)	500 (500, 500)	5·3	0 (0, 0)	<500 (<500, <500)	0·0 (0·0, 0·0)	500 (500, 500)
Pacific Islands & territories	146	0	··	··	··	··	··	··	··
Australasia	267	0	··	··	··	··	··	··	··
Sub-Saharan Africa	165	77	4·0 (1·8, 7·2)	41,000 (18,000, 73,500)	11·8	3·7 (1·9, 6·2)	1,500 (500, 2,500)	4·1 (1·8, 7·3)	40,000 (17,500, 71,500)
Middle East and North Africa	292	2	1·6 (0·9, 2·5)	15,500 (9,500, 24,500)	4·3	0·4 (0·3, 0·7)	<500 (<500, <500)	1·6 (1·0, 2·5)	15,500 (9,000, 24,500)
**Global**	**221**	**173**	**2·5 (1·5, 3·8)**	**278,500 (168,500, 434,500)**	**45·3**	**0·9 (0·5, 1·3)**	**6,500 (4,000, 10,000)**	**2·6 (1·6, 4·0)**	**272,500 (164,500, 424,500)**

**Notes:** For country-level estimates of these characteristics and the sources for those estimates, please see Appendix 9. CI = confidence interval (see Appendix for details of estimation). **IDU:** injecting drug use; **HIV**: Human Immunodeficiency Virus; **HCV**: Hepatitis C; **HBV**: Hepatitis B; **TB**: tuberculosis; **gen. pop**. : general population; **incarc.**: incarcerated.

Indicates there were no data to inform a region’s estimate.

1 Country level data that informed these regional and global incarceration estimates were sourced from the World Prison Brief, collated by the Institute for Crime and Justice Policy Research at Burbeck University. See: https://www.prisonstudies.org/world-prison-brief-data. Note that we used the country estimates to make rates for 15-64 years (not the total country population) so our rates differ from the World Prison Brief estimates.

* The ratio of HCV among people who are incarcerated relative to the general population for Central Asia could not be calculated as there was no HCV estimate for the general population in Kyrgyzstan (our only HCV estimate for Central Asia is from Kyrgyzstan).

**Table 2:** Country estimates of the prevalence and number of people who are incarcerated who are living with HIV, have current HCV or HBV infection, have active tuberculosis and have injected drugs.

	People who are incarcerated		People with lifetime injecting drug use		People living with HIV		People with current HCV		People with current HBV		People with active Tuberculosis	
Country	Estimated number^1^	Rate per 100,000	% (CI)	Estimated no. (CI)	% (CI)	Estimated no. (CI)	% (CI)	Estimated no. (CI)	% (CI)	Estimated no. (CI)	% (CI)	Estimated no. (CI)
**Eastern Europe**												
Armenia	2469	132	··	··	1·3 (0·5, 2·5)	< 500 (<500,<500)	17·6 (14·5, 20·9)	500 (500,500)	3·6 (2·2, 5·4)	< 500 (<500,<500)	··	··
Azerbaijan	24698	345	32·0 (28·0, 36·1)	8000 (7000,9000)	3·6 (2·5, 5·1)	1000 (500,1500)	33·2 (22·7, 44·7)	8000 (5500,11000)	4·7 (3·2, 6·7)	1000 (1000,1500)	··	··
Belarus	32556	498	··	··	··	··	··	··	··	··	··	··
Bosnia & Herzegovina	2212	24	17·3 (14·5, 20·4)	500 (500,500)	0·0 (0·0, 0·3)	< 500 (<500,<500)	9·8 (6·1, 14·3)	< 500 (<500,500)	1·5 (0·7, 2·7)	< 500 (<500,<500)	··	··
Bulgaria	6378	146	28·5 (24·6, 32·6)	2000 (1500,2000)	0·7 (0·2, 1·4)	< 500 (<500,<500)	21·4 (18·4, 24·6)	1500 (1000,1500)	··	··	0·3 (0·0, 1·0)	< 500 (<500,<500)
Czechia	19649	294	··	··	··	··	··	··	4·1 (1·7, 7·5)	1000 (500,1500)	··	··
Estonia	1688	201	··	··	15·6 (14·1, 17·1)	500 (<500,500)	··	··	··	··	··	··
Georgia	10457	432	··	··	··	··	20·9 (20·2, 21·5)	2000 (2000,2500)	··	··	6·0 (5·5, 6·5)	500 (500,500)
Hungary	18270	289	13·5 (11·8, 15·2)	2500 (2000,3000)	0·0 (0·0, 0·0)	< 500 (<500,<500)	3·9 (2·9, 4·9)	500 (500,1000)	1·1 (0·6, 1·9)	< 500 (<500,500)	··	··
Latvia	3271	278	32·3 (29·1, 35·5)	1000 (1000,1000)	··	··	··	··	··	··	··	··
Lithuania	4551	254	12·0 (3·5, 24·4)	500 (<500,1000)	··	··	··	··	··	··	··	··
Republic of Moldova	5695	279	··	··	··	··	··	··	··	··	··	··
Poland	70316	279	··	··	··	··	··	··	··	··	1·6 (0·8, 2·8)	1000 (500,2000)
Romania	24534	195	··	··	56·7 (46·7, 66·4)	14000 (11500,16500)	20·9 (3·5, 47·3)	5000 (1000,11500)	10·6 (6·0, 16·3)	2500 (1500,4000)	0·1 (0·0, 0·2)	< 500 (<500,<500)
Russian Federation	433006	447	··	··	··	··	··	··	··	··	5·4 (4·6, 6·4)	23500 (20000,27500)
Slovakia	8585	236	··	··	··	··	··	··	··	··	··	··
Ukraine	44024	150	43·7 (38·9, 48·5)	19000 (17000,21500)	12·6 (10·8, 14·5)	5500 (4500,6500)	73·8 (50·5, 99·8)	32500 (22000,44000)	5·2 (3·2, 7·6)	2500 (1500,3500)	··	··
**Western Europe**												
Albania	4653	242	··	··	··	··	··	··	··	··	··	··
Andorra	51	89	··	··	··	··	··	··	··	··	··	··
Austria	9288	157	4·3 (0·5, 10·5)	500 (<500,1000)	0·0 (0·0, 2·6)	< 500 (<500,<500)	1·5 (0·0, 6·4)	< 500 (<500,500)	··	··	··	··
Belgium	12575	169	20·4 (17·3, 23·6)	2500 (2000,3000)	1·1 (0·1, 3·3)	< 500 (<500,500)	7·6 (3·7, 12·7)	1000 (500,1500)	0·8 (0·3, 1·5)	< 500 (<500,<500)	··	··
Croatia	4445	171	24·6 (22·9, 26·3)	1000 (1000,1000)	0·1 (0·0, 0·3)	< 500 (<500,<500)	10·4 (9·3, 11·6)	500 (500,500)	1·1 (0·8, 1·6)	< 500 (<500,<500)	··	··
Denmark	4083	110	43·3 (37·9, 48·7)	2000 (1500,2000)	0·0 (0·0, 0·6)	< 500 (<500,<500)	28·9 (24·1, 34·0)	1000 (1000,1500)	4·9 (2·7, 7·7)	< 500 (<500,500)	··	··
England and Wales	85867	223	23·7 (21·5, 26·1)	20500 (18500,22500)	0·2 (0·1, 0·4)	< 500 (<500,500)	6·3 (3·5, 10·0)	5500 (3000,8500)	0·9 (0·0, 2·7)	500 (<500,2500)	··	··
Finland	2912	85	54·5 (50·5, 58·5)	1500 (1500,1500)	0·9 (0·2, 1·9)	< 500 (<500,<500)	32·3 (28·3, 36·4)	1000 (1000,1000)	0·3 (0·0, 1·5)	< 500 (<500,<500)	··	··
France	79631	201	19·0 (5·7, 37·3)	15000 (4500,29500)	2·7 (0·8, 5·6)	2000 (500,4500)	3·5 (1·2, 6·7)	3000 (1000,5500)	1·2 (0·1, 3·4)	1000 (<500,2500)	··	··
Germany	57955	109	29·3 (21·5, 37·9)	17000 (12500,22000)	2·7 (0·7, 5·7)	1500 (500,3500)	5·5 (4·3, 6·9)	3000 (2500,4000)	··	··	··	··
Greece	10242	155	37·1 (30·9, 43·6)	4000 (3000,4500)	0·0 (0·0, 2·3)	< 500 (<500,<500)	11·2 (8·7, 14·0)	1000 (1000,1500)	··	··	13·3 (8·8, 18·4)	1500 (1000,2000)
Greenland	154	393	NK	NK	··	··	··	··	··	··	··	··
Iceland	140	57	··	··	··	··	3·1 (0·1, 9·2)	< 500 (<500,<500)	··	··	··	··
Ireland	5074	156	30·4 (24·9, 36·1)	1500 (1500,2000)	1·2 (0·5, 2·1)	< 500 (<500,<500)	10·8 (7·0, 15·4)	500 (500,1000)	0·3 (0·0, 0·8)	< 500 (<500,<500)	··	··
Italy	62110	166	27·7 (19·2, 38·0)	17000 (12000,23500)	3·1 (1·8, 4·9)	2000 (1000,3000)	11·1 (6·9, 16·2)	7000 (4500,10000)	3·0 (1·3, 5·3)	2000 (1000,3500)	··	··
Liechtenstein	14	54	··	··	··	··	··	··	··	··	··	··
Luxembourg	600	135	22·8 (17·1, 29·0)	< 500 (<500,<500)	··	··	··	··	··	··	··	··
Malta	671	187	··	··	1·3 (0·4, 2·6)	< 500 (<500,<500)	18·3 (15·0, 21·9)	< 500 (<500,<500)	··	··	0·8 (0·1, 2·1)	< 500 (<500,<500)
Monaco	31	165	··	··	··	··	··	··	··	··	··	··
Montenegro	1046	254	16·4 (13·3, 19·8)	< 500 (<500,<500)	0·0 (0·0, 0·7)	< 500 (<500,<500)	16·0 (12·9, 19·3)	< 500 (<500,<500)	1·5 (0·6, 2·9)	< 500 (<500,<500)	··	··
Netherlands	11537	102	8·6 (5·8, 12·0)	1000 (500,1500)	0·4 (0·0, 1·6)	< 500 (<500,<500)	4·9 (2·4, 8·2)	500 (500,1000)	1·5 (0·2, 3·7)	< 500 (<500,500)	··	··
North Macedonia	2555	176	··	··	0·0 (0·0, 0·9)	< 500 (<500,<500)	15·0 (10·4, 20·3)	500 (500,500)	··	··	··	··
Northern Ireland	1911	159	12·7 (8·9, 17·3)	< 500 (<500,500)	0·0 (0·0, 0·3)	< 500 (<500,<500)	0·8 (0·2, 1·6)	< 500 (<500,<500)	··	··	··	··
Norway	3052	87	51·6 (39·1, 64·0)	1500 (1000,2000)	··	··	24·2 (14·2, 35·7)	500 (500,1000)	··	··	··	··
Portugal	12379	188	14·7 (12·2, 17·5)	2000 (1500,2000)	6·1 (4·5, 8·0)	1000 (500,1000)	9·6 (7·7, 11·9)	1000 (1000,1500)	2·7 (1·9, 4·0)	500 (<500,500)	··	··
San Marino	1	4	··	··	··	··	··	··	··	··	··	··
Scotland	8253	235	19·9 (13·1, 27·6)	1500 (1000,2500)	0·7 (0·0, 2·2)	< 500 (<500,<500)	3·4 (2·5, 4·3)	500 (<500,500)	··	··	··	··
Serbia	10787	227	··	··	··	··	··	··	··	··	··	··
Slovenia	1798	133	··	··	··	··	··	··	··	··	··	··
Spain	56698	181	25·4 (19·4, 32·0)	14500 (11000,18000)	5·4 (2·4, 9·5)	3000 (1500,5500)	12·0 (7·6, 18·3)	7000 (4500,10500)	2·0 (0·6, 4·2)	1000 (500,2500)	1·3 (0·1, 5·2)	1000 (<500,3000)
Sweden	10175	156	25·1 (17·5, 33·7)	2500 (2000,3500)	0·4 (0·0, 1·6)	< 500 (<500,<500)	11·3 (8·0, 15·1)	1000 (1000,1500)	1·9 (0·8, 3·8)	< 500 (<500,500)	··	··
Switzerland	6881	120	4·5 (2·9, 6·5)	500 (<500,500)	2·2 (1·4, 3·1)	< 500 (<500,<500)	3·7 (2·7, 4·8)	500 (<500,500)	2·0 (1·2, 2·9)	< 500 (<500,<500)	1·7 (1·3, 2·2)	< 500 (<500,<500)
**East and South East Asia**												
Brunei Darussalam	636	199	··	··	··	··	··	··	··	··	··	··
Cambodia	45122	417	··	··	··	··	··	··	··	··	··	··
China	1690000	13	··	··	··	··	··	··	··	··	1·5 (0·9, 2·3)	26000 (15500,39000)
Hong Kong†	9079	178	··	··	··	··	··	··	··	··	5·4 (4·0, 7·0)	500 (500,500)
Indonesia	274060	148	5·2 (4·2, 6·2)	14000 (11500,17000)	1·1 (0·3, 2·2)	3000 (1000,6000)	22·4 (6·3, 46·6)	61500 (17500,128000)	5·0 (2·8, 7·7)	13500 (8000,21000)	0·0 (0·0, 0·5)	< 500 (<500,1500)
Japan	40881	56	··	··	··	··	··	··	··	··	··	··
Lao People's Democratic Republic	11885	262	··	··	··	··	··	··	··	··	··	··
Malaysia	87419	373	··	··	··	··	16·2 (15·6, 16·7)	14000 (13500,14500)	··	··	··	··
Mongolia	5700	270	··	··	··	··	··	··	··	··	··	··
Myanmar	100324	274	··	··	5·5 (4·9, 6·2)	5500 (5000,6000)	··	··	··	··	··	··
Democratic People's Republic of Korea*	100000	552	NK	NK	··	··	··	··	··	··	··	··
Philippines	171247	235	··	··	0·0 (0·0, 0·6)	< 500 (<500,1000)	··	··	7·4 (5·2, 9·9)	12500 (9000,17000)	··	··
Singapore	9536	217	··	··	··	··	··	··	··	··	··	··
Republic of Korea	52940	143	··	··	0·2 (0·1, 0·2)	< 500 (<500,<500)	··	··	··	··	··	··
Taiwan†	58889	346	42·3 (41·2, 43·3)	25000 (24500,25500)	0·0 (0·0, 2·4)	< 500 (<500,1500)	20·3 (18·5, 22·1)	12000 (11000,13000)	··	··	0·2 (0·2, 0·2)	< 500 (<500,<500)
Thailand	274277	550	6·8 (5·3, 8·4)	18500 (14500,23000)	4·0 (2·7, 5·5)	11000 (7500,15000)	3·7 (2·7, 4·9)	10000 (7500,13500)	6·5 (5·1, 8·2)	18000 (14000,22500)	1·7 (0·8, 2·9)	4500 (2000,8000)
Timor-Leste	763	97	··	··	··	··	··	··	··	··	··	··
Viet Nam	133986	200	··	··	··	··	··	··	··	··	··	··
**South Asia**												
Afghanistan	19000	87	1·9 (1·0, 3·2)	500 (<500,500)	0·8 (0·4, 1·4)	< 500 (<500,500)	2·2 (1·2, 3·5)	500 (<500,500)	··	··	··	··
Bangladesh	53831	47	··	··	··	··	··	··	··	··	1·0 (0·9, 1·1)	500 (500,500)
Bhutan	1119	227	··	··	··	··	··	··	··	··	··	··
India	573220	60	3·0 (2·1, 4·1)	17500 (12000,23500)	0·9 (0·6, 1·3)	5000 (3000,7500)	8·9 (8·4, 9·6)	51000 (48000,55000)	7·9 (0·6, 22·0)	45500 (3500,126000)	3·5 (3·0, 4·0)	20000 (17500,23000)
Iran (Islamic Republic of)	189000	314	15·5 (13·2, 18·1)	29500 (25000,34000)	2·0 (1·2, 3·0)	4000 (2000,5500)	8·4 (4·3, 13·9)	16000 (8000,26500)	3·4 (2·6, 4·4)	6500 (5000,8500)	0·1 (0·1, 0·2)	500 (<500,500)
Maldives	1700	448	··	··	··	··	··	··	··	··	··	··
Nepal	27550	142	10·8 (8·7, 13·1)	3000 (2500,3500)	··	··	··	··	··	··	1·8 (0·7, 3·2)	500 (<500,1000)
Pakistan	108643	80	19·5 (5·7, 38·7)	21000 (6000,42000)	1·9 (0·8, 3·5)	2000 (1000,4000)	8·3 (5·1, 12·1)	9000 (5500,13000)	2·7 (2·2, 3·4)	3000 (2500,3500)	0·5 (0·3, 0·7)	500 (500,1000)
Sri Lanka	29686	208	4·2 (2·4, 6·6)	1500 (500,2000)	··	··	0·6 (0·0, 1·9)	< 500 (<500,500)	0·3 (0·0, 1·4)	< 500 (<500,500)	··	··
**Central Asia**												
Kazakhstan	35228	294	··	··	··	··	··	··	··	··	··	··
Kyrgyzstan	7728	194	48·2 (26·4, 70·3)	3500 (2000,5500)	8·4 (5·8, 11·5)	500 (500,1000)	31·8 (27·1, 36·6)	2500 (2000,3000)	6·3 (3·7, 9·5)	500 (500,500)	··	··
Tajikistan	14000	238	4·1 (3·1, 5·2)	500 (500,500)	0·3 (0·2, 0·5)	< 500 (<500,<500)	··	··	··	··	4·4 (3·4, 5·5)	500 (500,1000)
Turkmalesistan	35000	864	··	··	··	··	··	··	··	··	··	··
Uzbekistan	29000	131	··	··	··	··	··	··	··	··	··	··
**Caribbean**												
Antigua & Barbuda	400	604	NK	NK	2·9 (0·4, 7·1)	< 500 (<500,<500)	··	··	··	··	··	··
Bahamas	1912	653	··	··	··	··	··	··	··	··	··	··
Barbados	692	367	··	··	··	··	··	··	··	··	··	··
Bermuda	124	295	··	··	··	··	··	··	··	··	··	··
Cuba	90000	1161	··	··	··	··	··	··	··	··	··	··
Dominica	260	507	NK	NK	2·6 (0·7, 5·4)	< 500 (<500,<500)	··	··	··	··	··	··
Dominican Republic	25987	357	··	··	··	··	··	··	··	··	··	··
Grenada	385	468	NK	NK	2·2 (0·3, 5·4)	< 500 (<500,<500)	··	··	··	··	··	··
Haiti	7523	104	··	··	··	··	··	··	··	··	··	··
Jamaica	3559	174	··	··	3·4 (2·3, 4·7)	< 500 (<500,<500)	··	··	··	··	··	··
Commonwealth of Puerto Rico	5798	278	31·6 (29·0, 34·3)	2000 (1500,2000)	··	··	··	··	··	··	··	··
Saint Kitts & Nevis	160	476	NK	NK	2·3 (0·5, 5·1)	< 500 (<500,<500)	··	··	··	··	··	··
Saint Lucia	572	438	NK	NK	2·0 (0·7, 3·7)	< 500 (<500,<500)	··	··	··	··	··	··
Saint Vincent & the Grenadines	404	577	NK	NK	4·0 (2·2, 6·3)	< 500 (<500,<500)	··	··	··	··	··	··
Trinidad & Tobago	3802	358	NK	NK	··	··	··	··	··	··	··	··
**Latin America**												
Argentina	125041	426	··	··	2·2 (1·4, 3·5)	2500 (1500,4500)	2·1 (1·3, 3·1)	2500 (1500,4000)	0·5 (0·3, 0·8)	500 (500,1000)	··	··
Belize	1339	501	NK	NK	4·1 (2·7, 5·8)	< 500 (<500,<500)	··	··	··	··	··	··
Bolivia (Plurinational State of)	31105	403	··	··	0·2 (0·0, 0·6)	< 500 (<500,<500)	··	··	0·5 (0·0, 1·6)	< 500 (<500,500)	··	··
Brazil	888791	593	7·0 (2·9, 12·6)	62000 (26000,112000)	3·2 (1·8, 5·1)	28500 (15500,45000)	4·9 (2·2, 8·6)	43500 (19500,76500)	1·1 (0·3, 2·6)	9500 (2500,23000)	3·8 (2·4, 5·8)	34000 (21000,51500)
Chile	59037	440	··	··	··	··	··	··	··	··	··	··
Colombia	104346	291	0·7 (0·1, 1·7)	500 (<500,2000)	1·2 (0·3, 2·4)	1000 (500,2500)	··	··	0·4 (0·0, 1·3)	500 (<500,1500)	0·8 (0·5, 1·1)	1000 (500,1000)
Costa Rica	17829	502	··	··	··	··	··	··	··	··	··	··
Ecuador	33669	286	··	··	··	··	··	··	··	··	··	··
El Salvador	109519	2625	··	··	··	··	··	··	··	··	··	··
Guatemala	23361	213	4·9 (3·3, 6·8)	1000 (1000,1500)	0·8 (0·2, 1·7)	< 500 (<500,500)	··	··	··	··	··	··
Guyana	2300	439	··	··	··	··	··	··	··	··	··	··
Honduras	19481	291	··	··	··	··	··	··	··	··	··	··
Mexico	234514	277	6·5 (5·5, 7·5)	15000 (13000,17500)	0·6 (0·5, 0·8)	1500 (1000,2000)	2·3 (2·0, 2·7)	5500 (4500,6500)	0·1 (0·0, 0·2)	< 500 (<500,500)	··	··
Nicaragua	20918	496	··	··	··	··	··	··	··	··	··	··
Panama	23798	841	··	··	··	··	··	··	··	··	··	··
Paraguay	17712	408	··	··	··	··	··	··	··	··	··	··
Peru	97605	443	··	··	1·2 (0·5, 2·3)	1000 (500,2000)	··	··	··	··	24·1 (12·2, 39·9)	23500 (12000,39000)
Suriname	1000	270	··	··	··	··	··	··	··	··	··	··
Uruguay	15767	707	··	··	6·8 (4·1, 10·1)	1000 (500,1500)	··	··	··	··	··	··
Venezuela (Bolivarian Republic of)	67200	375	5·2 (2·4, 8·8)	3500 (1500,6000)	0·6 (0·4, 0·8)	500 (500,500)	1·4 (0·2, 3·8)	1000 (<500,2500)	0·0 (0·0, 4·1)	< 500 (<500,2500)	··	··
**North America**												
Canada	34986	139	29·7 (26·8, 32·7)	10500 (9500,11500)	1·7 (1·2, 2·3)	500 (500,1000)	13·6 (11·1, 16·4)	4500 (4000,5500)	0·0 (0·0, 2·5)	< 500 (<500,1000)	··	··
United States of America	1808100	522	13·1 (10·0, 16·4)	236000 (181000,297500)	1·6 (1·3, 2·0)	29500 (24000,35500)	15·1 (13·1, 17·2)	272500 (237000,310500)	2·9 (0·3, 9·2)	53000 (4500,166500)	0·0 (0·0, 0·0)	500 (500,500)
**Pacific Island States & Terr·**												
American Samoa	301	1018	··	··	··	··	··	··	··	··	··	··
Micronesia (Federated States of)	132	40	··	··	··	··	··	··	··	··	··	··
Fiji	2276	377	··	··	1·0 (0·0, 3·1)	< 500 (<500,<500)	··	··	··	··	··	··
French Polynesia	575	275	··	··	··	··	··	··	··	··	··	··
Guam	896	844	··	··	··	··	··	··	··	··	··	··
Kiribati	129	179	··	··	··	··	··	··	··	··	··	··
Marshall Islands	35	117	··	··	··	··	··	··	··	··	··	··
Nauru	38	516	NK	NK	··	··	··	··	··	··	··	··
New Caledonia	609	317	··	··	··	··	··	··	··	··	··	··
Northern Mariana Islands	170	501	··	··	··	··	··	··	··	··	··	··
Palau	66	527	··	··	··	··	··	··	··	··	··	··
Papua New Guinea	5373	87	··	··	··	··	··	··	··	··	··	··
Samoa	358	296	··	··	··	··	··	··	··	··	··	··
Solomon Islands	500	131	··	··	··	··	··	··	··	··	··	··
Tonga	557	888	··	··	··	··	··	··	··	··	··	··
Tuvalu	11	161	··	··	··	··	··	··	··	··	··	··
Vanuatu	195	108	··	··	··	··	··	··	··	··	··	··
**Australasia**												
Australia‡§	44051	261	51·0 (47·0, 54·9)	22500 (20500,24000)	0·8 (0·4, 1·7)	500 (<500,500)	8·0 (6·4, 9·9)	3500 (3000,4500)	0·5 (0·3, 1·1)	< 500 (<500,500)	··	··
New Zealand	9924	297	··	··	0·0 (0·0, 0·1)	< 500 (<500,<500)	··	··	··	··	··	··
**Sub Saharan Africa**												
Angola	24068	134	··	··	··	··	··	··	··	··	··	··
Benin	19563	277	0·6 (0·1, 1·5)	< 500 (<500,500)	1·4 (0·5, 2·7)	500 (<500,500)	··	··	··	··	··	··
Botswana	3971	242	··	··	··	··	··	··	··	··	2·0 (1·4, 2·7)	< 500 (<500,<500)
Burkina Faso	8800	75	3·3 (2·4, 4·4)	500 (<500,500)	2·3 (1·6, 3·2)	< 500 (<500,500)	5·7 (4·7, 6·8)	500 (500,500)	28·0 (22·9, 33·7)	2500 (2000,3000)	1·3 (0·3, 3·0)	< 500 (<500,500)
Burundi	13824	215	··	··	··	··	··	··	··	··	··	··
Cameroon	34419	231	2·8 (1·8, 3·9)	1000 (500,1500)	11·3 (9·5, 13·2)	4000 (3500,4500)	··	··	12·9 (10·8, 15·1)	4500 (3500,5000)	2·3 (0·6, 5·0)	1000 (<500,1500)
Cabo Verde	2700	676	··	··	··	··	··	··	··	··	··	··
Central African Republic	2678	100	··	··	··	··	··	··	··	··	··	··
Chad	9589	111	··	··	··	··	··	··	··	··	··	··
Comoros	422	89	··	··	··	··	··	··	··	··	··	··
Côte d'Ivoire	27149	177	··	··	4·7 (4·0, 5·4)	1500 (1000,1500)	··	··	··	··	5·6 (4·4, 7·1)	1500 (1000,2000)
Democratic Republic of the Congo	44536	92	··	··	6·1 (4·9, 7·4)	2500 (2000,3500)	··	··	··	··	22·7 (7·8, 42·5)	10000 (3500,19000)
Djibouti	750	105	··	··	··	··	··	··	··	··	··	··
Equatorial Guinea	500	64	NK	NK	··	··	··	··	··	··	··	··
Eritrea**	··	··	NK	NK	··	··	··	··	··	··	··	··
Eswatini	3405	468	··	··	33·4 (28·9, 38·1)	1000 (1000,1500)	··	··	··	··	··	··
Ethiopia	110000	166	5·6 (3·9, 7·6)	6000 (4500,8500)	3·1 (2·6, 3·5)	3500 (3000,4000)	1·8 (0·1, 5·0)	2000 (<500,5500)	7·3 (5·6, 9·1)	8000 (6000,10000)	2·3 (1·2, 3·9)	2500 (1500,4500)
Gabon	5501	394	··	··	··	··	··	··	··	··	··	··
Gambia	543	38	··	··	··	··	··	··	··	··	··	··
Ghana	14262	73	17·1 (16·2, 18·0)	2500 (2500,2500)	7·3 (5·0, 10·2)	1000 (500,1500)	14·4 (12·2, 16·9)	2000 (1500,2500)	16·9 (11·8, 22·7)	2500 (1500,3000)	0·5 (0·0, 1·7)	< 500 (<500,<500)
Guinea	5549	75									2·5 (1·6, 3·5)	< 500 (<500,<500)
Guinea-Bissau	596	58	··	··	··	··	··	··	··	··	··	··
Kenya	60000	193	··	··	9·6 (7·8, 11·6)	6000 (4500,7000)	··	··	··	··	10·2 (5·9, 15·5)	6000 (3500,9500)
Lesotho	2216	162	3·3 (0·7, 7·3)	< 500 (<500,<500)	··	··	··	··	··	··	··	··
Liberia	3000	104	33·0 (24·1, 42·6)	1000 (500,1500)	5·6 (3·9, 7·6)	< 500 (<500,<500)	0·8 (0·2, 1·8)	< 500 (<500,<500)	13·3 (6·3, 22·2)	500 (<500,500)	··	··
Madagascar	30530	184	··	··	0·2 (0·0, 0·7)	< 500 (<500,<500)	··	··	··	··	11·4 (2·6, 24·5)	3500 (1000,7500)
Malawi	16536	154	··	··	26·2 (17·6, 36·2)	4500 (3000,6000)	0·0 (0·0, 1·3)	< 500 (<500,<500)	3·5 (1·0, 7·3)	500 (<500,1000)	1·0 (0·6, 1·6)	< 500 (<500,500)
Mali	8670	79	··	··	··	··	··	··	··	··	··	··
Mauritania	2826	112	NK	NK	··	··	··	··	··	··	··	··
Mauritius	2755	298	··	··	··	··	··	··	··	··	··	··
Mozambique	22000	128	··	··	12·0 (11·2, 12·9)	2500 (2500,3000)	··	··	··	··	3·3 (3·0, 3·7)	500 (500,1000)
Namibia	8900	589	··	··	··	··	··	··	··	··	··	··
Niger	13005	106	··	··	··	··	··	··	··	··	··	··
Nigeria	84011	73	0·0 (0·0, 0·6)	< 500 (<500,500)	6·5 (3·1, 10·9)	5500 (2500,9000)	13·0 (9·7, 16·8)	11000 (8000,14000)	18·1 (15·1, 21·4)	15000 (12500,18000)	1·8 (0·2, 4·5)	1500 (<500,3500)
Congo	1388	45	2·1 (0·0, 6·2)	< 500 (<500,<500)	8·3 (3·5, 14·8)	< 500 (<500,<500)	··	··	··	··	··	··
Rwanda	87621	1123	··	··	··	··	5·5 (5·3, 5·7)	5000 (4500,5000)	4·3 (4·1, 4·5)	4000 (3500,4000)	··	··
Sao Tome & Principe	300	239	··	··	··	··	··	··	··	··	··	··
Senegal	13185	142	··	··	1·8 (0·9, 2·9)	< 500 (<500,500)	0·6 (0·0, 1·8)	< 500 (<500,<500)	13·9 (10·4, 17·9)	2000 (1500,2500)	··	··
Seychelles	474	646	··	··	··	··	··	··	··	··	··	··
Sierra Leone	4453	92	··	··	2·3 (1·0, 4·1)	< 500 (<500,<500)	··	··	··	··	··	··
Somalia	2799	33	··	··	··	··	··	··	··	··	··	··
South Africa	157056	405	1·4 (0·7, 2·2)	2000 (1000,3500)	16·3 (6·7, 30·1)	25500 (10500,47000)	3·2 (1·9, 4·7)	5000 (3000,7500)	3·2 (1·9, 4·8)	5000 (3000,7500)	0·6 (0·5, 0·8)	1000 (1000,1000)
United Republic of Tanzania	32671	96	··	··	8·0 (5·6, 11·0)	2500 (2000,3500)	4·8 (2·9, 7·1)	1500 (1000,2500)	7·0 (4·7, 9·7)	2500 (1500,3000)	3·4 (1·4, 6·6)	1000 (500,2000)
Togo	4990	102	1·0 (0·5, 1·6)	< 500 (<500,<500)	5·8 (3·9, 8·0)	500 (<500,500)	0·3 (0·0, 1·2)	< 500 (<500,<500)	10·8 (7·8, 14·3)	500 (500,500)	··	··
Uganda	78539	322	··	··	10·9 (8·2, 13·9)	8500 (6500,11000)	0·8 (0·2, 1·6)	500 (<500,1500)	··	··	0·9 (0·3, 1·8)	500 (<500,1500)
Zambia	28225	264	··	··	23·5 (21·7, 25·5)	6500 (6000,7000)	21·7 (18·2, 25·3)	6000 (5000,7000)	··	··	1·8 (1·3, 2·4)	500 (500,500)
Zimbabwe	20997	236	··	··	··	··	··	··	··	··	··	··
**Middle East & North Africa**												
Algeria	94749	340	··	··	··	··	··	··	··	··	··	··
Bahrain	3485	310	··	··	··	··	··	··	··	··	··	··
Cyprus	966	112	7·8 (3·9, 12·9)	< 500 (<500,<500)	··	··	··	··	··	··	··	··
Egypt	120000	177	··	··	0·0 (0·0, 0·3)	< 500 (<500,500)	12·2 (9·5, 15·2)	14500 (11500,18500)	··	··	··	··
Iraq	73715	290	··	··	··	··	··	··	··	··	··	··
Israel	19756	371	··	··	··	··	··	··	··	··	··	··
Jordan	19140	270	··	··	··	··	··	··	··	··	··	··
Kuwait	5300	168	··	··	··	··	··	··	··	··	··	··
Lebanon	9254	264	12·3 (10·3, 14·6)	1000 (1000,1500)	0·2 (0·0, 0·7)	< 500 (<500,<500)	1·9 (0·6, 3·8)	< 500 (<500,500)	2·3 (0·8, 4·6)	< 500 (<500,500)	··	··
Libya	19103	428	··	··	17·5 (16·6, 18·5)	3500 (3000,3500)	17·9 (17·3, 18·6)	3500 (3500,3500)	6·8 (6·2, 7·5)	1500 (1000,1500)	··	··
Morocco	102653	421	··	··	18·0 (3·8, 40·6)	18500 (4000,41500)	··	··	··	··	··	··
Oman	1960	62	··	··	··	··	··	··	··	··	··	··
Occupied Palestinian territories	··	··	··	··	··	··	··	··	··	··	··	··
Qatar	2055	92	··	··	··	··	··	··	··	··	··	··
Saudi Arabia	68056	280	··	··	0·2 (0·1, 0·3)	< 500 (<500,<500)	0·3 (0·0, 0·8)	< 500 (<500,500)	··	··	··	··
South Sudan	8400	149	NK	NK	··	··	··	··	··	··	··	··
Sudan	21000	93	··	··	2·0 (0·4, 4·5)	500 (<500,1000)	··	··	··	··	··	··
Syrian Arab Republic	10599	102	··	··	0·0 (0·0, 0·4)	< 500 (<500,<500)	1·3 (0·4, 2·6)	< 500 (<500,500)	2·5 (1·2, 4·3)	500 (<500,500)	··	··
Tunisia	23484	289	··	··	··	··	··	··	··	··	··	··
Türkiye	371587	643	16·1 (14·3, 17·9)	59500 (53000,66500)	0·6 (0·1, 1·5)	2000 (500,5500)	3·8 (2·7, 5·4)	14000 (10000,20000)	3·3 (1·8, 5·2)	12500 (6500,19500)	0·1 (0·0, 0·2)	500 (<500,1000)
United Arab Emirates	9826	131	··	··	··	··	··	··	··	··	··	··
Yemen	4268	23	··	··	··	··	··	··	··	··	··	··

**Notes:** References for studies included in pooled estimates are reported in Appendix 9. Study level data informing each pooled estimate are listed in Appendices 15.1-15.5. Data reporting on risk of bias for each study used in the pooled estimates are reported in Appendices 16.1-16.5. This table reports country-level estimates among total people incarcerated, country-level tables on females, males and combined (mixed estimates only) samples are reported in Appendices Table 9.3-9.8.
