# Supplementary material for: The global epidemiology of injecting drug use, HIV, viral hepatitis and tuberculosis among people who are incarcerated: a multistage systematic review
Source: Int J Drug Policy. Author manuscript; Available in PMC 2026 Apr 8. (PMC13058553; doi:10.1016/j.drugpo.2025.105062)
Supplement: 9 [file NIHMS2157186-supplement-9.docx]

## Appendix 16.4: JBI Prevalence Risk of Bias Assessment for included papers with HBV estimates

| **Country** | **Author** | **Year of Publication** | **Risk of Bias Score** | | | | | | | | | | **Reference** |
| --- | --- | --- | --- | --- | --- | --- | --- | --- | --- | --- | --- | --- | --- |
|  |  |  | **1** | **2** | **3** | **4** | **5** | **6** | **7** | **8** | **9** | **Total** |  |
| **Eastern Europe** |  |  |  |  |  |  |  |  |  |  |  |  |  |
| Armenia | Weilandt | 2007 | 1 | 0 | 1 | 1 | 0 | 1 | 1 | 1 | 0 | 6 | ^1^ |
| Azerbaijan | Kasumov | 2008 | 0 | 0 | 1 | 0 | 0 | 1 | 1 | 1 | 0 | 4 | ^2^ |
| Azerbaijan | Azbel | 2015 | 1 | 0 | 1 | 1 | 0 | 1 | 1 | 1 | 0 | 6 | ^3^ |
| Azerbaijan | Handanagic | 2015 | 0 | 0 | 1 | 1 | 0 | 1 | 1 | 1 | 0 | 5 | ^4^ |
| Bosnia & Herzegovina | Ravlija | 2014 | 1 | 0 | 1 | 0 | 0 | 1 | 1 | 1 | 0 | 5 | ^5^ |
| Czech Republic | Klusonová | 2004 | 0 | 0 | 1 | 0 | 0 | 1 | 1 | 1 | 0 | 4 | ^6^ |
| Hungary | Treso | 2012 | 1 | 0 | 1 | 1 | 0 | 1 | 1 | 0 | 0 | 5 | ^7^ |
| Hungary | Treso | 2012 | 1 | 0 | 1 | 0 | 0 | 1 | 1 | 1 | 0 | 5 | ^7^ |
| Romania | Nazare | 2011 | 0 | 0 | 0 | 1 | 0 | 1 | 1 | 1 | 0 | 4 | ^8^ |
| Ukraine | Azbel | 2013 | 1 | 0 | 1 | 1 | 0 | 1 | 1 | 1 | 0 | 6 | ^9^ |
| **Western Europe** |  |  |  |  |  |  |  |  |  |  |  |  |  |
| Belgium | Busschotts | 2021 | 1 | 0 | 1 | 0 | 0 | 1 | 1 | 1 | 0 | 5 | ^10^ |
| Croatia | Burek | 2010 | 1 | 0 | 0 | 1 | 0 | 1 | 1 | 1 | 0 | 5 | ^11^ |
| Croatia | Vilibic-Cavlek | 2011 | 1 | 0 | 0 | 1 | 0 | 1 | 1 | 1 | 0 | 5 | ^12^ |
| Croatia | Burek | 2010 | 1 | 0 | 1 | 1 | 0 | 1 | 1 | 1 | 0 | 6 | ^11^ |
| Denmark | Christensen | 2000 | 0 | 0 | 1 | 0 | 0 | 1 | 1 | 1 | 0 | 4 | ^13^ |
| England and Wales | Morey | 2018 | 0 | 0 | 1 | 0 | 0 | 1 | 1 | 1 | 0 | 4 | ^14^ |
| England and Wales | Kirwan | 2011 | 1 | 0 | 1 | 1 | 0 | 1 | 1 | 1 | 0 | 6 | ^15^ |
| Finland | Rautanen | 2024 | 1 | 0 | 1 | 1 | 0 | 1 | 1 | 1 | 0 | 6 | ^16^ |
| Finland | Rautanen | 2024 | 1 | 0 | 1 | 1 | 0 | 1 | 1 | 0 | 0 | 5 | ^16^ |
| Finland | Rautanen | 2024 | 1 | 0 | 0 | 1 | 0 | 1 | 1 | 0 | 0 | 4 | ^16^ |
| France | Verneuil | 2008 | 0 | 0 | 1 | 1 | 0 | 1 | 1 | 1 | 0 | 5 | ^17^ |
| France | Jacomet | 2016 | 1 | 0 | 1 | 1 | 0 | 1 | 1 | 1 | 0 | 6 | ^18^ |
| France | Michault | 2000 | 0 | 0 | 0 | 1 | 0 | 1 | 1 | 1 | 0 | 4 | ^19^ |
| France | Lelievre | 2020 | 0 | 0 | 1 | 1 | 0 | 1 | 1 | 1 | 0 | 5 | ^20^ |
| France | Arrada | 2001 | 0 | 0 | 1 | 1 | 0 | 1 | 1 | 1 | 0 | 5 | ^21^ |
| Ireland | Drummond | 2014 | 1 | 0 | 1 | 0 | 0 | 1 | 1 | 1 | 0 | 5 | ^22^ |
| Ireland | Drummond | 2014 | 1 | 0 | 1 | 0 | 0 | 1 | 1 | 1 | 0 | 5 | ^22^ |
| Italy | Giuliani | 2020 | 0 | 0 | 1 | 0 | 0 | 1 | 1 | 1 | 0 | 4 | ^23^ |
| Italy | Brandolini | 2013 | 0 | 0 | 1 | 1 | 0 | 1 | 1 | 1 | 0 | 5 | ^24^ |
| Italy | Giuliani | 2020 | 0 | 0 | 1 | 0 | 0 | 1 | 1 | 1 | 0 | 4 | ^23^ |
| Italy | Scelza | 2022 | 0 | 0 | 0 | 1 | 0 | 1 | 1 | 1 | 0 | 4 | ^25^ |
| Italy | Babudieri | 2005 | 1 | 0 | 1 | 1 | 0 | 1 | 1 | 1 | 0 | 6 | ^26^ |
| Portugal | Passadouro | 2004 | 0 | 0 | 1 | 1 | 0 | 1 | 1 | 1 | 0 | 5 | ^27^ |
| Portugal | Garcia | 2004 | 0 | 0 | 1 | 0 | 0 | 1 | 1 | 1 | 0 | 4 | ^28^ |
| Portugal | da Silva Marques | 2010 | 0 | 0 | 0 | 1 | 0 | 1 | 1 | 1 | 0 | 4 | ^29^ |
| Portugal | Carvalhana | 2014 | 0 | 0 | 1 | 0 | 0 | 1 | 1 | 1 | 0 | 4 | ^30^ |
| Spain | Ferrer-Castro | 2012 | 0 | 0 | 1 | 0 | 0 | 1 | 1 | 0 | 0 | 3 | ^31^ |
| Spain | Vicente-Alcalde | 2020 | 1 | 0 | 1 | 1 | 0 | 1 | 1 | 1 | 0 | 6 | ^32^ |
| Spain | Ferrer-Castro | 2012 | 0 | 0 | 1 | 0 | 0 | 1 | 1 | 1 | 0 | 4 | ^31^ |
| Spain | de la Hoya | 2011 | 1 | 0 | 1 | 1 | 0 | 1 | 1 | 1 | 0 | 6 | ^33^ |
| Spain | Cuadrado | 2018 | 0 | 0 | 1 | 1 | 0 | 1 | 1 | 1 | 0 | 5 | ^34^ |
| Sweden | Gahrton | 2019 | 1 | 0 | 1 | 1 | 0 | 1 | 1 | 1 | 0 | 6 | ^35^ |
| Switzerland | Baggio | 2020 | 0 | 0 | 1 | 0 | 0 | 1 | 1 | 0 | 0 | 3 | ^36^ |
| Switzerland | Wolff | 2011 | 0 | 0 | 1 | 1 | 0 | 1 | 1 | 1 | 0 | 5 | ^37^ |
| Switzerland | Gétaz | 2018 | 0 | 0 | 0 | 1 | 0 | 1 | 1 | 1 | 0 | 4 | ^38^ |
| Switzerland | Baggio | 2020 | 0 | 0 | 1 | 0 | 0 | 1 | 1 | 0 | 0 | 3 | ^36^ |
| Switzerland | Gétaz | 2018 | 0 | 0 | 0 | 1 | 0 | 1 | 1 | 1 | 0 | 4 | ^38^ |
| **East and South East Asia** |  |  |  |  |  |  |  |  |  |  |  |  |  |
| Indonesia | Arends | 2019 | 0 | 0 | 1 | 0 | 0 | 1 | 1 | 1 | 0 | 4 | ^39^ |
| Indonesia | Rey | 2018 | 0 | 0 | 0 | 0 | 0 | 1 | 1 | 1 | 0 | 3 | ^40^ |
| Philippines | Ong-Chu | 2016 | 0 | 0 | 1 | 1 | 0 | 1 | 1 | 1 | 0 | 5 | ^41^ |
| Philippines | Simbulan | 2001 | 0 | 0 | 0 | 1 | 0 | 1 | 1 | 1 | 0 | 4 | ^42^ |
| Thailand | Harnpariphan | 2022 | 0 | 0 | 1 | 0 | 0 | 1 | 1 | 1 | 0 | 4 | ^43^ |
| **South Asia** |  |  |  |  |  |  |  |  |  |  |  |  |  |
| India | Rana | 2015 | 0 | 0 | 1 | 0 | 0 | 1 | 1 | 1 | 0 | 4 | ^44^ |
| India | Ramamoorthy | 2016 | 0 | 0 | 1 | 1 | 0 | 1 | 1 | 1 | 0 | 5 | ^45^ |
| India | National AIDS Control Organization | 2022 | 1 | 0 | 1 | 1 | 0 | 1 | 1 | 1 | 0 | 6 | ^46^ |
| Iran (Islamic Republic of) | Khademi | 2019 | 1 | 0 | 1 | 1 | 0 | 1 | 1 | 1 | 0 | 6 | ^47^ |
| Iran (Islamic Republic of) | Moradi | 2018 | 1 | 0 | 1 | 0 | 0 | 1 | 1 | 1 | 0 | 5 | ^48^ |
| Iran (Islamic Republic of) | Ziaee | 2014 | 1 | 0 | 1 | 1 | 0 | 1 | 1 | 1 | 0 | 6 | ^49^ |
| Iran (Islamic Republic of) | Moradi | 2019 | 1 | 0 | 1 | 1 | 0 | 1 | 1 | 1 | 0 | 6 | ^50^ |
| Iran (Islamic Republic of) | Salem | 2013 | 1 | 0 | 1 | 0 | 0 | 1 | 1 | 1 | 0 | 5 | ^51^ |
| Iran (Islamic Republic of) | Nokhodian | 2012 | 0 | 0 | 0 | 1 | 0 | 1 | 1 | 1 | 0 | 4 | ^52^ |
| Iran (Islamic Republic of) | Khajedaluee | 2016 | 1 | 0 | 1 | 0 | 0 | 1 | 1 | 1 | 0 | 5 | ^53^ |
| Iran (Islamic Republic of) | Khajedaluee | 2016 | 1 | 0 | 1 | 1 | 0 | 1 | 1 | 1 | 0 | 6 | ^53^ |
| Iran (Islamic Republic of) | Khajedaluee | 2016 | 1 | 0 | 0 | 1 | 0 | 1 | 1 | 1 | 0 | 5 | ^53^ |
| Pakistan | Kazi | 2010 | 0 | 0 | 1 | 1 | 0 | 1 | 1 | 1 | 0 | 5 | ^54^ |
| Pakistan | Fayyaz | 2006 | 0 | 0 | 1 | 1 | 0 | 1 | 1 | 1 | 0 | 5 | ^55^ |
| Pakistan | Memon | 2012 | 0 | 0 | 1 | 0 | 0 | 1 | 1 | 1 | 0 | 4 | ^56^ |
| Pakistan | Khan | 2017 | 0 | 0 | 1 | 0 | 0 | 1 | 1 | 1 | 0 | 4 | ^57^ |
| Sri Lanka | Niriella | 2015 | 0 | 0 | 1 | 0 | 0 | 1 | 1 | 1 | 0 | 4 | ^58^ |
| **Central Asia** |  |  |  |  |  |  |  |  |  |  |  |  |  |
| Kyrgyzstan | Azbel | 2016 | 1 | 0 | 1 | 1 | 0 | 1 | 1 | 1 | 0 | 6 | ^59^ |
| **Latin America** |  |  |  |  |  |  |  |  |  |  |  |  |  |
| Argentina | Mendizabal | 2020 | 1 | 0 | 1 | 1 | 0 | 1 | 1 | 1 | 0 | 6 | ^60^ |
| Bolivia (Plurinational State of) | Villarroel-Torrico | 2018 | 0 | 0 | 1 | 1 | 0 | 1 | 1 | 1 | 0 | 5 | ^61^ |
| Bolivia (Plurinational State of) | Villarroel-Torrico | 2018 | 0 | 0 | 1 | 1 | 0 | 1 | 1 | 1 | 0 | 5 | ^62^ |
| Brazil | Catalan-Soares | 2000 | 0 | 0 | 0 | 0 | 0 | 1 | 1 | 1 | 0 | 3 | ^63^ |
| Brazil | Silva | 2017 | 1 | 0 | 1 | 0 | 0 | 1 | 1 | 1 | 0 | 5 | ^64^ |
| Brazil | Miranda | 2025 | 0 | 0 | 0 | 1 | 0 | 1 | 1 | 1 | 0 | 4 | ^65^ |
| Brazil | do Nascimento | 2020 | 0 | 0 | 1 | 0 | 0 | 1 | 1 | 1 | 0 | 4 | ^66^ |
| Brazil | Benedetti | 2020 | 1 | 0 | 0 | 1 | 0 | 1 | 1 | 1 | 0 | 5 | ^67^ |
| Brazil | Rezende | 2020 | 1 | 0 | 1 | 0 | 0 | 1 | 1 | 1 | 0 | 5 | ^68^ |
| Brazil | Barros | 2013 | 1 | 0 | 0 | 0 | 0 | 1 | 1 | 1 | 0 | 4 | ^69^ |
| Brazil | Machado | 2019 | 0 | 0 | 1 | 1 | 0 | 1 | 1 | 1 | 0 | 5 | ^70^ |
| Brazil | Guimarães | 2001 | 0 | 0 | 1 | 1 | 0 | 1 | 1 | 1 | 0 | 5 | ^71^ |
| Brazil | Rezende | 2020 | 1 | 0 | 1 | 0 | 0 | 1 | 1 | 0 | 0 | 4 | ^68^ |
| Brazil | Ferreto | 2018 | 1 | 0 | 1 | 0 | 0 | 1 | 1 | 1 | 0 | 5 | ^72^ |
| Brazil | Stief | 2010 | 0 | 0 | 1 | 0 | 0 | 1 | 1 | 1 | 0 | 4 | ^73^ |
| Brazil | de Gois | 2022 | 1 | 0 | 1 | 0 | 0 | 1 | 1 | 1 | 0 | 5 | ^74^ |
| Colombia | Sanchez-Vanegas | 2021 | 0 | 0 | 1 | 1 | 0 | 1 | 1 | 1 | 0 | 5 | ^75^ |
| Mexico | Bautista-Arredondo | 2015 | 1 | 0 | 1 | 1 | 0 | 1 | 1 | 0 | 0 | 5 | ^76^ |
| Mexico | Alvarado-Esquivel | 2005 | 0 | 0 | 0 | 1 | 0 | 1 | 1 | 1 | 0 | 4 | ^77^ |
| Mexico | M Gonzalez | 2011 | 0 | 0 | 1 | 1 | 0 | 1 | 1 | 1 | 0 | 5 | ^78^ |
| Mexico | Bautista-Arredondo | 2015 | 1 | 0 | 1 | 1 | 0 | 1 | 1 | 0 | 0 | 5 | ^76^ |
| Mexico | Bautista-Arredondo | 2015 | 1 | 0 | 1 | 1 | 0 | 1 | 1 | 0 | 0 | 5 | ^76^ |
| Mexico | Belaunzaran-Zamudio | 2017 | 1 | 0 | 1 | 0 | 0 | 1 | 1 | 1 | 0 | 5 | ^79^ |
| Mexico | Bautista-Arredondo | 2015 | 1 | 0 | 1 | 1 | 0 | 1 | 1 | 1 | 0 | 6 | ^76^ |
| Venezuela (Bolivarian Republic of) | Alcivar | 2020 | 0 | 0 | 0 | 1 | 0 | 1 | 1 | 1 | 0 | 4 | ^80^ |
| **North America** |  |  |  |  |  |  |  |  |  |  |  |  |  |
| United States of America | Macalino | 2004 | 0 | 0 | 1 | 0 | 0 | 1 | 1 | 1 | 0 | 4 | ^81^ |
| United States of America | Lincoln | 2006 | 0 | 0 | 1 | 0 | 0 | 1 | 1 | 1 | 0 | 4 | ^82^ |
| United States of America | Solomon | 2004 | 1 | 0 | 1 | 0 | 0 | 1 | 1 | 1 | 0 | 5 | ^83^ |
| United States of America | Hennessey | 2009 | 1 | 0 | 1 | 0 | 0 | 1 | 1 | 1 | 0 | 5 | ^84^ |
| United States of America | Baillargeon | 2009 | 1 | 0 | 1 | 0 | 0 | 1 | 1 | 1 | 0 | 5 | ^85^ |
| United States of America | Khan | 2005 | 0 | 0 | 1 | 1 | 0 | 1 | 1 | 1 | 0 | 5 | ^86^ |
| **Australasia** |  |  |  |  |  |  |  |  |  |  |  |  |  |
| Australia | Butler | 2017 | 1 | 0 | 1 | 1 | 0 | 1 | 1 | 1 | 0 | 6 | ^87^ |
| Australia | Butler | 2017 | 1 | 0 | 1 | 1 | 0 | 1 | 1 | 1 | 0 | 6 | ^87^ |
| Australia | Butler | 2017 | 1 | 0 | 1 | 0 | 0 | 1 | 1 | 1 | 0 | 5 | ^87^ |
| Australia | Butler | 2017 | 1 | 0 | 1 | 0 | 0 | 1 | 1 | 1 | 0 | 5 | ^87^ |
| Australia | Bah | 2024 | 1 | 0 | 1 | 1 | 0 | 1 | 1 | 1 | 0 | 6 | ^88^ |
| Australia | Butler | 2017 | 1 | 0 | 1 | 0 | 0 | 1 | 1 | 1 | 0 | 5 | ^87^ |
| Australia | Stoove | 2011 | 0 | 0 | 1 | 0 | 0 | 1 | 1 | 1 | 0 | 4 | ^89^ |
| Australia | Indig | 2010 | 1 | 0 | 0 | 1 | 0 | 1 | 1 | 1 | 0 | 5 | ^90^ |
| Australia | Indig | 2010 | 1 | 0 | 1 | 0 | 0 | 1 | 1 | 1 | 0 | 5 | ^90^ |
| Australia | Gilles | 2008 | 0 | 0 | 0 | 0 | 0 | 1 | 1 | 1 | 0 | 3 | ^91^ |
| Australia | Young | 2016 | 1 | 0 | 0 | 0 | 0 | 1 | 1 | 1 | 0 | 4 | ^92^ |
| Australia | Indig | 2010 | 1 | 0 | 1 | 1 | 0 | 1 | 1 | 1 | 0 | 6 | ^90^ |
| **Sub Saharan Africa** |  |  |  |  |  |  |  |  |  |  |  |  |  |
| Burkina Faso | Diendere | 2011 | 0 | 0 | 1 | 1 | 0 | 1 | 1 | 1 | 0 | 5 | ^93^ |
| Cameroon | Kowo | 2021 | 0 | 0 | 1 | 1 | 0 | 1 | 1 | 1 | 0 | 5 | ^94^ |
| Ethiopia | Kedebe | 2017 | 0 | 0 | 0 | 1 | 0 | 1 | 1 | 1 | 0 | 4 | ^95^ |
| Ethiopia | Kassa | 2021 | 0 | 0 | 1 | 1 | 0 | 1 | 1 | 1 | 0 | 5 | ^96^ |
| Ghana | Ghana AIDS Commission | 2013 |  | 0 | 1 | 0 | 0 | 1 | 1 | 1 | 0 | 4 | ^97^ |
| Ghana | Adjei | 2008 | 1 | 0 | 1 | 1 | 0 | 1 | 1 | 1 | 0 | 6 | ^98^ |
| Ghana | Adjei | 2006 | 0 | 0 | 1 | 0 | 0 | 1 | 1 | 1 | 0 | 4 | ^99^ |
| Ghana | Adjei | 2006 | 0 | 0 | 1 | 0 | 0 | 1 | 1 | 1 | 0 | 4 | ^99^ |
| Ghana | Ghana AIDS Commission | 2013 | 1 | 0 | 1 | 0 | 0 | 1 | 1 | 0 | 0 | 4 | ^97^ |
| Ghana | Adjei | 2006 | 0 | 0 | 1 | 0 | 0 | 1 | 1 | 1 | 0 | 4 | ^99^ |
| Malawi | Chimphambano | 2007 | 0 | 1 | 0 | 0 | 1 | 1 | 1 | 1 | 1 | 6 | ^100^ |
| Nigeria | Adoga | 2009 | 1 | 0 | 1 | 1 | 0 | 1 | 1 | 1 | 0 | 6 | ^101^ |
| Nigeria | Dan-Nwafor | 2021 | 0 | 0 | 1 | 0 | 0 | 1 | 1 | 1 | 0 | 4 | ^102^ |
| Rwanda | Umutesi | 2021 | 1 | 0 | 1 | 1 | 0 | 1 | 1 | 1 | 0 | 6 | ^103^ |
| Senegal | Jaquet | 2016 | 0 | 0 | 1 | 1 | 0 | 1 | 1 | 1 | 0 | 5 | ^104^ |
| South Africa | The Aurum Institute NICD | 2020 | 0 | 0 | 1 | 0 | 0 | 1 | 1 | 1 | 0 | 4 | ^105^ |
| South Africa | The Aurum Institute NICD | 2022 | 0 | 0 | 1 | 0 | 0 | 1 | 1 | 1 | 0 | 4 | ^105^ |
| United Republic of Tanzania | Dahoma | 2009 | 1 | 0 | 1 | 0 | 0 | 1 | 1 | 1 | 0 | 5 | ^106^ |
| Togo | Jaquet | 2016 | 0 | 0 | 1 | 1 | 0 | 1 | 1 | 1 | 0 | 5 | ^104^ |
| **Middle East & North Africa** |  |  |  |  |  |  |  |  |  |  |  |  |  |
| Lebanon | Mahfoud | 2010 | 0 | 0 | 1 | 0 | 0 | 1 | 1 | 1 | 0 | 4 | ^107^ |
| Libya | Ziglam | 2012 | 1 | 0 | 1 | 0 | 0 | 1 | 1 | 1 | 0 | 5 | ^108^ |
| Syrian Arab Republic | Kobeissi | 2014 | 0 | 0 | 1 | 0 | 0 | 1 | 1 | 1 | 0 | 4 | ^109^ |
| Türkiye | Kose | 2019 | 0 | 0 | 1 | 0 | 0 | 1 | 1 | 1 | 0 | 4 | ^110^ |
| Türkiye | Balci | 2012 | 1 | 0 | 1 | 0 | 0 | 1 | 1 | 1 | 0 | 5 | ^111^ |
| Türkiye | Sahin | 2022 | 1 | 0 | 1 | 0 | 0 | 1 | 1 | 1 | 0 | 5 | ^112^ |
| Türkiye | Keten | 2016 | 0 | 0 | 1 | 1 | 0 | 1 | 1 | 1 | 0 | 5 | ^113^ |

**References**

1. Weilandt C, Stöver H, Eckert J, Grigoryan G. Anonymous survey on infectious diseases and related risk behaviour among Armenian prisoners and prison staff. *International Journal of Prisoner Health* 2007.

2. V. Kasumov AK, D. Makhmudova, F. Juzbashov, S. Hasiev, S. Babazade, R. Sultanova, G. Kasumova, N. Kerimova. Prevalence of HIV, hepatitis and syphilis, and behavioural risk factors among most-at-risk groups in the Republic of Azerbaijan. In: Ministry of Health of the Republic of Azerbaijan Republican AIDS Centre, editor.; 2008.

3. Azbel L, Wickersham JA, Wegman MP, et al. Burden of substance use disorders, mental illness, and correlates of infectious diseases among soon-to-be released prisoners in Azerbaijan. *Drug and Alcohol Dependence* 2015.

4. Handanagic S. Report on the Integrated Bio-behavioural Surveillance Surveys among Key Populations in Azerbaijan, 2015. In: Ministry of Health of Republic of Azerbaijan WHO, WHO Collaborating Centre for HIV Surveillance, Zagreb Croatia, editor.; 2015.

5. Ravlija J, Vasilj I, Marijanovic I, Vasilj M. Risk behaviour of prison inmates in relation to HIV/STI. *Psychiatria Danubina* 2014.

6. Klusonová H, Stĕpánová V, Cízek J, Plísková L. [Viral hepatitis in users of addictive drugs in the Czech Republic]. *Epidemiologie, Mikrobiologie, Imunologie* 2004.

7. Treso B, Barcsay E, Tarjan A, et al. Prevalence and correlates of HCV, HVB, and HIV infection among prison inmates and staff, Hungary. *Journal of urban health : bulletin of the New York Academy of Medicine* 2012.

8. Nazare C, Girleanu I, Cojocariu-Salloum C, Trifan A. [Prevalence of chronic hepatitis B virus (HBV) infection in closed communities and risk behaviour]. *Revista Medico-chirurgicala a Societatii de Medici si Naturalisti din Iasi* 2011.

9. Azbel L, Wickersham JA, Grishaev Y, Dvoryak S, Altice FL. Burden of infectious diseases, substance use disorders, and mental illness among Ukrainian prisoners transitioning to the community. *PLoS One* 2013.

10. Busschots D, Kremer C, Bielen R, et al. A multicentre interventional study to assess blood-borne viral infections in Belgian prisons. *BMC Infectious Diseases* 2021.

11. Burek V, Horvat J, Butorac K, Mikulić R. Viral hepatitis B, C and HIV infection in Croatian prisons. *Epidemiology and Infection* 2010.

12. Vilibic-Cavlek T, Gjenero-Margan I, Retkovac B, et al. Sociodemographic characteristics and risk behaviors for HIV, hepatitis B and hepatitis C virus infection among Croatian male prisoners. *International Journal pf Prisoner Health* 2011; **7**(1): 28-31.

13. Christensen PB, Krarup HB, Niesters HGM, Norder H, Georgsen J. Prevalence and incidence of bloodborne viral infections among Danish prisoners. *European Journal of Epidemiology* 2000.

14. Morey S, Hamoodi A, Valappil M, et al. A universal offer of blood borne virus testing substantially increases diagnosis and treatment of hepatitis C in prisons. *Journal of Hepatology* 2018.

15. Kirwan P, Evans B, Brant L, Sentinel Surveillance Hepatitis T. Hepatitis C and B testing in English prisons is low but increasing. *Journal of Public Health* 2011.

16. Rautanen M, Harald, K, & Tyni, S. Health and Wellbeing of Prisoners 2023 The Wattu IV Prison Population Study Finland, 2024.

17. Verneuil L, Vidal JS, Bekolo RZ, et al. Prevalence and risk factors of the whole spectrum of sexually transmitted diseases in male incoming prisoners in France. *European Journal of Clinical Microbiology & Infectious Diseases* 2009.

18. Jacomet C, Guyot-Lénat A, Bonny C, et al. Addressing the challenges of chronic viral infections and addiction in prisons: the PRODEPIST study. *European Journal of Public Health* 2016.

19. Michault A, Faulques B, Sevadjan B, Troalen D, Marais A, Barau G. Prevalence of hepatitis A, B, C virus markers in Reunion (south hospital and Saint Pierre prison). [French]. *Bulletin de la Societe de pathologie exotique (1990)* 2000.

20. Lelievre C, Prissette G, Reuche AA, et al. Detection of sexually transmitted infections at the Amiens prison. State of play from February 2019 to May 2019. *Revue de Medecine Legale* 2020.

21. Arrada A, Zbar OZD, Vasseur V. Prevalence of HBV and HCV infections and incidence of HCV infection after 3, 6 and 12 months detention in La Sante prison, Paris. *Annales de medecine interne* 2001.

22. Drummond A, Codd M, Donnelly N, et al. Study on the prevalence of drug use, including intravenous drug use, and blood-borne viruses among the Irish prisoner population. *Dublin: National Advisory Committee on Drugs and Alcohol* 2014.

23. Giuliani R, Casigliani V, Fornili M, et al. HCV micro-elimination in two prisons in Milan, Italy: A model of care. *Journal of Viral Hepatitis* 2020.

24. Brandolini M, Novati, S., De Silvestri A, Tinelli C, Patruno SFA, Ranieri R, Seminari E. Prevalence and epidemiological correlates and treatment outcome of HCV infection in an Italian prison setting. *BMC Public Health* 2013.

25. Scelza G, Amato A, Pagano AM, et al. Effect of hepatitis C antiviral therapy on oral lichen planus and hyposalivation in inmates. *Annals of Gastroenterology* 2022.

26. Babudieri S, Longo B, Sarmati L, et al. Correlates of HIV, HBV, and HCV infections in a prison inmate population: results from a multicentre study in Italy. *Journal of Medical Virology* 2005.

27. Passadouro R. [Prevalence infections and risk factors due to HIV, Hepatitis B and C in a prison establishment in Leiria]. *Acta Medica Portuguesa* 2004.

28. Garcia A, Exposto F, Prieto E, Lopes M, Duarte A, da Silva RC. Association of Trichomonas vaginalis with sociodemographic factors and other STDs among females inmates in Lisbon. *International Journal of STD & AIDS* 2004.

29. da Silva Marques NM, Margalho R, Melo MJ, da Cunha JGS, Melico-Silvestre AA. Seroepidemiological survey of transmissible infectious diseases in a Portuguese prison establishment. *Brazilian Journal of Infectious Diseases* 2011.

30. Carvalhana S, Pinto R, Leitao J, et al. HCV and HBV prevalence in the population: Large disparity between hepatitis c in the general population, comparing with high risk groups. *United European Gastroenterology Journal* 2014.

31. Ferrer-Castro V, Crespo-Leiro MR, García-Marcos LS, et al. [Evaluation of needle exchange program at Pereiro de Aguiar prison (Ourense, Spain): ten years of experience]. *Revista Espanola de Sanidad Penitenciaria* 2012.

32. Vicente-Alcalde N, Tuells J, Egoavil CM, Ruescas-Escolano E, Altavilla C, Caballero P. Immunization Coverage of Inmates in Spanish Prisons. *International Journal of Environmental Research and Public Health* 2020.

33. de la Hoya PS, Marco A, Garcia-Guerrero J, Rivera A, Prevalhep Study Group. Hepatitis C and B prevalence in Spanish prisons. *European Journal of Clinical Microbiology & Infectious Diseases* 2011.

34. Cuadrado A, Llerena S, Cobo C, et al. Microenvironment Eradication of Hepatitis C: A Novel Treatment Paradigm. *American Journal of Gastroenterology* 2018.

35. Gahrton C, Westman G, Lindahl K, et al. Prevalence of Viremic hepatitis C, hepatitis B, and HIV infection, and vaccination status among prisoners in Stockholm County. *BMC Infectious Diseases* 2019.

36. Baggio S, Pala KC, Rieder JP, Tran NT, Wolff H, Getaz L. Infectious diseases in post-trial detention and comparisons with pre-trial detention: A study in Geneva, Switzerland. *Journal of Infection and Public Health* 2020.

37. Wolff H, Sebo P, Haller DM, et al. Health problems among detainees in Switzerland: a study using the ICPC-2 classification. *BMC Public Health* 2011.

38. Gétaz L, Casillas A, Siegrist CA, et al. Hepatitis B prevalence, risk factors, infection awareness and disease knowledge among inmates: a cross-sectional study in Switzerland's largest pre-trial prison. *Journal of Global Health* 2018.

39. Arends RM, Nelwan EJ, Soediro R, et al. Associations between impulsivity, risk behavior and HIV, HBV, HCV and syphilis seroprevalence among female prisoners in Indonesia: A cross-sectional study. *PLoS One* 2019.

40. Rey I, Saragih R, Effendi-Ys R, Sembiring J, Siregar G, Zain L. Profile of hepatitis B and C virus infection in prisoners in Lubuk Pakam correctional facilities. IOP Conference Series: Earth and Environmental Science; 2018: IOP Publishing; 2018. p. 012033.

41. Ong-Chu MC, Lao-Tan JY, Gabriel EA. Prevalence of hepatitis B and C and risk factors among prison inmates in Cebu, Philippines. *Hepatology International* 2016.

42. Simbulan NP, Aguilar AS, Flanigan T, Cu-Uvin S. High-risk behaviors and the prevalence of sexually transmitted diseases among women prisoners at the women state penitentiary in Metro Manila. *Social Science & Medicine* 2001.

43. Harnpariphan W, Han WM, Supanun R, et al. High Proportion of Blood-Borne and Sexually Transmitted Infections Among People Deprived of Liberty in a Central Male Prison in Thailand: A Cross-Sectional Study 2018–2019. *AIDS research and human retroviruses* 2022; **38**(5): 370-7.

44. Rana S, Girdgar N, Gill MK, Kumar AJIJRMS. Prevalence of hepatitis-B surface antigen among population of inmates in Tihar Jail, New Delhi. 2015; **3**: 100-4.

45. Ramamoorthy M, Venketeswaran A, Seenivasan P, et al. Risk factors and prevalence, hepatitis B virus and hepatitis C virus among prison inmates, Chennai, India, 2015. *International Journal of Infectious Diseases* 2016; **53**: 90.

46. National AIDS Control Organization. HIV Sentinel Surveillance Plus 2021, Central Prison Sites, 2022.

47. Khademi N, Shakiba E, Khodadost M, Khoramdad M. Seroprevalence and related risk behaviors of hepatitis C, hepatitis B and HIV infections among Male prisoners in Kermanshah, Iran. *Archives of Iranian Medicine* 2019.

48. Moradi G, Gouya MM, Zavareh FA, et al. Prevalence and risk factors for HBV and HCV in prisoners in Iran: a national bio-behavioural surveillance survey in 2015. *Tropical Medicine & International Health* 2018.

49. Ziaee M, Sharifzadeh G, Namaee MH, Fereidouni M. Prevalence of HIV and Hepatitis B, C, D Infections and Their Associated Risk Factors among Prisoners in Southern Khorasan Province, Iran. *Iranian Journal of Public Health* 2014.

50. Moradi G, Jafari S, Zarei B, et al. Prevalence and Risk Factors for Hepatitis B and Hepatitis C Exposure in Iranian Prisoners: A National Study in 2016. *Hepatitis Monthly* 2019.

51. Salem F, Hekmat S, Aghasadeghi MR, Javadi F, Gholami H, Mostafavi E. Prevalence and Risk Factors of Hepatitis B Virus Genotype D Amongst Inmates in Alborz Province, Iran: A Cross-Sectional Survey. *Jundishapur Journal of Microbiology* 2013.

52. Nokhodian Z, Yazdani MR, Yaran M, et al. Prevalence and risk factors of HIV, syphilis, hepatitis B and C among female prisoners in Isfahan, Iran. *Hepatitis Monthly* 2012.

53. Khajedaluee M, Babaei A, Vakili R, et al. Sero-prevalence of bloodborne tumor viruses (HCV, HBV, HTLV-I and KSHV infections) and related risk factors among prisoners in Razavi Khorasan province, Iran, in 2008. *Hepatitis Monthly* 2016.

54. Kazi AM, Shah SA, Jenkins CA, Shepherd BE, Vermund SH. Risk factors and prevalence of tuberculosis, human immunodeficiency virus, syphilis, hepatitis B virus, and hepatitis C virus among prisoners in Pakistan. *International Journal pf Infectious Diseases* 2010.

55. Fayyaz M, Qazi M, Ishaq M, Chaudhry G, Bukhari MJB. Frequency of hepatitis B and C seropositivity in prisoners. 2006; **22**: 55-8.

56. Memon AR, Shafique K, Memon A, Draz AU, Rauf MUA, Afsar S. Hepatitis B and C prevalence among the high risk groups of Pakistani population. A cross sectional study. *Archives of Public Health* 2012.

57. Khan MA, Ayub A, Ayub H, Shafique M, Rahman JA. A comparative study of Hepatitis B and C prevalence using ICT and elisa method in jail inmates. *Pakistan Journal of Medical and Health Sciences* 2017.

58. Niriella MA, Hapangama A, Luke H, Pathmeswaran A, Kuruppuarachchi K, de Silva HJ. Prevalence of hepatitis B and hepatitis C infections and their relationship to injectable drug use in a cohort of Sri Lankan prison inmates. *Ceylon Medical Journal* 2015.

59. Azbel L, Polonsky M, Wegman M, et al. Intersecting epidemics of HIV, HCV, and syphilis among soon-to-be released prisoners in Kyrgyzstan: Implications for prevention and treatment. *International Journal of Drug Policy* 2016.

60. Mendizabal M, Testa P, Rojas M, et al. Pilot study using the ECHO model to enhance linkage to care for patients with hepatitis C in the custodial setting. *Journal of Viral Hepatitis* 2020.

61. Villarroel-Torrico M, Montaño K, Flores-Arispe P, et al. Syphilis, human immunodeficiency virus, herpes genital and hepatitis B in a women's prison in Cochabamba, Bolivia: prevalence and risk factors. *Revista Espanola de Sanidad Penitenciaria* 2018.

62. Villarroel-Torrico M, Montano K, Flores-Arispe P, et al. Syphilis, human immunodeficiency virus, herpes genital and hepatitis B in a women's prison in Cochabamba, Bolivia: prevalence and risk factors. *Revista Espanola de Sanidad Penitenciaria* 2018.

63. Catalan-Soares BC, Almeida RT, Carneiro-Proietti AB. Prevalence of HIV-1/2, HTLV-I/II, hepatitis B virus (HBV), hepatitis C virus (HCV), Treponema pallidum and Trypanosoma cruzi among prison inmates at Manhuacu, Minas Gerais State, Brazil. *Revista da Sociedade Brasileira de Medicina Tropical* 2000.

64. De Sena Silva AA, De Araújo TME, Teles SA, De Lima Brito Magalhães R, Andrade ELR. Prevalence of Hepatitis B and associated factors in prisoners. *Acta paulista de enfermagem* 2017; **30**(1): 66-72.

65. Miranda AE, Vargas, P.M., Louis, M.E.S. & Viana, M.C. Sexually transmitted diseases among female prisoners in Brazil: prevalence and risk factors. *Sexually transmitted diseases* 2000; **27**(9): 491-5.

66. do Nascimento CT, Pena DZ, Giuffrida R, et al. Prevalence and epidemiological characteristics of inmates diagnosed with infectious diseases living in a region with a high number of prisons in Sao Paulo state, Brazil. *BMJ Open* 2020.

67. Benedetti MSG, Nogami ASA, da Costa BB, et al. Sexually transmitted infections in women deprived of liberty in Roraima, Brazil. *Revista de Saude Publica* 2020.

68. Rezende GR, Lago BV, Puga MA, et al. Prevalence, incidence and associated factors for HBV infection among male and female prisoners in Central Brazil: A multicenter study. *International Journal of Infectious Diseases* 2020.

69. Barros LAS, Pessoni GC, Teles SA, et al. Epidemiology of the viral hepatitis B and C in female prisoners of Metropolitan Regional Prison Complex in the State of Goias, Central Brazil. *Revista da Sociedade Brasileira de Medicina Tropical* 2013.

70. Machado F, Becker D, de Oliveira CF, Possuelo LG, Renner JDP. Seroprevalence of HIV, hepatitis B and C and syphilis infection in prisoners of the central region of Rio Grande do Sul, Brazil. *O Mundo da Saúde* 2019.

71. Guimarães T, Granato CF, Varella D, Ferraz ML, Castelo A, Kallás EG. High prevalence of hepatitis C infection in a Brazilian prison: identification of risk factors for infection. *The Brazilian Journal of Infectious Diseases* 2001.

72. Ferreto LED, Follador FAC, Coelho HC, et al. Prevalence and risk factors for hepatitis B infection in men in the penitentiary system in Parana, Brazil. *Journal of Viral Hepatitis* 2018.

73. Stief ACF, Martins RMB, de Andrade SMO, et al. Seroprevalence of hepatitis b virus infection and associated factors among prison inmates in state of mato grosso do sul, Brazil. *Revista da Sociedade Brasileira de Medicina Tropical* 2011.

74. de Gois JG, Guedes SJKO, Vieira AP, et al. Seroprevalence and factors associated with hepatitis B virus exposure in the incarcerated population from southern Brazil. *PLoS One* 2022.

75. Sanchez-Vanegas G, Rodriguez-Vallejo D, Pinzon-Duran AC, Reina-Cifuentes MA, Monterrosa-Blanco A, Tiga-Segura JA. Prevalence of syphilis, hepatitis B and human immunodeficiency virus in the male prison population in Bogota, Colombia in 2019. [Spanish]. *Infectio* 2020.

76. Bautista-Arredondo S, González A, Servan-Mori E, et al. A Cross-Sectional Study of Prisoners in Mexico City Comparing Prevalence of Transmissible Infections and Chronic Diseases with That in the General Population. *PLoS One* 2015.

77. Alvarado-Esquivel C, Sablon E, Martínez-García S, Estrada-Martínez S. Hepatitis virus and HIV infections in inmates of a state correctional facility in Mexico. *Epidemiology and Infection* 2005.

78. Gonzalez CAM, Ortiz BES, Aguilar MB, Gonzalez JDM. Risk factors and the seroprevalence of viral markers of hepatitis B (HVB) and hepatitis C (HCV) in high-risk groups in Chiapas. *Medwave* 2011.

79. Belaunzaran-Zamudio PF, Mosqueda-Gomez JL, Macias-Hernandez A, Rodríguez-Ramírez S, Sierra-Madero J, Beyrer C. Burden of HIV, Syphilis, and Hepatitis B and C Among Inmates in a Prison State System in Mexico. *AIDS Res Hum Retroviruses* 2017.

80. Alcivar JC, Zambrano MM, Madronero MG, et al. Sexually transmitted infections in inmates in Merida Venezuela. *INVESTIGACION CLINICA* 2020.

81. Macalino GE, Vlahov D, Sanford-Colby S, et al. Prevalence and Incidence of HIV, Hepatitis B Virus, and Hepatitis C Virus Infections Among Males in Rhode Island Prisons. [References]. *American journal of public health* 2004.

82. Lincoln T, Tuthill RW, DePietro SL. Viral hepatitis, risk behaviors, aminotransferase levels, and screening options at a county correctional center. *Journal of Correctional Health Care* 2006.

83. Solomon L, Flynn C, Muck K, Vertefeuille J. Prevalence of HIV, syphilis, hepatitis B, and hepatitis C among entrants to Maryland correctional facilities. *Journal of Urban Health* 2004.

84. Hennessey KA, Kim AA, Griffin V, Collins NT, Weinbaum CM, Sabin K. Prevalence of infection with hepatitis B and C viruses and co-infection with HIV in three jails: a case for viral hepatitis prevention in jails in the United States. *Journal of Urban Health* 2009.

85. Baillargen J, Snyder N, Soloway RD, et al. Hepatocellular Carcinoma Prevalence and Mortality in a Male State Prison Population. *Public Health Reports* 2009.

86. Khan AJ, Simard EP, Bower WA, et al. Ongoing transmission of hepatitis B virus infection among inmates at a state correctional facility. *American Journal of Public Health* 2005.

87. Butler TS, M. National Prison Entrants’ Bloodborne Virus and Risk Behaviour Survey Report: Kirby Institute 2017.

88. Bah R, Sheehan Y, Li X, et al. Prevalence of blood-borne virus infections and uptake of hepatitis C testing and treatment in Australian prisons: the AusHep study. *The Lancet Regional Health–Western Pacific* 2024; **53**.

89. Stoové M, Kirwan A. External component of the evaluation of drug policies and services and their subsequent effects on prisoners and staff within the Alexander Maconochie Centre. 2011.

90. Indig D, Topp L, Ross B, et al. 2009 NSW Inmate Health Survey: Key Findings Report. Sydney: Justice Health, 2010.

91. Gilles M, Swingler E, Craven C, Larson A. Prison health and public health responses at a regional prison in Western Australia. *Australia and New Zealand Journal of Public Health* 2008.

92. Young JT, Van Dooren K, Borschmann R, Kinner S. ACT detainee health and wellbeing survey 2016: Summary results: ACT government; 2017.

93. Diendéré EA, Tiéno H, Bognounou R, et al. Prevalence and risk factors associated with infection by human immunodeficiency virus, hepatitis B virus, syphilis and bacillary pulmonary tuberculosis in prisons in Burkina Faso. *Medecine Tropicale : revue du Corps de sante colonial* 2011.

94. Kowo MP, Andoulo FA, Sizimboue DT, et al. Seroprevalence of hepatitis B and associated factors among inmates: a cross sectional study in the Douala New Bell Prison, Cameroon. *Pan African Medical Journal* 2021.

95. Kebede W, Abdissa A, Seid Y, Mekonnen Z. Seroprevalence and risk factors of hepatitis B, hepatitis C and HIV infections among prisoners in Jimma Town, Southwest Ethiopia. *Asian Pacific Journal of Tropical Disease* 2017.

96. Kassa Y, Million Y, Biset S, Moges F. Hepatitis b and hepatitis c viral infections and associated factors among prisoners in northeast ethiopia. *Journal of Blood Medicine* 2021.

97. Commission GA. National Health and HIV Survey of Prison Inmates in Ghana. 2013.

98. Adjei AA, Armah HB, Gbagbo F, et al. Correlates of HIV, HBV, HCV and syphilis infections among prison inmates and officers in Ghana: A national multicenter study. *BMC Infectious Diseases* 2008.

99. Adjei AA, Armah HB, Gbagbo F, et al. Prevalence of human immunodeficiency virus, hepatitis B virus, hepatitis C virus and syphilis among prison inmates and officers at Nsawam and Accra, Ghana. *Journal of Medical Microbiology* 2006; **55**(5): 593-7.

100. Chimphambano C, Komolafe I, Muula A. Prevalence of HIV, HepBsAg and Hep C antibodies among inmates in Chichiri prison, Blantyre, Malawi. *Malawi Medical Journal* 2007; **19**(3): 107-10.

101. Adoga MP, Banwat EB, Forbi JC, et al. Human immunonodeficiency virus, hepatitis B virus and hepatitis C virus: sero-prevalence, co-infection and risk factors among prison inmates in Nasarawa State, Nigeria. *The Journal of Infection in Developing Countries* 2009.

102. Dan-Nwafor CC, Adeoye I, Aderemi K, et al. Serological markers and risk factors associated with Hepatitis B virus infection among Federal Capital Territory prison inmates, Nigeria: Should we be concerned? *PLoS One* 2021.

103. Umutesi J, Klett-Tammen C, Nsanzimana S, Krause G, Ott JJ. Cross-sectional study of chronic hepatitis B virus infection in Rwandan high-risk groups: Unexpected findings on prevalence and its determinants. *BMJ Open* 2021.

104. Jaquet A, Wandeler G, Tine J, et al. HIV infection, viral hepatitis and liver fibrosis among prison inmates in West Africa. *BMC Infectious Diseases* 2016.

105. The Aurum Institute NICD. Socio-behavioural and structural factors driving HIV/AIDS, STIs and Hepatitis B & C infections among inmates in Correctional Facilities, Johannesburg, 2020.

106. M. Dahoma EM, A. Othman, A. Seha, A. Abdullah. Predisposing sexual and drug related risk factors among prisoners in Zanzibar. International Aids Society (IAS) 2009. Cape Town; 2009.

107. Mahfoud Z, Kassak K, Kreidieh K, Shamra S, Ramia S. Prevalence of antibodies to human immunodeficiency virus (HIV), hepatitis B and hepatitis C and risk factors in prisoners in Lebanon. *The Journal of Infection in Developing Countries* 2010.

108. Ziglam H, Zorgani AA, Balouz A, Abudhe AH, Elahmer O. Prevalence of antibodies to human immunodeficiency virus, hepatitis B, and hepatitis C in prisoners in Libya. *Libyan Journal of Medicine* 2012.

109. Kobeissi L. The Integrated Bio-Behavioral Survey (IBBS) in Syria: 2013-2014. In: United Nations Development Program, editor.; 2014.

110. Kose S, Adar P, Gozaydin A, Kuzucu L, Akkoclu G. Hepatitis B and Hepatitis C in prisons: a prevalence study. *International Journal of Prisoner Health* 2019.

111. Balci E, Turker K, Senol V, Gunay O. Screening Indicators of Hepatitis A, Hepatitis B, Hepatitis C and HIV infections in Prisoners. *Viral Hepatitis Journal* 2012.

112. Sahin AM, Sahin AR, Gunduz A, Aktemur A, Uzun N. Prevalence of Hepatitis B virus and Hepatitis C virus among prison inmates in Istanbul, Turkey. *Annals of Clinical and Analytical Medicine* 2022.

113. Keten D, Ova ME, Keten HS, et al. The prevalence of hepatitis B and C among prisoners in Kahramanmaras, Turkey. *Jundishapur Journal of Microbiology* 2016.
