# Supplementary material for: The global epidemiology of injecting drug use, HIV, viral hepatitis and tuberculosis among people who are incarcerated: a multistage systematic review
Source: Int J Drug Policy. Author manuscript; Available in PMC 2026 Apr 8. (PMC13058553; doi:10.1016/j.drugpo.2025.105062)
Supplement: 11 [file NIHMS2157186-supplement-11.docx]

## Appendix 16.5: JBI Prevalence Risk of Bias Assessment for included papers with TB estimates

| **Country** | **Author** | **Year of Publication** | **Risk of Bias Score** | | | | | | | | | | **Reference** |
| --- | --- | --- | --- | --- | --- | --- | --- | --- | --- | --- | --- | --- | --- |
|  |  |  | **1** | **2** | **3** | **4** | **5** | **6** | **7** | **8** | **9** | **Total** |  |
| **Eastern Europe** |  |  |  |  |  |  |  |  |  |  |  |  |  |
| Bulgaria | Andreev | 2011 | 0 | 0 | 1 | 0 | 0 | 1 | 1 | 1 | 0 | 4 | ^1^ |
| Georgia | Aerts | 2000 | 1 | 0 | 1 | 0 | 0 | 1 | 1 | 1 | 0 | 5 | ^2^ |
| Poland | Pendzich | 2015 | 1 | 0 | 1 | 1 | 0 | 1 | 1 | 1 | 0 | 6 | ^3^ |
| Romania | Mahler | 2021 | 1 | 0 | 1 | 0 | 0 | 1 | 1 | 1 | 0 | 5 | ^4^ |
| Russian Federation | Slavuckij | 2002 | 1 | 0 | 1 | 0 | 0 | 1 | 1 | 1 | 0 | 5 | ^5^ |
| **Western Europe** |  |  |  |  |  |  |  |  |  |  |  |  |  |
| Greece | Karabela | 2010 | 0 | 0 | 1 | 0 | 0 | 1 | 1 | 1 | 0 | 4 | ^6^ |
| Malta | Muscat | 2022 | 0 | 0 | 1 | 0 | 0 | 1 | 1 | 1 | 0 | 4 | ^7^ |
| Spain | Martin | 2001 | 0 | 0 | 1 | 0 | 0 | 1 | 1 | 1 | 0 | 4 | ^8^ |
| Switzerland | Ritter | 2012 | 0 | 0 | 1 | 1 | 0 | 1 | 1 | 1 | 0 | 5 | ^9^ |
| **East and South East Asia** |  |  |  |  |  |  |  |  |  |  |  |  |  |
| China | Jiang | 2019 | 1 | 0 | 1 | 0 | 0 | 1 | 1 | 1 | 0 | 5 | ^10^ |
| Hong Kong | Leung | 2005 | 0 | 0 | 1 | 0 | 0 | 1 | 1 | 0 | 0 | 3 | ^11^ |
| Taiwan | Chiang | 2002 | 1 | 0 | 1 | 0 | 0 | 1 | 1 | 1 | 0 | 5 | ^12^ |
| Thailand | Buangoen | 2020 | 1 | 0 | 1 | 0 | 0 | 1 | 1 | 1 | 0 | 5 | ^13^ |
| Thailand | Morasert | 2018 | 0 | 0 | 1 | 1 | 0 | 1 | 1 | 1 | 0 | 5 | ^14^ |
| Thailand | Jittimanee | 2022 | 0 | 0 | 1 | 0 | 0 | 1 | 1 | 1 | 0 | 4 | ^15^ |
| Thailand | Sretrirutchai | 2002 | 1 | 0 | 1 | 0 | 0 | 1 | 1 | 1 | 0 | 5 | ^16^ |
| Thailand | Jittimanee | 2007 | 1 | 0 | 1 | 0 | 0 | 1 | 1 | 1 | 0 | 5 | ^17^ |
| **South Asia** |  |  |  |  |  |  |  |  |  |  |  |  |  |
| Bangladesh | Islam | 2013 | 0 | 0 | 1 | 0 | 0 | 1 | 1 | 0 | 0 | 3 | ^18^ |
| Bangladesh | Banu | 2015 | 0 | 0 | 1 | 0 | 0 | 1 | 1 | 1 | 0 | 4 | ^19^ |
| Bangladesh | Banu | 2010 | 0 | 0 | 1 | 0 | 0 | 1 | 1 | 1 | 0 | 4 | ^20^ |
| India | Dolla | 2018 | 0 | 0 | 1 | 0 | 0 | 1 | 1 | 1 | 0 | 4 | ^21^ |
| India | Kosambiya | 2022 | 0 | 0 | 1 | 1 | 0 | 1 | 1 | 1 | 0 | 5 | ^22^ |
| India | Prasad | 2017 | 1 | 0 | 1 | 0 | 0 | 1 | 1 | 1 | 0 | 5 | ^23^ |
| India | Bhatnagar | 2019 | 0 | 0 | 1 | 0 | 0 | 1 | 1 | 1 | 0 | 4 | ^24^ |
| Iran (Islamic Republic of) | Farhoudi | 2019 | 0 | 0 | 1 | 1 | 0 | 1 | 1 | 1 | 0 | 5 | ^25^ |
| Iran (Islamic Republic of) | Moosazadeh | 2012 | 1 | 0 | 1 | 1 | 0 | 1 | 1 | 1 | 0 | 6 | ^26^ |
| Nepal | Shrestha | 2019 | 0 | 0 | 1 | 0 | 0 | 1 | 1 | 1 | 1 | 5 | ^27^ |
| Pakistan | Rao | 2004 | 0 | 0 | 1 | 0 | 0 | 1 | 1 | 1 | 0 | 4 | ^28^ |
| Pakistan | Jamal | 2019 | 0 | 0 | 1 | 0 | 0 | 1 | 1 | 1 | 0 | 4 | ^29^ |
| Pakistan | Kakar | 2018 | 0 | 0 | 1 | 0 | 0 | 1 | 1 | 1 | 0 | 4 | ^30^ |
| **Central Asia** |  |  |  |  |  |  |  |  |  |  |  |  |  |
| Tajikistan | Winetsky | 2014 | 1 | 0 | 1 | 1 | 0 | 1 | 1 | 1 | 0 | 6 | ^31^ |
| **Latin America** |  |  |  |  |  |  |  |  |  |  |  |  |  |
| Brazil | Santos | 2021 | 1 | 0 | 1 | 1 | 0 | 1 | 1 | 1 | 0 | 6 | ^32^ |
| Brazil | Valenca | 2015 | 0 | 0 | 1 | 1 | 0 | 1 | 1 | 1 | 0 | 5 | ^33^ |
| Brazil | Sanchez | 2013 | 0 | 0 | 1 | 0 | 0 | 1 | 1 | 1 | 0 | 4 | ^34^ |
| Brazil | Abrahao | 2006 | 0 | 0 | 1 | 0 | 0 | 1 | 1 | 1 | 0 | 4 | ^35^ |
| Brazil | Sanchez | 2005 | 0 | 0 | 1 | 0 | 0 | 1 | 1 | 1 | 0 | 4 | ^36^ |
| Brazil | Pedro | 2011 | 1 | 1 | 1 | 0 | 1 | 1 | 1 | 0 | 0 | 6 | ^37^ |
| Brazil | Perreira | 2013 | 0 | 0 | 1 | 0 | 0 | 1 | 1 | 1 | 0 | 4 | ^38^ |
| Brazil | Pelissari | 2018 | 0 | 0 | 1 | 0 | 0 | 1 | 1 | 1 | 0 | 4 | ^39^ |
| Brazil | Sanchez | 2009 | 0 | 0 | 1 | 1 | 0 | 1 | 1 | 1 | 0 | 5 | ^40^ |
| Brazil | Sanchez | 2007 | 0 | 0 | 1 | 0 | 0 | 1 | 1 | 1 | 0 | 4 | ^41^ |
| Brazil | Estevan | 2013 | 0 | 0 | 1 | 0 | 0 | 1 | 1 | 1 | 0 | 4 | ^42^ |
| Brazil | Nogueira | 2012 | 0 | 0 | 1 | 0 | 0 | 1 | 1 | 1 | 0 | 4 | ^43^ |
| Brazil | Sanchez | 2007 | 0 | 0 | 1 | 0 | 0 | 1 | 1 | 1 | 0 | 4 | ^41^ |
| Brazil | Nogueira | 2010 | 1 | 0 | 1 | 0 | 0 | 1 | 1 | 1 | 0 | 5 | ^44^ |
| Brazil | Soares | 2023 | 1 | 0 | 1 | 1 | 0 | 1 | 1 | 1 | 0 | 6 | ^45^ |
| Brazil | Kuhleis | 2012 | 0 | 0 | 1 | 0 | 0 | 1 | 1 | 0 | 0 | 3 | ^46^ |
| Brazil | Sanchez | 2007 | 0 | 0 | 1 | 0 | 0 | 1 | 1 | 1 | 0 | 4 | ^41^ |
| Colombia | Guerra | 2019 | 0 | 0 | 1 | 1 | 0 | 1 | 1 | 1 | 0 | 5 | ^47^ |
| Colombia | Rueda | 2013 | 1 | 0 | 1 | 0 | 0 | 1 | 1 | 1 | 0 | 5 | ^48^ |
| Colombia | Castañeda-Hernández | 2012 | 0 | 1 | 1 | 1 | 1 | 1 | 1 | 1 | 0 | 7 | ^49^ |
| **North America** |  |  |  |  |  |  |  |  |  |  |  |  |  |
| United States | Nduaguba | 2010 | 0 | 0 | 1 | 0 | 0 | 1 | 1 | 1 | 0 | 4 | ^50^ |
| United States | Hung | 2003 | 1 | 0 | 1 | 0 | 0 | 1 | 1 | 1 | 0 | 5 | ^51^ |
| United States | White | 2001 | 0 | 0 | 1 | 1 | 0 | 1 | 1 | 1 | 0 | 5 | ^52^ |
| United States | White | 2001 | 0 | 0 | 1 | 0 | 0 | 1 | 1 | 1 | 0 | 4 | ^52^ |
| **Sub Saharan Africa** |  |  |  |  |  |  |  |  |  |  |  |  |  |
| Botswana | Wang | 2003 | 1 | 0 | 1 | 1 | 0 | 1 | 1 | 1 | 0 | 6 | ^53^ |
| Cameroon | Noeske | 2006 | 0 | 0 | 1 | 1 | 0 | 1 | 1 | 1 | 0 | 5 | ^54^ |
| Cameroon | Donkeng-Donfack | 2022 |  | 0 | 1 | 0 | 0 | 1 | 1 | 1 | 0 | 4 | ^55^ |
| Cameroon | Noeske | 2011 | 0 | 0 | 1 | 1 | 0 | 1 | 1 | 1 | 0 | 5 | ^56^ |
| Cote d'Ivoire | Seri | 2017 | 0 | 0 | 1 | 0 | 0 | 1 | 1 | 1 | 1 | 5 | ^57^ |
| Democratic Republic of the Congo | Kayomo | 2018 | 0 | 0 | 1 | 1 | 0 | 1 | 1 | 1 | 0 | 5 | ^58^ |
| Democratic Republic of the Congo | Kalonji | 2019 | 0 | 0 | 1 | 1 | 0 | 1 | 1 | 1 | 0 | 5 | ^59^ |
| Democratic Republic of the Congo | Kalonji | 2016 | 0 | 0 | 1 | 0 | 0 | 1 | 1 | 1 | 0 | 4 | ^60^ |
| Ethiopia | Zerdo | 2014 | 1 | 0 | 1 | 0 | 0 | 1 | 1 | 1 | 0 | 5 | ^61^ |
| Ethiopia | Tadesse | 2021 | 1 | 0 | 1 | 0 | 0 | 1 | 1 | 1 | 0 | 5 | ^62^ |
| Ethiopia | Sahle | 2019 | 0 | 0 | 1 | 1 | 0 | 1 | 1 | 1 | 0 | 5 | ^63^ |
| Ethiopia | Merid | 2018 | 0 | 0 | 1 | 0 | 0 | 1 | 1 | 1 | 0 | 4 | ^64^ |
| Ethiopia | Abebe | 2011 | 1 | 0 | 1 | 0 | 0 | 1 | 1 | 1 | 0 | 5 | ^65^ |
| Ethiopia | Agajie | 2018 | 1 | 0 | 1 | 0 | 0 | 1 | 1 | 1 | 0 | 5 | ^66^ |
| Ethiopia | Adane | 2016 | 1 | 0 | 1 | 0 | 0 | 1 | 1 | 1 | 0 | 5 | ^67^ |
| Ethiopia | Gebrecherkos | 2016 | 1 | 0 | 1 | 0 | 0 | 1 | 1 | 1 | 0 | 5 | ^68^ |
| Ethiopia | Adane | 2019 | 1 | 0 | 1 | 0 | 0 | 1 | 1 | 1 | 1 | 6 | ^69^ |
| Ethiopia | Addis | 2015 | 0 | 0 | 1 | 0 | 0 | 1 | 1 | 1 | 0 | 4 | ^70^ |
| Ghana | Kwabla | 2015 | 0 | 0 | 1 | 0 | 0 | 1 | 1 | 1 | 0 | 4 | ^71^ |
| Guinea | Bah | 2012 | 0 | 0 | 1 | 0 | 0 | 1 | 1 | 0 | 0 | 3 | ^72^ |
| Malawi | Kanyerere | 2012 | 1 | 0 | 1 | 0 | 0 | 1 | 1 | 1 | 0 | 5 | ^73^ |
| Malawi | Banda | 2009 | 1 | 0 | 1 | 0 | 0 | 1 | 1 | 1 | 0 | 5 | ^74^ |
| Malawi | Mangochi | 2022 | 0 | 0 | 1 | 1 | 0 | 1 | 1 | 1 | 0 | 5 | ^75^ |
| Nigeria | Chigbu | 2010 | 0 | 0 | 0 | 1 | 0 | 1 | 1 | 1 | 0 | 4 | ^76^ |
| Nigeria | Ekundayo | 2015 | 0 | 0 | 1 | 0 | 0 | 1 | 1 | 1 | 0 | 4 | ^77^ |
| South Africa | Velen | 2022 | 1 | 0 | 1 | 1 | 0 | 1 | 1 | 1 | 0 | 6 | ^78^ |
| South Africa | Telisinghe | 2014 | 0 | 0 | 1 | 1 | 0 | 1 | 1 | 1 | 0 | 5 | ^79^ |
| South Africa | Hanifa | 2015 | 0 | 0 | 1 | 1 | 0 | 1 | 1 | 1 | 0 | 5 | ^80^ |
| South Africa | Jordan | 2019 | 1 | 0 | 1 | 0 | 0 | 1 | 1 | 1 | 0 | 5 | ^81^ |
| South Africa | Kim | 2020 | 1 | 0 | 1 | 1 | 0 | 1 | 1 | 1 | 0 | 6 | ^82^ |
| South Africa | Baird | 2022 | 0 | 0 | 1 | 0 | 0 | 1 | 1 | 1 | 0 | 4 | ^83^ |
| South Africa | Stevenson | 2020 | 1 | 0 | 1 | 0 | 0 | 1 | 1 | 1 | 0 | 5 | ^84^ |
| United Republic of Tanzania | Angolwisye | 2011 | 1 | 1 | 1 | 0 | 1 | 1 | 1 | 1 | 0 | 7 | ^85^ |
| United Republic of Tanzania | Steiner | 2015 | 0 | 0 | 1 | 1 | 0 | 1 | 1 | 1 | 0 | 5 | ^86^ |
| Uganda | United Nations Office on Drugs and Crime | 2008 | 1 | 1 | 1 | 0 | 1 | 1 | 1 | 1 | 0 | 7 | ^87^ |
| Zambia | Habeenzu | 2007 | 1 | 0 | 1 | 0 | 0 | 1 | 1 | 1 | 0 | 5 | ^88^ |
| Zambia | Maggard | 2015 | 1 | 0 | 1 | 0 | 0 | 1 | 1 | 1 | 0 | 5 | ^89^ |
| Zambia | Kagujje | 2021 | 1 | 0 | 1 | 1 | 0 | 1 | 1 | 1 | 0 | 6 | ^90^ |
| **Middle East and North Africa** |  |  |  |  |  |  |  |  |  |  |  |  |  |
| Türkiye | Kiter | 2003 |  | 0 | 1 | 0 | 0 | 1 | 1 | 1 | 0 | 4 | ^91^ |

**References**

1. Andreev V, Karcheva A, Petrova K, Lazarova E. Tuberculosis in prison. *European Respiratory Journal* 2011.

2. Aerts A, Habouzit M, Mschiladze L, et al. Pulmonary tuberculosis in prisons of the ex-USSR state Georgia: Results of a nation-wide prevalence survey among sentenced inmates. *International Journal of Tuberculosis and Lung Disease* 2000.

3. Pendzich J, Maksymowicz-Mazur W, Pawlowska J, et al. Tuberculosis among the homeless and inmates kept in custody and in penitentiary institutions in the Silesia region. *Pneumonologia i Alergologia Polska* 2015.

4. Mahler B, De Vries G, Van Hest R, et al. Use of targeted mobile X-ray screening and computer-aided detection software to identify tuberculosis among high-risk groups in Romania: Descriptive results of the E-DETECT TB active case-finding project. *BMJ Open* 2021.

5. Slavuckij A, Sizaire V, Lobera L, Matthys F, Kimerling ME. Decentralization of the DOTS programme within a Russian penitentiary system: How to ensure the continuity of tuberculosis treatment in pre‐trial detention centres. *The European Journal of Public Health* 2002; **12**(2): 94-8.

6. Karabela S, Papaventsis, D., Georgoulas, S., Nikolaou, S., Ioannidis, P., Konstantinidou, E., Sainti, A., Marinou, I. and Kanavaki, S Epidemiological monitoring of pulmonary tuberculosis in a correctional facility population, Athens, Greece, 2005-2009: P2080 *Clinical Microbiology & Infection* 2010.

7. Muscat K, Cremona C, Fenech TM, Abela M, Padovese V. Sexually transmitted infections epidemiology and risk assessment at the main correctional facility in Malta (2017-2019). *Journal of the European Academy of Dermatology and Venereology* 2022.

8. Sanchez VM, Guerra JM, Cayla JA, Rodriguez JC, Blanco MD, Alcoba M. Incidence of tuberculosis and the importance of treatment of latent tuberculosis infection in a Spanish prison population. *International Journal of Tuberculosis and Lung Disease* 2001.

9. Ritter C, Elger BS. Prevalence of positive tuberculosis skin tests during 5 years of screening in a Swiss remand prison. *International Journal of Tuberculosis and Lung Disease* 2012.

10. Tony Y, Jiang S, Guan X, et al. Epidemic situation of tuberculosis in prisons in the central region of China. *American Journal of Tropical Medicine and Hygiene* 2019.

11. Leung CC, Chan CK, Tam CM, et al. Chest radiograph screening for tuberculosis in a Hong Kong prison. *International Journal of Tuberculosis and Lung Disease* 2005.

12. Chiang CY, Hsu CJ, Hsu PK, Suo J, Lin TP. Pulmonary tuberculosis in the Taiwanese prison population. *Journal of the Formosan Medical Association* 2002.

13. Buangoen A, Ingviya T. Characteristics and Xpert MTB/RIF assay results of prisoners with pulmonary tuberculosis, Songkhla Province, southern Thailand. *Journal of the Medical Association of Thailand* 2020.

14. Morasert T, Worapas W, Kaewmahit R, Uphala W. Prevalence and risk factors associated with tuberculosis disease in Suratthani Central Prison, Thailand. *International Journal of Tuberculosis and Lung Disease* 2018.

15. Jittimanee S, Namonta A, Charuenporn C. Systematic TB screening using WHO radiograph categorisation and care outcomes. *The international journal of tuberculosis and lung disease : the official journal of the International Union against Tuberculosis and Lung Disease* 2022.

16. Sretrirutchai S, Silapapojakul K, Palittapongarnpim P, Phongdara A, Vuddhakul V. Tuberculosis in Thai prisons: magnitude, transmission and drug susceptibility. *International Journal of Tuberculosis and Lung Disease* 2002.

17. Jittimanee SX, Ngamtrairai N, White MC, Jittimanee S. A prevalence survey for smear-positive tuberculosis in Thai prisons. *International Journal of Tuberculosis and Lung Disease* 2007.

18. Islam MR, Khatun R, Uddin MKM, et al. Yield of Two Consecutive Sputum Specimens for the Effective Diagnosis of Pulmonary Tuberculosis. *PLoS One* 2013.

19. Banu S, Rahman MT, Uddin MKM, et al. Effect of active case finding on prevalence and transmission of pulmonary tuberculosis in Dhaka Central Jail, Bangladesh. *PLoS One* 2015.

20. Banu S, Hossain A, Uddin MKM, et al. Pulmonary tuberculosis and drug resistance in Dhaka central jail, the largest prison in Bangladesh. *PLoS One* 2010.

21. Dolla CK, Dhanraj B, Malaisamy M, et al. Burden of pulmonary tuberculosis in modern prison: A cross sectional prevalence survey from south India. *Indian Journal of Tuberculosis* 2019.

22. Kosambiya JK, Vadgama P, Samudyatha UC, Rathod D, Buch R, Damor R. Active case finding of pulmonary tuberculosis and HIV infection among prisoners of South Gujarat: A cross sectional study. *Indian Journal of Tuberculosis* 2022.

23. Prasad BM, Thapa B, Chadha SS, et al. Status of Tuberculosis services in Indian Prisons. *International Journal of Infectious Diseases* 2017.

24. Bhatnagar T, Ralte M, Ralte L, Chawnglungmuana, Sundaramoorthy L, Chhakchhuak L. Intensified tuberculosis and HIV surveillance in a prison in Northeast India: Implementation research. *PLoS One* 2019.

25. Farhoudi B, Alinaghi SAS, Hosseini M, et al. Prevalence of tuberculosis in a prison in tehran by active case finding. *Infectious Disorders - Drug Targets* 2019.

26. Moosazadeh M, Amiresmaili MR, Parsaei MR, Ahmadi M, Jalahi H. Prevalence of Tuberculosis Among the Prisoners of Mazandaran. *Journal of Rafsanjan University of Medical Sciences* 2011; **10**(4): 309-16.

27. Shrestha G, Yadav DK, Gautam R, Mulmi R, Baral D, Pokharel PK. Pulmonary tuberculosis among male inmates in the largest prison of Eastern Nepal. *Tuberculosis research and treatment* 2019; **2019**(1): 3176167.

28. Rao NA. Prevalence of pulmonary tuberculosis in Karachi central prison. *Journal of the Pakistan Medical Association* 2004.

29. Jamal W, Azeemi K, Waqar M, Ikram K, Zaidi SA, Habib S. Active case finding for tuberculosis among prisoners in Karachi, Pakistan. European Respiratory Society; 2019.

30. Kakar N, Abbas F, Shafee M, Asmat T. Study on Accuracy and Efficiency of Molecular Diagnostic Techniques used for Tuberculosis and Analysis of Associated Risk Factors for Tuberculosis in Jail Inmates of Quetta, Pakistan. *Pakistan Journal of Zoology* 2018.

31. Winetsky DE, Almukhamedov O, Pulatov D, Vezhnina N, Dooronbekova A, Zhussupov B. Prevalence, risk factors and social context of active pulmonary tuberculosis among prison inmates in Tajikistan. *PLoS One* 2014.

32. Santos AdS, Oliveira RDd, Lemos EF, et al. Yield, Efficiency, and Costs of Mass Screening Algorithms for Tuberculosis in Brazilian Prisons. *Clinical Infectious Diseases* 2021.

33. Valença MS, Scaini JL, Abileira FS, Gonçalves CV, von Groll A, Silva PE. Prevalence of tuberculosis in prisons: risk factors and molecular epidemiology. *International Journal of Tuberculosis and Lung Disease* 2015.

34. Sanchez A, Massari V, Gerhardt G, et al. X ray screening at entry and systematic screening for the control of tuberculosis in a highly endemic prison. *BMC public health* 2013; **13**: 1-7.

35. Abrahao RMCM, Nogueira PA, Malucelli MIC. Tuberculosis in county jail prisoners in the western sector of the city of Sao Paulo, Brazil. *International Journal of Tuberculosis and Lung Disease* 2006.

36. Sanchez A, Gerhardt G, Natal S, et al. Prevalence of pulmonary tuberculosis and comparative evaluation of screening strategies in a Brazilian prison. *International Journal of Tuberculosis and Lung Disease* 2005.

37. Heloisa da Silveira Paro Pedro SMTN, Maria Izabel Ferreira Pereira, Maria do Rosário Assad Goloni, Fernanda Carina Pires, Fernanda Modesto Tolentino, Rosangela Siqueira Oliveira, Andrea Regina Baptista Rossit. Mycobacterium tuberculosis detection in the penitentiary system. *Revista de Patologia Tropical/Journal of Tropical Pathology,* 2011; **40**(4): 287-2.

38. Pereira CC, Borges TS, Daronco A, et al. Prevalence of Respiratory Symptoms and Active Tuberculosis in a Prison in the South of Brazil. *Revista de Epidemiologia e Controle de Infecção* 2013.

39. Pelissari DM, Kuhleis DC, Bartholomay P, et al. Prevalence and screening of active tuberculosis in a prison in the South of Brazil. *International Journal of Tuberculosis and Lung Disease* 2018.

40. Sanchez A, Larouze B, Espinola AB, et al. Screening for tuberculosis on admission to highly endemic prisons? The case of Rio de Janeiro State prisons. *International Journal of Tuberculosis and Lung Disease* 2009.

41. Sanchez AR, Massari V, Gerhardt G, et al. Tuberculosis in Rio de Janeiro prisons, Bra zil: An urgent public health problem. *Cadernos de Saude Publica* 2007.

42. Estevan AO, de Oliveira SMVL, Croda J. Active and latent tuberculosis in prisoners in the Central-West Region of Brazil. *Revista da Sociedade Brasileira de Medicina Tropical* 2013.

43. Nogueira PA, Abrahao RMCM, Galesi VMN. Tuberculosis and latent tuberculosis in prison inmates. *Revista de Saude Publica* 2012.

44. Nogueira PA, Abrahao RMCDM, Galesi VMN. Tuberculosis in prison system - Survey in two prisons in the State of Sao Paulo, Brazil, 2008. *American Journal of Respiratory and Critical Care Medicine Conference: American Thoracic Society International Conference, ATS* 2010.

45. Soares TR, de Oliveira RD, Liu YE, et al. Evaluation of chest X-ray with automated interpretation algorithms for mass tuberculosis screening in prisons: A cross-sectional study. *The Lancet Regional Health – Americas* 2023.

46. Kuhleis D, Ribeiro AW, Dalla Costa ER, et al. Tuberculosis in a southern Brazilian prison. *MEMORIAS DO INSTITUTO OSWALDO CRUZ* 2012.

47. Guerra J, Mogollon D, Gonzalez D, et al. Active and latent tuberculosis among inmates in La Esperanza prison in Guaduas, Colombia. *PLoS One* 2019.

48. Rueda ZV, Lopez L, Velez LA, et al. High incidence of tuberculosis, low sensitivity of current diagnostic scheme and prolonged culture positivity in four Colombian prisons. A cohort study. *PLoS One* 2013.

49. Castañeda-Hernández DM, Martínez-Ramírez JE, Bolivar-Mejía A, Rodríguez-Morales AJ. Differences in TB incidence between prison and general populations, Pereira, Colombia, 2010-2011. *Tuberculosis (Edinb)* 2013.

50. Nduaguba IP, Brannan G, Shubrook J. Evaluation of identifying tuberculosis infection and disease in a rural institutionalized population. *Osteopathic Family Physician* 2010.

51. Hung R, Shelton S, Rischitelli G. Risk factors for tuberculosis conversion in a state prison. *McGill Journal of Medicine* 2002.

52. Castle White M, Tulsky JP, Portillo CJ, Menendez E, Cruz E, Goldenson J. Tuberculosis prevalence in an urban jail: 1994 and 1998. *International Journal of Tuberculosis and Lung Disease* 2001.

53. Wang EA. Rapid assessment of tuberculosis in a large prison system--Botswana, 2002. *Morbidity and mortality weekly report* 2003.

54. Noeske J, Kuaban C, Amougou G, Piubello A, Pouillot R. Pulmonary tuberculosis in the Central Prison of Douala, Cameroon. *East African Medical Journal* 2006.

55. Donkeng-Donfack VF, Tchatchueng-Mbougua JB, Abanda NN, et al. A cost-benefit algorithm for rapid diagnosis of tuberculosis and rifampicin resistance detection during mass screening campaigns. *BMC Infectious Diseases* 2022.

56. Noeske J, Ndi N, Mbondi S. Controlling tuberculosis in prisons against confinement conditions: a lost case? Experience from Cameroon. *International Journal of Tuberculosis and Lung Disease* 2011.

57. Seri B, Koffi A, Danel C, et al. Prevalence of pulmonary tuberculosis among prison inmates: A cross-sectional survey at the Correctional and Detention Facility of Abidjan, Cote d'Ivoire. *PLoS One* 2017.

58. Kayomo MK, Hasker E, Aloni M, et al. Outbreak of tuberculosis and multidrug-resistant tuberculosis, Mbuji-Mayi central prison, democratic Republic of the Congo. *Emerging Infectious Diseases* 2018.

59. Kalonji GMP, Ngongo Okenge L, Ilunga-Ilunga F, Albert A, Giet D. [Factors associated with prison survival: Study in the Democratic Republic of Congo]. *Sante Publique* 2019.

60. Kalonji GMP, Connick GD, Ngongo LO, et al. Prevalence of tuberculosis and associated risk factors in the central prison of Mbuji-Mayi, DEmocratic republic of Congo. *Tropical Medicine and Health* 2016.

61. Zerdo Z, Medhin G, Worku A, Ameni G. Prevalence of pulmonary tuberculosis and associated risk factors in prisons of Gamo Goffa Zone, south Ethiopia: A cross-sectional study. *American Journal of Health Research* 2014; **2**(5): 291-7.

62. Tadesse M, Diriba G, Getahun M, et al. The Burdon and determinant of pulmonary tuberculosis in Ethiopian federal prison facilities. *International Journal of Infectious Diseases* 2020.

63. Sahle ET, Blumenthal J, Jain S, et al. Bacteriologically-confirmed pulmonary tuberculosis in an Ethiopian prison: Prevalence from screening of entrant and resident prisoners. *PLoS One* 2019.

64. Merid Y, Woldeamanuel Y, Abebe M, et al. High utility of active tuberculosis case finding in an Ethiopian prison. *International Journal of Tuberculosis and Lung Disease* 2018.

65. Abebe DS, Bjune G, Ameni G, Biffa D, Abebe F. Prevalence of pulmonary tuberculosis and associated risk factors in Eastern Ethiopian prisons. *International Journal of Tuberculosis and Lung Disease* 2011.

66. Agajie M, Disassa H, Birhanu M, Amentie M. Prevalence of pulmonary tuberculosis and associated factors in prisons of BenishangulGumuz region, Western Ethiopia. *International Journal of Social Relevance & Concern* 2018; **6**(9).

67. Adane K, Spigt M, Ferede S, Asmelash T, Abebe M, Dinant GJ. Half of Pulmonary Tuberculosis Cases Were Left Undiagnosed in Prisons of the Tigray Region of Ethiopia: Implications for Tuberculosis Control. *PLoS One* 2016.

68. Gebrecherkos T, Gelaw B, Tessema B. Smear positive pulmonary tuberculosis and HIV co-infection in prison settings of North Gondar Zone, Northwest Ethiopia. *BMC Public Health* 2016.

69. Adane K, Spigt M, Winkens B, Dinant GJ. Tuberculosis case detection by trained inmate peer educators in a resource-limited prison setting in Ethiopia: a cluster-randomised trial. *The Lancet Global Health* 2019.

70. Addis Z, Adem E, Alemu A, et al. Prevalence of smear positive pulmonary tuberculosis in Gondar prisoners, North West Ethiopia. *Asian Pacific Journal of Tropical Medicine* 2015.

71. Kwabla M, Ameme D, Nortey P. Pulmonary tuberculosis and its risk factors among inmates of a Ghanaian prison. *International Journal of Tropical Disease & Health* 2015; **9**(3): 1-10.

72. Bah H, Cisse FA, Camara LM, Diallo OH, Diallo M, Sow OY. Prevalence of tuberculosis in the prison population of Conakry, Guinea Republic. *Revue de Medecine Legale* 2012.

73. Kanyerere HS, Banda RP, Gausi F, et al. Surveillance of tuberculosis in Malawian prisons. *Public Health Action* 2012.

74. Banda HT, Gausi F, Harries AD, Salaniponi FM. Prevalence of smear-positive pulmonary tuberculosis among prisoners in Malawi: A national survey. *International Journal of Tuberculosis and Lung Disease* 2009.

75. Mangochi P, Bossard C, Catacutan C, et al. TB screening, prevention and treatment cascade in a Malawi prison. *The International Journal of Tuberculosis and Lung Disease* 2022; **26**(10): 956-62.

76. Chigbu LN, Iroegbu CU. Incidence and spread of Mycobacterium tuberculosis-associated infection among Aba Federal prison inmates in Nigeria. *Journal of Health, Population and Nutrition* 2010.

77. Ekundayo EO, Onuka O, Mustapha G, Geoffrey M. Active case finding of pulmonary tuberculosis among prison inmates in aba Federal prison, Abia state, Nigeria. *Advances in Infectious Diseases* 2015; **5**(01): 57.

78. Velen K, Sathar F, Hoffmann CJ, et al. Digital Chest X-Ray with Computer-aided Detection for Tuberculosis Screening within Correctional Facilities. *Annals of the American Thoracic Society* 2022.

79. Telisinghe L, Fielding KL, Malden JL, et al. High tuberculosis prevalence in a South African prison: the need for routine tuberculosis screening. *PLoS One* 2014.

80. Hanifa Y, Telisinghe L, Fielding KL, et al. The diagnostic accuracy of urine lipoarabinomannan test for tuberculosis screening in a South African correctional facility. *PLoS One* 2015.

81. Jordan AM, Podewils LJ, Castro KG, Zishiri V, Charalambous S. Prevalence and risk factors of tuberculosis disease in South African correctional facilities in 2015. *International Journal of Tuberculosis and Lung Disease* 2019.

82. Kim HY, Zishiri V, Page-Shipp L, et al. Symptom and digital chest X-ray TB screening in South African prisons: Yield and cost-effectiveness. *International Journal of Tuberculosis and Lung Disease* 2020.

83. Baird K, Said H, Koornhof HJ, Duse AG. Tuberculosis control at a South African correctional centre: Diagnosis, treatment and strain characterisation. *PLoS One* 2022; **17**(11-Nov): e0277459.

84. Stevenson KA, Podewils LJ, Zishiri VK, Castro KG, Charalambous S. HIV prevalence and the cascade of care in five South African correctional facilities. *PLoS One* 2020.

85. J Angolwisye FK, F Nichombe, M Minja, A Rachow, H Machibia, M Pletschette, P Clowes. First survey on TB and HIV prevalence in the prisons of the Mbeya region in Tanzania. *42nd World Conference on Lung Health of the International Union Against Tuberculosis and Lung Disease* 2011; (157).

86. Steiner A, Mangu C, van den Hombergh J, et al. Screening for pulmonary tuberculosis in a tanzanian prison and computer-aided interpretation of chest X-rays. *Public Health Action* 2015.

87. United Nations Office on Drugs and Crime. A Rapid Situation Assessment of HIV/STI/TB and Drug Abuse among Prisoners in Uganda Prisons Service, 2009.

88. Habeenzu C, Mitarai S, Lubasi D, et al. Tuberculosis and multidrug resistance in Zambian prisons, 2000-2001. *International Journal of Tuberculosis and Lung Disease* 2007.

89. Maggard KR, Hatwiinda S, Harris JB, et al. Screening for tuberculosis and testing for human immunodeficiency virus in Zambian prisons. *Bulletin of the World Health Organization* 2015.

90. Kagujje M, Somwe P, Hatwiinda S, et al. Cross-sectional assessment of tuberculosis and HIV prevalence in 13 correctional facilities in Zambia. *BMJ Open* 2021.

91. Kiter G, Arpaz S, Keskin S, Sezgin N, Budin D, Seref O. Tuberculosis in Nazilli District Prison, Turkey, 1997-2001. *International Journal of Tuberculosis and Lung Disease* 2003.
