# Supplementary material for: The global epidemiology of injecting drug use, HIV, viral hepatitis and tuberculosis among people who are incarcerated: a multistage systematic review
Source: Int J Drug Policy. Author manuscript; Available in PMC 2026 Apr 8. (PMC13058553; doi:10.1016/j.drugpo.2025.105062)
Supplement: 3 [file NIHMS2157186-supplement-3.docx]

## Appendix 15.3: Study Characteristics of included papers for HCV estimates

| **Country** | **Author** | **Year of Publication** | **Geographic area** | **Female data** | **Male data** | **Mixed data** | **Risk of Bias Total Score** | **Reference** |
| --- | --- | --- | --- | --- | --- | --- | --- | --- |
| **Eastern Europe** |  |  |  |  |  |  |  |  |
| Armenia | Weilandt | 2007 | National | No | Yes | No | 7 | ^1^ |
| Azerbaijan | Kasumov | 2008 | City | No | Yes | No | 5 | ^2^ |
| Azerbaijan | Azbel | 2015 | National | Yes | Yes | No | 7 | ^3^ |
| Azerbaijan | Čakalo | 2012 | National | No | Yes | No | 7 | ^4^ |
| Azerbaijan | Handanagic | 2015 | City | No | Yes | No | 6 | ^5^ |
| Bosnia & Herzegovina | Ravlija | 2014 | National | No | No | Yes | 6 | ^6^ |
| Bosnia & Herzegovina | Hodžić | 2017 | Facility | No | Yes | No | 6 | ^7^ |
| Bulgaria | Popov | 2011 | Facility | Yes | Yes | No | 5 | ^8^ |
| Bulgaria | Popov | 2013 | Subnational | No | No | Yes | 6 | ^9^ |
| Georgia | Harris | 2019 | National | No | No | Yes | 6 | ^10^ |
| Georgia | Bergen-Cico | 2017 | Subnational | No | No | Yes | 5 | ^11^ |
| Hungary | Vanya | 2017 | Subnational | No | No | Yes | 6 | ^12^ |
| Hungary | Treso | 2012 | National | No | No | Yes | 6 | ^13^ |
| Hungary | Werling | 2022 | National | No | No | Yes | 6 | ^14^ |
| Hungary | Werling | 2022 | National | No | No | Yes | 6 | ^15^ |
| Romania | Nazare | 2011 | Facility | No | No | Yes | 4 | ^16^ |
| Romania | Bivegete | 2019 | Subnational | No | No | Yes | 5 | ^17^ |
| Ukraine | Azbel | 2013 | National | No | No | Yes | 7 | ^18^ |
| **Western Europe** |  |  |  |  |  |  |  |  |
| Austria | Silbernagl | 2018 | Subnational | No | Yes | No | 5 | ^19^ |
| Austria | Silbernagl | 2018 | Subnational | No | Yes | No | 5 | ^19^ |
| Belgium | Todts | 2008 | National | No | No | Yes | 5 | ^20^ |
| Belgium | Pletteinckx | 2024 | Subnational | No | No | Yes | 6 | ^21^ |
| Belgium | Busschotts | 2021 | Subnational | Yes | Yes | No | 6 | ^22^ |
| Belgium | Todts | 2008 | National | No | No | Yes | 5 | ^20^ |
| Croatia | Burek | 2010 | National | Yes | Yes | No | 7 | ^23^ |
| Croatia | Burek | 2010 | National | Yes | Yes | No | 6 | ^23^ |
| Croatia | Vilibic-Cavlek | 2011 | Subnational | No | Yes | No | 6 | ^24^ |
| Denmark | Soholm | 2019 | Subnational | No | No | Yes | 7 | ^25^ |
| Denmark | Christensen | 2000 | Facility | No | Yes | No | 5 | ^26^ |
| England and Wales | Bhandari | 2020 | Subnational | No | No | Yes | 6 | ^27^ |
| England and Wales | Kirwan | 2011 | Subnational | Yes | Yes | Yes | 7 | ^28^ |
| England and Wales | Skipper | 2003 | Subnational | No | Yes | No | 5 | ^29^ |
| England and Wales | Morey | 2018 | Facility | No | No | Yes | 5 | ^30^ |
| England and Wales | Jack | 2018 | Subnational | No | No | Yes | 6 | ^31^ |
| England and Wales | Mahto | 2008 | Facility | Yes | No | No | 5 | ^32^ |
| England and Wales | Patel | 2016 | Facility | No | Yes | No | 4 | ^33^ |
| England and Wales | Connoley | 2020 | Facility | No | Yes | No | 5 | ^34^ |
| England and Wales | Allsop | 2021 | Facility | Yes | No | No | 5 | ^35^ |
| England and Wales | Connoley | 2020 | Facility | No | Yes | No | 5 | ^34^ |
| England and Wales | Aisyah | 2017 | City | No | No | Yes | 5 | ^36^ |
| England and Wales | Horne | 2004 | Facility | No | No | Yes | 5 | ^37^ |
| England and Wales | Duncan | 2013 | Facility | No | Yes | No | 4 | ^38^ |
| England and Wales | Mohamed | 2020 | Facility | No | Yes | No | 5 | ^39^ |
| England and Wales | Weild | 2000 | Multinational | Yes | Yes | No | 6 | ^40^ |
| Finland | Rautanen | 2023 | National | Yes | Yes | No | 4 | ^41^ |
| Finland | Viitanen | 2011 | Facility | Yes | Yes | No | 5 | ^42^ |
| Finland | Viitanen | 2011 | Subnational | Yes | Yes | No | 7 | ^42^ |
| France | Vergniol | 2014 | Subnational | No | No | Yes | 6 | ^43^ |
| France | Jacomet | 2016 | Subnational | No | No | Yes | 6 | ^44^ |
| France | Remy | 2006 | National | No | No | Yes | 6 | ^45^ |
| France | Izquierdo | 2019 | Facility | No | No | Yes | 6 | ^46^ |
| France | Semaille | 2013 | National | No | No | Yes | 5 | ^47^ |
| France | Reynaud-Maurupt | 2005 | Subnational | No | No | Yes | 5 | ^48^ |
| France | Lelievre | 2020 | Facility | No | Yes | No | 5 | ^49^ |
| France | Perrodeau | 2016 | Facility | No | No | Yes | 4 | ^50^ |
| France | Remy | 2021 | Facility | No | No | Yes | 5 | ^51^ |
| France | Remy | 2021 |  | No | No | Yes | 4 | ^52^ |
| France | Michault | 2000 | Facility | No | Yes | No | 5 | ^53^ |
| France | Remy | 2021 |  | No | No | Yes | 4 | ^52^ |
| France | Roux | 2014 | Subnational | No | No | Yes | 7 | ^54^ |
| France | Abel | 2018 | Facility | No | No | Yes | 4 | ^55^ |
| France | Perrodeau | 2016 | Facility | No | No | Yes | 4 | ^50^ |
| Germany | Schulte | 2009 | National | No | No | Yes | 6 | ^56^ |
| Germany | Lehmann | 2004 | Facility | No | Yes | No | 6 | ^57^ |
| Greece | Tourkochristou | 2020 | Facility | No | Yes | No | 5 | ^58^ |
| Iceland | Fridriksdottir | 2020 | National | No | No | Yes | 4 | ^59^ |
| Ireland | Wright | 2006 | National | Yes | No | No | 6 | ^60^ |
| Ireland | Crowley | 2019 | Facility | No | Yes | No | 6 | ^61^ |
| Ireland | Allwright | 2000 | National | Yes | Yes | No | 7 | ^62^ |
| Ireland | Long | 2001 | National | Yes | Yes | No | 7 | ^63^ |
| Ireland | Drummond | 2014 | National | Yes | Yes | No | 6 | ^64^ |
| Ireland | Drummond | 2014 | National | Yes | Yes | No | 6 | ^64^ |
| Ireland | Bivegete | 2019 | Subnational | No | No | Yes | 5 | ^17^ |
| Italy | Scelza | 2022 | City | Yes | Yes | No | 5 | ^65^ |
| Italy | Stasi | 2016 | Subnational | No | No | Yes | 7 | ^66^ |
| Italy | Marco | 2020 | Facility | Yes | Yes | No | 6 | ^67^ |
| Italy | Babudieri | 2005 | National | Yes | Yes | No | 7 | ^68^ |
| Italy | Sagnelli | 2012 | National | No | No | Yes | 5 | ^69^ |
| Italy | Brandolini | 2013 | Facility | Yes | Yes | Yes | 6 | ^70^ |
| Italy | Giuliani | 2020 | City | No | No | Yes | 4 | ^71^ |
| Italy | Masarone | 2020 | Subnational | No | No | Yes | 5 | ^72^ |
| Italy | Giuliani | 2020 | City | No | No | Yes | 4 | ^71^ |
| Italy | Ranieri | 2016 | City | No | No | Yes | 5 | ^73^ |
| Italy | Fiore | 2021 | National | No | No | Yes | 7 | ^74^ |
| Italy | Izzo | 2021 | Facility | Yes | Yes | No | 4 | ^75^ |
| Italy | Caruso | 2019 | Facility | No | No | Yes | 5 | ^76^ |
| Italy | Voller | 2011 | Subnational | No | Yes | No | 6 | ^77^ |
| Italy | Montella | 2005 | Facility | No | Yes | No | 5 | ^78^ |
| Italy | Ciccarese | 2020 | Facility | No | Yes | No | 6 | ^79^ |
| Malta | Muscat | 2022 | Facility | No | No | Yes | 4 | ^80^ |
| North Macedonia | Jovanovska | 2014 | Subnational | No | No | Yes | 6 | ^81^ |
| Northern Ireland | Danis | 2007 | Subnational | No | No | Yes | 6 | ^82^ |
| Norway | Hannula | 2021 | Facility | No | No | Yes | 5 | ^83^ |
| Portugal | Passadouro | 2004 | Facility | No | No | Yes | 6 | ^84^ |
| Portugal | Garcia | 2004 | Facility | Yes | No | No | 5 | ^85^ |
| Portugal | Liberal | 2017 | Facility | No | No | Yes | 5 | ^86^ |
| Portugal | Marques | 2011 | Facility | No | Yes | No | 5 | ^87^ |
| Portugal | Carvalhana | 2014 | Facility | No | No | Yes | 5 | ^88^ |
| Portugal | Barros | 2008 | Facility | Yes | No | No | 5 | ^89^ |
| Portugal | Gaspar | 2020 | Facility | No | Yes | No | 5 | ^90^ |
| Scotland | Champion | 2004 | Facility | No | Yes | No | 5 | ^91^ |
| Scotland | Taylor | 2012 | National | No | No | Yes | 7 | ^92^ |
| Scotland | Morrison | 2001 | Facility | No | Yes | No | 6 | ^93^ |
| Spain | Gonzalez | 2008 | Facility | Yes | No | No | 6 | ^94^ |
| Spain | Cabezas | 2020 | National | No | No | Yes | 6 | ^95^ |
| Spain | Lerena | 2016 | Facility | No | No | Yes | 5 | ^96^ |
| Spain | García-Guerrero | 2010 | National | No | No | Yes | 7 | ^97^ |
| Spain | Saiz de la Hoya | 2005 | Facility | No | No | Yes | 6 | ^98^ |
| Spain | Olivan | 2001 | Facility | No | No | Yes | 6 | ^99^ |
| Spain | Cabezas | 2020 | National | No | No | Yes | 6 | ^95^ |
| Spain | Cabezas | 2020 | National | No | No | Yes | 6 | ^95^ |
| Spain | Cabezas | 2020 | National | No | No | Yes | 6 | ^95^ |
| Spain | Vicente-Alcalde | 2020 | Subnational | No | No | Yes | 6 | ^100^ |
| Spain | Ferrer-Castro | 2012 | Facility | No | No | Yes | 5 | ^101^ |
| Spain | Gil-Sierra | 2019 | Subnational | No | No | Yes | 6 | ^102^ |
| Spain | de la Hoya | 2011 | National | Yes | Yes | Yes | 7 | ^103^ |
| Spain | Murcia | 2009 | Facility | No | No | Yes | 6 | ^104^ |
| Spain | Cuadrado | 2018 | Facility | No | No | Yes | 6 | ^105^ |
| Spain | Marcos | 2022 | Facility | No | Yes | No | 5 | ^106^ |
| Spain | Serroukh | 2022 | Facility | Yes | Yes | No | 5 | ^107^ |
| Sweden | Gahrton | 2019 | Subnational | Yes | Yes | No | 6 | ^108^ |
| Switzerland | Baggio | 2020 | City | No | Yes | No | 4 | ^109^ |
| Switzerland | Pala | 2018 | Facility | No | Yes | No | 6 | ^110^ |
| Switzerland | Moschetti | 2015 | Subnational | Yes | Yes | No | 7 | ^111^ |
| Switzerland | Baggio | 2020 | City | No | Yes | No | 4 | ^109^ |
| **East and South East Asia** |  |  |  |  |  |  |  |  |
| Indonesia | Arends | 2019 | Facility | Yes | No | No | 5 | ^112^ |
| Indonesia | Hetty | 2020 | City | No | No | Yes | 5 | ^113^ |
| Indonesia | Rey | 2018 | Facility | No | No | Yes | 4 | ^114^ |
| Taiwan | Lu | 2021 | Facility | No | No | Yes | 6 | ^115^ |
| Taiwan | Yang | 2020 | Facility | No | No | Yes | 4 | ^116^ |
| Thailand | Harnpariphan | 2022 | Facility | No | Yes | No | 6 | ^117^ |
| **South Asia** |  |  |  |  |  |  |  |  |
| Afghanistan | John Hopkins University | 2012 | Facility | No | No | Yes | 5 | ^118^ |
| Afghanistan | John Hopkins University | 2011 | Facility | No | No | Yes | 5 | ^119^ |
| Afghanistan | John Hopkins University | 2012 | Facility | No | No | Yes | 5 | ^118^ |
| Afghanistan | John Hopkins University | 2011 | Facility | No | No | Yes | 5 | ^119^ |
| Iran (Islamic Republic of) | Tyagi | 2018 | Facility | Yes | Yes | No | 5 | ^120^ |
| Iran (Islamic Republic of) | Kaur | 2022 | Subnational | No | No | Yes | 6 | ^121^ |
| Iran (Islamic Republic of) | Singh | 2017 | Subnational | Yes | Yes | No | 6 | ^122^ |
| Iran (Islamic Republic of) | Ramamoorthy | 2016 | Facility | Yes | Yes | No | 6 | ^123^ |
| Iran (Islamic Republic of) | Sharifi | 2019 | Facility | No | Yes | No | 6 | ^124^ |
| Iran (Islamic Republic of) | Moradi | 2019 | National | Yes | Yes | No | 7 | ^125^ |
| Iran (Islamic Republic of) | Seyedalinaghi | 2023 | Facility | No | Yes | No | 5 | ^126^ |
| Iran (Islamic Republic of) | Khajedaluee | 2016 | Subnational | Yes | Yes | Yes | 6 | ^127^ |
| Iran (Islamic Republic of) | Ziaee | 2014 | Subnational | Yes | Yes | No | 7 | ^128^ |
| Iran (Islamic Republic of) | Hariri | 2021 | Facility | No | No | Yes | 6 | ^129^ |
| Iran (Islamic Republic of) | Hariri | 2020 | Facility | No | Yes | No | 5 | ^130^ |
| Iran (Islamic Republic of) | Khademi | 2019 | Subnational | No | Yes | No | 7 | ^131^ |
| Iran (Islamic Republic of) | Moradi | 2018 | National | Yes | Yes | No | 6 | ^132^ |
| Iran (Islamic Republic of) | Nokhodian | 2012 | Facility | Yes | No | No | 5 | ^133^ |
| Iran (Islamic Republic of) | Sarkari | 2012 | Subnational | No | No | Yes | 6 | ^134^ |
| Iran (Islamic Republic of) | Moradi | 2021 | Subnational | Yes | Yes | No | 6 | ^135^ |
| Pakistan | Wali | 2019 | Facility | No | No | Yes | 6 | ^136^ |
| Pakistan | Kazi | 2010 | Facility | No | Yes | No | 6 | ^137^ |
| Pakistan | Pervaiz | 2012 | Subnational | Yes | Yes | No | 7 | ^138^ |
| Pakistan | Memon | 2012 | Facility | No | Yes | No | 5 | ^139^ |
| Pakistan | Gorar | 2010 | Subnational | No | No | Yes | 6 | ^140^ |
| Pakistan | Butt | 2010 | Facility | No | Yes | No | 5 | ^141^ |
| Pakistan | Khan | 2017 | Facility | No | Yes | No | 5 | ^142^ |
| Pakistan | Fayyaz | 2006 | Facility | No | No | Yes | 6 | ^143^ |
| Sri Lanka | Niriella | 2015 | Facility | Yes | Yes | No | 5 | ^144^ |
| **Central Asia** |  |  |  |  |  |  |  |  |
| Kyrgyzstan | Azbel | 2016 | National | No | No | Yes | 7 | ^145^ |
| **Latin America** |  |  |  |  |  |  |  |  |
| Argentina | Mendizabal | 2020 | National | Yes | Yes | No | 7 | ^146^ |
| Argentina | Adaszko | 2017 | Subnational | No | No | Yes | 7 | ^147^ |
| Brazil | Leal | 2022 | City | No | No | Yes | 7 | ^148^ |
| Brazil | Coelho | 2009 | Facility | No | No | Yes | 5 | ^149^ |
| Brazil | Falquetto | 2013 | Facility | No | Yes | No | 5 | ^150^ |
| Brazil | Machado | 2019 | Facility | Yes | No | No | 6 | ^151^ |
| Brazil | Pompilio | 2011 | Facility | No | Yes | No | 6 | ^152^ |
| Brazil | Guimarães | 2001 | Facility | Yes | No | No | 6 | ^153^ |
| Brazil | Miranda | 2000 | Subnational | No | Yes | No | 5 | ^154^ |
| Brazil | Ronchi | 2017 | Facility | No | Yes | No | 5 | ^155^ |
| Brazil | Felisberto | 2019 | City | No | Yes | No | 5 | ^156^ |
| Brazil | Santos Barros | 2013 | City | No | No | Yes | 4 | ^157^ |
| Brazil | Ferreto | 2021 | Facility | No | Yes | No | 7 | ^158^ |
| Brazil | Gonçalves | 2005 | Facility | No | No | Yes | 5 | ^159^ |
| Brazil | Catalan-Soares | 2000 | Facility | No | No | Yes | 4 | ^160^ |
| Brazil | do Nascimento | 2020 | Facility | No | Yes | No | 5 | ^161^ |
| Brazil | El Maerrawi | 2015 | Subnational | Yes | No | No | 6 | ^162^ |
| Brazil | Massad | 1999 | Subnational | Yes | Yes | Yes | 5 | ^163^ |
| Brazil | Leite | 2022 | Subnational | Yes | Yes | Yes | 5 | ^164^ |
| Brazil | Barros | 2013 | Facility | Yes | No | No | 5 | ^165^ |
| Brazil | Soares | 2022 | Facility | No | No | Yes | 5 | ^166^ |
| Brazil | Puga | 2017 | Subnational | No | No | Yes | 7 | ^167^ |
| Brazil | Santos | 2011 | City | No | No | Yes | 7 | ^168^ |
| Brazil | Strazza | 2004 | City | No | No | Yes | 6 | ^169^ |
| Brazil | da Rosa | 2012 | Subnational | Yes | Yes | Yes | 5 | ^170^ |
| Mexico | Belaunzaran-Zamudio | 2017 | Subnational | Yes | Yes | Yes | 5 | ^171^ |
| Mexico | Silverman-Retana | 2017 | Facility | No | No | Yes | 5 | ^172^ |
| Mexico | Bautista-Arredondo | 2012 | Facility | Yes | Yes | No | 5 | ^173^ |
| Mexico | Bautista-Arredondo | 2015 | Facility | Yes | No | No | 7 | ^174^ |
| Mexico | Bautista-Arredondo | 2015 | Facility | No | Yes | No | 7 | ^174^ |
| Mexico | Alvarado-Esquivel | 2005 | National | Yes | Yes | No | 5 | ^175^ |
| Mexico | M Gonzalez | 2011 | Subnational | No | No | Yes | 6 | ^176^ |
| Venezuela (Bolivarian Republic of) | Alcivar | 2020 | City | No | No | Yes | 5 | ^177^ |
| Venezuela (Bolivarian Republic of) | Monsalve-Castillo | 2009 | Facility | No | No | Yes | 5 | ^178^ |
| **North America** |  |  |  |  |  |  |  |  |
| Canada | Courtemanche | 2018 | Subnational | Yes | Yes | No | 4 | ^179^ |
| Canada | Courtemanche | 2018 | Subnational | Yes | Yes | No | 6 | ^179^ |
| Canada | Kronfli | 2019 | Facility | No | Yes | No | 5 | ^180^ |
| Canada | Poulin | 2007 | Subnational | Yes | Yes | Yes | 7 | ^181^ |
| Canada | Besney | 2018 | Facility | Yes | No | No | 4 | ^182^ |
| Canada | Calzavara | 2007 | Subnational | No | No | Yes | 7 | ^183^ |
| Canada | Ford | 2000 | Facility | No | Yes | No | 5 | ^184^ |
| Canada | Poulin | 2007 | Subnational | Yes | Yes | Yes | 7 | ^181^ |
| Canada | Calzavara | 2007 | Subnational | No | No | Yes | 6 | ^183^ |
| United States of America | Akiyama | 2017 | City | Yes | Yes | No | 6 | ^185^ |
| United States of America | Abe | 2019 | Facility | Yes | Yes | No | 6 | ^186^ |
| United States of America | Solomon | 2004 | Subnational | No | No | Yes | 6 | ^187^ |
| United States of America | Alvarez | 2014 | Facility | Yes | Yes | No | 6 | ^188^ |
| United States of America | Hoff | 2023 | Facility | Yes | Yes | Yes | 6 | ^189^ |
| United States of America | Phung | 2021 | Subnational | No | No | Yes | 6 | ^190^ |
| United States of America | Assoumou | 2019 | Subnational | No | No | Yes | 6 | ^191^ |
| United States of America | Bai | 2014 | City | Yes | Yes | No | 6 | ^192^ |
| United States of America | Sieck | 2011 | Facility | No | No | Yes | 5 | ^193^ |
| United States of America | Nijhawan | 2019 | Facility | No | No | Yes | 5 | ^194^ |
| United States of America | Cocoros | 2014 | Facility | No | No | Yes | 4 | ^195^ |
| United States of America | Larney | 2014 | Subnational | Yes | Yes | No | 6 | ^196^ |
| United States of America | Scott | 2011 | Subnational | Yes | Yes | No | 5 | ^197^ |
| United States of America | Larney | 2014 | Subnational | Yes | Yes | No | 6 | ^196^ |
| United States of America | Larney | 2014 | Subnational | Yes | Yes | No | 7 | ^196^ |
| United States of America | Ruiz | 2002 | Subnational | Yes | Yes | No | 5 | ^198^ |
| United States of America | Lincoln | 2006 | Facility | No | No | Yes | 5 | ^199^ |
| United States of America | Weant | 2012 | Subnational | No | No | Yes | 5 | ^200^ |
| United States of America | Magaldi | 2022 | Facility | No | No | Yes | 5 | ^201^ |
| United States of America | Spaulding | 2019 | Subnational | No | No | Yes | 6 | ^202^ |
| United States of America | Bailargeon | 2003 | Subnational | Yes | Yes | No | 6 | ^203^ |
| United States of America | Ruiz | 2002 | Subnational | Yes | Yes | No | 5 | ^198^ |
| United States of America | Macalino | 2004 | Facility | No | Yes | No | 5 | ^204^ |
| United States of America | Phung | 2021 | Subnational | No | No | Yes | 6 | ^190^ |
| United States of America | Chan | 2020 | City | No | No | Yes | 5 | ^205^ |
| United States of America | Leukefeld | 2015 | Subnational | Yes | No | No | 5 | ^206^ |
| United States of America | Alvarez | 2014 | Facility | Yes | Yes | No | 6 | ^188^ |
| United States of America | Larney | 2014 | Subnational | Yes | Yes | No | 6 | ^196^ |
| United States of America | Kuncio | 2015 | City | Yes | Yes | No | 5 | ^207^ |
| United States of America | Baillargeon | 2009 | Subnational | No | No | Yes | 5 | ^208^ |
| United States of America | Akiyama | 2016 | City | No | No | Yes | 5 | ^209^ |
| United States of America | Abe | 2018 | Facility | No | No | Yes | 4 | ^210^ |
| United States of America | Deb | 2022 | Subnational | Yes | Yes | No | 6 | ^211^ |
| United States of America | Macalino | 2005 | Facility | Yes | Yes | No | 4 | ^212^ |
| United States of America | Nijhawan | 2019 | Facility | No | No | Yes | 5 | ^194^ |
| United States of America | Phung | 2021 | Subnational | No | No | Yes | 6 | ^190^ |
| United States of America | Fox | 2005 | Subnational | Yes | Yes | No | 6 | ^213^ |
| United States of America | de la Flor | 2017 | Facility | No | No | Yes | 5 | ^214^ |
| United States of America | Baillargeon | 2003 | Subnational | Yes | Yes | No | 6 | ^203^ |
| United States of America | Nijhawan | 2019 | Facility | No | No | Yes | 5 | ^194^ |
| United States of America | Wenger | 2014 | Facility | Yes | Yes | No | 6 | ^215^ |
| **Australasia** |  |  |  |  |  |  |  |  |
| Australia | Indig | 2009 | National | No | Yes | No | 6 | ^216^ |
| Australia | Bah | 2024 | National | Yes | Yes | No | 7 | ^217^ |
| Australia | Butler | 2017 | National | Yes | Yes | No | 5 | ^218^ |
| Australia | Indig | 2010 | Subnational | Yes | Yes | No | 6 | ^219^ |
| Australia | Butler | 2017 | National | Yes | Yes | No | 6 | ^218^ |
| Australia | Young | 2016 | Subnational | No | No | Yes | 5 | ^220^ |
| Australia | Hellard | 2004 | Subnational | Yes | Yes | No | 7 | ^221^ |
| Australia | Miller | 2006 | Subnational | Yes | Yes | No | 6 | ^222^ |
| Australia | Butler | 2017 | National | Yes | Yes | No | 5 | ^218^ |
| Australia | Hajarizadeh | 2021 | Subnational | No | Yes | Yes | 7 | ^223^ |
| Australia | Murray | 2004 | Subnational | No | No | Yes | 5 | ^224^ |
| Australia | Butler | 2007 | Subnational | Yes | Yes | No | 5 | ^225^ |
| Australia | Snow | 2017 | Subnational | No | No | Yes | 6 | ^226^ |
| Australia | Gilles | 2008 | Facility | No | No | Yes | 3 | ^227^ |
| Australia | Butler | 2017 | National | Yes | Yes | No | 6 | ^218^ |
| Australia | Watkins | 2009 | Subnational | No | No | Yes | 5 | ^228^ |
| Australia | Miller | 2009 | Subnational | No | No | Yes | 6 | ^229^ |
| Australia | Justice Health | 2017 | Subnational | Yes | Yes | No | 6 | ^230^ |
| Australia | Reekie | 2014 | Subnational | No | No | Yes | 5 | ^231^ |
| Australia | Awofeso | 2000 | Subnational | No | No | Yes | 6 | ^232^ |
| Australia | Dore | 2020 | Subnational | No | No | Yes | 6 | ^233^ |
| Australia | Papaluca | 2018 | Subnational | No | No | Yes | 6 | ^234^ |
| Australia | Hajarizadeh | 2021 | Subnational | No | Yes | Yes | 7 | ^223^ |
| Australia | Indig | 2009 | National | No | Yes | No | 6 | ^216^ |
| Australia | Butler | 2017 | National | Yes | Yes | No | 5 | ^218^ |
| Australia | Butler | 1999 | Subnational | Yes | Yes | No | 6 | ^235^ |
| Australia | Hajarizadeh | 2021 | Subnational | No | Yes | Yes | 7 | ^223^ |
| Australia | Hockings | 2002 | Subnational | Yes | No | No | 7 | ^236^ |
| Australia | Hajarizadeh | 2021 | Subnational | No | Yes | Yes | 7 | ^223^ |
| Australia | Justice Health | 2017 | Subnational | Yes | Yes | No | 6 | ^230^ |
| Australia | Hajarizadeh | 2021 | Subnational | No | Yes | Yes | 7 | ^223^ |
| Australia | Stoove | 2011 | Facility | No | No | Yes | 4 | ^237^ |
| Australia | Indig | 2010 | Subnational | Yes | Yes | No | 5 | ^219^ |
| Australia | Butler | 1999 | Subnational | Yes | Yes | No | 6 | ^235^ |
| **Sub Saharan Africa** |  |  |  |  |  |  |  |  |
| Burkina Faso | Ouedraogo | 2015 | Facility | No | Yes | No | 5 | ^238^ |
| Ethiopia | Kassa | 2021 | Facility | Yes | Yes | No | 6 | ^239^ |
| Ethiopia | Kedebe | 2017 | Facility | No | No | Yes | 5 | ^240^ |
| Ghana | Adjei | 2007 | National | Yes | Yes | No | 7 | ^241^ |
| Ghana | Adjei | 2006 | National | No | No | Yes | 6 | ^242^ |
| Ghana | Adjei | 2006 | Subnational | No | No | Yes | 7 | ^243^ |
| Liberia | Jones | 2019 | Subnational | No | No | Yes | 6 | ^244^ |
| Malawi | Chimphambano | 2007 | Facility | Yes | Yes | No | 6 | ^245^ |
| Nigeria | Adoga | 2009 | Subnational | No | Yes | No | 7 | ^246^ |
| Nigeria | Okafor | 2020 | Facility | Yes | Yes | No | 4 | ^247^ |
| Rwanda | Umutesi | 2021 | National | No | No | Yes | 7 | ^248^ |
| Senegal | Ministry of Health and Social Action | 2020 | National | No | No | Yes | 7 | ^249^ |
| Senegal | Jaquet | 2016 | Facility | No | Yes | No | 6 | ^250^ |
| South Africa | The Aurum Institute NICD | 2022 | Facility | No | No | Yes | 5 | ^251^ |
| South Africa | The Aurum Institute NICD | 2020 | Facility | No | No | Yes | 5 | ^251^ |
| United Republic of Tanzania | Dahoma | 2009 | Subnational | No | No | Yes | 6 | ^252^ |
| Togo | Jaquet | 2016 | Facility | No | Yes | No | 6 | ^250^ |
| Uganda | United Nations Office on Drugs and Crime | 2008 | National | No | No | Yes | 8 | ^253^ |
| Zambia | Maggard | 2015 | National | Yes | Yes | No | 5 | ^254^ |
| **Middle East & North Africa** |  |  |  |  |  |  |  |  |
| Egypt | Mohamed | 2013 | Facility | No | No | Yes | 5 | ^255^ |
| Lebanon | Mahfoud | 2010 | Facility | No | Yes | No | 4 | ^256^ |
| Libya | Ziglam | 2012 | Subnational | No | Yes | No | 6 | ^257^ |
| Libya | Elahmer | 2012 | Subnational | No | Yes | No | 7 | ^258^ |
| Syrian Arab Republic | Kobeissi | 2014 | Facility | No | No | Yes | 5 | ^259^ |
| Türkiye | Balci | 2012 | Subnational | No | No | Yes | 6 | ^260^ |
| Türkiye | Ozger | 2017 | Subnational | Yes | Yes | Yes | 7 | ^261^ |
| Türkiye | Sahin | 2022 | Subnational | No | Yes | No | 6 | ^262^ |
| Türkiye | Keten | 2016 | Facility | No | No | Yes | 6 | ^263^ |

**References**

1. Weilandt C, Stöver H, Eckert J, Grigoryan G. Anonymous survey on infectious diseases and related risk behaviour among Armenian prisoners and prison staff. *International Journal of Prisoner Health* 2007.

2. V. Kasumov AK, D. Makhmudova, F. Juzbashov, S. Hasiev, S. Babazade, R. Sultanova, G. Kasumova, N. Kerimova. Prevalence of HIV, hepatitis and syphilis, and behavioural risk factors among most-at-risk groups in the Republic of Azerbaijan. In: Ministry of Health of the Republic of Azerbaijan Republican AIDS Centre, editor.; 2008.

3. Azbel L, Wickersham JA, Wegman MP, et al. Burden of substance use disorders, mental illness, and correlates of infectious diseases among soon-to-be released prisoners in Azerbaijan. *Drug and Alcohol Dependence* 2015.

4. Jurja-Ivana Čakalo SH. The report on results of a surveillance survey on knowledge, risks and prevalence of HIV and sexually and parenterally transmitted infections in most-at-risk populations in Azerbaijan. 2012.

5. Handanagic S. Report on the Integrated Bio-behavioural Surveillance Surveys among Key Populations in Azerbaijan, 2015. In: Ministry of Health of Republic of Azerbaijan WHO, WHO Collaborating Centre for HIV Surveillance, Zagreb Croatia, editor.; 2015.

6. Ravlija J, Vasilj I, Marijanovic I, Vasilj M. Risk behaviour of prison inmates in relation to HIV/STI. *Psychiatria Danubina* 2014.

7. Hodžić H, Bajramović A, Obradović Z, Mahmić-Kaknjo M. Intravenous drugs abuse as the main risk factor of increasing hepatitis C infection prevalence in prisoners in Zenica, Bosnia and Herzegovina. *Med Glas (Zenica)* 2017.

8. Popov G, Plochev K. Prevalence and correlates of hepatitis C virus infection among inmates of Bulgarian prisons. *Clinical Microbiology and Infection* 2011.

9. Popov G, Plochev K, Pekova L, Pishmisheva M, Popov T, Tchervenyakova T. Prevalence of viral hepatitis, human immunodeficiency virus and syphilis among inmates of Bulgarian prisons. *Journal of Hepatology* 2013.

10. Harris AM, Chokoshvili O, Biddle J, et al. An evaluation of the hepatitis C testing, care and treatment program in the country of Georgia's corrections system, December 2013 - April 2015. *BMC Public Health* 2019.

11. Bergen-Cico D, Sikharulidze K, Ivanashvili N, Ivanishvili M, Keshelava T. Hepatitis C Risk and Protective Factors Associated With Drug Policies in the Republic of Georgia. *World Medical and Health Policy* 2017.

12. Vanya M, Szili K, Magori K, Krisztina V. Skin diseases and sexually transmitted infection in a Hungarian prison. *Reviews and Research in Medical Microbiology* 2017.

13. Tresó B, Barcsay E, Tarján A, et al. Prevalence and correlates of HCV, HVB, and HIV infection among prison inmates and staff, Hungary. *J Urban Health* 2012.

14. Werling K, Hunyady B, Makara M, et al. Hepatitis C Screening and Treatment Program in Hungarian Prisons in the Era of Direct Acting Antiviral Agents. *Viruses* 2022.

15. Werling K, Makara M, Nemesi K, et al. Screening and treatment of hepatitis C virus in prisons: 10 years of experience. *Orvosi Hetilap* 2022.

16. Nazare C, Girleanu I, Cojocariu-Salloum C, Trifan A. [Characteristics of hepatitis C virus (HCV) infection in closed communities]. *Rev Med Chir Soc Med Nat Iasi* 2011.

17. Bivegete S, Ward Z, Walker J, et al. Comparison of costs of different HCV screening and linkage to care interventions across Europe. *Journal of Hepatology* 2020.

18. Azbel L, Wickersham JA, Grishaev Y, Dvoryak S, Altice FL. Burden of infectious diseases, substance use disorders, and mental illness among Ukrainian prisoners transitioning to the community. *PLoS One* 2013.

19. Silbernagl M, Slamanig R, Fischer G, Brandt L. Hepatitis C infection and psychiatric burden in two imprisoned cohorts: Young offenders and opioid-maintained prisoners. *Health Policy* 2018.

20. Todts S, Glibert P, Van Malderen S, Van Huyck C, Saliez V, Hogge M. Usage de drogues dans les prisons belges: monitoring des risques sanitaires. *Bruxelles: SPF Justice* 2008.

21. Plettinckx E HN, de Smet S, Gremeaux L, Dirkx N. Health, well-being and drug use among persons in prison - Belgian results of the PRS-20 project 2021-2023, 2023.

22. Busschots D, Kremer C, Bielen R, et al. A multicentre interventional study to assess blood-borne viral infections in Belgian prisons. *BMC Infectious Diseases* 2021.

23. Burek V, Horvat J, Butorac K, Mikulić R. Viral hepatitis B, C and HIV infection in Croatian prisons. *Epidemiology and Infection* 2010.

24. Vilibic-Cavlek T, Gjenero-Margan I, Retkovac B, et al. Sociodemographic characteristics and risk behaviors for HIV, hepatitis B and hepatitis C virus infection among Croatian male prisoners. *International Journal pf Prisoner Health* 2011; **7**(1): 28-31.

25. Soholm J, Holm DK, Mossner B, et al. Incidence, prevalence and risk factors for hepatitis C in Danish prisons. *PLoS One* 2019.

26. Christensen PB, Krarup HB, Niesters HGM, Norder H, Georgsen J. Prevalence and incidence of bloodborne viral infections among Danish prisoners. *European Journal of Epidemiology* 2000.

27. Bhandari R, Morey S, Hamoodi A, et al. High rate of hepatitis C reinfection following antiviral treatment in the North East England Prisons. *J Viral Hepat* 2020.

28. Kirwan P, Evans B, Brant L, Sentinel Surveillance Hepatitis T. Hepatitis C and B testing in English prisons is low but increasing. *Journal of Public Health* 2011.

29. Skipper C, Guy JM, Parkes J, Roderick P, Rosenberg WM. Evaluation of a prison outreach clinic for the diagnosis and prevention of hepatitis C: implications for the national strategy. *Gut* 2003.

30. Morey S, Hamoodi A, Jones D, et al. Increased diagnosis and treatment of hepatitis C in prison by universal offer of testing and use of telemedicine. *J Viral Hepat* 2019.

31. Jack K, Thomson B, Irving W. Outcomes of an opt-out strategy for Hepatitis C testing in the East Midlands prison estate. *Journal of Hepatology* 2018.

32. Mahto M, Zia S. Measuring the gap: from Home Office to the National Health Service in the provision of a one-stop shop sexual health service in a female prison in the UK. *Int J STD AIDS* 2008.

33. Patel S, Clarke B, Bird G. Hepatitis B and hepatitis c virus case finding in a medium security UK prison. *Canadian Journal of Gastroenterology and Hepatology Conference* 2016.

34. Connoley D, Francis-Graham S, Storer M, et al. Detection, stratification and treatment of hepatitis C-positive prisoners in the United Kingdom prison estate: Development of a pathway of care to facilitate the elimination of hepatitis C in a London prison. *J Viral Hepat* 2020.

35. Allsop C, McCullough F, Miller C, et al. Impact of a 'high intensity test and treat' initiative for Hcv in low newton prison. *Gut* 2021.

36. Aisyah DN, Shallcross L, Hayward A, et al. Hepatitis C among vulnerable populations: A seroprevalence study of homeless, people who inject drugs and prisoners in London. *J Viral Hepat* 2018.

37. Horne JA, Clements AJ, Drennan P, Stein K, Cramp ME. Screening for hepatitis C virus in the Dartmoor prison population: an observational study. *J Public Health (Oxf)* 2004.

38. Duncan S, Sherrard J. Experience of screening for hepatitis C in an oxfordshire prison. *Sexually Transmitted Infections Conference: STI and AIDS World Congress* 2013.

39. Mohamed Z, Al-Kurdi D, Nelson M, et al. Time matters: Point of care screening and streamlined linkage to care dramatically improves hepatitis C treatment uptake in prisoners in England. *Int J Drug Policy* 2020.

40. Weild AR, Gill ON, Bennett D, Livingstone SJ, Parry JV, Curran L. Prevalence of HIV, hepatitis B, and hepatitis C antibodies in prisoners in England and Wales: a national survey. *Commun Dis Public Health* 2000.

41. Rautanen M, Harald, K, & Tyni, S. The Health and Wellbeing of Finnish Prisoners 2023 (Wattu IV). *Finnish institute for health and welfare (THL) Report 007/2023 256 pages Helsinki 2023* 2023.

42. Viitanen P, Vartiainen H, Aarnio J, et al. Hepatitis A, B, C and HIV infections among Finnish female prisoners--young females a risk group. *J Infect* 2011.

43. Vergniol J, Capdepont M, El Aouadi S, et al. Prevalence of viral hepatitis and liver fibrosis in a population of incomers in French prisons. UCSASCAN study. *Hepatology* 2014.

44. Jacomet C, Guyot-Lénat A, Bonny C, et al. Addressing the challenges of chronic viral infections and addiction in prisons: the PRODEPIST study. *European Journal of Public Health* 2016.

45. Remy AJ. Hepatitis C in prison settings: Screening and therapy are improving - Comparative survey between 2000 and 2003. [French]. *Presse Medicale* 2006.

46. Izquierdo L, Mellon G, Buchaillet C, et al. Prevalence of hepatitis E virus and reassessment of HIV and other hepatitis virus seroprevalences among French prison inmates. *PLoS One* 2019.

47. Semaille C, Le Strat Y, Chiron E, et al. Prevalence of human immunodeficiency virus and hepatitis C virus among French prison inmates in 2010: a challenge for public health policy. *Euro surveillance : bulletin Europeen sur les maladies transmissibles = European communicable disease bulletin* 2013.

48. Reynaud-Maurupt C, Caer Y, Escaffre N, et al. High-dose buprenorphine substitution during incarceration - Management of opiate addicts. *Presse Medicale* 2005.

49. Lelievre C, Prissette G, Reuche AA, et al. Detection of sexually transmitted infections at the Amiens prison. State of play from February 2019 to May 2019. *Revue de Medecine Legale* 2020.

50. Perrodeau F, Pillot-Debelleix M, Vergniol J, et al. Optimizing hepatitis B vaccination in prison. *Med Mal Infect* 2016.

51. Remy AJ, Roy B, Hervet J. The 'prison zero hepatitits' project in france: a new pathway for hcv microelimination. *Heroin Addiction and Related Clinical Problems* 2021.

52. Remy AJ, Roy B, Hervet J, Bouchkira H. Lack of COVID-19 impact on managing hepatitis c in prison like the general population! *Hepatology* 2021.

53. Michault A, Faulques B, Sevadjan B, Troalen D, Marais A, Barau G. Prevalence of hepatitis A, B, C virus markers in Reunion (south hospital and Saint Pierre prison). [French]. *Bulletin de la Societe de pathologie exotique (1990)* 2000.

54. Roux P, Sagaon-Teyssier L, Lions C, Fugon L, Verger P, Carrieri MP. HCV seropositivity in inmates and in the general population: an averaging approach to establish priority prevention interventions. *BMJ Open* 2014.

55. Abel S, Cuzin L, Da Cunha S, et al. Reaching the WHO target of testing persons in jails in prisons will need diverse efforts and resources. *PLoS One* 2018.

56. Schulte B, Stover H, Thane K, Schreiter C, Gansefort D, Reimer J. Substitution treatment and HCV/HIV-infection in a sample of 31 German prisons for sentenced inmates. *International journal of prisoner health* 2009.

57. Lehmann M, Meyer MF, Monazahian M, Tillmann HL, Manns MP, Wedemeyer H. High rate of spontaneous clearance of acute hepatitis C virus genotype 3 infection. *J Med Virol* 2004.

58. Tourkochristou E, Beskos G, Kanaloupitis S, et al. Prevalence of anti-HCV antibodies and risk factors among prison inmates in Southwestern Greece. *Achaiki Iatriki* 2020; **39**: 23-8.

59. Fridriksdottir R, Ingibergsdotir, B, Frodjonsdottir, H, Alexiusdottir, K, Tomasdottir, A, Bjornsdottir, Th, Finnbogsdottir, A, Olafsdottir, B, Tyrfingsson, Th, Runarsdottir, V, Bergmann, OM, Bjornsson, ES, Johansson, B, Sigurdardottir, B, Heimisdottir, M, Olafsson, S & Gottfredsson, M. Sustained reduction in prevalence of hepatitis C viremia in the prison setting after 3rd year of TrapHepC (Treatment as Prevention for hepatitis C) program in Iceland. 2020.

60. Wright B, Duffy D, Curtin K, Linehan S, Monks S, Kennedy HG. Psychiatric morbidity among women prisoners newly committed and amongst remanded and sentenced women in the Irish prison system. *Irish journal of psychological medicine* 2006.

61. Crowley D, Lambert JS, Betts-Symonds G, et al. The seroprevalence of untreated chronic hepatitis C virus (HCV) infection and associated risk factors in male Irish prisoners: a cross-sectional study, 2017. *Euro Surveill* 2019.

62. Allwright S, Bradley F, Long J, Barry J, Thornton L, Parry JV. Prevalence of antibodies to hepatitis B, hepatitis C, and HIV and risk factors in Irish prisoners: results of a national cross sectional survey. *Bmj* 2000.

63. Long J, Allwright S, Barry J, et al. Prevalence of antibodies to hepatitis B, hepatitis C, and HIV and risk factors in entrants to Irish prisons: a national cross sectional survey. *Bmj* 2001.

64. Drummond A, Codd M, Donnelly N, et al. Study on the prevalence of drug use, including intravenous drug use, and blood-borne viruses among the Irish prisoner population. *Dublin: National Advisory Committee on Drugs and Alcohol* 2014.

65. Scelza G, Amato A, Pagano AM, et al. Effect of hepatitis C antiviral therapy on oral lichen planus and hyposalivation in inmates. *Annals of Gastroenterology* 2022.

66. Stasi C, Silvestri C, Fanti E, Di Fiandra T, Voller F. Prevalence and features of chronic viral hepatitis and HIV coinfection in Italian prisons. *Eur J Intern Med* 2016.

67. Marco LD, Tullio P, Scalici F, et al. Screening and linkage to care of prisoners with HCV infection: the resist-HCV project. *Journal of Hepatology* 2020.

68. Babudieri S, Longo B, Sarmati L, et al. Correlates of HIV, HBV, and HCV infections in a prison inmate population: results from a multicentre study in Italy. *Journal of Medical Virology* 2005.

69. Sagnelli E, Starnini G, Sagnelli C, et al. Blood born viral infections, sexually transmitted diseases and latent tuberculosis in italian prisons: a preliminary report of a large multicenter study. *European Review for Medical and Pharmacological Sciences* 2012.

70. Brandolini M, Novati, S., De Silvestri A, Tinelli C, Patruno SFA, Ranieri R, Seminari E. Prevalence and epidemiological correlates and treatment outcome of HCV infection in an Italian prison setting. *BMC Public Health* 2013.

71. Giuliani R, Casigliani V, Fornili M, et al. HCV micro-elimination in two prisons in Milan, Italy: A model of care. *Journal of Viral Hepatitis* 2020.

72. Masarone M, Caruso R, Aglitti A, et al. Hepatitis C virus infection in jail: Difficult-to-reach, not to-treat. Results of a point-of-care screening and treatment program. *Dig Liver Dis* 2020.

73. Ranieri R, Foschi A, Casana M, et al. Treating HCV-positive Italian inmates with direct-acting antivirals: the clinical experience in three major correctional houses of Milan. *Journal of the International AIDS Society* 2016.

74. Fiore V, De Matteis G, Ranieri R, et al. HCV testing and treatment initiation in an Italian prison setting: A step-by-step model to micro-eliminate hepatitis C. *Int J Drug Policy* 2021.

75. Izzo C, Masarone M, Torre P, et al. Solving the Gap Between HCV Detection and Treatment in Prison HCV-RNA Testing and Treatment in a Cohort of Newly Arrived Convicts in Southern Italy. *Reviews on Recent Clinical Trials* 2022.

76. Caruso R, Aglitti A, Di Zenzo C, et al. Hepatitis C virus prevalence in a cohort of jailbirds in a city of Southern Italy. *Digestive and Liver Disease* 2019.

77. Voller F, Silvestri C, Orsini C, Aversa L, Da Frè M, Cipriani F. [The health conditions of prison inmates in Tuscany]. *Epidemiol Prev* 2011.

78. Montella M, Crispo A, Grimaldi M, et al. Prevalence of hepatitis C virus infection in different population groups in southern Italy. *Infection* 2005.

79. Ciccarese G, Drago F, Oddenino G, Crosetto S, Rebora A, Parodi A. Sexually transmitted infections in male prison inmates. Prevalence, level of knowledge and risky behaviours. *Infez Med* 2020.

80. Muscat K, Cremona C, Fenech TM, Abela M, Padovese V. Sexually transmitted infections epidemiology and risk assessment at the main correctional facility in Malta (2017-2019). *Journal of the European Academy of Dermatology and Venereology* 2022.

81. Jovanovska T, Kocic B, Stojcevska VP. Prevalence, attitudes and knowledge about HIV HBV and HCV infections among inmates in prisons Prilep and Bitola--a pilot study. *Coll Antropol* 2014.

82. Danis K, Doherty L, McCartney M, McCarrol J, Kennedy H. Hepatitis and HIV in Northern Ireland prisons: a cross-sectional study. *Euro Surveill* 2007; **12**(1).

83. Hannula R, Soderholm J, Svendsen T, et al. Hepatitis C outreach project and cross-sectional epidemiology in high-risk populations in Trondheim, Norway. *Therapeutic Advances in Infectious Disease* 2021.

84. Passadouro R. [Prevalence infections and risk factors due to HIV, Hepatitis B and C in a prison establishment in Leiria]. *Acta Medica Portuguesa* 2004.

85. Garcia A, Exposto F, Prieto E, Lopes M, Duarte A, da Silva RC. Association of Trichomonas vaginalis with sociodemographic factors and other STDs among females inmates in Lisbon. *International Journal of STD & AIDS* 2004.

86. Liberal R, Gaspar R, Andrade P, et al. Doctors in jails: A proof of concept for tackling hepatitis C in prisons. *Hepatology* 2017.

87. Marques NMD, Margalho R, Melo MJ, da Cunha JGS, Melico-Silvestre AA. Seroepidemiological survey of transmissible infectious diseases in a Portuguese prison establishment. *Brazilian Journal of Infectious Diseases* 2011.

88. Carvalhana S, Pinto R, Leitao J, et al. HCV and HBV prevalence in the population: Large disparity between hepatitis c in the general population, comparing with high risk groups. *United European Gastroenterology Journal* 2014.

89. Barros H, Ramos E, Lucas R. A survey of HIV and HCV among female prison inmates in Portugal. *Cent Eur J Public Health* 2008.

90. Gaspar R, Liberal R, Tavares J, Morgado R, Macedo G. HIPPOCRATES((R)) project: A proof of concept of a collaborative program for hepatitis C virus micro-elimination in a prison setting. *WORLD JOURNAL OF HEPATOLOGY* 2020.

91. Champion JK, Taylor A, Hutchinson S, et al. Incidence of hepatitis C virus infection and associated risk factors among Scottish prison inmates: a cohort study. *Am J Epidemiol* 2004.

92. Taylor A, Munro A, Allen E, et al. Low incidence of hepatitis C virus among prisoners in Scotland. [References]. *Addiction* 2013.

93. Morrison DS, Gilchrist G. Prison admission health screening as a measure of health needs. *Health Bull (Edinb)* 2001.

94. Gonzalez C, Canals J, Ortiz M, et al. Prevalence and determinants of high-risk human papillomavirus (HPV) infection and cervical cytological abnormalities in imprisoned women. *Epidemiology and Infection* 2008.

95. Cabezas J, Castrejon OM, Acin E, et al. Hepatitis C infection in the Spanish prison system. Elimination is a dream at our fingertips. *Journal of Hepatology* 2020.

96. Lerena SL, Cobo C, Alvarez S, et al. A program of testing and treat intended to eliminate hepatitis c in a prison: The JAILFREE-C study. *Hepatology* 2016.

97. García-Guerrero J MMA, Sáiz de la Hoya Zamácola P, Vera-Remartínez EJ. Multi-centre study of the prevalence of latent tuberculosis infection amongst inmates in Spanish prisons. 2010.

98. Saiz De La Hoya P, Bedia M, Murcia J, Cebria J, Sanchez-Paya J, Portilla J. Predictive markers of HIV and HCV infection and co-infection among inmates in a Spanish prison. [Spanish]. *Enfermedades Infecciosas y Microbiologia Clinica* 2005.

99. Olivan G. The health profile of Spanish incarcerated delinquent youths. [References]. *Journal of Adolescent Health* 2001.

100. Vicente-Alcalde N, Tuells J, Egoavil CM, Ruescas-Escolano E, Altavilla C, Caballero P. Immunization Coverage of Inmates in Spanish Prisons. *International Journal of Environmental Research and Public Health* 2020.

101. Ferrer-Castro V, Crespo-Leiro MR, García-Marcos LS, et al. [Evaluation of needle exchange program at Pereiro de Aguiar prison (Ourense, Spain): ten years of experience]. *Revista Espanola de Sanidad Penitenciaria* 2012.

102. Gil-Sierra M, Tellez-Perez F, Rios-Sanchez E, et al. 4CPS-085 Stewardships of hepatitis c virus patients in prisons. British Medical Journal Publishing Group; 2019.

103. de la Hoya PS, Marco A, Garcia-Guerrero J, Rivera A, Prevalhep Study Group. Hepatitis C and B prevalence in Spanish prisons. *European Journal of Clinical Microbiology & Infectious Diseases* 2011.

104. Murcia J, Portilla J, Bedia M, et al. Chronic hepatitis C virus infection and associated liver disease among the inmates of a Spanish prison. *Enfermedades Infecciosas y Microbiologia Clinica* 2009.

105. Cuadrado A, Llerena S, Cobo C, et al. Microenvironment Eradication of Hepatitis C: A Novel Treatment Paradigm. *American Journal of Gastroenterology* 2018.

106. Marco A, Gallego C, Perez-Caceres V, et al. Public Health response to an outbreak of SARS-CoV2 infection in a Barcelona prison. *Epidemiology and Infection* 2021.

107. Serroukh SC-Y. Consumo de sustancias, tratamiento con psicofármacos y patología infecciosa en personas redusas del centro penitenciario Puig de las Basses. *Metas de Enfermería* 2022.

108. Gahrton C, Westman G, Lindahl K, et al. Prevalence of Viremic hepatitis C, hepatitis B, and HIV infection, and vaccination status among prisoners in Stockholm County. *BMC Infectious Diseases* 2019.

109. Baggio S, Pala KC, Rieder JP, Tran NT, Wolff H, Getaz L. Infectious diseases in post-trial detention and comparisons with pre-trial detention: A study in Geneva, Switzerland. *Journal of Infection and Public Health* 2020.

110. Pala KC, Baggio S, Tran NT, Girardin F, Wolff H, Getaz L. Blood-borne and sexually transmitted infections: a cross-sectional study in a Swiss prison. *BMC Infectious Diseases* 2018.

111. Moschetti K, Stadelmann P, Wangmo T, et al. Disease profiles of detainees in the Canton of Vaud in Switzerland: gender and age differences in substance abuse, mental health and chronic health conditions. *BMC Public Health* 2015.

112. Arends RM, Nelwan EJ, Soediro R, et al. Associations between impulsivity, risk behavior and HIV, HBV, HCV and syphilis seroprevalence among female prisoners in Indonesia: A cross-sectional study. *PLoS One* 2019.

113. Hetty W, Alima S, Heri A, et al. Eliminating HCV within prisons in Jakarta. *Hepatology International* 2020.

114. Rey I, Saragih R, Effendi-Ys R, Sembiring J, Siregar G, Zain L. Profile of hepatitis B and C virus infection in prisoners in Lubuk Pakam correctional facilities. IOP Conference Series: Earth and Environmental Science; 2018: IOP Publishing; 2018. p. 012033.

115. Lu MY, Chen CT, Shih YL, et al. Changing epidemiology and viral interplay of hepatitis B, C and D among injecting drug user-dominant prisoners in Taiwan. *Sci Rep* 2021.

116. Yang TH, Fang YJ, Hsu SJ, et al. Microelimination of chronic hepatitis C by universal screening plus direct-acting antivirals for incarcerated persons in Taiwan. *Open Forum Infectious Diseases* 2020.

117. Harnpariphan W, Han, W. M., Supanun R, Ubolyam S, et al. High Proportion of Blood-Borne and Sexually Transmitted Infections Among People Deprived of Liberty in a Central Male Prison in Thailand: A Cross-Sectional Study 2018-2019. *AIDS Research and Human Retroviruses* 2022.

118. Johns Hopkins University Bloomberg School of Public Health. Integrated Biological & Behavioral Surveillance (IBBS) in Selected Cities of Afghanistan Findings of 2012 IBBS survey and comparison to 2009 IBBS

survey. *National AIDS Control Program (NACP) Ministry of Public Health, Afghanistan* 2012.

119. Johns Hopkins University Bloomberg School of Public Health. Integrated Behavioral & Biological Surveillance (IBBS) in Afghanistan: Year 1 Report. *Johns Hopkins University Bloomberg School of Public Health* 2011.

120. Tyagi SK, Sovani V, Dias NP, Tyagi D, Saxena S. Prevalence and risk factors of HCV infection in a prison setting in Uttar Pradesh, India. *Indian Journal of Public Health Research and Development* 2018.

121. Kaur K, Grover GS, Boora PK, et al. Feasibility and effectiveness of hepatitis C micro-elimination among 19 prisons of northern India. *Journal of Hepatology* 2022.

122. Singh V, Kaur A, Kumari S, et al. Seroprevalence of HCV and Transient Elastography in a Correctional Setting. HEPATOLOGY; 2017: WILEY 111 RIVER ST, HOBOKEN 07030-5774, NJ USA; 2017. p. 558A-A.

123. Ramamoorthy M, Venketeswaran A, Seenivasan P, et al. Risk factors and prevalence, hepatitis B virus and hepatitis C virus among prison inmates, Chennai, India, 2015. *International Journal of Infectious Diseases* 2016; **53**: 90.

124. Sharafi H, Poustchi H, Azimian F, et al. Performance of a rapid diagnostic test for screening of hepatitis C in a real-life prison setting. *J Clin Virol* 2019.

125. Moradi G, Jafari S, Zarei B, et al. Prevalence and Risk Factors for Hepatitis B and Hepatitis C Exposure in Iranian Prisoners: A National Study in 2016. *Hepatitis Monthly* 2019.

126. SeyedAlinaghi S, Farhoudi B, Shahmohamadi E, et al. Prevalence of and risk factors for HCV among incarcerated people at Great Tehran Prison: a cross-sectional study. *International journal of prisoner health* 2023.

127. Khajedaluee M, Babaei A, Vakili R, et al. Sero-prevalence of bloodborne tumor viruses (HCV, HBV, HTLV-I and KSHV infections) and related risk factors among prisoners in Razavi Khorasan province, Iran, in 2008. *Hepatitis Monthly* 2016.

128. Ziaee M, Sharifzadeh G, Namaee MH, Fereidouni M. Prevalence of HIV and Hepatitis B, C, D Infections and Their Associated Risk Factors among Prisoners in Southern Khorasan Province, Iran. *Iranian Journal of Public Health* 2014.

129. Hariri S, Alavi M, Roshandel G, et al. An intervention to increase hepatitis C virus diagnosis and treatment uptake among people in custody in Iran. *Int J Drug Policy* 2021.

130. Hariri S, Sharafi H, Sheikh M, et al. Continuum of hepatitis C care cascade in prison and following release in the direct-acting antivirals era. *Harm Reduct J* 2020.

131. Khademi N, Shakiba E, Khodadost M, Khoramdad M. Seroprevalence and related risk behaviors of hepatitis C, hepatitis B and HIV infections among Male prisoners in Kermanshah, Iran. *Archives of Iranian Medicine* 2019.

132. Moradi G, Gouya MM, Zavareh FA, et al. Prevalence and risk factors for HBV and HCV in prisoners in Iran: a national bio-behavioural surveillance survey in 2015. *Tropical Medicine & International Health* 2018.

133. Nokhodian Z, Yazdani MR, Yaran M, et al. Prevalence and risk factors of HIV, syphilis, hepatitis B and C among female prisoners in Isfahan, Iran. *Hepatitis Monthly* 2012.

134. Sarkari B, Eilami O, Khosravani A, Sharifi A, Tabatabaee M, Fararouei M. High prevalence of hepatitis C infection among high risk groups in Kohgiloyeh and Boyerahmad Province, Southwest Iran. *Archives of Iranian Medicine* 2012.

135. Moradi G, Alavian SM, Gholami F, et al. Prevalence of hepatitis B and hepatitis C infections among incarcerated individuals in Iran: A cross-sectional national bio-behavioral study in 2019. *Pathogens* 2021.

136. Wali A, Khan D, Safdar N, et al. Prevalence of tuberculosis, HIV/AIDS, and hepatitis; in a prison of Balochistan: a cross-sectional survey. *BMC Public Health* 2019.

137. Kazi AM, Shah SA, Jenkins CA, Shepherd BE, Vermund SH. Risk factors and prevalence of tuberculosis, human immunodeficiency virus, syphilis, hepatitis B virus, and hepatitis C virus among prisoners in Pakistan. *International Journal pf Infectious Diseases* 2010.

138. Pervaiz A, Ghafoor T, Asghar RJ. Screening of prisoners for Human Immunodeficiency Virus (HIV), Hepatitis C (HCV) and B (HBV) in Punjab Province, Pakistan, 2009. *International Journal of Infectious Diseases* 2012.

139. Memon AR, Shafique K, Memon A, Draz AU, Rauf MUA, Afsar S. Hepatitis B and C prevalence among the high risk groups of Pakistani population. A cross sectional study. *Archives of Public Health* 2012.

140. Gorar ZA, Zulfikar I. Seropositivity of hepatitis C in prison inmates of Pakistan--a cross sectional study in prisons of Sindh. *J Pak Med Assoc* 2010.

141. Butt A, Jafri W, Janjua N, Pasha O. Seroprevalence and risk factors for hepatitis C infection among male prisoners in Karachi, Pakistan. *American Journal of Gastroenterology* 2010.

142. Khan MA, Ayub A, Ayub H, Shafique M, Rahman JA. A comparative study of Hepatitis B and C prevalence using ICT and elisa method in jail inmates. *Pakistan Journal of Medical and Health Sciences* 2017.

143. Fayyaz M, Qazi M, Ishaq M, Chaudhry G, Bukhari MJB. Frequency of hepatitis B and C seropositivity in prisoners. 2006; **22**: 55-8.

144. Niriella MA, Hapangama A, Luke H, Pathmeswaran A, Kuruppuarachchi K, de Silva HJ. Prevalence of hepatitis B and hepatitis C infections and their relationship to injectable drug use in a cohort of Sri Lankan prison inmates. *Ceylon Medical Journal* 2015.

145. Azbel L, Polonsky M, Wegman M, et al. Intersecting epidemics of HIV, HCV, and syphilis among soon-to-be released prisoners in Kyrgyzstan: Implications for prevention and treatment. *International Journal of Drug Policy* 2016.

146. Mendizabal M, Testa P, Rojas M, et al. Pilot study using the ECHO model to enhance linkage to care for patients with hepatitis C in the custodial setting. *Journal of Viral Hepatitis* 2020.

147. Adaszko D, Sotelo JA, Orlando M, Angelerei P. Estudio de prevalencia de VIH, sífilis, hepatitis virales y tuberculosis en personas en contextos de encierro en unidades del Servicio Penitenciario Federal. *Buenos Aires, Ministerio de Salud* 2017.

148. Leal M, Kerr L, Mota RMS, Neto RDP, Seal D, Kendall C. Health of female prisoners in Brazil. *Ciencia & saude coletiva* 2022.

149. Coelho HC, de Oliveira SAN, Miguel JC, et al. Predictive markers for hepatitis C virus infection among Brazilian inmates. *Revista da Sociedade Brasileira de Medicina Tropical* 2009.

150. Falquetto TC, Endringer DC, Andrade TU, Lenz D. Hepatitis c in prisoners and non-prisoners in Colatina, Espirito santo, Brazil. *Brazilian Journal of Pharmaceutical Sciences* 2013.

151. Machado F, Becker D, de Oliveira CF, Possuelo LG, Renner JDP. Seroprevalence of HIV, hepatitis B and C and syphilis infection in prisoners of the central region of Rio Grande do Sul, Brazil. *O Mundo da Saúde* 2019.

152. Pompilio MA, Pontes ERJC, Castro ARCM, et al. Prevalence and epidemiology of chronic hepatitis c among prisoners of Mato Grosso do Sul State, Brazil. *Journal of Venomous Animals and Toxins Including Tropical Diseases* 2011.

153. Guimarães T, Granato CF, Varella D, Ferraz ML, Castelo A, Kallás EG. High prevalence of hepatitis C infection in a Brazilian prison: identification of risk factors for infection. *The Brazilian Journal of Infectious Diseases* 2001.

154. Miranda AE, Vargas PM, St Louis ME, Viana MC. Sexually transmitted diseases among female prisoners in Brazil - Prevalence and risk factors. *Sexually Transmitted Diseases* 2000.

155. Ronchi BR, Rios GM, Knoll RK, Cardoso C. Prevalence of HIV, sifilis, hepatitis B and hepatitis c in the inmates of the penitentiary complex of vale do Itajai-SC. *Sexually Transmitted Infections* 2017.

156. Felisberto M, Saretto AA, Wopereis S, Machado MJ, Spada C. Prevalence of HCV infection in a prison population of the greater Florianopolis area. *Revista da Sociedade Brasileira de Medicina Tropical* 2019.

157. Santos Barros LA, Carolina Pessoni G, Araujo Teles S, et al. Epidemiology of the viral hepatitis B and C in female prisoners of metropolitan regional prison complex in the State of Goias, Central Brazil. *Revista da Sociedade Brasileira de Medicina Tropical* 2013.

158. Defante Ferreto LE, Guedes S, Braz Pauli F, et al. Seroprevalence and associated factors of HIV and Hepatitis C in Brazilian high-security prisons: A state-wide epidemiological study. *PLoS One* 2021.

159. Gonçalves KJGUCdG. História de vida e situação de saúde no ambiente prisional de Goiás: estudo da prevalência de hepatite C em detentos [dissertação]. 2005.

160. Catalan-Soares BC, Almeida RT, Carneiro-Proietti AB. Prevalence of HIV-1/2, HTLV-I/II, hepatitis B virus (HBV), hepatitis C virus (HCV), Treponema pallidum and Trypanosoma cruzi among prison inmates at Manhuacu, Minas Gerais State, Brazil. *Revista da Sociedade Brasileira de Medicina Tropical* 2000.

161. do Nascimento CT, Pena DZ, Giuffrida R, et al. Prevalence and epidemiological characteristics of inmates diagnosed with infectious diseases living in a region with a high number of prisons in Sao Paulo state, Brazil. *BMJ Open* 2020.

162. El Maerrawi I, Carvalho HB. Prevalence and risk factors associated with HIV infection, hepatitis and syphilis in a state prison of São Paulo. *Int J STD AIDS* 2015.

163. Massad E, Rozman M, Azevedo R, et al. Seroprevalence of HIV, HCV and syphilis in Brazilian prisoners: preponderance of parenteral transmission. *European journal of epidemiology* 1999; **15**: 439-45.

164. Leite AGD, Damasceno LM, Conceicao SC, Motta PFC. Rapid tests for HIV, syphilis, and chronic hepatitis in a prison population in a prison complex in Salvador (BA), Brazil. *Ciencia & saude coletiva* 2022.

165. Barros LAS, Pessoni GC, Teles SA, et al. Epidemiology of the viral hepatitis B and C in female prisoners of Metropolitan Regional Prison Complex in the State of Goias, Central Brazil. *Revista da Sociedade Brasileira de Medicina Tropical* 2013.

166. Soares Epifania P, Santos Passos Costa J, Costa Barros KC, Santos de Freitas K, Sampaio Maciel G, da Silva Santos Passos S. Doenças infectocontagiosas em indivíduos privados de liberdade. *Enfermagem Brasil* 2022.

167. Puga MAM, Bandeira LM, Pompilio MA, et al. Prevalence and Incidence of HCV Infection among Prisoners in Central Brazil. *PLoS One* 2017.

168. Santos BFO, de Santana NO, Franca AVC. Prevalence, genotypes and factors associated with HCV infection among prisoners in Northeastern Brazil. *World Journal of Gastroenterology* 2011.

169. Strazza L, Azevedo RS, Carvalho HB, Massad E. The vulnerability of Brazilian female prisoners to HIV infection. *Braz J Med Biol Res* 2004.

170. da Rosa F, Carneiro M, Duro LN, et al. Prevalence of anti-HCV in an inmate population. *Revista da Associacao Medica Brasileira* 2012.

171. Belaunzaran-Zamudio PF, Mosqueda-Gomez JL, Macias-Hernandez A, Rodríguez-Ramírez S, Sierra-Madero J, Beyrer C. Burden of HIV, Syphilis, and Hepatitis B and C Among Inmates in a Prison State System in Mexico. *AIDS Res Hum Retroviruses* 2017.

172. Silverman-Retana O, Servan-Mori E, McCoy SI, Larney S, Bautista-Arredondo S. Hepatitis C antibody prevalence among Mexico City prisoners injecting legal and illegal substances. *Drug and Alcohol Dependence* 2017.

173. Bautista-Arredondo S. Prevalence of transmissible infections and socio-demographic and behavioral risk factors amongst prisoners in Mexico City: A cross-sectional study of 17,296 inmates. *Journal of the International AIDS Society* 2012.

174. Bautista-Arredondo S, González A, Servan-Mori E, et al. A Cross-Sectional Study of Prisoners in Mexico City Comparing Prevalence of Transmissible Infections and Chronic Diseases with That in the General Population. *PLoS One* 2015.

175. Alvarado-Esquivel C, Sablon E, Martínez-García S, Estrada-Martínez S. Hepatitis virus and HIV infections in inmates of a state correctional facility in Mexico. *Epidemiology and Infection* 2005.

176. Gonzalez CAM, Ortiz BES, Aguilar MB, Gonzalez JDM. Risk factors and the seroprevalence of viral markers of hepatitis B (HVB) and hepatitis C (HCV) in high-risk groups in Chiapas. *Medwave* 2011.

177. Alcivar JC, Zambrano MM, Madronero MG, et al. Sexually transmitted infections in inmates in Merida Venezuela. *INVESTIGACION CLINICA* 2020.

178. Monsalve-Castillo F, Chacín-Bonilla L, Atencio RJ, et al. Low prevalence of hepatitis C virus infection in a prisoner population from Maracaibo, Venezuela. *Biomedica* 2009.

179. Courtemanche Y, Poulin C, Serhir B, Alary M. HIV and hepatitis C virus infections in Quebec's provincial detention centres: comparing prevalence and related risky behaviours between 2003 and 2014-2015. *Can J Public Health* 2018.

180. Kronfli N, Dussault C, Klein MB, Lebouche B, Sebastiani G, Cox J. The hepatitis C virus cascade of care in a Quebec provincial prison: a retrospective cohort study. *CMAJ Open* 2019.

181. Poulin C, Alary M, Lambert G, et al. Prevalence of HIV and hepatitis C virus infections among inmates of Quebec provincial prisons. [References]. *Canadian Medical Association Journal* 2007.

182. Besney JD, Angel C, Pyne D, Martell R, Keenan L, Ahmed R. Addressing Women’s Unmet Health Care Needs in a Canadian Remand Center. *Journal of Correctional Health Care* 2018.

183. Calzavara L, Ramuscak N, Burchell AN, et al. Prevalence of HIV and hepatitis C virus infections among inmates of Ontario remand facilities. *Cmaj* 2007.

184. Ford PM, Pearson M, Sankar-Mistry P, Stevenson T, Bell D, Austin J. HIV, hepatitis C and risk behaviour in a Canadian medium-security federal penitentiary. Queen's University HIV Prison Study Group. *Qjm* 2000.

185. Akiyama MJ, Kaba F, Rosner Z, et al. Correlates of hepatitis C virus infection in the targeted testing program of the New York city jail system: Epidemiologic patterns and priorities for action. *Public Health Reports* 2017.

186. Abe CM, Aguwa M, Zhao M, Sullivan J, Porsa E, Nijhawan AE. Hepatitis C Virus Infection in the Dallas County Jail: Implications for Screening, Prevention, and Linkage to Care. *Public Health Rep* 2019.

187. Solomon L, Flynn C, Muck K, Vertefeuille J. Prevalence of HIV, syphilis, hepatitis B, and hepatitis C among entrants to Maryland correctional facilities. *Journal of Urban Health* 2004.

188. Alvarez KJ, Befus M, Herzig CTA, Larson E. Prevalence and correlates of hepatitis C virus infection among inmates at two New York State correctional facilities. *Journal of Infection and Public Health* 2014.

189. Hoff E, Warden A, Taylor R, Nijhawan AE. Hepatitis C Epidemiology in a Large Urban Jail: A Changing Demographic. *Public Health Reports* 2023.

190. Phung J, Pham HM, Shin RB, Tsolova V, Patel MC, Chan J. Prevalence of hepatitis c virus in an incarcerated population. *Hepatology* 2021.

191. Assoumou SA, Wang J, Tasillo A, et al. Hepatitis C Testing and Patient Characteristics in Washington State's Prisons Between 2012 and 2016. *Am J Prev Med* 2019.

192. Bai JR, Mukherjee DV, Befus M, Apa Z, Lowy FD, Larson EL. Concordance between medical records and interview data in correctional facilities. *BMC Med Res Methodol* 2014.

193. Sieck CJ, Dembe AE. Results of a pilot study of pre-release STD testing and inmates' risk behaviors in an Ohio prison. *J Urban Health* 2011.

194. Nijhawan AE, Sullivan J, Aguwa M, Porsa E. Demographic trends in HCV diagnosis and linkage to HCV care among jail detainees. *Topics in Antiviral Medicine* 2019.

195. Cocoros N, Nettle E, Church D, et al. Screening for hepatitis C as a prevention enhancement (SHAPE) for HIV: An integration pilot initiative in a massachusetts county correctional facility. *Public Health Reports* 2014.

196. Larney S, Mahowald MK, Scharff N, Flanigan TP, Beckwith CG, Zaller ND. Epidemiology of hepatitis C virus in Pennsylvania state prisons, 2004-2012: limitations of 1945-1965 birth cohort screening in correctional settings. *Am J Public Health* 2014.

197. Scott J, Sampson LA, Clymore JM, Moore PR, Leone PA. Integrated HIV, syphilis, and other STI testing in North Carolina county jails. *Sexually Transmitted Infections* 2011.

198. Ruiz JD, Molitor F, Plagenhoef JA. Trends in hepatitis C and HIV infection among inmates entering prisons in California, 1994 versus 1999. *Aids* 2002.

199. Lincoln T, Tuthill RW, DePietro SL. Viral hepatitis, risk behaviors, aminotransferase levels, and screening options at a county correctional center. *Journal of Correctional Health Care* 2006.

200. Weant TE, Turner AN, Murphy-Weiss M, Murray DM, Wang SH. Can social history variables predict prison inmates' risk for latent tuberculosis infection? *Tuberc Res Treat* 2012; **2012**: 132406.

201. Magaldi LN, Trooskin S, Anderson J, et al. Routine Opt out Hepatitis C Testing Upon Intake in the Philadelphia Jail System. *Hepatology* 2022.

202. Spaulding AC, Chen J, Mackey CA, et al. Assessment and Comparison of Hepatitis C Viremia in the Prison Systems of New Mexico and Georgia. *JAMA Netw Open* 2019.

203. Baillargeon J, Wu H, Kelley MJ, Grady J, Linthicum L, Dunn K. Hepatitis C seroprevalence among newly incarcerated inmates in the Texas correctional system. *Public Health* 2003.

204. Macalino GE, Vlahov D, Sanford-Colby S, et al. Prevalence and Incidence of HIV, Hepatitis B Virus, and Hepatitis C Virus Infections Among Males in Rhode Island Prisons. [References]. *American journal of public health* 2004.

205. Chan J, Kaba F, Schwartz J, et al. The hepatitis C virus care cascade in the New York City jail system during the direct acting antiviral treatment era, 2014-2017. *eClinicalMedicine* 2020.

206. Leukefeld C, Harp KLH, Webster M, Staton-Tindall M, Oser CB, Havens JR. Examining HCV and other risks among rural women offenders. *Drug and Alcohol Dependence* 2015.

207. Kuncio DE, Newbern EC, Fernandez-Viña MH, Herdman B, Johnson CC, Viner KM. Comparison of risk-based hepatitis C screening and the true seroprevalence in an urban prison system. *J Urban Health* 2015.

208. Baillargen J, Snyder N, Soloway RD, et al. Hepatocellular Carcinoma Prevalence and Mortality in a Male State Prison Population. *Public Health Reports* 2009.

209. Akiyama MJ, Kaba F, Rosner Z, Alper H, Holzman RS, MacDonald R. Hepatitis C Screening of the Birth Cohort (Born 1945-1965) and Younger Inmates of New York City Jails. *Am J Public Health* 2016.

210. Abe C, Porsa E, Nijhawan AE. Hepatitis C care cascade in jail: Implications for hard-to-reach populations. *Topics in Antiviral Medicine* 2018.

211. Deb LC, Hove H, Miller TK, et al. Epidemiology of Hepatitis C virus infection among incarcerated populations in North Dakota. *PLoS One* 2022.

212. Macalino GE, Dhawan D, Rich JD. A missed opportunity: hepatitis C screening of prisoners. *Am J Public Health* 2005.

213. Fox RK, Currie SL, Evans J, et al. Hepatitis C virus infection among prisoners in the California State correctional system. *Clinical Infectious Diseases* 2005.

214. de la Flor C, Porsa E, Nijhawan AE. Opt-out HIV and Hepatitis C Testing at the Dallas County Jail: Uptake, Prevalence, and Demographic Characteristics of Testers. *Public Health Rep* 2017.

215. Wenger PJ, Rottnek F, Parker T, Crippin JS. Assessment of hepatitis C risk factors and infection prevalence in a jail population. *American journal of public health* 2014; **104**(9): 1722-7.

216. Indig D, Topp L, Ross B, et al. 2009 NSW inmate health survey: key findings report. *Justice Health, Sydney* 2010: 16.

217. Bah R, Sheehan Y, Li X, et al. Prevalence of blood-borne virus infections and uptake of hepatitis C testing and treatment in Australian prisons: the AusHep study. *The Lancet Regional Health–Western Pacific* 2024; **53**.

218. Butler TS, M. National Prison Entrants’ Bloodborne Virus and Risk Behaviour Survey Report: Kirby Institute 2017.

219. Indig D, Topp L, Ross B, et al. 2009 NSW Inmate Health Survey: Key Findings Report. Sydney: Justice Health, 2010.

220. Young JT, Van Dooren K, Borschmann R, Kinner S. ACT detainee health and wellbeing survey 2016: Summary results: ACT government; 2017.

221. Hellard ME, Hocking JS, Crofts N. The prevalence and the risk behaviours associated with the transmission of hepatitis C virus in Australian correctional facilities. *Epidemiol Infect* 2004.

222. Miller ER, Bi P, Ryan P. The prevalence of HCV antibody in South Australian prisoners. *J Infect* 2006.

223. Hajarizadeh B, Grebely J, Byrne M, et al. Evaluation of hepatitis C treatment-as-prevention within Australian prisons (SToP-C): a prospective cohort study. *LANCET GASTROENTEROLOGY & HEPATOLOGY* 2021.

224. Murray N, LePage E, Butler T. Hearing health of New South Wales prison inmates. *Aust N Z J Public Health* 2004.

225. Butler T, Boonwaat L, Hailstone S, et al. The 2004 Australian prison entrants' blood-borne virus and risk behaviour survey. *Aust N Z J Public Health* 2007.

226. Snow KJ, Richards AH, Kinner SA. Use of multiple data sources to estimate hepatitis C seroprevalence among prisoners: A retrospective cohort study. *PLoS One* 2017.

227. Gilles M, Swingler E, Craven C, Larson A. Prison health and public health responses at a regional prison in Western Australia. *Australia and New Zealand Journal of Public Health* 2008.

228. Watkins RE, Mak DB, Connelly C. Testing for sexually transmitted infections and blood borne viruses on admission to Western Australian prisons. *BMC Public Health* 2009.

229. Miller ER, Bi P, Ryan P. Hepatitis C virus infection in South Australian prisoners: seroprevalence, seroconversion, and risk factors. *Int J Infect Dis* 2009.

230. Health J. National Patient Health Survey. 2017.

231. Reekie JM, Levy MH, Richards AH, et al. Trends in prevalence of HIV infection, hepatitis B and hepatitis C among Australian prisoners - 2004, 2007, 2010. *Medical Journal of Australia* 2014.

232. Awofeso N, Harper SE, Levy MH. Prevalence of exposure to hepatitis C virus among prison inmates, 1999 [4]. *Medical Journal of Australia* 2000.

233. Dore G, Hajarizadeh B, Grebely J, et al. Declining HCV incidence following rapid HCV treatment scale-up in a prison network in Australia: Evidence of treatment as prevention from the SToP-C study. *Journal of Hepatology* 2020.

234. Papaluca T, Howell J, McDonald L, Craigie A, Iser D. Chronic hepatitis B within the Victorian Prisons, Australia-high prevalence of cirrhosis and HBV-HCV and HBV-HDV coinfection. *Hepatology International* 2018.

235. Butler T, Spencer J, Cui J, Vickery K, Zou J, Kaldor J. Seroprevalence of markers for hepatitis B, C and G in male and female prisoners‐NSW, 1996. *Australian and New Zealand journal of public health* 1999; **23**(4): 377-84.

236. Hockings BA, Young, M., Falconer, A., and O'Rourke, P.K. Queensland Women Prisoners' Health Survey. Brisbane: Department of Corrective Services, 2002.

237. Stoové M, Kirwan A. External component of the evaluation of drug policies and services and their subsequent effects on prisoners and staff within the Alexander Maconochie Centre. 2011.

238. Ouedraogo O, Garanet F, Sawadogo S, Mesenge C, Schmid JBG. Vulnerability of male prisoners to HIV/AIDS in Ouagadougou, Burkina Faso. *Sante Publique* 2015.

239. Kassa Y, Million Y, Biset S, Moges F. Hepatitis b and hepatitis c viral infections and associated factors among prisoners in northeast ethiopia. *Journal of Blood Medicine* 2021.

240. Kebede W, Abdissa A, Seid Y, Mekonnen Z. Seroprevalence and risk factors of hepatitis B, hepatitis C and HIV infections among prisoners in Jimma Town, Southwest Ethiopia. *Asian Pacific Journal of Tropical Disease* 2017.

241. Adjei AA, Armah HB, Gbagbo F, et al. Correlates of hepatitis C virus infection among incarcerated Ghanaians: a national multicentre study. *J Med Microbiol* 2007.

242. Adjei AA, Armah HB, Gbagbo F, et al. Prevalence of human immunodeficiency virus, hepatitis B virus, hepatitis C virus and syphilis among prison inmates and officers at Nsawan and Accra, Ghana. *Journal of Medical Microbiology* 2006.

243. Adjei AA, Armah HB, Gbagbo F, et al. Prevalence of human immunodeficiency virus, hepatitis B virus, hepatitis C virus and syphilis among prison inmates and officers at Nsawam and Accra, Ghana. *Journal of Medical Microbiology* 2006; **55**(5): 593-7.

244. Jones MJ. INTEGRATED BIO-BEHAVIOURAL SURVEILLANCE SURVEY REPORT OF KEY POPULATIONS IN LIBERIA (IBBSS, 2018). In: Health Mo, editor.; 2019.

245. Chimphambano C, Komolafe I, Muula A. Prevalence of HIV, HepBsAg and Hep C antibodies among inmates in Chichiri prison, Blantyre, Malawi. *Malawi Medical Journal* 2007; **19**(3): 107-10.

246. Adoga MP, Banwat EB, Forbi JC, et al. Human immunonodeficiency virus, hepatitis B virus and hepatitis C virus: sero-prevalence, co-infection and risk factors among prison inmates in Nasarawa State, Nigeria. *The Journal of Infection in Developing Countries* 2009.

247. Okafor IM, Ugwu SO, Okoroiwu HU. Hepatitis C virus infection and its associated factors among prisoners in a Nigerian prison. *BMC Gastroenterol* 2020.

248. Umutesi J, Klett-Tammen C, Nsanzimana S, Krause G, Ott JJ. Cross-sectional study of chronic hepatitis B virus infection in Rwandan high-risk groups: Unexpected findings on prevalence and its determinants. *BMJ Open* 2021.

249. Agency for the Promotion of Population Activities Senegal. ENQU bÊTE NATIO NALE DE SURVEILLANCE COMBINEE DES IST ET DU VIH/SIDA (ENSC 2019) COMPOSANTE COMPORTEMENTALE. In: SOCIALE MDLSEDLA, editor.; 2020.

250. Jaquet A, Wandeler G, Tine J, et al. HIV infection, viral hepatitis and liver fibrosis among prison inmates in West Africa. *BMC Infectious Diseases* 2016.

251. The Aurum Institute NICD. Socio-behavioural and structural factors driving HIV/AIDS, STIs and Hepatitis B & C infections among inmates in Correctional Facilities, Johannesburg, 2020.

252. M. Dahoma EM, A. Othman, A. Seha, A. Abdullah. Predisposing sexual and drug related risk factors among prisoners in Zanzibar. International Aids Society (IAS) 2009. Cape Town; 2009.

253. United Nations Office on Drugs and Crime. A Rapid Situation Assessment of HIV/STI/TB and Drug Abuse among Prisoners in Uganda Prisons Service, 2009.

254. Maggard KR, Hatwiinda S, Harris JB, et al. Screening for tuberculosis and testing for human immunodeficiency virus in Zambian prisons. *Bulletin of the World Health Organization* 2015.

255. Mohamed HI, Saad ZM, Abd-Elreheem EM, et al. Hepatitis C, hepatitis B and HIV infection among Egyptian prisoners: seroprevalence, risk factors and related chronic liver diseases. *J Infect Public Health* 2013.

256. Mahfoud Z, Kassak K, Kreidieh K, Shamra S, Ramia S. Prevalence of antibodies to human immunodeficiency virus (HIV), hepatitis B and hepatitis C and risk factors in prisoners in Lebanon. *The Journal of Infection in Developing Countries* 2010.

257. Ziglam H, Zorgani AA, Balouz A, Abudhe AH, Elahmer O. Prevalence of antibodies to human immunodeficiency virus, hepatitis B, and hepatitis C in prisoners in Libya. *Libyan Journal of Medicine* 2012.

258. Elahmer O, Zorgani A, Abudher A, Ziglam H. Prevalence of human immunodeficiency virus, hepatitis B virus, hepatitis C virus among prison inmates, western Libya. *Clinical Microbiology and Infection* 2012.

259. Kobeissi L. The Integrated Bio-Behavioral Survey (IBBS) in Syria: 2013-2014. In: United Nations Development Program, editor.; 2014.

260. Balci E, Turker K, Senol V, Gunay O. Screening Indicators of Hepatitis A, Hepatitis B, Hepatitis C and HIV infections in Prisoners. *Viral Hepatitis Journal* 2012.

261. ÖZger HS, KaraŞAhİN Ö, Toy MA, Yilmaz Sİ, Hizel K. Hepatitis C Prevalence and Responses to Pegylated Interferon + Ribavirin Treatment Among Prisoners. *Viral Hepatitis Journal / Viral Hepatit Dergisi* 2017.

262. Sahin AM, Sahin AR, Gunduz A, Aktemur A, Uzun N. Prevalence of Hepatitis B virus and Hepatitis C virus among prison inmates in Istanbul, Turkey. *Annals of Clinical and Analytical Medicine* 2022.

263. Keten D, Ova ME, Keten HS, et al. The prevalence of hepatitis B and C among prisoners in Kahramanmaras, Turkey. *Jundishapur Journal of Microbiology* 2016.
