# Supplementary material for: The global epidemiology of injecting drug use, HIV, viral hepatitis and tuberculosis among people who are incarcerated: a multistage systematic review
Source: Int J Drug Policy. Author manuscript; Available in PMC 2026 Apr 8. (PMC13058553; doi:10.1016/j.drugpo.2025.105062)
Supplement: 10 [file NIHMS2157186-supplement-10.docx]

**Supplementary material for**

**The global epidemiology of injecting drug use, HIV, viral hepatitis and tuberculosis among people who are incarcerated: a multistage systematic review**

**Louisa Degenhardt^1^, Matthew Hickman^1,2^, Frederick L. Altice^3^, Jason Grebely^4^, Sophia Taylor^1^, Michelle Lynch^1^, Aleksa Kamenjas^1^, Jack Marsden^1^, Lucy T. Tran^1^, Paige Webb^1^, Olivia Price^1^, Christel MacDonald^1^, Filipa Alves da Costa^5^, Justin Berk^6^, Anja Busse^7^, Evan Cunningham^4^, Colleen Daniels^8^, Behzad Hajarizadeh^4^, Linda Montanari^9^, Luis Royeula^9^, Keith Sabin^10^, Jack Stone^2^, Annette Verster^11^, Peter Vickerman^2^, Michael Farrell^1^, and Thomas Santo Jr.^1^**

**Table of contents**

[Appendix 1: GATHER and PRISMA checklist 4](#_Toc212110394)

[1.1 GATHER checklist 4](#_Toc212110395)

[1.2 PRISMA checklist 5](#_Toc212110396)

[Appendix 2: Peer Reviewed Literature Search 7](#_Toc212110397)

[2.1 Medline/PubMed Search Strategy 7](#_Toc212110398)

[2.2. Embase Search Strategy 8](#_Toc212110399)

[2.3. PsycINFO Search Strategy 10](#_Toc212110400)

[2.4. Web of Science Search Strategy 11](#_Toc212110401)

[2.5. CINAHL Search Strategy 13](#_Toc212110402)

[Appendix 3: Grey Literature Search 15](#_Toc212110403)

[Table 3.1: Websites searched in the grey literature search 16](#_Toc212110404)

[Appendix 4: Additional requests for information 32](#_Toc212110405)

[4.1 Example email 32](#_Toc212110406)

[4.2.1 Facebook advertisement example 33](#_Toc212110407)

[4.2.2 Twitter advertisement example 34](#_Toc212110408)

[Appendix 5: Screening Process and Criteria 35](#_Toc212110409)

[Appendix 6: Classification system used in the assessment of study methodologies 37](#_Toc212110410)

[Appendix 6.1: Classification approach for grading studies 37](#_Toc212110411)

[Appendix 7: Summary of decision rules for data extraction and estimation processes 38](#_Toc212110412)

[Appendix 8: Method of estimating regional and global numbers 41](#_Toc212110413)

[Appendix 9: Country level prevalence and estimated numbers of injecting drug use, HIV, current HCV infection, HBV and tuberculosis among people, females and males who are incarcerated 44](#_Toc212110414)

[*Table 9.1*: Country-level estimates of the number and rate of incarceration, prevalence of lifetime injecting drug use, HIV and HCV among total people incarcerated 44](#_Toc212110415)

[*Table 9.2*: Country-level estimates of the number and rate of incarceration, prevalence of HBV and tuberculosis among total people incarcerated 67](#_Toc212110416)

[*Table 9.3*: Country-level estimates of the number and rate of incarceration, prevalence of lifetime injecting drug use, HIV and HCV among females who are incarcerated 81](#_Toc212110417)

[*Table 9.4*: Country-level estimates of the number and rate of incarceration, prevalence of HBV and tuberculosis among females who are incarcerated 93](#_Toc212110418)

[*Table 9.5:* Country-level estimates of the number and rate of incarceration, prevalence of lifetime injecting drug use, HIV and HCV among males who are incarcerated 102](#_Toc212110419)

[*Table 9.6*: Country-level estimates of the number and rate of incarceration, prevalence of HBV and tuberculosis among men who are incarcerated 116](#_Toc212110420)

[*Table 9.7*: Combined country-level estimates of the number and rate of incarceration, prevalence of lifetime injecting drug use, HIV and HCV among people who are incarcerated (mixed estimates only) 126](#_Toc212110421)

[*Table 9.8*: Combined country-level estimates of the number and rate of incarceration, prevalence of lifetime HBV and Tuberculosis among people who are incarcerated (mixed estimates only) 141](#_Toc212110422)

[Appendix 10: Sensitivity analysis - regional and global estimates of the prevalence and number of people who are incarcerated who are living with HIV, have current HCV or HBV infection, have active tuberculosis and have injected drugs, with only the most recent five years of data included for countries with multiple estimates available 151](#_Toc212110423)

[Appendix 11: Summary of records included in estimates 154](#_Toc212110424)

[Appendix 12. Lists of studies excluded or not extracted 197](#_Toc212110425)

[*Table 12.1:* List of studies excluded at full text screening stage and Dolan et al. references not included in estimates of the current review with reasons for exclusion 197](#_Toc212110426)

[*Table 12.2:* List of studies excluded at full text screening stage from the search update with reasons for exclusion 295](#_Toc212110427)

[*Table 12.3:* List of studies included in screening but not extracted and reasons not extracted 303](#_Toc212110428)

[*Table 12.4:* List of studies included in screening from search update but not extracted and reasons not extracted 356](#_Toc212110429)

[Appendix 13: People who provided data or advice regarding the reviews 364](#_Toc212110430)

[Appendix 14: Details of methods used to assess risk of bias 365](#_Toc212110431)

[Table 14.1. Appraisal rules (Yes/No/Unclear) 365](#_Toc212110432)

# Appendix 1: GATHER and PRISMA checklist

## GATHER checklist

| **#** | **GATHER checklist item** | **Description of compliance** | **Reference** |
| --- | --- | --- | --- |
| **Objectives and funding** | | | |
| 1 | Define the indicators, populations, and time periods for which estimates were made. | Narrative provided in paper and methods appendix describing indicators, definitions, and populations | Manuscript (Methods) and methods appendix |
| 2 | List the funding sources for the work. | Funding sources listed in paper | Manuscript (Acknowledgements) |
| **Data Inputs** | | | |
| *For all data inputs from multiple sources that are synthesized as part of the study:* | | | |
| 3 | Describe how the data were identified and how the data were accessed. | Narrative description of data seeking  methods provided | Manuscript (Methods) and appendix |
| 4 | Specify the inclusion and exclusion criteria. Identify all ad-hoc exclusions. | Narrative about inclusion and exclusion criteria by data type provided | Methods appendix |
| 5 | Provide information on all included data sources and their main characteristics. For each data source used, report reference information or contact name/institution, population represented, data collection method, year(s) of data collection, sex and age range, diagnostic criteria or measurement method, and sample size, as relevant. | Included in appendix and will be accessible online | Appendix and online web page |
| 6 | Identify and describe any categories of input data that have potentially important biases (e.g., based on characteristics listed in item 5). | Discussed in limitations section and in methods appendix | Manuscript and appendix |
| *For data inputs that contribute to the analysis but were not synthesized as part of the study:* | | | |
| 7 | Describe and give sources for any other data inputs. | N/A | N/A |
| *For all data inputs* | | | |

## PRISMA checklist

| **Section/topic** | **#** | **Checklist item** | **Reported on page #** |
| --- | --- | --- | --- |
| **TITLE** | | |  |
| Title | 1 | Identify the report as a systematic review, meta-analysis, or both. | 1 |
| **ABSTRACT** | | |  |
| Structured summary | 2 | Provide a structured summary including, as applicable: background; objectives; data sources; study eligibility criteria, participants, and interventions; study appraisal and synthesis methods; results; limitations; conclusions and implications of key findings; systematic review registration number. | 2 |
| **INTRODUCTION** | | |  |
| Rationale | 3 | Describe the rationale for the review in the context of what is already known. | 3 |
| Objectives | 4 | Provide an explicit statement of questions being addressed with reference to participants, interventions, comparisons, outcomes, and study design (PICOS). | 3 |
| **METHODS** | | |  |
| Protocol and registration | 5 | Indicate if a review protocol exists, where it can be accessed, and, if available, provide registration information including registration number. | 4 |
| Eligibility criteria | 6 | Specify study characteristics (e.g., PICOS, length of follow-up) and report characteristics (e.g., years considered, language, publication status) used as criteria for eligibility, giving rationale. | 4-6, appendix |
| Information sources | 7 | Describe all information sources (e.g., databases with dates of coverage, contact with study authors to identify additional studies) in the search and date last searched. | 4-5, appendix |
| Search | 8 | Present full electronic search strategy for at least one database, including any limits used, such that it could be repeated. | Appendix |
| Study selection | 9 | State the process for selecting studies (i.e., screening, eligibility, included in systematic review, and, if applicable, included in the meta-analysis). | 5-6 |
| Data collection process | 10 | Describe method of data extraction (e.g., piloted forms, independently, in duplicate) and any processes for obtaining and confirming data from investigators. | Appendix |
| Data items | 11 | List and define all variables for which data were sought (e.g., PICOS, funding sources) and any assumptions and simplifications made. | Appendix |
| Risk of bias in individual studies | 12 | Describe methods used for assessing risk of bias of individual studies (including specification of whether this was done at the study or outcome level), and how this information is to be used in any data synthesis. | 8, Appendix |
| Summary measures | 13 | State the principal summary measures (e.g., risk ratio, difference in means). | 6-7, Appendix |
| Synthesis of results | 14 | Describe the methods of handling data and combining results of studies, if done, including measures of consistency (e.g., I^2^) for each meta-analysis. | 6-7, Appendix |
| Risk of bias across studies | 15 | Specify any assessment of risk of bias that may affect the cumulative evidence (e.g., publication bias, selective reporting within studies). | Study characteristics in Appendix |
| Additional analyses | 16 | Describe methods of additional analyses (e.g., sensitivity or subgroup analyses, meta-regression), if done, indicating which were pre-specified. | 7-8 |
| **RESULTS** | | |  |
| Study selection | 17 | Give no. studies screened, assessed for eligibility, and included in the review, with reasons for exclusions at each stage, ideally with a flow diagram. | 9, Appendix |
| Study characteristics | 18 | For each study, present characteristics for which data were extracted (e.g., study size, PICOS, follow-up period) and provide the citations. | Appendix |
| Risk of bias within studies | 19 | Present data on risk of bias of each study and, if available, any outcome level assessment (see item 12). | Appendix |
| Results of individual studies | 20 | For all outcomes considered (benefits or harms), present, for each study: (a) simple summary data for each intervention group (b) effect estimates and confidence intervals, ideally with a forest plot. | Appendix |
| Synthesis of results | 21 | Present results of each meta-analysis done, including confidence intervals and measures of consistency. | 10-12, Appendix |
| Risk of bias across studies | 22 | Present results of any assessment of risk of bias across studies (see Item 15). | 12-13 |
| Additional analysis | 23 | Give results of additional analyses, if done (e.g., sensitivity or subgroup analyses, meta-regression [see Item 16]). | 12, Appendix |
| **DISCUSSION** | | |  |
| Summary of evidence | 24 | Summarize the main findings including the strength of evidence for each main outcome; consider their relevance to key groups | 14 |
| Limitations | 25 | Discuss limitations at study and outcome level (e.g., risk of bias), and at review-level (e.g., incomplete retrieval of identified research, reporting bias). | 15-17 |
| Conclusions | 26 | Provide a general interpretation of the results in the context of other evidence, and implications for future research. | 19 |
| **FUNDING** | | |  |
| Funding | 27 | Describe sources of funding for the systematic review and other support (e.g., supply of data); role of funders for the systematic review. | 8, 20 |

# Appendix 2: Peer Reviewed Literature Search

Five peer reviewed literature databases were searched in this exercise: Medline, EMBASE, PsycINFO, Web of Science, and CINAHL.

Listed below are the different literature and search strategies and terminologies used for each of the different databases. Because different databases use different terminologies and require searching in different ways it was necessary to develop search strategies for each database.

These searches were developed in close consultation with a specialist drug and alcohol archivist and with a generalist university librarian with expertise across all the databases searched.

## 2.1 Medline/PubMed Search Strategy

The search strategy was as follows:

Results were restricted to those articles containing terms from search 1 and terms from one or more results of searches 2-8, combined as 1 AND (2 OR 3 OR 4 OR 5 OR 6 OR 7 OR 8). Results were restricted to articles published 2000-current and limited to population group: humans.

Original searches were conducted in March 2023, the searches were then updated to 2^nd^ June 2025. Medline was used in the original search and the updated search was done in PubMed.

Key-words are noted below in regular type, ‘MESH’ (Medical subject heading) terms in **bold**.

| **Search 1: Incarceration settings** |
| --- |
| Prison* OR Jail* or Detention* OR "Compulsory Drug Treatment" OR "Closed Setting" OR Prisoners* OR Criminal* OR Offender* OR Post-Release* OR Incarerat* OR **prisoners** |

| **Search 2: Injecting drug use** |
| --- |
| "IDU" OR "IDUs" OR "PWID" OR "injecting drug*" OR "intravenous drug*" OR "injecting substance*" OR "intravenous substance*" OR "people who inject*" OR "injection drug*" OR **substance abuse, intravenous** |

| **Search 3. HIV/AIDS** |
| --- |
| "HIV" OR "AIDS" OR "HIV/AIDS" OR “Human Immunodeficiency Virus” OR “Human Immune Deficiency Virus” OR “Acquired Immunodeficiency Syndrome” OR “Acquired Immune Deficiency Syndrome” OR "anti?retroviral" OR "anti?HIV agents" OR “highly active anti?retroviral therapy” OR “combination anti?retroviral therapy” OR "HAART" OR "cART" |

| **Search 4: Hepatitis** |
| --- |
| Hepatitis OR “hepatitis C” OR “hep C” OR HCV OR DAA OR "hepatitis B" or "hep B" or "HBV" OR **hepatitis b** OR **hepatitis c** |

| **Search 5: Needle syringe program** |
| --- |
| ("needle" AND ("exchange" OR "program*")) OR ("syringe" AND ("exchange" OR "program*")) OR **needle-exchange programs** OR **harm reduction** |

| **Search 6: Opioid agonist treatment** |
| --- |
| OST OR OAT OR “opioid substitution treatment” OR “methadone” OR “buprenorphine” OR “opioid replacement” OR “opioid substitution” OR “opiate substitution” OR “opioid agonist” OR “opiate agonist” OR “drug treatment” OR “medication assisted” OR “medication for opioid use disorder” OR “heroin assisted” OR “oral morphine” OR hydromorphone OR naltrexone OR "MAT" OR MOUD OR HAT OR **buprenorphine** OR **methadone** OR **opiate substitution treatment** OR **buprenorphine, naloxone drug combination** |

| **Search 7: Naloxone** |
| --- |
| Naloxone OR Narcan OR “take home naloxone” OR “take-home naloxone” OR THN OR **Naloxone** |

| **Search 8: COVID or HBV vaccinations** |
| --- |
| "COVID*" OR “SARS-CoV-2” OR **COVID-19 Vaccines** OR **Hepatitis B Vaccines** OR **COVID-19** |

| **Search 9: TB** |
| --- |
| **Tuberculosis** OR **“tuberculosis, multidrug-resistant”** OR **"Mycobacterium tuberculosis"** OR "Mycobacterium tuberculosis" OR “MDR-TB” OR “XDR-TB” OR “Mtb” OR (extensively AND **("drug resistance"** OR drug-resistant)) |

## 2.2. Embase Search Strategy

The search strategy was as follows:

Results were restricted to those articles containing terms from search 1 and terms from one or more results of searches 2-8, combined as 1 AND (2 OR 3 OR 4 OR 5 OR 6 OR 7 OR 8). Results were restricted to articles published 2000-current and limited to population group: humans.

Original searches were conducted in March 2023, the searches were then updated to 2^nd^ June 2025.

Key-words are noted in regular type, ‘EMTREE’ terms in **bold**.

| **Search 1: Incarceration settings** |
| --- |
| prison* OR jail OR gaol OR incarcerat* OR carceral OR detention OR compulsory and (drug OR treat* OR rehab*) OR offend* OR post-release OR custod* OR parole* OR probation OR crim* OR convict* OR detain* OR **prisons** OR **incarceration** OR **prisoners** OR **criminal** OR **parole** |

| **Search 2: Injecting drug use** |
| --- |
| IDU OR IDUs OR PWID OR "injecting drug*" OR "injecting substance*" OR "people who inject*" OR "injection drug*" OR **intravenous drug abuse/** OR **intravenous drug administration/** |

| **Search 3: HIV** |
| --- |
| HIV OR **HIV/** |

| **Search 4: Hepatitis** |
| --- |
| hepatitis OR "HBV" OR "hepatitis B" OR "hep B" OR "hep C" OR "HCV" OR "hepatitis C" OR **hepatitis B/** OR **hepatitis C/** |

| **Search 5: Needle syringe program** |
| --- |
| ("needle" AND ("exchange" OR "program*")) OR ("syringe" AND ("exchange" OR "program*")) OR “harm reduction” OR **needle-exchange programs/** OR **harm reduction/** |

| **Search 6: Opioid agonist treatment** |
| --- |
| "OST" OR "OAT" OR "MMT" OR "BMT" OR "MAT" OR "OUD" OR methadone OR buprenorphine* OR "opioid replacement" OR "opioid substitution" OR "opiate substitution" OR "opioid agonist" OR agonist OR "drug treatment" OR "medication assisted" OR "medication for opioid use disorder" OR "heroin assisted" OR "oral morphine" OR hydromorphone OR naltrexone OR "MOUD" OR "HAT" OR **buprenorphine** OR **methadone** OR **opiate substitution treatment** OR **buprenorphine, naloxone drug combination** |

| **Search 7: Naloxone** |
| --- |
| Naloxone OR Narcan OR THN OR **naloxone/** |

| **Search 8: COVID-19 or HBV vaccinations** |
| --- |
| "COVID*" OR “SARS-CoV-2” OR **COVID-19 Vaccines/** OR **Hepatitis B Vaccines/** OR **COVID-19/** |

| **Search 9: TB** |
| --- |
| **Tuberculosis/** OR'multidrug resistance' OR **Mycobacterium tuberculosis/ OR** 'mdr -tb' OR 'xdr -tb' OR 'mtb' OR 'tb' |

## 2.3. PsycINFO Search Strategy

The search strategy was as follows:

Results were restricted to those articles containing terms from search 1 and terms from one or more results of searches 2-8, combined as 1 AND (2 OR 3 OR 4 OR 5 OR 6 OR 7 OR 8). Results were restricted to articles published 2000-current and limited to population group: humans.

Original searches were conducted in March 2023, the searches were then updated to 2^nd^ June 2025.

Key-words in regular type, ‘PsycINFO thesaurus’ terms in **bold**.

| **Search 1: Incarceration settings** |
| --- |
| prison* OR jail OR gaol OR incarcerat* OR carceral OR detention OR compulsory and (drug OR treat* OR rehab*) OR offend* OR post-release OR custod* OR parole* OR probation OR crim* OR convict* OR detain* OR **prisons** OR **incarceration** OR **prisoners** OR **criminals** OR **parole** |

| **Search 2: Injecting drug use** |
| --- |
| IDU OR IDUs OR PWID OR “injecting drug*” OR “injecting substance*” OR "people who inject*" OR "injection drug*" OR (**"Intravenous Drug Usage"** OR **"Intravenous Injections"**) |

| **Search 3: HIV** |
| --- |
| HIV OR **"HIV"** |

| **Search 4: Hepatitis** |
| --- |
| hepatitis OR "HBV" OR "hepatitis B" OR "hep B" OR "hep C" OR "HCV" OR "hepatitis C" OR (**"Hepatitis"**) |

| **Search 5: Needle syringe program** |
| --- |
| ("needle" AND ("exchange" OR "program*")) OR ("syringe" AND ("exchange" OR "program*")) OR “harm reduction” OR (**"Needle-Exchange Programs"** OR **"Harm Reduction"**) |

| **Search 6: Opioid agonist treatment** |
| --- |
| ("OST" OR "OAT" OR "MMT" OR "BMT" OR "MAT" OR "OUD" “opioid substitution treatment” OR “methadone” OR “buprenorphine” OR “opioid replacement” OR “opioid substitution” OR “opiate substitution” OR “opioid agonist” OR “opiate agonist” OR “drug treatment” OR “medication assisted” OR “medication for opioid use disorder” OR “heroin assisted” OR “oral morphine” OR hydromorphone OR naltrexone OR "MOUD" OR "HAT").ti,ab. OR (**Buprenorphine** OR **Methadone** OR **opiate substitution treatment** OR **buprenorphine, naloxone drug combination**) |

| **Search 7: Naloxone** |
| --- |
| Naloxone OR Narcan OR THN OR (**"Naloxone"**) |

| **Search 8: COVID-19 and HBV vaccinations** |
| --- |
| "COVID*" OR “SARS-CoV-2” OR **COVID-19 Vaccines/** OR **Hepatitis B Vaccines/** OR **COVID-19/** |

| **Search 9: TB** |
| --- |
| **Tuberculosis/** OR 'multidrug resistance' OR 'mycobacterium tuberculosis' OR 'mdr-tb' OR 'xdr-tb' OR 'mtb' OR 'tb' |

## 2.4. Web of Science Search Strategy

The search strategy was as follows:

Results were restricted to those articles containing terms from search 1 and terms from one or more results of searches 2-8, combined as 1 AND (2 OR 3 OR 4 OR 5 OR 6 OR 7 OR 8). Results were restricted to articles published 2000-current and limited to population group: humans.

Original searches were conducted in March 2023, the searches were then updated to 2^nd^ June 2025. Key-words in regular type.

| **Search 1: Incarceration settings** |
| --- |
| prison* or jail or gaol or incarcerat* or "carceral system" or detention or compulsory and (drug or treat* or rehab*) or "offend*" or post-release or custod* or parole* or probation or crim* or convict* OR prisons OR incarceration OR prisoners OR "Criminal Offenders" OR "Criminal Conviction" OR Parole |

| **Search 2: Injecting drug use** |
| --- |
| "IDU" or "IDUs" or "PWID" or "injecting drug*" or "injecting substance*" or "people who inject*" or "injection drug*" or "intravenous drug" OR "intravenous drug abuse" or "intravenous drug administration" |

| **Search 3: HIV** |
| --- |
| “HIV” OR HIV |

| **Search 4: Hepatitis** |
| --- |
| hepatitis or "HBV" or "hepatitis B" or "hep B" or "hep C" or "HCV" or "hepatitis C" OR "hepatitis c" or "hepatitis b" |

| **Search 5: Needle syringe program** |
| --- |
| "needle" AND ("exchange" OR "program*") OR "harm reduction" OR "needle-exchange programs" OR "harm reduction" |

| **Search 6: Opioid agonist treatment** |
| --- |
| "OST" OR "OAT" OR "MMT" OR "BMT" OR "MAT" OR "OUD" “opioid substitution treatment” OR “methadone” OR “buprenorphine” OR “opioid replacement” OR “opioid substitution” OR “opiate substitution” OR “opioid agonist” OR “opiate agonist” OR “drug treatment” OR “medication assisted” OR “medication for opioid use disorder” OR “heroin assisted” OR “oral morphine” OR hydromorphone OR naltrexone OR "MOUD" OR "HAT" OR Methadone or Buprenorphine or "opiate substitution treatment" or "naloxone drug combination" |

| **Search 7: Naloxone** |
| --- |
| Naloxone OR Narcan OR “take home naloxone” OR “take-home naloxone” OR "THN" OR Naloxone |

| **Search 8: COVID-19 and HBV vaccinations** |
| --- |
| "COVID*" OR “SARS-CoV-2” OR "COVID-19 Vaccines" OR "Hepatitis B Vaccines" OR COVID-19 |

| **Search 9: TB** |
| --- |
| Tuberculosis OR 'mycobacterium tuberculosis' OR TB OR “MDR-TB” OR “XDR-TB” |

##

## 2.5. CINAHL Search Strategy

The search strategy was as follows:

Results were restricted to those articles containing terms from search 1 and terms from one or more results of searches 2-8, combined as 1 AND (2 OR 3 OR 4 OR 5 OR 6 OR 7 OR 8). Results were restricted to articles published 2000-current and limited to population group: humans.

Original searches were conducted in March 2023, the searches were then updated to 2^nd^ June 2025.

Key-words in regular type, ‘CINAHL Subject Headings’ terms in **bold**.

| **Search 1: Incarceration settings** |
| --- |
| Prisoners OR Prison* OR Jail* OR Detention* OR "Compulsory drug treatment" OR "Closed setting" OR Prisoners* OR Criminal* OR Offender* OR Post-release* |

| **Search 2: Injecting drug use** |
| --- |
| "IDU" OR "IDUs" OR PWID OR "injecting drug*" OR "intravenous drug*" OR "injecting substance*" OR "intravenous substance*" OR "people who inject*" OR "injection drug*" OR "substance abuse" OR intravenous |

| **Search 3: HIV** |
| --- |
| "HIV" OR "AIDS" OR "HIV/AIDS" OR “Human Immunodeficiency Virus” OR “Human Immune Deficiency Virus” OR “Acquired Immunodeficiency Syndrome” OR “Acquired Immune Deficiency Syndrome” |

| **Search 4: Hepatitis** |
| --- |
| (HCV or hepatitis C) and (test* or incidence* or prevalence*) |

| **Search 5: Needle syringe program** |
| --- |
| "needle" and ("exchange" OR "program*") OR "syringe" and "exchange" OR "program*") OR "harm reduction" OR "NSP" |

| **Search 6: Opioid agonist treatment** |
| --- |
| (((opioid OR opiate) AND (subtitut* OR maintenance) AND treatment) OR methadone OR buprenorphine OR ((medication* adj3 ("OUD" or ((opiate or opioid or heroin) and (dependen* or addict*)))) or "medication-assisted treatment" or "LAAM") OR "Narcotic Agonists" OR ((Opioid OR Opiate) AND "agonist treatment") OR ("OAT" OR "OST" OR "MMT" OR "BMT" OR "MAT")) |

| **Search 7: Naloxone** |
| --- |
| Naloxone or (naloxone and (provision or program or programme)) or "THN" |

| **Search 8: COVID-19 and HBV vaccinations** |
| --- |
| ("COVID*" or "SARS-CoV-2" or ("Hepatitis B" or "Hep B" or "HBV")) and (Vaccin* or immunisation or immunization or "vaccination coverage") |

| **Search 9: TB** |
| --- |
| Tuberculosis OR ‘mycobacterium tuberculosis’ OR “TB” OR “mdr-tb” OR “xdr-tb OR “mtb” |

# Appendix 3: Grey Literature Search

A wide range of online databases and websites were searched for additional information to that collected in the peer reviewed literature (see Table 3.1 below for full list). These databases are crucial sources of information on the epidemiology of injecting drug use and blood-borne virus among injecting drug users because so much of the work in this area appear to be published only in the form of reports. In total, we searched 145 websites and databases, including: government and substance use organisation websites, ongoing study sites, harm reduction organisation websites and infectious disease databases. The full technical report of grey literature sources is published elsewhere. ^1^

The search terms used in the grey literature search were similar to those used in the peer review search and are listed below. The ‘Google Advanced Search’ option was used to search for these terms within each website. Manual searching, involving clicking through relevant sections of the website in order to find relevant documents, was also conducted on some websites, particularly when the Google Advanced Search produced no results. Websites in languages other than English were also searched by translating the below search terms into English (United Kingdom and United States versions) through ‘Google Translate’. All searches were conducted in October 2023, with additional searches up until December 2024.

**Grey Literature Search Strategy:**

| **Search 1: Carceral Settings** |
| --- |
| prison OR criminal OR justice OR jail OR correctional OR detention OR incarcerated OR incarceration OR custody |

| **Search 2: Health** |
| --- |
| hiv OR "human immunodeficiency virus" OR hepatitis OR hcv OR hbv OR bbv OR inject OR needle OR nsp OR intravenous OR pwid OR condom OR opioid OR opiate OR heroin OR narcotic OR methadone OR buprenorphine OR fentanyl OR "OST" OR methamphetamine OR stimulant OR amphetamine OR cocaine OR "crack" OR naloxone OR naltrexone OR tuberculosis |

## Table 3.1: Websites searched in the grey literature search

| **Region** | **Website Name and Link** | **Search Strategy** | **Search Results (N)^1^** | **Sources/Web Pages with Relevant Data (N)**^2^ |
| --- | --- | --- | --- | --- |
| **Global** | Harm Reduction International  [https://hri.global/](https://hri.global/%20%20) | Search 1: Carceral Settings | 291 | 3 |
| **Global** | National Drug & Alcohol Research Centre  [https://ndarc.med.unsw.edu.au/](https://ndarc.med.unsw.edu.au/%20%20) | Search 1: Carceral Settings | 256 | 1 |
| **Global** | International Narcotics Control Board  [https://www.incb.org/](https://www.incb.org/%20%20) | Search 1: Carceral Settings | 0 | 0 |
| **Global** | International Narcotics Control Board  [https://www.incb.org/](https://www.incb.org/%20%20) | Search 1: Carceral Settings | 290 | 0 |
| **Global** | SALIS (Substance Abuse Librarians & Information Specialists)  <https://salis.org> | Search 1: Carceral Settings | 1 | 0 |
| **Global** | International Treatment Preparedness Coalition (ITPC)  <https://itpcglobal.org> | Search 1: Carceral Settings  Manual | 0  24 | 0  2 |
| **Global** | United Nations Office on Drugs and Crime  <http://www.unodc.org> | Manual | 4340 |  |
| **Global** | United Nations Office on Drugs and Crime  <https://dataunodc.un.org> | Search 1: Carceral Settings | 3 | 0 |
| **Global** | United Nations Office on Drugs and Crime  [https://www.unodc.org/unodc/en/treatment-and-care/publications.html](https://www.unodc.org/unodc/en/treatment-and-care/publications.html%20) |  | n/a | n/a |
| **Global** | United Nations Office on Drugs and Crime  [https://www.unodc.org/unodc/en/justice-and-prison-reform/cpcj-tools-prisonreform.html](https://www.unodc.org/unodc/en/justice-and-prison-reform/cpcj-tools-prisonreform.html%20) |  | n/a | n/a |
| **Global** | United Nations Office on Drugs and Crime  [https://www.unodc.org/unodc/en/crimecongress/about.html](https://www.unodc.org/unodc/en/crimecongress/about.html%20%20) |  | n/a | n/a |
| **Global** | United Nations Office on Drugs and Crime  [https://www.unodc.org/unodc/en/commissions/CND/index.html](https://www.unodc.org/unodc/en/commissions/CND/index.html%20) | Search 1: Carceral Settings | 1 | 0 |
| **Global** | United Nations Office on Drugs and Crime  [https://www.unodc.org/unodc/en/commissions/CCPCJ/index.html](https://www.unodc.org/unodc/en/commissions/CCPCJ/index.html%20) | Search 1: Carceral Settings | 1 | 0 |
| **Global** | United Nations Office on Drugs and Crime  [https://www.unodc.org/unodc/en/commissions/CCPCJ/PNI/institutes-UNAFEI.html](https://www.unodc.org/unodc/en/commissions/CCPCJ/PNI/institutes-UNAFEI.html%20) | Search 1: Carceral Settings | 1 | 0 |
| **Global** | United Nations Interregional Crime and Justice Research Institute  [https://unicri.it/](https://unicri.it/%20) | Search 1: Carceral Settings | 302 | 0 |
| **Global** | World Health Organization  <https://www.who.int/europe/home> | Search 1: Carceral Settings | 0 | 0 |
| **Global** | World Health Organization  <https://www.who.int/data/gho> | Search 1: Carceral Settings | 1 | 0 |
| **Global** | World Health Organization  <https://www.who.int/teams/mentalhealth-and-substance-use/alcohol-drugsand-addictive-behaviours/overview> | Search 1: Carceral Settings | 276 | 0 |
| **Global** | United Nations Statistics Division  <https://unstats.un.org/home/nso_sites> | Search 1: Carceral Settings | 280 |  |
| **Global** | National Commission on Correctional Health Care  [https://www.ncchc.org/](https://www.ncchc.org/%20) | Search 1: Carceral Settings | 300 | 0 |
| **Global** | Penal Reform International  <https://www.penalreform.org/globalprison-trends-2022> | Manual | 1 | 1 |
| **Global** | International Committee of the Red Cross  <https://www.icrc.org/en> | Manual | 271 | 0 |
| **Global** | The Worldwide Prison Health Research & Engagement Network (WEPHREN)  <https://wephren.tghn.org> | Manual | 186 | 0 |
| **Global** | Coalition for Global Hepatitis Elimination  [https://www.globalhep.org/data-dashboards/national-hepatitis-elimination-profiles](https://www.globalhep.org/data-dashboards/national-hepatitis-elimination-profiles%20) | Manual | 1 | 1 |
| **Global** | HIV and AIDS Data Hub  <https://www.aidsdatahub.org> | Search 1: Carceral Settings | 70 | 0 |
| **Global** | Eurasian Harm Reduction Association  [https://harmreductioneurasia.org/drug-policy/criminalization-costs-2](https://harmreductioneurasia.org/drug-policy/criminalization-costs-2%20) | Search 1: Carceral Settings | 244 | 2 |
| **Global** | Virtual AIDS Office of Hong Kong  [https://www.aids.gov.hk/english/surveillance/off_surreport.html](https://www.aids.gov.hk/english/surveillance/off_surreport.html%20%20) | Search 1: Carceral Settings | 100 | 0 |
| **Global** | Narcotics Division, Security Bureau – The Government of the Hong Kong Special Administrative Region of the People’s Republic of China  [https://www.nd.gov.hk/en/index.html](https://www.nd.gov.hk/en/index.html%20) | Manual | 308 | 0 |
| **Global** | Nai Zindagi Trust  [https://www.naizindagi.org/](https://www.naizindagi.org/%20) | Search 1: Carceral Settings | 30 | 0 |
| **Global** | Recovering Nepal  [www.recoveringnepal.org.np](http://www.recoveringnepal.org.np) | Search 1: Carceral Settings | 20 | 0 |
| **Global** | Be in the Know  [https://www.beintheknow.org/understanding-hiv-epidemic/community/hiv-and-prisoners](https://www.beintheknow.org/understanding-hiv-epidemic/community/hiv-and-prisoners%20%20) | Search 1: Carceral Settings  Manual | 0  0 | 0  0 |
| **Global** | FHI360  <https://www.fhi360.org> | Search 1: Carceral Settings | 200 | 0 |
| **Global** | Global Network of People Living with HIV  <https://gnpplus.net/> | Search 1: Carceral Settings | 192 | 0 |
| **Global** | Open Society Foundations  <https://www.opensocietyfoundations.org/voices/%20topics/criminal-justice> | Search 1: Carceral Settings | 1 | 0 |
| **Global** | HIV and AIDS Data Hub  <https://www.aidsdatahub.org/resource/technicalbrief-addressing-hiv-and-tb-prisons-pre-trialdetention-and-other-closed> | Search 1: Carceral Settings | 2 | 0 |
| **Global** | The Global Fund  <https://www.theglobalfund.org/en> | Search 1: Carceral Settings  Manual | 0  1120 | 0  1 |
| **Global** | United Nations Development Programme  <https://www.undp.org> | Search 1: Carceral Settings  Manual | 0  4083 | 0  0 |
| **Global** | United Nations Population Fund  <https://www.unfpa.org> | Search 1: Carceral Settings | 2 | 0 |
| **Asia** | HIV and AIDS Data Hub  [https://www.aidsdatahub.org/](https://www.aidsdatahub.org/%20%20) | Search 1: Carceral Settings | 295 | 1 |
| **Asia** | Eurasian Harm Reduction Association  [https://harmreductioneurasia.org/dru g-policy/criminalization-costs-2](https://harmreductioneurasia.org/dru%20g-policy/criminalization-costs-2) | Manual | 29 | 29 |
| **Asia** | Virtual AIDS Office of Hong Kong  [https://www.aids.gov.hk/english/surv eillance/off_surreport.html](https://www.aids.gov.hk/english/surv%20eillance/off_surreport.html%20%20) | Search 1: Carceral Settings | 0 | 0 |
| **Asia** | Virtual AIDS Office of Hong Kong  [https://www.aids.gov.hk/english/surv eillance/off_surreport.html](https://www.aids.gov.hk/english/surv%20eillance/off_surreport.html%20%20) | Manual | 23 | 1 |
| **Asia** | Narcotics Division, Security Bureau – The Government of the Hong Kong Special Administrative Region of the People’s Republic of China  [https://www.nd.gov.hk/en/index.html](https://www.nd.gov.hk/en/index.html%20) | Search 1: Carceral Settings  Manual | 0  309 | 0  0 |
| **Asia** | Nai Zindagi Trust  <https://www.naizindagi.org> | Search 1: Carceral Settings | 30 | 0 |
| **Asia** | Recovering Nepal  [www.recoveringnepal.org.np](http://www.recoveringnepal.org.np) | Search 1: Carceral Settings | 26 | 0 |
| **Asia** | The Asia Foundation  <https://asiafoundation.org> | Search 1: Carceral Settings  Manual | 0  1 | 0  0 |
| **Asia** | International Drug Policy Consortium  <https://idpc.net> | Manual | 107 | 0 |
| **Asia** | National AIDS Control Organisation  [http://naco.gov.in/](http://naco.gov.in/%20) | Search 1: Carceral Settings | 251 | 2 |
| **Asia** | National Center for AIDS and STD Control – Government of Nepal – Ministry of Health and Population  <https://www.ncasc.gov.np> | Search 1: Carceral Settings  Manual | 0  1 | 0  1 |
| **Asia** | Bureau of Jail Management and Penology  <https://www.bjmp.gov.ph> | Search 2: Health | 2 | 0 |
| **Asia** | Correctional Services Department Hong Kong  <https://www.csd.gov.hk/tc_chi/home/home.html> | Search 2: Health  Manual | 0  0 | 0  0 |
| **Asia** | Ministry of Justice – Agency of Corrections  <https://www.mjac.moj.gov.tw> | Search 2: Health | 104 | 2 |
| **Asia** | Singapore Prison Service  <https://www.sps.gov.sg> | Search 2: Health | 6 | 1 |
| **Asia** | Malaysian Prison Department  <https://www.prison.gov.my/ms> | Search 2: Health | 23 | 0 |
| **Asia** | Prisons Organization of Iran  <https://www.prisons.ir> | Search 2: Health | 8 | 1 |
| **Australasia** | Australian Institute of Health and Welfare  <https://www.aihw.gov.au> | Search 1: Carceral Settings | 256 | 1 |
| **Australasia** | Australian Government – Attorney-General’s Department  <https://www.ag.gov.au/crime/federaloffenders/state-and-territorycorrective-services-websites> | Manual | 86 | 1 |
| **Australasia** | Department of Health and Aged Care  http://www.health.gov.au | Search 1: Carceral Settings | 249 | 0 |
| **Australasia** | Alcohol and Drug Foundation  <https://adf.org.au> | Search 1: Carceral Settings | 247 | 0 |
| **Australasia** | Ministry of Health Manatu Hauora  <https://www.health.govt.nz/nzhealth-statistics/health-statistics-anddata-sets/prisoner-health-data-andstats> | Search 1: Carceral Settings | 1 | 0 |
| **Australasia** | Kirby Institute  <http://kirby.unsw.edu.au/> | Search 1: Carceral Settings  Manual | 0  11 | 0  2 |
| **Australasia** | Justice and Community Safety Directorate  <https://justice.act.gov.au> | Search 2: Health | 2 | 0 |
| **Australasia** | Department of Justice – Government of Western Australia  [https://www.wa.gov.au/organisation/department-of-justice](https://www.wa.gov.au/organisation/department-of-justice%20) | Search 2: Health | 1 | 0 |
| **Australasia** | Justice and Community Safety – Victoria State Government  <https://www.justice.vic.gov.au> | Search 2: Health | 15 | 0 |
| **Australasia** | Department of Corrections – Ara Poutama Aotearoa  <http://www.corrections.govt.nz> | Search 2: Health | 20 | 0 |
| **Europe** | European Union Drugs Agency  <https://www.emcdda.europa.eu/topics/prison_en> | Search 1: Carceral Settings  Manual | 1  71 | 0  26 |
| **Europe** | Department of Health & Social Care United Kingdom Government  <https://www.gov.uk/health-andsocial-care/drug-misuse-and-dependency> | Search 1: Carceral Settings  Manual | 0  27 | 0  2 |
| **Europe** | Federal Ministry Republic of Austria – Social Affairs, Health, Care and Consumer Protection  [https://www.sozialministerium.at/en.html](https://www.sozialministerium.at/en.html%20) | Search 1: Carceral Settings  Manual | 0  0 | 0  0 |
| **Europe** | Sciensano  [https://www.sciensano.be/en](https://www.sciensano.be/en%20%20) | Search 1: Carceral Settings | 85 | 0 |
| **Europe** | Ministry of Health Bulgaria  <https://www.mh.government.bg/%20bg/novini/aktualno/v-sofiiskiiazatvor-shche-uchat-kak-da-sepaziat-o/> | Search 1: Carceral Settings  Manual | 0  1 | 0  1 |
| **Europe** | National Monitoring Center for Drugs and Addiction [https://www.drogyinfo.cz/data/obj_files/33369/107 3/VZdrogy2019_www_fin.pdf](https://www.drogyinfo.cz/data/obj_files/33369/107%203/VZdrogy2019_www_fin.pdf) | Search 1: Carceral Settings  Manual | 0  0 | 0  0 |
| **Europe** | Statens Serum Institut  <https://en.ssi.dk/news/epinews/2016/no-9---2016> | Search 1: Carceral Settings | 8 | 0 |
| **Europe** | DrugWise  <http://www.drugwise.org.uk/> | Search 1: Carceral Settings | 296 | 0 |
| **Europe** | United Kingdom Government  <https://www.gov.uk/government%20/organisations/public-healthengland> | Search 1: Carceral Settings  Manual | 0  0 | 0  0 |
| **Europe** | The French Monitoring Centre for Drugs and Addiction (OFDT)  [https://en.ofdt.fr/publications/m emo-posters-maps/drug-useprison-practices-consequencesand-responses-summary/](https://en.ofdt.fr/publications/m%20emo-posters-maps/drug-useprison-practices-consequencesand-responses-summary/%20%20) | Search 1: Carceral Settings | 1 | 1 |
| **Europe** | Sante Publique France  <https://www.santepubliquefrance.fr/recherche/#search=prison> |  | n/a | n/a |
| **Europe** | Robert Koch Institut  <https://www.rki.de/SiteGlobals/Forms/Suche/en/serviceSucheFormen> | Search 1: Carceral Settings  Manual | 0  24 | 0  0 |
| **Europe** | Public Health Scotland  <https://www.hps.scot.nhs.uk/search?q=prison> | Search 1: Carceral Settings | 9 | 0 |
| **Europe** | Public Health Scotland  <https://www.isdscotland.org/search/?q=prison> | Search 1: Carceral Settings | 229 | 2 |
| **Europe** | The Scottish Public Health Observatory  <https://www.scotpho.org.uk/search> | Search 1: Carceral Settings | 153 | 1 |
| **Europe** | National Institute of Health Italy  http://www.iss.it | Search 1: Carceral Settings | 216 | 0 |
| **Europe** | Ministry of Health of the Republic of Lithuania  <http://sam.lrv.lt/> | Search 1: Carceral Settings | 63 | 1 |
| **Europe** | National Institute for Public Health and the Environment Netherlands  <https://www.rivm.nl/en/search?search=prison> | Search 1: Carceral Settings | 19 | 0 |
| **Europe** | Instituto de Salud Carlos III  [https://www.isciii.es/en/layouts/15/osssearchresults.aspx#k=prisi%C%203%B3n#s=11](https://www.isciii.es/en/layouts/15/osssearchresults.aspx#k=prisi%C%203%B3n) | Search 1: Carceral Settings | 21 | 0 |
| **Europe** | Federal Office of Public Health Switzerland  [https://www.bag.admin.ch/bag/e n/home/strategie-undpolitik/nationalegesundheitsstrategien/nationales-programm-hiv-und-anderesexuell-uebertragbareinfektionen/zielgruppe-miterhoehtem-expositionsrisikoachse2/bekampfung-voninfektionskrankheiten-imgefangnis.html](https://www.bag.admin.ch/bag/e%20n/home/strategie-undpolitik/nationalegesundheitsstrategien/nationales-programm-hiv-und-anderesexuell-uebertragbareinfektionen/zielgruppe-miterhoehtem-expositionsrisikoachse2/bekampfung-voninfektionskrankheiten-imgefangnis.html) | Search 1: Carceral Settings | 23 | 0 |
| **Europe** | Eurasian Harm Reduction Association  [https://harmreductioneurasia.org/](https://harmreductioneurasia.org/%20) | Search 1: Carceral Settings | 244 | 0 |
| **Europe** | French National AIDS Council  <https://cns.sante.fr/> | Search 1: Carceral Settings | 205 | 1 |
| **Europe** | Health Protection Surveillance Centre  <https://www.hpsc.ie> | Search 1: Carceral Settings | 233 | 1 |
| **Europe** | Country Coordinating Mechanism of Moldova  <http://www.ccm.md/index.php/> | Search 1: Carceral Settings | 8 | 0 |
| **Europe** | UK Harm Reduction Alliance  <https://www.ukhra.org/index.html> | Search 1: Carceral Settings  Manual | 0  0 | 0  0 |
| **Europe** | Ministry of Justice Republic Moldova  <http://anp.gov.md/> | Search 2: Health | 50 | 1 |
| **Europe** | Prison Service of the Czech Republic  <https://www.vscr.cz/> | Search 2: Health | 77 | 1 |
| **Europe** | National Penitentiary Administration Romania  <https://anp.gov.ro/> | Search 2: Health | 290 | 0 |
| **Europe** | Prison Service Estonia  <https://www.vangla.ee> | Search 2: Health | 3 | 1 |
| **Europe** | Prison Service Poland  <https://sw.gov.pl> | Search 2: Health | 229 | 0 |
| **Europe** | Special Penitentiary Service of Georgia  <http://www.sps.gov.ge> | Search 2: Health | 44 | 0 |
| **Europe** | Service Public Federal Justice  [https://justice.belgium.be/fr/themes_et_dossiers/prisons](https://justice.belgium.be/fr/themes_et_dossiers/prisons%20) | Search 2: Health | 1 | 0 |
| **Europe** | Directorate-General for Reintegration and Prison Services Portugal  <https://dgrsp.justica.gov.pt> | Search 2: Health | 35 | 0 |
| **Europe** | Norwegian Correctional Service  <https://www.kriminalomsorgen.no> | Search 2: Health | 23 | 0 |
| **Europe** | Irish Prison Service  <https://www.irishprisons.ie> | Search 2: Health | 92 | 0 |
| **Europe** | Ministry of Justice France  <http://www.justice.gouv.fr> | Search 2: Health  Manual | 1  8 | 0  0 |
| **Europe** | Department of Justice Northern Ireland  <https://www.justice-ni.gov.uk/topics/prisons> | Search 2: Health  Manual | 0  0 | 0  0 |
| **Europe** | Ministry of Justice Albania  <https://dpbsh.gov.al> | Search 2: Health | 18 | 0 |
| **Europe** | Scottish Prison Service  <https://www.sps.gov.uk/> | Search 2: Health | 2 | 0 |
| **Europe** | Directorate of the Danish Prison and Probation Service  [https://www.kriminalforsorgen.dk/](https://www.kriminalforsorgen.dk/%20) | Manual | 188 | 0 |
| **Europe** | Criminal Sanctions Agency Finland  [https://rikosseuraamus.fi/](https://rikosseuraamus.fi/%20) | Manual | 293 | 0 |
| **Europe** | Ministry of Justice – Directorate for the Execution of Criminal Sanctions Serbia  <http://www.uiks.mpravde.gov.rs/> | Manual | 3 | 0 |
| **Europe** | HM Prison Service – Public Sector Prisons UK  [https://www.gov.uk/government/organisations/hm-prison-service](https://www.gov.uk/government/organisations/hm-prison-service%20) | Search 2: Health | 2 | 0 |
| **Europe** | Ministry of Justice Italy  [https://www.giustizia.it/giustizia/](https://www.giustizia.it/giustizia/%20) | Search 2: Health | 40 | 0 |
| **Africa** | Ministry of Health and Wellness Mauritius  <https://health.govmu.org/Pages/default.aspx> | Search 1: Carceral Settings  Manual | 0  2 | 0  1 |
| **Africa** | Ghana AIDS Commission  [https://www.ghanaids.gov.gh/](https://www.ghanaids.gov.gh/%20) | Search 1: Carceral Settings  Manual | 0  6 | 0  1 |
| **Africa** | The National Syndemic Diseases Control Council (NSDCC)  <https://nsdcc.go.ke/> | Search 1: Carceral Settings  Manual | 0  11 | 0  0 |
| **Africa** | National AIDS Council Zimbabwe  <https://www.nac.org.zw/> | Search 1: Carceral Settings  Manual | 0  8 | 0  0 |
| **Africa** | The South African National AIDS Council  <http://www.sanac.org.za/> | Search 1: Carceral Settings  Manual | 0  7 | 0  0 |
| **Africa** | Ministry of Justice - Prisons Directorate Lebanon  <http://pa.justice.gov.lb/index.php> | Search 1: Carceral Settings  Manual | 0  0 | 0  0 |
| **Africa** | Sierra Leone Correctional Service  <https://slcs.gov.sl/> | Search 2: Health  Manual | 0  0 | 0  0 |
|  | Nigerian Correctional Service  [https://corrections.gov.ng/](https://corrections.gov.ng/%20%20) | Search 2: Health | 4 |  |
| **Africa** | Malawi Prisons Service  [https://www.mps.gov.mw/](https://www.mps.gov.mw/%20) | Manual | 30 | 0 |
| **Africa** | Seychelles Prison Service  <http://www.prisonservice.gov.sc/> | Manual | 20 | 0 |
| **Americas** | Correctional Service of Canada  <https://www.csc-scc.gc.ca/health/002006index-en.shtml> | Search 1: Carceral Settings  Manual | 118  407 | 1  2 |
| **Americas** | Canadian Centre on Substance Use and Addiction  [https://www.ccsa.ca/](https://www.ccsa.ca/%20%20) | Search 1: Carceral Settings  Manual | 59  401 | 0  0 |
| **Americas** | Government of Canada  [https://www.canada.ca/en/publichealth.html](https://www.canada.ca/en/publichealth.html%20) | Search 1: Carceral Settings | 374 | 3 |
| **Americas** | Substance Abuse and Mental Health Services Administration  [https://www.samhsa.gov/](https://www.samhsa.gov/%20%20) | Search 1: Carceral Settings | 684 | 0 |
| **Americas** | Bureau of Justice Statistics  <https://bjs.ojp.gov> | Search 2: Health  Manual | 120 | 3  0 |
| **Americas** | Correctional Health – Centers for Disease Control and Prevention  [https://www.cdc.gov/correctionalhealth/](https://www.cdc.gov/correctionalhealth/%20) | Manual | 120 | 0 |
| **Americas** | Partnership to End Addiction  <https://drugfree.org/> | Search 1: Carceral Settings | 10 | 0 |
| **Americas** | National Center for Health Statistics - Centers for Disease Control and Prevention  <https://www.cdc.gov/nchs/index.htm> | Search 1: Carceral Settings  Manual | 284  621 | 1  7 |
| **Americas** | The White House  <https://www.whitehouse.gov/ondcp> | Search 1: Carceral Settings | 96 | 0 |
| **Americas** | Inter-American Drug Abuse Control Commission  <http://www.cicad.oas.org/main/default_eng.asp> | n/a | n/a | n/a |
| **Americas** | National Prevention Information Network – Centers for Disease Control and Prevention  <https://npin.cdc.gov/> | n/a | n/a | n/a |
| **Americas** | National Harm Reduction Coalition  [https://harmreduction.org/](https://harmreduction.org/%20) | Search 1: Carceral Settings | 142 | 0 |
| **Americas** | Department of HIV, AIDS, Tuberculosis, Viral Hepatitis and Sexually Transmitted Infections Brazil  <http://www.aids.gov.br> | Search 1: Carceral Settings | 50 | 0 |
| **Americas** | HIV Legal Network Canada  <https://www.hivlegalnetwork.ca/site/?lang=en> | Search 1: Carceral Settings | 295 | 0 |
| **Americas** | The National Council for Comprehensive HIV AIDS Care (CONASIDA)  [http://www.conasida.go.cr/](http://www.conasida.go.cr/%20%20) | Search 1: Carceral Settings | 172 | 0 |
| **Americas** | Ministry of Health and Wellness Jamaica  [https://www.moh.gov.jm/](https://www.moh.gov.jm/%20%20) | Search 1: Carceral Settings | 72 | 0 |
| **Americas** | Department of Correctional Services Jamaica  <https://www.dcs.gov.jm/> | Search 2: Health | 5 | 0 |
| **Americas** | Department of Corrections and Rehabilitation Puerto Rico  [http://dcr.pr.gov/](http://dcr.pr.gov/%20) | Manual | n/a | n/a |
| **Americas** | Ministry of Home Affairs, Justice and National Security  [https://homeaffairs.govt.lc/](https://homeaffairs.govt.lc/%20) | Manual | 20 | 0 |
| **Americas** | National Secretariat for Penal Policies – Ministry of Justice and Public Security Brazil  [https://www.gov.br/depen/pt-br](https://www.gov.br/depen/pt-br%20) | Manual | 173 | 0 |
| **Americas** | INPEC National Penitentiary and Prison Institute Colombia  [https://www.inpec.gov.co/](https://www.inpec.gov.co/%20) | Manual | 294 | 0 |
| **Americas** | General Directorate of the Penitentiary System Guatemala  https://dgsp.gob.gt | Manual | 201 | 0 |
| **Americas** | Guyana Prison Service  [https://gps.moha.gov.gy/](https://gps.moha.gov.gy/%20) | Manual | 51 | 0 |
| **Americas** | General Directorate of the Penitentiary System Panama  <https://www.sistemapenitenciario.gob.pa/> | Manual | 299 | 0 |
| **Americas** | Ministry of People's Power for the Penitentiary Service Venezuela  [https://www.mppsp.gob.ve/](https://www.mppsp.gob.ve/%20) | Manual | 283 | 0 |
| **Americas** | Ministry of Justice and Public Security El Salvador  [https://www.seguridad.gob.sv/](https://www.seguridad.gob.sv/%20) | Manual | 282 | 0 |
| **Americas** | Statistics Canada  <https://www.statcan.gc.ca/en/subjectsstart/crime_and_justice/correctional_services> | Manual | 256 | 0 |

**Notes: 1. Search results** refers to sources/web pages found in search results that had potential data for inclusion **2. Sources/Web Pages with Relevant Data** refers to sources that were included for full text review

# Appendix 4: Additional requests for information

## 4.1 Example email

**NDARC and Kirby Institute** **researchers gathering global data on people who inject drugs**

We hope this message finds you well.

We are conducting a global review of the prevalence and treatment practices related to blood-borne viruses and injecting drug use among incarcerated populations.

Perhaps you recall our previous work on the epidemiology of injecting drug use, the prevalence of injecting-related harm, and exposure to behavioural and environmental risks among people who inject drugs (see attached). That research was intended to inform the provision of services, such as blood-borne virus treatment, for people who inject drugs globally.

In our current review, we aim to extend our work to estimate the prevalence of injecting drug use, blood-borne viruses, and related harms, and summarise the state of treatment coverage within prisons for injecting drug use and related harms. A summary of these reviews is attached for your reference.

We are keen to gain access particularly to reports and papers that may not be accessible online. Given your expertise, we were hoping you could assist by providing any reports or data you may be aware of related to injecting drug use, HIV, HCV, other infectious diseases and treatment among people who are incarcerated?

We will acknowledge all people who submit literature for our consideration in our review.

Thank you for considering this request.

**4.2 Social media advertising**

An advertisement was posted on Facebook and Twitter to request relevant information during April-June 2024.

### 4.2.1 Facebook advertisement example

**
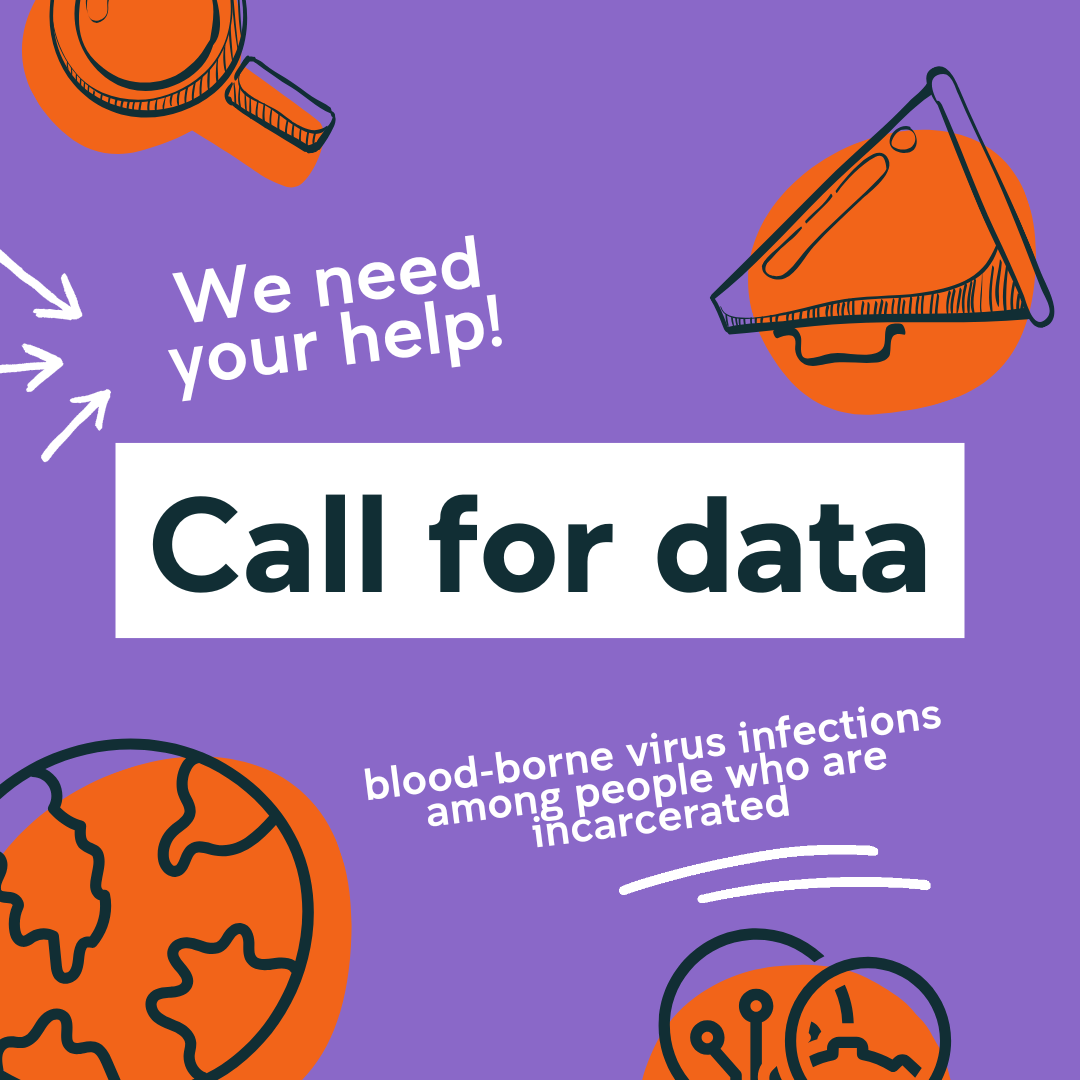
**

### 4.2.2 Twitter advertisement example


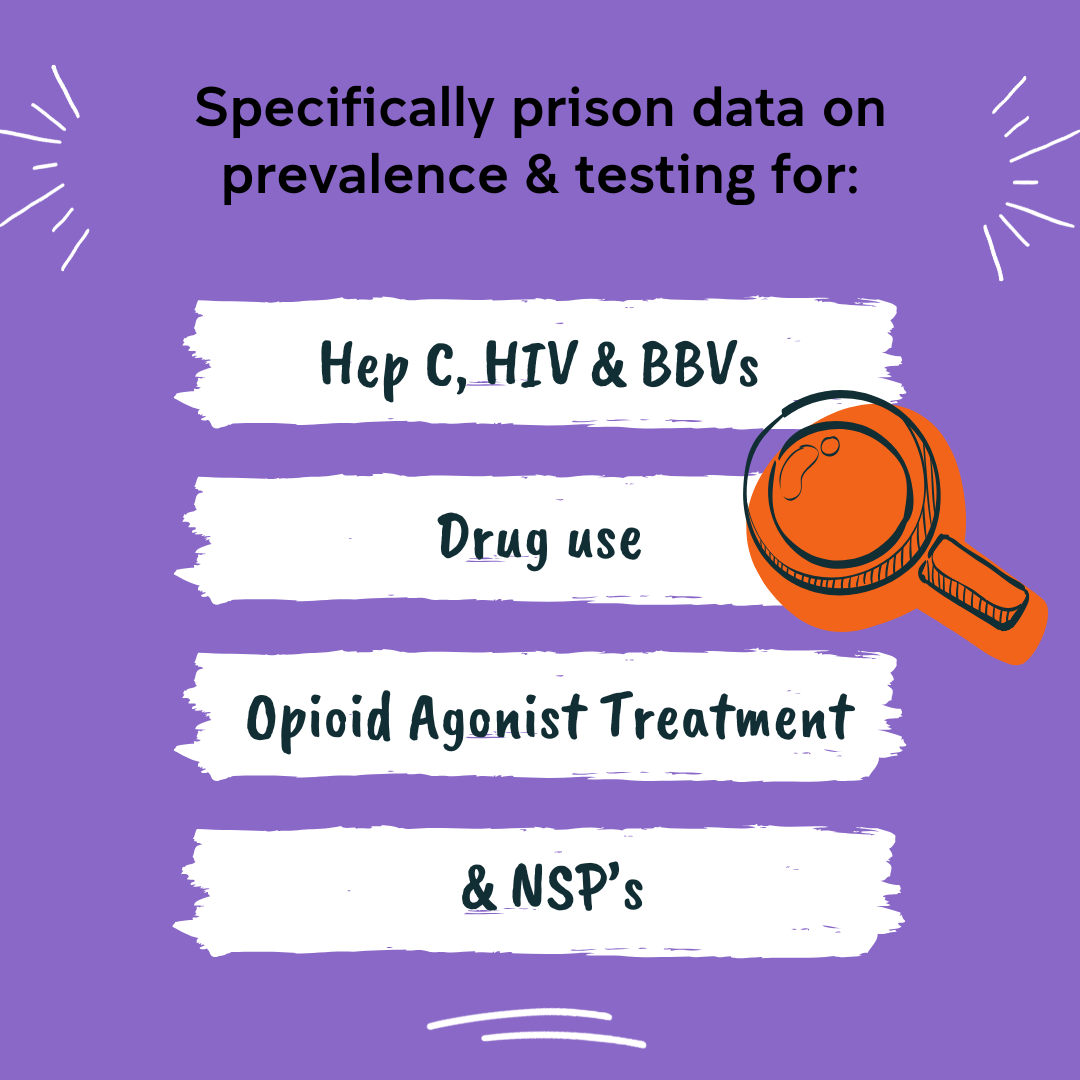


# Appendix 5: Screening Process and Criteria

Initial screening of title and abstract was conducted by two reviewers: Sophia Taylor (ST), Louisa Degenhardt (LD), Behzad Hajarizadeh (BH), Evan Cunningham (EC), Michelle Lynch (ML), Sophie Ottaviano (SO), Aleksa Kamenjaš (AK), Paige Webb (PW), Benson Yiu (BY), Brodie Clark (BC), Marion Barault (MB), Jason Grebely (JG), Thomas Santo Jr (TS), Rachel Jenkins (RJ), or Olivia Price (OP).

Full-text review was also independently conducted by two reviewers: AK, ST, BY, Jack Marsden (JM), ML, LD, Jonathan Wu (JW), Noni O’Dea (NO), MB, TS, BC, Eunice Ling (EL), OP, or Christopher Manning (CM).

All discrepancies at both stages were resolved by consensus.

**Inclusion criteria**

- Primary observational studies of people in prisons or other carceral settings that are likely to include one of the following outcomes:
  - Any study reporting on the correct sample, that is people in incarceration settings and reporting on infectious diseases or drug use
- Papers with information on imprisonment rates or estimated prison population sizes
- Relevant 100% subpopulation samples (e.g. 100% BBV, 100% TB, 100% IDU etc.)
- Relevant systematic reviews for reference checking

**Exclusion Criteria**

- Commentary, editorial, or review
- Case study, or sample size < 40 incarcerated people
- No primary data presented
- Modelling studies
- Methodology not reported
- Delphi, consensus, or government estimate with no methodology reported
- Duplicate, or conference abstract where full report is not available
- Registration or notification of cases as an indicator of # of people positive
- Non-human sample
- People in closed psychiatric settings
- Arrestee samples (exclude ADAM samples)
- Samples of people post-release
- Subpopulations of people who are incarcerated
  - Samples of specific race groups (e.g. African American incarcerated population)
  - Samples of offenders who are referred to mental health or hospital services whilst admitted
  - Samples of specific offence types (e.g. Sexual offenders, violent offenders), with the exemption of people incarcerated for drug offences
  - 100% negative infectious disease sample (e.g. All inmates were anti-HCV ab negative)
  - Samples of people incarcerated who have never used illicit drugs.

# Appendix 6: Classification system used in the assessment of study methodologies

## Appendix 6.1: Classification approach for grading studies

Studies were graded based on the geographical scope of their sample size:

- **National**: Grade A
- **Sub-National (e.g., state or regional level)**: Grade B
- **City-level studies**: Grade C
- **Single facility**: Grade D

# Appendix 7: Summary of decision rules for data extraction and estimation processes

**Overall:**

- Estimates with sample sizes <40 were excluded.
- Samples which represented a subpopulation (e.g., all injecting drug users, or all HIV+) were excluded.
- Where multiple sources were identified using data from the same sample, the source(s) with the most complete data regarding the various indicators of interest were included.
- Where possible, if calculation or typesetting errors were detected in reported estimates, these were recalculated.
- Only adult samples were included in prevalence estimates for all outcomes. Juvenile samples were excluded from the analysis.

**Prevalence of Injecting Drug Use:**

- Estimates of ever or lifetime injecting were selected in preference to estimates for injecting drug use during current incarceration, past 12 month injecting drug use or injecting defined by other criteria due to greater data availability for ever/lifetime injecting.

**Prevalence of Infectious Diseases:**

- To account for the differences between the included infectious diseases, the following decisions were made:

**HIV:**

- Data collected using both clinical records and testing or screening as part of the study process were included in the extraction and analysis. Data reported based on case notifications, self-report or where the reported methodology was unclear were excluded.
- For estimates of the prevalence of HIV among people who are incarcerated in Australian carceral settings, data was taken from the AusHep study^1^, allowing us to report more recent and complete estimates of HIV among people who are incarcerated in Australia.

**HCV:**

- Data collected using both clinical records and testing or screening as part of the study process were included in the extraction and analysis. Data reported based on case notifications, self-report or where the reported methodology was unclear were excluded.
- Only data indicating current HCV infection were included in generated HCV prevalence estimates. Current HCV infection was estimated using data reporting on HCV RNA. In countries where these data were unavailable, HCV-antibody prevalence data was used, assuming a 25% clearance proportion. This method has been used previously^2^.
- For estimates of the prevalence of HCV among people who are incarcerated in Australian carceral settings, data was taken from the AusHep study^1^, allowing us to report more recent and complete estimates of HCV among people who are incarcerated in Australia.

**HBV:**

- Data collected using both clinical records and testing or screening as part of the study process were included in the extraction and analysis. Data reported based on case notifications, self-report or where the reported methodology was unclear were excluded.
- Only data on HBV surface antigen (i.e. HBsAg) were included in data extraction and analysis. Where HBV infection was reported but the serological marker was unclear, these data were excluded.
- For estimates of the prevalence of HBV among people who are incarcerated in Australian carceral settings, data was taken from the AusHep study^1^, allowing us to report more recent and complete estimates of HBV among people who are incarcerated in Australia.

**Tuberculosis**

- Data collected using testing or screening as part of the study process were included in the extraction and analysis. Data reported based on clinical records, case notifications, self-report or where the reported methodology was unclear were excluded.
- Only data indicating the prevalence of active tuberculosis were included in data extraction and analysis. Where data reporting on the prevalence of tuberculosis did not specify whether they were reporting on active or latent tuberculosis, these data were excluded.
- For studies reporting screening data, where previously known cases or those on tuberculosis treatment were excluded from screening, an ‘overall prevalence’ was extracted that included those positive through serological screening and these previously known cases.
- Where a study reported an overall prevalence of active tuberculosis that differed from those reported as positive through serological testing, the reported overall prevalence was taken as preference due to different regional/setting approaches to calculating prevalence based.
- For studies that did not state an overall prevalence, an overall prevalence was calculated on a case-by-case basis taking into consideration the context of the study. This process was completed in consultation with a tuberculosis expert.

**References**

1. Bah R, Sheehan Y, Li X, et al. Prevalence of blood-borne virus infections and uptake of hepatitis C testing and treatment in Australian prisons: the AusHep study. The Lancet Regional Health–Western Pacific 2024; 53.

2. Grebely J, Larney S, Peacock A, et al. Global, regional, and country-level estimates of hepatitis C infection among people who have recently injected drugs. Addiction (Abingdon, England) 2018.

# Appendix 8: Method of estimating regional and global numbers

A range of situations existed with respect to the amount of data available on the prevalence of injecting drug use and of HIV, HCV, tuberculosis and HBsAg among people who are incarcerated. Using the following protocol, available country level data were extrapolated to derive regional and global estimates of the number of people who are incarcerated who are HIV, HCV, TB or HBsAg positive.

Country-level estimates were pooled in STATA 18 via random-effects using the metaprop command. This was done separately for male and female estimates (for sources which disaggregated by sex), and mixed estimates (for sources which did not disaggregate by sex). The confidence intervals (CIs) were computed using an exact method (the Clopper-Pearson interval method) based on the binomial distribution. In cases where CIs estimates fell outside the 0-100% range, the double arcsine transformation method was used (command: ftt), as it is the preferred method for addressing the problem of variance instability in addition to the CI range problem.

If a source contributed male and female estimates for a country, then we did not combine them to form a mixed estimate as this would result in a single source contributing to overall estimates for a country twice, giving that source undue weight. For countries with estimates for at least two of females, males and mixed samples (without separating females and males within the latter sample), these estimates were weighted by precision (which was determined by the sample size of the study) and the relative proportion of the prison population that were females or males in that country (obtained from the World Prison Brief data). We then found the estimated prevalence ratio between the sexes for that country (if both male and female prevalence estimates were available) and used this to inform the final male and female estimates for these countries. The prevalence ratio was calculated as below:

$$Prevalence ratio for outcome X=\frac{Estimated prevalence of outcome X in females}{Estimated prevalence of outcome X in males}$$

Once we had the prevalence ratio and the combined prevalence estimate, we generated an estimate of male prevalence using this equation:

$$Male prevalence estimate= \frac{N_{c}*\pi_{C}}{N_{M}+P*N_{F}}, with$$

$$N_{c}=Combined prison population$$

$$N_{M}=Male prison population$$

$$N_{F}=Female prison population$$

$$\pi_{C}=Combined prevalence estimate, and$$

$$P=Prevalence ratio estimate (regional or global)$$

The female prevalence estimate was the prevalence ratio estimate multiplied by the above.

If a prevalence ratio was not calculable for a country, we used the average prevalence ratio from that country’s region (weighted by prison population) if the region had two or more prevalence ratio estimates. The exceptions to this were the North American and Australasian regions, in which we generated a regional prevalence ratio estimate if a minimum of one country had a prevalence ratio estimate.

If a regional prevalence estimate was not available, we calculated a global prevalence ratio by the average prevalence ratio of all regions with available prevalence ratio estimates, weighted by regional prison population. If a region had one or fewer estimates of the prevalence ratio (or zero in Australasia or North America), the global prevalence ratio was taken as the regional prevalence ratio for that outcome.

If a country only had an outcome estimate for only of the sexes (e.g., only reported a male-specific estimate without a female-specific estimate), the unavailable country-level sex prevalence estimate was calculated by applying that region’s prevalence ratio to the available country-level sex estimate. The global estimate of prevalence ratio was used if the region did not have a region-specific prevalence ratio estimate.

If a country did not have any sex-specific estimates for an outcome, but had a mixed (females and males) estimate, then we derived country-level estimates for females and males for that outcome. To do so, we applied the region-specific prevalence ratio for females vs. males to the mixed estimate, or the global ratio for females vs. males who are incarcerated if no region-specific ratio was available, using the same equation as outlined above.

Country-level estimates of the number of males and females with the outcome were estimated through multiplying the estimated country-level male and female prevalence by the respective number of males and females incarcerated in that country as reported by the World Prison Brief (<https://www.prisonstudies.org/>). Country-level overall prevalence estimates were then derived by averaging the male and female estimates by the total number of people incarcerated as reported by the World Prison Brief.

**Regional estimates:**

Following the above calculations, the regional-level male- and female-specific prevalence estimates were derived using the same method as in previous global reviews for injecting drug use. The regional estimate for an outcome was based on the average of countries with an estimate available, weighted by country-level incarcerated populations. If a region had fewer than two countries with an estimate available for an outcome, then the global estimate of that outcome was imputed as that region’s prevalence estimate. North America and Australasia were the exceptions, where the global estimate was only imputed for an outcome if there no regional estimates available for that outcome.

Number of people incarcerated with each outcome was calculated through multiplying the estimated regional prevalence by the total number of people incarcerated (overall, male and females) as reported by the World Prison Brief (<https://www.prisonstudies.org/>).

**Global estimate:**

Similar to regional estimates, global estimates were derived using the same method as in previous reviews. For each outcome, the global estimate was based on the average of all regions with at least two countries with prevalence data, or one for North America and Australasia, weighted by regional incarceration populations. The global prevalence estimate for each outcome was then multiplied by the global number of people incarcerated (overall, males and females) to estimate the number of people with each outcome globally

# Appendix 9: Country level prevalence and estimated numbers of injecting drug use, HIV, current HCV infection, HBV and tuberculosis among people, females and males who are incarcerated

## *Table 9.1*: Country-level estimates of the number and rate of incarceration, prevalence of lifetime injecting drug use, HIV and HCV among total people incarcerated

|  | **People who are incarcerated** | | | | **People with lifetime injecting drug use** | | | | **People living with HIV** | | | | **People with current HCV** | | | |
| --- | --- | --- | --- | --- | --- | --- | --- | --- | --- | --- | --- | --- | --- | --- | --- | --- |
| **Country** | **Estimated number^1^** | **Year of estimate^1^** | **Rate per 100,000** | **Trend in incarceration¶** | **% (CI)** | **Estimated no. (CI)** | **Year of estimate** | **Sources** | **% (CI)** | **Estimated no. (CI)** | **Year of estimate** | **Sources** | **% (CI)** | **Estimated no. (CI)** | **Year of estimate** | **Sources** |
| **Eastern Europe** |  |  |  |  |  |  |  |  |  |  |  |  |  |  |  |  |
| Armenia | 2469 | 2024 | 132 | Decreasing | ·· | ·· | ·· |  | 1·3 (0·5, 2·5) | < 500 (<500,<500) | 2004 | 1 | 17·6 (14·5, 20·9) | 500 (500,500) | 2004 | 1 |
| Azerbaijan | 24698 | 2023 | 345 | Stable | 32·0 (28·0, 36·1) | 8000 (7000,9000) | 2014 | 2 | 3·6 (2·5, 5·1) | 1000  (500,1500) | 2008-15 | 2-5 | 33·2 (22·7, 44·7) | 8000 (5500,11000) | 2008-15 | 2-5 |
| Belarus | 32556 | 2018 | 498 | Stable | ·· | ·· | ·· |  | ·· | ·· | ·· |  | ·· | ·· | ·· |  |
| Bosnia & Herzegovina | 2212 | 2024/2023 | 24 | Decreasing | 17·3 (14·5, 20·4) | 500  (500,500) | 2011 | 6 | 0·0 (0·0, 0·3) | < 500 (<500,<500) | 2011 | 6 | 9·8 (6·1, 14·3) | < 500 (<500,500) | 2011-13 | 6,7 |
| Bulgaria | 6378 | 2024 | 146 | Decreasing | 28·5 (24·6, 32·6) | 2000 (1500,2000) | 2009 | 8 | 0·7 (0·2, 1·4) | < 500 (<500,<500) | 2009-20 | 8,9 | 21·4 (18·4, 24·6) | 1500 (1000,1500) | 2009-11 | 8,10 |
| Czechia | 19649 | 2024 | 294 | Stable | ·· | ·· | ·· |  | ·· | ·· | ·· |  | ·· | ·· | ·· |  |
| Estonia | 1688 | 2024 | 201 | Decreasing | ·· | ·· | ·· |  | 15·6 (14·1, 17·1) | 500  (<500,500) | 2012 | 11 | ·· | ·· | ·· |  |
| Georgia | 10457 | 2024 | 432 | Increasing | ·· | ·· | ·· |  | ·· | ·· | ·· |  | 20·9 (20·2, 21·5) | 2000 (2000,2500) | 2015-16 | 12,13 |
| Hungary | 18270 | 2023 | 289 | Increasing | 13·5 (11·8, 15·2) | 2500 (2000,3000) | 1998-2008 | 14,15 | 0·0 (0·0, 0·0) | < 500 (<500,<500) | 2008-14 | 15,16 | 3·9 (2·9, 4·9) | 500 (500,1000) | 2008-19 | 15-18 |
| Latvia | 3271 | 2024 | 278 | Decreasing | 32·3 (29·1, 35·5) | 1000 (1000,1000) | 2022 | 19 | ·· | ·· | ·· |  | ·· | ·· | ·· |  |
| Lithuania | 4551 | 2024 | 254 | Decreasing | 12·0 (3·5, 24·4) | 500  (<500,1000) | 2009-22 | 20,21 | ·· | ·· | ·· |  | ·· | ·· | ·· |  |
| Republic of Moldova | 5695 | 2024 | 279 | Decreasing | ·· | ·· | ·· |  | ·· | ·· | ·· |  | ·· | ·· | ·· |  |
| Poland | 70316 | 2024 | 279 | Stable | ·· | ·· | ·· |  | ·· | ·· | ·· |  | ·· | ·· | ·· |  |
| Romania | 24534 | 2024 | 195 | Stable | ·· | ·· | ·· |  | 56·7 (46·7, 66·4) | 14000 (11500,16500) | 2023 | 22 | 20·9 (3·5, 47·3) | 5000 (1000,11500) | 2010-23 | 22-24 |
| Russian Federation | 433006 | 2023 | 447 | Decreasing | ·· | ·· | ·· |  | ·· | ·· | ·· |  | ·· | ·· | ·· |  |
| Slovakia | 8585 | 2024 | 236 | Decreasing | ·· | ·· | ·· |  | ·· | ·· | ·· |  | ·· | ·· | ·· |  |
| Ukraine | 44024 | 2024 | 150 | Decreasing | 43·7 (38·9, 48·5) | 19000 (17000,21500) | 2011-15 | 25-27 | 12·6 (10·8, 14·5) | 5500 (4500,6500) | 2011 | 25,26 | 73·8 (50·5, 99·8) | 32500 (22000,44000) | 2011-21 | 25,28 |
| **Western Europe** |  |  |  |  |  |  |  |  |  |  |  |  |  |  |  |  |
| Albania | 4653 | 2024 | 242 | Decreasing | ·· | ·· | ·· |  | ·· | ·· | ·· |  | ·· | ·· | ·· |  |
| Andorra | 51 | 2024 | 89 | Decreasing | ·· | ·· | ·· |  | ·· | ·· | ·· |  | ·· | ·· | ·· |  |
| Austria | 9288 | 2024 | 157 | Stable | 4·3 (0·5, 10·5) | 500  (<500,1000) | 2016 | 29 | 0·0 (0·0, 2·6) | < 500 (<500,<500) | 2016 | 29 | 1·5 (0·0, 6·4) | < 500 (<500,500) | 2016 | 29 |
| Belgium | 12575 | 2024 | 169 | Increasing | 20·4 (17·3, 23·6) | 2500 (2000,3000) | 2020 | 30 | 1·1 (0·1, 3·3) | < 500  (<500,500) | 2006-20 | 30,31 | 7·6 (3·7, 12·7) | 1000  (500,1500) | 2006-22 | 30-32 |
| Croatia | 4445 | 2023 | 171 | Increasing | 24·6 (22·9, 26·3) | 1000 (1000,1000) | 2007-09 | 33,34 | 0·1 (0·0, 0·3) | < 500 (<500,<500) | 2006-09 | 34,35 | 10·4 (9·3, 11·6) | 500  (500,500) | 2007-09 | 33,34 |
| Denmark | 4083 | 2024 | 110 | Increasing | 43·3 (37·9, 48·7) | 2000 (1500,2000) | 1997 | 36 | 0·0 (0·0, 0·6) | < 500 (<500,<500) | 1997 | 36 | 28·9 (24·1, 34·0) | 1000 (1000,1500) | 1997-2017 | 36,37 |
| England and Wales | 85867 | 2024 | 223 | Stable | 23·7 (21·5, 26·1) | 20500 (18500,22500) | 1995-2018 | 38-44 | 0·2 (0·1, 0·4) | < 500  (<500,500) | 1998-2020 | 38,42,44-46 | 6·3 (3·5, 10·0) | 5500 (3000,8500) | 1998-2024 | 38,42,44-61 |
| Finland | 2912 | 2023 | 85 | Stable | 54·5 (50·5, 58·5) | 1500 (1500,1500) | 2007-23 | 62,63 | 0·9 (0·2, 1·9) | < 500 (<500,<500) | 2007-23 | 63,64 | 32·3 (28·3, 36·4) | 1000 (1000,1000) | 2007-23 | 63,64 |
| France | 79631 | 2024 | 201 | Increasing | 19·0 (5·7, 37·3) | 15000 (4500,29500) | 1997-2019 | 65-72 | 2·7 (0·8, 5·6) | 2000  (500,4500) | 1997-2019 | 65,70,73-78 | 3·5 (1·2, 6·7) | 3000 (1000,5500) | 1997-2020 | 65,68,71,73-82 |
| Germany | 57955 | 2023 | 109 | Stable | 29·3 (21·5, 37·9) | 17000 (12500,22000) | 1997-2007 | 70,83 | 2·7 (0·7, 5·7) | 1500  (500,3500) | 1997-2007 | 70,83,84 | 5·5 (4·3, 6·9) | 3000 (2500,4000) | 2002-07 | 83,84 |
| Greece | 10242 | 2024 | 155 | Stable | 37·1 (30·9, 43·6) | 4000 (3000,4500) | 1996-2018 | 85-88 | 0·0 (0·0, 2·3) | < 500 (<500,<500) | 2002 | 85 | 11·2 (8·7, 14·0) | 1000 (1000,1500) | 2018 | 89 |
| Greenland | 154 | 2023 | 393 | Increasing | NK | NK | ·· |  | ·· | ·· | ·· |  | ·· | ·· | ·· |  |
| Iceland | 140 | 2024 | 57 | Decreasing | ·· | ·· | ·· |  | ·· | ·· | ·· |  | 3·1 (0·1, 9·2) | < 500 (<500,<500) | 2020 | 90 |
| Ireland | 5074 | 2024 | 156 | Increasing | 30·4 (24·9, 36·1) | 1500 (1500,2000) | 1998-2017 | 91-94 | 1·2 (0·5, 2·1) | < 500 (<500,<500) | 1998-2017 | 91-93,95-97 | 10·8 (7·0, 15·4) | 500  (500,1000) | 1998-2017 | 23,91-93,97,98 |
| Italy | 62110 | 2024 | 166 | Increasing | 27·7 (19·2, 38·0) | 17000 (12000,23500) | 1997-2021 | 70,99-106 | 3·1 (1·8, 4·9) | 2000 (1000,3000) | 1997-2023 | 70,99-101,107-113 | 11·1 (6·9, 16·2) | 7000 (4500,10000) | 2002-23 | 99-102,104-108,111-120 |
| Liechtenstein | 14 | 2024 | 54 | Increasing | ·· | ·· | ·· |  | ·· | ·· | ·· |  | ·· | ·· | ·· |  |
| Luxembourg | 600 | 2024 | 135 | Decreasing | 22·8 (17·1, 29·0) | < 500 (<500,<500) | 2022 | 121 | ·· | ·· | ·· |  | ·· | ·· | ·· |  |
| Malta | 671 | 2024 | 187 | Stable | ·· | ·· | ·· |  | 1·3 (0·4, 2·6) | < 500 (<500,<500) | 2019 | 122 | 18·3 (15·0, 21·9) | < 500 (<500,<500) | 2019 | 122 |
| Monaco | 31 | 2024 | 165 | Increasing | ·· | ·· | ·· |  | ·· | ·· | ·· |  | ·· | ·· | ·· |  |
| Montenegro | 1046 | 2024 | 254 | Stable | 16·4 (13·3, 19·8) | < 500 (<500,<500) | 2012-21 | 123 | 0·0 (0·0, 0·7) | < 500 (<500,<500) | 2012-21 | 123 | 16·0 (12·9, 19·3) | < 500 (<500,<500) | 2012-21 | 123 |
| Netherlands | 11537 | 2023 | 102 | Increasing | 8·6 (5·8, 12·0) | 1000 (500,1500) | 1997-2010 | 70,124 | 0·4 (0·0, 1·6) | < 500 (<500,<500) | 1997-2010 | 70,124 | 4·9 (2·4, 8·2) | 500 (500,1000) | 2010 | 124 |
| North Macedonia | 2555 | 2024 | 176 | Decreasing | ·· | ·· | ·· |  | 0·0 (0·0, 0·9) | < 500 (<500,<500) | 2011 | 125 | 15·0 (10·4, 20·3) | 500 (500,500) | 2011 | 125 |
| Northern Ireland | 1911 | 2024 | 159 | Increasing | 12·7 (8·9, 17·3) | < 500  (<500,500) | 2005 | 126 | 0·0 (0·0, 0·3) | < 500 (<500,<500) | 2005 | 126 | 0·8 (0·2, 1·6) | < 500 (<500,<500) | 2005 | 126 |
| Norway | 3052 | 2024 | 87 | Decreasing | 51·6 (39·1, 64·0) | 1500 (1000,2000) | 2014-17 | 127,128 | ·· | ·· | ·· |  | 24·2 (14·2, 35·7) | 500 (500,1000) | 2017 | 128 |
| Portugal | 12379 | 2024 | 188 | Decreasing | 14·7 (12·2, 17·5) | 2000 (1500,2000) | 2002-05 | 129-131 | 6·1 (4·5, 8·0) | 1000  (500,1000) | 2002-23 | 129-133 | 9·6 (7·7, 11·9) | 1000 (1000,1500) | 2002-23 | 129-132,134-137 |
| San Marino | 1 | 2024 | 4 | Decreasing | ·· | ·· | ·· |  | ·· | ·· | ·· |  | ·· | ·· | ·· |  |
| Scotland | 8253 | 2024 | 235 | Stable | 19·9 (13·1, 27·6) | 1500 (1000,2500) | 1997-2018 | 70,138-140 | 0·7 (0·0, 2·2) | < 500 (<500,<500) | 1997-2016 | 70,141 | 3·4 (2·5, 4·3) | 500 (<500,500) | 1998-2011 | 139,140,142 |
| Serbia | 10787 | 2023 | 227 | Increasing | ·· | ·· | ·· |  | ·· | ·· | ·· |  | ·· | ·· | ·· |  |
| Slovenia | 1798 | 2024 | 133 | Increasing | ·· | ·· | ·· |  | ·· | ·· | ·· |  | ·· | ·· | ·· |  |
| Spain | 56698 | 2023 | 181 | Decreasing | 25·4 (19·4, 32·0) | 14500 (11000,18000) | 1999-2022 | 143-150 | 5·4 (2·4, 9·5) | 3000 (1500,5500) | 1999-2023 | 145-149,151-160 | 12·0 (7·6, 18·3) | 7000 (4500,10500) | 1999-2021 | 145-147,149,151,155,157-159,161-166 |
| Sweden | 10175 | 2024 | 156 | Increasing | 25·1 (17·5, 33·7) | 2500 (2000,3500) | 1997 | 70 | 0·4 (0·0, 1·6) | < 500 (<500,<500) | 1997-2017 | 70,167 | 11·3 (8·0, 15·1) | 1000 (1000,1500) | 2017 | 167 |
| Switzerland | 6881 | 2024 | 120 | Stable | 4·5 (2·9, 6·5) | 500 (<500,500) | 2007-13 | 168-170 | 2·2 (1·4, 3·1) | < 500 (<500,<500) | 2011 | 169,171 | 3·7 (2·7, 4·8) | 500 (<500,500) | 2011-13 | 168,169,171 |
| **East and South East Asia** |  |  |  |  |  |  |  |  |  |  |  |  |  |  |  |  |
| Brunei Darussalam | 636 | 2022 | 199 | Stable | ·· | ·· | ·· |  | ·· | ·· | ·· |  | ·· | ·· | ·· |  |
| Cambodia | 45122 | 2024 | 417 | Increasing | ·· | ·· | ·· |  | ·· | ·· | ·· |  | ·· | ·· | ·· |  |
| China | 1690000 | 2018 | 13 | Decreasing | ·· | ·· | ·· |  | ·· | ·· | ·· |  | ·· | ·· | ·· |  |
| Hong Kong† | 9079 | 2023 | 178 | Increasing | ·· | ·· | ·· |  | ·· | ·· | ·· |  | ·· | ·· | ·· |  |
| Indonesia | 274060 | 2024 | 148 | Increasing | 5·2 (4·2, 6·2) | 14000 (11500,17000) | 2010-21 | 172-175 | 1·1 (0·3, 2·2) | 3000 (1000,6000) | 2010-21 | 172,174-178 | 22·4 (6·3, 46·6) | 61500 (17500,128000) | 2016-19 | 176,178,179 |
| Japan | 40881 | 2023 | 56 | Decreasing | ·· | ·· | ·· |  | ·· | ·· | ·· |  | ·· | ·· | ·· |  |
| Lao People's Democratic Republic | 11885 | 2018 | 262 | Increasing | ·· | ·· | ·· |  | ·· | ·· | ·· |  | ·· | ·· | ·· |  |
| Malaysia | 87419 | 2024 | 373 | Increasing | ·· | ·· | ·· |  | ·· | ·· | ·· |  | 16·2 (15·6, 16·7) | 14000 (13500,14500) | 2023 | 180 |
| Mongolia | 5700 | 2023 | 270 | Stable | ·· | ·· | ·· |  | ·· | ·· | ·· |  | ·· | ·· | ·· |  |
| Myanmar | 100324 | 2020 | 274 | Increasing | ·· | ·· | ·· |  | 5·5 (4·9, 6·2) | 5500 (5000,6000) | 2018 | 181 | ·· | ·· | ·· |  |
| Democratic People's Republic of Korea* | 100000 | 2016 | 552 | ·· | NK | NK | ·· |  | ·· | ·· | ·· |  | ·· | ·· | ·· |  |
| Philippines | 171247 | 2024 | 235 | Stable | ·· | ·· | ·· |  | 0·0 (0·0, 0·6) | < 500 (<500,1000) | 1997 | 182 | ·· | ·· | ·· |  |
| Singapore | 9536 | 2022 | 217 | Decreasing | ·· | ·· | ·· |  | ·· | ·· | ·· |  | ·· | ·· | ·· |  |
| Republic of Korea | 52940 | 2022 | 143 | Stable | ·· | ·· | ·· |  | 0·2 (0·1, 0·2) | < 500 (<500,<500) | 2019 | 183 | ·· | ·· | ·· |  |
| Taiwan† | 58889 | 2024 | 346 | Stable | 42·3 (41·2, 43·3) | 25000 (24500,25500) | 1994-2019 | 184-186 | 0·0 (0·0, 2·4) | < 500 (<500,1500) | 2019 | 186 | 20·3 (18·5, 22·1) | 12000 (11000,13000) | 2019 | 186,187 |
| Thailand | 274277 | 2023 | 550 | Decreasing | 6·8 (5·3, 8·4) | 18500 (14500,23000) | 2019 | 188 | 4·0 (2·7, 5·5) | 11000 (7500,15000) | 2015-19 | 189 | 3·7 (2·7, 4·9) | 10000 (7500,13500) | 2019 | 188 |
| Timor-Leste | 763 | 2021 | 97 | Increasing | ·· | ·· | ·· |  | ·· | ·· | ·· |  | ·· | ·· | ·· |  |
| Viet Nam | 133986 | 2022 | 200 | Stable | ·· | ·· | ·· |  | ·· | ·· | ·· |  | ·· | ·· | ·· |  |
| **South Asia** |  |  |  |  |  |  |  |  |  |  |  |  |  |  |  |  |
| Afghanistan | 19000 | 2024 | 87 | Decreasing | 1·9 (1·0, 3·2) | 500  (<500,500) | 2010 | 190 | 0·8 (0·4, 1·4) | < 500 (<500,500) | 2010-12 | 190,191 | 2·2 (1·2, 3·5) | 500 (<500,500) | 2010-12 | 190,191 |
| Bangladesh | 53831 | 2024 | 47 | Decreasing | ·· | ·· | ·· |  | ·· | ·· | ·· |  | ·· | ·· | ·· |  |
| Bhutan | 1119 | 2014 | 227 | Increasing | ·· | ·· | ·· |  | ·· | ·· | ·· |  | ·· | ·· | ·· |  |
| India | 573220 | 2022 | 60 | Increasing | 3·0 (2·1, 4·1) | 17500 (12000,23500) | 2015-21 | 192-196 | 0·9 (0·6, 1·3) | 5000 (3000,7500) | 2010-21 | 192-195,197-199 | 8·9 (8·4, 9·6) | 51000 (48000,55000) | 2015-22 | 196,199-201 |
| Iran (Islamic Republic of) | 189000 | 2020 | 314 | Decreasing | 15·5 (13·2, 18·1) | 29500 (25000,34000) | 2008-97 | 202-223 | 2·0 (1·2, 3·0) | 4000 (2000,5500) | 2003-17 | 218,219,221,222,224-229 | 8·4 (4·3, 13·9) | 16000 (8000,26500) | 2003-22 | 206,207,211,215,216,218,220,225,226,229-233 |
| Maldives | 1700 | 2020 | 448 | Decreasing | ·· | ·· | ·· |  | ·· | ·· | ·· |  | ·· | ·· | ·· |  |
| Nepal | 27550 | 2022 | 142 | Increasing | 10·8 (8·7, 13·1) | 3000 (2500,3500) | 2015 | 234,235 | ·· | ·· | ·· |  | ·· | ·· | ·· |  |
| Pakistan | 108643 | 2024 | 80 | Increasing | 19·5 (5·7, 38·7) | 21000 (6000,42000) | 2008-18 | 236-239 | 1·9 (0·8, 3·5) | 2000 (1000,4000) | 2006-18 | 237,238,240-244 | 8·3 (5·1, 12·1) | 9000 (5500,13000) | 2005-17 | 236,237,239,241,244-247 |
| Sri Lanka | 29686 | 2024 | 208 | Increasing | 4·2 (2·4, 6·6) | 1500 (500,2000) | 2013 | 248 | ·· | ·· | ·· |  | 0·6 (0·0, 1·9) | < 500 (<500,500) | 2013 | 248 |
| **Central Asia** |  |  |  |  |  |  |  |  |  |  |  |  |  |  |  |  |
| Kazakhstan | 35228 | 2022 | 294 | Decreasing | ·· | ·· | ·· |  | ·· | ·· | ·· |  | ·· | ·· | ·· |  |
| Kyrgyzstan | 7728 | 2023 | 194 | Decreasing | 48·2 (26·4, 70·3) | 3500 (2000,5500) | 2006-14 | 249,250 | 8·4 (5·8, 11·5) | 500 (500,1000) | 2014 | 249 | 31·8 (27·1, 36·6) | 2500 (2000,3000) | 2014 | 249 |
| Tajikistan | 14000 | 2022 | 238 | Increasing | 4·1 (3·1, 5·2) | 500 (500,500) | 2010 | 251 | 0·3 (0·2, 0·5) | < 500 (<500,<500) | 2019 | 252 | ·· | ·· | ·· |  |
| Turkmenistan | 35000 | 2021 | 864 | Increasing | ·· | ·· | ·· |  | ·· | ·· | ·· |  | ·· | ·· | ·· |  |
| Uzbekistan | 29000 | 2022 | 131 | Decreasing | ·· | ·· | ·· |  | ·· | ·· | ·· |  | ·· | ·· | ·· |  |
| **Caribbean** |  |  |  |  |  |  |  |  |  |  |  |  |  |  |  |  |
| Antigua & Barbuda | 400 | 2023 | 604 | Stable | NK | NK | ·· |  | 2·9 (0·4, 7·1) | < 500 (<500,<500) | 2005 | 253 | ·· | ·· | ·· |  |
| Bahamas | 1912 | 2021 | 653 | Increasing | ·· | ·· | ·· |  | ·· | ·· | ·· |  | ·· | ·· | ·· |  |
| Barbados | 692 | 2023 | 367 | Decreasing | ·· | ·· | ·· |  | ·· | ·· | ·· |  | ·· | ·· | ·· |  |
| Bermuda | 124 | 2021 | 295 | Decreasing | ·· | ·· | ·· |  | ·· | ·· | ·· |  | ·· | ·· | ·· |  |
| Cuba | 90000 | 2020 | 1161 | Increasing | ·· | ·· | ·· |  | ·· | ·· | ·· |  | ·· | ·· | ·· |  |
| Dominica | 260 | 2024 | 507 | Increasing | NK | NK | ·· |  | 2·6 (0·7, 5·4) | < 500 (<500,<500) | 2005 | 253 | ·· | ·· | ·· |  |
| Dominican Republic | 25987 | 2024 | 357 | Stable | ·· | ·· | ·· |  | ·· | ·· | ·· |  | ·· | ·· | ·· |  |
| Grenada | 385 | 2023 | 468 | Decreasing | NK | NK | ·· |  | 2·2 (0·3, 5·4) | < 500 (<500,<500) | 2005 | 253 | ·· | ·· | ·· |  |
| Haiti | 7523 | 2024 | 104 | Decreasing | ·· | ·· | ·· |  | ·· | ·· | ·· |  | ·· | ·· | ·· |  |
| Jamaica | 3559 | 2022 | 174 | Decreasing | ·· | ·· | ·· |  | 3·4 (2·3, 4·7) | < 500 (<500,<500) | 2008 | 254 | ·· | ·· | ·· |  |
| Commonwealth of Puerto Rico | 5798 | 2022 | 278 | Decreasing | 31·6 (29·0, 34·3) | 2000 (1500,2000) | 2005 | 255 | ·· | ·· | ·· |  | ·· | ·· | ·· |  |
| Saint Kitts & Nevis | 160 | 2022 | 476 | Decreasing | NK | NK | ·· |  | 2·3 (0·5, 5·1) | < 500 (<500,<500) | 2005 | 253 | ·· | ·· | ·· |  |
| Saint Lucia | 572 | 2023 | 438 | Decreasing | NK | NK | ·· |  | 2·0 (0·7, 3·7) | < 500 (<500,<500) | 2005 | 253 | ·· | ·· | ·· |  |
| Saint Vincent & the Grenadines | 404 | 2024 | 577 | Stable | NK | NK | ·· |  | 4·0 (2·2, 6·3) | < 500 (<500,<500) | 2005 | 253 | ·· | ·· | ·· |  |
| Trinidad & Tobago | 3802 | 2021 | 358 | Stable | NK | NK | ·· |  | ·· | ·· | ·· |  | ·· | ·· | ·· |  |
| **Latin America** |  |  |  |  |  |  |  |  |  |  |  |  |  |  |  |  |
| Argentina | 125041 | 2023 | 426 | Increasing | ·· | ·· | ·· |  | 2·2 (1·4, 3·5) | 2500 (1500,4500) | 2016-20 | 256-259 | 2·1 (1·3, 3·1) | 2500 (1500,4000) | 2016-20 | 256-259 |
| Belize | 1339 | 2024 | 501 | Decreasing | NK | NK | ·· |  | 4·1 (2·7, 5·8) | < 500 (<500,<500) | 2005 | 260 | ·· | ·· | ·· |  |
| Bolivia (Plurinational State of) | 31105 | 2024 | 403 | Increasing | ·· | ·· | ·· |  | 0·2 (0·0, 0·6) | < 500 (<500,<500) | 2002-13 | 261,262 | ·· | ·· | ·· |  |
| Brazil | 888791 | 2024 | 593 | Increasing | 7·0 (2·9, 12·6) | 62000 (26000,112000) | 1994-2020 | 263-280 | 3·2 (1·8, 5·1) | 28500 (15500,45000) | 1994-2022 | 263,266,269-272,275,279-304 | 4·9 (2·2, 8·6) | 43500 (19500,76500) | 1994-2022 | 264-268,270,272,273,275,277,278,282,284,286-288,294,296,298,303,305-312 |
| Chile | 59037 | 2024 | 440 | Increasing | ·· | ·· | ·· |  | ·· | ·· | ·· |  | ·· | ·· | ·· |  |
| Colombia | 104346 | 2024 | 291 | Decreasing | 0·7 (0·1, 1·7) | 500 (<500,2000) | 2013-19 | 313,314 | 1·2 (0·3, 2·4) | 1000  (500,2500) | 2019 | 314 | ·· | ·· | ·· |  |
| Costa Rica | 17829 | 2022 | 502 | Decreasing | ·· | ·· | ·· |  | ·· | ·· | ·· |  | ·· | ·· | ·· |  |
| Ecuador | 33669 | 2024 | 286 | Stable | ·· | ·· | ·· |  | ·· | ·· | ·· |  | ·· | ·· | ·· |  |
| El Salvador | 109519 | 2024 | 2625 | Increasing | ·· | ·· | ·· |  | ·· | ·· | ·· |  | ·· | ·· | ·· |  |
| Guatemala | 23361 | 2023 | 213 | Stable | 4·9 (3·3, 6·8) | 1000 (1000,1500) | 2012 | 315 | 0·8 (0·2, 1·7) | < 500  (<500,500) | 2012 | 315 | ·· | ·· | ·· |  |
| Guyana | 2300 | 2024 | 439 | Stable | ·· | ·· | ·· |  | ·· | ·· | ·· |  | ·· | ·· | ·· |  |
| Honduras | 19481 | 2023 | 291 | Stable | ·· | ·· | ·· |  | ·· | ·· | ·· |  | ·· | ·· | ·· |  |
| Mexico | 234514 | 2024 | 277 | Decreasing | 6·5 (5·5, 7·5) | 15000 (13000,17500) | 2002-12 | 316-319 | 0·6 (0·5, 0·8) | 1500  (1000,2000) | 2002-12 | 316,317,320,321 | 2.3 (2.0, 2.7) | 5500 (4500,6500) | 2002-24 | 316,317,319-324 |
| Nicaragua | 20918 | 2018 | 496 | Increasing | ·· | ·· | ·· |  | ·· | ·· | ·· |  | ·· | ·· | ·· |  |
| Panama | 23798 | 2024 | 841 | Increasing | ·· | ·· | ·· |  | ·· | ·· | ·· |  | ·· | ·· | ·· |  |
| Paraguay | 17712 | 2023 | 408 | Increasing | ·· | ·· | ·· |  | ·· | ·· | ·· |  | ·· | ·· | ·· |  |
| Peru | 97605 | 2024 | 443 | Increasing | ·· | ·· | ·· |  | 1·2 (0·5, 2·3) | 1000  (500,2000) | 2011-15 | 325,326 | ·· | ·· | ·· |  |
| Suriname | 1000 | 2014 | 270 | Stable | ·· | ·· | ·· |  | ·· | ·· | ·· |  | ·· | ·· | ·· |  |
| Uruguay | 15767 | 2024 | 707 | Increasing | ·· | ·· | ·· |  | 6·8 (4·1, 10·1) | 1000  (500,1500) | 2005 | 327 | ·· | ·· | ·· |  |
| Venezuela (Bolivarian Republic of) | 67200 | 2022 | 375 | Increasing | 5·2 (2·4, 8·8) | 3500 (1500,6000) | 2007 | 328 | 0·6 (0·4, 0·8) | 500  (500,500) | 2001-22 | 329-331 | 1.4 (0.2, 3.8) | 1000 (<500,2500) | 2007-18 | 328,329 |
| **North America** |  |  |  |  |  |  |  |  |  |  |  |  |  |  |  |  |
| Canada | 34986 | 2023 | 139 | Decreasing | 29·7 (26·8, 32·7) | 10500 (9500,11500) | 1997-2019 | 332-340 | 1·7 (1·2, 2·3) | 500  (500,1000) | 1998-2021 | 334,336,341-343 | 13.6 (11.1, 16.4) | 4500 (4000,5500) | 1998-2022 | 332,334,336,340,341,343-346 |
| United States of America | 1808100 | 2022 | 522 | Decreasing | 13·1 (10·0, 16·4) | 236000 (181000,297500) | 1993-2018 | 347-379 | 1·6 (1·3, 2·0) | 29500 (24000,35500) | 1991-2023 | 350-352,355,356,361-364,368,373-375,377,379-425 | 15.1 (13.1, 17.2) | 272500 (237000,310500) | 1994-2024 | 350,352,358,359,369,379,381,391,394,402,408,410,412,415,421,425-444 |
| **Pacific Island States & Terr·** |  |  |  |  |  |  |  |  |  |  |  |  |  |  |  |  |
| American Samoa | 301 | 2022 | 1018 | Increasing | ·· | ·· | ·· |  | ·· | ·· | ·· |  | ·· | ·· | ·· |  |
| Micronesia (Federated States of) | 132 | 2014 | 40 | Increasing | ·· | ·· | ·· |  | ·· | ·· | ·· |  | ·· | ·· | ·· |  |
| Fiji | 2276 | 2024 | 377 | Increasing | ·· | ·· | ·· |  | 1·0 (0·0, 3·1) | < 500 (<500,<500) | 2013 | 445 | ·· | ·· | ·· |  |
| French Polynesia | 575 | 2024 | 275 | Increasing | ·· | ·· | ·· |  | ·· | ·· | ·· |  | ·· | ·· | ·· |  |
| Guam | 896 | 2024 | 844 | Increasing | ·· | ·· | ·· |  | ·· | ·· | ·· |  | ·· | ·· | ·· |  |
| Kiribati | 129 | 2016 | 179 | Increasing | ·· | ·· | ·· |  | ·· | ·· | ·· |  | ·· | ·· | ·· |  |
| Marshall Islands | 35 | 2014 | 117 | Decreasing | ·· | ·· | ·· |  | ·· | ·· | ·· |  | ·· | ·· | ·· |  |
| Nauru | 38 | 2023 | 516 | Increasing | NK | NK | ·· |  | ·· | ·· | ·· |  | ·· | ·· | ·· |  |
| New Caledonia | 609 | 2024 | 317 | Increasing | ·· | ·· | ·· |  | ·· | ·· | ·· |  | ·· | ·· | ·· |  |
| Northern Mariana Islands | 170 | 2022 | 501 | Stable | ·· | ·· | ·· |  | ·· | ·· | ·· |  | ·· | ·· | ·· |  |
| Palau | 66 | 2023 | 527 | Stable | ·· | ·· | ·· |  | ·· | ·· | ·· |  | ·· | ·· | ·· |  |
| Papua New Guinea | 5373 | 2023 | 87 | Stable | ·· | ·· | ·· |  | ·· | ·· | ·· |  | ·· | ·· | ·· |  |
| Samoa | 358 | 2019 | 296 | Decreasing | ·· | ·· | ·· |  | ·· | ·· | ·· |  | ·· | ·· | ·· |  |
| Solomon Islands | 500 | 2019 | 131 | Increasing | ·· | ·· | ·· |  | ·· | ·· | ·· |  | ·· | ·· | ·· |  |
| Tonga | 557 | 2022 | 888 | Increasing | ·· | ·· | ·· |  | ·· | ·· | ·· |  | ·· | ·· | ·· |  |
| Tuvalu | 11 | 2014 | 161 | Increasing | ·· | ·· | ·· |  | ·· | ·· | ·· |  | ·· | ·· | ·· |  |
| Vanuatu | 195 | 2021 | 108 | Increasing | ·· | ·· | ·· |  | ·· | ·· | ·· |  | ·· | ·· | ·· |  |
| **Australasia** |  |  |  |  |  |  |  |  |  |  |  |  |  |  |  |  |
| Australia‡§ | 44051 | 2024 | 261 | Increasing | 51·0 (47·0, 54·9) | 22500 (20500,24000) | 1996-2023 | 446-463 | 0·8 (0·4, 1·7) | 500  (<500,500) | 2023 | 447 | 8.0 (6.4, 9.9) | 3500 (3000,4500) | 2023 | 447 |
| New Zealand | 9924 | 2024 | 297 | Stable | ·· | ·· | ·· |  | 0·0 (0·0, 0·1) | < 500 (<500,<500) | 2022 | 464 | ·· | ·· | ·· |  |
| **Sub Saharan Africa** |  |  |  |  |  |  |  |  |  |  |  |  |  |  |  |  |
| Angola | 24068 | 2024 | 134 | Decreasing | ·· | ·· | ·· |  | ·· | ·· | ·· |  | ·· | ·· | ·· |  |
| Benin | 19563 | 2024 | 277 | Increasing | 0·6 (0·1, 1·5) | < 500 (<500,500) | 2015 | 465 | 1·4 (0·5, 2·7) | 500 (<500,500) | 2015 | 465 | ·· | ·· | ·· |  |
| Botswana | 3971 | 2022 | 242 | Decreasing | ·· | ·· | ·· |  | ·· | ·· | ·· |  | ·· | ·· | ·· |  |
| Burkina Faso | 8800 | 2022 | 75 | Stable | 3·3 (2·4, 4·4) | 500 (<500,500) | 2017 | 466 | 2·3 (1·6, 3·2) | < 500 (<500,500) | 2009-17 | 466-468 | 5.7 (4.7, 6.8) | 500 (500,500) | 2012 | 469 |
| Burundi | 13824 | 2024 | 215 | Increasing | ·· | ·· | ·· |  | ·· | ·· | ·· |  | ·· | ·· | ·· |  |
| Cameroon | 34419 | 2024 | 231 | Stable | 2·8 (1·8, 3·9) | 1000 (500,1500) | 2018 | 470 | 11·3 (9·5, 13·2) | 4000 (3500,4500) | 2004 | 471 | ·· | ·· | ·· |  |
| Cabo Verde | 2700 | 2024 | 676 | Increasing | ·· | ·· | ·· |  | ·· | ·· | ·· |  | ·· | ·· | ·· |  |
| Central African Republic | 2678 | 2023 | 100 | Increasing | ·· | ·· | ·· |  | ·· | ·· | ·· |  | ·· | ·· | ·· |  |
| Chad | 9589 | 2022 | 111 | Stable | ·· | ·· | ·· |  | ·· | ·· | ·· |  | ·· | ·· | ·· |  |
| Comoros | 422 | 2023 | 89 | Increasing | ·· | ·· | ·· |  | ·· | ·· | ·· |  | ·· | ·· | ·· |  |
| Côte d'Ivoire | 27149 | 2024 | 177 | Increasing | ·· | ·· | ·· |  | 4·7 (4·0, 5·4) | 1500 (1000,1500) | 2010-15 | 472,473 | ·· | ·· | ·· |  |
| Democratic Republic of the Congo | 44536 | 2022 | 92 | Increasing | ·· | ·· | ·· |  | 6·1 (4·9, 7·4) | 2500 (2000,3500) | 2009-15 | 474,475 | ·· | ·· | ·· |  |
| Djibouti | 750 | 2022 | 105 | Increasing | ·· | ·· | ·· |  | ·· | ·· | ·· |  | ·· | ·· | ·· |  |
| Equatorial Guinea | 500 | 2015 | 64 | Stable | NK | NK | ·· |  | ·· | ·· | ·· |  | ·· | ·· | ·· |  |
| Eritrea** | ·· | N/A | ·· | ·· | NK | NK | ·· |  | ·· | ·· | ·· |  | ·· | ·· | ·· |  |
| Eswatini | 3405 | 2022 | 468 | Decreasing | ·· | ·· | ·· |  | 33.4 (28.9, 38.1) | 1000 (1000,1500) | 2010 | 476 | ·· | ·· | ·· |  |
| Ethiopia | 110000 | 2020 | 166 | Decreasing | 5·6 (3·9, 7·6) | 6000 (4500,8500) | 2020-22 | 477,478 | 3·1 (2·6, 3·5) | 3500 (3000,4000) | 2016 | 479-481 | 1.8 (0.1, 5.0) | 2000 (<500,5500) | 2016-22 | 477-479,482 |
| Gabon | 5501 | 2024 | 394 | Increasing | ·· | ·· | ·· |  | ·· | ·· | ·· |  | ·· | ·· | ·· |  |
| Gambia | 543 | 2021 | 38 | Decreasing | ·· | ·· | ·· |  | ·· | ·· | ·· |  | ·· | ·· | ·· |  |
| Ghana | 14262 | 2024 | 73 | Decreasing | 17·1 (16·2, 18·0) | 2500 (2500,2500) | 2005-13 | 483-485 | 7·3 (5·0, 10·2) | 1000 (500,1500) | 2005-13 | 483-486 | 14.4 (12.2, 16.9) | 2000 (1500,2500) | 2005 | 486-488 |
| Guinea | 5549 | 2024 | 75 | Increasing | ·· | ·· | ·· |  | ·· | ·· | ·· |  | ·· | ·· | ·· |  |
| Guinea-Bissau | 596 | 2017 | 58 | Increasing | ·· | ·· | ·· |  | ·· | ·· | ·· |  | ·· | ·· | ·· |  |
| Kenya | 60000 | 2023 | 193 | Stable | ·· | ·· | ·· |  | 9·6 (7·8, 11·6) | 6000 (4500,7000) | 2010-23 | 489,490 | ·· | ·· | ·· |  |
| Lesotho | 2216 | 2019 | 162 | Stable | 3·3 (0·7, 7·3) | < 500 (<500,<500) | 2012 | 491 | ·· | ·· | ·· |  | ·· | ·· | ·· |  |
| Liberia | 3000 | 2023 | 104 | Increasing | 33·0 (24·1, 42·6) | 1000 (500,1500) | 2021 | 492 | 5·6 (3·9, 7·6) | < 500 (<500,<500) | 2017 | 493 | 0.8 (0.2, 1.8) | < 500 (<500,<500) | 2017 | 493 |
| Madagascar | 30530 | 2023 | 184 | Increasing | ·· | ·· | ·· |  | 0·2 (0·0, 0·7) | < 500 (<500,<500) | 2020-21 | 494,495 | ·· | ·· | ·· |  |
| Malawi | 16536 | 2024 | 154 | Stable | ·· | ·· | ·· |  | 26·2 (17·6, 36·2) | 4500 (3000,6000) | 1997-2022 | 496-500 | 0.0 (0.0, 1.3) | < 500 (<500,<500) | 2005 | 497 |
| Mali | 8670 | 2022 | 79 | Increasing | ·· | ·· | ·· |  | ·· | ·· | ·· |  | ·· | ·· | ·· |  |
| Mauritania | 2826 | 2022 | 112 | Increasing | NK | NK | ·· |  | ·· | ·· | ·· |  | ·· | ·· | ·· |  |
| Mauritius | 2755 | 2024 | 298 | Increasing | ·· | ·· | ·· |  | ·· | ·· | ·· |  | ·· | ·· | ·· |  |
| Mozambique | 22000 | 2024 | 128 | Increasing | ·· | ·· | ·· |  | 12·0 (11·2, 12·9) | 2500 (2500,3000) | 2023 | 501 | ·· | ·· | ·· |  |
| Namibia | 8900 | 2021/2022 | 589 | Increasing | ·· | ·· | ·· |  | ·· | ·· | ·· |  | ·· | ·· | ·· |  |
| Niger | 13005 | 2023 | 106 | Stable | ·· | ·· | ·· |  | ·· | ·· | ·· |  | ·· | ·· | ·· |  |
| Nigeria | 84011 | 2024 | 73 | Increasing | 0·0 (0·0, 0·6) | < 500 (<500,500) | 2007-18 | 502,503 | 6·5 (3·1, 10·9) | 5500 (2500,9000) | 2000-17 | 502,504-510 | 13.0 (9.7, 16.8) | 11000 (8000,14000) | 2007-18 | 502,503 |
| Congo | 1388 | 2019 | 45 | Stable | 2·1 (0·0, 6·2) | < 500 (<500,<500) | 2012 | 511 | 8·3 (3·5, 14·8) | < 500 (<500,<500) | 2012 | 511 | ·· | ·· | ·· |  |
| Rwanda | 87621 | 2024 | 1123 | Increasing | ·· | ·· | ·· |  | ·· | ·· | ·· |  | 5.5 (5.3, 5.7) | 5000 (4500,5000) | 2017 | 512 |
| Sao Tome & Principe | 300 | 2023 | 239 | Increasing | ·· | ·· | ·· |  | ·· | ·· | ·· |  | ·· | ·· | ·· |  |
| Senegal | 13185 | 2023 | 142 | Increasing | ·· | ·· | ·· |  | 1·8 (0·9, 2·9) | < 500 (<500,500) | 2014-19 | 513,514 | 0.6 (0.0, 1.8) | < 500 (<500,<500) | 2014-19 | 513,514 |
| Seychelles | 474 | 2024 | 646 | Decreasing | ·· | ·· | ·· |  | ·· | ·· | ·· |  | ·· | ·· | ·· |  |
| Sierra Leone | 4453 | 2024 | 92 | Stable | ·· | ·· | ·· |  | 2·3 (1·0, 4·1) | < 500 (<500,<500) | 2015-21 | 515,516 | ·· | ·· | ·· |  |
| Somalia | 2799 | 2023 | 33 | ·· | ·· | ·· | ·· |  | ·· | ·· | ·· |  | ·· | ·· | ·· |  |
| South Africa | 157056 | 2023 | 405 | Stable | 1·4 (0·7, 2·2) | 2000 (1000,3500) | 2010 | 517 | 16·3 (6·7, 30·1) | 25500 (10500,47000) | 2010-20 | 517-522 | 3.2 (1.9, 4.7) | 5000 (3000,7500) | 2018-20 | 518 |
| United Republic of Tanzania | 32671 | 2022 | 96 | Decreasing | ·· | ·· | ·· |  | 8·0 (5·6, 11·0) | 2500 (2000,3500) | 2007-14 | 523-527 | 4.8 (2.9, 7.1) | 1500 (1000,2500) | 2007 | 524 |
| Togo | 4990 | 2021 | 102 | Stable | 1·0 (0·5, 1·6) | < 500 (<500,<500) | 2012 | 528 | 5·8 (3·9, 8·0) | 500 (<500,500) | 2011-13 | 513,528,529 | 0.3 (0.0, 1.2) | < 500 (<500,<500) | 2013 | 513 |
| Uganda | 78539 | 2024 | 322 | Increasing | ·· | ·· | ·· |  | 10·9 (8·2, 13·9) | 8500 (6500,11000) | 2008-14 | 530,531 | 0.8 (0.2, 1.6) | 500 (<500,1500) | 2008 | 531 |
| Zambia | 28225 | 2024 | 264 | Increasing | ·· | ·· | ·· |  | 23·5 (21·7, 25·5) | 6500 (6000,7000) | 1017-19 | 519,532-536 | 21.7 (18.2, 25.3) | 6000 (5000,7000) | 2011 | 534 |
| Zimbabwe | 20997 | 2024 | 236 | Stable | ·· | ·· | ·· |  | ·· | ·· | ·· |  | ·· | ·· | ·· |  |
| **Middle East & North Africa** |  |  |  |  |  |  |  |  |  |  |  |  |  |  |  |  |
| Algeria | 94749 | 2021 | 340 | Increasing | ·· | ·· | ·· |  | ·· | ·· | ·· |  | ·· | ·· | ·· |  |
| Bahrain | 3485 | 2017 | 310 | Increasing | ·· | ·· | ·· |  | ·· | ·· | ·· |  | ·· | ·· | ·· |  |
| Cyprus | 966 | 2024 | 112 | Increasing | 7·8 (3·9, 12·9) | < 500 (<500,<500) | 2022 | 537 | ·· | ·· | ·· |  | ·· | ·· | ·· |  |
| Egypt | 120000 | 2022 | 177 | Stable | ·· | ·· | ·· |  | 0·0 (0·0, 0·3) | < 500 (<500,500) | 2011 | 538 | 12.2 (9.5, 15.2) | 14500 (11500,18500) | 2011 | 538 |
| Iraq | 73715 | 2021 | 290 | Increasing | ·· | ·· | ·· |  | ·· | ·· | ·· |  | ·· | ·· | ·· |  |
| Israel | 19756 | 2023 | 371 | Decreasing | ·· | ·· | ·· |  | ·· | ·· | ·· |  | ·· | ·· | ·· |  |
| Jordan | 19140 | 2022 | 270 | Increasing | ·· | ·· | ·· |  | ·· | ·· | ·· |  | ·· | ·· | ·· |  |
| Kuwait | 5300 | 2024 | 168 | Increasing | ·· | ·· | ·· |  | ·· | ·· | ·· |  | ·· | ·· | ·· |  |
| Lebanon | 9254 | 2023 | 264 | Increasing | 12·3 (10·3, 14·6) | 1000 (1000,1500) | 2008 | 539,540 | 0·2 (0·0, 0·7) | < 500 (<500,<500) | 2008 | 540 | 1.9 (0.6, 3.8) | < 500 (<500,500) | 2008 | 539 |
| Libya | 19103 | 2023 | 428 | Increasing | ·· | ·· | ·· |  | 17·5 (16·6, 18·5) | 3500 (3000,3500) | 2006 | 541 | 17.9 (17.3, 18.6) | 3500 (3500,3500) | 2006 | 541,542 |
| Morocco | 102653 | 2023 | 421 | Increasing | ·· | ·· | ·· |  | 18·0 (3·8, 40·6) | 18500 (4000,41500) | 2004 | 543 | ·· | ·· | ·· |  |
| Oman | 1960 | 2015 | 62 | Increasing | ·· | ·· | ·· |  | ·· | ·· | ·· |  | ·· | ·· | ·· |  |
| Occupied Palestinian territories | ·· | N/A | ·· | ·· | ·· | ·· | ·· |  | ·· | ·· | ·· |  | ·· | ·· | ·· |  |
| Qatar | 2055 | 2022 | 92 | Increasing | ·· | ·· | ·· |  | ·· | ·· | ·· |  | ·· | ·· | ·· |  |
| Saudi Arabia | 68056 | 2017 | 280 | Increasing | ·· | ·· | ·· |  | 0·2 (0·1, 0·3) | < 500 (<500,<500) | 2022 | 544 | 0.3 (0.0, 0.8) | < 500 (<500,500) | 2022 | 545 |
| South Sudan | 8400 | 2021 | 149 | Increasing | NK | NK | ·· |  | ·· | ·· | ·· |  | ·· | ·· | ·· |  |
| Sudan | 21000 | 2017 | 93 | Stable | ·· | ·· | ·· |  | 2·0 (0·4, 4·5) | 500 (<500,1000) | 2002 | 546 | ·· | ·· | ·· |  |
| Syrian Arab Republic | 10599 | 2004 | 102 | Decreasing | ·· | ·· | ·· |  | 0·0 (0·0, 0·4) | < 500 (<500,<500) | 2014 | 547 | 1.3 (0.4, 2.6) | < 500 (<500,500) | 2014 | 547 |
| Tunisia | 23484 | 2021 | 289 | Increasing | ·· | ·· | ·· |  | ·· | ·· | ·· |  | ·· | ·· | ·· |  |
| Türkiye | 371587 | 2024 | 643 | Increasing | 16·1 (14·3, 17·9) | 59500 (53000,66500) | 2015-23 | 548-550 | 0·6 (0·1, 1·5) | 2000 (500,5500) | 2015-88 | 548-551 | 3.8 (2.7, 5.4) | 14000 (10000,20000) | 2012-88 | 548,550-553 |
| United Arab Emirates | 9826 | 2014 | 131 | Decreasing | ·· | ·· | ·· |  | ·· | ·· | ·· |  | ·· | ·· | ·· |  |
| Yemen | 4268 | 2022 | 23 | Decreasing | ·· | ·· | ·· |  | ·· | ·· | ·· |  | ·· | ·· | ·· |  |

**Notes:**

Ns are rounded to the nearest 500.

^1^ Country level data that informed these regional and global incarceration estimates were sourced from the World Prison Brief, collated by the Institute for Crime and Justice Policy Research at Burbeck University. See: https://www.prisonstudies.org/world-prison-brief-data. Note that we used the country estimates to make rates for 15-64 years (not the total country population), so our rates differ from the World Prison Brief estimates.

* Estimates of the prison population total range between 80,000 and 120,000.

† For reporting purposes, these countries or territories are reported separately due to differences in service provision.

‡ The following sources were found for HIV prevalence in Australian prisons, but due to the quality of the AusHep^447^ study we did not include them in the meta analysis: ^447,449,451,462,554-557^

§ The following sources were found for HCV prevalence in Australian prisons, but due to the quality of the AusHep^447^ study we did not include them in the meta analysis: ^447,449,451,453,454,462,463,554-569^

** No incarceration population estimate was available from the World Prison Brief, so no total estimates could be calculated for Eritrea.

·· Indicates that no estimates of the prevalence for that outcome were obtained for that country.

¶ The direction of the trend was based on the most recent estimate, with an average change of at least 10% over the past five years considered indicative of a trend.

NK Indicates no evidence was located that injecting drug use was occurring in this country.

HCV – Hepatitis C

Please see Appendix 5-8 for details of approach to assessment of study methodology and approach to selection and synthesis of data.

**References**

1. Weilandt C, Stöver H, Eckert J, Grigoryan G. Anonymous survey on infectious diseases and related risk behaviour among Armenian prisoners and prison staff. *International Journal of Prisoner Health* 2007.

2. Azbel L, Wickersham JA, Wegman MP, et al. Burden of substance use disorders, mental illness, and correlates of infectious diseases among soon-to-be released prisoners in Azerbaijan. *Drug and Alcohol Dependence* 2015.

3. Handanagic S. Report on the Integrated Bio-behavioural Surveillance Surveys among Key Populations in Azerbaijan, 2015. In: Ministry of Health of Republic of Azerbaijan WHO, WHO Collaborating Centre for HIV Surveillance, Zagreb Croatia, editor.; 2015.

4. V. Kasumov AK, D. Makhmudova, F. Juzbashov, S. Hasiev, S. Babazade, R. Sultanova, G. Kasumova, N. Kerimova. PREVALENCE OF HIV, HEPATITIS AND SYPHILIS, AND BEHAVIOURAL RISK FACTORS AMONG MOST-AT-RISK GROUPS IN THE REPUBLIC OF AZERBAIJAN. In: CENTRE MOHOTROARA, editor.; 2008.

5. Jurja-Ivana Čakalo SH. The report on results of a surveillance survey on knowledge, risks and prevalence of HIV and sexually and parenterally transmitted infections in most-at-risk populations in Azerbaijan. 2012.

6. Ravlija J, Vasilj I, Marijanovic I, Vasilj M. RISK BEHAVIOUR OF PRISON INMATES IN RELATION TO HIV/STI. *Psychiatria Danubina* 2014.

7. Hodžić H, Bajramović A, Obradović Z, Mahmić-Kaknjo M. Intravenous drugs abuse as the main risk factor of increasing hepatitis C infection prevalence in prisoners in Zenica, Bosnia and Herzegovina. *Med Glas (Zenica)* 2017.

8. Popov G, Plochev K. Prevalence and correlates of hepatitis C virus infection among inmates of Bulgarian prisons. *Clinical Microbiology and Infection* 2011.

9. Panayotov AS, G; Petkova, I; Ivanova, T; Metodieva, I; Chipeva, S. Annual Report on the problems related to drugs and drug addiction in Bulgaria: National Focus Center on Drugs and Addiction, 2022.

10. Popov G, Plochev K, Pekova L, Pishmisheva M, Popov T, Tchervenyakova T. Prevalence of viral hepatitis, human immunodeficiency virus and syphilis among inmates of Bulgarian prisons. *Journal of Hepatology* 2013.

11. Kivimets K, Uuskula A. HIV testing and counselling in Estonian prisons, 2012 to 2013: aims, processes and impacts. *Euro Surveill* 2014.

12. Bergen-Cico D, Sikharulidze K, Ivanashvili N, Ivanishvili M, Keshelava T. Hepatitis C Risk and Protective Factors Associated With Drug Policies in the Republic of Georgia. *World Medical and Health Policy* 2017.

13. Harris AM, Chokoshvili O, Biddle J, et al. An evaluation of the hepatitis C testing, care and treatment program in the country of Georgia's corrections system, December 2013 - April 2015. *BMC Public Health* 2019.

14. Gyarmathy VA, Neaigus A, Szamado S. HIV risk behavior history of prison inmates in Hungary. *AIDS Education and Prevention* 2003.

15. Tresó B, Barcsay E, Tarján A, et al. Prevalence and correlates of HCV, HVB, and HIV infection among prison inmates and staff, Hungary. *Journal of Urban Health* 2012.

16. Vanya M, Szili K, Magori K, Krisztina V. Skin diseases and sexually transmitted infection in a Hungarian prison. *Reviews and Research in Medical Microbiology* 2017.

17. Werling K, Makara M, Nemesi K, et al. Screening and treatment of hepatitis C virus in prisons: 10 years of experience. *Orvosi Hetilap* 2022.

18. Werling K, Hunyady B, Makara M, et al. Hepatitis C Screening and Treatment Program in Hungarian Prisons in the Era of Direct Acting Antiviral Agents. *Viruses* 2022.

19. Kurcalte O SM, Manson E, Karadzhan J, Krastiÿš I, & Zalans O. Analysis of trends in the use of addictive substances in prisons in Latvia in 2022": Final report, 2023.

20. Narkauskaitė L, Juozulynas A, Mackiewicz Z, Venalis A, Utkuvienė J. Prevalence of psychoactive substances use in a Lithuanian women's prison revisited after 5 years. *Medical Science Monitor* 2010.

21. Rasimaite B. DRID in prisons Lituhania. 2024.

22. Sultana C, Falanga C, Chicin G, et al. HIV, HCV and HIV-HCV Coinfections in the General Population versus Inmates from Romania. *Viruses* 2024; **16**(8).

23. Bivegete S, Ward Z, Walker J, et al. Comparison of costs of different HCV screening and linkage to care interventions across Europe. *Journal of Hepatology* 2020.

24. Nazare C, Girleanu I, Cojocariu-Salloum C, Trifan A. [Characteristics of hepatitis C virus (HCV) infection in closed communities]. *Revista medico-chirurgicala a Societatii de Medici si Naturalisti din Iasi* 2011.

25. Azbel L, Wickersham JA, Grishaev Y, Dvoryak S, Altice FL. Burden of infectious diseases, substance use disorders, and mental illness among Ukrainian prisoners transitioning to the community. *PLoS One* 2013.

26. Balakireva O SV, Salabai N, Kryvoruk A. Analysis of HIV/AIDS Response in Penitentiary System of Ukraine: Ukrainian Institute for Social Research after Olexander Yaremenko; UNODC, 2012.

27. Kiriazova T, Sereda Y. History of injection drug use mediates the effect of project start intervention in men who were released from prison in Ukraine. *Drug and Alcohol Dependence* 2017.

28. Osinskaya T, Zapolsky M, Shcherbakova Y, Dzhoraieva S. Prevalence of Chlamydia among Women in Places of Deprivation of Liberty. *Georgian Medical News* 2023; (337): 34-7.

29. Silbernagl M, Slamanig R, Fischer G, Brandt L. Hepatitis C infection and psychiatric burden in two imprisoned cohorts: Young offenders and opioid-maintained prisoners. *Health Policy* 2018.

30. Busschots D, Kremer C, Bielen R, et al. A multicentre interventional study to assess blood-borne viral infections in Belgian prisons. *BMC Infectious Diseases* 2021.

31. Todts S, Glibert P, Van Malderen S, Van Huyck C, Saliez V, Hogge M. Usage de drogues dans les prisons belges: monitoring des risques sanitaires. *Bruxelles: SPF Justice* 2008.

32. Plettinckx E HN, de Smet S, Gremeaux L, Dirkx N. Health, well-being and drug use among persons in prison - Belgian results of the PRS-20 project 2021-2023, 2023.

33. Burek V, Horvat J, Butorac K, Mikulić R. Viral hepatitis B, C and HIV infection in Croatian prisons. *Epidemiol Infect* 2010.

34. Vilibic-Cavlek T, Gjenero-Margan I, Retkovac B, et al. Sociodemographic characteristics and risk behaviors for HIV, hepatitis B and hepatitis C virus infection among Croatian male prisoners. *International Journal of Prison Health* 2011; **7**(1): 28-31.

35. Burek V, Horvat J, Susic E, Mikulic R. Prevalence of hepatitis B and C among prison population in Croatia. [Croatian]. *Acta Medica Croatica* 2009.

36. Christensen PB, Krarup HB, Niesters HGM, Norder H, Georgsen J. Prevalence and incidence of bloodborne viral infections among Danish prisoners. *European Journal of Epidemiology* 2000.

37. Soholm J, Holm DK, Mossner B, et al. Incidence, prevalence and risk factors for hepatitis C in Danish prisons. *PLoS One* 2019.

38. Aisyah DN, Shallcross L, Hayward A, et al. Hepatitis C among vulnerable populations: A seroprevalence study of homeless, people who inject drugs and prisoners in London. *Journal of Viral Hepatitis* 2018.

39. Boys A, Farrell M, Bebbington P, et al. Drug use and initiation in prison: results from a national prison survey in England and Wales. *Addiction* 2002.

40. Jack K, Smith SA, Lloyd J, Smith H, Thomson BJ. Hepatitis B and C management pathways in prison: An audit against UK NICE public health guidance (2013); 2013.

41. Jack K. Hepatitis C virus infection risk factors and test uptake in an English prison. *Gastrointestinal Nursing* 2020.

42. Mahto M, Zia S. Measuring the gap: from Home Office to the National Health Service in the provision of a one-stop shop sexual health service in a female prison in the UK. *International Journal of STD & AIDS* 2008.

43. Plugge E, Yudkin P, Douglas N. Changes in women's use of illicit drugs following imprisonment. *Addiction* 2009.

44. Weild AR, Gill ON, Bennett D, Livingstone SJ, Parry JV, Curran L. Prevalence of HIV, hepatitis B, and hepatitis C antibodies in prisoners in England and Wales: a national survey. *Communicable Disease and Public Health* 2000.

45. Morey S, Hamoodi A, Jones D, et al. Increased diagnosis and treatment of hepatitis C in prison by universal offer of testing and use of telemedicine. *Journal of Viral Hepatitis* 2019.

46. Phaw NA, Thant AM, Thompson C, et al. Prospective evaluation of the impact of repeated whole prison testing for hepatitis C. *BMJ Open Gastroenterol* 2025; **12**(1).

47. Allsop C, McCullough F, Miller C, et al. Impact of a 'high intensity test and treat' initiative for Hcv in low newton prison. *Gut* 2021.

48. Bhandari R, Morey S, Hamoodi A, et al. High rate of hepatitis C reinfection following antiviral treatment in the North East England Prisons. *Journal of Viral Hepatitis* 2020.

49. Connoley D, Francis-Graham S, Storer M, et al. Detection, stratification and treatment of hepatitis C-positive prisoners in the United Kingdom prison estate: Development of a pathway of care to facilitate the elimination of hepatitis C in a London prison. *Journal of Viral Hepatitis* 2020.

50. Davies L, Healy B, Matthews G, et al. Elimination of hepatitis C in a remand prison using a rapid point of care driven test and treat pathway. *Journal of Hepatology* 2020.

51. Duncan S, Sherrard J. Experience of screening for hepatitis C in an oxfordshire prison. *Sexually Transmitted Infections Conference: STI and AIDS World Congress* 2013.

52. Halford R, Christensen L, Cox S, et al. Chronic hepatitis C elimination prison initiative: HCV-intensive test and treat, a whole prisoner population HCV test-and-treat program in England. *Health Science Reports* 2023; **6(12) (no pagination)**.

53. Horne JA, Clements AJ, Drennan P, Stein K, Cramp ME. Screening for hepatitis C virus in the Dartmoor prison population: an observational study. *Journal of Public Health (Oxf)* 2004.

54. Jack K, Thomson B, Irving W. Outcomes of an opt-out strategy for Hepatitis C testing in the East Midlands prison estate. *Journal of Hepatology* 2018.

55. Johnson A, Shearer J, Thompson C, et al. Impact of 5 years of hepatitis C testing and treatment in the North East of England prisons. *Journal Of Viral Hepatitis* 2023; **30**(12): 914-21.

56. Kirwan P, Evans B, Brant L, Sentinel Surveillance Hepatitis T. Hepatitis C and B testing in English prisons is low but increasing. *Journal of Public Health* 2011.

57. Mohamed Z, Al-Kurdi D, Nelson M, et al. Time matters: Point of care screening and streamlined linkage to care dramatically improves hepatitis C treatment uptake in prisoners in England. *International Journal of Drug Policy* 2020.

58. Mongale E, Allen S, Brew I, et al. Development and optimisation of a reception testing protocol designed to eliminate HCV in the UK prison population. *JHEP Reports* 2024; **6(1) (no pagination)**.

59. Patel S, Clarke B, Bird G. Hepatitis B and hepatitis c virus case finding in a medium security UK prison. *Canadian Journal of Gastroenterology and Hepatology Conference* 2016.

60. Skipper C, Guy JM, Parkes J, Roderick P, Rosenberg WM. Evaluation of a prison outreach clinic for the diagnosis and prevention of hepatitis C: implications for the national strategy. *Gut* 2003.

61. West A, Deverell H, Brown A, et al. First hepatitis C mass-testing in a category A prison in the United Kingdom. *Journal of Hepatology* 2023; **78(Supplement 1)**: 848.

62. Rautanen M, Harald, K, & Tyni, S. Health and Wellbeing of Prisoners 2023 The Wattu IV Prison Population Study Finland, 2024.

63. Viitanen P, Vartiainen H, Aarnio J, et al. Hepatitis A, B, C and HIV infections among Finnish female prisoners--young females a risk group. *Journal of Infection* 2011.

64. Rautanen M, Harald, K, & Tyni, S. The Health and Wellbeing of Finnish Prisoners 2023 (Wattu IV). *Finnish Institute for Health and Welfare (THL) Report 007/2023 256 pages Helsinki 2023* 2023.

65. Jacomet C, Guyot-Lénat A, Bonny C, et al. Addressing the challenges of chronic viral infections and addiction in prisons: the PRODEPIST study. *European Journal of Public Health* 2016.

66. Marzo JN, Rotily M, Meroueh F, et al. Maintenance therapy and 3-year outcome of opioid-dependent prisoners: a prospective study in France (2003-06). *Addiction* 2009.

67. Messiah A, Escaffre N, Sannino N, Rotily M, Galinier-Pujol A. Sexuality in the age of AIDS in a vulnerable population: findings from a survey on prisoners. *Population* 2001.

68. Remy AJ, Roy B, Hervet J. The 'prison zero hepatitits' project in france: a new pathway for hcv microelimination. *Heroin Addiction and Related Clinical Problems* 2021.

69. Rotily M, Delorme C, Galinier A, Escaffre N, Moatti JP. HIV risk behavior in prison and factors related to reincarceration among injecting drug users. *Presse Medicale* 2000.

70. Rotily M, Weilandt C, Bird SM, et al. Surveillance of HIV infection and related risk behaviour in European prisons: A multicentre pilot study. *European Journal of Public Health* 2001.

71. Roux P, Sagaon-Teyssier L, Lions C, Fugon L, Verger P, Carrieri MP. HCV seropositivity in inmates and in the general population: an averaging approach to establish priority prevention interventions. *BMJ Open* 2014.

72. Verneuil L, Vidal JS, Bekolo RZ, et al. Prevalence and risk factors of the whole spectrum of sexually transmitted diseases in male incoming prisoners in France. European Journal of Clinical Microbiology & Infectious Diseases 2009.

73. Abel S, Cuzin L, Da Cunha S, et al. Reaching the WHO target of testing persons in jails in prisons will need diverse efforts and resources. *PLoS One* 2018.

74. Izquierdo L, Mellon G, Buchaillet C, et al. Prevalence of hepatitis E virus and reassessment of HIV and other hepatitis virus seroprevalences among French prison inmates. *PLoS One* 2019.

75. Lelievre C, Prissette G, Reuche AA, et al. Detection of sexually transmitted infections at the Amiens prison. State of play from February 2019 to May 2019. *Revue de Medecine Legale* 2020.

76. Perrodeau F, Pillot-Debelleix M, Vergniol J, et al. Optimizing hepatitis B vaccination in prison. *Med Mal Infect* 2016.

77. Reynaud-Maurupt C, Caer Y, Escaffre N, et al. High-dose buprenorphine substitution during incarceration - Management of opiate addicts. *Presse Medicale* 2005.

78. Semaille C, Le Strat Y, Chiron E, et al. Prevalence of human immunodeficiency virus and hepatitis C virus among French prison inmates in 2010: a challenge for public health policy. *Euro surveillance : bulletin Europeen sur les maladies transmissibles = European communicable disease bulletin* 2013.

79. Michault A, Faulques B, Sevadjan B, Troalen D, Marais A, Barau G. Prevalence of hepatitis A, B, C virus markers in Reunion (south hospital and Saint Pierre prison). [French]. *Bulletin de la Societe de pathologie exotique (1990)* 2000.

80. Remy AJ. Hepatitis C in prison settings: Screening and therapy are improving - Comparative survey between 2000 and 2003. [French]. *Presse Medicale* 2006.

81. Remy AJ, Roy B, Hervet J, Bouchkira H. Lack of COVID-19 impact on managing hepatitis c in prison like the general population! *Hepatology* 2021.

82. Vergniol J, Capdepont M, El Aouadi S, et al. Prevalence of viral hepatitis and liver fibrosis in a population of incomers in French prisons. UCSASCAN study. *Hepatology* 2014.

83. Schulte B, Stover H, Thane K, Schreiter C, Gansefort D, Reimer J. Substitution treatment and HCV/HIV-infection in a sample of 31 German prisons for sentenced inmates. *International Journal of Prisoner Health* 2009.

84. Lehmann M, Meyer MF, Monazahian M, Tillmann HL, Manns MP, Wedemeyer H. High rate of spontaneous clearance of acute hepatitis C virus genotype 3 infection. *Journal of Medical Virology* 2004.

85. Fotiadou M, Livaditis M, Manou I, et al. Self-reported substance misuse in Greek male prisoners. *Eur Addict Res* 2004.

86. Koulierakis G. Drug use and related precautions prior to imprisonment, inside prison and intentions after release among Greek inmates. *Addiction Research & Theory* 2006.

87. Koulierakis G, Gnardellis C, Agrafiotis D, Power KG. HIV risk behaviour correlates among injecting drug users in Greek prisons. *Addiction* 2000.

88. Koulierakis G, Power KG, Gnardellis C, Agrafiotis D. HIV/AIDS related knowledge of inmates in Greek prisons. *Addiction Research & Theory* 2003.

89. Tourkochristou E, Beskos G, Kanaloupitis S, et al. Prevalence of anti-HCV antibodies and risk factors among prison inmates in Southwestern Greece. *Achaiki Iatriki* 2020; **39**: 23-8.

90. Fridriksdottir R, Ingibergsdotir, B, Frodjonsdottir, H, Alexiusdottir, K, Tomasdottir, A, Bjornsdottir, Th, Finnbogsdottir, A, Olafsdottir, B, Tyrfingsson, Th, Runarsdottir, V, Bergmann, OM, Bjornsson, ES, Johansson, B, Sigurdardottir, B, Heimisdottir, M, Olafsson, S & Gottfredsson, M. Sustained reduction in prevalence of hepatitis C viremia in the prison setting after 3rd year of TrapHepC (Treatment as Prevention for hepatitis C) program in Iceland. 2020.

91. Allwright S, Bradley F, Long J, Barry J, Thornton L, Parry JV. Prevalence of antibodies to hepatitis B, hepatitis C, and HIV and risk factors in Irish prisoners: results of a national cross sectional survey. *BMJ* 2000.

92. Crowley D, Lambert JS, Betts-Symonds G, et al. The seroprevalence of untreated chronic hepatitis C virus (HCV) infection and associated risk factors in male Irish prisoners: a cross-sectional study, 2017. *Euro Surveill* 2019.

93. Drummond A, Codd M, Donnelly N, et al. Study on the prevalence of drug use, including intravenous drug use, and blood-borne viruses among the Irish prisoner population. *Dublin: National Advisory Committee on Drugs and Alcohol* 2014.

94. Long J, Allwright S, Barry J, et al. Prevalence of antibodies to hepatitis B, hepatitis C, and HIV and risk factors in entrants to Irish prisons: a national cross sectional survey...including commentary by Bird SM. *BMJ: British Medical Journal (International Edition)* 2001.

95. Bannan CL, Lynch PA, Conroy EP, et al. Point-of-care testing for HIV in an Irish prison setting: results from three major Irish prisons. *International Journal of STD & AIDS* 2016.

96. National Advisory Committee on Drugs and Alcohol. Drugs and health in Irish prisons 2011. A report for prisoners., 2014.

97. Wright B, Duffy D, Curtin K, Linehan S, Monks S, Kennedy HG. Psychiatric morbidity among women prisoners newly committed and amongst remanded and sentenced women in the Irish prison system. *Irish Journal of Psychological Medicine* 2006.

98. Long J, Allwright S, Barry J, et al. Prevalence of antibodies to hepatitis B, hepatitis C, and HIV and risk factors in entrants to Irish prisons: a national cross sectional survey. *BMJ* 2001.

99. Babudieri S, Longo B, Sarmati L, et al. Correlates of HIV, HBV, and HCV infections in a prison inmate population: results from a multicentre study in Italy. *Journal of Medical Virology* 2005.

100. Ciccarese G, Drago F, Oddenino G, Crosetto S, Rebora A, Parodi A. Sexually transmitted infections in male prison inmates. Prevalence, level of knowledge and risky behaviours. *Infez Med* 2020.

101. Fiore V, De Vito A, Rastrelli E, et al. Differences in HCV Seroprevalence, Clinical Features, and Treatment Outcomes between Female and Male Incarcerated Population: Results from a Matched Cohort Study. *Viruses* 2023; **15**(12).

102. Fiore V, De Matteis G, Ranieri R, et al. HCV testing and treatment initiation in an Italian prison setting: A step-by-step model to micro-eliminate hepatitis C. *International Journal of Drug Policy* 2021.

103. Izzo C, Monica A, De Matteis G, et al. Not Only COVID-19: Prevalence and Management of Latent Mycobacterium Tuberculosis Infection in Three Penitentiary Facilities in Southern Italy. *Healthcare* 2022; **10**(2).

104. Izzo C, Masarone M, Torre P, et al. Solving the Gap Between HCV Detection and Treatment in Prison HCV-RNA Testing and Treatment in a Cohort of Newly Arrived Convicts in Southern Italy. *Reviews on Recent Clinical Trials* 2022.

105. Marco LD, Tullio P, Scalici F, et al. Screening and linkage to care of prisoners with HCV infection: the resist-HCV project. *Journal of Hepatology* 2020.

106. Scelza G, Amato A, Pagano AM, et al. Effect of hepatitis C antiviral therapy on oral lichen planus and hyposalivation in inmates. *Annals of Gastroenterology* 2022.

107. Geremia N, Giovagnorio F, De Vito A, et al. HBV in Italian Women's Jail: An Underestimated Problem? *Journal Of Clinical Medicine* 2024; **13(5) (no pagination)**.

108. Giuliani R, Casigliani V, Fornili M, et al. HCV micro-elimination in two prisons in Milan, Italy: A model of care. *Journal of Viral Hepatitis* 2020.

109. Monarca R, Madeddu G, Ranieri R, et al. HIV treatment and care among Italian inmates: a one-month point survey. *BMC Infectious Diseases* 2015.

110. Ranieri R, Sommella J, D'Angelo C, et al. Antiretroviral therapy in inmates: Between guidelines and reality of Italian correctional facilities. [Italian]. *Infezioni in Medicina* 2015.

111. Sagnelli E, Starnini G, Sagnelli C, et al. Blood born viral infections, sexually transmitted diseases and latent tuberculosis in italian prisons: a preliminary report of a large multicenter study. *European Review for Medical and Pharmacological Sciences* 2012.

112. Stasi C, Silvestri C, Fanti E, Di Fiandra T, Voller F. Prevalence and features of chronic viral hepatitis and HIV coinfection in Italian prisons. *European Journal of Internal Medicine* 2016.

113. Voller F, Silvestri C, Orsini C, Aversa L, Da Frè M, Cipriani F. [The health conditions of prison inmates in Tuscany]. *Epidemiology and Prevalence* 2011.

114. Brandolini M, Novati, S., De Silvestri A, Tinelli C, Patruno SFA, Ranieri R, Seminari E. Prevalence and epidemiological correlates and treatment outcome of HCV infection in an Italian prison setting. *BMC Public Health* 2013.

115. Cartabellotta F, Di Marco L, Santangelo F, et al. Eliminating Hcv Infection from Prisons in Sicily: The Sintesi Project. *Hepatology* 2023; **78(Supplement 1)**: S1308-S9.

116. Caruso R, Aglitti A, Di Zenzo C, et al. Hepatitis C virus prevalence in a cohort of jailbirds in a city of Southern Italy. *Digestive and Liver Disease* 2019.

117. Di Marco L, Cartabellotta F, Santangelo F, et al. Eliminating HCV infection from prisons in sicily: the SINTESI project. *Digestive and Liver Disease* 2024; **56(Supplement 1)**: S15.

118. Masarone M, Caruso R, Aglitti A, et al. Hepatitis C virus infection in jail: Difficult-to-reach, not to-treat. Results of a point-of-care screening and treatment program. *Digestive and Liver Disease* 2020.

119. Montella M, Crispo A, Grimaldi M, et al. Prevalence of hepatitis C virus infection in different population groups in southern Italy. *Infection* 2005.

120. Ranieri R, Foschi A, Casana M, et al. Treating HCV-positive Italian inmates with direct-acting antivirals: the clinical experience in three major correctional houses of Milan. *Journal of the International AIDS Society* 2016.

121. Teyssier E, Kugener T, Seixas R, Berndt N., Seixas R., Kugener T., Origer A. PRS20: Luxembourg Results of the national quantitative study. 2023.

122. Muscat K, Cremona C, Fenech TM, Abela M, Padovese V. Sexually transmitted infections epidemiology and risk assessment at the main correctional facility in Malta (2017-2019). *Journal of the European Academy of Dermatology and Venereology* 2022.

123. Bakić M, Stevanović J, Milić M, et al. Factors associated with the prevalence of viral hepatitis B and C among prisoners: Results of two consecutive national surveys in Montenegro. *PLoS One* 2025; **20**(4): e0321464.

124. Schreuder I, van der Sande MA, Osterhaus AD, et al. No HIV infections despite high numbers of hepatitis B and C virus infections in Dutch prisoners. *Journal of Public Health and Epidemiology* 2011; **3**(6): 284-93.

125. Jovanovska T, Kocic B, Stojcevska VP. Prevalence, attitudes and knowledge about HIV HBV and HCV infections among inmates in prisons Prilep and Bitola--a pilot study. *Collegium Antropologicum* 2014.

126. Danis K, Doherty L, McCartney M, McCarrol J, Kennedy H. Hepatitis and HIV in Northern Ireland prisons: a cross-sectional study. *Euro Surveill* 2007; **12**(1).

127. Bukten A, Lund IO, Kinner SA, et al. Factors associated with drug use in prison – results from the Norwegian offender mental health and addiction (NorMA) study. *Health & Justice* 2020.

128. Hannula R, Soderholm J, Svendsen T, et al. Hepatitis C outreach project and cross-sectional epidemiology in high-risk populations in Trondheim, Norway. *Therapeutic Advances in Infectious Disease* 2021.

129. Barros H, Ramos E, Lucas R. A survey of HIV and HCV among female prison inmates in Portugal. *Central European Journl of Public Health* 2008.

130. Garcia A, Exposto F, Prieto E, Lopes M, Duarte A, da Silva RC. Association of Trichomonas vaginalis with sociodemographic factors and other STDs among females inmates in Lisbon. *International Journal of STD & AIDS* 2004.

131. Passadouro R. [Prevalence infections and risk factors due to HIV, Hepatitis B and C in a prison establishment in Leiria]. *Acta Médica Portuguesa* 2004.

132. Morgado D, Alves J, Martinho G, Gonçalves M, Cerqueira A, Maia A. Concordance Between Health Records and a Self-Report Measure in a Sample of Female Inmates in Portugal. *Women & Criminal Justice* 2025.

133. da Silva Marques NM, Margalho R, Melo MJ, da Cunha JGS, Melico-Silvestre AA. Seroepidemiological survey of transmissible infectious diseases in a Portuguese prison establishment. *Brazilian Journal of Infectious Diseases* 2011.

134. Carvalhana S, Pinto R, Leitao J, et al. HCV and HBV prevalence in the population: Large disparity between hepatitis c in the general population, comparing with high risk groups. *United European Gastroenterology Journal* 2014.

135. Gaspar R, Liberal R, Tavares J, Morgado R, Macedo G. HIPPOCRATES((R)) project: A proof of concept of a collaborative program for hepatitis C virus micro-elimination in a prison setting. *World Journal of Hepatology* 2020.

136. Liberal R, Gaspar R, Andrade P, et al. Doctors in jails: A proof of concept for tackling hepatitis C in prisons. *Hepatology* 2017.

137. Marques NMD, Margalho R, Melo MJ, da Cunha JGS, Melico-Silvestre AA. Seroepidemiological survey of transmissible infectious diseases in a Portuguese prison establishment. *Brazilian Journal of Infectious Diseases* 2011.

138. Arora G, Humphris G, Lahti S, Richards D, Freeman R. Depression, drugs and dental anxiety in prisons: A mediation model explaining dental decay experience. *Community Dentistry and Oral Epidemiology* 2020.

139. Morrison DS, Gilchrist G. Prison admission health screening as a measure of health needs. *Health Bulletin (Edinb)* 2001.

140. Taylor A, Munro A, Allen E, et al. Low incidence of hepatitis C virus among prisoners in Scotland. [References]. *Addiction* 2013.

141. Peters SE, Bissett B, Cassells Y, Paton J, Aitken C. HIV testing and care in prisoners: The first year results of opt-out BBV testing in Glasgow, UK. *Journal of the International AIDS Society* 2016.

142. Champion JK, Taylor A, Hutchinson S, et al. Incidence of hepatitis C virus infection and associated risk factors among Scottish prison inmates: a cohort study. *American Journal of Epidemiology* 2004.

143. Abdo Sanmartino IJ, Guerrero-Moreno RA, Mouriño AM. Changes in the epidemiological pattern of chronic B hepatitis amongst inmates in Catalonia: current prevalence and predictive variables. *Revista Española de Sanidad Penitenciaria* 2023; **25**(3): 89-97.

144. Brime B, Llorens, N & Sanchez, E. SURVEY ON HEALTH AND DRUG USE IN THE INMATE POPULATION IN PENITENTIARY INSTITUTIONS (ESDIP), 2022.

145. Cuadrado A, Llerena S, Cobo C, et al. Microenvironment Eradication of Hepatitis C: A Novel Treatment Paradigm. *American Journal of Gastroenterology* 2018.

146. Ferrer-Castro V, Crespo-Leiro MR, García-Marcos LS, et al. [Evaluation of needle exchange program at Pereiro de Aguiar prison (Ourense, Spain): ten years of experience]. *Revista Española de Sanidad Penitenciaria* 2012.

147. García-Guerrero J MMA, Sáiz de la Hoya Zamácola P, Vera-Remartínez EJ. Multi-centre study of the prevalence of latent tuberculosis infection amongst inmates in Spanish prisons. 2010.

148. Martin V, Guerra JM, Cayla JA, Rodriguez JC, Blanco MD, Alcoba M. Incidence of tuberculosis and the importance of treatment of latent tuberculosis infection in a Spanish prison population. *International Journal of Tuberculosis and Lung Disease* 2001.

149. Murcia J, Portilla J, Bedia M, et al. Chronic hepatitis C virus infection and associated liver disease among the inmates of a Spanish prison. *Enfermedades Infecciosas y Microbiologia Clinica* 2009.

150. Sánchez Recio R, Alonso Pérez de Ágreda JP, Santabárbara Serrano J. [Sexually transmitted infections in male prison inmates: risk of development of new diseases]. *Gaceta Sanitaria* 2016.

151. Gonzalez C, Canals J, Ortiz M, et al. Prevalence and determinants of high-risk human papillomavirus (HPV) infection and cervical cytological abnormalities in imprisoned women. *Epidemiology and Infection* 2008.

152. Marco A, Gallego, C, Escribano, M, Arguelles, MJ, Sole, N & Sanchez-Roig, M. Prevalencia de diagnóstico tardío y de infección avanzada en los casos con infección por vih detectados en dos prisiones de Barcelona. Revista Espanola de Medicina Penitentiaria 18th SESP Conference. Barcelona; 2014.

153. Marco A, Saiz de la Hoya P, Garcia-Guerrero J, Grupo P. [Multi-centre study of the prevalence of infection from HIV and associated factors in Spanish prisons]. [Spanish]. *Revista Española de Sanidad Penitenciaria* 2012.

154. Marco Mouriño A, Rivera-Esteban J, Augustin S, Turu Santigosa E, Pericàs JM. [Metabolic morbidity in the prison population of Catalonia, Spain]. *Atención Primaria* 2023; **55**(6): 102620.

155. Marco A, Gallego C, Perez-Caceres V, et al. Public Health response to an outbreak of SARS-CoV2 infection in a Barcelona prison. *Epidemiology and Infection* 2021.

156. Mourino AM, Gallego Castellvi C, Garcia De Olalla P, et al. Late diagnosis of HIV infection among prisoners. *AIDS Reviews* 2013.

157. Olivan G. The health profile of Spanish incarcerated delinquent youths. [References]. *Journal of Adolescent Health* 2001.

158. Serroukh SC-Y. Consumo de sustancias, tratamiento con psicofármacos y patología infecciosa en personas redusas del centro penitenciario Puig de las Basses. *Metas de Enfermería* 2022.

159. Vicente-Alcalde N, Tuells J, Egoavil CM, Ruescas-Escolano E, Altavilla C, Caballero P. Immunization Coverage of Inmates in Spanish Prisons. *International Journal of Environmental Research and Public Health* 2020.

160. Yela E, Puig L, De Odriozola M, et al. Tuberculin test measurement at 48 and 72 hours: mismatch and clinical significance. *Revista Española de Sanidad Penitenciaria* 2024; **26**(3): 92-7.

161. Cabezas J, Castrejon OM, Acin E, et al. Hepatitis C infection in the Spanish prison system. Elimination is a dream at our fingertips. *Journal of Hepatology* 2020.

162. Gil-Sierra M, Tellez-Perez F, Rios-Sanchez E, et al. 4CPS-085 Stewardships of hepatitis c virus patients in prisons. British Medical Journal Publishing Group; 2019.

163. Lerena SL, Cobo C, Alvarez S, et al. A program of testing and treat intended to eliminate hepatitis c in a prison: The JAILFREE-C study. *Hepatology* 2016.

164. Saiz De La Hoya P, Bedia M, Murcia J, Cebria J, Sanchez-Paya J, Portilla J. Predictive markers of HIV and HCV infection and co-infection among inmates in a Spanish prison. [Spanish]. *Enfermedades Infecciosas y Microbiologia Clinica* 2005.

165. Saludes V, Bordoy AE, Yela E, et al. Incidence and molecular epidemiology of hepatitis C virus reinfection in prisons in Catalonia, Spain (Re-HCV study). *Scientific Reports* 2023; **13**(1): 16012.

166. de la Hoya PS, Marco A, Garcia-Guerrero J, Rivera A, Prevalhep Study G. Hepatitis C and B prevalence in Spanish prisons. *European Journal of Clinical Microbiology & Infectious Diseases* 2011.

167. Gahrton C, Westman G, Lindahl K, et al. Prevalence of Viremic hepatitis C, hepatitis B, and HIV infection, and vaccination status among prisoners in Stockholm County. *BMC Infectious Diseases* 2019.

168. Baggio S, Pala KC, Rieder JP, Tran NT, Wolff H, Getaz L. Infectious diseases in post-trial detention and comparisons with pre-trial detention: A study in Geneva, Switzerland. *Journal of Infection and Public Health* 2020.

169. Pala KC, Baggio S, Tran NT, Girardin F, Wolff H, Getaz L. Blood-borne and sexually transmitted infections: a cross-sectional study in a Swiss prison. *BMC Infectious Diseases* 2018.

170. Wolff H, Favrod-Coune T, Baroudi M, et al. Substitution treatment for all dependent opioid users is possible in jail: A case study of Switzerland. *Journal of General Internal Medicine* 2012.

171. Moschetti K, Stadelmann P, Wangmo T, et al. Disease profiles of detainees in the Canton of Vaud in Switzerland: gender and age differences in substance abuse, mental health and chronic health conditions. *BMC Public Health* 2015.

172. Blogg S, Utomo, B,, Silitonga N, Hidayati DAN, Sattler G. Indonesian National Inmate Bio-Behavioral Survey for HIV and Syphilis Prevalence and Risk Behaviors in Prisons and Detention Centers, 2010. *SAGE Open* 2014.

173. Kemenkes R. Integrated Biological and Behavioural Survey 2011. *Jakarta: Kementerian Kesehatan RI* 2011.

174. Niode NJ, Raranta H, Purwanto DS, Mamuaja EH, Tallei TE. The prevalence and risk factors of sexually transmitted infections among correctional institution inmates in Manado, Indonesia. *Journal of Pakistan Association of Dermatologists* 2024; **34(2)**: 445-52.

175. Sembiring E, Ginting Y, Saragih RH. Factors associated with syphilis seropositive and Human Immunodeficiency Virus (HIV) infection among inmates at Lubuk Pakam prison, Indonesia. *1ST INT CONF ON TROP MED & INFECT DIS FAC OF MED UNIV SUMATERA UTARA IN CONJUNCTION WITH THE 23RD NATL CONGRESS OF THE INDONESIAN SOC OF TROP & INFECT DIS CONSULTANT AND THE 18TH ANNUAL MEETING OF INTERNAL MED DEPT FAC OF MED UNIV SUMATERA UTARA* 2018.

176. Arends RM, Nelwan EJ, Soediro R, et al. Associations between impulsivity, risk behavior and HIV, HBV, HCV and syphilis seroprevalence among female prisoners in Indonesia: A cross-sectional study. *PLoS One* 2019.

177. Ministry of Health Republic of Indonesia. IBBS 2011 Integrated Bioloigcal and Behavioral Survey, 2011.

178. Rey I, Saragih R, Effendi-Ys R, Sembiring J, Siregar G, Zain L. Profile of hepatitis B and C virus infection in prisoners in Lubuk Pakam correctional facilities. IOP Conference Series: Earth and Environmental Science; 2018: IOP Publishing; 2018. p. 012033.

179. Hetty W, Alima S, Heri A, et al. Eliminating HCV within prisons in Jakarta. *Hepatology International* 2020.

180. Suan MAM, Zulkifli AM, Ani NH, et al. Feasibility of a prison-based test-and-treat model for enhancing hepatitis C care in Kedah, Malaysia. *BMC Public Health* 2025; **25**(1): 1152.

181. Mwe Nom NA, Kyaw KWY, Kumar AMV, et al. HIV care cascade among prisoners of the Mandalay Central Prison in Myanmar: 2011-2018. *Tropical Medicine and Infectious Disease* 2020.

182. Simbulan NP, Aguilar AS, Flanigan T, Cu-Uvin S. High-risk behaviors and the prevalence of sexually transmitted diseases among women prisoners at the women state penitentiary in Metro Manila. *Social Science & Medicine* 2001.

183. Choi S, Lee E, Bang JH. High Prevalence of Human Immunodeficiency Virus Infection among Inmates in Korean Correctional Facilities. *Journal of Korean Medical Science* 2021.

184. Feng MC, Feng JY, Chen YH, Chang PY, Lu PL. Prevalence and knowledge of sexual transmitted infections, drug abuse, and AIDS among male inmates in a Taiwan prison. *Kaohsiung Journal of Medical Science* 2012.

185. Lin CF, Twu SJ, Chen PH, Cheng JS, Wang JD. Prevalence and determinants of hepatitis B antigenemia in 15,007 inmates in Taiwan. *Journal of Epidemiology* 2010.

186. Lu MY, Chen CT, Shih YL, et al. Changing epidemiology and viral interplay of hepatitis B, C and D among injecting drug user-dominant prisoners in Taiwan. *Scientific Reports* 2021.

187. Yang TH, Fang YJ, Hsu SJ, et al. Microelimination of chronic hepatitis C by universal screening plus direct-acting antivirals for incarcerated persons in Taiwan. *Open Forum Infectious Diseases* 2020.

188. Harnpariphan W, Han, W. M., Supanun R, Ubolyam S, et al. High Proportion of Blood-Borne and Sexually Transmitted Infections Among People Deprived of Liberty in a Central Male Prison in Thailand: A Cross-Sectional Study 2018-2019. *AIDS Research and Human Retroviruses* 2022.

189. Morasert T, Worapas W, Kaewmahit R, Uphala W. Prevalence and risk factors associated with tuberculosis disease in Suratthani Central Prison, Thailand. *International Journal of Tuberculosis Lung Disease* 2018.

190. Johns Hopkins University Bloomberg School of Public Health. Integrated Behavioral & Biological Surveillance (IBBS) in Afghanistan: Year 1 Report. *Johns Hopkins University Bloomberg School of Public Health* 2011.

191. Johns Hopkins University Bloomberg School of Public Health. Integrated Biological & Behavioral Surveillance (IBBS) in Selected Cities of Afghanistan Findings of 2012 IBBS survey and comparison to 2009 IBBS survey. *National AIDS Control Program (NACP) Ministry of Public Health, Afghanistan* 2012.

192. Choudhury R, Singh N. Prevalence of HIV/AIDS in inmates of two district jails of central Uttar Pradesh, India. *Medico-Legal Update* 2016.

193. Jha S, Kant S, Thakur N, et al. Prevalence of HIV among inmates in four states of North India: Findings from the 16th round of HIV sentinel surveillance. *International Journal of Prisoner Health* 2023; **19**(4): 699-708.

194. National AIDS Control Organization. HIV Sentinel Surveillance Plus 2021, Central Prison Sites, 2022.

195. Organization NAC. HIV Sentinel Surveillance Plus 2019, Central Prison Sites, 2019.

196. Ramamoorthy M, Venketeswaran A, Seenivasan P, et al. Risk factors and prevalence, hepatitis B virus and hepatitis C virus among prison inmates, Chennai, India, 2015. 2016; **53**: 90.

197. Kosambiya JK, Vadgama P, Samudyatha UC, Rathod D, Buch R, Damor R. Active case finding of pulmonary tuberculosis and HIV infection among prisoners of South Gujarat: A cross sectional study. *Indian Journal of Tuberculosis* 2022.

198. Sabharwal ER, Mathur DR, Mehta P. HIV seroprevalence among prison inmates in Rajasthan, India. *Journal of Clinical and Diagnostic Research* 2012.

199. Tyagi SK, Sovani V, Dias NP, Tyagi D, Saxena S. Prevalence and risk factors of HCV infection in a prison setting in Uttar Pradesh, India. *Indian Journal of Public Health Research and Development* 2018.

200. Kaur K, Grover GS, Boora PK, et al. Feasibility and effectiveness of hepatitis C micro-elimination among 19 prisons of northern India. *Journal of Hepatology* 2022.

201. Singh V, Kaur A, Kumari S, et al. Seroprevalence of HCV and Transient Elastography in a Correctional Setting. Hepatology; 2017: WILEY 111 RIVER ST, HOBOKEN 07030-5774, NJ USA; 2017. p. 558A-A.

202. National HIV bio­behavioral Surveillance Survey (BSS) in prisoners. 2009.

203. Ataei B, Khorvash F, Azadeh S, Nokhodian Z, Kassaian N, Babak A. The prevalence of high risk behaviors among women prisoners in Isfahan, Iran. *Journal of Isfahan Medical School* 2011.

204. Darbandi M, McFarland W, Mehmandoost S, et al. History of injection drug use in the month preceding incarceration in Iranian prisons. *Scientific Reports* 2025; **15**(1): 17469.

205. Hariri S, Sharafkhah M, Alavi M, et al. A simple risk-based strategy for hepatitis C virus screening among incarcerated people in a low- to middle-income setting. *Harm Reduction Journal* 2020.

206. Hariri S, Alavi M, Roshandel G, et al. An intervention to increase hepatitis C virus diagnosis and treatment uptake among people in custody in Iran. *International Journal of Drug Policy* 2021.

207. Khajedaluee M, Babaei A, Vakili R, et al. Sero-prevalence of bloodborne tumor viruses (HCV, HBV, HTLV-I and KSHV infections) and related risk factors among prisoners in Razavi Khorasan province, Iran, in 2008. *Hepatitis Monthly* 2016.

208. Khajehkazemi R, Haghdoost A, Navadeh S, et al. Risk and vulnerability of key populations to HIV infection in Iran; Knowledge, attitude and practises of female sex workers, prison inmates and people who inject drugs. *Sexual Health* 2014.

209. Khezri M, Sharifi H, Mirzazadeh A, et al. A National Study of Suicidal Ideation and Suicide Attempt Among Incarcerated People in Iran. *International Journal of Mental Health and Addiction* 2023.

210. Mamani M, Mahmudian H, Majzoobi MM, Poorolajal J. Prevalence and incidence rates of latent tuberculous infection in a large prison in Iran. *International Journal of Tuberculosis and Lung Dis* 2016.

211. Metanat M, Almasi SZ, Sepehri Rad N, Tabatabaee SM, Rezaei K. Seroepidemiological Investigation of Hepatitis B and C Prevalence and Associated Factors Among People in Custody at Zahedan Central Prison. *Archives of Iranian Medicine* 2024; **27**(6): 298-304.

212. Mirzazadeh A, Shokoohi M, Navadeh S, et al. Underreporting in HIV-Related High-Risk Behaviors: Comparing the Results of Multiple Data Collection Methods in a Behavioral Survey of Prisoners in Iran. *Prison Journal* 2018.

213. Mohtasham-Amiri Z, Rezvani SM, Ashoori F, Behboodi M, Toosi H, Jafari-Shakib R. Seroprevalence of Hepatitis C Virus among Prisoners in Lakan Prison, North of Iran, Is There Still a Concern? *Archives of Iranian Medicine* 2021.

214. Moradi G, Darvishi S, Asaadi L, et al. Patterns of Drug Use and Related Factors Among Prisoners in Iran: Results from the National Survey in 2015. *Journal of Primary Prevention* 2020.

215. Moradi G, Gouya MM, Zavareh FA, et al. Prevalence and risk factors for HBV and HCV in prisoners in Iran: a national bio-behavioural surveillance survey in 2015. *Tropical Medicine & International Health* 2018.

216. Moradi G, Jafari S, Zarei B, et al. Prevalence and Risk Factors for Hepatitis B and Hepatitis C Exposure in Iranian Prisoners: A National Study in 2016. *Hepatitis Monthly* 2019.

217. Navadeh S, Mirzazadeh A, Gouya MM, Farnia M, Alasvand R, Haghdoost AA. HIV prevalence and related risk behaviours among prisoners in Iran: results of the national biobehavioural survey, 2009. *Sexually Transmitted Infections* 2013.

218. Nokhodian Z, Yazdani MR, Yaran M, et al. Prevalence and risk factors of HIV, syphilis, hepatitis B and C among female prisoners in Isfahan, Iran. *Hepatitis Monthly* 2012.

219. Seyedalinaghi SA, Farhoudi B, Mohraz M, et al. Prevalence and Associated Factors of HIV Infection among Male Prisoners in Tehran, Iran. *Archives of Iranian Medicine* 2017.

220. SeyedAlinaghi S, Farhoudi B, Shahmohamadi E, et al. Prevalence of and risk factors for HCV among incarcerated people at Great Tehran Prison: a cross-sectional study. *International Journal of Prisoner Health* 2023.

221. Shahesmaeili A, Karamouzian M, Tavakoli F, et al. HIV prevalence and continuum of care among incarcerated people in Iran from 2010 to 2017. *Harm Reduction Journal* 2022.

222. Shahesmaeili A, Karamouzian M, Tavakoli F, et al. HIV prevalence and continuum of care among incarcerated people in Iran from 2010 to 2017. *Harm Reduction Journal* 2022; **19**(1).

223. Zamani S, Farnia M, Torknejad A, et al. Patterns of drug use and HIV-related risk behaviors among incarcerated people in a prison in Iran. *Journal of Urban Health* 2010.

224. Haghdoost AA, Mirzazadeh A, Shokoohi M, Sedaghat A, Gouya MM. HIV trend among Iranian prisoners in 1990s and 2000s; analysis of aggregated data from HIV sentinel sero-surveys. *Harm Reduction Journal* 2013.

225. Javadi A, Pourahmad M, Ataei B. The relationship between frequency and duration of imprisonment and the prevalence of HBsAg, AntiHCV and HIV antibody seropositivity in Iranian prisoners. 2006.

226. Khademi N, Shakiba E, Khodadost M, Khoramdad M. Seroprevalence and related risk behaviors of hepatitis C, hepatitis B and HIV infections among Male prisoners in Kermanshah, Iran. *Archives of Iranian Medicine* 2019.

227. Rafiee M, Karamouzian M, Sharifi M, et al. Non-injection drug use among incarcerated people in Iran: Findings from three consecutive national bio-behavioral surveys. *Harm Reduction Journal* 2024; **21**(1): 147.

228. Shahbazi M, Farnia M, Rahmani K, Moradi G. Trend of HIV/AIDS Prevalence and Related Interventions Administered in Prisons of Iran -13 Years' Experience. *Iranian Journal of Public Health* 2014.

229. Ziaee M, Sharifzadeh G, Namaee MH, Fereidouni M. Prevalence of HIV and Hepatitis B, C, D Infections and Their Associated Risk Factors among Prisoners in Southern Khorasan Province, Iran. *Iranian Journal of Public Health* 2014.

230. Hariri S, Sharafi H, Sheikh M, et al. Continuum of hepatitis C care cascade in prison and following release in the direct-acting antivirals era. *Harm Reduction Journal* 2020.

231. Moradi G, Alavian SM, Gholami F, et al. Prevalence of hepatitis B and hepatitis C infections among incarcerated individuals in Iran: A cross-sectional national bio-behavioral study in 2019. *Pathogens* 2021.

232. Sarkari B, Eilami O, Khosravani A, Sharifi A, Tabatabaee M, Fararouei M. High prevalence of hepatitis C infection among high risk groups in Kohgiloyeh and Boyerahmad Province, Southwest Iran. *Archives of Iranian Medicine* 2012.

233. Sharafi H, Poustchi H, Azimian F, et al. Performance of a rapid diagnostic test for screening of hepatitis C in a real-life prison setting. *Journal of Clinical Virology* 2019.

234. Shrestha G, Mulmi R, Yadav DK, et al. Health needs and risky behaviours among inmates in the largest prison of eastern Nepal. *International Journal of Prison Health* 2018.

235. Shrestha G. Prevalence of hypertension among incarcerated males of jhumka regional prison, Eastern Nepal. *Journal of Hypertension* 2018.

236. Butt A, Jafri W, Janjua N, Pasha O. Seroprevalence and risk factors for hepatitis C infection among male prisoners in Karachi, Pakistan. *American Journal of Gastroenterology* 2010.

237. Kazi AM, Shah SA, Jenkins CA, Shepherd BE, Vermund SH. Risk factors and prevalence of tuberculosis, human immunodeficiency virus, syphilis, hepatitis B virus, and hepatitis C virus among prisoners in Pakistan. *Int J Infect Dis* 2010.

238. Khan MD, Wali A, Fatima R, Yaqoob A, Aziz S. Prevalence and associated risk factors of HIV in prisons in Balochistan, Pakistan: A cross-sectional study. *F1000Research* 2019.

239. Memon AR, Shafique K, Memon A, Draz AU, Rauf MUA, Afsar S. Hepatitis B and C prevalence among the high risk groups of Pakistani population. A cross sectional study. *Archives of Public Health* 2012.

240. Nafees M, Qasim A, Jafferi G, Anwar MS, Muazzam M. HIV infection, HIV/HCV and HIV/HBV co-infections among jail inmates of Lahore. *Pakistan Journal of Medical Sciences* 2011.

241. Pervaiz A, Ghafoor T, Asghar RJ. Screening of prisoners for Human Immunodeficiency Virus (HIV), Hepatitis C (HCV) and B (HBV) in Punjab Province, Pakistan, 2009. *International Journal of Infectious Diseases* 2012.

242. Safdar S, Mehmood A, Abbas SQ. Prevalence of HIV/AIDS among jail inmates in Sindh. *J Pak Med Assoc* 2009.

243. Shah SSA, Ali M, Ahmad M, Hamadan U. Screening of jail inmates for HIV and tuberculosis. *Pakistan Journal of Medical and Health Sciences* 2013.

244. Wali A, Khan D, Safdar N, et al. Prevalence of tuberculosis, HIV/AIDS, and hepatitis; in a prison of Balochistan: a cross-sectional survey. *BMC Public Health* 2019.

245. Fayyaz M, Qazi M, Ishaq M, Chaudhry G, Bukhari MJB. Frequency of hepatitis B and C seropositivity in prisoners. 2006; **22**: 55-8.

246. Gorar ZA, Zulfikar I. Seropositivity of hepatitis C in prison inmates of Pakistan--a cross sectional study in prisons of Sindh. *Journal of the Pakistan Medical Association* 2010.

247. Khan MA, Ayub A, Ayub H, Shafique M, Rahman JA. A comparative study of Hepatitis B and C prevalence using ICT and elisa method in jail inmates. *Pakistan Journal of Medical and Health Sciences* 2017.

248. Niriella MA, Hapangama A, Luke H, Pathmeswaran A, Kuruppuarachchi K, de Silva HJ. Prevalence of hepatitis B and hepatitis C infections and their relationship to injectable drug use in a cohort of Sri Lankan prison inmates. *Ceylon Medical Journal* 2015.

249. Azbel L, Polonsky M, Wegman M, et al. Intersecting epidemics of HIV, HCV, and syphilis among soon-to-be released prisoners in Kyrgyzstan: Implications for prevention and treatment. *International Journal of Drug Policy* 2016.

250. Moller LF, van den Bergh BJ, Karymbaeva S, Esenamanova A, Muratalieva R. Drug use in prisons in Kyrgyzstan: a study about the effect of health promotion among prisoners. *International Journal of Prison Health* 2008.

251. Winetsky DE, Almukhamedov O, Pulatov D, Vezhnina N, Dooronbekova A, Zhussupov B. Prevalence, risk factors and social context of active pulmonary tuberculosis among prison inmates in Tajikistan. *PLoS One* 2014.

252. Ministry of Health and Social Protection The Republic of Tajikistan. Reference on the implementation of the National Epidemic Control Program human immunodeficiency syndrome in the Republic of Tajikistan for 2017 2020, 2019.

253. Boisson EV, Trotman C. HIV seroprevalence among male prison inmates in the six countries of the Organization of Eastern Caribbean states in the Caribbean (OECS). *West Indian Medical Journal* 2009.

254. Andrinopoulos K, Kerrigan D, Figueroa JP, et al. Establishment of an HIV/sexually transmitted disease programme and prevalence of infection among incarcerated men in Jamaica. *International Journal of STD & AIDS* 2010.

255. Pena-Orellana M, Hernandez-Viver A, Caraballo-Correa G, Albizu-Garcia CE. Prevalence of HCV risk behaviors among prison inmates: Tattooing and injection drug use. *Journal of Health Care for the Poor and Underserved* 2011.

256. Adaszko D, Sotelo J, Orlando M, Adaszko A, Angeleri P. HIV, hepatitis B and C, syphilis and Tuberculosis prevalence in people deprived of liberty for criminal reasons in Argentina. Final results of a national study. *International Journal Of Infectious Diseases* 2018; **73**: 202-.

257. Adaszko D, Sotelo JA, Orlando M, Angelerei P. Estudio de prevalencia de VIH, sífilis, hepatitis virales y tuberculosis en personas en contextos de encierro en unidades del Servicio Penitenciario Federal. *Buenos Aires, Ministerio de Salud* 2017.

258. Mendizabal M, Testa P, Rojas M, et al. Pilot study using the ECHO model to enhance linkage to care for patients with hepatitis C in the custodial setting. *Journal of Viral Hepatitis* 2020.

259. Villar G, Rolla L, Hadid L, et al. P-16 First Micro Elimination Intervention of Hepatitis B &C in Inmates of the Eight Prisons in the Province of Mendoza, Argentina. *Annals of Hepatology* 2023; **Conference: 2022 Annual Meeting of the ALEH. Buenos Aires Argentina. 28(Supplement 1) (no pagination)**.

260. Gough E, Edwards P. HIV seroprevalence and associated risk factors among male inmates at the Belize Central Prison. *Revista Panamericana de Salud Pública* 2009.

261. Lambert ML, Torrico F, Billot C, Mazina D, Marleen B, Van der Stuyft P. Street youths are the only high-risk group for HIV in a low-prevalence South American country. *Sexually Transmitted Diseases* 2005; **32**(4): 240-2.

262. Villarroel-Torrico M, Montaño K, Flores-Arispe P, et al. Syphilis, human immunodeficiency virus, herpes genital and hepatitis B in a women's prison in Cochabamba, Bolivia: prevalence and risk factors. *Revista Española de Sanidad Penitenciaria* 2018.

263. de Albuquerque ACC, da Silva DM, Rabelo DCC, et al. Seroprevalence and factors associated with human immunodeficiency virus (HIV) and syphilis in inmates in the state of Pernambuco, Brazil. *Ciencia & Saude Coletiva* 2014.

264. Castro LS, de Rezende GR, Puga MAM, et al. Hepatitis A virus infection in Brazilian correctional facilities. *PLoS One* 2023; **18**(4): e0283868.

265. Coelho HC, de Oliveira SAN, Miguel JC, et al. Predictive markers for hepatitis C virus infection among Brazilian inmates. *Revista da Sociedade Brasileira de Medicina Tropical* 2009.

266. El Maerrawi I, Carvalho HB. Prevalence and risk factors associated with HIV infection, hepatitis and syphilis in a state prison of São Paulo. *International Journal of STD & AIDS* 2015.

267. Falquetto TC, Endringer DC, Andrade TU, Lenz D. Hepatitis c in prisoners and non-prisoners in Colatina, Espirito santo, Brazil. *Brazilian Journal of Pharmaceutical Sciences* 2013.

268. Felisberto M, Saretto AA, Wopereis S, Machado MJ, Spada C. Prevalence of HCV infection in a prison population of the greater Florianopolis area. *Revista da Sociedade Brasileira de Medicina Tropical* 2019.

269. Felisberto M, Saretto AA, Wopereis S, Treitinger A, Machado MJ, Spada C. Prevalence of human immunodeficiency virus infection and associated risk factors among prison inmates in the city of Florianopolis. *Revista da Sociedade Brasileira de Medicina Tropical* 2016.

270. Guimarães T, Granato CF, Varella D, Ferraz ML, Castelo A, Kallás EG. High prevalence of hepatitis C infection in a Brazilian prison: identification of risk factors for infection. *Brazilain Journal of Infectious Diseases* 2001.

271. Lopes F, Latorre MR, Campos Pignatari AC, Buchalla CM. [HIV, HPV, and syphilis prevalence in a women's penitentiary in the city of São Paulo, 1997-1998]. *Cad Saude Publica* 2001.

272. Massad E, Rozman M, Azevedo R, et al. Seroprevalence of HIV, HCV and syphilis in Brazilian prisoners: preponderance of parenteral transmission. *European Journal of Epidemiology* 1999; **15**: 439-45.

273. Miranda AE, Vargas PM, St Louis ME, Viana MC. Sexually transmitted diseases among female prisoners in Brazil - Prevalence and risk factors. *Sexually Transmitted Diseases* 2000.

274. Okita MT, de Matos MA, de Freitas NR, et al. Human T-lymphotropic virus 1/2 infection among prisoners of a major penitentiary complex of Goiás State, Central-West Brazil. *Frontiers In Public Health* 2024; **12**.

275. Pompilio MA, Pontes ERJC, Castro ARCM, et al. Prevalence and epidemiology of chronic hepatitis c among prisoners of Mato Grosso do Sul State, Brazil. *Journal of Venomous Animals and Toxins Including Tropical Diseases* 2011.

276. Rosa F, Carneiro M, Duro LN, et al. Prevalence of anti-HCV in an inmate population. *Rev Assoc Med Bras (1992)* 2012.

277. Santos BFO, de Santana NO, Franca AVC. Prevalence, genotypes and factors associated with HCV infection among prisoners in Northeastern Brazil. *World Journal of Gastroenterology* 2011.

278. Strazza L, Azevedo RS, Carvalho HB, Massad E. The vulnerability of Brazilian female prisoners to HIV infection. *Brazilian Journal of Medical and Biology Research* 2004.

279. Strazza L, Massad E, Azevedo RS, Carvalho HB. Behavior associated with HIV and HCV infection in female prison inmates in Sao Paulo, Brazil. *Cadernos de Saude Publica* 2007.

280. de Navarro PD, de Almeida IN, Kritski AL, et al. Prevalence of latent Mycobacterium tuberculosis infection in prisoners. *Jornal Brasileiro De Pneumologia* 2016; **42**(5): 348-55.

281. Benedetti MSG, Nogami ASA, da Costa BB, et al. Sexually transmitted infections in women deprived of liberty in Roraima, Brazil. *Revista de Saude Publica* 2020.

282. Catalan-Soares BC, Almeida RT, Carneiro-Proietti AB. Prevalence of HIV-1/2, HTLV-I/II, hepatitis B virus (HBV), hepatitis C virus (HCV), Treponema pallidum and Trypanosoma cruzi among prison inmates at Manhuacu, Minas Gerais State, Brazil. *Revista da Sociedade Brasileira de Medicina Tropical* 2000.

283. Coelho HC, Perdoná GC, Neves FR, Passos AD. HIV prevalence and risk factors in a Brazilian penitentiary. *Cad Saude Publica* 2007.

284. Defante Ferreto LE, Guedes S, Braz Pauli F, et al. Seroprevalence and associated factors of HIV and Hepatitis C in Brazilian high-security prisons: A state-wide epidemiological study. *PLoS One* 2021.

285. Kerr L, Smith DG, Kendall C, et al. HIV testing inside Brazilian female prisons: results of a national survey. *AIDS Care* 2023; **35**(6): 841-9.

286. Leal M, Kerr L, Mota RMS, Neto RDP, Seal D, Kendall C. Health of female prisoners in Brazil. *Ciencia & Saude Coletiva* 2022.

287. Leite AGD, Damasceno LM, Conceicao SC, Motta PFC. Rapid tests for HIV, syphilis, and chronic hepatitis in a prison population in a prison complex in Salvador (BA), Brazil. *Ciencia & Saude Coletiva* 2022.

288. Machado F, Becker D, de Oliveira CF, Possuelo LG, Renner JDP. Seroprevalence of HIV, hepatitis B and C and syphilis infection in prisoners of the central region of Rio Grande do Sul, Brazil. *Mundo Da Saude* 2019.

289. Marins JR, Page-Shafer K, De Azevedo Barros MB, Hudes ES, Chen S, Hearst N. Seroprevalence and risk factors for HIV infection among incarcerated men in Sorocaba, Brazil. *AIDS and Behavior* 2000.

290. Miranda AE, Vargas, P.M., Louis, M.E.S. & Viana, M.C. Sexually transmitted diseases among female prisoners in Brazil: prevalence and risk factors. *Sexually Transmitted Diseases* 2000; **27**(9): 491-5.

291. Moura RJ, Romero GAS. HIV prevalence in recently incarcerated adult males in the Federal District, Brasilia, Brazil. *Revista da Sociedade Brasileira de Medicina Tropical* 2020.

292. Pelissari DM, Kuhleis DC, Bartholomay P, et al. Prevalence and screening of active tuberculosis in a prison in the South of Brazil. *International Journal of Tuberculosis and Lung Disease* 2018.

293. Pivetta de Araujo RC, Martinez L, da Silva Santos A, et al. Serial Mass Screening for Tuberculosis Among Incarcerated Persons in Brazil. *Clinical Infectious Diseases* 2024; **78**(6): 1669-76.

294. Prates Fonseca CE, Tupinambás U. Epidemiological profile of cases of HIV, Syphilis and Hepatitis in private of freedom, Minas Gerais. *Saúde Coletiva* 2023; **13**(88): 13381-8.

295. Prellwitz IM, Alves BM, Ikeda ML, et al. HIV behind bars: human immunodeficiency virus cluster analysis and drug resistance in a reference correctional unit from southern Brazil. *PLoS One* 2013.

296. Ronchi BR, Rios GM, Knoll RK, Cardoso C. Prevalence of HIV, sifilis, hepatitis B and hepatitis c in the inmates of the penitentiary complex of vale do Itajai-SC. *Sexually Transmitted Infections* 2017.

297. Sgarbi RVE, Carbone ADS, Paiao DSG, et al. A Cross-Sectional Survey of HIV Testing and Prevalence in Twelve Brazilian Correctional Facilities. *PLoS One* 2015.

298. Soares Epifania P, Santos Passos Costa J, Costa Barros KC, Santos de Freitas K, Sampaio Maciel G, da Silva Santos Passos S. Doenças infectocontagiosas em indivíduos privados de liberdade. *Enfermagem Brasil* 2022.

299. Sousa KAA, Araujo TME, Teles SA, Rangel EML, Nery IS. Factors associated with HIV prevalence in a prison population. *Revista da Escola de Enfermagem da U S P* 2017.

300. Vale EP, Carvalho LD, Pereira FCD. HIV seroprevalence in prisoners in Amapascritores. *Revista De Epidemiologia E Controle De Infeccao* 2016.

301. Valença MS, Scaini JL, Abileira FS, Gonçalves CV, von Groll A, Silva PE. Prevalence of tuberculosis in prisons: risk factors and molecular epidemiology. *International Journal of Tuberculosis and Lung Disease* 2015.

302. de Andrade FM, de Amorim Andrade SG, Araujo Júnior E, et al. Pap smear and colposcopy findings in female inmates of a prison unit in the state of São Paulo, Brazil. *Ceska Gynekol* 2024; **89**(6): 459-68.

303. do Nascimento CT, Pena DZ, Giuffrida R, et al. Prevalence and epidemiological characteristics of inmates diagnosed with infectious diseases living in a region with a high number of prisons in Sao Paulo state, Brazil. *BMJ Open* 2020.

304. dos Santos Bet GM, de Souza GHdA, Croda J, et al. Treatment outcomes of brazilian inmates with treponema pallidum and human immunodeficiency virus infection: A prospective cohort study. *The American Journal of Tropical Medicine and Hygiene* 2018; **98**(6): 1603.

305. Barros LAS, Pessoni GC, Teles SA, et al. Epidemiology of the viral hepatitis B and C in female prisoners of Metropolitan Regional Prison Complex in the State of Goias, Central Brazil. *Revista da Sociedade Brasileira de Medicina Tropical* 2013.

306. Emerim E, Cezar VDL, Ferreira EMP, et al. P-23 Test and Treat: Profile of Patients Diagnosed with Hepatitis C in the Prison System of Porto Alegre, Brazil. *Annals of Hepatology* 2023; **Conference: 2022 Annual Meeting of the ALEH. Buenos Aires Argentina. 28(Supplement 1) (no pagination)**.

307. Gonçalves KJGUCdG. História de vida e situação de saúde no ambiente prisional de Goiás: estudo da prevalência de hepatite C em detentos [dissertação]. 2005.

308. Pinheiro DM, da Silva Souza AT, Alencar DdC, et al. PREVALENCIA DE ANTI-HCV E FATORES ASSOCIADOS EM DETENTOS DE UNIDADES PRISIONAIS. *Enfermagem em Foco* 2024; **15**: S50-S7.

309. Puga MAM, Bandeira LM, Pompilio MA, et al. Prevalence and Incidence of HCV Infection among Prisoners in Central Brazil. *PLoS One* 2017.

310. Santos Barros LA, Carolina Pessoni G, Araujo Teles S, et al. Epidemiology of the viral hepatitis B and C in female prisoners of metropolitan regional prison complex in the State of Goias, Central Brazil. *Revista da Sociedade Brasileira de Medicina Tropical* 2013.

311. da Rosa F, Carneiro M, Duro LN, et al. Prevalence of anti-HCV in an inmate population. *Revista da Associacao Medica Brasileira* 2012.

312. da Silva JB, Soares E, Munhoz A, et al. P- 43 HCV TESTING AND TREATMENT IN FOUR BRAZILIANS' CORRECTIONAL SETTINGS. *Annals of Hepatology* 2024; **29**(Supplement 1): 101230.

313. Castillo RL, Noriega KJR, Briceno ML, Munoz NG, Pacheco JR. CONSUMPTION OF PSYCHOACTIVE SUBSTANCES BEFORE AND AFTER ADMISSION TO PRISON IN FOURTEEN PRISONS IN COLOMBIA. *REVISTA COLOMBIANA DE CIENCIAS SOCIALES* 2017.

314. Sanchez-Vanegas G, Rodriguez-Vallejo D, Pinzon-Duran AC, Reina-Cifuentes MA, Monterrosa-Blanco A, Tiga-Segura JA. Prevalence of syphilis, hepatitis B and human immunodeficiency virus in the male prison population in Bogota, Colombia in 2019. [Spanish]. *Infectio* 2020.

315. Alvarez Rodriguez BE, Pinzon Z, Huaman BJ, et al. Prevalence of HIV, syphilis, drugs use and sexual risk behaviours among prisoners in Guatemala, 2012. *Sexually Transmitted Infections Conference: STI and AIDS World Congress* 2013.

316. Alvarado-Esquivel C, Sablon E, Martínez-García S, Estrada-Martínez S. Hepatitis virus and HIV infections in inmates of a state correctional facility in Mexico. *Epidemiology & Infection* 2005.

317. Bautista-Arredondo S, González A, Servan-Mori E, et al. A Cross-Sectional Study of Prisoners in Mexico City Comparing Prevalence of Transmissible Infections and Chronic Diseases with That in the General Population. *PLoS One* 2015.

318. Belaunzaran-Zamudio PF, Mosqueda-Gomez JL, Macias-Hernandez A, Sierra-Madero JG, Ahmed S, Beyrer C. Risk factors for prevalent hepatitis C virus-infection among inmates in a state prison system in Mexico. *PLoS One* 2017.

319. Gonzalez CAM, Ortiz BES, Aguilar MB, Gonzalez JDM. Risk factors and the seroprevalence of viral markers of hepatitis B (HVB) and hepatitis C (HCV) in high-risk groups in Chiapas. *Medwave* 2011.

320. Belaunzaran-Zamudio PF, Mosqueda-Gomez JL, Macias-Hernandez A, Rodríguez-Ramírez S, Sierra-Madero J, Beyrer C. Burden of HIV, Syphilis, and Hepatitis B and C Among Inmates in a Prison State System in Mexico. *AIDS Res Hum Retroviruses* 2017.

321. Silverman-Retana O, Servan-Mori E, McCoy SI, Larney S, Bautista-Arredondo S. Hepatitis C antibody prevalence among Mexico City prisoners injecting legal and illegal substances. *Drug and Alcohol Dependence* 2017.

322. Bautista-Arredondo S. Prevalence of transmissible infections and socio-demographic and behavioral risk factors amongst prisoners in Mexico City: A cross-sectional study of 17,296 inmates. *Journal of the International AIDS Society* 2012.

323. Flores VMF, Carreon OP, Gomez AF, Gallo AM, Garmendia AO, Marquez M. P-10 Seroprevalence of Chronic Hepatitis C Infection and Virological Cure with a Daily Administration System of Ns3/4a Protease Inhibitor and Ns5a Inhibitor in Imprisoned Patients. *Annals of Hepatology* 2024; **Conference: 2023 Annual Meeting of the ALEH. Bagota Colombia. 29(Supplement 3) (no pagination)**.

324. Terrones MTG, Acosta IMP, Centeno MA. P-58 Prevalence of Chronic Viral Hepatitis Type C in People Deprived of Their Liberty in the Ceresos of the State of Veracruz, Mexico: Towards Micro-Elimination. *Annals of Hepatology* 2023; **Conference: 2022 Annual Meeting of the ALEH. Buenos Aires Argentina. 28(Supplement 1) (no pagination)**.

325. Cyrus E, Sanchez J, Madhivanan P, et al. Prevalence of Intimate Partner Violence, Substance Use Disorders and Depression among Incarcerated Women in Lima, Perú. *International Journal of Environmental Research Public Health* 2021.

326. Garaycochea MC, Pino R, Chavez I, et al. Sexually transmitted infections in women living in a prison in Lima, Peru. *Revista Peruana de Medicina Experimental y Salud Publica* 2013.

327. Troya MM, Vila RB. HIV infection and associated risk behaviours in a prison in Montevideo, Uruguay. *Revista Española de Sanidad Penitenciaria* 2010; **12**(1): 21-8.

328. Monsalve-Castillo F, Chacín-Bonilla L, Atencio RJ, et al. Low prevalence of hepatitis C virus infection in a prisoner population from Maracaibo, Venezuela. *Biomedica* 2009.

329. Alcivar JC, Zambrano MM, Madronero MG, et al. Sexually transmitted infections in inmates in Merida Venezuela. *Investigacion Clinica* 2020.

330. Gil YMF. Estudio de prevalencia de VIH en población de Personas Privada de Libertad en 30 establecimientos penitenciarios de la República Bolivariana de Venezuela, 2022. In: Salud MdPPpl, editor.; 2022.

331. Posada A, Díaz Tremarias M. [HIV, hepatitis B and syphilis infection in inmates of Venezuela's prisons, 1998-2001]. *Revista Española de Sanidad Penitenciaria* 2008; **10**(3): 73-9.

332. Besney JD, Angel C, Pyne D, Martell R, Keenan L, Ahmed R. Addressing Women’s Unmet Health Care Needs in a Canadian Remand Center. *Journal of Correctional Health Care* 2018.

333. Bonnycastle KD, Villebrun C. Injecting Risk Into Prison Sentences: A Quantitative Analysis of a Prisoner-Driven Survey to Measure HCV/HIV Seroprevalence, Risk Practices, and Viral Testing at One Canadian Male Federal Prison. *Prison Journal* 2011.

334. Calzavara L, Ramuscak N, Burchell AN, et al. Prevalence of HIV and hepatitis C virus infections among inmates of Ontario remand facilities. *CMAJ* 2007.

335. Calzavara LM, Burchell AN, Schlossberg J, et al. Prior opiate injection and incarceration history predict injection drug use among inmates. *Addiction* 2003.

336. Courtemanche Y, Poulin C, Serhir B, Alary M. HIV and hepatitis C virus infections in Quebec's provincial detention centres: comparing prevalence and related risky behaviours between 2003 and 2014-2015. *Canadian Journal of Public Health* 2018.

337. Dussault C, Cox J, Klein M, Sebastiani G, Lebouche B, Kronfli N. Factors associated with on-demand HCV screening among Canadian provincial inmates. *Canadian Liver Journal* 2020.

338. Martin RE, Remple V, Gold F, Berkowitz J, Murphy W, Money D. Drug use and risk of bloodborne infections: A survey of female prisoners in British Columbia. *Canadian Journal of Public Health-Revue Canadienne De Sante Publique* 2005.

339. Nolan AM, Stewart LA. Chronic Health Conditions Among Incoming Canadian Federally Sentenced Women. *Journal of Correctional Health Care* 2017.

340. Poulin C, Alary M, Lambert G, et al. Prevalence of HIV and hepatitis C virus infections among inmates of Quebec provincial prisons. [References]. *Canadian Medical Association Journal* 2007.

341. Ford PM, Pearson M, Sankar-Mistry P, Stevenson T, Bell D, Austin J. HIV, hepatitis C and risk behaviour in a Canadian medium-security federal penitentiary. Queen's University HIV Prison Study Group. *QJM* 2000.

342. Poulin C, Alary M, Lambert G, et al. Prevalence of HIV and hepatitis C virus infections among inmates of Quebec provincial prisons. *CMAJ* 2007.

343. Whitten C, Turner A, Howell B, Sparkes B, Ricciardelli R, Daley P. Retrospective review of rates of sexually transmitted and blood-borne infection (STBBI) testing in provincial corrections facilities in Newfoundland and Labrador. *JAMMI: Journal of the Association of Medical Microbiology & Infectious Disease Canada* 2023; **8**(2): 141-9.

344. Bartlett S, Yu A, Young P, et al. The first provincial correctional system-wide hepatitis C care cascade in Canada: Monitoring hepatitis C care in British Columbia provincial correctional centres. *Canadian Liver Journal* 2023; **6(1)**: 90-2.

345. Kronfli N, Mambro A, Dussault C, et al. Optimizing linkage to hepatitis C virus (HCV) care for untreated individuals released from Quebec provincial prison: Interim analysis of the beyond prison walls study. *Canadian Liver Journal* 2023; **6(1)**: 85.

346. Kronfli N, Dussault C, Klein MB, Lebouche B, Sebastiani G, Cox J. The hepatitis C virus cascade of care in a Quebec provincial prison: a retrospective cohort study. *CMAJ Open* 2019.

347. Abiona TC, Adefuye AS, Balogun JA, Sloan PE. Gender differences in HIV risk behaviors of inmates. *Journal of Womens Health (Larchmt)* 2009.

348. Abiona TC, Balogun JA, Adefuye AS, Sloan PE. Pre-incarceration HIV risk behaviours of male and female inmates. [References]. *International Journal of Prisoner Health* 2009.

349. Adams LM, Kendall S, Smith A, Quigley E, Stuewig JB, Tangney JP. HIV risk behaviors of male and female jail inmates prior to incarceration and one year post-release. *AIDS Behaviors* 2013.

350. Akiyama MJ, Kaba F, Rosner Z, et al. Correlates of hepatitis C virus infection in the targeted testing program of the New York city jail system: Epidemiologic patterns and priorities for action. *Public Health Reports* 2017.

351. Altice FL, Marinovich A, Khoshnood K, Blankenship KM, Springer SA, Selwyn PA. Correlates of HIV infection among incarcerated women: implications for improving detection of HIV infection. *Journal of Urban Health* 2005.

352. Alvarez KJ, Befus M, Herzig CTA, Larson E. Prevalence and correlates of hepatitis C virus infection among inmates at two New York State correctional facilities. *Journal of Infection and Public Health* 2014.

353. Arndt S, Turvey CL, Flaum M. Older offenders, substance abuse, and treatment. *American Journal of Geriatric Psychiatry* 2002.

354. Beckwith CG, Liu T, Bazerman LB, et al. HIV risk behavior before and after HIV counseling and testing in jail: a pilot study. *Journal of Acquired Immune Deficiency Syndromes* 2010.

355. Beckwith CG, Atunah-Jay S, Cohen J, et al. Feasibility and Acceptability of Rapid HIV Testing in Jail. [References]. *AIDS Patient Care and STDs* 2007.

356. Begier EM, Bennani Y, Forgione L, et al. Undiagnosed HIV infection among New York City jail entrants, 2006: results of a blinded serosurvey. *Journal of Acquired Immune Deficiency Syndromes* 2010.

357. Brinkley-Rubinstein L, Crowley C, Montgomery MC, et al. Interest and Knowledge of HIV Pre-Exposure Prophylaxis in a Unified Jail and Prison Setting. *Journal of Correctional Health Care* 2020.

358. Deb LC, Hove H, Miller TK, et al. Epidemiology of Hepatitis C virus infection among incarcerated populations in North Dakota. *PLoS One* 2022.

359. Fox RK, Currie SL, Evans J, et al. Hepatitis C virus infection among prisoners in the California State correctional system. *Clinical Infectious Diseases* 2005.

360. Gates ML, Turney A, Ferguson E, Walker V, Staples-Horne M. Associations among Substance Use, Mental Health Disorders, and Self-Harm in a Prison Population: Examining Group Risk for Suicide Attempt. *International Journal of Environmental Research and Public Health* 2017.

361. Harrison LD, Bachman T, Freeman C, Inciardi JA. The acceptability of the female condom among US women at high risk from HIV. [References]. *Culture, Health & Sexuality* 2001.

362. Katyal M, Leibowitz R, Venters H. IGRA-Based Screening for Latent Tuberculosis Infection in Persons Newly Incarcerated in New York City Jails. *Journal of Correctional Health Care* 2018.

363. Keleekai NL. Patterns and predictors of HIV, sexually transmitted infections, and staphylococcus aureus co-infection among New York state prison inmates. *Dissertation Abstracts International: Section B: The Sciences and Engineering* 2012.

364. Kendrick SR, Kroc KA, Couture E, Weinstein RA. Comparison of point-of-care rapid HIV testing in three clinical venues. *Aids* 2004.

365. Khan AJ, Simard EP, Bower WA, et al. Ongoing transmission of hepatitis B virus infection among inmates at a state correctional facility. *American Journal of Public Health* 2005.

366. Kim AY, Nagami EH, Birch CE, Bowen MJ, Lauer GM, McGovern BH. A simple strategy to identify acute hepatitis C virus infection among newly incarcerated injection drug users. *Hepatology* 2013.

367. Lally M, Gaitanis M, Vallabhaneni S, et al. Willingness to receive an HIV vaccine among incarcerated persons. *Preventive Medicine* 2006.

368. MacGowan R, Margolis A, Richardson-Moore A, et al. Voluntary Rapid Human Immunodeficiency Virus (HIV) Testing in Jails. *Sexually Transmitted Diseases* 2009.

369. Macalino GE, Dhawan D, Rich JD. A missed opportunity: hepatitis C screening of prisoners. *American Journal of Public Health* 2005.

370. McClelland GM, Teplin LA, Abram KM, Jacobs N. HIV and AIDS risk behaviors among female jail detainees: Implications for public heath policy. [References]. *American Journal of Public Health* 2002.

371. Mullings JL, Marquart JW, Hartley DJ. Exploring the effects of childhood sexual abuse and its impact on HIV/AIDS risk-taking behavior among women prisoners. *Prison Journal* 2003.

372. Mullings JL, Marquart JW, Diamond PM. Cumulative continuity and injection drug use among women: A test of the downward spiral framework. [References]. *Deviant Behavior* 2001.

373. Nijhawan AE, Iroh PA, Brown LS, Winetsky D, Porsa E. Cost analysis of tuberculin skin test and the QuantiFERON-TB Gold In-tube test for tuberculosis screening in a correctional setting in Dallas, Texas, USA. *BMC Infectious Diseases* 2016.

374. Rice DK. Design, implementation, and evaluation of a jail-based HIV screening program. *Dissertation Abstracts International: Section B: The Sciences and Engineering* 2011.

375. Simonsen KA, Shaikh RA, Earley M, et al. Rapid HIV Screening in an Urban Jail: How Testing at Exit With Linkage to Community Care Can Address Perceived Barriers. *Journal of Primary Prevention* 2015.

376. Swartz JA, Lurigio AJ, Weiner DA. CORRELATES OF HIV-RISK BEHAVIORS AMONG PRISON INMATES: IMPLICATIONS FOR TAILORED AIDS PREVENTION PROGRAMMING. *Prison Journal* 2004.

377. Tartaro C, Levy MP. An Evaluation of an HIV Testing Program in the Jail Setting: Results and Recommendations. *Prison Journal* 2013.

378. Trevino S. The relationship between age of first reported trauma and substance specific use in incarcerated women. *Dissertation Abstracts International: Section B: The Sciences and Engineering* 2013.

379. Wenger PJ, Rottnek F, Parker T, Crippin JS. Assessment of hepatitis C risk factors and infection prevalence in a jail population. *American Journal of Public Health* 2014; **104**(9): 1722-7.

380. Arriola KR, Braithwaite RL, Kennedy S, et al. A collaborative effort to enhance HIV/STI screening in five county jails. *Public Health Reports* 2001.

381. Baillargeon J, Wu H, Kelley MJ, Grady J, Linthicum L, Dunn K. Hepatitis C seroprevalence among newly incarcerated inmates in the Texas correctional system. *Public Health* 2003.

382. Baillargeon J, Pulvino JS, Leonardson JE, et al. The changing epidemiology of HIV in the criminal justice system. *International Journal of STD & AIDS* 2017.

383. Baillargeon J, Black SA, Pulvino J, Dunn K. The disease profile of Texas prison inmates. *Ann Epidemiol* 2000.

384. Baillargeon JG, Paar DP, Wu H, et al. Psychiatric disorders, HIV infection and HIV/hepatitis co-infection in the correctional setting. [References]. *AIDS Care* 2008.

385. Bauserman RL, Ward MA, Eldred L, Swetz A. Increasing voluntary HIV testing by offering oral tests in incarcerated populations. *American Journal of Public Health* 2001.

386. Beckwith CG, Bazerman L, Cornwall AH, et al. An evaluation of a routine opt-out rapid HIV testing program in a Rhode Island jail. *AIDS Education and Prevention* 2011.

387. Beckwith CG, Nunn A, Baucom S, et al. Rapid HIV testing in large urban jails. *Am J Public Health* 2012.

388. Carvajal RI, Ross MW, Byrd T, Shelton A. HIV Counseling and Testing Program for Female Inmates: Analysis of Data From the Harris County Jail. [References]. *Journal of Correctional Health Care* 2005.

389. Chin ET, Leidner D, Zhang Y, et al. Effectiveness of Coronavirus Disease 2019 (COVID-19) Vaccines Among Incarcerated People in California State Prisons: Retrospective Cohort Study. *Clinical Infectious Diseases* 2022.

390. Chin ET, Ryckman T, Prince L, et al. COVID-19 in the California State Prison System: an Observational Study of Decarceration, Ongoing Risks, and Risk Factors. *Journal of General Internal Medicine* 2021.

391. Cocoros N, Nettle E, Church D, et al. Screening for hepatitis C as a prevention enhancement (SHAPE) for HIV: An integration pilot initiative in a massachusetts county correctional facility. *Public Health Reports* 2014.

392. Desai J, Nijhawan A, Krakower D, Harris BL, Taherzadeh D. Hiv/sti testing and prep eligibility among women incarcerated in an urban county jail. *Topics in Antiviral Medicine* 2021.

393. Feld S, Steele J, Klinedinst S, et al. Implementing Opt-Out HIV Testing in the Alameda County Jails. *Journal of correctional health care : the official journal of the National Commission on Correctional Health Care* 2023.

394. Irvin R, Landry G, Jones MR, et al. High prevalence of hepatitis C virus infection among incarcerated persons: Results from the Louisiana Hepatitis C Elimination Plan's opt-out testing program in prisons. *Journal of Viral Hepatitis* 2024; **31**(7): 432-5.

395. Javanbakht M, Boudov M, Anderson LJ, et al. Sexually transmitted infections among incarcerated women: Findings from a decade of screening in a Los Angeles County jail, 2002-2012. [References]. *American Journal of Public Health* 2014.

396. Kavasery R, Maru DS, Sylla LN, Smith D, Altice FL. A prospective controlled trial of routine opt-out HIV testing in a men's jail. *PLoS One* 2009.

397. Kavasery R, Maru DS, Cornman-Homonoff J, Sylla LN, Smith D, Altice FL. Routine opt-out HIV testing strategies in a female jail setting: a prospective controlled trial. *PLoS One* 2009.

398. Klein SJ, O'Connell DA, Devore BS, Wright LN, Birkhead GS. Building an HIV continuum for inmates: New York state's criminal justice initiative. [References]. *AIDS Education and Prevention* 2002.

399. Krebs CP. INMATE FACTORS ASSOCIATED WITH HIV TRANSMISSION IN PRISON. *Criminology & Public Policy* 2006.

400. Leukefeld CG, Staton M, Hiller ML, et al. A descriptive profile of health problems, health services utilization, and HIV serostatus among incarcerated male drug abusers. *Journal of Behavioral Health Services & Research* 2002.

401. Lucas KD, Eckert V, Behrends CN, Wheeler C, MacGowan RJ, Mohle-Boetani JC. Evaluation of Routine HIV Opt-Out Screening and Continuum of Care Services Following Entry into Eight Prison Reception Centers--California, 2012. *Morbidity and Mortality Weekly Report* 2016.

402. Macalino GE, Vlahov D, Sanford-Colby S, et al. Prevalence and Incidence of HIV, Hepatitis B Virus, and Hepatitis C Virus Infections Among Males in Rhode Island Prisons. [References]. *American Journal of Public Health* 2004.

403. Maruschak LM. HIV in Prisons, 2021 – Statistical Tables: Bureau of Justice Statistics, 2023.

404. Peter P. Impact of Opt-Out and Opt-in HIV testing and education program on discovering HIV in jail populations. *Dissertation Abstracts International: Section B: The Sciences and Engineering* 2013.

405. Rosen DL, Wohl DA, Golin CE, et al. Comparing HIV Case Detection in Prison During Opt-In vs. Opt-Out Testing Policies. *Journal of Acquired Immune Deficiency Syndromes* 2016.

406. Rosen DL, Schoenbach VJ, Wohl DA, White BL, Stewart PW, Golin CE. Characteristics and behaviors associated with HIV infection among inmates in the North Carolina prison system. *American Journal of Public Health* 2009.

407. Rowell-Cunsolo TL, Szeto B, Sampong SA, Larson EL. Predictors of sexual behaviour among men and women in New York City area prisons. *Culture Health & Sexuality* 2016.

408. Ruiz JD, Molitor F, Plagenhoef JA. Trends in hepatitis C and HIV infection among inmates entering prisons in California, 1994 versus 1999. *Aids* 2002.

409. Sampson LA. Screening for syphilis and HIV in North Carolina jails. *Dissertation Abstracts International: Section B: The Sciences and Engineering* 2009.

410. Scott J, Sampson LA, Clymore JM, Moore PR, Leone PA. Integrated HIV, syphilis, and other STI testing in North Carolina county jails. *Sexually Transmitted Infections* 2011.

411. Seth P, Figueroa A, Wang G, Reid L, Belcher L. HIV Testing, HIV Positivity, and Linkage and Referral Services in Correctional Facilities in the United States, 2009-2013. *Sexually Transmitted Diseases* 2015.

412. Sieck CJ, Dembe AE. Results of a pilot study of pre-release STD testing and inmates' risk behaviors in an Ohio prison. *Journal of Urban Health* 2011.

413. Simonson R, Koenigsberg B, Varela NG, et al. Jail Length of Stay Does Not Account for Gender Differences in Hepatitis C Treatment Initiation in the New York City Jail System, 2019-2023. *HEPATOLOGY* 2024; **80(Supplement 1)**: S1980-S1.

414. Smith L, Moncur BL, Anderson B, et al. HIV prevalence and care in the New York state department of corrections. *Topics in Antiviral Medicine* 2016.

415. Solomon L, Flynn C, Muck K, Vertefeuille J. Prevalence of HIV, syphilis, hepatitis B, and hepatitis C among entrants to Maryland correctional facilities. *Journal of Urban Health* 2004.

416. Spaulding AC, Kim MJ, Corpening KT, Carpenter T, Watlington P, Bowden CJ. Establishing an HIV Screening Program Led by Staff Nurses in a County Jail. *Journal of Public Health Management and Practice* 2015.

417. Spaulding AC, Seals RM, McCallum VA, Perez SD, Brzozowski AK, Steenland NK. Prisoner survival inside and outside of the institution: implications for health-care planning. *American Journal of Epidemiology* 2011.

418. Spaulding A, Booker C, Freeman S, et al. Jails, HIV Testing, and Linkage to Care Services: An Overview of the EnhanceLink Initiative. *AIDS & Behavior* 2013.

419. Strick LB, MacGowan, R.J., Margolis & Belcher, L. HIV screening of male inmates during prison intake medical evaluation--Washington, 2006-2010. *Morbidity and Mortality Weekly Report* 2011.

420. Taussig J. HIV Transmission Among Male Inmates in a State Prison System -- Georgia, 1992-2005. (cover story). *MMWR: Morbidity & Mortality Weekly Report* 2006.

421. Weant TE, Turner AN, Murphy-Weiss M, Murray DM, Wang SH. Can social history variables predict prison inmates' risk for latent tuberculosis infection? *Tuberculosis Research and Treatment* 2012; **2012**: 132406.

422. Wohl DA, Golin C, Rosen DL, May JM, White BL. Detection of undiagnosed HIV among state prison entrants. *Jama* 2013.

423. de Ravello L, Brantley MD, Lamarre M, Qayad MG, Aubert H, Beck-Sague C. Sexually transmitted infections and other health conditions of women entering prison in Georgia, 1998-1999. *Sexually Transmitted Diseases* 2005.

424. de Voux A, Spaulding AC, Beckwith C, et al. Early identification of HIV: empirical support for jail-based screening. *PLoS One* 2012; **7**(5): e37603.

425. de la Flor C, Porsa E, Nijhawan AE. Opt-out HIV and Hepatitis C Testing at the Dallas County Jail: Uptake, Prevalence, and Demographic Characteristics of Testers. *Public Health Reports* 2017.

426. Abe C, Porsa E, Nijhawan AE. Hepatitis C care cascade in jail: Implications for hard-to-reach populations. *Topics in Antiviral Medicine* 2018.

427. Abe CM, Aguwa M, Zhao M, Sullivan J, Porsa E, Nijhawan AE. Hepatitis C Virus Infection in the Dallas County Jail: Implications for Screening, Prevention, and Linkage to Care. *Public Health Reports* 2019.

428. Akiyama MJ, Kaba F, Rosner Z, Alper H, Holzman RS, MacDonald R. Hepatitis C Screening of the Birth Cohort (Born 1945-1965) and Younger Inmates of New York City Jails. *American Journal of Public Health* 2016.

429. Archer GRD, Deming P, Ceniceros JA, Tomedi LE, Selvage D, Thornton K. Changes in hepatitis C virus infections after implementation of an expanded treatment program in New Mexico state prisons. *International Journal Of Drug Policy* 2025; **142(no pagination)**.

430. Assoumou SA, Wang J, Tasillo A, et al. Hepatitis C Testing and Patient Characteristics in Washington State's Prisons Between 2012 and 2016. *American Journal of Preventive Medicine* 2019.

431. Bai JR, Mukherjee DV, Befus M, Apa Z, Lowy FD, Larson EL. Concordance between medical records and interview data in correctional facilities. *BMC Medical Research Methodology* 2014.

432. Baillargen J, Snyder N, Soloway RD, et al. Hepatocellular Carcinoma Prevalence and Mortality in a Male State Prison Population. *Public Health Reports* 2009.

433. Chan J, Kaba F, Schwartz J, et al. The hepatitis C virus care cascade in the New York City jail system during the direct acting antiviral treatment era, 2014-2017. *eClinicalMedicine* 2020.

434. Hoff E, Warden A, Taylor R, Nijhawan AE. Hepatitis C Epidemiology in a Large Urban Jail: A Changing Demographic. *Public Health Reports* 2023.

435. Kennedy BS, Richeson RP, Houde AJ. Hepatitis C Virus Care Cascade by Race/Ethnicity in a Statewide Correctional Population, 2019-2023. *Journal of Racial and Ethnic Health Disparities* 2024; **01**.

436. Kuncio DE, Newbern EC, Fernandez-Viña MH, Herdman B, Johnson CC, Viner KM. Comparison of risk-based hepatitis C screening and the true seroprevalence in an urban prison system. *Journal of Urban Health* 2015.

437. Larney S, Mahowald MK, Scharff N, Flanigan TP, Beckwith CG, Zaller ND. Epidemiology of hepatitis C virus in Pennsylvania state prisons, 2004-2012: limitations of 1945-1965 birth cohort screening in correctional settings. *American Journal of Public Health* 2014.

438. Leukefeld C, Harp KLH, Webster M, Staton-Tindall M, Oser CB, Havens JR. Examining HCV and other risks among rural women offenders. *Drug and Alcohol Dependence* 2015.

439. Lincoln T, Tuthill RW, DePietro SL. Viral hepatitis, risk behaviors, aminotransferase levels, and screening options at a county correctional center. *Journal of Correctional Health Care* 2006.

440. Lucas KD, Krawiec A, Wada J, Kanan RJ. The hepatitis C care cascade in California state prisons: Screening and treatment scale-up and progress toward elimination, 2016-2023. *Clinical Liver Disease* 2024; **23(1) (no pagination)**.

441. Magaldi LN, Trooskin S, Anderson J, et al. Routine Opt out Hepatitis C Testing Upon Intake in the Philadelphia Jail System. *Hepatology* 2022.

442. Nijhawan AE, Sullivan J, Aguwa M, Porsa E. Demographic trends in HCV diagnosis and linkage to HCV care among jail detainees. *Topics in Antiviral Medicine* 2019.

443. Phung J, Pham HM, Shin RB, Tsolova V, Patel MC, Chan J. Prevalence of hepatitis c virus in an incarcerated population. *Hepatology* 2021.

444. Spaulding AC, Chen J, Mackey CA, et al. Assessment and Comparison of Hepatitis C Viremia in the Prison Systems of New Mexico and Georgia. *JAMA Network Open* 2019.

445. Kinner SA, Winter R, Saxton K. A longitudinal study of health outcomes for people released from prison in Fiji: the HIP-Fiji project. *Australas Psychiatry* 2015.

446. Welfare AIoHa. The health of Australia’s prisoners 2018. Canberra: AIHW, 2019.

447. Bah R, Sheehan Y, Li X, et al. Prevalence of blood-borne virus infections and uptake of hepatitis C testing and treatment in Australian prisons: the AusHep study. *The Lancet Regional Health–Western Pacific* 2024; **53**.

448. Butler T, Levy M, Dolan K, Kaldor J. Drug use and its correlates in an Australian prisoner population. *Addiction Research & Theory* 2003.

449. Butler TS, M. National Prison Entrants’ Bloodborne Virus and Risk Behaviour Survey Report: Kirby Institute 2017.

450. Cumming C, Kinner SA, McKetin R, Young JT, Li I, Preen DB. Using the Alcohol, Smoking and Substance Involvement Screening Test to predict substance‐related hospitalisation after release from prison: A cohort study. *Addiction* 2024; **119**(2): 236-47.

451. Gilles M, Swingler E, Craven C, Larson A. Prison health and public health responses at a regional prison in Western Australia. *Australian and New Zealand Journal of Public Health* 2008.

452. Hajarizadeh B, Grebely J, Byrne M, et al. Evaluation of hepatitis C treatment-as-prevention within Australian prisons (SToP-C): a prospective cohort study. *Lancet Gastroenterology & Hepatology* 2021.

453. Hellard ME, Hocking JS, Crofts N. The prevalence and the risk behaviours associated with the transmission of hepatitis C virus in Australian correctional facilities. *Epidemiology & Infection* 2004.

454. Hockings BA, Young, M., Falconer, A., and O'Rourke, P.K. Queensland Women Prisoners' Health Survey. Brisbane: Department of Corrective Services, 2002.

455. Indig D, Topp L, Ross B, et al. 2009 NSW Inmate Health Survey: Key Findings Report. Sydney: Justice Health, 2010.

456. Keen C, Kinner SA, Borschmann R, Young JT. Comparing the predictive capability of self-report and medically-verified non-fatal overdose in adults released from prison: A prospective data linkage study. *Drug and Alcohol Dependence* 2020.

457. Kevin M. Drug Use in the Inmate Population–prevalence, nature and context. *DUIP NSW–6th Biennial data collection 2009‑10: Overview and Series Trend* 2013.

458. Kinner SA. The post-release experience of prisoners in Queensland. 2006.

459. Larney S, Monkley DL, Indig D, Hampton SE. A cross-sectional study of susceptibility to vaccine-preventable diseases among prison entrants in New South Wales. *Medical Journal of Australia* 2013.

460. Loxley W. Drug use, intoxication and offence type in two groups of alleged offenders in Perth: A pilot study. *Australian and New Zealand Journal of Criminology* 2001.

461. Nicholson J, Almond L, Rizvi N, Fairley CK. Low prevalence of STIs among women in prison, but bacterial vaginosis is common. *Australian and New Zealand Journal of Public Health* 2003.

462. Reekie JM, Levy MH, Richards AH, et al. Trends in prevalence of HIV infection, hepatitis B and hepatitis C among Australian prisoners - 2004, 2007, 2010. *Medical Journal of Australia* 2014.

463. Sheehan Y, Cunningham EB, Cochrane A, et al. A 'one-stop-shop' point-of-care hepatitis C RNA testing intervention to enhance treatment uptake in a reception prison: The PIVOT study. *Journal of Hepatology* 2023; **79(3)**: 635-44.

464. Lythgoe J, Kolodziej, J & Hollingshead, B. People living with HIV in prisons. 2022.

465. Hessou S, Dougnon VT, Glele-Ahanhanzo Y, et al. A behavioral and serological survey on HIV prevalence among prisoners in Benin. *Journal of Public Health in Africa* 2017.

466. Catraye DJ, Ky-Ba, A & Tavi-Ouattarra, A.Y. ENQUETE BIO COMPORTEMENTALE DU VIH-SIDA EN MILIEU CARCERAL AUPRES DES DETENUS HOMMES ET FEMMES AU BURKINA FASO. 2017.

467. Ba AK, Sanou M, Diallo I, et al. Bio-behavioural HIV survey in prisons on men and women in Burkina Faso. *Australasian Medical Journal* 2017.

468. Diendéré EA, Tiéno H, Bognounou R, et al. Prevalence and risk factors associated with infection by human immunodeficiency virus, hepatitis B virus, syphilis and bacillary pulmonary tuberculosis in prisons in Burkina Faso. *Med Trop (Mars)* 2011.

469. Ouedraogo O, Garanet F, Sawadogo S, Mesenge C, Schmid JBG. Vulnerability of male prisoners to HIV/AIDS in Ouagadougou, Burkina Faso. *Sante Publique* 2015.

470. Kowo MP, Andoulo FA, Sizimboue DT, et al. Seroprevalence of hepatitis B and associated factors among inmates: a cross sectional study in the Douala New Bell Prison, Cameroon. *Pan African Medical Journal* 2021.

471. Noeske J, Kuaban C, Amougou G, Piubello A, Pouillot R. Pulmonary tuberculosis in the Central Prison of Douala, Cameroon. *East African Medical Journal* 2006.

472. Angora B, Assemien J, Laurent A, et al. HIV in prison in low income countries. *Aids* 2011.

473. Receveur MC, Seri B, Koffi A, et al. Prevalence of pulmonary tuberculosis among prison inmates: A cross-sectional survey at the Correctional and Detention Facility of Abidjan, Cote d'Ivoire. *Tropical Medicine and International Health* 2017.

474. Kayomo MK, Hasker E, Aloni M, et al. Outbreak of tuberculosis and multidrug-resistant tuberculosis, Mbuji-Mayi central prison, democratic Republic of the Congo. *Emerging Infectious Diseases* 2018.

475. Mashako KY, Sebahire, V & Murhabazi, V. HIV care and prevention in prison in a country in conflict: community approach in SOFEDI, Bukavu, DR Congo. XIX International AIDS Conference. Washington, D.C.; 2012.

476. Dlamini P, Dlamini P, Mnisi Z, Hariga F. A situational assessment on TB, HIV, syphilis, hepatitis C and hepatitis B infections and associated risk behaviours among prisoners and prison officers in Swaziland; 2012.

477. Kassa Y, Million Y, Biset S, Moges F. Hepatitis b and hepatitis c viral infections and associated factors among prisoners in northeast ethiopia. *Journal of Blood Medicine* 2021.

478. Tadesse K, Ayalew G, Million Y, Gelaw A. Hepatitis B and hepatitis C virus infections and associated factors among prisoners in Gondar City, Northwest Ethiopia. *PLoS One* 2024; **19**(4): e0301973.

479. Kebede W, Abdissa A, Seid Y, Mekonnen Z. Seroprevalence and risk factors of hepatitis B, hepatitis C and HIV infections among prisoners in Jimma Town, Southwest Ethiopia. *Asian Pacific Journal of Tropical Disease* 2017.

480. Sahle ET, Amogne W, Manyazewal T, et al. Prevalence of and risk factors for Human Immunodeficiency Virus (HIV) infection in entrants and residents of an Ethiopian prison. *PLoS One* 2023.

481. Sahle ET, Blumenthal J, Jain S, et al. Bacteriologically-confirmed pulmonary tuberculosis in an Ethiopian prison: Prevalence from screening of entrant and resident prisoners. *PLoS One* 2019.

482. Tsegay B, Gebrecherkos T, Kahsay AG, Abdulkader M. Seroprevalence and Associated Factors of Hepatitis B and Hepatitis C Viral Infections Among Prisoners in Tigrai, Northern Ethiopia. *Infection and Drug Resistance* 2023; **16**: 3743-50.

483. Adjei AA, Armah HB, Gbagbo F, et al. Correlates of HIV, HBV, HCV and syphilis infections among prison inmates and officers in Ghana: A national multicenter study. *BMC Infectious Diseases* 2008.

484. Commission GA. National Health and HIV Survey of Prison Inmates in Ghana. 2013.

485. Sagoe KWC, Atuahene K, Ayiku ANA, et al. Hepatitis B and human immunodeficiency virus infections within correctional facilities in Ghana. *PLoS One* 2023; **18**(11): e0293009.

486. Adjei AA, Armah HB, Gbagbo F, et al. Prevalence of human immunodeficiency virus, hepatitis B virus, hepatitis C virus and syphilis among prison inmates and officers at Nsawan and Accra, Ghana. *Journal of Medical Microbiology* 2006.

487. Adjei AA, Armah HB, Gbagbo F, et al. Correlates of hepatitis C virus infection among incarcerated Ghanaians: a national multicentre study. *Journal of Medical Microbiology* 2007.

488. Adjei AA, Armah HB, Gbagbo F, et al. Prevalence of human immunodeficiency virus, hepatitis B virus, hepatitis C virus and syphilis among prison inmates and officers at Nsawam and Accra, Ghana. 2006; **55**(5): 593-7.

489. Ministry of Health Kenya. MARPs SURVEILLANCE REPORT. *Ministry of Health, National AIDS & STI Control Programme-NASCOP* 2012.

490. Mwatenga SA, Musa AA, Muturi MW, Musyoki AM. Prevalence and associated factors of TB and HIV coinfections among adult inmates with presumptive pulmonary TB in a Kenyan prison. *Tropical Medicine and Health* 2024; **52**(1): 54.

491. Akeke VA, Mokgatle M, Oguntibeju OO. Prevalence of risk factors for transmission of HIV and blood-borne viruses in a prison population. *African Journal of Microbiology Research* 2014.

492. Vessellee DB, Yalley AK, Adjei DN, et al. Prevalence of Hepatitis B Virus Infection among Inmates at the Monrovia Central Prison, Liberia. *Tropical Medicine and Infectious Disease* 2023; **8(3) (no pagination)**.

493. Jones MJ. INTEGRATED BIO-BEHAVIOURAL SURVEILLANCE SURVEY REPORT OF KEY POPULATIONS IN LIBERIA (IBBSS, 2018). In: Health Mo, editor.; 2019.

494. Fenomanana J, Randriatsarafara FM, Ranampy FF, Randriamanantany ZA. A behavioural and HIV serological survey among detainees of Ankazondrano jail in Fianarantsoa, Madagascar. *African Journal of AIDS Research* 2021.

495. Rakotomanana F, Dreyfus A, Randrianarisoa MM, et al. Prevalence of pulmonary tuberculosis and HIV infections and risk factors associated to tuberculosis in detained persons in Antananarivo, Madagascar. *Scientific Reports* 2024; **14**(1): 8640.

496. Banerjee A, Harries AD, Mphasa N, Yadid AE, Nyirenda T, Salaniponi FM. Prevalence of HIV, sexually transmitted disease and tuberculosis amongst new prisoners in a district prison, Malawi. *Tropical Doctor* 2000.

497. Chimphambano C, Komolafe I, Muula A. Prevalence of HIV, HepBsAg and Hep C antibodies among inmates in Chichiri prison, Blantyre, Malawi. *Malawi Medical Journal* 2007; **19**(3): 107-10.

498. Garone DB, Mateyu G, Van Oosterhout J, et al. Expanding HIV and STI care to prisoners: The experience from Zomba Central Prison, Malawi. *Journal of the International Aids Society* 2016; **19**(Supplement 5): 171 EP-2.

499. Mallewa J, Kaombe, T & Simbeye, J. Access to preventive, testing, treatment services and retention in care for HIV key populations groups in Malawi. 2023.

500. Mangochi P, Bossard C, Catacutan C, et al. TB screening, prevention and treatment cascade in a Malawi prison. *The International Journal of Tuberculosis and Lung Disease* 2022; **26**(10): 956-62.

501. Olotu AA, Chiramal JA, Boehm RA, et al. Accelerating Tuberculosis Diagnosis in Mozambican Prisons Using Digital Chest X-rays with Computer Aided Detection: Preliminary Results from a Longitudinal, Comprehensive Health Intervention. *medRxiv* 2024; **02**.

502. Adoga MP, Banwat EB, Forbi JC, et al. Human immunonodeficiency virus, hepatitis B virus and hepatitis C virus: sero-prevalence, co-infection and risk factors among prison inmates in Nasarawa State, Nigeria. *Journal of Infection in Developing Countries* 2009.

503. Okafor IM, Ugwu SO, Okoroiwu HU. Hepatitis C virus infection and its associated factors among prisoners in a Nigerian prison. *BMC Gastroenterol* 2020.

504. Abba OJ, Ibraheem IS, Idoko J. Prevalence and risk factors for HIV/AIDS among male inmates in Jos Prison, Plateau State, Nigeria. *Nigerian Journal of Parasitology* 2011.

505. Bashorun A. HIV prevalence and associated factors amongst prison inmates in Kuje federal prison, Federal Capital Territory, Abuja, Nigeria, 2013. *Pan African Medical Journal* 2015.

506. Chigbu LN, Iroegbu CU. Incidence and spread of Mycobacterium tuberculosis-associated infection among Aba Federal prison inmates in Nigeria. *Journal of Health, Population and Nutrition* 2010.

507. Dada MO, Akanmu AS, Esan OA. Seroprevalence of HIV among male prisoners in Lagos State, Nigeria. *Nigerian Postgraduate Medical Journal* 2006.

508. Lawrence QO, Amadi ANC, Okosa C, Ikpi PO, Chukwuemeka BC. Co-infection of Trichomonas vaginalis and HIV infection and its risk factors among prison inmates in Umuahia, Abia State, South Eastern Nigeria. *Journal of Basic and Applied Zoology* 2021.

509. Muhammed OT, Akpa OM, Atilola GO, Komolafe IOO. Seroprevalence of HIV/AIDS and HIV risk factors among prison inmates in Ogun State, Nigeria. *HIV & AIDS Review* 2012; **11**(1): 25-30.

510. Onoja A, Mohammed SB, Ya'aba Y, Liman M, Njab J. Seroprevalence of HIV among the people of lake Chad basin of borno state, nigeria. *Journal of Phytomedicine and Therapeutics* 2016.

511. AIDS NCftFa. BEHAVIORAL SURVEY COUPLED TO HIV SEROLOGY AMONG PROFESSIONALS SEX, MEN HAVING SEX SEX WITH MEN AND INMATES IN REPUBLIC OF CONGO FINAL REPORT. In: AIDS NCftFa, editor.; 2012.

512. Umutesi J, Klett-Tammen C, Nsanzimana S, Krause G, Ott JJ. Cross-sectional study of chronic hepatitis B virus infection in Rwandan high-risk groups: Unexpected findings on prevalence and its determinants. *BMJ Open* 2021.

513. Jaquet A, Wandeler G, Tine J, et al. HIV infection, viral hepatitis and liver fibrosis among prison inmates in West Africa. *BMC Infectious Diseases* 2016.

514. Agency for the Promotion of Population Activities Senegal. ENQU bÊTE NATIO NALE DE SURVEILLANCE COMBINEE DES IST ET DU VIH/SIDA (ENSC 2019) COMPOSANTE COMPORTEMENTALE. In: SOCIALE MDLSEDLA, editor.; 2020.

515. Sesay M. Seroprevalence Study for Key Populations Sierra Leone. 2012.

516. Ampofo WK. Sierra Leone Integrated Bio-Behavioural Survey and Size Estimation Among Female Sex Workers (FSWs), Men who Have Sex with Men (MSM), Persons who Inject Drugs (PWID), Transgender (TG) and People in Close Settings (PCS). In: Secretariat NHA, editor.; 2021.

517. Telisinghe L, Fielding KL, Malden JL, et al. High tuberculosis prevalence in a South African prison: the need for routine tuberculosis screening. *PLoS One* 2014.

518. The Aurum Institute NICD. Socio-behavioural and structural factors driving HIV/AIDS, STIs and Hepatitis B & C infections among inmates in Correctional Facilities, Johannesburg, 2020.

519. Hoffmann CJ, Herce ME, Chimoyi L, et al. Reaching for 90:90:90 in Correctional Facilities in South Africa and Zambia: Virtual Cross-Section of Coverage of HIV Testing and Antiretroviral Therapy during Universal Test and Treat Implementation. *Journal of Acquired Immune Deficiency Syndromes* 2024; **96(5)**: 465-71.

520. Kim HY, Zishiri V, Page-Shipp L, et al. Symptom and digital chest X-ray TB screening in South African and cost-effectiveness. *International Journal of Tuberculosis and Lung Disease* 2020; **24**(3): 295-+.

521. V Skiti EG, P Gribble, H Hausler. Screening and testing for tuberculosis and HIV in correctional facilitiesin the Western Cape, South Africa. 44th World Conference on Lung Health of the International Union Against Tuberculosis; 2013.

522. Stevenson KA, Podewils LJ, Zishiri VK, Castro KG, Charalambous S. HIV prevalence and the cascade of care in five South African correctional facilities. *PLoS One* 2020.

523. J Angolwisye FK, F Nichombe, M Minja, A Rachow, H Machibia, M Pletschette, P Clowes. First survey on TB and HIV prevalence in the prisons of the Mbeya region in Tanzania. *42nd World Conference on Lung Health of the International Union Against Tuberculosis and Lung Disease* 2011; (157).

524. M. Dahoma EM, A. Othman, A. Seha, A. Abdullah. Predisposing sexual and drug related risk factors among prisoners in Zanzibar. International Aids Society (IAS) 2009. Cape Town; 2009.

525. Mmbaga VM. Prevalence and factors associated with pulmonary tuberculosis among prisoners in Dar es salaam, Tanzania, 2012: Muhimbili University of Health and Allied Sciences; 2013.

526. Mutayoba B, Ngowi B, Kohi W. HIV prevalence and related risk factors in prison settings: findings from a rapid situational assessment in mainland Tanzania. 20th International AIDS Conference, Melbourne, Australia; 2014; 2014.

527. Steiner A, Mangu C, van den Hombergh J, et al. Screening for pulmonary tuberculosis in a tanzanian prison and computer-aided interpretation of chest X-rays. *Public Health Action* 2015.

528. Ekouevi DK, D'Almeida S, Salou M, et al. HIV seroprevalence among inmates in Togo. *Med Mal Infect* 2013.

529. Akakpo AS, Ekouevi DK, Toure AM, et al. Skin disease and HIV infection among inmates in Lome, Togo: a study of 194 prisoners. *Medecine et Sante Tropicales* 2014.

530. Kinaalwa GN, F. Integrated Legal Aid in HIV Programming in Prison Rehabilitation Services for Better Access and Quality HIV Care – Mityana Uganda Charity Experience. 18th International Conference on AIDS and STI's in Africa; 2015.

531. United Nations Office on Drugs and Crime. A Rapid Situation Assessment of HIV/STI/TB and Drug Abuse among Prisoners in Uganda Prisons Service, 2009.

532. Harris JB, Siyambango M, Levitan EB, et al. Derivation of a tuberculosis screening rule for sub-Saharan African prisons. *International Journal of Tuberculosis and Lung Disease* 2014.

533. Kagujje M, Somwe P, Hatwiinda S, et al. Cross-sectional assessment of tuberculosis and HIV prevalence in 13 correctional facilities in Zambia. *BMJ Open* 2021.

534. Maggard KR, Hatwiinda S, Harris JB, et al. Screening for tuberculosis and testing for human immunodeficiency virus in Zambian prisons. *Bulletin of the World Health Organization* 2015.

535. Simooya OO, Sanjobo N, Mulenga C, et al. Aggressive awareness campaigns may not be enough for HIV prevention in prisons-studies in Zambia suggest time for evidence based interventions. *Open Infectious Diseases Journal* 2014.

536. Simooya OO, Sanjobo NE, Kaetano L, et al. 'Behind walls': a study of HIV risk behaviours and seroprevalence in prisons in Zambia. *Aids* 2001.

537. European Union Drug Agency. EUDA Prison Data 2024. Lisbon; 2024.

538. Mohamed HI, Saad ZM, Abd-Elreheem EM, et al. Hepatitis C, hepatitis B and HIV infection among Egyptian prisoners: seroprevalence, risk factors and related chronic liver diseases. *Journal of Infection and Public Health* 2013.

539. Mahfoud Z, Kassak K, Kreidieh K, Shamra S, Ramia S. Prevalence of antibodies to human immunodeficiency virus (HIV), hepatitis B and hepatitis C and risk factors in prisoners in Lebanon. *Journal of Infection in Developing Countries* 2010.

540. Ministry of Health Lebanon. AN INTEGRATED BIO-BEHAVIORAL SURVEILLANCE STUDY AMONG MOST AT RISK POPULATIONS IN LEBANON: FEMALE SEX WORKERS, INJECTING DRUG USERS, MEN WHO HAVE SEX WITH MEN, AND PRISONERS. 2008.

541. Ziglam H, Zorgani AA, Balouz A, Abudhe AH, Elahmer O. Prevalence of antibodies to human immunodeficiency virus, hepatitis B, and hepatitis C in prisoners in Libya. *Libyan Journal of Medicine* 2012.

542. Elahmer O, Zorgani A, Abudher A, Ziglam H. Prevalence of human immunodeficiency virus, hepatitis B virus, hepatitis C virus among prison inmates, western Libya. *Clinical Microbiology and Infection* 2012.

543. El Ghrari K, Terrab Z, Benchikhi H, Lakhdar H, Jroundi I, Bennani M. Prevalence of syphilis and HIV infection in female prisoners in Morocco. [French]. *Eastern Mediterranean Health Journal* 2007.

544. Alshowair A, Assiri AM, Balfas AH, et al. Magnitude and Determinants of Latent Tuberculosis Among Inmates of Saudi Correctional Facilities: A Cross-Sectional Study. *International Journal of General Medicine* 2024; **17**: 4475-83.

545. El-Daly MM, Fageeh W, El-Kafrawy SA, et al. Hepatitis B and Hepatitis C Infections among Female Inmates in a Prison in Jeddah, Saudi Arabia. *Clinical Laboratory* 2024; **70(3)**: 571-8.

546. Group SHEA. Epidemiology of HIV in Sudan Staging and Analysis. In: Group SHEA, editor.; 2013.

547. Kobeissi L. The Integrated Bio-Behavioral Survey (IBBS) in Syria: 2013-2014. In: Program UND, editor.; 2014.

548. Keten D, Ova ME, Keten HS, et al. The prevalence of hepatitis B and C among prisoners in Kahramanmaras, Turkey. *Jundishapur Journal of Microbiology* 2016.

549. Sahin AR, Sahin AM, Gunduz A, Aktemur A, Kes-Uzun N. HIV seropositivity in a penal institution in Turkey: A cross-sectional study. [Turkish]. *Klimik Dergisi* 2018.

550. Yilmaz EM, Bilgin M, Koksal ZS, Oruc MA, Tabak F. Investigation of the Prevalence of HBsAg, Anti-HCV, and Anti-HIV in a Prison in Turkey: A Point Prevalence Study. *Viral Hepatitis Journal* 2023; **29(3)**: 119-23.

551. Balci E, Turker K, Senol V, Gunay O. Screening Indicators of Hepatitis A, Hepatitis B, Hepatitis C and HIV infections in Prisoners. *Viral Hepatit Dergisi-Viral Hepatitis Journal* 2012.

552. ÖZger HS, KaraŞAhİN Ö, Toy MA, Yilmaz Sİ, Hizel K. Hepatitis C Prevalence and Responses to Pegylated Interferon + Ribavirin Treatment Among Prisoners. *Viral Hepatitis Journal / Viral Hepatit Dergisi* 2017.

553. Sahin AM, Sahin AR, Gunduz A, Aktemur A, Uzun N. Prevalence of Hepatitis B virus and Hepatitis C virus among prison inmates in Istanbul, Turkey. *Annals of Clinical and Analytical Medicine* 2022.

554. Butler T, Boonwaat L, Hailstone S, et al. The 2004 Australian prison entrants' blood-borne virus and risk behaviour survey. *Australian and New Zealand Journal of Public Health* 2007.

555. Health J. National Patient Health Survey. 2017.

556. Stoové M, Kirwan A. External component of the evaluation of drug policies and services and their subsequent effects on prisoners and staff within the Alexander Maconochie Centre. 2011.

557. Watkins RE, Mak DB, Connelly C. Testing for sexually transmitted infections and blood borne viruses on admission to Western Australian prisons. *BMC Public Health* 2009.

558. Awofeso N, Harper SE, Levy MH. Prevalence of exposure to hepatitis C virus among prison inmates, 1999 [4]. *Medical Journal of Australia* 2000.

559. Butler T, Spencer J, Cui J, Vickery K, Zou J, Kaldor J. Seroprevalence of markers for hepatitis B, C and G in male and female prisoners‐NSW, 1996. *Australian and New Zealand Journal of Public Health* 1999; **23**(4): 377-84.

560. Dore G, Hajarizadeh B, Grebely J, et al. Declining HCV incidence following rapid HCV treatment scale-up in a prison network in Australia: Evidence of treatment as prevention from the SToP-C study. *Journal of Hepatology* 2020.

561. Indig D, Topp L, Ross B, et al. 2009 NSW inmate health survey: key findings report. *Justice Health, Sydney* 2010: 16.

562. McCartney EM, Ralton L, Dawe J, et al. Point-of-Care Testing for Hepatitis C in the Priority Settings of Mental Health, Prisons, and Drug and Alcohol Facilities—the PROMPt Study. *Clinical Infectious Diseases* 2024; **79**(4): 965-73.

563. Miller ER, Bi P, Ryan P. The prevalence of HCV antibody in South Australian prisoners. *Journal of Infection* 2006.

564. Miller ER, Bi P, Ryan P. Hepatitis C virus infection in South Australian prisoners: seroprevalence, seroconversion, and risk factors. *International Journal of Infectious Diseases* 2009.

565. Murray N, LePage E, Butler T. Hearing health of New South Wales prison inmates. *Australian and New Zealand Journal of Public Health* 2004.

566. Papaluca T, Howell J, McDonald L, Craigie A, Iser D. Chronic hepatitis B within the Victorian Prisons, Australia-high prevalence of cirrhosis and HBV-HCV and HBV-HDV coinfection. *Hepatology International* 2018.

567. Snow KJ, Richards AH, Kinner SA. Use of multiple data sources to estimate hepatitis C seroprevalence among prisoners: A retrospective cohort study. *PLoS One* 2017.

568. Wallis C, O'Flynn M, Fenech M, Grimstrup D. Hepatitis C virus point-of-care RNA testing: Experience from screening an entire high-security Australian prison population over 3 days. *Australian and New Zealand Journal of Public Health* 2023; **47(5)**: 100083.

569. Young JT, Van Dooren K, Borschmann R, Kinner S. ACT detainee health and wellbeing survey 2016: Summary results: ACT government; 2017.

## *Table 9.2*: Country-level estimates of the number and rate of incarceration, prevalence of HBV and tuberculosis among total people incarcerated

|  | **People who are incarcerated** | | | | **People with current HBV** | | | | **People with active Tuberculosis** | | | |
| --- | --- | --- | --- | --- | --- | --- | --- | --- | --- | --- | --- | --- |
| **Country** | **Estimated number^1^** | **Year of estimate^1^** | **Rate per 100,000** | **Trend in incarceration¶** | **% (CI)** | **Estimated no. (CI)** | **Year of estimate** | **Sources** | **% (CI)** | **Estimated no. (CI)** | **Year of estimate** | **Sources** |
| **Eastern Europe** |  |  |  |  |  |  |  |  |  |  |  |  |
| Armenia | 2469 | 2024 | 132 | Decreasing | 3·6 (2·2, 5·4) | < 500 (<500,<500) | 2004 | 1 | ·· | ·· | ·· |  |
| Azerbaijan | 24698 | 2023 | 345 | Stable | 4·7 (3·2, 6·7) | 1000 (1000,1500) | 2006-14 | 2-4 | ·· | ·· | ·· |  |
| Belarus | 32556 | 2018 | 498 | Stable | ·· | ·· | ·· |  | ·· | ·· | ·· |  |
| Bosnia & Herzegovina | 2212 | 2024/2023 | 24 | Decreasing | 1·5 (0·7, 2·7) | < 500 (<500,<500) | 2012 | 5 | ·· | ·· | ·· |  |
| Bulgaria | 6378 | 2024 | 146 | Decreasing | ·· | ·· | ·· |  | 0·3 (0·0, 1·0) | < 500 (<500,<500) | 2009 | 6 |
| Czechia | 19649 | 2024 | 294 | Stable | 4·1 (1·7, 7·5) | 1000 (500,1500) | 2002 | 7 | ·· | ·· | ·· |  |
| Estonia | 1688 | 2024 | 201 | Decreasing | ·· | ·· | ·· |  | ·· | ·· | ·· |  |
| Georgia | 10457 | 2024 | 432 | Increasing | ·· | ·· | ·· |  | 6·0 (5·5, 6·5) | 500 (500,500) | 1998 | 8 |
| Hungary | 18270 | 2023 | 289 | Increasing | 1·1 (0·6, 1·9) | < 500 (<500,500) | 2010 | 9 | ·· | ·· | ·· |  |
| Latvia | 3271 | 2024 | 278 | Decreasing | ·· | ·· | ·· |  | ·· | ·· | ·· |  |
| Lithuania | 4551 | 2024 | 254 | Decreasing | ·· | ·· | ·· |  | ·· | ·· | ·· |  |
| Republic of Moldova | 5695 | 2024 | 279 | Decreasing | ·· | ·· | ·· |  | ·· | ·· | ·· |  |
| Poland | 70316 | 2024 | 279 | Stable | ·· | ·· | ·· |  | 1·6 (0·8, 2·8) | 1000 (500,2000) | 2012 | 10 |
| Romania | 24534 | 2024 | 195 | Stable | 10·6 (6·0, 16·3) | 2500 (1500,4000) | 2009 | 11 | 0·1 (0·0, 0·2) | < 500 (<500,<500) | 2019 | 12 |
| Russian Federation | 433006 | 2023 | 447 | Decreasing | ·· | ·· | ·· |  | 5·4 (4·6, 6·4) | 23500 (20000,27500) | 1998 | 13 |
| Slovakia | 8585 | 2024 | 236 | Decreasing | ·· | ·· | ·· |  | ·· | ·· | ·· |  |

| Ukraine | 44024 | 2024 | 150 | Decreasing | 5·2 (3·2, 7·6) | 2500 (1500,3500) | 2011 | 14 | ·· | ·· | ·· |  |
| --- | --- | --- | --- | --- | --- | --- | --- | --- | --- | --- | --- | --- |

| **Western Europe** |  |  |  |  |  |  |  |  |  |  |  |  |
| --- | --- | --- | --- | --- | --- | --- | --- | --- | --- | --- | --- | --- |
| Albania | 4653 | 2024 | 242 | Decreasing | ·· | ·· | ·· |  | ·· | ·· | ·· |  |
| Andorra | 51 | 2024 | 89 | Decreasing | ·· | ·· | ·· |  | ·· | ·· | ·· |  |
| Austria | 9288 | 2024 | 157 | Stable | ·· | ·· | ·· |  | ·· | ·· | ·· |  |
| Belgium | 12575 | 2024 | 169 | Increasing | 0·8 (0·3, 1·5) | < 500 (<500,<500) | 2019 | 15 | ·· | ·· | ·· |  |
| Croatia | 4445 | 2023 | 171 | Increasing | 1·1 (0·8, 1·6) | < 500 (<500,<500) | 2008-09 | 16,17 | ·· | ·· | ·· |  |
| Denmark | 4083 | 2024 | 110 | Increasing | 4·9 (2·7, 7·7) | < 500 (<500,500) | 1999 | 18 | ·· | ·· | ·· |  |
| England and Wales | 85867 | 2024 | 223 | Stable | 0·9 (0·0, 2·7) | 500 (<500,2500) | 2009-23 | 19-21 | ·· | ·· | ·· |  |
| Finland | 2912 | 2023 | 85 | Stable | 0·3 (0·0, 1·5) | < 500 (<500,<500) | 2022 | 22 | ·· | ·· | ·· |  |
| France | 79631 | 2024 | 201 | Increasing | 1·2 (0·1, 3·4) | 1000 (<500,2500) | 1998-2019 | 23-27 | ·· | ·· | ·· |  |
| Germany | 57955 | 2023 | 109 | Stable | ·· | ·· | ·· |  | ·· | ·· | ·· |  |
| Greece | 10242 | 2024 | 155 | Stable | ·· | ·· | ·· |  | 13·3 (8·8, 18·4) | 1500 (1000,2000) | 2008 | 28 |
| Greenland | 154 | 2023 | 393 | Increasing | ·· | ·· | ·· |  | ·· | ·· | ·· |  |
| Iceland | 140 | 2024 | 57 | Decreasing | ·· | ·· | ·· |  | ·· | ·· | ·· |  |
| Ireland | 5074 | 2024 | 156 | Increasing | 0·3 (0·0, 0·8) | < 500 (<500,<500) | 2011 | 29 | ·· | ·· | ·· |  |
| Italy | 62110 | 2024 | 166 | Increasing | 3·0 (1·3, 5·3) | 2000 (1000,3500) | 2003-22 | 30-35 | ·· | ·· | ·· |  |
| Liechtenstein | 14 | 2024 | 54 | Increasing | ·· | ·· | ·· |  | ·· | ·· | ·· |  |
| Luxembourg | 600 | 2024 | 135 | Decreasing | ·· | ·· | ·· |  | ·· | ·· | ·· |  |
| Malta | 671 | 2024 | 187 | Stable | ·· | ·· | ·· |  | 0·8 (0·1, 2·1) | < 500 (<500,<500) | 2019 | 36 |
| Monaco | 31 | 2024 | 165 | Increasing | ·· | ·· | ·· |  | ·· | ·· | ·· |  |
| Montenegro | 1046 | 2024 | 254 | Stable | 1·5 (0·6, 2·9) | < 500 (<500,<500) | 2012-21 | 37 | ·· | ·· | ·· |  |
| Netherlands | 11537 | 2023 | 102 | Increasing | 1·5 (0·2, 3·7) | < 500 (<500,500) | 2010 | 38 | ·· | ·· | ·· |  |
| North Macedonia | 2555 | 2024 | 176 | Decreasing | ·· | ·· | ·· |  | ·· | ·· | ·· |  |
| Northern Ireland | 1911 | 2024 | 159 | Increasing | ·· | ·· | ·· |  | ·· | ·· | ·· |  |
| Norway | 3052 | 2024 | 87 | Decreasing | ·· | ·· | ·· |  | ·· | ·· | ·· |  |
| Portugal | 12379 | 2024 | 188 | Decreasing | 2·7 (1·9, 4·0) | 500 (<500,500) | 2002-12 | 39-42 | ·· | ·· | ·· |  |
| San Marino | 1 | 2024 | 4 | Decreasing | ·· | ·· | ·· |  | ·· | ·· | ·· |  |
| Scotland | 8253 | 2024 | 235 | Stable | ·· | ·· | ·· |  | ·· | ·· | ·· |  |
| Serbia | 10787 | 2023 | 227 | Increasing | ·· | ·· | ·· |  | ·· | ·· | ·· |  |
| Slovenia | 1798 | 2024 | 133 | Increasing | ·· | ·· | ·· |  | ·· | ·· | ·· |  |
| Spain | 56698 | 2023 | 181 | Decreasing | 2·0 (0·6, 4·2) | 1000 (500,2500) | 2008-18 | 43-46 | 1·3 (0·1, 5·2) | 1000 (<500,3000) | 1999 | 47 |
| Sweden | 10175 | 2024 | 156 | Increasing | 1·9 (0·8, 3·8) | < 500 (<500,500) | 2017 | 48 | ·· | ·· | ·· |  |
| Switzerland | 6881 | 2024 | 120 | Stable | 2·0 (1·2, 2·9) | < 500 (<500,<500) | 2007-18 | 49-51 | 1·7 (1·3, 2·2) | < 500 (<500,<500) | 2009 | 52 |
| **East and South East Asia** |  |  |  |  |  |  |  |  |  |  |  |  |
| Brunei Darussalam | 636 | 2022 | 199 | Stable | ·· | ·· | ·· |  | ·· | ·· | ·· |  |
| Cambodia | 45122 | 2024 | 417 | Increasing | ·· | ·· | ·· |  | ·· | ·· | ·· |  |
| China | 1690000 | 2018 | 13 | Decreasing | ·· | ·· | ·· |  | 1·5 (0·9, 2·3) | 26000 (15500,39000) | 2017 | 53 |
| Hong Kong† | 9079 | 2023 | 178 | Increasing | ·· | ·· | ·· |  | 5·4 (4·0, 7·0) | 500 (500,500) | 2001 | 54 |
| Indonesia | 274060 | 2024 | 148 | Increasing | 5·0 (2·8, 7·7) | 13500 (8000,21000) | 2008-21 | 55-59 | 0·0 (0·0, 0·5) | < 500 (<500,1500) | 2022 | 60JW02 |
| Japan | 40881 | 2023 | 56 | Decreasing | ·· | ·· | ·· |  | ·· | ·· | ·· |  |
| Lao People's Democratic Republic | 11885 | 2018 | 262 | Increasing | ·· | ·· | ·· |  | ·· | ·· | ·· |  |
| Malaysia | 87419 | 2024 | 373 | Increasing | ·· | ·· | ·· |  | ·· | ·· | ·· |  |
| Mongolia | 5700 | 2023 | 270 | Stable | ·· | ·· | ·· |  | ·· | ·· | ·· |  |
| Myanmar | 100324 | 2020 | 274 | Increasing | ·· | ·· | ·· |  | ·· | ·· | ·· |  |
| Democratic People's Republic of Korea* | 100000 | 2016 | 552 |  | ·· | ·· | ·· |  | ·· | ·· | ·· |  |
| Philippines | 171247 | 2024 | 235 | Stable | 7·4 (5·2, 9·9) | 12500 (9000,17000) | 1997-2014 | 61,62 | ·· | ·· | ·· |  |
| Singapore | 9536 | 2022 | 217 | Decreasing | ·· | ·· | ·· |  | ·· | ·· | ·· |  |
| Republic of Korea | 52940 | 2022 | 143 | Stable | ·· | ·· | ·· |  | ·· | ·· | ·· |  |
| Taiwan† | 58889 | 2024 | 346 | Stable | ·· | ·· | ·· |  | 0·2 (0·2, 0·2) | < 500 (<500,<500) | 2000 | 63 |
| Thailand | 274277 | 2023 | 550 | Decreasing | 6·5 (5·1, 8·2) | 18000 (14000,22500) | 2020 | 64 | 1·7 (0·8, 2·9) | 4500 (2000,8000) | 2000-22 | 65-68,69ZW01,70 |
| Timor-Leste | 763 | 2021 | 97 | Increasing | ·· | ·· | ·· |  | ·· | ·· | ·· |  |

| Viet Nam | 133986 | 2022 | 200 | Stable | ·· | ·· | ·· |  | ·· | ·· | ·· |  |
| --- | --- | --- | --- | --- | --- | --- | --- | --- | --- | --- | --- | --- |

| **South Asia** |  |  |  |  |  |  |  |  |  |  |  |  |
| --- | --- | --- | --- | --- | --- | --- | --- | --- | --- | --- | --- | --- |
| Afghanistan | 19000 | 2024 | 87 | Decreasing | ·· | ·· | ·· |  | ·· | ·· | ·· |  |
| Bangladesh | 53831 | 2024 | 47 | Decreasing | ·· | ·· | ·· |  | 1·0 (0·9, 1·1) | 500 (500,500) | 2008-13 | 71-73 |
| Bhutan | 1119 | 2014 | 227 | Increasing | ·· | ·· | ·· |  | ·· | ·· | ·· |  |
| India | 573220 | 2022 | 60 | Increasing | 7·9 (0·6, 22·0) | 45500 (3500,126000) | 2010-21 | 74-76 | 3·5 (3·0, 4·0) | 20000 (17500,23000) | 2013-19 | 77-80 |
| Iran (Islamic Republic of) | 189000 | 2020 | 314 | Decreasing | 3·4 (2·6, 4·4) | 6500 (5000,8500) | 2003-18 | 81-89 | 0·1 (0·1, 0·2) | 500 (<500,500) | 2007-17 | 90-94 |
| Maldives | 1700 | 2020 | 448 | Decreasing | ·· | ·· | ·· |  | ·· | ·· | ·· |  |
| Nepal | 27550 | 2022 | 142 | Increasing | ·· | ·· | ·· |  | 1·8 (0·7, 3·2) | 500 (<500,1000) | 2014-17 | 95ZW01,96 |
| Pakistan | 108643 | 2024 | 80 | Increasing | 2·7 (2·2, 3·4) | 3000 (2500,3500) | 2005-10 | 97-100 | 0·5 (0·3, 0·7) | 500 (500,1000) | 2002-17 | 98,101-103 |

| Sri Lanka | 29686 | 2024 | 208 | Increasing | 0·3 (0·0, 1·4) | < 500 (<500,500) | 2013 | 104 | ·· | ·· | ·· |  |
| --- | --- | --- | --- | --- | --- | --- | --- | --- | --- | --- | --- | --- |

| **Central Asia** |  |  |  |  |  |  |  |  |  |  |  |  |
| --- | --- | --- | --- | --- | --- | --- | --- | --- | --- | --- | --- | --- |
| Kazakhstan | 35228 | 2022 | 294 | Decreasing | ·· | ·· | ·· |  | ·· | ·· | ·· |  |
| Kyrgyzstan | 7728 | 2023 | 194 | Decreasing | 6·3 (3·7, 9·5) | 500 (500,500) | 2014 | 105 | ·· | ·· | ·· |  |
| Tajikistan | 14000 | 2022 | 238 | Increasing | ·· | ·· | ·· |  | 4·4 (3·4, 5·5) | 500 (500,1000) | 2010 | 106 |
| Turkmenistan | 35000 | 2021 | 864 | Increasing | ·· | ·· | ·· |  | ·· | ·· | ·· |  |

| Uzbekistan | 29000 | 2022 | 131 | Decreasing | ·· | ·· | ·· |  | ·· | ·· | ·· |  |
| --- | --- | --- | --- | --- | --- | --- | --- | --- | --- | --- | --- | --- |

| **Caribbean** |  |  |  |  |  |  |  |  |  |  |  |  |
| --- | --- | --- | --- | --- | --- | --- | --- | --- | --- | --- | --- | --- |
| Antigua & Barbuda | 400 | 2023 | 604 | Stable | ·· | ·· | ·· |  | ·· | ·· | ·· |  |
| Bahamas | 1912 | 2021 | 653 | Increasing | ·· | ·· | ·· |  | ·· | ·· | ·· |  |
| Barbados | 692 | 2023 | 367 | Decreasing | ·· | ·· | ·· |  | ·· | ·· | ·· |  |
| Bermuda | 124 | 2021 | 295 | Decreasing | ·· | ·· | ·· |  | ·· | ·· | ·· |  |
| Cuba | 90000 | 2020 | 1161 | Increasing | ·· | ·· | ·· |  | ·· | ·· | ·· |  |
| Dominica | 260 | 2024 | 507 | Increasing | ·· | ·· | ·· |  | ·· | ·· | ·· |  |
| Dominican Republic | 25987 | 2024 | 357 | Stable | ·· | ·· | ·· |  | ·· | ·· | ·· |  |
| Grenada | 385 | 2023 | 468 | Decreasing | ·· | ·· | ·· |  | ·· | ·· | ·· |  |
| Haiti | 7523 | 2024 | 104 | Decreasing | ·· | ·· | ·· |  | ·· | ·· | ·· |  |
| Jamaica | 3559 | 2022 | 174 | Decreasing | ·· | ·· | ·· |  | ·· | ·· | ·· |  |
| Commonwealth of Puerto Rico | 5798 | 2022 | 278 | Decreasing | ·· | ·· | ·· |  | ·· | ·· | ·· |  |
| Saint Kitts & Nevis | 160 | 2022 | 476 | Decreasing | ·· | ·· | ·· |  | ·· | ·· | ·· |  |
| Saint Lucia | 572 | 2023 | 438 | Decreasing | ·· | ·· | ·· |  | ·· | ·· | ·· |  |
| Saint Vincent & the Grenadines | 404 | 2024 | 577 | Stable | ·· | ·· | ·· |  | ·· | ·· | ·· |  |

| Trinidad & Tobago | 3802 | 2021 | 358 | Stable | ·· | ·· | ·· |  | ·· | ·· | ·· |  |
| --- | --- | --- | --- | --- | --- | --- | --- | --- | --- | --- | --- | --- |

| **Latin America** |  |  |  |  |  |  |  |  |  |  |  |  |
| --- | --- | --- | --- | --- | --- | --- | --- | --- | --- | --- | --- | --- |
| Argentina | 125041 | 2023 | 426 | Increasing | 0·5 (0·3, 0·8) | 500 (500,1000) | 2016-18 | 107,108 | ·· | ·· | ·· |  |
| Belize | 1339 | 2024 | 501 | Decreasing | ·· | ·· | ·· |  | ·· | ·· | ·· |  |
| Bolivia (Plurinational State of) | 31105 | 2024 | 403 | Increasing | 0·5 (0·0, 1·6) | < 500 (<500,500) | 2013 | 109,110 | ·· | ·· | ·· |  |
| Brazil | 888791 | 2024 | 593 | Increasing | 1·1 (0·3, 2·6) | 9500 (2500,23000) | 1994-2021 | 111-124 | 3·8 (2·4, 5·8) | 34000 (21000,51500) | 2002-23 | 125JW01,126-129,130ZW01,131,132ZW01,133,134,135JW01,136-138,139ZW01,140ZW01,141,142ZW01,143-145,146ZW01,147ZW01,148-150,151ZW01,152,153,154ZW01 |
| Chile | 59037 | 2024 | 440 | Increasing | ·· | ·· | ·· |  | ·· | ·· | ·· |  |
| Colombia | 104346 | 2024 | 291 | Decreasing | 0·4 (0·0, 1·3) | 500 (<500,1500) | 2019 | 155 | 0·8 (0·5, 1·1) | 1000 (500,1000) | 2010-15 | 156-159 |
| Costa Rica | 17829 | 2022 | 502 | Decreasing | ·· | ·· | ·· |  | ·· | ·· | ·· |  |
| Ecuador | 33669 | 2024 | 286 | Stable | ·· | ·· | ·· |  | ·· | ·· | ·· |  |
| El Salvador | 109519 | 2024 | 2625 | Increasing | ·· | ·· | ·· |  | ·· | ·· | ·· |  |
| Guatemala | 23361 | 2023 | 213 | Stable | ·· | ·· | ·· |  | ·· | ·· | ·· |  |
| Guyana | 2300 | 2024 | 439 | Stable | ·· | ·· | ·· |  | ·· | ·· | ·· |  |
| Honduras | 19481 | 2023 | 291 | Stable | ·· | ·· | ·· |  | ·· | ·· | ·· |  |
| Mexico | 234514 | 2024 | 277 | Decreasing | 0·1 (0·0, 0·2) | < 500 (<500,500) | 2003-15 | 160-163 | ·· | ·· | ·· |  |
| Nicaragua | 20918 | 2018 | 496 | Increasing | ·· | ·· | ·· |  | ·· | ·· | ·· |  |
| Panama | 23798 | 2024 | 841 | Increasing | ·· | ·· | ·· |  | ·· | ·· | ·· |  |
| Paraguay | 17712 | 2023 | 408 | Increasing | ·· | ·· | ·· |  | ·· | ·· | ·· |  |
| Peru | 97605 | 2024 | 443 | Increasing | ·· | ·· | ·· |  | 24·1 (12·2, 39·9) | 23500 (12000,39000) | 2021 | 164ZW01,165ZW01 |
| Suriname | 1000 | 2014 | 270 | Stable | ·· | ·· | ·· |  | ·· | ·· | ·· |  |
| Uruguay | 15767 | 2024 | 707 | Increasing | ·· | ·· | ·· |  | ·· | ·· | ·· |  |

| Venezuela (Bolivarian Republic of) | 67200 | 2022 | 375 | Increasing | 0·0 (0·0, 4·1) | < 500 (<500,2500) | 2018 | 166 | ·· | ·· | ·· |  |
| --- | --- | --- | --- | --- | --- | --- | --- | --- | --- | --- | --- | --- |

| **North America** |  |  |  |  |  |  |  |  |  |  |  |  |
| --- | --- | --- | --- | --- | --- | --- | --- | --- | --- | --- | --- | --- |
| Canada | 34986 | 2023 | 139 | Decreasing | 0·0 (0·0, 2·5) | < 500 (<500,1000) | 2021 | 167 | ·· | ·· | ·· |  |

| United States of America | 1808100 | 2022 | 522 | Decreasing | 2·9 (0·3, 9·2) | 53000 (4500,166500) | 1999-2022 | 168-174 | 0·0 (0·0, 0·0) | 500 (500,500) | 1994-2008 | 175,176,177ZW01 |
| --- | --- | --- | --- | --- | --- | --- | --- | --- | --- | --- | --- | --- |

| **Pacific Island States & Terr·** |  |  |  |  |  |  |  |  |  |  |  |  |
| --- | --- | --- | --- | --- | --- | --- | --- | --- | --- | --- | --- | --- |
| American Samoa | 301 | 2022 | 1018 | Increasing | ·· | ·· | ·· |  | ·· | ·· | ·· |  |
| Micronesia (Federated States of) | 132 | 2014 | 40 | Increasing | ·· | ·· | ·· |  | ·· | ·· | ·· |  |
| Fiji | 2276 | 2024 | 377 | Increasing | ·· | ·· | ·· |  | ·· | ·· | ·· |  |
| French Polynesia | 575 | 2024 | 275 | Increasing | ·· | ·· | ·· |  | ·· | ·· | ·· |  |
| Guam | 896 | 2024 | 844 | Increasing | ·· | ·· | ·· |  | ·· | ·· | ·· |  |
| Kiribati | 129 | 2016 | 179 | Increasing | ·· | ·· | ·· |  | ·· | ·· | ·· |  |
| Marshall Islands | 35 | 2014 | 117 | Decreasing | ·· | ·· | ·· |  | ·· | ·· | ·· |  |
| Nauru | 38 | 2023 | 516 | Increasing | ·· | ·· | ·· |  | ·· | ·· | ·· |  |
| New Caledonia | 609 | 2024 | 317 | Increasing | ·· | ·· | ·· |  | ·· | ·· | ·· |  |
| Northern Mariana Islands | 170 | 2022 | 501 | Stable | ·· | ·· | ·· |  | ·· | ·· | ·· |  |
| Palau | 66 | 2023 | 527 | Stable | ·· | ·· | ·· |  | ·· | ·· | ·· |  |
| Papua New Guinea | 5373 | 2023 | 87 | Stable | ·· | ·· | ·· |  | ·· | ·· | ·· |  |
| Samoa | 358 | 2019 | 296 | Decreasing | ·· | ·· | ·· |  | ·· | ·· | ·· |  |
| Solomon Islands | 500 | 2019 | 131 | Increasing | ·· | ·· | ·· |  | ·· | ·· | ·· |  |
| Tonga | 557 | 2022 | 888 | Increasing | ·· | ·· | ·· |  | ·· | ·· | ·· |  |
| Tuvalu | 11 | 2014 | 161 | Increasing | ·· | ·· | ·· |  | ·· | ·· | ·· |  |

| Vanuatu | 195 | 2021 | 108 | Increasing | ·· | ·· | ·· |  | ·· | ·· | ·· |  |
| --- | --- | --- | --- | --- | --- | --- | --- | --- | --- | --- | --- | --- |

| **Australasia** |  |  |  |  |  |  |  |  |  |  |  |  |
| --- | --- | --- | --- | --- | --- | --- | --- | --- | --- | --- | --- | --- |
| Australia‡§ | 44051 | 2024 | 261 | Increasing | 0·5 (0·3, 1·1) | < 500 (<500,500) | 2023 | 178 | ·· | ·· | ·· |  |

| New Zealand | 9924 | 2024 | 297 | Stable | ·· | ·· | ·· |  | ·· | ·· | ·· |  |
| --- | --- | --- | --- | --- | --- | --- | --- | --- | --- | --- | --- | --- |

| **Sub Saharan Africa** |  |  |  |  |  |  |  |  |  |  |  |  |
| --- | --- | --- | --- | --- | --- | --- | --- | --- | --- | --- | --- | --- |
| Angola | 24068 | 2024 | 134 | Decreasing | ·· | ·· | ·· |  | ·· | ·· | ·· |  |
| Benin | 19563 | 2024 | 277 | Increasing | ·· | ·· | ·· |  | ·· | ·· | ·· |  |
| Botswana | 3971 | 2022 | 242 | Decreasing | ·· | ·· | ·· |  | 2·0 (1·4, 2·7) | < 500 (<500,<500) | 2002 | 179JW01,180 |
| Burkina Faso | 8800 | 2022 | 75 | Stable | 28·0 (22·9, 33·7) | 2500 (2000,3000) | 2009 | 181 | 1·3 (0·3, 3·0) | < 500 (<500,500) | 2009 | 181 |
| Burundi | 13824 | 2024 | 215 | Increasing | ·· | ·· | ·· |  | ·· | ·· | ·· |  |
| Cameroon | 34419 | 2024 | 231 | Stable | 12·9 (10·8, 15·1) | 4500 (3500,5000) | 2019 | 182 | 2·3 (0·6, 5·0) | 1000 (<500,1500) | 2004-19 | 183-185 |
| Cabo Verde | 2700 | 2024 | 676 | Increasing | ·· | ·· | ·· |  | ·· | ·· | ·· |  |
| Central African Republic | 2678 | 2023 | 100 | Increasing | ·· | ·· | ·· |  | ·· | ·· | ·· |  |
| Chad | 9589 | 2022 | 111 | Stable | ·· | ·· | ·· |  | ·· | ·· | ·· |  |
| Comoros | 422 | 2023 | 89 | Increasing | ·· | ·· | ·· |  | ·· | ·· | ·· |  |
| Côte d'Ivoire | 27149 | 2024 | 177 | Increasing | ·· | ·· | ·· |  | 5·6 (4·4, 7·1) | 1500 (1000,2000) | 2015 | 186,187ZW01 |
| Democratic Republic of the Congo | 44536 | 2022 | 92 | Increasing | ·· | ·· | ·· |  | 22·7 (7·8, 42·5) | 10000 (3500,19000) | 2015-17 | 188,189,190ZW01,191 |
| Djibouti | 750 | 2022 | 105 | Increasing | ·· | ·· | ·· |  | ·· | ·· | ·· |  |
| Equatorial Guinea | 500 | 2015 | 64 | Stable | ·· | ·· | ·· |  | ·· | ·· | ·· |  |
| Eritrea** | ·· | N/A | ·· | ·· | ·· | ·· | ·· |  | ·· | ·· | ·· |  |
| Eswatini | 3405 | 2022 | 468 | Decreasing | ·· | ·· | ·· |  | ·· | ·· | ·· |  |
| Ethiopia | 110000 | 2020 | 166 | Decreasing | 7·3 (5·6, 9·1) | 8000 (6000,10000) | 2016-22 | 192-195 | 2·3 (1·2, 3·9) | 2500 (1500,4500) | 2008-21 | 196-204,205ZW01,206,207ZW01,208-213 |
| Gabon | 5501 | 2024 | 394 | Increasing | ·· | ·· | ·· |  | ·· | ·· | ·· |  |
| Gambia | 543 | 2021 | 38 | Decreasing | ·· | ·· | ·· |  | ·· | ·· | ·· |  |
| Ghana | 14262 | 2024 | 73 | Decreasing | 16·9 (11·8, 22·7) | 2500 (1500,3000) | 2004-13 | 214-217 | 0·5 (0·0, 1·7) | < 500 (<500,<500) | 2014 | 218 |
| Guinea | 5549 | 2024 | 75 | Increasing | ·· | ·· | ·· |  | 2·5 (1·6, 3·5) | < 500 (<500,<500) | 2010 | 219 |
| Guinea-Bissau | 596 | 2017 | 58 | Increasing | ·· | ·· | ·· |  | ·· | ·· | ·· |  |
| Kenya | 60000 | 2023 | 193 | Stable | ·· | ·· | ·· |  | 10·2 (5·9, 15·5) | 6000 (3500,9500) | 2023 | 220JW01 |
| Lesotho | 2216 | 2019 | 162 | Stable | ·· | ·· | ·· |  | ·· | ·· | ·· |  |
| Liberia | 3000 | 2023 | 104 | Increasing | 13·3 (6·3, 22·2) | 500 (<500,500) | 2021 | 221 | ·· | ·· | ·· |  |
| Madagascar | 30530 | 2023 | 184 | Increasing | ·· | ·· | ·· |  | 11·4 (2·6, 24·5) | 3500 (1000,7500) | 2021 | 222ZW01 |
| Malawi | 16536 | 2024 | 154 | Stable | 3·5 (1·0, 7·3) | 500 (<500,1000) | 2005 | 223 | 1·0 (0·6, 1·6) | < 500 (<500,500) | 1997-2019 | 224-227 |
| Mali | 8670 | 2022 | 79 | Increasing | ·· | ·· | ·· |  | ·· | ·· | ·· |  |
| Mauritania | 2826 | 2022 | 112 | Increasing | ·· | ·· | ·· |  | ·· | ·· | ·· |  |
| Mauritius | 2755 | 2024 | 298 | Increasing | ·· | ·· | ·· |  | ·· | ·· | ·· |  |
| Mozambique | 22000 | 2024 | 128 | Increasing | ·· | ·· | ·· |  | 3·3 (3·0, 3·7) | 500 (500,1000) | 2022 | 228ZW02 |
| Namibia | 8900 | 2021/2022 | 589 | Increasing | ·· | ·· | ·· |  | ·· | ·· | ·· |  |
| Niger | 13005 | 2023 | 106 | Stable | ·· | ·· | ·· |  | ·· | ·· | ·· |  |
| Nigeria | 84011 | 2024 | 73 | Increasing | 18·1 (15·1, 21·4) | 15000 (12500,18000) | 2007-16 | 229,230 | 1·8 (0·2, 4·5) | 1500 (<500,3500) | 2006-15 | 231-234 |
| Congo | 1388 | 2019 | 45 | Stable | ·· | ·· | ·· |  | ·· | ·· | ·· |  |
| Rwanda | 87621 | 2024 | 1123 | Increasing | 4·3 (4·1, 4·5) | 4000 (3500,4000) | 2017 | 235 | ·· | ·· | ·· |  |
| Sao Tome & Principe | 300 | 2023 | 239 | Increasing | ·· | ·· | ·· |  | ·· | ·· | ·· |  |
| Senegal | 13185 | 2023 | 142 | Increasing | 13·9 (10·4, 17·9) | 2000 (1500,2500) | 2014 | 236 | ·· | ·· | ·· |  |
| Seychelles | 474 | 2024 | 646 | Decreasing | ·· | ·· | ·· |  | ·· | ·· | ·· |  |
| Sierra Leone | 4453 | 2024 | 92 | Stable | ·· | ·· | ·· |  | ·· | ·· | ·· |  |
| Somalia | 2799 | 2023 | 33 |  | ·· | ·· | ·· |  | ·· | ·· | ·· |  |
| South Africa | 157056 | 2023 | 405 | Stable | 3·2 (1·9, 4·8) | 5000 (3000,7500) | 2019 | 237 | 0·6 (0·5, 0·8) | 1000 (1000,1000) | 2012-18 | 238-241,242ZW01,243-247 |
| United Republic of Tanzania | 32671 | 2022 | 96 | Decreasing | 7·0 (4·7, 9·7) | 2500 (1500,3000) | 2007 | 248 | 3·4 (1·4, 6·6) | 1000 (500,2000) | 2009-22 | 249,250ZW01,251,252 |
| Togo | 4990 | 2021 | 102 | Stable | 10·8 (7·8, 14·3) | 500 (500,500) | 2013 | 236 | ·· | ·· | ·· |  |
| Uganda | 78539 | 2024 | 322 | Increasing | ·· | ·· | ·· |  | 0·9 (0·3, 1·8) | 500 (<500,1500) | 2008-14 | 253-255 |
| Zambia | 28225 | 2024 | 264 | Increasing | ·· | ·· | ·· |  | 1·8 (1·3, 2·4) | 500 (500,500) | 2005-19 | 256-259 |

| Zimbabwe | 20997 | 2024 | 236 | Stable | ·· | ·· | ·· |  | ·· | ·· | ·· |  |
| --- | --- | --- | --- | --- | --- | --- | --- | --- | --- | --- | --- | --- |

| **Middle East & North Africa** |  |  |  |  |  |  |  |  |  |  |  |  |
| --- | --- | --- | --- | --- | --- | --- | --- | --- | --- | --- | --- | --- |
| Algeria | 94749 | 2021 | 340 | Increasing | ·· | ·· | ·· |  | ·· | ·· | ·· |  |
| Bahrain | 3485 | 2017 | 310 | Increasing | ·· | ·· | ·· |  | ·· | ·· | ·· |  |
| Cyprus | 966 | 2024 | 112 | Increasing | ·· | ·· | ·· |  | ·· | ·· | ·· |  |
| Egypt | 120000 | 2022 | 177 | Stable | ·· | ·· | ·· |  | ·· | ·· | ·· |  |
| Iraq | 73715 | 2021 | 290 | Increasing | ·· | ·· | ·· |  | ·· | ·· | ·· |  |
| Israel | 19756 | 2023 | 371 | Decreasing | ·· | ·· | ·· |  | ·· | ·· | ·· |  |
| Jordan | 19140 | 2022 | 270 | Increasing | ·· | ·· | ·· |  | ·· | ·· | ·· |  |
| Kuwait | 5300 | 2024 | 168 | Increasing | ·· | ·· | ·· |  | ·· | ·· | ·· |  |
| Lebanon | 9254 | 2023 | 264 | Increasing | 2·3 (0·8, 4·6) | < 500 (<500,500) | 2008 | 260 | ·· | ·· | ·· |  |
| Libya | 19103 | 2023 | 428 | Increasing | 6·8 (6·2, 7·5) | 1500 (1000,1500) | 2006 | 261 | ·· | ·· | ·· |  |
| Morocco | 102653 | 2023 | 421 | Increasing | ·· | ·· | ·· |  | ·· | ·· | ·· |  |
| Oman | 1960 | 2015 | 62 | Increasing | ·· | ·· | ·· |  | ·· | ·· | ·· |  |
| Occupied Palestinian territories | ·· | N/A | ·· | ·· | ·· | ·· | ·· |  | ·· | ·· | ·· |  |
| Qatar | 2055 | 2022 | 92 | Increasing | ·· | ·· | ·· |  | ·· | ·· | ·· |  |
| Saudi Arabia | 68056 | 2017 | 280 | Increasing | ·· | ·· | ·· |  | ·· | ·· | ·· |  |
| South Sudan | 8400 | 2021 | 149 | Increasing | ·· | ·· | ·· |  | ·· | ·· | ·· |  |
| Sudan | 21000 | 2017 | 93 | Stable | ·· | ·· | ·· |  | ·· | ·· | ·· |  |
| Syrian Arab Republic | 10599 | 2004 | 102 | Decreasing | 2·5 (1·2, 4·3) | 500 (<500,500) | 2012 | 262 | ·· | ·· | ·· |  |
| Tunisia | 23484 | 2021 | 289 | Increasing | ·· | ·· | ·· |  | ·· | ·· | ·· |  |
| Türkiye | 371587 | 2024 | 643 | Increasing | 3·3 (1·8, 5·2) | 12500 (6500,19500) | 2008-17 | 263-266 | 0·1 (0·0, 0·2) | 500 (<500,1000) | 2001-11 | 267,268 |
| United Arab Emirates | 9826 | 2014 | 131 | Decreasing | ·· | ·· | ·· |  | ·· | ·· | ·· |  |
| Yemen | 4268 | 2022 | 23 | Decreasing | ·· | ·· | ·· |  | ·· | ·· | ·· |  |

**Notes:**

Ns are rounded to the nearest 500.

^1^ Country level data that informed these regional and global incarceration estimates were sourced from the World Prison Brief, collated by the Institute for Crime and Justice Policy Research at Burbeck University. See: https://www.prisonstudies.org/world-prison-brief-data. Note that we used the country estimates to make rates for 15-64 years (not the total country population), so our rates differ from the World Prison Brief estimates.

* Estimates of the prison population total range between 80,000 and 120,000.

† For reporting purposes, these countries or territories are reported separately due to differences in service provision.

‡ The following sources were found for HBV prevalence in Australian prisons, but due to the quality of the AusHep study^178^ we did not include them in the meta analysis: ^178,269-273^

·· Indicates that no estimates of the prevalence for that outcome were obtained for that country.

** No incarceration population estimate was available from the World Prison Brief, so no total estimates could be calculated for Eritrea.

¶ The direction of the trend was based on the most recent estimate, with an average change of at least 10% over the past five years considered indicative of a trend.

NK Indicates no evidence was located that injecting drug use was occurring in this country.

HBV - Hepatitis B.

Please see Appendix 5-8 for details of approach to assessment of study methodology and approach to selection and synthesis of data.

**References for Table 9.2**

1. Weilandt C, Stöver H, Eckert J, Grigoryan G. Anonymous survey on infectious diseases and related risk behaviour among Armenian prisoners and prison staff. *International Journal of Prisoner Health* 2007.

2. Azbel L, Wickersham JA, Wegman MP, et al. Burden of substance use disorders, mental illness, and correlates of infectious diseases among soon-to-be released prisoners in Azerbaijan. *Drug and Alcohol Dependence* 2015.

3. Handanagic S. Report on the Integrated Bio-behavioural Surveillance Surveys among Key Populations in Azerbaijan, 2015. In: Ministry of Health of Republic of Azerbaijan WHO, WHO Collaborating Centre for HIV Surveillance, Zagreb Croatia, editor.; 2015.

4. V. Kasumov AK, D. Makhmudova, F. Juzbashov, S. Hasiev, S. Babazade, R. Sultanova, G. Kasumova, N. Kerimova. PREVALENCE OF HIV, HEPATITIS AND SYPHILIS, AND BEHAVIOURAL RISK FACTORS AMONG MOST-AT-RISK GROUPS IN THE REPUBLIC OF AZERBAIJAN. In: CENTRE MOHOTROARA, editor.; 2008.

5. Ravlija J, Vasilj I, Marijanovic I, Vasilj M. RISK BEHAVIOUR OF PRISON INMATES IN RELATION TO HIV/STI. *Psychiatria Danubina* 2014.

6. Andreev V, Karcheva A, Petrova K, Lazarova E. Tuberculosis in prison. *European Respiratory Journal* 2011.

7. Klusonová H, Stĕpánová V, Cízek J, Plísková L. [Viral hepatitis in users of addictive drugs in the Czech Republic]. *Epidemiol Mikrobiol Imunol* 2004.

8. Aerts A, Habouzit M, Mschiladze L, et al. Pulmonary tuberculosis in prisons of the ex-USSR state Georgia: Results of a nation-wide prevalence survey among sentenced inmates. *International Journal of Tuberculosis and Lung Disease* 2000.

9. Treso B, Barcsay E, Tarjan A, et al. Prevalence and correlates of HCV, HVB, and HIV infection among prison inmates and staff, Hungary. *Journal of urban health : bulletin of the New York Academy of Medicine* 2012.

10. Pendzich J, Maksymowicz-Mazur W, Pawlowska J, et al. Tuberculosis among the homeless and inmates kept in custody and in penitentiary institutions in the Silesia region. *Pneumonologia i Alergologia Polska* 2015.

11. Nazare C, Girleanu I, Cojocariu-Salloum C, Trifan A. PREVALENCE OF CHRONIC HEPATITIS B VIRUS (HBV) INFECTION IN CLOSED COMMUNITIES AND RISK BEHAVIOUR. *MEDICAL-SURGICAL JOURNAL-REVISTA MEDICO-CHIRURGICALA* 2011.

12. Mahler B, De Vries G, Van Hest R, et al. Use of targeted mobile X-ray screening and computer-aided detection software to identify tuberculosis among high-risk groups in Romania: Descriptive results of the E-DETECT TB active case-finding project. *BMJ Open* 2021.

13. Slavuckij A, Sizaire V, Lobera L, Matthys F, Kimerling ME. Decentralization of the DOTS programme within a Russian penitentiary system: How to ensure the continuity of tuberculosis treatment in pre‐trial detention centres. *The European Journal of Public Health* 2002; **12**(2): 94-8.

14. Azbel L, Wickersham JA, Grishaev Y, Dvoryak S, Altice FL. Burden of infectious diseases, substance use disorders, and mental illness among Ukrainian prisoners transitioning to the community. *PLoS One* 2013.

15. Busschots D, Kremer C, Bielen R, et al. A multicentre interventional study to assess blood-borne viral infections in Belgian prisons. *BMC Infect Dis* 2021.

16. Burek V, Horvat J, Butorac K, Mikulić R. Viral hepatitis B, C and HIV infection in Croatian prisons. *Epidemiol Infect* 2010.

17. Vilibic-Cavlek T, Gjenero-Margan I, Retkovac B, et al. Sociodemographic characteristics and risk behaviors for HIV, hepatitis B and hepatitis C virus infection among Croatian male prisoners. *International Journal of Prisoner Health* 2011; **7**(1): 28-31.

18. Christensen PB, Krarup HB, Niesters HGM, Norder H, Georgsen J. Prevalence and incidence of bloodborne viral infections among Danish prisoners. *European Journal of Epidemiology* 2000.

19. Kirwan P, Evans B, Brant L, Sentinel Surveillance Hepatitis T. Hepatitis C and B testing in English prisons is low but increasing. *Journal of Public Health* 2011.

20. Morey S, Hamoodi A, Valappil M, et al. A universal offer of blood borne virus testing substantially increases diagnosis and treatment of hepatitis C in prisons. *Journal of Hepatology* 2018.

21. Phaw NA, Thant AM, Thompson C, et al. Prospective evaluation of the impact of repeated whole prison testing for hepatitis C. *BMJ Open Gastroenterol* 2025; **12**(1).

22. Rautanen M, Harald, K, & Tyni, S. Health and Wellbeing of Prisoners 2023 The Wattu IV Prison Population Study Finland, 2024.

23. Arrada A, Zbar OZD, Vasseur V. Prevalence of HBV and HCV infections and incidence of HCV infection after 3, 6 and 12 months detention in La Sante prison, Paris. *Annales de Medecine Interne* 2001.

24. Jacomet C, Guyot-Lénat A, Bonny C, et al. Addressing the challenges of chronic viral infections and addiction in prisons: the PRODEPIST study. *European Journal of Public Health* 2016.

25. Lelievre C, Prissette G, Reuche AA, et al. Detection of sexually transmitted infections at the Amiens prison. State of play from February 2019 to May 2019. *Revue de Medecine Legale* 2020.

26. Michault A, Faulques B, Sevadjan B, Troalen D, Marais A, Barau G. Prevalence of hepatitis A, B, C virus markers in Reunion (south hospital and Saint Pierre prison). [French]. *Bulletin de la Societe de pathologie exotique (1990)* 2000.

27. Verneuil L, Vidal JS, Bekolo RZ, et al. Prevalence and risk factors of the whole spectrum of sexually transmitted diseases in male incoming prisoners in France. *European Journal of Clinical Microbiology & Infectious Diseases* 2009.

28. Karabela S, Papaventsis, D., Georgoulas, S., Nikolaou, S., Ioannidis, P., Konstantinidou, E., Sainti, A., Marinou, I. and Kanavaki, S Epidemiological monitoring of pulmonary tuberculosis in a correctional facility population, Athens, Greece, 2005-2009: P2080 *Clinical Microbiology & Infection* 2010.

29. Drummond A, Codd M, Donnelly N, et al. Study on the prevalence of drug use, including intravenous drug use, and blood-borne viruses among the Irish prisoner population. *Dublin: National Advisory Committee on Drugs and Alcohol* 2014.

30. Babudieri S, Longo B, Sarmati L, et al. Correlates of HIV, HBV, and HCV infections in a prison inmate population: results from a multicentre study in Italy. *Journal of Medical Virology* 2005.

31. Brandolini M, Novati, S., De Silvestri A, Tinelli C, Patruno SFA, Ranieri R, Seminari E. Prevalence and epidemiological correlates and treatment outcome of HCV infection in an Italian prison setting. *BMC Public Health* 2013.

32. Fiore V, De Vito A, Rastrelli E, et al. Differences in HCV Seroprevalence, Clinical Features, and Treatment Outcomes between Female and Male Incarcerated Population: Results from a Matched Cohort Study. *Viruses* 2023; **15**(12).

33. Geremia N, Giovagnorio F, De Vito A, et al. HBV in Italian Women's Jail: An Underestimated Problem? *Journal of Clinical Medicine* 2024; **13(5) (no pagination)**.

34. Giuliani R, Casigliani V, Fornili M, et al. HCV micro-elimination in two prisons in Milan, Italy: A model of care. *Journal of Viral Hepatitis* 2020.

35. Scelza G, Amato A, Pagano AM, et al. Effect of hepatitis C antiviral therapy on oral lichen planus and hyposalivation in inmates. *Annals of Gastroenterology* 2022.

36. Muscat K, Cremona C, Fenech TM, Abela M, Padovese V. Sexually transmitted infections epidemiology and risk assessment at the main correctional facility in Malta (2017-2019). *Journal of the European Academy of Dermatology and Venereology* 2022.

37. Bakić M, Stevanović J, Milić M, et al. Factors associated with the prevalence of viral hepatitis B and C among prisoners: Results of two consecutive national surveys in Montenegro. *PLOS ONE* 2025; **20**(4): e0321464.

38. Schreuder I, van der Sande MA, Osterhaus AD, et al. No HIV infections despite high numbers of hepatitis B and C virus infections in Dutch prisoners. *Journal of Public Health and Epidemiology* 2011; **3**(6): 284-93.

39. Carvalhana S, Pinto R, Leitao J, et al. HCV and HBV prevalence in the population: Large disparity between hepatitis c in the general population, comparing with high risk groups. *United European Gastroenterology Journal* 2014.

40. Garcia A, Exposto F, Prieto E, Lopes M, Duarte A, da Silva RC. Association of Trichomonas vaginalis with sociodemographic factors and other STDs among females inmates in Lisbon. *International Journal of STD & AIDS* 2004.

41. Passadouro R. [Prevalence infections and risk factors due to HIV, Hepatitis B and C in a prison establishment in Leiria]. *Acta Med Portuguesa* 2004.

42. da Silva Marques NM, Margalho R, Melo MJ, da Cunha JGS, Melico-Silvestre AA. Seroepidemiological survey of transmissible infectious diseases in a Portuguese prison establishment. *Brazilian Journal of Infectious Diseases* 2011.

43. Cuadrado A, Llerena S, Cobo C, et al. Microenvironment Eradication of Hepatitis C: A Novel Treatment Paradigm. *American Journal of Gastroenterology* 2018.

44. Ferrer-Castro V, Crespo-Leiro MR, García-Marcos LS, et al. [Evaluation of needle exchange program at Pereiro de Aguiar prison (Ourense, Spain): ten years of experience]. *Revista Espanola de Sanidad Penitenciaria* 2012.

45. Vicente-Alcalde N, Tuells J, Egoavil CM, Ruescas-Escolano E, Altavilla C, Caballero P. Immunization Coverage of Inmates in Spanish Prisons. *International Journal of Environmental Research and Public Health* 2020.

46. de la Hoya PS, Marco A, Garcia-Guerrero J, Rivera A, Prevalhep Study G. Hepatitis C and B prevalence in Spanish prisons. *European Journal of Clinical Microbiology & Infectious Diseases* 2011.

47. Sanchez VM, Guerra JM, Cayla JA, Rodriguez JC, Blanco MD, Alcoba M. Incidence of tuberculosis and the importance of treatment of latent tuberculosis infection in a Spanish prison population. *International Journal of Tuberculosis and Lung Disease* 2001.

48. Gahrton C, Westman G, Lindahl K, et al. Prevalence of Viremic hepatitis C, hepatitis B, and HIV infection, and vaccination status among prisoners in Stockholm County. *BMC Infectious Dis*eases 2019.

49. Baggio S, Pala KC, Rieder JP, Tran NT, Wolff H, Getaz L. Infectious diseases in post-trial detention and comparisons with pre-trial detention: A study in Geneva, Switzerland. *Journal of Infection and Public Health* 2020.

50. Gétaz L, Casillas A, Siegrist CA, et al. Hepatitis B prevalence, risk factors, infection awareness and disease knowledge among inmates: a cross-sectional study in Switzerland's largest pre-trial prison. *Journal of Global Health* 2018.

51. Wolff H, Sebo P, Haller DM, et al. Health problems among detainees in Switzerland: a study using the ICPC-2 classification. *BMC Public Health* 2011.

52. Ritter C, Elger BS. Prevalence of positive tuberculosis skin tests during 5 years of screening in a Swiss remand prison. *International Journal of Tuberculosis and Lung Disease* 2012.

53. Tony Y, Jiang S, Guan X, et al. Epidemic situation of tuberculosis in prisons in the central region of China. *American Journal of Tropical Medicine and Hygiene* 2019.

54. Leung CC, Chan CK, Tam CM, et al. Chest radiograph screening for tuberculosis in a Hong Kong prison. *International Journal of Tuberculosis and Lung Disease* 2005.

55. Arends RM, Nelwan EJ, Soediro R, et al. Associations between impulsivity, risk behavior and HIV, HBV, HCV and syphilis seroprevalence among female prisoners in Indonesia: A cross-sectional study. *PLoS One* 2019.

56. Nelwan EJ, Van Crevel R, Alisjahbana B, et al. Human immunodeficiency virus, hepatitis B and hepatitis C in an Indonesian prison: prevalence, risk factors and implications of HIV screening. *Tropical Medicine & International Health* 2010.

57. Niode NJ, Raranta H, Purwanto DS, Mamuaja EH, Tallei TE. The prevalence and risk factors of sexually transmitted infections among correctional institution inmates in Manado, Indonesia. *Journal of Pakistan Association of Dermatologists* 2024; **34(2)**: 445-52.

58. Prasetyo AA, Dirgahayu P, Sari Y, Hudiyono H, Kageyama S. Molecular epidemiology of HIV, HBV, HCV, and HTLV-1/2 in drug abuser inmates in central Javan prisons, Indonesia. *Journal of Infection in Developing Countries* 2013.

59. Rey I, Saragih R, Effendi-Ys R, Sembiring J, Siregar G, Zain L. Profile of hepatitis B and C virus infection in prisoners in Lubuk Pakam correctional facilities. IOP Conference Series: Earth and Environmental Science; 2018: IOP Publishing; 2018. p. 012033.

60. Aurelia, Kamaludin, Muslimin C, et al. Comprehensive Tuberculosis Screening and Treatment at a Prison in Central Papua Province, Indonesia. *Tropical Medicine and Infectious Disease* 2024; **9**(10): 241.

61. Ong-Chu MC, Lao-Tan JY, Gabriel EA. Prevalence of hepatitis B and C and risk factors among prison inmates in Cebu, Philippines. *Hepatology International* 2016.

62. Simbulan NP, Aguilar AS, Flanigan T, Cu-Uvin S. High-risk behaviors and the prevalence of sexually transmitted diseases among women prisoners at the women state penitentiary in Metro Manila. *Soc Sci Med* 2001.

63. Chiang CY, Hsu CJ, Hsu PK, Suo J, Lin TP. Pulmonary tuberculosis in the Taiwanese prison population. *Journal of the Formosan Medical Association* 2002.

64. Harnpariphan W, Han WM, Supanun R, et al. High Proportion of Blood-Borne and Sexually Transmitted Infections Among People Deprived of Liberty in a Central Male Prison in Thailand: A Cross-Sectional Study 2018–2019. *AIDS Research and Human Retroviruses* 2022; **38**(5): 370-7.

65. Buangoen A, Ingviya T. Characteristics and Xpert MTB/RIF assay results of prisoners with pulmonary tuberculosis, Songkhla Province, southern Thailand. *Journal of the Medical Association of Thailand* 2020.

66. Jittimanee S, Namonta A, Charuenporn C. Systematic TB screening using WHO radiograph categorisation and care outcomes. *International Journal of Tuberculosis And Lung Disease* 2022.

67. Jittimanee SX, Ngamtrairai N, White MC, Jittimanee S. A prevalence survey for smear-positive tuberculosis in Thai prisons. *International Journal of Tuberculosis and Lung Disease* 2007.

68. Morasert T, Worapas W, Kaewmahit R, Uphala W. Prevalence and risk factors associated with tuberculosis disease in Suratthani Central Prison, Thailand. *International Journal of Tuberculosis And Lung Disease* 2018.

69. Rodgerd A, Morasert T. Effectiveness of Systematic Screening and Treatment of Tuberculosis in Prison in Thailand. *American Journal of Tropical Medicine and Hygiene* 2024; **111**(5): 1041 EP-5.

70. Sretrirutchai S, Silapapojakul K, Palittapongarnpim P, Phongdara A, Vuddhakul V. Tuberculosis in Thai prisons: magnitude, transmission and drug susceptibility. *International Journal of Tuberculosis and Lung Disease* 2002.

71. Banu S, Rahman MT, Uddin MKM, et al. Effect of active case finding on prevalence and transmission of pulmonary tuberculosis in Dhaka Central Jail, Bangladesh. *PLoS One* 2015.

72. Banu S, Hossain A, Uddin MKM, et al. Pulmonary tuberculosis and drug resistance in Dhaka central jail, the largest prison in Bangladesh. *PLoS One* 2010.

73. Islam MR, Khatun R, Uddin MKM, et al. Yield of Two Consecutive Sputum Specimens for the Effective Diagnosis of Pulmonary Tuberculosis. *PLoS One* 2013.

74. National AIDS Control Organization. HIV Sentinel Surveillance Plus 2021, Central Prison Sites, 2022.

75. Ramamoorthy M, Venketeswaran A, Seenivasan P, et al. Risk factors and prevalence, hepatitis B virus and hepatitis C virus among prison inmates, Chennai, India, 2015. 2016; **53**: 90.

76. Rana S, Girdgar N, Gill MK, Kumar AJIJRMS. Prevalence of hepatitis-B surface antigen among population of inmates in Tihar Jail, New Delhi. 2015; **3**: 100-4.

77. Bhatnagar T, Ralte M, Ralte L, Chawnglungmuana, Sundaramoorthy L, Chhakchhuak L. Intensified tuberculosis and HIV surveillance in a prison in Northeast India: Implementation research. *PLoS One* 2019.

78. Dolla CK, Dhanraj B, Malaisamy M, et al. Burden of pulmonary tuberculosis in modern prison: A cross sectional prevalence survey from south India. *Indian Journal of Tuberculosis* 2019.

79. Kosambiya JK, Vadgama P, Samudyatha UC, Rathod D, Buch R, Damor R. Active case finding of pulmonary tuberculosis and HIV infection among prisoners of South Gujarat: A cross sectional study. *Indian Journal of Tuberculosis* 2022.

80. Prasad BM, Thapa B, Chadha SS, et al. Status of Tuberculosis services in Indian Prisons. *International Journal of Infectious Diseases* 2017.

81. Ghafari S, Sharifzadeh G, Jamali S, Taji B, Javadmoosavi SY, Ziaee M. Prevalence of Hepatitis B and C among Drug-Abusing Male Prisoners in Birjand, South Khorasan, Iran. *Archives of Iranian Medicine* 2019.

82. Javadi A, Pourahmad M, Ataei B. The relationship between frequency and duration of imprisonment and the prevalence of HBsAg, AntiHCV and HIV antibody seropositivity in Iranian prisoners. 2006.

83. Khademi N, Shakiba E, Khodadost M, Khoramdad M. Seroprevalence and related risk behaviors of hepatitis C, hepatitis B and HIV infections among Male prisoners in Kermanshah, Iran. *Archives of Iranian Medicine* 2019.

84. Khajedaluee M, Babaei A, Vakili R, et al. Sero-prevalence of bloodborne tumor viruses (HCV, HBV, HTLV-I and KSHV infections) and related risk factors among prisoners in Razavi Khorasan province, Iran, in 2008. *Hepatitis Monthly* 2016.

85. Moradi G, Gouya MM, Zavareh FA, et al. Prevalence and risk factors for HBV and HCV in prisoners in Iran: a national bio-behavioural surveillance survey in 2015. *Tropical Medicine & International Health* 2018.

86. Moradi G, Jafari S, Zarei B, et al. Prevalence and Risk Factors for Hepatitis B and Hepatitis C Exposure in Iranian Prisoners: A National Study in 2016. *Hepatitis Monthly* 2019.

87. Nokhodian Z, Yazdani MR, Yaran M, et al. Prevalence and risk factors of HIV, syphilis, hepatitis B and C among female prisoners in Isfahan, Iran. *Hepatitis Monthly* 2012.

88. Salem F, Hekmat S, Aghasadeghi MR, Javadi F, Gholami H, Mostafavi E. Prevalence and Risk Factors of Hepatitis B Virus Genotype D Amongst Inmates in Alborz Province, Iran: A Cross-Sectional Survey. *Jundishapur Journal of Microbiology* 2013.

89. Ziaee M, Sharifzadeh G, Namaee MH, Fereidouni M. Prevalence of HIV and Hepatitis B, C, D Infections and Their Associated Risk Factors among Prisoners in Southern Khorasan Province, Iran. *Iranian Journal of Public Health* 2014.

90. Assefzadeh M, Barghi RG, Shahidi SS. Tuberculosis case-finding and treatment in the central prison of Qazvin province, Islamic Republic of Iran. *Eastern Mediterranean Health Journal* 2009.

91. Farhoudi B, Alinaghi SAS, Hosseini M, et al. Prevalence of tuberculosis in a prison in tehran by active case finding. *Infectious Disorders - Drug Targets* 2019.

92. Moosazadeh M, Amiresmaili MR, Parsaei MR, Ahmadi M, Jalahi H. Prevalence of Tuberculosis Among the Prisoners of Mazandaran. *Rums Journal* 2011; **10**(4): 309-16.

93. mohammad reza N, siavash V, alireza J. The prevalence of smears positive pulmonary tuberculosis in a prison. *Yafteh* 2015; **17**(1): 15-27.

94. Seyedalinaghi S, Farhoudi B, Najafi Z, Jafari S, Shahbazi M. Comparing Tuberculosis Incidence in a Prison with the Society, Tehran, Iran. *Archives of Clinical Infectious Diseases* 2018.

95. Khanal S, Baral S, Shrestha P, et al. Yield of intensified tuberculosis case-finding activities using Xpert® MTB/RIF among risk groups in Nepal. *Public Health Action* 2016; **6**(2): 136-41.

96. Shrestha G, Yadav DK, Gautam R, Mulmi R, Baral D, Pokharel PK. Pulmonary tuberculosis among male inmates in the largest prison of Eastern Nepal. *Tuberculosis research and treatment* 2019; **2019**(1): 3176167.

97. Fayyaz M, Qazi M, Ishaq M, Chaudhry G, Bukhari MJB. Frequency of hepatitis B and C seropositivity in prisoners. 2006; **22**: 55-8.

98. Kazi AM, Shah SA, Jenkins CA, Shepherd BE, Vermund SH. Risk factors and prevalence of tuberculosis, human immunodeficiency virus, syphilis, hepatitis B virus, and hepatitis C virus among prisoners in Pakistan. *International Journal of Infectious Diseases* 2010.

99. Khan MA, Ayub A, Ayub H, Shafique M, Rahman JA. A comparative study of Hepatitis B and C prevalence using ICT and elisa method in jail inmates. *Pakistan Journal of Medical and Health Sciences* 2017.

100. Memon AR, Shafique K, Memon A, Draz AU, Rauf MUA, Afsar S. Hepatitis B and C prevalence among the high risk groups of Pakistani population. A cross sectional study. *Archives of Public Health* 2012.

101. Jamal W, Azeemi K, Waqar M, Ikram K, Zaidi SA, Habib S. Active case finding for tuberculosis among prisoners in Karachi, Pakistan. Eur Respiratory Soc; 2019.

102. Kakar N, Abbas F, Shafee M, Asmat T. Study on Accuracy and Efficiency of Molecular Diagnostic Techniques used for Tuberculosis and Analysis of Associated Risk Factors for Tuberculosis in Jail Inmates of Quetta, Pakistan. *Pakistan Journal of Zoology* 2018.

103. Rao NA. Prevalence of pulmonary tuberculosis in Karachi central prison. *Journal of the Pakistan Medical Association* 2004.

104. Niriella MA, Hapangama A, Luke H, Pathmeswaran A, Kuruppuarachchi K, de Silva HJ. Prevalence of hepatitis B and hepatitis C infections and their relationship to injectable drug use in a cohort of Sri Lankan prison inmates. *Ceylon Medical Journal* 2015.

105. Azbel L, Polonsky M, Wegman M, et al. Intersecting epidemics of HIV, HCV, and syphilis among soon-to-be released prisoners in Kyrgyzstan: Implications for prevention and treatment. *International Journal of Drug Policy* 2016.

106. Winetsky DE, Almukhamedov O, Pulatov D, Vezhnina N, Dooronbekova A, Zhussupov B. Prevalence, risk factors and social context of active pulmonary tuberculosis among prison inmates in Tajikistan. *PLoS One* 2014.

107. Adaszko D, Sotelo J, Orlando M, Adaszko A, Angeleri P. HIV, hepatitis B and C, syphilis and Tuberculosis prevalence in people deprived of liberty for criminal reasons in Argentina. Final results of a national study. *International Journal of Infectious Diseases* 2018; **73**: 202-.

108. Mendizabal M, Testa P, Rojas M, et al. Pilot study using the ECHO model to enhance linkage to care for patients with hepatitis C in the custodial setting. *Journal of Viral Hepatitis* 2020.

109. Villarroel-Torrico M, Montaño K, Flores-Arispe P, et al. Syphilis, human immunodeficiency virus, herpes genital and hepatitis B in a women's prison in Cochabamba, Bolivia: prevalence and risk factors. *Revista Española de Sanidad Penitenciaria* 2018.

110. Villarroel-Torrico M, Montano K, Flores-Arispe P, et al. Syphilis, human immunodeficiency virus, herpes genital and hepatitis B in a women's prison in Cochabamba, Bolivia: prevalence and risk factors. *Revista Espanola de Sanidad Penitenciaria* 2018.

111. Barros LAS, Pessoni GC, Teles SA, et al. Epidemiology of the viral hepatitis B and C in female prisoners of Metropolitan Regional Prison Complex in the State of Goias, Central Brazil. *Revista da Sociedade Brasileira de Medicina Tropical* 2013.

112. Benedetti MSG, Nogami ASA, da Costa BB, et al. Sexually transmitted infections in women deprived of liberty in Roraima, Brazil. *Revista de Saude Publica* 2020.

113. Catalan-Soares BC, Almeida RT, Carneiro-Proietti AB. Prevalence of HIV-1/2, HTLV-I/II, hepatitis B virus (HBV), hepatitis C virus (HCV), Treponema pallidum and Trypanosoma cruzi among prison inmates at Manhuacu, Minas Gerais State, Brazil. *Revista da Sociedade Brasileira de Medicina Tropical* 2000.

114. Cossetin Costa M, Carneiro Mussi F, da Silva Pires CG, D'Almeida Miranda FM, Mantovani MdF. INFECÇÕES SEXUALMENTE TRANSMISSÍVEIS EM PESSOAS PRIVADAS DE LIBERDADE: AS GRADES COMO LIMITANTES À SAÚDE. *Revista de Enfermagem e Atenção à Saúde (REAS)* 2024; **13**(3): 1-11.

115. Ferreto LED, Follador FAC, Coelho HC, et al. Prevalence and risk factors for hepatitis B infection in men in the penitentiary system in Parana, Brazil. *Journal of Viral Hepatitis* 2018.

116. Guimarães T, Granato CF, Varella D, Ferraz ML, Castelo A, Kallás EG. High prevalence of hepatitis C infection in a Brazilian prison: identification of risk factors for infection. The *Brazilian Journal of Infectious Diseases* 2001.

117. Machado F, Becker D, de Oliveira CF, Possuelo LG, Renner JDP. Seroprevalence of HIV, hepatitis B and C and syphilis infection in prisoners of the central region of Rio Grande do Sul, Brazil. *Mundo da Saude* 2019.

118. Miranda AE, Vargas, P.M., Louis, M.E.S. & Viana, M.C. Sexually transmitted diseases among female prisoners in Brazil: prevalence and risk factors. *Sexually Transmitted Diseases* 2000; **27**(9): 491-5.

119. Prates Fonseca CE, Tupinambás U. Epidemiological profile of cases of HIV, Syphilis and Hepatitis in private of freedom, Minas Gerais. *Saúde Coletiva* 2023; **13**(88): 13381-8.

120. Rezende GR, Lago BV, Puga MA, et al. Prevalence, incidence and associated factors for HBV infection among male and female prisoners in Central Brazil: A multicenter study. *International Journal of Infectious Diseases* 2020.

121. Stief ACF, Martins RMB, de Andrade SMO, et al. Seroprevalence of hepatitis b virus infection and associated factors among prison inmates in state of mato grosso do sul, Brazil. *Revista da Sociedade Brasileira de Medicina Tropical* 2011.

122. de Gois JG, Guedes SJKO, Vieira AP, et al. Seroprevalence and factors associated with hepatitis B virus exposure in the incarcerated population from southern Brazil. *PLoS One* 2022.

123. De Sena Silva AA, De Araújo TME, Teles SA, De Lima Brito Magalhães R, Andrade ELR. Prevalence of Hepatitis B and associated factors in prisoners. *Acta Paulista de Enfermagem* 2017; **30**(1): 66-72.

124. do Nascimento CT, Pena DZ, Giuffrida R, et al. Prevalence and epidemiological characteristics of inmates diagnosed with infectious diseases living in a region with a high number of prisons in Sao Paulo state, Brazil. *BMJ Open* 2020.

125. Abrahao R, Nogueira PA, Malucelli MIC. Tuberculosis in county jail prisoners in the western sector of the city of Sao Paulo, Brazil. *International Journal of Tuberculosis and Lung Disease* 2006; **10**(2): 203-8.

126. Abrahao RMCM, Nogueira PA, Malucelli MIC. Tuberculosis in county jail prisoners in the western sector of the city of Sao Paulo, Brazil. *International Journal of Tuberculosis and Lung Disease* 2006.

127. Carbone ADS, Paiao DSG, Sgarbi RVE, et al. Active and latent tuberculosis in Brazilian correctional facilities: a cross-sectional study. *BMC Infectious Diseases* 2015.

128. Estevan AO, de Oliveira SMVL, Croda J. Active and latent tuberculosis in prisoners in the Central-West Region of Brazil. *Revista da Sociedade Brasileira de Medicina Tropical* 2013.

129. Kuhleis D, Ribeiro AW, Dalla Costa ER, et al. Tuberculosis in a southern Brazilian prison. *Memorias do Instituto Oswaldo Cruz* 2012.

130. Kuhleis D, Ribeiro AW, Costa ERD, et al. Tuberculosis in a southern Brazilian prison. *Memorias do Instituto Oswaldo Cruz* 2012; **107**(7): 909-15.

131. Leal M, Kerr L, Mota RMS, Neto RDP, Seal D, Kendall C. Health of female prisoners in Brazil. *Ciencia & Saude Coletiva* 2022.

132. Moreira FMF, Verma R, dos Santos PCP, et al. Blood-based host biomarker diagnostics in active case finding for pulmonary tuberculosis: A diagnostic case-control study. *eClinicalMedicine*2021; **33**.

133. Nogueira PA, Abrahao RMCDM, Galesi VMN. Tuberculosis in prison system - Survey in two prisons in the State of Sao Paulo, Brazil, 2008. *American Journal of Respiratory and Critical Care Medicine Conference: American Thoracic Society International Conference, ATS* 2010.

134. Nogueira PA, Abrahao RMCM, Galesi VMN. Tuberculosis and latent tuberculosis in prison inmates. *Revista de Saude Publica* 2012.

135. Nogueira PA, Abrahao R, Galesi VMN. Tuberculosis and latent tuberculosis in prison inmates. *Revista de Saude Publica* 2012; **46**(1): 119-27.

136. Heloisa da Silveira Paro Pedro SMTN, Maria Izabel Ferreira Pereira, Maria do Rosário Assad Goloni, Fernanda Carina Pires, Fernanda Modesto Tolentino, Rosangela Siqueira Oliveira, Andrea Regina Baptista Rossit. Mycobacterium tuberculosis detection in the penitentiary system. *Revista de Patologia Tropical/Journal of Tropical Pathology,* 2011; **40**(4): 287-2.

137. Pelissari DM, Kuhleis DC, Bartholomay P, et al. Prevalence and screening of active tuberculosis in a prison in the South of Brazil. *International Journal of Tuberculosis and Lung Disease* 2018.

138. Pereira CC, Borges TS, Daronco A, et al. Prevalence of Respiratory Symptoms and Active Tuberculosis in a Prison in the South of Brazil. *Revista de Epidemiologia e Controle de Infeccao* 2013.

139. Pivetta de Araujo RC, Martinez L, da Silva Santos A, et al. Serial Mass Screening for Tuberculosis Among Incarcerated Persons in Brazil. *Clinical Infectious Diseases* 2024; **78**(6): 1669-76.

140. Reis AJ, de David SMM, Nunes LD, Valim ARD, Possuelo LG. Recent transmission of drug-resistant Mycobacterium tuberculosis in a prison population in southern Brazil. *Jornal Brasileiro de Pneumologia* 2016; **42**(4): 286-9.

141. Rocha JZ, Valenca MS, Carrion LL, Silva LV, von Groll A, Silva PA. Respiratory symptoms and active tuberculosis in a prison in Southern Brazil: Associated epidemiologic variables. *Revista de Epidemiologia e Controle de Infeccao* 2013.

142. Salindri AD, Bampi JVB, Goncalves IB, et al. Tuberculosis Active Case Finding. *American Journal of Respiratory and Critical Care Medicine* 2024; **209**.

143. Sanchez A, Massari V, Gerhardt G, et al. X ray screening at entry and systematic screening for the control of tuberculosis in a highly endemic prison. *BMC Public Health* 2013; **13**: 1-7.

144. Sanchez A, Gerhardt G, Natal S, et al. Prevalence of pulmonary tuberculosis and comparative evaluation of screening strategies in a Brazilian prison. *International Journal of Tuberculosis and Lung Disease* 2005.

145. Sanchez A, Larouze B, Espinola AB, et al. Screening for tuberculosis on admission to highly endemic prisons? The case of Rio de Janeiro State prisons. *International Journal of Tuberculosis and Lung Disease* 2009.

146. Sánchez AR, Massari W, Gerhardt G, et al. Tuberculosis in Rio de Janeiro prisons, Brazil:: an urgent public health problem. *Cadernos de Saude Publica* 2007; **23**(3): 545-52.

147. Santos AD, de Oliveira RD, Lemos EF, et al. Yield, Efficiency, and Costs of Mass Screening Algorithms for Tuberculosis in Brazilian Prisons. *Clinical Infectious Diseases* 2021; **72**(5): 771-7.

148. Santos AdS, Oliveira RDd, Lemos EF, et al. Yield, Efficiency, and Costs of Mass Screening Algorithms for Tuberculosis in Brazilian Prisons. *Clinical Infectious Diseases* 2021.

149. Soares TR, de Oliveira RD, Liu YE, et al. Evaluation of chest X-ray with automated interpretation algorithms for mass tuberculosis screening in prisons: A cross-sectional study. *Lancet Regional Health-Americas* 2023.

150. Valença MS, Scaini JL, Abileira FS, Gonçalves CV, von Groll A, Silva PE. Prevalence of tuberculosis in prisons: risk factors and molecular epidemiology. *International Journal of Tuberculosis and Lung Disease* 2015.

151. Valenca MS, Cezar-Vaz MR, Brum CB, da Silva PEA. The process of detection and treatment of cases of tuberculosis in a prison. *Ciencia & Saude Coletiva* 2016; **21**(7): 2111-22.

152. Vieira AA, Ribeiro SA, de Siqueira AM, Galesi VMN, dos Santos LAR, Golub JE. Prevalence of patients with respiratory symptoms through active case finding and diagnosis of pulmonary tuberculosis among prisoners and related predictors in a jail in the city of carapicuiba, Brazil. *Revista Brasileira de Epidemiologia* 2010.

153. Warsinske HC, Rao AM, Moreira FMF, et al. Assessment of Validity of a Blood-Based 3-Gene Signature Score for Progression and Diagnosis of Tuberculosis, Disease Severity, and Treatment Response. *JAMA network open* 2018.

154. de Navarro PD, de Almeida IN, Kritski AL, et al. Prevalence of latent Mycobacterium tuberculosis infection in prisoners. *Jornal Brasileiro de Pneumologia* 2016; **42**(5): 348-55.

155. Sanchez-Vanegas G, Rodriguez-Vallejo D, Pinzon-Duran AC, Reina-Cifuentes MA, Monterrosa-Blanco A, Tiga-Segura JA. Prevalence of syphilis, hepatitis B and human immunodeficiency virus in the male prison population in Bogota, Colombia in 2019. [Spanish]. *Infectio* 2020.

156. Alarcón-Robayo JF, Martinez-Casallas L, Samir-Sánchez M, Valderrama-Mendoza JS, Bados-Enriquez DM, Jiménez-Canizales CE. Prevalencia de tuberculosis pulmonar en población privada de la libertad de 10 centros penitenciarios en Colombia, 2013. *Acta Médica Peruana* 2016; **33**: 202-7.

157. Castañeda-Hernández DM, Martínez-Ramírez JE, Bolivar-Mejía A, Rodríguez-Morales AJ. Differences in TB incidence between prison and general populations, Pereira, Colombia, 2010-2011. *Tuberculosis (Edinb)* 2013.

158. Guerra J, Mogollon D, Gonzalez D, et al. Active and latent tuberculosis among inmates in La Esperanza prison in Guaduas, Colombia. *PLoS One* 2019.

159. Rueda ZV, Lopez L, Velez LA, et al. High incidence of tuberculosis, low sensitivity of current diagnostic scheme and prolonged culture positivity in four Colombian prisons. A cohort study. *PLoS One* 2013.

160. Alvarado-Esquivel C, Sablon E, Martínez-García S, Estrada-Martínez S. Hepatitis virus and HIV infections in inmates of a state correctional facility in Mexico. *Epidemiology & Infection* 2005.

161. Bautista-Arredondo S, González A, Servan-Mori E, et al. A Cross-Sectional Study of Prisoners in Mexico City Comparing Prevalence of Transmissible Infections and Chronic Diseases with That in the General Population. *PLoS One* 2015.

162. Belaunzaran-Zamudio PF, Mosqueda-Gomez JL, Macias-Hernandez A, Rodríguez-Ramírez S, Sierra-Madero J, Beyrer C. Burden of HIV, Syphilis, and Hepatitis B and C Among Inmates in a Prison State System in Mexico. *AIDS Research and Human Retroviruses* 2017.

163. Gonzalez CAM, Ortiz BES, Aguilar MB, Gonzalez JDM. Risk factors and the seroprevalence of viral markers of hepatitis B (HVB) and hepatitis C (HCV) in high-risk groups in Chiapas. *Medwave* 2011.

164. Geadas C, Calderon RI, Yuen CM, et al. Active case-finding for TB among incarcerated women in Peru. *International Journal Of Tuberculosis And Lung Disease* 2023; **27**(10): 784 EP-6.

165. Jimenez J, Millones AK, Puma D, et al. Active search for tuberculosis in three youth detention centers in Peru. *Revista Peruana de Medicina Experimental y Salud Publica* 2025; **41**(4): 417-21.

166. Alcivar JC, Zambrano MM, Madronero MG, et al. Sexually transmitted infections in inmates in Merida Venezuela. *Investigacion Clinica* 2020.

167. Whitten C, Turner A, Howell B, Sparkes B, Ricciardelli R, Daley P. Retrospective review of rates of sexually transmitted and blood-borne infection (STBBI) testing in provincial corrections facilities in Newfoundland and Labrador. *JAMMI: Journal of the Association of Medical Microbiology & Infectious Disease Canada* 2023; **8**(2): 141-9.

168. Baillargen J, Snyder N, Soloway RD, et al. Hepatocellular Carcinoma Prevalence and Mortality in a Male State Prison Population. *Public Health Reports* 2009.

169. Hennessey KA, Kim AA, Griffin V, Collins NT, Weinbaum CM, Sabin K. Prevalence of infection with hepatitis B and C viruses and co-infection with HIV in three jails: a case for viral hepatitis prevention in jails in the United States. *Journal of Urban Health* 2009.

170. Irvin R, Landry G, Jones MR, et al. High prevalence of hepatitis C virus infection among incarcerated persons: Results from the Louisiana Hepatitis C Elimination Plan's opt-out testing program in prisons. *Journal of Viral Hepatitis* 2024; **31**(7): 432-5.

171. Khan AJ, Simard EP, Bower WA, et al. Ongoing transmission of hepatitis B virus infection among inmates at a state correctional facility. *American Journal of Public Health* 2005.

172. Lincoln T, Tuthill RW, DePietro SL. Viral hepatitis, risk behaviors, aminotransferase levels, and screening options at a county correctional center. *Journal of Correctional Health Care* 2006.

173. Macalino GE, Vlahov D, Sanford-Colby S, et al. Prevalence and Incidence of HIV, Hepatitis B Virus, and Hepatitis C Virus Infections Among Males in Rhode Island Prisons. [References]. *American Journal of Public Health* 2004.

174. Solomon L, Flynn C, Muck K, Vertefeuille J. Prevalence of HIV, syphilis, hepatitis B, and hepatitis C among entrants to Maryland correctional facilities. *Journal of Urban Health* 2004.

175. Hung R, Shelton S, Rischitelli G. Risk factors for tuberculosis conversion in a state prison. *McGill Journal of Medicine* 2002.

176. Nduaguba IP, Brannan G, Shubrook J. Evaluation of identifying tuberculosis infection and disease in a rural institutionalized population. *Osteopathic Family Physician* 2010.

177. Risser WL, Smith KC. Tuberculosis in incarcerated youth in Texas [4]. *JAMA* 2005; **293**(22): 2716 EP-7.

178. Bah R, Sheehan Y, Li X, et al. Prevalence of blood-borne virus infections and uptake of hepatitis C testing and treatment in Australian prisons: the AusHep study. *The Lancet Regional Health–Western Pacific* 2024; **53**.

179. Rapid assessment of tuberculosis in a large prison system--Botswana, 2002 A1 - Anonymous. *Morbidity and Mortality Weekly Report* 2003; **52**(12): 250 EP-2.

180. Wang EA. Rapid assessment of tuberculosis in a large prison system--Botswana, 2002. *Morbidity and Mortality Weekly Report* 2003.

181. Diendéré EA, Tiéno H, Bognounou R, et al. Prevalence and risk factors associated with infection by human immunodeficiency virus, hepatitis B virus, syphilis and bacillary pulmonary tuberculosis in prisons in Burkina Faso. *Med Trop (Mars)* 2011.

182. Kowo MP, Andoulo FA, Sizimboue DT, et al. Seroprevalence of hepatitis B and associated factors among inmates: a cross sectional study in the Douala New Bell Prison, Cameroon. *Pan African Medical Journal* 2021.

183. Donkeng-Donfack VF, Tchatchueng-Mbougua JB, Abanda NN, et al. A cost-benefit algorithm for rapid diagnosis of tuberculosis and rifampicin resistance detection during mass screening campaigns. *BMC Infectious Diseases* 2022.

184. Noeske J, Kuaban C, Amougou G, Piubello A, Pouillot R. Pulmonary tuberculosis in the Central Prison of Douala, Cameroon. *East Africa Medical Journal* 2006.

185. Noeske J, Ndi N, Mbondi S. Controlling tuberculosis in prisons against confinement conditions: a lost case? Experience from Cameroon. *International Journal of Tuberculosis and Lung Disease* 2011.

186. Seri B, Koffi A, Danel C, et al. Prevalence of pulmonary tuberculosis among prison inmates: A cross-sectional survey at the Correctional and Detention Facility of Abidjan, Cote d'Ivoire. *PLoS One* 2017.

187. Séri B, Koffi A, Danel C, et al. Prevalence of pulmonary tuberculosis among prison inmates: A cross-sectional survey at the Correctional and Detention Facility of Abidjan, Côte d'Ivoire. *PLoS One* 2017; **12**(7): e0181995.

188. Kalonji GMP, Connick GD, Ngongo LO, et al. Prevalence of tuberculosis and associated risk factors in the central prison of Mbuji-Mayi, DEmocratic republic of Congo. *Tropical Medicine and Health* 2016.

189. Kalonji GMP, Ngongo Okenge L, Ilunga-Ilunga F, Albert A, Giet D. [Factors associated with prison survival: Study in the Democratic Republic of Congo]. *Sante Publique* 2019.

190. Kaswa MK, Bakaswa GN, Boelaert M. Outbreak investigation of tuberculosis and multidrug-resistant tuberculosis in the central prison of Mbuji-Mayi the diamond capital of the Democratic Republic of Congo. *Tropical Medicine & International Health* 2015; **20**: 198-.

191. Kayomo MK, Hasker E, Aloni M, et al. Outbreak of tuberculosis and multidrug-resistant tuberculosis, Mbuji-Mayi central prison, democratic Republic of the Congo. *Emerging Infectious Diseases* 2018.

192. Kassa Y, Million Y, Biset S, Moges F. Hepatitis b and hepatitis c viral infections and associated factors among prisoners in northeast ethiopia. *Journal of Blood Medicine* 2021.

193. Kebede W, Abdissa A, Seid Y, Mekonnen Z. Seroprevalence and risk factors of hepatitis B, hepatitis C and HIV infections among prisoners in Jimma Town, Southwest Ethiopia. *Asian Pacific Journal of Tropical Disease* 2017.

194. Tadesse K, Ayalew G, Million Y, Gelaw A. Hepatitis B and hepatitis C virus infections and associated factors among prisoners in Gondar City, Northwest Ethiopia. *PLOS One* 2024; **19**(4): e0301973.

195. Tsegay B, Gebrecherkos T, Kahsay AG, Abdulkader M. Seroprevalence and Associated Factors of Hepatitis B and Hepatitis C Viral Infections Among Prisoners in Tigrai, Northern Ethiopia. *Infection and Drug Resistance* 2023; **16**: 3743-50.

196. Abebe DS, Bjune G, Ameni G, Biffa D, Abebe F. Prevalence of pulmonary tuberculosis and associated risk factors in Eastern Ethiopian prisons. *International Journal of Tuberculosis and Lung Disease* 2011.

197. Adane K, Spigt M, Winkens B, Dinant GJ. Tuberculosis case detection by trained inmate peer educators in a resource-limited prison setting in Ethiopia: a cluster-randomised trial. *The Lancet Global Health* 2019.

198. Adane K, Spigt M, Ferede S, Asmelash T, Abebe M, Dinant GJ. Half of Pulmonary Tuberculosis Cases Were Left Undiagnosed in Prisons of the Tigray Region of Ethiopia: Implications for Tuberculosis Control. *PLoS One* 2016.

199. Addis Z, Adem E, Alemu A, et al. Prevalence of smear positive pulmonary tuberculosis in Gondar prisoners, North West Ethiopia. *Asian Pacific Journal of Tropical Medicine* 2015.

200. Agajie M, Disassa H, Birhanu M, Amentie M. Prevalence of pulmonary tuberculosis and associated factors in prisons of BenishangulGumuz region, Western Ethiopia. *Prevalence* 2018; **6**(9).

201. Gizachew Beza M, Hunegnaw E, Tiruneh M. Prevalence and associated factors of tuberculosis in prisons settings of East Gojjam Zone, Northwest Ethiopia. *International Journal of Bacteriology* 2017; **2017**.

202. Dememew ZG, Jerene D, Datiko DG, et al. The yield of community-based tuberculosis and HIV among key populations in hotspot settings of Ethiopia: A cross-sectional implementation study. *PLoS One* 2020.

203. Dibissa KE, Waktole ZD, Tolessa BE. Prevalence of pulmonary tuberculosis and associated factors among prisoners in Western Oromia, Ethiopia: A cross-sectional study. *bioRxiv* 2019: 869727.

204. Fuge TG, Ayanto SY. Prevalence of smear positive pulmonary tuberculosis and associated risk factors among prisoners in Hadiya Zone prison, Southern Ethiopia. *BMC Research Notes* 2016.

205. Gebrecherkos T, Gelaw B, Tessema B. PREVALENCE, HIV CO-INFECTION AND MULTI-DRUG RESISTANCE OF SMEAR POSITIVE PULMONARY TUBERCULOSIS IN PRISON SETTINGS OF NORTHWEST ETHIOPIA. *Clinical Chemistry and Laboratory Medicine* 2017; **55**(Supplement 2): S1245.

206. Gebrecherkos T, Gelaw B, Tessema B. Smear positive pulmonary tuberculosis and HIV co-infection in prison settings of North Gondar Zone, Northwest Ethiopia. *BMC Public Health* 2016.

207. Hordofa G, Mulatu G, Daka D. Prevalence, drug-susceptibility pattern and associated factors of Mycobacterium tuberculosis infection among prisoners in western Arsi zonal prisons, Oromia, South West Ethiopia. *IJID Regions* 2023; **9**: 1-6.

208. Merid Y, Woldeamanuel Y, Abebe M, et al. High utility of active tuberculosis case finding in an Ethiopian prison. *International Journal of Tuberculosis and Lung Disease* 2018.

209. Moges B, Amare B, Asfaw F, et al. Prevalence of smear positive pulmonary tuberculosis among prisoners in North Gondar Zone Prison, northwest Ethiopia. *BMC Infectious Diseases* 2012.

210. Sahle ET, Blumenthal J, Jain S, et al. Bacteriologically-confirmed pulmonary tuberculosis in an Ethiopian prison: Prevalence from screening of entrant and resident prisoners. *PLoS One* 2019.

211. Tadesse M, Diriba G, Getahun M, et al. The Burdon and determinant of pulmonary tuberculosis in Ethiopian federal prison facilities. *International Journal of Infectious Diseases* 2020.

212. Winsa BB, Mohammed AE. Investigation on pulmonary tuberculosis among Bedele Woreda prisoners, Southwest Ethiopia. *International Journal of Biomedical Science and Engineering* 2015; **3**(6): 69-73.

213. Zerdo Z, Medhin G, Worku A, Ameni G. Prevalence of pulmonary tuberculosis and associated risk factors in prisons of Gamo Goffa Zone, south Ethiopia: A cross-sectional study. *American Journal of Health Research* 2014; **2**(5): 291-7.

214. Adjei AA, Armah HB, Gbagbo F, et al. Correlates of HIV, HBV, HCV and syphilis infections among prison inmates and officers in Ghana: A national multicenter study. *BMC Infectious Diseases* 2008.

215. Adjei AA, Armah HB, Gbagbo F, et al. Prevalence of human immunodeficiency virus, hepatitis B virus, hepatitis C virus and syphilis among prison inmates and officers at Nsawam and Accra, Ghana. 2006; **55**(5): 593-7.

216. Commission GA. National Health and HIV Survey of Prison Inmates in Ghana. 2013.

217. Sagoe KWC, Atuahene K, Ayiku ANA, et al. Hepatitis B and human immunodeficiency virus infections within correctional facilities in Ghana. *PLOS One* 2023; **18**(11): e0293009.

218. Kwabla M, Ameme D, Nortey P. Pulmonary tuberculosis and its risk factors among inmates of a Ghanaian prison. *International Journal of Tropical Disease & Health* 2015; **9**(3): 1-10.

219. Bah H, Cisse FA, Camara LM, Diallo OH, Diallo M, Sow OY. Prevalence of tuberculosis in the prison population of Conakry, Guinea Republic. *Revue de Medecine Legale* 2012.

220. Mwatenga SA, Musa AA, Muturi MW, Musyoki AM. Prevalence and associated factors of TB and HIV coinfections among adult inmates with presumptive pulmonary TB in a Kenyan prison. *Tropical Medicine and Health* 2024; **52**(1): 54.

221. Vessellee DB, Yalley AK, Adjei DN, et al. Prevalence of Hepatitis B Virus Infection among Inmates at the Monrovia Central Prison, Liberia. *Tropical Medicine and Infectious Disease* 2023; **8(3) (no pagination)**.

222. Rakotomanana F, Dreyfus A, Randrianarisoa MM, et al. Prevalence of pulmonary tuberculosis and HIV infections and risk factors associated to tuberculosis in detained persons in Antananarivo, Madagascar. *Sci Rep* 2024; **14**(1): 8640.

223. Chimphambano C, Komolafe I, Muula A. Prevalence of HIV, HepBsAg and Hep C antibodies among inmates in Chichiri prison, Blantyre, Malawi. *Malawi Medical Journal* 2007; **19**(3): 107-10.

224. Banda HT, Gausi F, Harries AD, Salaniponi FM. Prevalence of smear-positive pulmonary tuberculosis among prisoners in Malawi: A national survey. *International Journal of Tuberculosis and Lung Disease* 2009.

225. Banerjee A, Harries AD, Mphasa N, Yadid AE, Nyirenda T, Salaniponi FM. Prevalence of HIV, sexually transmitted disease and tuberculosis amongst new prisoners in a district prison, Malawi. *Tropical Doctor* 2000.

226. Kanyerere HS, Banda RP, Gausi F, et al. Surveillance of tuberculosis in Malawian prisons. *Public Health Action* 2012.

227. Mangochi P, Bossard C, Catacutan C, et al. TB screening, prevention and treatment cascade in a Malawi prison. *The International Journal of Tuberculosis and Lung Disease* 2022; **26**(10): 956-62.

228. Olotu AA, Chiramal JA, Boehm RA, et al. Accelerating Tuberculosis Diagnosis in Mozambican Prisons Using Digital Chest X-rays with Computer Aided Detection: Preliminary Results from a Longitudinal, Comprehensive Health Intervention. *medRxiv* 2024; **02**.

229. Adoga MP, Banwat EB, Forbi JC, et al. Human immunonodeficiency virus, hepatitis B virus and hepatitis C virus: sero-prevalence, co-infection and risk factors among prison inmates in Nasarawa State, Nigeria. *Journal of Infection in Developing Countries* 2009.

230. Dan-Nwafor CC, Adeoye I, Aderemi K, et al. Serological markers and risk factors associated with Hepatitis B virus infection among Federal Capital Territory prison inmates, Nigeria: Should we be concerned? *PLoS One* 2021.

231. Adesokan H, Cadmus E, Adeyemi W, et al. Prevalence of previously undetected tuberculosis and underlying risk factors for transmission in a prison setting in Ibadan, south-western Nigeria. *African Journal of Medicine and Medical Sciences* 2014; **43**(Suppl 1): 45.

232. Chigbu LN, Iroegbu CU. Incidence and spread of Mycobacterium tuberculosis-associated infection among Aba Federal prison inmates in Nigeria. *Journal of Health, Population and Nutrition* 2010.

233. Ekundayo EO, Onuka O, Mustapha G, Geoffrey M. Active case finding of pulmonary tuberculosis among prison inmates in aba Federal prison, Abia state, Nigeria. *Advances in Infectious Diseases* 2015; **5**(01): 57.

234. Onu E, Enejoh VA, Olarewaju J, et al. What is the tb burden in nigerian prisons? e an enhanced tb case finding program experience from 13 nigerian prisons. *Annals of Global Health* 2017.

235. Umutesi J, Klett-Tammen C, Nsanzimana S, Krause G, Ott JJ. Cross-sectional study of chronic hepatitis B virus infection in Rwandan high-risk groups: Unexpected findings on prevalence and its determinants. *BMJ Open* 2021.

236. Jaquet A, Wandeler G, Tine J, et al. HIV infection, viral hepatitis and liver fibrosis among prison inmates in West Africa. *BMC Infectious Diseases* 2016.

237. The Aurum Institute NICD. Socio-behavioural and structural factors driving HIV/AIDS, STIs and Hepatitis B & C infections among inmates in Correctional Facilities, Johannesburg, 2020.

238. Baird K, Said H, Koornhof HJ, Duse AG. Tuberculosis control at a South African correctional centre: Diagnosis, treatment and strain characterisation. *PLoS One* 2022; **17**(11-Nov): e0277459.

239. Hanifa Y, Telisinghe L, Fielding KL, et al. The diagnostic accuracy of urine lipoarabinomannan test for tuberculosis screening in a South African correctional facility. *PLoS One* 2015.

240. Jordan AM, Podewils LJ, Castro KG, Zishiri V, Charalambous S. Prevalence and risk factors of tuberculosis disease in South African correctional facilities in 2015. *International Journal of Tuberculosis and Lung Disease* 2019.

241. Kim HY, Zishiri V, Page-Shipp L, et al. Symptom and digital chest X-ray TB screening in South African prisons: Yield and cost-effectiveness. *International Journal of Tuberculosis and Lung Disease* 2020.

242. Kim HY, Zishiri V, Page-Shipp L, et al. Symptom and digital chest X-ray TB screening in South African and cost-effectiveness. *International Journal of Tuberculosis and Lung Disease* 2020; **24**(3): 295-+.

243. V Skiti EG, P Gribble, H Hausler. Screening and testing for tuberculosis and HIV in correctional facilities in the Western Cape, South Africa. 44th World Conference on Lung Health of the International Union Against Tuberculosis; 2013.

244. Stevenson KA, Podewils LJ, Zishiri VK, Castro KG, Charalambous S. HIV prevalence and the cascade of care in five South African correctional facilities. *PLoS One* 2020.

245. Telisinghe L, Fielding KL, Malden JL, et al. High tuberculosis prevalence in a South African prison: the need for routine tuberculosis screening. *PLoS One* 2014.

246. Velen K, Sathar F, Hoffmann CJ, et al. Digital Chest X-Ray with Computer-aided Detection for Tuberculosis Screening within Correctional Facilities. *Annals of the American Thoracic Society* 2022.

247. Zishiri V, Charalambous S, Shah MR, et al. Implementing a large-scale systematic tuberculosis screening program in correctional facilities in South Africa. Open forum infectious diseases; 2015: Oxford University Press; 2015. p. ofu121.

248. M. Dahoma EM, A. Othman, A. Seha, A. Abdullah. Predisposing sexual and drug related risk factors among prisoners in Zanzibar. International Aids Society (IAS) 2009. Cape Town; 2009.

249. J Angolwisye FK, F Nichombe, M Minja, A Rachow, H Machibia, M Pletschette, P Clowes. First survey on TB and HIV prevalence in the prisons of the Mbeya region in Tanzania. *42nd World Conference on Lung Health of the International Union Against Tuberculosis and Lung Disease* 2011; (157).

250. Mangu CD, Clowes P, van den Hombergh J, et al. New admissions and asymptomatic TB cases seem to fuel TB epidemic in prisons, a cross sectional survey in Tanzania. *PLOS Global Public Health* 2024; **4**(10).

251. Mmbaga VM. Prevalence and factors associated with pulmonary tuberculosis among prisoners in Dar es salaam, Tanzania, 2012: Muhimbili University of Health and Allied Sciences; 2013.

252. Steiner A, Mangu C, van den Hombergh J, et al. Screening for pulmonary tuberculosis in a tanzanian prison and computer-aided interpretation of chest X-rays. *Public Health Action* 2015.

253. Mpeirwe M, Rugera S, Boum Ii Y. Diagnosis of tuberculosis in a high TB-HIV environment using microscopy and culture: The example of Kakiika Prison-Kyamugorani, Mbarara, Uganda. *Journal of Science and Technology (Ghana)* 2016; **36**: 29.

254. Owokuhaisa J, Thokerunga E, Bazira J. Prevalence of pulmonary tuberculosis among prison inmates at Mbarara central prison, South Western Uganda. *Advances in Research* 2014; **2**(11): 618.

255. United Nations Office on Drugs and Crime. A Rapid Situation Assessment of HIV/STI/TB and Drug Abuse among Prisoners in Uganda Prisons Service, 2009.

256. Habeenzu C, Mitarai S, Lubasi D, et al. Tuberculosis and multidrug resistance in Zambian prisons, 2000-2001. *International Journal of Tuberculosis and Lung Disease* 2007.

257. Harris JB, Siyambango M, Levitan EB, et al. Derivation of a tuberculosis screening rule for sub-Saharan African prisons. *International Journal of Tuberculosis and Lung Disease* 2014.

258. Kagujje M, Somwe P, Hatwiinda S, et al. Cross-sectional assessment of tuberculosis and HIV prevalence in 13 correctional facilities in Zambia. *BMJ Open* 2021.

259. Maggard KR, Hatwiinda S, Harris JB, et al. Screening for tuberculosis and testing for human immunodeficiency virus in Zambian prisons. *Bulletin of the World Health Organization* 2015.

260. Mahfoud Z, Kassak K, Kreidieh K, Shamra S, Ramia S. Prevalence of antibodies to human immunodeficiency virus (HIV), hepatitis B and hepatitis C and risk factors in prisoners in Lebanon. *Journal of Infection in Developing Countries* 2010.

261. Ziglam H, Zorgani AA, Balouz A, Abudhe AH, Elahmer O. Prevalence of antibodies to human immunodeficiency virus, hepatitis B, and hepatitis C in prisoners in Libya. *Libyan Journal of Medicine* 2012.

262. Kobeissi L. The Integrated Bio-Behavioral Survey (IBBS) in Syria: 2013-2014. In: Program UND, editor.; 2014.

263. Balci E, Turker K, Senol V, Gunay O. Screening Indicators of Hepatitis A, Hepatitis B, Hepatitis C and HIV infections in Prisoners. *Viral Hepatit Dergisi-Viral Hepatitis Journal* 2012.

264. Keten D, Ova ME, Keten HS, et al. The prevalence of hepatitis B and C among prisoners in Kahramanmaras, Turkey. *Jundishapur Journal of Microbiology* 2016.

265. Kose S, Adar P, Gozaydin A, Kuzucu L, Akkoclu G. Hepatitis B and Hepatitis C in prisons: a prevalence study. *International Journal of Prison Health* 2019.

266. Sahin AM, Sahin AR, Gunduz A, Aktemur A, Uzun N. Prevalence of Hepatitis B virus and Hepatitis C virus among prison inmates in Istanbul, Turkey. *Annals of Clinical and Analytical Medicine* 2022.

267. Kiter G, Arpaz S, Keskin S, Sezgin N, Budin D, Seref O. Tuberculosis in Nazilli District Prison, Turkey, 1997-2001. *International Journal of Tuberculosis and Lung Disease* 2003.

268. Borekci S, Ongen G, Icmeli OS, et al. Pulmonary tuberculosis incidence in Turkish prisons: Importance of screening and case finding strategies. *Tuberkuloz ve Toraks* 2013.

269. Butler TS, M. National Prison Entrants’ Bloodborne Virus and Risk Behaviour Survey Report: Kirby Institute 2017.

270. Gilles M, Swingler E, Craven C, Larson A. Prison health and public health responses at a regional prison in Western Australia. *Australian and New Zealand Journal of Public Health* 2008.

271. Indig D, Topp L, Ross B, et al. 2009 NSW Inmate Health Survey: Key Findings Report. Sydney: Justice Health, 2010.

272. Stoové M, Kirwan A. External component of the evaluation of drug policies and services and their subsequent effects on prisoners and staff within the Alexander Maconochie Centre. 2011.

273. Young JT, Van Dooren K, Borschmann R, Kinner S. ACT detainee health and wellbeing survey 2016: Summary results: ACT Government; 2017.

## *Table 9.3*: Country-level estimates of the number and rate of incarceration, prevalence of lifetime injecting drug use, HIV and HCV among females who are incarcerated

|  | **Females who are incarcerated** | | | **Females with lifetime injecting drug use** | | | | **Females living with HIV** | | | | **Females with current HCV** | | | |
| --- | --- | --- | --- | --- | --- | --- | --- | --- | --- | --- | --- | --- | --- | --- | --- |
| **Country** | **Estimated number^1^** | **Rate per 100,000** | **Year of estimate^1^** | **% (CI)** | **Estimated no. (CI)** | **Year of estimate** | **Sources** | **% (CI)** | **Estimated no. (CI)** | **Year of estimate** | **Sources** | **% (CI)** | **Estimated no. (CI)** | **Year of estimate** | **Sources** |
| **Eastern Europe** |  |  |  |  |  |  |  |  |  |  |  |  |  |  |  |
| Armenia | 64 | 7 | 2024 | ·· | ·· | ·· |  | ·· | ·· | ·· |  | ·· | ·· | ·· |  |
| Azerbaijan | 716 | 19 | 2023 | ·· | ·· | ·· |  | 1·7 (0·0, 7·3) | < 500 (<500,<500) | 2014 | 1 | 8·6 (2·5, 17·5) | < 500 (<500,<500) | 2014 | 1 |
| Belarus | 3516 | 106 | 2018 | ·· | ·· | ·· |  | ·· | ·· | ·· |  | ·· | ·· | ·· |  |
| Bosnia & Herzegovina | 64 | 5 | 2024/2023 | ·· | ·· | ·· |  | ·· | ·· | ·· |  | ·· | ·· | ·· |  |
| Bulgaria | 230 | 11 | 2024 | ·· | ·· | ·· |  | ·· | ·· | ·· |  | 13·5 (11·6, 15·6) | < 500 (<500,<500) | 2009 | 2 |
| Czechia | 1709 | 52 | 2024 | ·· | ·· | ·· |  | ·· | ·· | ·· |  | ·· | ·· | ·· |  |
| Estonia | 76 | 19 | 2024 | ·· | ·· | ·· |  | 38·2 (31·1, 45·7) | < 500 (<500,<500) | 2012 | 3 | ·· | ·· | ·· |  |
| Georgia | 429 | 29 | 2024 | ·· | ·· | ·· |  | ·· | ·· | ·· |  | ·· | ·· | ·· |  |
| Hungary | 1535 | 49 | 2023 | 4·6 (4·0, 5·2) | < 500 (<500,<500) | 1998 | 4 | ·· | ·· | ·· |  | ·· | ·· | ·· |  |
| Latvia | 252 | 46 | 2024 | 60·0 (51·4, 68·3) | < 500 (<500,<500) | 2022 | 5 | ·· | ·· | ·· |  | ·· | ·· | ·· |  |
| Lithuania | 205 | 23 | 2024 | 7·0 (2·0, 14·4) | < 500 (<500,<500) | 2009 | 6 | ·· | ·· | ·· |  | ·· | ·· | ·· |  |
| Republic of Moldova | 302 | 29 | 2024 | ·· | ·· | ·· |  | ·· | ·· | ·· |  | ·· | ·· | ·· |  |
| Poland | 3727 | 28 | 2024 | ·· | ·· | ·· |  | ·· | ·· | ·· |  | ·· | ·· | ·· |  |
| Romania | 1104 | 18 | 2024 | ·· | ·· | ·· |  | ·· | ·· | ·· |  | ·· | ·· | ·· |  |
| Russian Federation | 38538 | 77 | 2023 | ·· | ·· | ·· |  | ·· | ·· | ·· |  | ·· | ·· | ·· |  |
| Slovakia | 627 | 37 | 2024 | ·· | ·· | ·· |  | ·· | ·· | ·· |  | ·· | ·· | ·· |  |
| Ukraine | 2333 | 11 | 2024 | ·· | ·· | ·· |  | 31·8 (26·4, 37·3) | 500 (500,1000) | 2011 | 7,8 | 25·2 (17·3, 34·1) | 500 (500,1000) | 2021 | 9 |
| **Western Europe** |  |  |  |  |  |  |  |  |  |  |  |  |  |  |  |
| Albania | 60 | 6 | 2024 | ·· | ·· | ·· |  | ·· | ·· | ·· |  | ·· | ·· | ·· |  |
| Andorra | 6 | 22 | 2024 | ·· | ·· | ·· |  | ·· | ·· | ·· |  | ·· | ·· | ·· |  |
| Austria | 632 | 21 | 2024 | ·· | ·· | ·· |  | ·· | ·· | ·· |  | ·· | ·· | ·· |  |
| Belgium | 566 | 15 | 2024 | ·· | ·· | ·· |  | ·· | ·· | ·· |  | 0·3 (0·2, 0·6) | < 500 (<500,<500) | 2020 | 10 |
| Croatia | 227 | 20 | 2023 | 19·1 (13·8, 25·1) | < 500 (<500,<500) | 2007 | 11 | 0·0 (0·0, 0·9) | < 500 (<500,<500) | 2006 | 12 | 8·0 (4·5, 12·3) | < 500 (<500,<500) | 2007 | 11 |
| Denmark | 216 | 11 | 2024 | ·· | ·· | ·· |  | ·· | ·· | ·· |  | ·· | ·· | ·· |  |
| England and Wales | 3521 | 18 | 2024 | 33·3 (30·2, 36·6) | 1000 (1000,1500) | 1998-2007 | 13-15 | 0·4 (0·2, 0·9) | < 500 (<500,<500) | 1998-2020 | 13,15,16 | 5·5 (3·0, 8·7) | < 500 (<500,500) | 1998-2024 | 13,15-19 |
| Finland | 224 | 12 | 2023 | 64·3 (59·5, 69·1) | < 500 (<500,<500) | 2007-23 | 20,21 | 1·1 (0·0, 3·3) | < 500 (<500,<500) | 2007-23 | 21,22 | 39·5 (32·5, 46·6) | < 500 (<500,<500) | 2007-23 | 21,22 |
| France | 2707 | 18 | 2024 | 17·3 (5·1, 33·9) | 500 (<500,1000) | 1997 | 23 | ·· | ·· | ·· |  | ·· | ·· | ·· |  |
| Germany | 3419 | 12 | 2023 | ·· | ·· | ·· |  | ·· | ·· | ·· |  | ·· | ·· | ·· |  |
| Greece | 512 | 15 | 2024 | ·· | ·· | ·· |  | ·· | ·· | ·· |  | ·· | ·· | ·· |  |
| Greenland | 21 | 113 | 2023 | NK | NK | ·· |  | ·· | ·· | ·· |  | ·· | ·· | ·· |  |
| Iceland | 14 | 10 | 2024 | ·· | ·· | ·· |  | ·· | ·· | ·· |  | ·· | ·· | ·· |  |
| Ireland | 269 | 14 | 2024 | 47·3 (38·8, 56·3) | < 500 (<500,<500) | 1998-2012 | 24,25 | 4·3 (1·9, 7·6) | < 500 (<500,<500) | 1998-2012 | 24-26 | 18·8 (12·1, 26·7) | < 500 (<500,<500) | 1998-2012 | 24-27 |
| Italy | 2733 | 14 | 2024 | 24·1 (16·7, 33·0) | 500 (500,1000) | 2019-21 | 28-30 | 3·7 (2·1, 5·9) | < 500 (<500,<500) | 2002-23 | 28,31,32 | 12·5 (7·8, 18·2) | 500 (<500,500) | 2002-23 | 28-35 |
| Liechtenstein | 0 | 0 | 2024 | ·· | ·· | ·· |  | ·· | ·· | ·· |  | ·· | ·· | ·· |  |
| Luxembourg | 33 | 15 | 2024 | ·· | ·· | ·· |  | ·· | ·· | ·· |  | ·· | ·· | ·· |  |
| Malta | 61 | 35 | 2024 | ·· | ·· | ·· |  | ·· | ·· | ·· |  | ·· | ·· | ·· |  |
| Monaco | 0 | 42 | 2024 | ·· | ·· | ·· |  | ·· | ·· | ·· |  | ·· | ·· | ·· |  |
| Montenegro | 37 | 16 | 2024 | ·· | ·· | ·· |  | 0·0 (0·0, 7·6) | < 500 (<500,<500) | 2012-21 | 36 | ·· | ·· | ·· |  |
| Netherlands | 542 | 9 | 2023 | ·· | ·· | ·· |  | ·· | ·· | ·· |  | ·· | ·· | ·· |  |
| North Macedonia | 95 | 11 | 2024 | ·· | ·· | ·· |  | ·· | ·· | ·· |  | ·· | ·· | ·· |  |
| Northern Ireland | 92 | 14 | 2024 | 27·3 (4·4, 57·9) | < 500 (<500,<500) | 2005 | 37 | ·· | ·· | ·· |  | ·· | ·· | ·· |  |
| Norway | 140 | 9 | 2024 | 54·2 (41·0, 67·2) | < 500 (<500,<500) | 2014 | 38 | ·· | ·· | ·· |  | ·· | ·· | ·· |  |
| Portugal | 891 | 26 | 2024 | 16·0 (13·2, 19·0) | < 500 (<500,<500) | 2002-05 | 39,40 | 5·8 (2·9, 9·5) | < 500 (<500,<500) | 2002-23 | 39-42 | 9·0 (7·2, 11·2) | < 500 (<500,<500) | 2002-23 | 39-41 |
| San Marino | 0 | 0 | 2024 | ·· | ·· | ·· |  | ·· | ·· | ·· |  | ·· | ·· | ·· |  |
| Scotland | 338 | 17 | 2024 | 15·4 (10·1, 21·3) | < 500 (<500,<500) | 2011 | 43 | ·· | ·· | ·· |  | ·· | ·· | ·· |  |
| Serbia | 453 | 15 | 2023 | ·· | ·· | ·· |  | ·· | ·· | ·· |  | ·· | ·· | ·· |  |
| Slovenia | 99 | 15 | 2024 | ·· | ·· | ·· |  | ·· | ·· | ·· |  | ·· | ·· | ·· |  |
| Spain | 4026 | 26 | 2023 | ·· | ·· | ·· |  | 16·3 (7·4, 28·7) | 500 (500,1000) | 2005-20 | 44,45 | 12·5 (8·0, 19·1) | 500 (500,1000) | 2005-20 | 44-46 |
| Sweden | 641 | 19 | 2024 | ·· | ·· | ·· |  | 0·0 (0·0, 6·3) | < 500 (<500,<500) | 2017 | 47 | 11·1 (1·5, 26·3) | < 500 (<500,<500) | 2017 | 47 |
| Switzerland | 392 | 14 | 2024 | ·· | ·· | ·· |  | 9·3 (5·0, 14·7) | < 500 (<500,<500) | 2011 | 48 | 7·9 (3·9, 13·0) | < 500 (<500,<500) | 2011 | 48 |
| **East and South East Asia** |  |  |  |  |  |  |  |  |  |  |  |  |  |  |  |
| Brunei Darussalam | 76 | 50 | 2022 | ·· | ·· | ·· |  | ·· | ·· | ·· |  | ·· | ·· | ·· |  |
| Cambodia | 2978 | 55 | 2024 | ·· | ·· | ·· |  | ·· | ·· | ·· |  | ·· | ·· | ·· |  |
| China | 145340 | 30 | 2018 | ·· | ·· | ·· |  | ·· | ·· | ·· |  | ·· | ·· | ·· |  |
| Hong Kong† | 1907 | 68 | 2023 | ·· | ·· | ·· |  | ·· | ·· | ·· |  | ·· | ·· | ·· |  |
| Indonesia | 13155 | 14 | 2024 | 4·2 (3·4, 5·1) | 500 (500,500) | 2010 | 49 | 4·8 (1·4, 10·1) | 500 (<500,1500) | 2010-17 | 49,50 | 2·3 (0·7, 4·9) | 500 (<500,500) | 2017 | 50 |
| Japan | 3598 | 10 | 2023 | ·· | ·· | ·· |  | ·· | ·· | ·· |  | ·· | ·· | ·· |  |
| Lao People's Democratic Republic | 1628 | 68 | 2018 | ·· | ·· | ·· |  | ·· | ·· | ·· |  | ·· | ·· | ·· |  |
| Malaysia | 5158 | 45 | 2024 | ·· | ·· | ·· |  | ·· | ·· | ·· |  | ·· | ·· | ·· |  |
| Mongolia | 257 | 24 | 2023 | ·· | ·· | ·· |  | ·· | ·· | ·· |  | ·· | ·· | ·· |  |
| Myanmar | 12340 | 67 | 2020 | ·· | ·· | ·· |  | 7·7 (5·8, 9·8) | 1000 (500,1000) | 2018 | 51 | ·· | ·· | ·· |  |
| Democratic People's Republic of Korea* | 8889 | 66 | 2016 | NK | NK | ·· |  | ·· | ·· | ·· |  | ·· | ·· | ·· |  |
| Philippines | 16782 | 47 | 2024 | ·· | ·· | ·· |  | 0·0 (0·0, 1·7) | < 500 (<500,500) | 1997 | 52 | ·· | ·· | ·· |  |
| Singapore | 973 | 47 | 2022 | ·· | ·· | ·· |  | ·· | ·· | ·· |  | ·· | ·· | ·· |  |
| Republic of Korea | 4447 | 24 | 2022 | ·· | ·· | ·· |  | ·· | ·· | ·· |  | ·· | ·· | ·· |  |
| Taiwan† | 5653 | 62 | 2024 | 46·8 (45·6, 47·9) | 2500 (2500,2500) | 1994 | 53 | 0·0 (0·0, 23·2) | < 500 (<500,1500) | 2019 | 54 | ·· | ·· | ·· |  |
| Thailand | 33188 | 130 | 2023 | ·· | ·· | ·· |  | ·· | ·· | ·· |  | ·· | ·· | ·· |  |
| Timor-Leste | 31 | 8 | 2021 | ·· | ·· | ·· |  | ·· | ·· | ·· |  | ·· | ·· | ·· |  |
| Viet Nam | 16212 | 48 | 2022 | ·· | ·· | ·· |  | ·· | ·· | ·· |  | ·· | ·· | ·· |  |
| **South Asia** |  |  |  |  |  |  |  |  |  |  |  |  |  |  |  |
| Afghanistan | 798 | 7 | 2024 | ·· | ·· | ·· |  | ·· | ·· | ·· |  | ·· | ·· | ·· |  |
| Bangladesh | 2099 | 4 | 2024 | ·· | ·· | ·· |  | ·· | ·· | ·· |  | ·· | ·· | ·· |  |
| Bhutan | 48 | 19 | 2014 | ·· | ·· | ·· |  | ·· | ·· | ·· |  | ·· | ·· | ·· |  |
| India | 23502 | 5 | 2022 | ·· | ·· | ·· |  | 0·1 (0·0, 0·1) | < 500 (<500,<500) | 2016 | 55 | 2·2 (2·1, 2·4) | 500 (500,500) | 2015-16 | 56-58 |
| Iran (Islamic Republic of) | 5859 | 20 | 2020 | 3·5 (3·0, 4·1) | < 500 (<500,<500) | 2009-13 | 59-62 | 0·7 (0·4, 1·0) | < 500 (<500,<500) | 2009-17 | 62-64 | 4·5 (2·3, 7·5) | 500 (<500,500) | 2008-19 | 62,65-69 |
| Maldives | 73 | 47 | 2020 | ·· | ·· | ·· |  | ·· | ·· | ·· |  | ·· | ·· | ·· |  |
| Nepal | 1488 | 14 | 2022 | ·· | ·· | ·· |  | ·· | ·· | ·· |  | ·· | ·· | ·· |  |
| Pakistan | 1738 | 3 | 2024 | ·· | ·· | ·· |  | 1·2 (0·5, 2·2) | < 500 (<500,<500) | 2009-10 | 70,71 | 10·9 (6·8, 16·0) | < 500 (<500,500) | 2010 | 71 |
| Sri Lanka | 1247 | 17 | 2024 | 2·9 (0·0, 8·5) | < 500 (<500,<500) | 2013 | 72 | ·· | ·· | ·· |  | 0·0 (0·0, 2·5) | < 500 (<500,<500) | 2013 | 72 |
| **Central Asia** |  |  |  |  |  |  |  |  |  |  |  |  |  |  |  |
| Kazakhstan | 2466 | 40 | 2022 | ·· | ·· | ·· |  | ·· | ·· | ·· |  | ·· | ·· | ·· |  |
| Kyrgyzstan | 224 | 11 | 2023 | ·· | ·· | ·· |  | ·· | ·· | ·· |  | ·· | ·· | ·· |  |
| Tajikistan | 336 | 11 | 2022 | ·· | ·· | ·· |  | ·· | ·· | ·· |  | ·· | ·· | ·· |  |
| Turkmenistan | 2275 | 111 | 2021 | ·· | ·· | ·· |  | ·· | ·· | ·· |  | ·· | ·· | ·· |  |
| Uzbekistan | 1672 | 9 | 2022 | ·· | ·· | ·· |  | ·· | ·· | ·· |  | ·· | ·· | ·· |  |
| **Caribbean** |  |  |  |  |  |  |  |  |  |  |  |  |  |  |  |
| Antigua & Barbuda | 22 | 64 | 2023 | NK | NK | ·· |  | ·· | ·· | ·· |  | ·· | ·· | ·· |  |
| Bahamas | 141 | 39 | 2021 | ·· | ·· | ·· |  | ·· | ·· | ·· |  | ·· | ·· | ·· |  |
| Barbados | 21 | 25 | 2023 | ·· | ·· | ·· |  | ·· | ·· | ·· |  | ·· | ·· | ·· |  |
| Bermuda | 4 | 19 | 2021 | ·· | ·· | ·· |  | ·· | ·· | ·· |  | ·· | ·· | ·· |  |
| Cuba | 2848 | 50 | 2020 | ·· | ·· | ·· |  | ·· | ·· | ·· |  | ·· | ·· | ·· |  |
| Dominica | 3 | 12 | 2024 | NK | NK | ·· |  | ·· | ·· | ·· |  | ·· | ·· | ·· |  |
| Dominican Republic | 676 | 18 | 2024 | ·· | ·· | ·· |  | ·· | ·· | ·· |  | ·· | ·· | ·· |  |
| Grenada | 6 | 12 | 2023 | NK | NK | ·· |  | ·· | ·· | ·· |  | ·· | ·· | ·· |  |
| Haiti | 263 | 7 | 2024 | ·· | ·· | ·· |  | ·· | ·· | ·· |  | ·· | ·· | ·· |  |
| Jamaica | 142 | 14 | 2022 | ·· | ·· | ·· |  | ·· | ·· | ·· |  | ·· | ·· | ·· |  |
| Commonwealth of Puerto Rico | 215 | 20 | 2022 | ·· | ·· | ·· |  | ·· | ·· | ·· |  | ·· | ·· | ·· |  |
| Saint Kitts & Nevis | 6 | 35 | 2022 | NK | NK | ·· |  | ·· | ·· | ·· |  | ·· | ·· | ·· |  |
| Saint Lucia | 15 | 23 | 2023 | NK | NK | ·· |  | ·· | ·· | ·· |  | ·· | ·· | ·· |  |
| Saint Vincent & the Grenadines | 8 | 32 | 2024 | NK | NK | ·· |  | ·· | ·· | ·· |  | ·· | ·· | ·· |  |
| Trinidad & Tobago | 110 | 21 | 2021 | NK | NK | ·· |  | ·· | ·· | ·· |  | ·· | ·· | ·· |  |
| **Latin America** |  |  |  |  |  |  |  |  |  |  |  |  |  |  |  |
| Argentina | 5127 | 35 | 2023 | ·· | ·· | ·· |  | 3·4 (2·1, 5·4) | < 500 (<500,500) | 2016 | 73 | 1·8 (1·1, 2·7) | < 500 (<500,<500) | 2016 | 73 |
| Belize | 48 | 30 | 2024 | NK | NK | ·· |  | ·· | ·· | ·· |  | ·· | ·· | ·· |  |
| Bolivia (Plurinational State of) | 2053 | 49 | 2024 | ·· | ·· | ·· |  | 0·6 (0·0, 1·7) | < 500 (<500,<500) | 2002-13 | 74,75 | ·· | ·· | ·· |  |
| Brazil | 50661 | 63 | 2024 | 10·9 (4·5, 19·7) | 5500 (2500,10000) | 1997-2013 | 76-80 | 5·1 (2·8, 8·1) | 2500 (1500,4000) | 1997-2020 | 76,78,81-88 | 4·2 (1·9, 7·4) | 2000 (1000,3500) | 1997-2015 | 77,79,83,89-94 |
| Chile | 4900 | 70 | 2024 | ·· | ·· | ·· |  | ·· | ·· | ·· |  | ·· | ·· | ·· |  |
| Colombia | 6365 | 37 | 2024 | ·· | ·· | ·· |  | ·· | ·· | ·· |  | ·· | ·· | ·· |  |
| Costa Rica | 784 | 48 | 2022 | ·· | ·· | ·· |  | ·· | ·· | ·· |  | ·· | ·· | ·· |  |
| Ecuador | 2087 | 31 | 2024 | ·· | ·· | ·· |  | ·· | ·· | ·· |  | ·· | ·· | ·· |  |
| El Salvador | 8104 | 367 | 2024 | ·· | ·· | ·· |  | ·· | ·· | ·· |  | ·· | ·· | ·· |  |
| Guatemala | 2827 | 51 | 2023 | ·· | ·· | ·· |  | ·· | ·· | ·· |  | ·· | ·· | ·· |  |
| Guyana | 60 | 24 | 2024 | ·· | ·· | ·· |  | ·· | ·· | ·· |  | ·· | ·· | ·· |  |
| Honduras | 1188 | 32 | 2023 | ·· | ·· | ·· |  | ·· | ·· | ·· |  | ·· | ·· | ·· |  |
| Mexico | 13836 | 31 | 2024 | 5·0 (4·2, 5·8) | 500 (500,1000) | 2010 | 95 | 0·7 (0·6, 0·8) | < 500 (<500,<500) | 2010 | 95 | 1·6 (1·4, 1·9) | < 500 (<500,500) | 2010 | 95,96 |
| Nicaragua | 1130 | 50 | 2018 | ·· | ·· | ·· |  | ·· | ·· | ·· |  | ·· | ·· | ·· |  |
| Panama | 1166 | 86 | 2024 | ·· | ·· | ·· |  | ·· | ·· | ·· |  | ·· | ·· | ·· |  |
| Paraguay | 213 | 43 | 2023 | ·· | ·· | ·· |  | ·· | ·· | ·· |  | ·· | ·· | ·· |  |
| Peru | 5075 | 46 | 2024 | ·· | ·· | ·· |  | 1·8 (0·7, 3·4) | < 500 (<500,<500) | 2011-15 | 97,98 | ·· | ·· | ·· |  |
| Suriname | 31 | 14 | 2014 | ·· | ·· | ·· |  | ·· | ·· | ·· |  | ·· | ·· | ·· |  |
| Uruguay | 1246 | 104 | 2024 | ·· | ·· | ·· |  | ·· | ·· | ·· |  | ·· | ·· | ·· |  |
| Venezuela (Bolivarian Republic of) | 5242 | 58 | 2022 | ·· | ·· | ·· |  | 0·3 (0·2, 0·3) | < 500 (<500,<500) | 2018-22 | 99,100 | 0·0 (0·0, 4·1) | < 500 (<500,<500) | 2018 | 99 |
| **North America** |  |  |  |  |  |  |  |  |  |  |  |  |  |  |  |
| Canada | 2449 | 20 | 2023 | 44·3 (40·0, 48·7) | 1000 (1000,1000) | 2001-15 | 101-105 | 0·7 (0·5, 0·9) | < 500 (<500,<500) | 2015 | 102 | 21·2 (17·4, 25·6) | 500 (500,500) | 2003-15 | 101,102,105 |
| United States of America | 159113 | 146 | 2022 | 20·2 (15·5, 25·5) | 32000 (24500,40500) | 1993-2013 | 106-117 | 2·6 (2·1, 3·1) | 4000 (3500,5000) | 1994-2023 | 108-112,118-141 | 21·1 (18·3, 24·0) | 33500 (29000,38000) | 1994-2022 | 109,113,121,136,138,142-152 |
| **Pacific Island States & Terr·** |  |  |  |  |  |  |  |  |  |  |  |  |  |  |  |
| American Samoa | 29 | 193 | 2022 | ·· | ·· | ·· |  | ·· | ·· | ·· |  | ·· | ·· | ·· |  |
| Micronesia (Federated States of) | 0 | 0 | 2014 | ·· | ·· | ·· |  | ·· | ·· | ·· |  | ·· | ·· | ·· |  |
| Fiji | 71 | 19 | 2024 | ·· | ·· | ·· |  | ·· | ·· | ·· |  | ·· | ·· | ·· |  |
| French Polynesia | 21 | 23 | 2024 | ·· | ·· | ·· |  | ·· | ·· | ·· |  | ·· | ·· | ·· |  |
| Guam | 35 | 67 | 2024 | ·· | ·· | ·· |  | ·· | ·· | ·· |  | ·· | ·· | ·· |  |
| Kiribati | 1 | 2 | 2016 | ·· | ·· | ·· |  | ·· | ·· | ·· |  | ·· | ·· | ·· |  |
| Marshall Islands | 0 | 0 | 2014 | ·· | ·· | ·· |  | ·· | ·· | ·· |  | ·· | ·· | ·· |  |
| Nauru | 2 | 55 | 2023 | NK | NK | ·· |  | ·· | ·· | ·· |  | ·· | ·· | ·· |  |
| New Caledonia | 9 | 9 | 2024 | ·· | ·· | ·· |  | ·· | ·· | ·· |  | ·· | ·· | ·· |  |
| Northern Mariana Islands | 10 | 64 | 2022 | ·· | ·· | ·· |  | ·· | ·· | ·· |  | ·· | ·· | ·· |  |
| Palau | 3 | 51 | 2023 | ·· | ·· | ·· |  | ·· | ·· | ·· |  | ·· | ·· | ·· |  |
| Papua New Guinea | 236 | 8 | 2023 | ·· | ·· | ·· |  | ·· | ·· | ·· |  | ·· | ·· | ·· |  |
| Samoa | 19 | 31 | 2019 | ·· | ·· | ·· |  | ·· | ·· | ·· |  | ·· | ·· | ·· |  |
| Solomon Islands | 9 | 5 | 2019 | ·· | ·· | ·· |  | ·· | ·· | ·· |  | ·· | ·· | ·· |  |
| Tonga | 21 | 65 | 2022 | ·· | ·· | ·· |  | ·· | ·· | ·· |  | ·· | ·· | ·· |  |
| Tuvalu | 0 | 0 | 2014 | ·· | ·· | ·· |  | ·· | ·· | ·· |  | ·· | ·· | ·· |  |
| Vanuatu | 13 | 14 | 2021 | ·· | ·· | ·· |  | ·· | ·· | ·· |  | ·· | ·· | ·· |  |
| **Australasia** |  |  |  |  |  |  |  |  |  |  |  |  |  |  |  |
| Australia‡§ | 3480 | 40 | 2024 | 68·4 (63·1, 73·7) | 2500 (2000,2500) | 1996-2016 | 153-158 | 0·7 (0·1, 4·5) | < 500 (<500,<500) | 2023 | 159 | 2·5 (0·9, 6·6) | < 500 (<500,<500) | 2023 | 159 |
| New Zealand | 675 | 37 | 2024 | ·· | ·· | ·· |  | ·· | ·· | ·· |  | ·· | ·· | ·· |  |
| **Sub Saharan Africa** |  |  |  |  |  |  |  |  |  |  |  |  |  |  |  |
| Angola | 602 | 7 | 2024 | ·· | ·· | ·· |  | ·· | ·· | ·· |  | ·· | ·· | ·· |  |
| Benin | 606 | 18 | 2024 | ·· | ·· | ·· |  | ·· | ·· | ·· |  | ·· | ·· | ·· |  |
| Botswana | 199 | 24 | 2022 | ·· | ·· | ·· |  | ·· | ·· | ·· |  | ·· | ·· | ·· |  |
| Burkina Faso | 158 | 3 | 2022 | ·· | ·· | ·· |  | 13·6 (9·2, 18·8) | < 500 (<500,<500) | 2017 | 160 | ·· | ·· | ·· |  |
| Burundi | 1037 | 32 | 2024 | ·· | ·· | ·· |  | ·· | ·· | ·· |  | ·· | ·· | ·· |  |
| Cameroon | 895 | 12 | 2024 | ·· | ·· | ·· |  | ·· | ·· | ·· |  | ·· | ·· | ·· |  |
| Cabo Verde | 78 | 40 | 2024 | ·· | ·· | ·· |  | ·· | ·· | ·· |  | ·· | ·· | ·· |  |
| Central African Republic | 104 | 5 | 2023 | ·· | ·· | ·· |  | ·· | ·· | ·· |  | ·· | ·· | ·· |  |
| Chad | 144 | 5 | 2022 | ·· | ·· | ·· |  | ·· | ·· | ·· |  | ·· | ·· | ·· |  |
| Comoros | 7 | 3 | 2023 | ·· | ·· | ·· |  | ·· | ·· | ·· |  | ·· | ·· | ·· |  |
| Côte d'Ivoire | 679 | 9 | 2024 | ·· | ·· | ·· |  | 18·6 (16·0, 21·5) | < 500 (<500,<500) | 2015 | 161 | ·· | ·· | ·· |  |
| Democratic Republic of the Congo | 1024 | 4 | 2022 | ·· | ·· | ·· |  | ·· | ·· | ·· |  | ·· | ·· | ·· |  |
| Djibouti | 38 | 11 | 2022 | ·· | ·· | ·· |  | ·· | ·· | ·· |  | ·· | ·· | ·· |  |
| Equatorial Guinea | 25 | 6 | 2015 | NK | NK | ·· |  | ·· | ·· | ·· |  | ·· | ·· | ·· |  |
| Eritrea** | ·· | ·· | N/A | NK | NK | ·· |  | 9·1 (3·5, 16·7) | < 500 (<500,<500) | 2014 | 162 | 0·0 (0·0, 2·2) | < 500 (<500,<500) | 2014 | 162 |
| Eswatini | 99 | 0 | 2022 | ·· | ·· | ·· |  | 71·4 (55·2, 85·4) | < 500 (<500,<500) | 2010 | 163 | ·· | ·· | ·· |  |
| Ethiopia | 5390 | 14 | 2020 | ·· | ·· | ·· |  | 3·9 (3·3, 4·5) | < 500 (<500,<500) | 2016 | 164 | 1·1 (0·1, 3·2) | < 500 (<500,<500) | 2020-22 | 165-167 |
| Gabon | 193 | 18 | 2024 | ·· | ·· | ·· |  | ·· | ·· | ·· |  | ·· | ·· | ·· |  |
| Gambia | 14 | 2 | 2021 | ·· | ·· | ·· |  | ·· | ·· | ·· |  | ·· | ·· | ·· |  |
| Ghana | 214 | 2 | 2024 | 1·5 (1·5, 1·6) | < 500 (<500,<500) | 2011 | 168 | 35·5 (24·1, 49·8) | < 500 (<500,<500) | 2005-13 | 168-170 | 10·2 (8·6, 11·9) | < 500 (<500,<500) | 2005 | 171 |
| Guinea | 166 | 4 | 2024 | ·· | ·· | ·· |  | ·· | ·· | ·· |  | ·· | ·· | ·· |  |
| Guinea-Bissau | 15 | 3 | 2017 | ·· | ·· | ·· |  | ·· | ·· | ·· |  | ·· | ·· | ·· |  |
| Kenya | 2820 | 19 | 2023 | ·· | ·· | ·· |  | ·· | ·· | ·· |  | ·· | ·· | ·· |  |
| Lesotho | 64 | 9 | 2019 | ·· | ·· | ·· |  | ·· | ·· | ·· |  | ·· | ·· | ·· |  |
| Liberia | 84 | 6 | 2023 | ·· | ·· | ·· |  | ·· | ·· | ·· |  | ·· | ·· | ·· |  |
| Madagascar | 1679 | 20 | 2023 | ·· | ·· | ·· |  | ·· | ·· | ·· |  | ·· | ·· | ·· |  |
| Malawi | 182 | 3 | 2024 | ·· | ·· | ·· |  | 44·0 (29·5, 60·7) | < 500 (<500,<500) | 2005 | 172 | 0·0 (0·0, 7·7) | < 500 (<500,<500) | 2005 | 172 |
| Mali | 260 | 5 | 2022 | ·· | ·· | ·· |  | ·· | ·· | ·· |  | ·· | ·· | ·· |  |
| Mauritania | 57 | 4 | 2022 | NK | NK | ·· |  | ·· | ·· | ·· |  | ·· | ·· | ·· |  |
| Mauritius | 174 | 38 | 2024 | ·· | ·· | ·· |  | ·· | ·· | ·· |  | ·· | ·· | ·· |  |
| Mozambique | 660 | 7 | 2024 | ·· | ·· | ·· |  | ·· | ·· | ·· |  | ·· | ·· | ·· |  |
| Namibia | 258 | 33 | 2021/2022 | ·· | ·· | ·· |  | ·· | ·· | ·· |  | ·· | ·· | ·· |  |
| Niger | 403 | 7 | 2023 | ·· | ·· | ·· |  | ·· | ·· | ·· |  | ·· | ·· | ·· |  |
| Nigeria | 1848 | 3 | 2024 | ·· | ·· | ·· |  | 5·1 (2·5, 8·6) | < 500 (<500,<500) | 2010-17 | 173,174 | 15·4 (0·4, 41·0) | 500 (<500,1000) | 2018 | 175 |
| Congo | 51 | 3 | 2019 | ·· | ·· | ·· |  | ·· | ·· | ·· |  | ·· | ·· | ·· |  |
| Rwanda | 5783 | 117 | 2024 | ·· | ·· | ·· |  | ·· | ·· | ·· |  | ·· | ·· | ·· |  |
| Sao Tome & Principe | 12 | 19 | 2023 | ·· | ·· | ·· |  | ·· | ·· | ·· |  | ·· | ·· | ·· |  |
| Senegal | 343 | 7 | 2023 | ·· | ·· | ·· |  | 5·4 (1·5, 11·1) | < 500 (<500,<500) | 2019 | 176 | ·· | ·· | ·· |  |
| Seychelles | 29 | 104 | 2024 | ·· | ·· | ·· |  | ·· | ·· | ·· |  | ·· | ·· | ·· |  |
| Sierra Leone | 156 | 3 | 2024 | ·· | ·· | ·· |  | 10·4 (4·4, 18·6) | < 500 (<500,<500) | 2021 | 177 | ·· | ·· | ·· |  |
| Somalia | 104 | 0 | 2023 | ·· | ·· | ·· |  | ·· | ·· | ·· |  | ·· | ·· | ·· |  |
| South Africa | 4712 | 24 | 2023 | ·· | ·· | ·· |  | 34·5 (14·3, 63·7) | 1500 (500,3000) | 2017-20 | 178,179 | ·· | ·· | ·· |  |
| United Republic of Tanzania | 1111 | 6 | 2022 | ·· | ·· | ·· |  | 14·6 (10·3, 20·2) | < 500 (<500,<500) | 2012 | 180 | ·· | ·· | ·· |  |
| Togo | 130 | 5 | 2021 | ·· | ·· | ·· |  | ·· | ·· | ·· |  | ·· | ·· | ·· |  |
| Uganda | 3770 | 30 | 2024 | ·· | ·· | ·· |  | 12·7 (9·6, 16·3) | 500 (500,500) | 2008 | 181 | ·· | ·· | ·· |  |
| Zambia | 931 | 14 | 2024 | ·· | ·· | ·· |  | 33·4 (30·8, 36·2) | 500 (500,500) | 1017-19 | 179,182-184 | 13·7 (7·7, 21·2) | < 500 (<500,<500) | 2011 | 185 |
| Zimbabwe | 651 | 9 | 2024 | ·· | ·· | ·· |  | ·· | ·· | ·· |  | ·· | ·· | ·· |  |
| **Middle East & North Africa** |  |  |  |  |  |  |  |  |  |  |  |  |  |  |  |
| Algeria | 1421 | 10 | 2021 | ·· | ·· | ·· |  | ·· | ·· | ·· |  | ·· | ·· | ·· |  |
| Bahrain | 164 | 43 | 2017 | ·· | ·· | ·· |  | ·· | ·· | ·· |  | ·· | ·· | ·· |  |
| Cyprus | 89 | 22 | 2024 | ·· | ·· | ·· |  | ·· | ·· | ·· |  | ·· | ·· | ·· |  |
| Egypt | 4440 | 13 | 2022 | ·· | ·· | ·· |  | ·· | ·· | ·· |  | ·· | ·· | ·· |  |
| Iraq | 1917 | 15 | 2021 | ·· | ·· | ·· |  | ·· | ·· | ·· |  | ·· | ·· | ·· |  |
| Israel | 198 | 7 | 2023 | ·· | ·· | ·· |  | ·· | ·· | ·· |  | ·· | ·· | ·· |  |
| Jordan | 421 | 13 | 2022 | ·· | ·· | ·· |  | ·· | ·· | ·· |  | ·· | ·· | ·· |  |
| Kuwait | 307 | 26 | 2024 | ·· | ·· | ·· |  | ·· | ·· | ·· |  | ·· | ·· | ·· |  |
| Lebanon | 268 | 15 | 2023 | ·· | ·· | ·· |  | ·· | ·· | ·· |  | ·· | ·· | ·· |  |
| Libya | 210 | 10 | 2023 | ·· | ·· | ·· |  | ·· | ·· | ·· |  | ·· | ·· | ·· |  |
| Morocco | 2566 | 20 | 2023 | ·· | ·· | ·· |  | 1·8 (0·4, 4·2) | < 500 (<500,<500) | 2004 | 186 | ·· | ·· | ·· |  |
| Oman | 74 | 8 | 2015 | ·· | ·· | ·· |  | ·· | ·· | ·· |  | ·· | ·· | ·· |  |
| Occupied Palestinian territories | ·· | ·· | N/A | ·· | ·· | ·· |  | ·· | ·· | ·· |  | ·· | ·· | ·· |  |
| Qatar | 60 | 19 | 2022 | ·· | ·· | ·· |  | ·· | ·· | ·· |  | ·· | ·· | ·· |  |
| Saudi Arabia | 1293 | 13 | 2017 | ·· | ·· | ·· |  | ·· | ·· | ·· |  | 1·5 (0·2, 3·7) | < 500 (<500,<500) | 2022 | 187 |
| South Sudan | 916 | 32 | 2021 | NK | NK | ·· |  | ·· | ·· | ·· |  | ·· | ·· | ·· |  |
| Sudan | 357 | 3 | 2017 | ·· | ·· | ·· |  | ·· | ·· | ·· |  | ·· | ·· | ·· |  |
| Syrian Arab Republic | 784 | 12 | 2004 | ·· | ·· | ·· |  | ·· | ·· | ·· |  | ·· | ·· | ·· |  |
| Tunisia | 775 | 19 | 2021 | ·· | ·· | ·· |  | ·· | ·· | ·· |  | ·· | ·· | ·· |  |
| Türkiye | 16350 | 55 | 2024 | ·· | ·· | ·· |  | ·· | ·· | ·· |  | 16·2 (11·4, 23·0) | 2500 (2000,4000) | 2016 | 188 |
| United Arab Emirates | 1150 | 55 | 2014 | ·· | ·· | ·· |  | ·· | ·· | ·· |  | ·· | ·· | ·· |  |
| Yemen | 55 | 1 | 2022 | ·· | ·· | ·· |  | ·· | ·· | ·· |  | ·· | ·· | ·· |  |

**Notes:**

Ns are rounded to the nearest 500.

^1^ Country level data that informed these regional and global incarceration estimates were sourced from the World Prison Brief, collated by the Institute for Crime and Justice Policy Research at Burbeck University. See: https://www.prisonstudies.org/world-prison-brief-data. Note that we used the country estimates to make rates for 15-64 years (not the total country population), so our rates differ from the World Prison Brief estimates.

* Estimates of the prison population total range between 80,000 and 120,000.

† For reporting purposes, these countries or territories are reported separately due to differences in service provision.

‡ The following sources were found for HIV prevalence in women in Australian prisons, but due to the quality of the AusHep^159^ study we did not include them in the meta analysis: ^153,159,189,190^

§ The following sources were found for HCV prevalence in women in Australian prisons, but due to the quality of the AusHep^159^ study we did not include them in the meta analysis: ^153,155,159,189-194^

** The HIV and HCV estimates weren't included in global and regional estimates as we could not proportionally weight the estimate to be included into global and regional estimates (due to no reporting of total incarcerated population by the World Prison Brief).

·· Indicates that no estimates of the prevalence for that outcome were obtained for that country.

NK Indicates no evidence was located that injecting drug use was occurring in this country.

HCV – Hepatitis C

Please see Appendix 5-8 for details of approach to assessment of study methodology and approach to selection and synthesis of data.

* Estimates of the prison population total range between 80,000 and 120,000.

**References for Table 9.3**

1. Azbel L, Wickersham JA, Wegman MP, et al. Burden of substance use disorders, mental illness, and correlates of infectious diseases among soon-to-be released prisoners in Azerbaijan. *Drug and Alcohol Dependence* 2015.

2. Popov G, Plochev K. Prevalence and correlates of hepatitis C virus infection among inmates of Bulgarian prisons. *Clinical Microbiology and Infection* 2011.

3. Kivimets K, Uuskula A. HIV testing and counselling in Estonian prisons, 2012 to 2013: aims, processes and impacts. *Euro Surveill* 2014.

4. Gyarmathy VA, Neaigus A, Szamado S. HIV risk behavior history of prison inmates in Hungary. *AIDS Education and Prevention* 2003.

5. Kurcalte O SM, Manson E, Karadzhan J, Krastiÿš I, & Zalans O. Analysis of trends in the use of addictive substances in prisons in Latvia in 2022": Final report, 2023.

6. Narkauskaitė L, Juozulynas A, Mackiewicz Z, Venalis A, Utkuvienė J. Prevalence of psychoactive substances use in a Lithuanian women's prison revisited after 5 years. *Medical Science Monitor* 2010.

7. Azbel L, Wickersham JA, Grishaev Y, Dvoryak S, Altice FL. Burden of infectious diseases, substance use disorders, and mental illness among Ukrainian prisoners transitioning to the community. *PLoS One* 2013.

8. Balakireva O SV, Salabai N, Kryvoruk A. Analysis of HIV/AIDS Response in Penitentiary System of Ukraine: Ukrainian Institute for Social Research after Olexander Yaremenko; UNODC, 2012.

9. Osinskaya T, Zapolsky M, Shcherbakova Y, Dzhoraieva S. Prevalence of Chlamydia among Women in Places of Deprivation of Liberty. *Georgian Medical News* 2023; (337): 34-7.

10. Busschots D, Kremer C, Bielen R, et al. A multicentre interventional study to assess blood-borne viral infections in Belgian prisons. *BMC Infectious Diseases* 2021.

11. Burek V, Horvat J, Butorac K, Mikulić R. Viral hepatitis B, C and HIV infection in Croatian prisons. *Epidemiology & Infection* 2010.

12. Burek V, Horvat J, Susic E, Mikulic R. Prevalence of hepatitis B and C among prison population in Croatia. [Croatian]. *Acta Medica Croatica* 2009.

13. Mahto M, Zia S. Measuring the gap: from Home Office to the National Health Service in the provision of a one-stop shop sexual health service in a female prison in the UK. *International Journal of STD & AIDS* 2008.

14. Plugge E, Yudkin P, Douglas N. Changes in women's use of illicit drugs following imprisonment. *Addiction* 2009.

15. Weild AR, Gill ON, Bennett D, Livingstone SJ, Parry JV, Curran L. Prevalence of HIV, hepatitis B, and hepatitis C antibodies in prisoners in England and Wales: a national survey. *Communicable Disease and Public Health* 2000.

16. Phaw NA, Thant AM, Thompson C, et al. Prospective evaluation of the impact of repeated whole prison testing for hepatitis C. *BMJ Open Gastroenterol* 2025; **12**(1).

17. Allsop C, McCullough F, Miller C, et al. Impact of a 'high intensity test and treat' initiative for Hcv in low newton prison. *Gut* 2021.

18. Halford R, Christensen L, Cox S, et al. Chronic hepatitis C elimination prison initiative: HCV-intensive test and treat, a whole prisoner population HCV test-and-treat program in England. *Health Science Reports* 2023; **6(12) (no pagination)**.

19. Kirwan P, Evans B, Brant L, Sentinel Surveillance Hepatitis T. Hepatitis C and B testing in English prisons is low but increasing. *Journal of Public Health* 2011.

20. Rautanen M, Harald, K, & Tyni, S. Health and Wellbeing of Prisoners 2023 The Wattu IV Prison Population Study Finland, 2024.

21. Viitanen P, Vartiainen H, Aarnio J, et al. Hepatitis A, B, C and HIV infections among Finnish female prisoners--young females a risk group. *Journal of Infection* 2011.

22. Rautanen M, Harald, K, & Tyni, S. The Health and Wellbeing of Finnish Prisoners 2023 (Wattu IV). *Finnish institute for health and welfare (THL) Report 007/2023 256 pages Helsinki 2023* 2023.

23. Messiah A, Escaffre N, Sannino N, Rotily M, Galinier-Pujol A. Sexuality in the age of AIDS in a vulnerable population: findings from a survey on prisoners. *Population* 2001.

24. Allwright S, Bradley F, Long J, Barry J, Thornton L, Parry JV. Prevalence of antibodies to hepatitis B, hepatitis C, and HIV and risk factors in Irish prisoners: results of a national cross sectional survey. *BMJ* 2000.

25. Drummond A, Codd M, Donnelly N, et al. Study on the prevalence of drug use, including intravenous drug use, and blood-borne viruses among the Irish prisoner population. *Dublin: National Advisory Committee on Drugs and Alcohol* 2014.

26. Wright B, Duffy D, Curtin K, Linehan S, Monks S, Kennedy HG. Psychiatric morbidity among women prisoners newly committed and amongst remanded and sentenced women in the Irish prison system. *Irish Journal of Psychological Medicine* 2006.

27. Long J, Allwright S, Barry J, et al. Prevalence of antibodies to hepatitis B, hepatitis C, and HIV and risk factors in entrants to Irish prisons: a national cross sectional survey. *BMJ* 2001.

28. Fiore V, De Vito A, Rastrelli E, et al. Differences in HCV Seroprevalence, Clinical Features, and Treatment Outcomes between Female and Male Incarcerated Population: Results from a Matched Cohort Study. *Viruses* 2023; **15**(12).

29. Marco LD, Tullio P, Scalici F, et al. Screening and linkage to care of prisoners with HCV infection: the resist-HCV project. *Journal of Hepatology* 2020.

30. Scelza G, Amato A, Pagano AM, et al. Effect of hepatitis C antiviral therapy on oral lichen planus and hyposalivation in inmates. *Annals of Gastroenterology* 2022.

31. Babudieri S, Longo B, Sarmati L, et al. Correlates of HIV, HBV, and HCV infections in a prison inmate population: results from a multicentre study in Italy. *Journal of Medical Virology* 2005.

32. Geremia N, Giovagnorio F, De Vito A, et al. HBV in Italian Women's Jail: An Underestimated Problem? *Journal Of Clinical Medicine* 2024; **13(5) (no pagination)**.

33. Brandolini M, Novati, S., De Silvestri A, Tinelli C, Patruno SFA, Ranieri R, Seminari E. Prevalence and epidemiological correlates and treatment outcome of HCV infection in an Italian prison setting. *BMC Public Health* 2013.

34. Di Marco L, Cartabellotta F, Santangelo F, et al. Eliminating HCV infection from prisons in sicily: the SINTESI project. *Digestive and Liver Disease* 2024; **56(Supplement 1)**: S15.

35. Izzo C, Masarone M, Torre P, et al. Solving the Gap Between HCV Detection and Treatment in Prison HCV-RNA Testing and Treatment in a Cohort of Newly Arrived Convicts in Southern Italy. *Reviews on Recent Clinical Trials* 2022.

36. Bakić M, Stevanović J, Milić M, et al. Factors associated with the prevalence of viral hepatitis B and C among prisoners: Results of two consecutive national surveys in Montenegro. *PLoS One* 2025; **20**(4): e0321464.

37. Danis K, Doherty L, McCartney M, McCarrol J, Kennedy H. Hepatitis and HIV in Northern Ireland prisons: a cross-sectional study. *Euro Surveill* 2007; **12**(1).

38. Bukten A, Lund IO, Kinner SA, et al. Factors associated with drug use in prison – results from the Norwegian offender mental health and addiction (NorMA) study. *Health & Justice* 2020.

39. Barros H, Ramos E, Lucas R. A survey of HIV and HCV among female prison inmates in Portugal. *Central Europena Journal of Public Health* 2008.

40. Garcia A, Exposto F, Prieto E, Lopes M, Duarte A, da Silva RC. Association of Trichomonas vaginalis with sociodemographic factors and other STDs among females inmates in Lisbon. *International Journal of STD & AIDS* 2004.

41. Morgado D, Alves J, Martinho G, Gonçalves M, Cerqueira A, Maia A. Concordance Between Health Records and a Self-Report Measure in a Sample of Female Inmates in Portugal. *Women & Criminal Justice* 2025.

42. Passadouro R. [Prevalence infections and risk factors due to HIV, Hepatitis B and C in a prison establishment in Leiria]. *Acta Medica Portuguesa* 2004.

43. Taylor A, Munro A, Allen E, et al. Low incidence of hepatitis C virus among prisoners in Scotland. [References]. *Addiction* 2013.

44. Gonzalez C, Canals J, Ortiz M, et al. Prevalence and determinants of high-risk human papillomavirus (HPV) infection and cervical cytological abnormalities in imprisoned women. *Epidemiology and Infection* 2008.

45. Serroukh SC-Y. Consumo de sustancias, tratamiento con psicofármacos y patología infecciosa en personas redusas del centro penitenciario Puig de las Basses. *Metas de Enfermería* 2022.

46. de la Hoya PS, Marco A, Garcia-Guerrero J, Rivera A, Prevalhep Study G. Hepatitis C and B prevalence in Spanish prisons. *European Journal of Clinical Microbiology & Infectious Diseases* 2011.

47. Gahrton C, Westman G, Lindahl K, et al. Prevalence of Viremic hepatitis C, hepatitis B, and HIV infection, and vaccination status among prisoners in Stockholm County. *BMC Infectious Diseases* 2019.

48. Moschetti K, Stadelmann P, Wangmo T, et al. Disease profiles of detainees in the Canton of Vaud in Switzerland: gender and age differences in substance abuse, mental health and chronic health conditions. *BMC Public Health* 2015.

49. Blogg S, Utomo, B,, Silitonga N, Hidayati DAN, Sattler G. Indonesian National Inmate Bio-Behavioral Survey for HIV and Syphilis Prevalence and Risk Behaviors in Prisons and Detention Centers, 2010. *SAGE Open* 2014.

50. Arends RM, Nelwan EJ, Soediro R, et al. Associations between impulsivity, risk behavior and HIV, HBV, HCV and syphilis seroprevalence among female prisoners in Indonesia: A cross-sectional study. *PLoS One* 2019.

51. Mwe Nom NA, Kyaw KWY, Kumar AMV, et al. HIV care cascade among prisoners of the Mandalay Central Prison in Myanmar: 2011-2018. *Tropical Medicine and Infectious Disease* 2020.

52. Simbulan NP, Aguilar AS, Flanigan T, Cu-Uvin S. High-risk behaviors and the prevalence of sexually transmitted diseases among women prisoners at the women state penitentiary in Metro Manila. *Social Science & Medicine* 2001.

53. Lin CF, Twu SJ, Chen PH, Cheng JS, Wang JD. Prevalence and determinants of hepatitis B antigenemia in 15,007 inmates in Taiwan. *Journal of Epidemiology* 2010.

54. Lu MY, Chen CT, Shih YL, et al. Changing epidemiology and viral interplay of hepatitis B, C and D among injecting drug user-dominant prisoners in Taiwan. *Scientific Reports* 2021.

55. Choudhury R, Singh N. Prevalence of HIV/AIDS in inmates of two district jails of central Uttar Pradesh, India. *Medico-Legal Update* 2016.

56. Ramamoorthy M, Venketeswaran A, Seenivasan P, et al. Risk factors and prevalence, hepatitis B virus and hepatitis C virus among prison inmates, Chennai, India, 2015. 2016; **53**: 90.

57. Singh V, Kaur A, Kumari S, et al. Seroprevalence of HCV and Transient Elastography in a Correctional Setting. HEPATOLOGY; 2017: WILEY 111 RIVER ST, HOBOKEN 07030-5774, NJ USA; 2017. p. 558A-A.

58. Tyagi SK, Sovani V, Dias NP, Tyagi D, Saxena S. Prevalence and risk factors of HCV infection in a prison setting in Uttar Pradesh, India. *Indian Journal of Public Health Research and Development* 2018.

59. National HIV bio­behavioral Surveillance Survey (BSS) in prisoners. 2009.

60. Ataei B, Khorvash F, Azadeh S, Nokhodian Z, Kassaian N, Babak A. The prevalence of high risk behaviors among women prisoners in Isfahan, Iran. *Journal of Isfahan Medical School* 2011.

61. Mirzazadeh A, Shokoohi M, Navadeh S, et al. Underreporting in HIV-Related High-Risk Behaviors: Comparing the Results of Multiple Data Collection Methods in a Behavioral Survey of Prisoners in Iran. *Prison Journal* 2018.

62. Nokhodian Z, Yazdani MR, Yaran M, et al. Prevalence and risk factors of HIV, syphilis, hepatitis B and C among female prisoners in Isfahan, Iran. *Hepatitis Monthly* 2012.

63. Shahesmaeili A, Karamouzian M, Tavakoli F, et al. HIV prevalence and continuum of care among incarcerated people in Iran from 2010 to 2017. *Harm Reduction Journal* 2022.

64. Shahesmaeili A, Karamouzian M, Tavakoli F, et al. HIV prevalence and continuum of care among incarcerated people in Iran from 2010 to 2017. *Harm Reduction Journal* 2022; **19**(1).

65. Khajedaluee M, Babaei A, Vakili R, et al. Sero-prevalence of bloodborne tumor viruses (HCV, HBV, HTLV-I and KSHV infections) and related risk factors among prisoners in Razavi Khorasan province, Iran, in 2008. *Hepatitis Monthly* 2016.

66. Moradi G, Alavian SM, Gholami F, et al. Prevalence of hepatitis B and hepatitis C infections among incarcerated individuals in Iran: A cross-sectional national bio-behavioral study in 2019. *Pathogens* 2021.

67. Moradi G, Gouya MM, Zavareh FA, et al. Prevalence and risk factors for HBV and HCV in prisoners in Iran: a national bio-behavioural surveillance survey in 2015. *Tropical Medicine & International Health* 2018.

68. Moradi G, Jafari S, Zarei B, et al. Prevalence and Risk Factors for Hepatitis B and Hepatitis C Exposure in Iranian Prisoners: A National Study in 2016. *Hepatitis Monthly* 2019.

69. Ziaee M, Sharifzadeh G, Namaee MH, Fereidouni M. Prevalence of HIV and Hepatitis B, C, D Infections and Their Associated Risk Factors among Prisoners in Southern Khorasan Province, Iran. *Iranian Journal of Public Health* 2014.

70. Nafees M, Qasim A, Jafferi G, Anwar MS, Muazzam M. HIV infection, HIV/HCV and HIV/HBV co-infections among jail inmates of Lahore. *Pakistan Journal of Medical Sciences* 2011.

71. Pervaiz A, Ghafoor T, Asghar RJ. Screening of prisoners for Human Immunodeficiency Virus (HIV), Hepatitis C (HCV) and B (HBV) in Punjab Province, Pakistan, 2009. *International Journal of Infectious Diseases* 2012.

72. Niriella MA, Hapangama A, Luke H, Pathmeswaran A, Kuruppuarachchi K, de Silva HJ. Prevalence of hepatitis B and hepatitis C infections and their relationship to injectable drug use in a cohort of Sri Lankan prison inmates. *Ceylon Medical Journal* 2015.

73. Adaszko D, Sotelo JA, Orlando M, Angelerei P. Estudio de prevalencia de VIH, sífilis, hepatitis virales y tuberculosis en personas en contextos de encierro en unidades del Servicio Penitenciario Federal. *Buenos Aires, Ministerio de Salud* 2017.

74. Lambert ML, Torrico F, Billot C, Mazina D, Marleen B, Van der Stuyft P. Street youths are the only high-risk group for HIV in a low-prevalence South American country. *Sexually Transmitted Diseases* 2005; **32**(4): 240-2.

75. Villarroel-Torrico M, Montaño K, Flores-Arispe P, et al. Syphilis, human immunodeficiency virus, herpes genital and hepatitis B in a women's prison in Cochabamba, Bolivia: prevalence and risk factors. *Revista Espanola de Sanidad Penitenciaria* 2018.

76. Lopes F, Latorre MR, Campos Pignatari AC, Buchalla CM. [HIV, HPV, and syphilis prevalence in a women's penitentiary in the city of São Paulo, 1997-1998]. *Cadernos de Saude Publica* 2001.

77. Miranda AE, Vargas PM, St Louis ME, Viana MC. Sexually transmitted diseases among female prisoners in Brazil - Prevalence and risk factors. *Sexually Transmitted Diseases* 2000.

78. Strazza L, Massad E, Azevedo RS, Carvalho HB. Behavior associated with HIV and HCV infection in female prison inmates in Sao Paulo, Brazil. *Cadernos de Saude Publica* 2007.

79. Strazza L, Azevedo RS, Carvalho HB, Massad E. The vulnerability of Brazilian female prisoners to HIV infection. *Brazilian Joiurnal of Medical and Biological Research* 2004.

80. de Navarro PD, de Almeida IN, Kritski AL, et al. Prevalence of latent Mycobacterium tuberculosis infection in prisoners. *Jornal Brasileiro De Pneumologia* 2016; **42**(5): 348-55.

81. Benedetti MSG, Nogami ASA, da Costa BB, et al. Sexually transmitted infections in women deprived of liberty in Roraima, Brazil. *Revista de Saude Publica* 2020.

82. Kerr L, Smith DG, Kendall C, et al. HIV testing inside Brazilian female prisons: results of a national survey. *AIDS Care* 2023; **35**(6): 841-9.

83. Leal M, Kerr L, Mota RMS, Neto RDP, Seal D, Kendall C. Health of female prisoners in Brazil. *Ciencia & Saude Coletiva* 2022.

84. Miranda AE, Vargas, P.M., Louis, M.E.S. & Viana, M.C. Sexually transmitted diseases among female prisoners in Brazil: prevalence and risk factors. *Sexually Transmitted Diseases* 2000; **27**(9): 491-5.

85. Sgarbi RVE, Carbone ADS, Paiao DSG, et al. A Cross-Sectional Survey of HIV Testing and Prevalence in Twelve Brazilian Correctional Facilities. *PLoS One* 2015.

86. Sousa KAA, Araujo TME, Teles SA, Rangel EML, Nery IS. Factors associated with HIV prevalence in a prison population. *Revista da Escola de Enfermagem da U S P* 2017.

87. Vale EP, Carvalho LD, Pereira FCD. HIV seroprevalence in prisoners in Amapascritores. *Revista de Epidemiologia e Controle de Infeccao* 2016.

88. de Andrade FM, de Amorim Andrade SG, Araujo Júnior E, et al. Pap smear and colposcopy findings in female inmates of a prison unit in the state of São Paulo, Brazil. *Ceska Gynekol* 2024; **89**(6): 459-68.

89. Barros LAS, Pessoni GC, Teles SA, et al. Epidemiology of the viral hepatitis B and C in female prisoners of Metropolitan Regional Prison Complex in the State of Goias, Central Brazil. *Revista da Sociedade Brasileira de Medicina Tropical* 2013.

90. Pinheiro DM, da Silva Souza AT, Alencar DdC, et al. Prevalencia de Anti-Hcv e Fatores Associados em Detentos de Unidades Prisionais. *Enfermagem em Foco* 2024; **15**: S50-S7.

91. Pompilio MA, Pontes ERJC, Castro ARCM, et al. Prevalence and epidemiology of chronic hepatitis c among prisoners of Mato Grosso do Sul State, Brazil. *Journal of Venomous Animals and Toxins Including Tropical Diseases* 2011.

92. Puga MAM, Bandeira LM, Pompilio MA, et al. Prevalence and Incidence of HCV Infection among Prisoners in Central Brazil. *PLoS One* 2017.

93. Santos BFO, de Santana NO, Franca AVC. Prevalence, genotypes and factors associated with HCV infection among prisoners in Northeastern Brazil. *World Journal of Gastroenterology* 2011.

94. Santos Barros LA, Carolina Pessoni G, Araujo Teles S, et al. Epidemiology of the viral hepatitis B and C in female prisoners of metropolitan regional prison complex in the State of Goias, Central Brazil. *Revista da Sociedade Brasileira de Medicina Tropical* 2013.

95. Bautista-Arredondo S, González A, Servan-Mori E, et al. A Cross-Sectional Study of Prisoners in Mexico City Comparing Prevalence of Transmissible Infections and Chronic Diseases with That in the General Population. *PLoS One* 2015.

96. Gonzalez CAM, Ortiz BES, Aguilar MB, Gonzalez JDM. Risk factors and the seroprevalence of viral markers of hepatitis B (HVB) and hepatitis C (HCV) in high-risk groups in Chiapas. *Medwave* 2011.

97. Cyrus E, Sanchez J, Madhivanan P, et al. Prevalence of Intimate Partner Violence, Substance Use Disorders and Depression among Incarcerated Women in Lima, Perú. *International Journal of Environmental Research and Public Health* 2021.

98. Garaycochea MC, Pino R, Chavez I, et al. Sexually transmitted infections in women living in a prison in Lima, Peru. *Revista Peruana de Medicina Experimental y Salud Publica* 2013.

99. Alcivar JC, Zambrano MM, Madronero MG, et al. Sexually transmitted infections in inmates in Merida Venezuela. *Investigacion Clinica* 2020.

100. Gil YMF. Estudio de prevalencia de VIH en población de Personas Privada de Libertad en 30 establecimientos penitenciarios de la República Bolivariana de Venezuela, 2022. In: Salud MdPPpl, editor.; 2022.

101. Besney JD, Angel C, Pyne D, Martell R, Keenan L, Ahmed R. Addressing Women’s Unmet Health Care Needs in a Canadian Remand Center. *Journal of Correctional Health Care* 2018.

102. Courtemanche Y, Poulin C, Serhir B, Alary M. HIV and hepatitis C virus infections in Quebec's provincial detention centres: comparing prevalence and related risky behaviours between 2003 and 2014-2015. *Canadian Journal of Public Health* 2018.

103. Martin RE, Remple V, Gold F, Berkowitz J, Murphy W, Money D. Drug use and risk of bloodborne infections: A survey of female prisoners in British Columbia. *Canadian Journal of Public Health-Revue Canadienne de Sante Publique* 2005.

104. Nolan AM, Stewart LA. Chronic Health Conditions Among Incoming Canadian Federally Sentenced Women. *Journal of Correctional Health Care* 2017.

105. Poulin C, Alary M, Lambert G, et al. Prevalence of HIV and hepatitis C virus infections among inmates of Quebec provincial prisons. [References]. *Canadian Medical Association Journal* 2007.

106. Abiona TC, Balogun JA, Adefuye AS, Sloan PE. Pre-incarceration HIV risk behaviours of male and female inmates. [References]. *International Journal of Prisoner Health* 2009.

107. Abiona TC, Adefuye AS, Balogun JA, Sloan PE. Gender differences in HIV risk behaviors of inmates. *Journal of Womens Health (Larchmt)* 2009.

108. Altice FL, Marinovich A, Khoshnood K, Blankenship KM, Springer SA, Selwyn PA. Correlates of HIV infection among incarcerated women: implications for improving detection of HIV infection. *Journal of Urban Health* 2005.

109. Alvarez KJ, Befus M, Herzig CTA, Larson E. Prevalence and correlates of hepatitis C virus infection among inmates at two New York State correctional facilities. *Journal of Infection and Public Health* 2014.

110. Harrison LD, Bachman T, Freeman C, Inciardi JA. The acceptability of the female condom among US women at high risk from HIV. [References]. *Culture, Health & Sexuality* 2001.

111. Keleekai NL. Patterns and predictors of HIV, sexually transmitted infections, and staphylococcus aureus co-infection among New York state prison inmates. *Dissertation Abstracts International: Section B: The Sciences and Engineering* 2012.

112. Kendrick SR, Kroc KA, Couture E, Weinstein RA. Comparison of point-of-care rapid HIV testing in three clinical venues. *Aids* 2004.

113. Macalino GE, Dhawan D, Rich JD. A missed opportunity: hepatitis C screening of prisoners. *American Journal of Public Health* 2005.

114. McClelland GM, Teplin LA, Abram KM, Jacobs N. HIV and AIDS risk behaviors among female jail detainees: Implications for public heath policy. [References]. *American Journal of Public Health* 2002.

115. Mullings JL, Marquart JW, Hartley DJ. Exploring the effects of childhood sexual abuse and its impact on HIV/AIDS risk-taking behavior among women prisoners. *Prison Journal* 2003.

116. Mullings JL, Marquart JW, Diamond PM. Cumulative continuity and injection drug use among women: A test of the downward spiral framework. [References]. *Deviant Behavior* 2001.

117. Trevino S. The relationship between age of first reported trauma and substance specific use in incarcerated women. *Dissertation Abstracts International: Section B: The Sciences and Engineering* 2013.

118. Arriola KR, Braithwaite RL, Kennedy S, et al. A collaborative effort to enhance HIV/STI screening in five county jails. *Public Health Reports* 2001.

119. Baillargeon J, Black SA, Pulvino J, Dunn K. The disease profile of Texas prison inmates. *Annals of Epidemiology* 2000.

120. Baillargeon J, Pulvino JS, Leonardson JE, et al. The changing epidemiology of HIV in the criminal justice system. *International Journal of STD & AIDS* 2017.

121. Baillargeon J, Wu H, Kelley MJ, Grady J, Linthicum L, Dunn K. Hepatitis C seroprevalence among newly incarcerated inmates in the Texas correctional system. *Public Health* 2003.

122. Baillargeon JG, Paar DP, Wu H, et al. Psychiatric disorders, HIV infection and HIV/hepatitis co-infection in the correctional setting. [References]. *AIDS Care* 2008.

123. Bauserman RL, Ward MA, Eldred L, Swetz A. Increasing voluntary HIV testing by offering oral tests in incarcerated populations. *American Journal of Public Health* 2001.

124. Begier EM, Bennani Y, Forgione L, et al. Undiagnosed HIV infection among New York City jail entrants, 2006: results of a blinded serosurvey. *Journal of Acquired Immune Deficiency Syndromes* 2010.

125. Carvajal RI, Ross MW, Byrd T, Shelton A. HIV Counseling and Testing Program for Female Inmates: Analysis of Data From the Harris County Jail. [References]. *Journal of Correctional Health Care* 2005.

126. Desai J, Nijhawan A, Krakower D, Harris BL, Taherzadeh D. Hiv/sti testing and prep eligibility among women incarcerated in an urban county jail. *Topics in Antiviral Medicine* 2021.

127. Feld S, Steele J, Klinedinst S, et al. Implementing Opt-Out HIV Testing in the Alameda County Jails. *Journal of correctional health care : the official journal of the National Commission on Correctional Health Care* 2023.

128. Javanbakht M, Boudov M, Anderson LJ, et al. Sexually transmitted infections among incarcerated women: Findings from a decade of screening in a Los Angeles County jail, 2002-2012. [References]. *American Journal of Public Health* 2014.

129. Katyal M, Leibowitz R, Venters H. IGRA-Based Screening for Latent Tuberculosis Infection in Persons Newly Incarcerated in New York City Jails. *Journal of Correctional Health Care* 2018.

130. Kavasery R, Maru DS, Cornman-Homonoff J, Sylla LN, Smith D, Altice FL. Routine opt-out HIV testing strategies in a female jail setting: a prospective controlled trial. *PLoS One* 2009.

131. Lucas KD, Eckert V, Behrends CN, Wheeler C, MacGowan RJ, Mohle-Boetani JC. Evaluation of Routine HIV Opt-Out Screening and Continuum of Care Services Following Entry into Eight Prison Reception Centers--California, 2012. *Morbidity and Mortality Weekly Report* 2016.

132. MacGowan R, Margolis A, Richardson-Moore A, et al. Voluntary Rapid Human Immunodeficiency Virus (HIV) Testing in Jails. *Sexually Transmitted Diseases* 2009.

133. Peter P. Impact of Opt-Out and Opt-in HIV testing and education program on discovering HIV in jail populations. *Dissertation Abstracts International: Section B: The Sciences and Engineering* 2013.

134. Rice DK. Design, implementation, and evaluation of a jail-based HIV screening program. *Dissertation Abstracts International: Section B: The Sciences and Engineering* 2011.

135. Rosen DL, Schoenbach VJ, Wohl DA, White BL, Stewart PW, Golin CE. Characteristics and behaviors associated with HIV infection among inmates in the North Carolina prison system. *American Journal of Public Health* 2009.

136. Ruiz JD, Molitor F, Plagenhoef JA. Trends in hepatitis C and HIV infection among inmates entering prisons in California, 1994 versus 1999. *AIDS* 2002.

137. Sampson LA. Screening for syphilis and HIV in North Carolina jails. *Dissertation Abstracts International: Section B: The Sciences and Engineering* 2009.

138. Scott J, Sampson LA, Clymore JM, Moore PR, Leone PA. Integrated HIV, syphilis, and other STI testing in North Carolina county jails. *Sexually Transmitted Infections* 2011.

139. Seth P, Figueroa A, Wang G, Reid L, Belcher L. HIV Testing, HIV Positivity, and Linkage and Referral Services in Correctional Facilities in the United States, 2009-2013. *Sexually Transmitted Diseases* 2015.

140. Simonson R, Koenigsberg B, Varela NG, et al. Jail Length of Stay Does Not Account for Gender Differences in Hepatitis C Treatment Initiation in the New York City Jail System, 2019-2023. *Hepatology* 2024; **80(Supplement 1)**: S1980-S1.

141. de Ravello L, Brantley MD, Lamarre M, Qayad MG, Aubert H, Beck-Sague C. Sexually transmitted infections and other health conditions of women entering prison in Georgia, 1998-1999. *Sexually Transmitted Diseases* 2005.

142. Abe CM, Aguwa M, Zhao M, Sullivan J, Porsa E, Nijhawan AE. Hepatitis C Virus Infection in the Dallas County Jail: Implications for Screening, Prevention, and Linkage to Care. *Public Health Reports* 2019.

143. Akiyama MJ, Kaba F, Rosner Z, et al. Correlates of hepatitis C virus infection in the targeted testing program of the New York city jail system: Epidemiologic patterns and priorities for action. *Public Health Reports* 2017.

144. Bai JR, Mukherjee DV, Befus M, Apa Z, Lowy FD, Larson EL. Concordance between medical records and interview data in correctional facilities. *BMC Medical Research Methodol* 2014.

145. Deb LC, Hove H, Miller TK, et al. Epidemiology of Hepatitis C virus infection among incarcerated populations in North Dakota. *PLoS One* 2022.

146. Fox RK, Currie SL, Evans J, et al. Hepatitis C virus infection among prisoners in the California State correctional system. *Clinical Infectious Diseases* 2005.

147. Hoff E, Warden A, Taylor R, Nijhawan AE. Hepatitis C Epidemiology in a Large Urban Jail: A Changing Demographic. *Public Health Reports* 2023.

148. Irvin R, Landry G, Jones MR, et al. High prevalence of hepatitis C virus infection among incarcerated persons: Results from the Louisiana Hepatitis C Elimination Plan's opt-out testing program in prisons. *Journal of Viral Hepatitis* 2024; **31**(7): 432-5.

149. Kuncio DE, Newbern EC, Fernandez-Viña MH, Herdman B, Johnson CC, Viner KM. Comparison of risk-based hepatitis C screening and the true seroprevalence in an urban prison system. *Journal of Urban Health* 2015.

150. Larney S, Mahowald MK, Scharff N, Flanigan TP, Beckwith CG, Zaller ND. Epidemiology of hepatitis C virus in Pennsylvania state prisons, 2004-2012: limitations of 1945-1965 birth cohort screening in correctional settings. *American Journal of Public Health* 2014.

151. Leukefeld C, Harp KLH, Webster M, Staton-Tindall M, Oser CB, Havens JR. Examining HCV and other risks among rural women offenders. *Drug and Alcohol Dependence* 2015.

152. Wenger PJ, Rottnek F, Parker T, Crippin JS. Assessment of hepatitis C risk factors and infection prevalence in a jail population. *American Journal of Public Health* 2014; **104**(9): 1722-7.

153. Butler TS, M. National Prison Entrants’ Bloodborne Virus and Risk Behaviour Survey Report: Kirby Institute 2017.

154. Butler T, Levy M, Dolan K, Kaldor J. Drug use and its correlates in an Australian prisoner population. *Addiction Research & Theory* 2003.

155. Hockings BA, Young, M., Falconer, A., and O'Rourke, P.K. Queensland Women Prisoners' Health Survey. Brisbane: Department of Corrective Services, 2002.

156. Kevin M. Drug Use in the Inmate Population–prevalence, nature and context. *DUIP NSW–6th Biennial data collection 2009‑10: Overview and series trend* 2013.

157. Kinner SA. The post-release experience of prisoners in Queensland. 2006.

158. Nicholson J, Almond L, Rizvi N, Fairley CK. Low prevalence of STIs among women in prison, but bacterial vaginosis is common. *Australian and New Zealand Journal of Public Health* 2003.

159. Bah R, Sheehan Y, Li X, et al. Prevalence of blood-borne virus infections and uptake of hepatitis C testing and treatment in Australian prisons: the AusHep study. *The Lancet Regional Health–Western Pacific* 2024; **53**.

160. Catraye DJ, Ky-Ba, A & Tavi-Ouattarra, A.Y. ENQUETE BIO COMPORTEMENTALE DU VIH-SIDA EN MILIEU CARCERAL AUPRES DES DETENUS HOMMES ET FEMMES AU BURKINA FASO. 2017.

161. Receveur MC, Seri B, Koffi A, et al. Prevalence of pulmonary tuberculosis among prison inmates: A cross-sectional survey at the Correctional and Detention Facility of Abidjan, Cote d'Ivoire. *Tropical Medicine and International Health* 2017.

162. Eritrea MoH. Report for HIV, HBsAg, HCV Ab and Syphilis Prevalence and HIV Risk Behavior Survey Among Prison Inmates in Eritrea. In: Division MoHDoPHCDC, editor.; 2015.

163. Dlamini P, Dlamini P, Mnisi Z, Hariga F. A situational assessment on TB, HIV, syphilis, hepatitis C and hepatitis B infections and associated risk behaviours among prisoners and prison officers in Swaziland; 2012.

164. Sahle ET, Amogne W, Manyazewal T, et al. Prevalence of and risk factors for Human Immunodeficiency Virus (HIV) infection in entrants and residents of an Ethiopian prison. *PLoS One* 2023.

165. Kassa Y, Million Y, Biset S, Moges F. Hepatitis b and hepatitis c viral infections and associated factors among prisoners in northeast ethiopia. *Journal of Blood Medicine* 2021.

166. Tadesse K, Ayalew G, Million Y, Gelaw A. Hepatitis B and hepatitis C virus infections and associated factors among prisoners in Gondar City, Northwest Ethiopia. *PLoS One* 2024; **19**(4): e0301973.

167. Tsegay B, Gebrecherkos T, Kahsay AG, Abdulkader M. Seroprevalence and Associated Factors of Hepatitis B and Hepatitis C Viral Infections Among Prisoners in Tigrai, Northern Ethiopia. *Infection and Drug Resistance* 2023; **16**: 3743-50.

168. Commission GA. National Health and HIV Survey of Prison Inmates in Ghana. 2013.

169. Adjei AA, Armah HB, Gbagbo F, et al. Correlates of HIV, HBV, HCV and syphilis infections among prison inmates and officers in Ghana: A national multicenter study. *BMC Infectious Diseases* 2008.

170. Sagoe KWC, Atuahene K, Ayiku ANA, et al. Hepatitis B and human immunodeficiency virus infections within correctional facilities in Ghana. *PLoS One* 2023; **18**(11): e0293009.

171. Adjei AA, Armah HB, Gbagbo F, et al. Correlates of hepatitis C virus infection among incarcerated Ghanaians: a national multicentre study. *Journal of Medical Microbiology* 2007.

172. Chimphambano C, Komolafe I, Muula A. Prevalence of HIV, HepBsAg and Hep C antibodies among inmates in Chichiri prison, Blantyre, Malawi. *Malawi Medical Journal* 2007; **19**(3): 107-10.

173. Lawrence QO, Amadi ANC, Okosa C, Ikpi PO, Chukwuemeka BC. Co-infection of Trichomonas vaginalis and HIV infection and its risk factors among prison inmates in Umuahia, Abia State, South Eastern Nigeria. *Journal of Basic and Applied Zoology* 2021.

174. Muhammed OT, Akpa OM, Atilola GO, Komolafe IOO. Seroprevalence of HIV/AIDS and HIV risk factors among prison inmates in Ogun State, Nigeria. *HIV & AIDS Review* 2012; **11**(1): 25-30.

175. Okafor IM, Ugwu SO, Okoroiwu HU. Hepatitis C virus infection and its associated factors among prisoners in a Nigerian prison. *BMC Gastroenterol* 2020.

176. Agency for the Promotion of Population Activities Senegal. ENQU bÊTE NATIO NALE DE SURVEILLANCE COMBINEE DES IST ET DU VIH/SIDA (ENSC 2019) COMPOSANTE COMPORTEMENTALE. In: SOCIALE MDLSEDLA, editor.; 2020.

177. Ampofo WK. Sierra Leone Integrated Bio-Behavioural Survey and Size Estimation Among Female Sex Workers (FSWs), Men who Have Sex with Men (MSM), Persons who Inject Drugs (PWID), Transgender (TG) and People in Close Settings (PCS). In: Secretariat NHA, editor.; 2021.

178. The Aurum Institute NICD. Socio-behavioural and structural factors driving HIV/AIDS, STIs and Hepatitis B & C infections among inmates in Correctional Facilities, Johannesburg, 2020.

179. Hoffmann CJ, Herce ME, Chimoyi L, et al. Reaching for 90:90:90 in Correctional Facilities in South Africa and Zambia: Virtual Cross-Section of Coverage of HIV Testing and Antiretroviral Therapy during Universal Test and Treat Implementation. *Journal of Acquired Immune Deficiency Syndromes* 2024; **96(5)**: 465-71.

180. Mutayoba B, Ngowi B, Kohi W. HIV prevalence and related risk factors in prison settings: findings from a rapid situational assessment in mainland Tanzania. 20th International AIDS Conference, Melbourne, Australia; 2014; 2014.

181. United Nations Office on Drugs and Crime. A Rapid Situation Assessment of HIV/STI/TB and Drug Abuse among Prisoners in Uganda Prisons Service, 2009.

182. Kagujje M, Somwe P, Hatwiinda S, et al. Cross-sectional assessment of tuberculosis and HIV prevalence in 13 correctional facilities in Zambia. *BMJ Open* 2021.

183. Simooya OO, Sanjobo NE, Kaetano L, et al. 'Behind walls': a study of HIV risk behaviours and seroprevalence in prisons in Zambia. *AIDS* 2001.

184. Simooya OO, Sanjobo N, Mulenga C, et al. Aggressive awareness campaigns may not be enough for HIV prevention in prisons-studies in Zambia suggest time for evidence based interventions. *Open Infectious Diseases Journal* 2014.

185. Maggard KR, Hatwiinda S, Harris JB, et al. Screening for tuberculosis and testing for human immunodeficiency virus in Zambian prisons. *Bulletin of the World Health Organization* 2015.

186. El Ghrari K, Terrab Z, Benchikhi H, Lakhdar H, Jroundi I, Bennani M. Prevalence of syphilis and HIV infection in female prisoners in Morocco. [French]. *Eastern Mediterranean Health Journal* 2007.

187. El-Daly MM, Fageeh W, El-Kafrawy SA, et al. Hepatitis B and Hepatitis C Infections among Female Inmates in a Prison in Jeddah, Saudi Arabia. *Clinical Laboratory* 2024; **70(3)**: 571-8.

188. ÖZger HS, KaraŞAhİN Ö, Toy MA, Yilmaz Sİ, Hizel K. Hepatitis C Prevalence and Responses to Pegylated Interferon + Ribavirin Treatment Among Prisoners. *Viral Hepatitis Journal / Viral Hepatit Dergisi* 2017.

189. Butler T, Boonwaat L, Hailstone S, et al. The 2004 Australian prison entrants' blood-borne virus and risk behaviour survey. *Australian and New Zealand Journal of Public Health* 2007.

190. Health J. National Patient Health Survey. 2017.

191. Butler T, Spencer J, Cui J, Vickery K, Zou J, Kaldor J. Seroprevalence of markers for hepatitis B, C and G in male and female prisoners‐NSW, 1996. *Australian and New Zealand Journal of Public Health* 1999; **23**(4): 377-84.

192. Hellard ME, Hocking JS, Crofts N. The prevalence and the risk behaviours associated with the transmission of hepatitis C virus in Australian correctional facilities. *Epidemiology & Infection* 2004.

193. Miller ER, Bi P, Ryan P. The prevalence of HCV antibody in South Australian prisoners. *Journal of Infection* 2006.

194. Wallis C, O'Flynn M, Fenech M, Grimstrup D. Hepatitis C virus point-of-care RNA testing: Experience from screening an entire high-security Australian prison population over 3 days. *Australian and New Zealand Journal of Public Health* 2023; **47(5)**: 100083.

## *Table 9.4*: Country-level estimates of the number and rate of incarceration, prevalence of HBV and tuberculosis among females who are incarcerated

|  | **Females who are incarcerated** | | | **Females with current HBV** | | | | **Females with active Tuberculosis** | | | |
| --- | --- | --- | --- | --- | --- | --- | --- | --- | --- | --- | --- |
| **Country** | **Estimated number^1^** | **Rate per 100,000** | **Year of estimate^1^** | **% (CI)** | **Estimated no. (CI)** | **Year of estimate** | **Sources** | **% (CI)** | **Estimated no. (CI)** | **Year of estimate** | **Sources** |
| **Eastern Europe** |  |  |  |  |  |  |  |  |  |  |  |
| Armenia | 64 | 7 | 2024 | ·· | ·· | ·· |  | ·· | ·· | ·· |  |
| Azerbaijan | 716 | 19 | 2023 | 0·0 (0·0, 2·9) | < 500 (<500,<500) | 2014 | 1 | ·· | ·· | ·· |  |
| Belarus | 3516 | 106 | 2018 | ·· | ·· | ·· |  | ·· | ·· | ·· |  |
| Bosnia & Herzegovina | 64 | 5 | 2024/2023 | ·· | ·· | ·· |  | ·· | ·· | ·· |  |
| Bulgaria | 230 | 11 | 2024 | ·· | ·· | ·· |  | ·· | ·· | ·· |  |
| Czechia | 1709 | 52 | 2024 | ·· | ·· | ·· |  | ·· | ·· | ·· |  |
| Estonia | 76 | 19 | 2024 | ·· | ·· | ·· |  | ·· | ·· | ·· |  |
| Georgia | 429 | 29 | 2024 | ·· | ·· | ·· |  | ·· | ·· | ·· |  |
| Hungary | 1535 | 49 | 2023 | 1·4 (0·2, 3·4) | < 500 (<500,<500) | 2010 | 2 | ·· | ·· | ·· |  |
| Latvia | 252 | 46 | 2024 | ·· | ·· | ·· |  | ·· | ·· | ·· |  |
| Lithuania | 205 | 23 | 2024 | ·· | ·· | ·· |  | ·· | ·· | ·· |  |
| Republic of Moldova | 302 | 29 | 2024 | ·· | ·· | ·· |  | ·· | ·· | ·· |  |
| Poland | 3727 | 28 | 2024 | ·· | ·· | ·· |  | 0·0 (0·0, 3·2) | < 500 (<500,<500) | 2012 | 3 |
| Romania | 1104 | 18 | 2024 | ·· | ·· | ·· |  | ·· | ·· | ·· |  |
| Russian Federation | 38538 | 77 | 2023 | ·· | ·· | ·· |  | ·· | ·· | ·· |  |
| Slovakia | 627 | 37 | 2024 | ·· | ·· | ·· |  | ·· | ·· | ·· |  |
| Ukraine | 2333 | 11 | 2024 | ·· | ·· | ·· |  | ·· | ·· | ·· |  |
| **Western Europe** |  |  |  |  |  |  |  |  |  |  |  |
| Albania | 60 | 6 | 2024 | ·· | ·· | ·· |  | ·· | ·· | ·· |  |
| Andorra | 6 | 22 | 2024 | ·· | ·· | ·· |  | ·· | ·· | ·· |  |
| Austria | 632 | 21 | 2024 | ·· | ·· | ·· |  | ·· | ·· | ·· |  |
| Belgium | 566 | 15 | 2024 | ·· | ·· | ·· |  | ·· | ·· | ·· |  |
| Croatia | 227 | 20 | 2023 | 0·0 (0·0, 0·9) | < 500 (<500,<500) | 2008 | 4 | ·· | ·· | ·· |  |
| Denmark | 216 | 11 | 2024 | ·· | ·· | ·· |  | ·· | ·· | ·· |  |
| England and Wales | 3521 | 18 | 2024 | ·· | ·· | ·· |  | ·· | ·· | ·· |  |
| Finland | 224 | 12 | 2023 | 0·0 (0·0, 1·7) | < 500 (<500,<500) | 2022 | 5 | ·· | ·· | ·· |  |
| France | 2707 | 18 | 2024 | ·· | ·· | ·· |  | ·· | ·· | ·· |  |
| Germany | 3419 | 12 | 2023 | ·· | ·· | ·· |  | ·· | ·· | ·· |  |
| Greece | 512 | 15 | 2024 | ·· | ·· | ·· |  | ·· | ·· | ·· |  |
| Greenland | 21 | 113 | 2023 | ·· | ·· | ·· |  | ·· | ·· | ·· |  |
| Iceland | 14 | 10 | 2024 | ·· | ·· | ·· |  | ·· | ·· | ·· |  |
| Ireland | 269 | 14 | 2024 | ·· | ·· | ·· |  | ·· | ·· | ·· |  |
| Italy | 2733 | 14 | 2024 | 1·2 (0·5, 2·2) | < 500 (<500,<500) | 2019-22 | 6-8 | ·· | ·· | ·· |  |
| Liechtenstein | 0 | 0 | 2024 | ·· | ·· | ·· |  | ·· | ·· | ·· |  |
| Luxembourg | 33 | 15 | 2024 | ·· | ·· | ·· |  | ·· | ·· | ·· |  |
| Malta | 61 | 35 | 2024 | ·· | ·· | ·· |  | ·· | ·· | ·· |  |
| Monaco | 0 | 42 | 2024 | ·· | ·· | ·· |  | ·· | ·· | ·· |  |
| Montenegro | 37 | 16 | 2024 | ·· | ·· | ·· |  | ·· | ·· | ·· |  |
| Netherlands | 542 | 9 | 2023 | ·· | ·· | ·· |  | ·· | ·· | ·· |  |
| North Macedonia | 95 | 11 | 2024 | ·· | ·· | ·· |  | ·· | ·· | ·· |  |
| Northern Ireland | 92 | 14 | 2024 | ·· | ·· | ·· |  | ·· | ·· | ·· |  |
| Norway | 140 | 9 | 2024 | ·· | ·· | ·· |  | ·· | ·· | ·· |  |
| Portugal | 891 | 26 | 2024 | 25·4 (17·7, 37·7) | < 500 (<500,500) | 2002 | 9 | ·· | ·· | ·· |  |
| San Marino | 0 | 0 | 2024 | ·· | ·· | ·· |  | ·· | ·· | ·· |  |
| Scotland | 338 | 17 | 2024 | ·· | ·· | ·· |  | ·· | ·· | ·· |  |
| Serbia | 453 | 15 | 2023 | ·· | ·· | ·· |  | ·· | ·· | ·· |  |
| Slovenia | 99 | 15 | 2024 | ·· | ·· | ·· |  | ·· | ·· | ·· |  |
| Spain | 4026 | 26 | 2023 | ·· | ·· | ·· |  | 2·3 (1·4, 3·3) | < 500 (<500,<500) | 1999 | 10 |
| Sweden | 641 | 19 | 2024 | 0·0 (0·0, 6·3) | < 500 (<500,<500) | 2017 | 11 | ·· | ·· | ·· |  |
| Switzerland | 392 | 14 | 2024 | 0·2 (0·1, 0·2) | < 500 (<500,<500) | 2007 | 12 | ·· | ·· | ·· |  |
| **East and South East Asia** |  |  |  |  |  |  |  |  |  |  |  |
| Brunei Darussalam | 76 | 50 | 2022 | ·· | ·· | ·· |  | ·· | ·· | ·· |  |
| Cambodia | 2978 | 55 | 2024 | ·· | ·· | ·· |  | ·· | ·· | ·· |  |
| China | 145340 | 30 | 2018 | ·· | ·· | ·· |  | 0·9 (0·6, 1·4) | 1500 (1000,2000) | 2017 | 13 |
| Hong Kong† | 1907 | 68 | 2023 | ·· | ·· | ·· |  | ·· | ·· | ·· |  |
| Indonesia | 13155 | 14 | 2024 | 5·4 (3·1, 8·4) | 500 (500,1000) | 2017 | 14 | ·· | ·· | ·· |  |
| Japan | 3598 | 10 | 2023 | ·· | ·· | ·· |  | ·· | ·· | ·· |  |
| Lao People's Democratic Republic | 1628 | 68 | 2018 | ·· | ·· | ·· |  | ·· | ·· | ·· |  |
| Malaysia | 5158 | 45 | 2024 | ·· | ·· | ·· |  | ·· | ·· | ·· |  |
| Mongolia | 257 | 24 | 2023 | ·· | ·· | ·· |  | ·· | ·· | ·· |  |
| Myanmar | 12340 | 67 | 2020 | ·· | ·· | ·· |  | ·· | ·· | ·· |  |
| Democratic People's Republic of Korea* | 8889 | 66 | 2016 | ·· | ·· | ·· |  | ·· | ·· | ·· |  |
| Philippines | 16782 | 47 | 2024 | 8·9 (5·3, 13·3) | 1500 (1000,2000) | 1997-2014 | 15,16 | ·· | ·· | ·· |  |
| Singapore | 973 | 47 | 2022 | ·· | ·· | ·· |  | ·· | ·· | ·· |  |
| Republic of Korea | 4447 | 24 | 2022 | ·· | ·· | ·· |  | ·· | ·· | ·· |  |
| Taiwan† | 5653 | 62 | 2024 | ·· | ·· | ·· |  | ·· | ·· | ·· |  |
| Thailand | 33188 | 130 | 2023 | ·· | ·· | ·· |  | 0·2 (0·1, 0·4) | < 500 (<500,<500) | 2005-17 | 17,18 |
| Timor-Leste | 31 | 8 | 2021 | ·· | ·· | ·· |  | ·· | ·· | ·· |  |
| Viet Nam | 16212 | 48 | 2022 | ·· | ·· | ·· |  | ·· | ·· | ·· |  |
| **South Asia** |  |  |  |  |  |  |  |  |  |  |  |
| Afghanistan | 798 | 7 | 2024 | ·· | ·· | ·· |  | ·· | ·· | ·· |  |
| Bangladesh | 2099 | 4 | 2024 | ·· | ·· | ·· |  | 0·2 (0·2, 0·2) | < 500 (<500,<500) | 2008 | 19 |
| Bhutan | 48 | 19 | 2014 | ·· | ·· | ·· |  | ·· | ·· | ·· |  |
| India | 23502 | 5 | 2022 | 1·0 (0·2, 2·4) | < 500 (<500,500) | 2010-15 | 20,21 | 0·8 (0·7, 0·9) | < 500 (<500,<500) | 2013-17 | 22,23 |
| Iran (Islamic Republic of) | 5859 | 20 | 2020 | 2·4 (1·4, 3·8) | < 500 (<500,<500) | 2008-18 | 24-28 | ·· | ·· | ·· |  |
| Maldives | 73 | 47 | 2020 | ·· | ·· | ·· |  | ·· | ·· | ·· |  |
| Nepal | 1488 | 14 | 2022 | ·· | ·· | ·· |  | ·· | ·· | ·· |  |
| Pakistan | 1738 | 3 | 2024 | ·· | ·· | ·· |  | ·· | ·· | ·· |  |
| Sri Lanka | 1247 | 17 | 2024 | 0·0 (0·0, 2·5) | < 500 (<500,<500) | 2013 | 29 | ·· | ·· | ·· |  |
| **Central Asia** |  |  |  |  |  |  |  |  |  |  |  |
| Kazakhstan | 2466 | 40 | 2022 | ·· | ·· | ·· |  | ·· | ·· | ·· |  |
| Kyrgyzstan | 224 | 11 | 2023 | 6·2 (1·8, 12·6) | < 500 (<500,<500) | 2014 | 30 | ·· | ·· | ·· |  |
| Tajikistan | 336 | 11 | 2022 | ·· | ·· | ·· |  | ·· | ·· | ·· |  |
| Turkmenistan | 2275 | 111 | 2021 | ·· | ·· | ·· |  | ·· | ·· | ·· |  |
| Uzbekistan | 1672 | 9 | 2022 | ·· | ·· | ·· |  | ·· | ·· | ·· |  |
| **Caribbean** |  |  |  |  |  |  |  |  |  |  |  |
| Antigua & Barbuda | 22 | 64 | 2023 | ·· | ·· | ·· |  | ·· | ·· | ·· |  |
| Bahamas | 141 | 39 | 2021 | ·· | ·· | ·· |  | ·· | ·· | ·· |  |
| Barbados | 21 | 25 | 2023 | ·· | ·· | ·· |  | ·· | ·· | ·· |  |
| Bermuda | 4 | 19 | 2021 | ·· | ·· | ·· |  | ·· | ·· | ·· |  |
| Cuba | 2848 | 50 | 2020 | ·· | ·· | ·· |  | ·· | ·· | ·· |  |
| Dominica | 3 | 12 | 2024 | ·· | ·· | ·· |  | ·· | ·· | ·· |  |
| Dominican Republic | 676 | 18 | 2024 | ·· | ·· | ·· |  | ·· | ·· | ·· |  |
| Grenada | 6 | 12 | 2023 | ·· | ·· | ·· |  | ·· | ·· | ·· |  |
| Haiti | 263 | 7 | 2024 | ·· | ·· | ·· |  | ·· | ·· | ·· |  |
| Jamaica | 142 | 14 | 2022 | ·· | ·· | ·· |  | ·· | ·· | ·· |  |
| Commonwealth of Puerto Rico | 215 | 20 | 2022 | ·· | ·· | ·· |  | ·· | ·· | ·· |  |
| Saint Kitts & Nevis | 6 | 35 | 2022 | ·· | ·· | ·· |  | ·· | ·· | ·· |  |
| Saint Lucia | 15 | 23 | 2023 | ·· | ·· | ·· |  | ·· | ·· | ·· |  |
| Saint Vincent & the Grenadines | 8 | 32 | 2024 | ·· | ·· | ·· |  | ·· | ·· | ·· |  |
| Trinidad & Tobago | 110 | 21 | 2021 | ·· | ·· | ·· |  | ·· | ·· | ·· |  |
| **Latin America** |  |  |  |  |  |  |  |  |  |  |  |
| Argentina | 5127 | 35 | 2023 | ·· | ·· | ·· |  | ·· | ·· | ·· |  |
| Belize | 48 | 30 | 2024 | ·· | ·· | ·· |  | ·· | ·· | ·· |  |
| Bolivia (Plurinational State of) | 2053 | 49 | 2024 | 0·5 (0·0, 1·4) | < 500 (<500,<500) | 2013 | 31,32 | ·· | ·· | ·· |  |
| Brazil | 50661 | 63 | 2024 | 0·8 (0·2, 1·8) | 500 (<500,1000) | 1997-2018 | 33-38 | 0·1 (0·1, 0·1) | < 500 (<500,<500) | 2004-20 | 39,40 |
| Chile | 4900 | 70 | 2024 | ·· | ·· | ·· |  | ·· | ·· | ·· |  |
| Colombia | 6365 | 37 | 2024 | ·· | ·· | ·· |  | ·· | ·· | ·· |  |
| Costa Rica | 784 | 48 | 2022 | ·· | ·· | ·· |  | ·· | ·· | ·· |  |
| Ecuador | 2087 | 31 | 2024 | ·· | ·· | ·· |  | ·· | ·· | ·· |  |
| El Salvador | 8104 | 367 | 2024 | ·· | ·· | ·· |  | ·· | ·· | ·· |  |
| Guatemala | 2827 | 51 | 2023 | ·· | ·· | ·· |  | ·· | ·· | ·· |  |
| Guyana | 60 | 24 | 2024 | ·· | ·· | ·· |  | ·· | ·· | ·· |  |
| Honduras | 1188 | 32 | 2023 | ·· | ·· | ·· |  | ·· | ·· | ·· |  |
| Mexico | 13836 | 31 | 2024 | 0·1 (0·0, 0·3) | < 500 (<500,<500) | 2009-10 | 41,42 | ·· | ·· | ·· |  |
| Nicaragua | 1130 | 50 | 2018 | ·· | ·· | ·· |  | ·· | ·· | ·· |  |
| Panama | 1166 | 86 | 2024 | ·· | ·· | ·· |  | ·· | ·· | ·· |  |
| Paraguay | 213 | 43 | 2023 | ·· | ·· | ·· |  | ·· | ·· | ·· |  |
| Peru | 5075 | 46 | 2024 | ·· | ·· | ·· |  | 0·6 (0·3, 1·0) | < 500 (<500,<500) | 2021 | 43ZW01 |
| Suriname | 31 | 14 | 2014 | ·· | ·· | ·· |  | ·· | ·· | ·· |  |
| Uruguay | 1246 | 104 | 2024 | ·· | ·· | ·· |  | ·· | ·· | ·· |  |
| Venezuela (Bolivarian Republic of) | 5242 | 58 | 2022 | ·· | ·· | ·· |  | ·· | ·· | ·· |  |
| **North America** |  |  |  |  |  |  |  |  |  |  |  |
| Canada | 2449 | 20 | 2023 | ·· | ·· | ·· |  | ·· | ·· | ·· |  |
| United States of America | 159113 | 146 | 2022 | 0·6 (0·1, 1·9) | 1000 (<500,3000) | 2000 | 44 | 0·0 (0·0, 0·0) | < 500 (<500,<500) | 1994-1998 | 45 |
| **Pacific Island States & Terr·** |  |  |  |  |  |  |  |  |  |  |  |
| American Samoa | 29 | 193 | 2022 | ·· | ·· | ·· |  | ·· | ·· | ·· |  |
| Micronesia (Federated States of) | 0 | 0 | 2014 | ·· | ·· | ·· |  | ·· | ·· | ·· |  |
| Fiji | 71 | 19 | 2024 | ·· | ·· | ·· |  | ·· | ·· | ·· |  |
| French Polynesia | 21 | 23 | 2024 | ·· | ·· | ·· |  | ·· | ·· | ·· |  |
| Guam | 35 | 67 | 2024 | ·· | ·· | ·· |  | ·· | ·· | ·· |  |
| Kiribati | 1 | 2 | 2016 | ·· | ·· | ·· |  | ·· | ·· | ·· |  |
| Marshall Islands | 0 | 0 | 2014 | ·· | ·· | ·· |  | ·· | ·· | ·· |  |
| Nauru | 2 | 55 | 2023 | ·· | ·· | ·· |  | ·· | ·· | ·· |  |
| New Caledonia | 9 | 9 | 2024 | ·· | ·· | ·· |  | ·· | ·· | ·· |  |
| Northern Mariana Islands | 10 | 64 | 2022 | ·· | ·· | ·· |  | ·· | ·· | ·· |  |
| Palau | 3 | 51 | 2023 | ·· | ·· | ·· |  | ·· | ·· | ·· |  |
| Papua New Guinea | 236 | 8 | 2023 | ·· | ·· | ·· |  | ·· | ·· | ·· |  |
| Samoa | 19 | 31 | 2019 | ·· | ·· | ·· |  | ·· | ·· | ·· |  |
| Solomon Islands | 9 | 5 | 2019 | ·· | ·· | ·· |  | ·· | ·· | ·· |  |
| Tonga | 21 | 65 | 2022 | ·· | ·· | ·· |  | ·· | ·· | ·· |  |
| Tuvalu | 0 | 0 | 2014 | ·· | ·· | ·· |  | ·· | ·· | ·· |  |
| Vanuatu | 13 | 14 | 2021 | ·· | ·· | ·· |  | ·· | ·· | ·· |  |
| **Australasia** |  |  |  |  |  |  |  |  |  |  |  |
| Australia‡§ | 3480 | 40 | 2024 | 0·4 (0·2, 2·5) | < 500 (<500,<500) | 2023 | 46 | ·· | ·· | ·· |  |
| New Zealand | 675 | 37 | 2024 | ·· | ·· | ·· |  | ·· | ·· | ·· |  |
| **Sub Saharan Africa** |  |  |  |  |  |  |  |  |  |  |  |
| Angola | 602 | 7 | 2024 | ·· | ·· | ·· |  | ·· | ·· | ·· |  |
| Benin | 606 | 18 | 2024 | ·· | ·· | ·· |  | ·· | ·· | ·· |  |
| Botswana | 199 | 24 | 2022 | ·· | ·· | ·· |  | ·· | ·· | ·· |  |
| Burkina Faso | 158 | 3 | 2022 | 6·7 (0·0, 26·4) | < 500 (<500,<500) | 2009 | 47 | ·· | ·· | ·· |  |
| Burundi | 1037 | 32 | 2024 | ·· | ·· | ·· |  | ·· | ·· | ·· |  |
| Cameroon | 895 | 12 | 2024 | ·· | ·· | ·· |  | ·· | ·· | ·· |  |
| Cabo Verde | 78 | 40 | 2024 | ·· | ·· | ·· |  | ·· | ·· | ·· |  |
| Central African Republic | 104 | 5 | 2023 | ·· | ·· | ·· |  | ·· | ·· | ·· |  |
| Chad | 144 | 5 | 2022 | ·· | ·· | ·· |  | ·· | ·· | ·· |  |
| Comoros | 7 | 3 | 2023 | ·· | ·· | ·· |  | ·· | ·· | ·· |  |
| Côte d'Ivoire | 679 | 9 | 2024 | ·· | ·· | ·· |  | 47·6 (37·0, 59·8) | 500 (500,500) | 2015 | 48 |
| Democratic Republic of the Congo | 1024 | 4 | 2022 | ·· | ·· | ·· |  | 9·9 (3·4, 18·6) | < 500 (<500,<500) | 2015 | 49 |
| Djibouti | 38 | 11 | 2022 | ·· | ·· | ·· |  | ·· | ·· | ·· |  |
| Equatorial Guinea | 25 | 6 | 2015 | ·· | ·· | ·· |  | ·· | ·· | ·· |  |
| Eritrea** | ·· | ·· | N/A | 1·3 (0·0, 5·5) | < 500 (<500,<500) | 2014 | 50 | ·· | ·· | ·· |  |
| Eswatini | 99 | 0 | 2022 | ·· | ·· | ·· |  | ·· | ·· | ·· |  |
| Ethiopia | 5390 | 14 | 2020 | 2·8 (0·1, 7·7) | < 500 (<500,500) | 2016-22 | 51-54 | 0·3 (0·1, 0·5) | < 500 (<500,<500) | 2008-17 | 55-60 |
| Gabon | 193 | 18 | 2024 | ·· | ·· | ·· |  | ·· | ·· | ·· |  |
| Gambia | 14 | 2 | 2021 | ·· | ·· | ·· |  | ·· | ·· | ·· |  |
| Ghana | 214 | 2 | 2024 | 17·4 (12·1, 23·4) | < 500 (<500,<500) | 2006-13 | 61-63 | 0·0 (0·0, 8·9) | < 500 (<500,<500) | 2014 | 64 |
| Guinea | 166 | 4 | 2024 | ·· | ·· | ·· |  | ·· | ·· | ·· |  |
| Guinea-Bissau | 15 | 3 | 2017 | ·· | ·· | ·· |  | ·· | ·· | ·· |  |
| Kenya | 2820 | 19 | 2023 | ·· | ·· | ·· |  | ·· | ·· | ·· |  |
| Lesotho | 64 | 9 | 2019 | ·· | ·· | ·· |  | ·· | ·· | ·· |  |
| Liberia | 84 | 6 | 2023 | 16·7 (4·0, 34·6) | < 500 (<500,<500) | 2021 | 65 | ·· | ·· | ·· |  |
| Madagascar | 1679 | 20 | 2023 | ·· | ·· | ·· |  | ·· | ·· | ·· |  |
| Malawi | 182 | 3 | 2024 | 0·0 (0·0, 7·7) | < 500 (<500,<500) | 2005 | 66 | ·· | ·· | ·· |  |
| Mali | 260 | 5 | 2022 | ·· | ·· | ·· |  | ·· | ·· | ·· |  |
| Mauritania | 57 | 4 | 2022 | ·· | ·· | ·· |  | ·· | ·· | ·· |  |
| Mauritius | 174 | 38 | 2024 | ·· | ·· | ·· |  | ·· | ·· | ·· |  |
| Mozambique | 660 | 7 | 2024 | ·· | ·· | ·· |  | ·· | ·· | ·· |  |
| Namibia | 258 | 33 | 2021/2022 | ·· | ·· | ·· |  | ·· | ·· | ·· |  |
| Niger | 403 | 7 | 2023 | ·· | ·· | ·· |  | ·· | ·· | ·· |  |
| Nigeria | 1848 | 3 | 2024 | ·· | ·· | ·· |  | ·· | ·· | ·· |  |
| Congo | 51 | 3 | 2019 | ·· | ·· | ·· |  | ·· | ·· | ·· |  |
| Rwanda | 5783 | 117 | 2024 | 3·5 (2·8, 4·1) | < 500 (<500,<500) | 2017 | 67 | ·· | ·· | ·· |  |
| Sao Tome & Principe | 12 | 19 | 2023 | ·· | ·· | ·· |  | ·· | ·· | ·· |  |
| Senegal | 343 | 7 | 2023 | ·· | ·· | ·· |  | ·· | ·· | ·· |  |
| Seychelles | 29 | 104 | 2024 | ·· | ·· | ·· |  | ·· | ·· | ·· |  |
| Sierra Leone | 156 | 3 | 2024 | ·· | ·· | ·· |  | ·· | ·· | ·· |  |
| Somalia | 104 | 0 | 2023 | ·· | ·· | ·· |  | ·· | ·· | ·· |  |
| South Africa | 4712 | 24 | 2023 | ·· | ·· | ·· |  | 0·0 (0·0, 0·0) | < 500 (<500,<500) | 2017 | 68 |
| United Republic of Tanzania | 1111 | 6 | 2022 | ·· | ·· | ·· |  | 6·5 (2·6, 12·7) | < 500 (<500,<500) | 2011 | 69 |
| Togo | 130 | 5 | 2021 | ·· | ·· | ·· |  | ·· | ·· | ·· |  |
| Uganda | 3770 | 30 | 2024 | ·· | ·· | ·· |  | 0·1 (0·0, 0·2) | < 500 (<500,<500) | 2012 | 70 |
| Zambia | 931 | 14 | 2024 | ·· | ·· | ·· |  | 1·3 (1·0, 1·7) | < 500 (<500,<500) | 2019 | 71 |
| Zimbabwe | 651 | 9 | 2024 | ·· | ·· | ·· |  | ·· | ·· | ·· |  |
| **Middle East & North Africa** |  |  |  |  |  |  |  |  |  |  |  |
| Algeria | 1421 | 10 | 2021 | ·· | ·· | ·· |  | ·· | ·· | ·· |  |
| Bahrain | 164 | 43 | 2017 | ·· | ·· | ·· |  | ·· | ·· | ·· |  |
| Cyprus | 89 | 22 | 2024 | ·· | ·· | ·· |  | ·· | ·· | ·· |  |
| Egypt | 4440 | 13 | 2022 | ·· | ·· | ·· |  | ·· | ·· | ·· |  |
| Iraq | 1917 | 15 | 2021 | ·· | ·· | ·· |  | ·· | ·· | ·· |  |
| Israel | 198 | 7 | 2023 | ·· | ·· | ·· |  | ·· | ·· | ·· |  |
| Jordan | 421 | 13 | 2022 | ·· | ·· | ·· |  | ·· | ·· | ·· |  |
| Kuwait | 307 | 26 | 2024 | ·· | ·· | ·· |  | ·· | ·· | ·· |  |
| Lebanon | 268 | 15 | 2023 | ·· | ·· | ·· |  | ·· | ·· | ·· |  |
| Libya | 210 | 10 | 2023 | ·· | ·· | ·· |  | ·· | ·· | ·· |  |
| Morocco | 2566 | 20 | 2023 | ·· | ·· | ·· |  | ·· | ·· | ·· |  |
| Oman | 74 | 8 | 2015 | ·· | ·· | ·· |  | ·· | ·· | ·· |  |
| Occupied Palestinian territories | ·· | ·· | N/A | ·· | ·· | ·· |  | ·· | ·· | ·· |  |
| Qatar | 60 | 19 | 2022 | ·· | ·· | ·· |  | ·· | ·· | ·· |  |
| Saudi Arabia | 1293 | 13 | 2017 | ·· | ·· | ·· |  | ·· | ·· | ·· |  |
| South Sudan | 916 | 32 | 2021 | ·· | ·· | ·· |  | ·· | ·· | ·· |  |
| Sudan | 357 | 3 | 2017 | ·· | ·· | ·· |  | ·· | ·· | ·· |  |
| Syrian Arab Republic | 784 | 12 | 2004 | ·· | ·· | ·· |  | ·· | ·· | ·· |  |
| Tunisia | 775 | 19 | 2021 | ·· | ·· | ·· |  | ·· | ·· | ·· |  |
| Türkiye | 16350 | 55 | 2024 | 0·0 (0·0, 0·0) | < 500 (<500,<500) | 2008 | 72 | ·· | ·· | ·· |  |
| United Arab Emirates | 1150 | 55 | 2014 | ·· | ·· | ·· |  | ·· | ·· | ·· |  |
| Yemen | 55 | 1 | 2022 | ·· | ·· | ·· |  | ·· | ·· | ·· |  |

**Notes:**

Ns are rounded to the nearest 500.

^1^ Country level data that informed these regional and global incarceration estimates were sourced from the World Prison Brief, collated by the Institute for Crime and Justice Policy Research at Burbeck University. See: https://www.prisonstudies.org/world-prison-brief-data. Note that we used the country estimates to make rates for 15-64 years (not the total country population), so our rates differ from the World Prison Brief estimates.

* Estimates of the prison population total range between 80,000 and 120,000.

† For reporting purposes, these countries or territories are reported separately due to differences in service provision.

‡ The following sources were found for HBV prevalence in women in Australian prisons, but due to the quality of the AusHep^46^ study we did not include them in the meta analysis: ^46,73,74^

** The HBV estimate wasn't included in global and regional estimates as we could not proportionally weight the estimate to be included into global and regional estimates (due to no reporting of total incarcerated population by the World Prison Brief).

·· Indicates that no estimates of the prevalence for that outcome were obtained for that country.

NK Indicates no evidence was located that injecting drug use was occurring in this country.

HBV - Hepatitis B

Please see Appendix 5-8 for details of approach to assessment of study methodology and approach to selection and synthesis of data.

**References for Table 9.4**

1. Azbel L, Wickersham JA, Wegman MP, et al. Burden of substance use disorders, mental illness, and correlates of infectious diseases among soon-to-be released prisoners in Azerbaijan. *Drug and Alcohol Dependence* 2015.

2. Treso B, Barcsay E, Tarjan A, et al. Prevalence and correlates of HCV, HVB, and HIV infection among prison inmates and staff, Hungary. *Journal of Urban Health: bulletin of the New York Academy of Medicine* 2012.

3. Pendzich J, Maksymowicz-Mazur W, Pawlowska J, et al. Tuberculosis among the homeless and inmates kept in custody and in penitentiary institutions in the Silesia region. *Pneumonologia i Alergologia Polska* 2015.

4. Burek V, Horvat J, Butorac K, Mikulić R. Viral hepatitis B, C and HIV infection in Croatian prisons. *Epidemiol Infect* 2010.

5. Rautanen M, Harald, K, & Tyni, S. Health and Wellbeing of Prisoners 2023 The Wattu IV Prison Population Study Finland, 2024.

6. Fiore V, De Vito A, Rastrelli E, et al. Differences in HCV Seroprevalence, Clinical Features, and Treatment Outcomes between Female and Male Incarcerated Population: Results from a Matched Cohort Study. *Viruses* 2023; **15**(12).

7. Geremia N, Giovagnorio F, De Vito A, et al. HBV in Italian Women's Jail: An Underestimated Problem? *Journal of Clinical Medicine* 2024; **13(5) (no pagination)**.

8. Scelza G, Amato A, Pagano AM, et al. Effect of hepatitis C antiviral therapy on oral lichen planus and hyposalivation in inmates. *Annals of Gastroenterology* 2022.

9. Garcia A, Exposto F, Prieto E, Lopes M, Duarte A, da Silva RC. Association of Trichomonas vaginalis with sociodemographic factors and other STDs among females inmates in Lisbon. *International Journal of STD & AIDS* 2004.

10. Sanchez VM, Guerra JM, Cayla JA, Rodriguez JC, Blanco MD, Alcoba M. Incidence of tuberculosis and the importance of treatment of latent tuberculosis infection in a Spanish prison population. *International Journal of Tuberculosis and Lung Disease* 2001.

11. Gahrton C, Westman G, Lindahl K, et al. Prevalence of Viremic hepatitis C, hepatitis B, and HIV infection, and vaccination status among prisoners in Stockholm County. *BMC Infectious Diseases* 2019.

12. Wolff H, Sebo P, Haller DM, et al. Health problems among detainees in Switzerland: a study using the ICPC-2 classification. *BMC Public Health* 2011.

13. Tony Y, Jiang S, Guan X, et al. Epidemic situation of tuberculosis in prisons in the central region of China. *American Journal of Tropical Medicine and Hygiene* 2019.

14. Arends RM, Nelwan EJ, Soediro R, et al. Associations between impulsivity, risk behavior and HIV, HBV, HCV and syphilis seroprevalence among female prisoners in Indonesia: A cross-sectional study. *PLoS One* 2019.

15. Ong-Chu MC, Lao-Tan JY, Gabriel EA. Prevalence of hepatitis B and C and risk factors among prison inmates in Cebu, Philippines. *Hepatology International* 2016.

16. Simbulan NP, Aguilar AS, Flanigan T, Cu-Uvin S. High-risk behaviors and the prevalence of sexually transmitted diseases among women prisoners at the women state penitentiary in Metro Manila. *Social Science & Medicine* 2001.

17. Jittimanee SX, Ngamtrairai N, White MC, Jittimanee S. A prevalence survey for smear-positive tuberculosis in Thai prisons. The *International Journal of Tuberculosis and Lung Disease* 2007.

18. Jittimanee S, Namonta A, Charuenporn C. Systematic TB screening using WHO radiograph categorisation and care outcomes. *The International Journal of Tuberculosis and Lung Disease: the official journal of the International Union against Tuberculosis and Lung Disease* 2022.

19. Banu S, Hossain A, Uddin MKM, et al. Pulmonary tuberculosis and drug resistance in Dhaka central jail, the largest prison in Bangladesh. *PLoS One* 2010.

20. Ramamoorthy M, Venketeswaran A, Seenivasan P, et al. Risk factors and prevalence, hepatitis B virus and hepatitis C virus among prison inmates, Chennai, India, 2015. 2016; **53**: 90.

21. Rana S, Girdgar N, Gill MK, Kumar AJIJRMS. Prevalence of hepatitis-B surface antigen among population of inmates in Tihar Jail, New Delhi. 2015; **3**: 100-4.

22. Bhatnagar T, Ralte M, Ralte L, Chawnglungmuana, Sundaramoorthy L, Chhakchhuak L. Intensified tuberculosis and HIV surveillance in a prison in Northeast India: Implementation research. *PLoS One* 2019.

23. Dolla CK, Dhanraj B, Malaisamy M, et al. Burden of pulmonary tuberculosis in modern prison: A cross sectional prevalence survey from south India. *Indian Journal of Tuberculosis* 2019.

24. Khajedaluee M, Babaei A, Vakili R, et al. Sero-prevalence of bloodborne tumor viruses (HCV, HBV, HTLV-I and KSHV infections) and related risk factors among prisoners in Razavi Khorasan province, Iran, in 2008. *Hepatitis Monthly* 2016.

25. Moradi G, Gouya MM, Zavareh FA, et al. Prevalence and risk factors for HBV and HCV in prisoners in Iran: a national bio-behavioural surveillance survey in 2015. *Tropical Medicine & International Health* 2018.

26. Moradi G, Jafari S, Zarei B, et al. Prevalence and Risk Factors for Hepatitis B and Hepatitis C Exposure in Iranian Prisoners: A National Study in 2016. *Hepatitis Monthly* 2019.

27. Nokhodian Z, Yazdani MR, Yaran M, et al. Prevalence and risk factors of HIV, syphilis, hepatitis B and C among female prisoners in Isfahan, Iran. *Hepatitis Monthly* 2012.

28. Ziaee M, Sharifzadeh G, Namaee MH, Fereidouni M. Prevalence of HIV and Hepatitis B, C, D Infections and Their Associated Risk Factors among Prisoners in Southern Khorasan Province, Iran. *Iranian Journal of Public Health* 2014.

29. Niriella MA, Hapangama A, Luke H, Pathmeswaran A, Kuruppuarachchi K, de Silva HJ. Prevalence of hepatitis B and hepatitis C infections and their relationship to injectable drug use in a cohort of Sri Lankan prison inmates. *Ceylon Medical Journal* 2015.

30. Azbel L, Polonsky M, Wegman M, et al. Intersecting epidemics of HIV, HCV, and syphilis among soon-to-be released prisoners in Kyrgyzstan: Implications for prevention and treatment. *International Journal of Drug Policy* 2016.

31. Villarroel-Torrico M, Montano K, Flores-Arispe P, et al. Syphilis, human immunodeficiency virus, herpes genital and hepatitis B in a women's prison in Cochabamba, Bolivia: prevalence and risk factors. *Revista Espanola de Sanidad Penitenciaria* 2018.

32. Villarroel-Torrico M, Montaño K, Flores-Arispe P, et al. Syphilis, human immunodeficiency virus, herpes genital and hepatitis B in a women's prison in Cochabamba, Bolivia: prevalence and risk factors. *Revista Espanola de Sanidad Penitenciaria* 2018.

33. Barros LAS, Pessoni GC, Teles SA, et al. Epidemiology of the viral hepatitis B and C in female prisoners of Metropolitan Regional Prison Complex in the State of Goias, Central Brazil. *Revista da Sociedade Brasileira de Medicina Tropical* 2013.

34. Benedetti MSG, Nogami ASA, da Costa BB, et al. Sexually transmitted infections in women deprived of liberty in Roraima, Brazil. *Revista de Saude Publica* 2020.

35. Miranda AE, Vargas, P.M., Louis, M.E.S. & Viana, M.C. Sexually transmitted diseases among female prisoners in Brazil: prevalence and risk factors. *Sexually Transmitted Diseases* 2000; **27**(9): 491-5.

36. Rezende GR, Lago BV, Puga MA, et al. Prevalence, incidence and associated factors for HBV infection among male and female prisoners in Central Brazil: A multicenter study. *International Journal of Infectious Diseases* 2020.

37. Stief ACF, Martins RMB, de Andrade SMO, et al. Seroprevalence of hepatitis b virus infection and associated factors among prison inmates in state of mato grosso do sul, Brazil. *Revista da Sociedade Brasileira de Medicina Tropical* 2011.

38. De Sena Silva AA, De Araújo TME, Teles SA, De Lima Brito Magalhães R, Andrade ELR. Prevalence of Hepatitis B and associated factors in prisoners. *Acta Paulista de Enfermagem* 2017; **30**(1): 66-72.

39. Abrahao RMCM, Nogueira PA, Malucelli MIC. Tuberculosis in county jail prisoners in the western sector of the city of Sao Paulo, Brazil. *International Journal of Tuberculosis and Lung Disease* 2006.

40. Leal M, Kerr L, Mota RMS, Neto RDP, Seal D, Kendall C. Health of female prisoners in Brazil. *Ciencia & Saude Coletiva* 2022.

41. Bautista-Arredondo S, González A, Servan-Mori E, et al. A Cross-Sectional Study of Prisoners in Mexico City Comparing Prevalence of Transmissible Infections and Chronic Diseases with That in the General Population. *PLoS One* 2015.

42. Gonzalez CAM, Ortiz BES, Aguilar MB, Gonzalez JDM. Risk factors and the seroprevalence of viral markers of hepatitis B (HVB) and hepatitis C (HCV) in high-risk groups in Chiapas. *Medwave* 2011.

43. Geadas C, Calderon RI, Yuen CM, et al. Active case-finding for TB among incarcerated women in Peru. *International Journal of Tuberculosis and Lung Disease* 2023; **27**(10): 784 EP-6.

44. Hennessey KA, Kim AA, Griffin V, Collins NT, Weinbaum CM, Sabin K. Prevalence of infection with hepatitis B and C viruses and co-infection with HIV in three jails: a case for viral hepatitis prevention in jails in the United States. *Journal of Urban Health* 2009.

45. Castle White M, Tulsky JP, Portillo CJ, Menendez E, Cruz E, Goldenson J. Tuberculosis prevalence in an urban jail: 1994 and 1998. *International Journal of Tuberculosis and Lung Disease* 2001.

46. Bah R, Sheehan Y, Li X, et al. Prevalence of blood-borne virus infections and uptake of hepatitis C testing and treatment in Australian prisons: the AusHep study. *The Lancet Regional Health–Western Pacific* 2024; **53**.

47. Diendéré EA, Tiéno H, Bognounou R, et al. Prevalence and risk factors associated with infection by human immunodeficiency virus, hepatitis B virus, syphilis and bacillary pulmonary tuberculosis in prisons in Burkina Faso. *Tropical Medicine and Infectious Disease* 2011.

48. Seri B, Koffi A, Danel C, et al. Prevalence of pulmonary tuberculosis among prison inmates: A cross-sectional survey at the Correctional and Detention Facility of Abidjan, Cote d'Ivoire. *PLoS One* 2017.

49. Kalonji GMP, Connick GD, Ngongo LO, et al. Prevalence of tuberculosis and associated risk factors in the central prison of Mbuji-Mayi, DEmocratic republic of Congo. *Tropical Medicine and Health* 2016.

50. Eritrea MoH. Report for HIV, HBsAg, HCV Ab and Syphilis Prevalence and HIV Risk Behavior Survey Among Prison Inmates in Eritrea. In: Division MoHDoPHCDC, editor.; 2015.

51. Kassa Y, Million Y, Biset S, Moges F. Hepatitis b and hepatitis c viral infections and associated factors among prisoners in northeast ethiopia. *Journal of Blood Medicine* 2021.

52. Kebede W, Abdissa A, Seid Y, Mekonnen Z. Seroprevalence and risk factors of hepatitis B, hepatitis C and HIV infections among prisoners in Jimma Town, Southwest Ethiopia. *Asian Pacific Journal of Tropical Disease* 2017.

53. Tadesse K, Ayalew G, Million Y, Gelaw A. Hepatitis B and hepatitis C virus infections and associated factors among prisoners in Gondar City, Northwest Ethiopia. *PLoS One* 2024; **19**(4): e0301973.

54. Tsegay B, Gebrecherkos T, Kahsay AG, Abdulkader M. Seroprevalence and Associated Factors of Hepatitis B and Hepatitis C Viral Infections Among Prisoners in Tigrai, Northern Ethiopia. *Infection and Drug Resistance* 2023; **16**: 3743-50.

55. Adane K, Spigt M, Winkens B, Dinant GJ. Tuberculosis case detection by trained inmate peer educators in a resource-limited prison setting in Ethiopia: a cluster-randomised trial. *The Lancet Global Health* 2019.

56. Addis Z, Adem E, Alemu A, et al. Prevalence of smear positive pulmonary tuberculosis in Gondar prisoners, North West Ethiopia. *Asian Pacific Journal of Tropical Medicine* 2015.

57. Gizachew Beza M, Hunegnaw E, Tiruneh M. Prevalence and associated factors of tuberculosis in prisons settings of East Gojjam Zone, Northwest Ethiopia. *International Journal of Bacteriology* 2017; **2017**.

58. Fuge TG, Ayanto SY. Prevalence of smear positive pulmonary tuberculosis and associated risk factors among prisoners in Hadiya Zone prison, Southern Ethiopia. *BMC Research Notes* 2016.

59. Gebrecherkos T, Gelaw B, Tessema B. Smear positive pulmonary tuberculosis and HIV co-infection in prison settings of North Gondar Zone, Northwest Ethiopia. *BMC Public Health* 2016.

60. Winsa BB, Mohammed AE. Investigation on pulmonary tuberculosis among Bedele Woreda prisoners, Southwest Ethiopia. *International Journal of Biomedical Science and Engineering* 2015; **3**(6): 69-73.

61. Adjei AA, Armah HB, Gbagbo F, et al. Correlates of HIV, HBV, HCV and syphilis infections among prison inmates and officers in Ghana: A national multicenter study. *BMC Infectious Diseases* 2008.

62. Commission GA. National Health and HIV Survey of Prison Inmates in Ghana. 2013.

63. Sagoe KWC, Atuahene K, Ayiku ANA, et al. Hepatitis B and human immunodeficiency virus infections within correctional facilities in Ghana. *PLoS One* 2023; **18**(11): e0293009.

64. Kwabla M, Ameme D, Nortey P. Pulmonary tuberculosis and its risk factors among inmates of a Ghanaian prison. *International Journal of Tropical Disease & Health* 2015; **9**(3): 1-10.

65. Vessellee DB, Yalley AK, Adjei DN, et al. Prevalence of Hepatitis B Virus Infection among Inmates at the Monrovia Central Prison, Liberia. *Tropical Medicine and Infectious Disease* 2023; **8(3) (no pagination)**.

66. Chimphambano C, Komolafe I, Muula A. Prevalence of HIV, HepBsAg and Hep C antibodies among inmates in Chichiri prison, Blantyre, Malawi. *Malawi Medical Journal* 2007; **19**(3): 107-10.

67. Umutesi J, Klett-Tammen C, Nsanzimana S, Krause G, Ott JJ. Cross-sectional study of chronic hepatitis B virus infection in Rwandan high-risk groups: Unexpected findings on prevalence and its determinants. *BMJ Open* 2021.

68. Baird K, Said H, Koornhof HJ, Duse AG. Tuberculosis control at a South African correctional centre: Diagnosis, treatment and strain characterisation. *PLoS One* 2022; **17**(11-Nov): e0277459.

69. Mmbaga VM. Prevalence and factors associated with pulmonary tuberculosis among prisoners in Dar es salaam, Tanzania, 2012: Muhimbili University of Health and Allied Sciences; 2013.

70. Owokuhaisa J, Thokerunga E, Bazira J. Prevalence of pulmonary tuberculosis among prison inmates at Mbarara central prison, South Western Uganda. *Advances in Research* 2014; **2**(11): 618.

71. Kagujje M, Somwe P, Hatwiinda S, et al. Cross-sectional assessment of tuberculosis and HIV prevalence in 13 correctional facilities in Zambia. *BMJ Open* 2021.

72. Balci E, Turker K, Senol V, Gunay O. Screening Indicators of Hepatitis A, Hepatitis B, Hepatitis C and HIV infections in Prisoners. *Viral Hepatitis Journal* 2012.

73. Butler TS, M. National Prison Entrants’ Bloodborne Virus and Risk Behaviour Survey Report: Kirby Institute 2017.

74. Indig D, Topp L, Ross B, et al. 2009 NSW Inmate Health Survey: Key Findings Report. Sydney: Justice Health, 2010.

## *Table 9.5:* Country-level estimates of the number and rate of incarceration, prevalence of lifetime injecting drug use, HIV and HCV among males who are incarcerated

|  | **Males who are incarcerated** | | | **Males with lifetime injecting drug use** | | | | **Males living with HIV** | | | | **Males with current HCV** | | | |
| --- | --- | --- | --- | --- | --- | --- | --- | --- | --- | --- | --- | --- | --- | --- | --- |
| **Country** | **Estimated number^1^** | **Rate per 100,000** | **Year of estimate^1^** | **% (CI)** | **Estimated no. (CI)** | **Year of estimate** | **Sources** | **% (CI)** | **Estimated no. (CI)** | **Year of estimate** | **Sources** | **% (CI)** | **Estimated no. (CI)** | **Year of estimate** | **Sources** |
| **Eastern Europe** |  |  |  |  |  |  |  |  |  |  |  |  |  |  |  |
| Armenia | 2405 | 287 | 2024 | ·· | ·· | ·· |  | 1·3 (0·5, 2·4) | < 500 (<500,<500) | 2004 | 1 | 17·9 (14·8, 21·2) | 500 (500,500) | 2004 | 1 |
| Azerbaijan | 23982 | 687 | 2023 | ·· | ·· | ·· |  | 3·7 (2·5, 5·0) | 1000 (500,1000) | 2008-15 | 2-5 | 33·9 (23·3, 45·5) | 8000 (5500,11000) | 2008-15 | 2-5 |
| Belarus | 29040 | 953 | 2018 | ·· | ·· | ·· |  | ·· | ·· | ·· |  | ·· | ·· | ·· |  |
| Bosnia & Herzegovina | 2148 | 195 | 2024/2023 | ·· | ·· | ·· |  | ·· | ·· | ·· |  | 10·0 (6·2, 14·6) | < 500 (<500,500) | 2013 | 6 |
| Bulgaria | 6148 | 276 | 2024 | ·· | ·· | ·· |  | ·· | ·· | ·· |  | 21·7 (18·6, 25·0) | 1500 (1000,1500) | 2009 | 7 |
| Czechia | 17940 | 526 | 2024 | ·· | ·· | ·· |  | ·· | ·· | ·· |  | ·· | ·· | ·· |  |
| Estonia | 1612 | 380 | 2024 | ·· | ·· | ·· |  | 14·5 (13·3, 15·7) | < 500 (<500,500) | 2012 | 8 | ·· | ·· | ·· |  |
| Georgia | 10028 | 866 | 2024 | ·· | ·· | ·· |  | ·· | ·· | ·· |  | ·· | ·· | ·· |  |
| Hungary | 16735 | 528 | 2023 | 14·3 (12·5, 16·1) | 2500 (2000,2500) | 1998 | 9 | ·· | ·· | ·· |  | ·· | ·· | ·· |  |
| Latvia | 3019 | 516 | 2024 | 30·0 (27·2, 32·8) | 1000 (1000,1000) | 2022 | 10 | ·· | ·· | ·· |  | ·· | ·· | ·· |  |
| Lithuania | 4346 | 486 | 2024 | ·· | ·· | ·· |  | ·· | ·· | ·· |  | ·· | ·· | ·· |  |
| Republic of Moldova | 5393 | 542 | 2024 | ·· | ·· | ·· |  | ·· | ·· | ·· |  | ·· | ·· | ·· |  |
| Poland | 66589 | 529 | 2024 | ·· | ·· | ·· |  | ·· | ·· | ·· |  | ·· | ·· | ·· |  |
| Romania | 23430 | 371 | 2024 | ·· | ·· | ·· |  | ·· | ·· | ·· |  | ·· | ·· | ·· |  |
| Russian Federation | 394468 | 845 | 2023 | ·· | ·· | ·· |  | ·· | ·· | ·· |  | ·· | ·· | ·· |  |
| Slovakia | 7958 | 430 | 2024 | ·· | ·· | ·· |  | ·· | ·· | ·· |  | ·· | ·· | ·· |  |
| Ukraine | 41691 | 298 | 2024 | 44·7 (39·8, 49·6) | 18500 (16500,20500) | 2015 | 11 | 11·5 (9·9, 13·2) | 5000 (4000,5500) | 2011 | 12,13 | ·· | ·· | ·· |  |
| **Western Europe** |  |  |  |  |  |  |  |  |  |  |  |  |  |  |  |
| Albania | 4593 | 477 | 2024 | ·· | ·· | ·· |  | ·· | ·· | ·· |  | ·· | ·· | ·· |  |
| Andorra | 45 | 152 | 2024 | ·· | ·· | ·· |  | ·· | ·· | ·· |  | ·· | ·· | ·· |  |
| Austria | 8656 | 291 | 2024 | 4·2 (0·5, 10·4) | 500 (<500,1000) | 2016 | 14 | 0·0 (0·0, 2·4) | < 500 (<500,<500) | 2016 | 14 | 1·5 (0·0, 6·4) | < 500 (<500,500) | 2016 | 14 |
| Belgium | 12009 | 321 | 2024 | ·· | ·· | ·· |  | ·· | ·· | ·· |  | 8·0 (3·9, 13·3) | 1000 (500,1500) | 2020 | 15 |
| Croatia | 4218 | 318 | 2023 | 24·9 (23·4, 26·4) | 1000 (1000,1000) | 2007-09 | 16,17 | 0·1 (0·0, 0·2) | < 500 (<500,<500) | 2006-09 | 17,18 | 10·5 (9·5, 11·6) | 500 (500,500) | 2007-09 | 16,17 |
| Denmark | 3867 | 207 | 2024 | 43·1 (37·7, 48·5) | 1500 (1500,2000) | 1997 | 19 | 0·0 (0·0, 0·5) | < 500 (<500,<500) | 1997 | 19 | 28·9 (24·1, 34·0) | 1000 (1000,1500) | 1997 | 19 |
| England and Wales | 82346 | 433 | 2024 | 23·3 (21·1, 25·6) | 19000 (17500,21000) | 1998-2018 | 20-22 | 0·2 (0·1, 0·4) | < 500 (<500,500) | 1998 | 22 | 6·4 (3·5, 10·1) | 5500 (3000,8500) | 1998-2021 | 22-29 |
| Finland | 2688 | 156 | 2023 | 53·7 (49·7, 57·7) | 1500 (1500,1500) | 2007-23 | 30,31 | 0·8 (0·2, 1·8) | < 500 (<500,<500) | 2007-23 | 31,32 | 31·7 (28·0, 35·6) | 1000 (1000,1000) | 2007-23 | 31,32 |
| France | 76924 | 390 | 2024 | 28·5 (0·7, 73·9) | 22000 (500,57000) | 1997-2004 | 33-36 | 2·6 (0·8, 5·4) | 2000 (500,4000) | 1997-2019 | 35,37 | 3·5 (1·2, 6·7) | 2500 (1000,5000) | 1997-2019 | 37,38 |
| Germany | 54536 | 202 | 2023 | 29·2 (21·3, 37·7) | 16000 (11500,20500) | 1997 | 35 | 2·5 (0·6, 5·3) | 1500 (500,3000) | 1997-2002 | 35,39 | 5·5 (4·3, 6·9) | 3000 (2500,4000) | 2002 | 39 |
| Greece | 9730 | 297 | 2024 | 36·9 (30·8, 43·4) | 3500 (3000,4000) | 1996-2002 | 40-42 | 0·0 (0·0, 2·1) | < 500 (<500,<500) | 2002 | 40 | 11·2 (8·7, 14·0) | 1000 (1000,1500) | 2018 | 43 |
| Greenland | 133 | 644 | 2023 | NK | NK | ·· |  | ·· | ·· | ·· |  | ·· | ·· | ·· |  |
| Iceland | 126 | 100 | 2024 | ·· | ·· | ·· |  | ·· | ·· | ·· |  | ·· | ·· | ·· |  |
| Ireland | 4805 | 300 | 2024 | 29·4 (24·2, 35·0) | 1500 (1000,1500) | 1998-2017 | 44-46 | 1·0 (0·5, 1·8) | < 500 (<500,<500) | 1998-2017 | 44-47 | 10·4 (6·7, 14·8) | 500 (500,500) | 1998-2017 | 44-46,48 |
| Italy | 59377 | 315 | 2024 | 29·4 (20·5, 39·7) | 17500 (12000,23500) | 1997-2019 | 35,49-51 | 3·0 (1·7, 4·9) | 2000 (1000,3000) | 1997-2019 | 35,49,52,53 | 11·1 (6·9, 16·1) | 6500 (4000,9500) | 2002-22 | 49-58 |
| Liechtenstein | 14 | 108 | 2024 | ·· | ·· | ·· |  | ·· | ·· | ·· |  | ·· | ·· | ·· |  |
| Luxembourg | 567 | 250 | 2024 | ·· | ·· | ·· |  | ·· | ·· | ·· |  | ·· | ·· | ·· |  |
| Malta | 610 | 319 | 2024 | ·· | ·· | ·· |  | ·· | ·· | ·· |  | ·· | ·· | ·· |  |
| Monaco | 31 | 289 | 2024 | ·· | ·· | ·· |  | ·· | ·· | ·· |  | ·· | ·· | ·· |  |
| Montenegro | 1009 | 495 | 2024 | ·· | ·· | ·· |  | 0·0 (0·0, 0·4) | < 500 (<500,<500) | 2012-21 | 59 | ·· | ·· | ·· |  |
| Netherlands | 10995 | 194 | 2023 | 8·6 (5·7, 11·9) | 1000 (500,1500) | 1997-2010 | 35,60 | 0·3 (0·0, 1·5) | < 500 (<500,<500) | 1997-2010 | 35,60 | 4·9 (2·4, 8·2) | 500 (500,1000) | 2010 | 60 |
| North Macedonia | 2460 | 336 | 2024 | ·· | ·· | ·· |  | ·· | ·· | ·· |  | ·· | ·· | ·· |  |
| Northern Ireland | 1819 | 305 | 2024 | 12·0 (9·1, 15·2) | < 500 (<500,500) | 2005 | 61 | ·· | ·· | ·· |  | ·· | ·· | ·· |  |
| Norway | 2912 | 161 | 2024 | 51·5 (39·0, 63·9) | 1500 (1000,2000) | 2014 | 62 | ·· | ·· | ·· |  | ·· | ·· | ·· |  |
| Portugal | 11488 | 361 | 2024 | ·· | ·· | ·· |  | 6·1 (4·6, 7·8) | 500 (500,1000) | 2003-08 | 63,64 | 9·6 (7·7, 12·0) | 1000 (1000,1500) | 2008-18 | 65,66 |
| San Marino | 1 | 9 | 2024 | ·· | ·· | ·· |  | ·· | ·· | ·· |  | ·· | ·· | ·· |  |
| Scotland | 7915 | 461 | 2024 | 26·1 (23·6, 28·6) | 2000 (2000,2500) | 1997-2011 | 35,67,68 | 0·7 (0·0, 2·1) | < 500 (<500,<500) | 1997 | 35 | 3·4 (2·5, 4·3) | 500 (<500,500) | 1998-2000 | 67,69 |
| Serbia | 10334 | 440 | 2023 | ·· | ·· | ·· |  | ·· | ·· | ·· |  | ·· | ·· | ·· |  |
| Slovenia | 1699 | 239 | 2024 | ·· | ·· | ·· |  | ·· | ·· | ·· |  | ·· | ·· | ·· |  |
| Spain | 52672 | 335 | 2023 | 25·3 (19·3, 31·8) | 13500 (10000,16500) | 2013 | 70 | 4·5 (2·1, 8·0) | 2500 (1000,4000) | 2012-20 | 71-73 | 11·9 (7·6, 18·2) | 6500 (4000,9500) | 2008-20 | 71,73,74 |
| Sweden | 9534 | 287 | 2024 | 25·0 (17·4, 33·5) | 2500 (1500,3000) | 1997 | 35 | 0·4 (0·0, 1·2) | < 500 (<500,<500) | 1997-2017 | 35,75 | 11·3 (8·5, 14·4) | 1000 (1000,1500) | 2017 | 75 |
| Switzerland | 6489 | 223 | 2024 | 5·9 (3·3, 9·0) | 500 (<500,500) | 2011-13 | 76,77 | 1·7 (1·2, 2·4) | < 500 (<500,<500) | 2011 | 77,78 | 3·4 (2·7, 4·3) | < 500 (<500,500) | 2011-13 | 76-78 |
| **East and South East Asia** |  |  |  |  |  |  |  |  |  |  |  |  |  |  |  |
| Brunei Darussalam | 560 | 336 | 2022 | ·· | ·· | ·· |  | ·· | ·· | ·· |  | ·· | ·· | ·· |  |
| Cambodia | 42144 | 786 | 2024 | ·· | ·· | ·· |  | ·· | ·· | ·· |  | ·· | ·· | ·· |  |
| China | 1544660 | 304 | 2018 | ·· | ·· | ·· |  | ·· | ·· | ·· |  | ·· | ·· | ·· |  |
| Hong Kong† | 7172 | 313 | 2023 | ·· | ·· | ·· |  | ·· | ·· | ·· |  | ·· | ·· | ·· |  |
| Indonesia | 260905 | 278 | 2024 | 4·3 (0·9, 9·5) | 11000 (2500,24500) | 2010-21 | 79,80 | 0·9 (0·3, 1·8) | 2500 (500,5000) | 2010-21 | 79,80 | ·· | ·· | ·· |  |
| Japan | 37283 | 101 | 2023 | ·· | ·· | ·· |  | ·· | ·· | ·· |  | ·· | ·· | ·· |  |
| Lao People's Democratic Republic | 10257 | 424 | 2018 | ·· | ·· | ·· |  | ·· | ·· | ·· |  | ·· | ·· | ·· |  |
| Malaysia | 82261 | 683 | 2024 | ·· | ·· | ·· |  | ·· | ·· | ·· |  | ·· | ·· | ·· |  |
| Mongolia | 5443 | 520 | 2023 | ·· | ·· | ·· |  | ·· | ·· | ·· |  | ·· | ·· | ·· |  |
| Myanmar | 87984 | 478 | 2020 | ·· | ·· | ·· |  | 5·2 (4·8, 5·7) | 4500 (4000,5000) | 2018 | 81 | ·· | ·· | ·· |  |
| Democratic People's Republic of Korea* | 91111 | 710 | 2016 | NK | NK | ·· |  | ·· | ·· | ·· |  | ·· | ·· | ·· |  |
| Philippines | 154465 | 416 | 2024 | ·· | ·· | ·· |  | ·· | ·· | ·· |  | ·· | ·· | ·· |  |
| Singapore | 8563 | 367 | 2022 | ·· | ·· | ·· |  | ·· | ·· | ·· |  | ·· | ·· | ·· |  |
| Republic of Korea | 48493 | 256 | 2022 | ·· | ·· | ·· |  | ·· | ·· | ·· |  | ·· | ·· | ·· |  |
| Taiwan† | 53236 | 630 | 2024 | 41·8 (40·7, 42·8) | 22000 (21500,23000) | 1994-2008 | 82,83 | 0·0 (0·0, 0·2) | < 500 (<500,<500) | 2019 | 84 | ·· | ·· | ·· |  |
| Thailand | 241089 | 987 | 2023 | ·· | ·· | ·· |  | 2·9 (2·0, 4·0) | 7000 (4500,9500) | 2019 | 85 | ·· | ·· | ·· |  |
| Timor-Leste | 732 | 183 | 2021 | ·· | ·· | ·· |  | ·· | ·· | ·· |  | ·· | ·· | ·· |  |
| Viet Nam | 117774 | 355 | 2022 | ·· | ·· | ·· |  | ·· | ·· | ·· |  | ·· | ·· | ·· |  |
| **South Asia** |  |  |  |  |  |  |  |  |  |  |  |  |  |  |  |
| Afghanistan | 18202 | 167 | 2024 | ·· | ·· | ·· |  | ·· | ·· | ·· |  | ·· | ·· | ·· |  |
| Bangladesh | 51732 | 92 | 2024 | ·· | ·· | ·· |  | ·· | ·· | ·· |  | ·· | ·· | ·· |  |
| Bhutan | 1071 | 361 | 2014 | ·· | ·· | ·· |  | ·· | ·· | ·· |  | ·· | ·· | ·· |  |
| India | 549718 | 112 | 2022 | 3·1 (2·1, 4·3) | 17000 (11500,23500) | 2019-21 | 86-88 | 0·9 (0·6, 1·3) | 5000 (3000,7500) | 2016-21 | 86-89 | 9·2 (8·6, 9·9) | 50500 (47500,54500) | 2015-16 | 90-92 |
| Iran (Islamic Republic of) | 183141 | 597 | 2020 | 15·9 (13·5, 18·5) | 29000 (24500,34000) | 2009-97 | 93-98 | 2·0 (1·2, 3·1) | 3500 (2000,5500) | 2003-17 | 96,99-102 | 8·5 (4·4, 14·1) | 15500 (8000,26000) | 2003-22 | 97,99,100,103-109 |
| Maldives | 1627 | 710 | 2020 | ·· | ·· | ·· |  | ·· | ·· | ·· |  | ·· | ·· | ·· |  |
| Nepal | 26062 | 288 | 2022 | 11·2 (9·1, 13·6) | 3000 (2500,3500) | 2015 | 110,111 | ·· | ·· | ·· |  | ·· | ·· | ·· |  |
| Pakistan | 106905 | 156 | 2024 | 19·7 (5·8, 39·1) | 21000 (6000,42000) | 2008-18 | 112-115 | 2·0 (0·8, 3·6) | 2000 (1000,4000) | 2008-10 | 113,116-118 | 8·2 (5·1, 12·0) | 9000 (5500,13000) | 2008-10 | 112,113,115,117,119 |
| Sri Lanka | 28439 | 410 | 2024 | 4·3 (2·5, 6·6) | 1000 (500,2000) | 2013 | 120 | ·· | ·· | ·· |  | 0·6 (0·0, 1·8) | < 500 (<500,500) | 2013 | 120 |
| **Central Asia** |  |  |  |  |  |  |  |  |  |  |  |  |  |  |  |
| Kazakhstan | 32762 | 567 | 2022 | ·· | ·· | ·· |  | ·· | ·· | ·· |  | ·· | ·· | ·· |  |
| Kyrgyzstan | 7504 | 386 | 2023 | ·· | ·· | ·· |  | ·· | ·· | ·· |  | ·· | ·· | ·· |  |
| Tajikistan | 13664 | 466 | 2022 | 4·2 (3·2, 5·3) | 500 (500,500) | 2010 | 121 | ·· | ·· | ·· |  | ·· | ·· | ·· |  |
| Turkmenistan | 32725 | 1636 | 2021 | ·· | ·· | ·· |  | ·· | ·· | ·· |  | ·· | ·· | ·· |  |
| Uzbekistan | 27328 | 149 | 2022 | ·· | ·· | ·· |  | ·· | ·· | ·· |  | ·· | ·· | ·· |  |
| **Caribbean** |  |  |  |  |  |  |  |  |  |  |  |  |  |  |  |
| Antigua & Barbuda | 378 | 1194 | 2023 | NK | NK | ·· |  | 3·0 (0·4, 7·5) | < 500 (<500,<500) | 2005 | 122 | ·· | ·· | ·· |  |
| Bahamas | 1771 | 1324 | 2021 | ·· | ·· | ·· |  | ·· | ·· | ·· |  | ·· | ·· | ·· |  |
| Barbados | 671 | 732 | 2023 | ·· | ·· | ·· |  | ·· | ·· | ·· |  | ·· | ·· | ·· |  |
| Bermuda | 120 | 578 | 2021 | ·· | ·· | ·· |  | ·· | ·· | ·· |  | ·· | ·· | ·· |  |
| Cuba | 87152 | 1602 | 2020 | ·· | ·· | ·· |  | ·· | ·· | ·· |  | ·· | ·· | ·· |  |
| Dominica | 257 | 997 | 2024 | NK | NK | ·· |  | 2·6 (0·7, 5·5) | < 500 (<500,<500) | 2005 | 122 | ·· | ·· | ·· |  |
| Dominican Republic | 25311 | 692 | 2024 | ·· | ·· | ·· |  | ·· | ·· | ·· |  | ·· | ·· | ·· |  |
| Grenada | 379 | 914 | 2023 | NK | NK | ·· |  | 2·2 (0·3, 5·5) | < 500 (<500,<500) | 2005 | 122 | ·· | ·· | ·· |  |
| Haiti | 7260 | 203 | 2024 | ·· | ·· | ·· |  | ·· | ·· | ·· |  | ·· | ·· | ·· |  |
| Jamaica | 3417 | 335 | 2022 | ·· | ·· | ·· |  | 3·5 (2·4, 4·8) | < 500 (<500,<500) | 2008 | 123 | ·· | ·· | ·· |  |
| Commonwealth of Puerto Rico | 5583 | 561 | 2022 | ·· | ·· | ·· |  | ·· | ·· | ·· |  | ·· | ·· | ·· |  |
| Saint Kitts & Nevis | 154 | 950 | 2022 | NK | NK | ·· |  | 2·4 (0·5, 5·3) | < 500 (<500,<500) | 2005 | 122 | ·· | ·· | ·· |  |
| Saint Lucia | 557 | 855 | 2023 | NK | NK | ·· |  | 2·0 (0·8, 3·8) | < 500 (<500,<500) | 2005 | 122 | ·· | ·· | ·· |  |
| Saint Vincent & the Grenadines | 396 | 1094 | 2024 | NK | NK | ·· |  | 4·1 (2·2, 6·4) | < 500 (<500,<500) | 2005 | 122 | ·· | ·· | ·· |  |
| Trinidad & Tobago | 3692 | 696 | 2021 | NK | NK | ·· |  | ·· | ·· | ·· |  | ·· | ·· | ·· |  |
[truncated: 1,107,988 more chars]
