# Supplementary material for: The global epidemiology of injecting drug use, HIV, viral hepatitis and tuberculosis among people who are incarcerated: a multistage systematic review
Source: Int J Drug Policy. Author manuscript; Available in PMC 2026 Apr 8. (PMC13058553; doi:10.1016/j.drugpo.2025.105062)
Supplement: 6 [file NIHMS2157186-supplement-6.docx]

## Appendix 16.1: JBI Prevalence Risk of Bias Assessment for included papers with IDU estimates

| **Country** | **Author** | **Year of Publication** | **Risk of Bias Score** | | | | | | | | | | **Reference** |
| --- | --- | --- | --- | --- | --- | --- | --- | --- | --- | --- | --- | --- | --- |
|  |  |  | **1** | **2** | **3** | **4** | **5** | **6** | **7** | **8** | **9** | **Total** |  |
| **Eastern Europe** |  |  |  |  |  |  |  |  |  |  |  |  |  |
| Azerbaijan | Azbel | 2015 | 1 | 0 | 1 | 1 | 0 | 1 | 1 | 1 | 1 | 7 | ^1^ |
| Bosnia & Herzegovina | Ravlija | 2014 | 1 | 0 | 1 | 0 | 0 | 1 | 1 | 1 | 1 | 6 | ^2^ |
| Bulgaria | Popov | 2011 | 0 | 0 | 1 | 0 | 0 | 1 | 1 | 1 | 1 | 5 | ^3^ |
| Hungary | Gyarmathy | 2003 | 1 | 0 | 1 | 1 | 0 | 1 | 1 | 1 | 1 | 7 | ^4^ |
| Hungary | Treso | 2012 | 1 | 0 | 1 | 0 | 0 | 1 | 1 | 1 | 0 | 5 | ^5^ |
| Latvia | Kurcalte | 2023 | 1 | 0 | 1 | 1 | 0 | 1 | 1 | 1 | 1 | 7 | ^6^ |
| Lithuania | Narkauskaitė | 2010 | 0 | 0 | 0 | 1 | 0 | 1 | 1 | 1 | 1 | 5 | ^7^ |
| Lithuania | Rasimaite | 2023 | 1 | 0 | 1 | 0 | 0 | 1 | 1 | 1 | 1 | 6 | ^8^ |
| Ukraine | Kiriazova | 2017 | 1 | 0 | 1 | 0 | 0 | 1 | 1 | 1 | 1 | 6 | ^9^ |
| Ukraine | Azbel | 2013 | 1 | 0 | 1 | 1 | 0 | 1 | 1 | 1 | 1 | 7 | ^10^ |
| Ukraine | Balakireva | 2012 | 1 | 1 | 1 | 0 | 1 | 1 | 1 | 1 | 1 | 8 | ^11^ |
| **Western Europe** |  |  |  |  |  |  |  |  |  |  |  |  |  |
| Austria | Silbernagl | 2018 | 1 | 0 | 0 | 0 | 0 | 1 | 1 | 1 | 1 | 5 | ^12^ |
| Austria | Silbernagl | 2018 | 1 | 0 | 0 | 0 | 0 | 1 | 1 | 1 | 1 | 5 | ^12^ |
| Belgium | Busschotts | 2021 | 1 | 0 | 1 | 0 | 0 | 1 | 1 | 1 | 0 | 5 | ^13^ |
| Croatia | Burek | 2010 | 1 | 0 | 1 | 1 | 0 | 1 | 1 | 1 | 1 | 7 | ^14^ |
| Croatia | Burek | 2010 | 1 | 0 | 0 | 1 | 0 | 1 | 1 | 1 | 1 | 6 | ^14^ |
| Croatia | Vilibic-Cavlek | 2011 | 1 | 0 | 0 | 1 | 0 | 1 | 1 | 1 | 0 | 5 | ^15^ |
| Denmark | Christensen | 2000 | 0 | 0 | 1 | 0 | 0 | 1 | 1 | 1 | 1 | 5 | ^16^ |
| England and Wales | Boys | 2002 | 1 | 0 | 1 | 0 | 0 | 1 | 1 | 1 | 1 | 6 | ^17^ |
| England and Wales | Plugge | 2009 | 1 | 0 | 1 | 0 | 0 | 1 | 1 | 1 | 0 | 5 | ^18^ |
| England and Wales | Mahto | 2008 | 0 | 0 | 1 | 1 | 0 | 1 | 1 | 1 | 1 | 6 | ^19^ |
| England and Wales | Jack | 2020 | 0 | 0 | 0 | 0 | 0 | 1 | 1 | 1 | 1 | 4 | ^20^ |
| England and Wales | Aisyah | 2017 | 0 | 0 | 1 | 0 | 0 | 1 | 1 | 1 | 1 | 5 | ^21^ |
| England and Wales | Jack | 2013 | 0 | 0 | 1 | 0 | 0 | 1 | 1 | 1 | 0 | 4 | ^22^ |
| England and Wales | Weild | 2000 | 1 | 0 | 1 | 0 | 0 | 1 | 1 | 1 | 1 | 6 | ^23^ |
| Finland | Rautanen | 2024 | 1 | 0 | 0 | 1 | 0 | 1 | 1 | 1 | 1 | 6 | ^24^ |
| Finland | Viitanen | 2011 | 0 | 0 | 0 | 1 | 0 | 1 | 1 | 1 | 1 | 5 | ^25^ |
| Finland | Rautanen | 2024 | 1 | 0 | 1 | 1 | 0 | 1 | 1 | 1 | 1 | 7 | ^24^ |
| Finland | Viitanen | 2011 | 1 | 0 | 1 | 1 | 0 | 1 | 1 | 1 | 1 | 7 | ^25^ |
| Finland | Rautanen | 2024 | 1 | 0 | 1 | 1 | 0 | 1 | 1 | 1 | 1 | 7 | ^24^ |
| France | Jacomet | 2016 | 1 | 0 | 1 | 1 | 0 | 1 | 1 | 1 | 1 | 7 | ^26^ |
| France | Messiah | 2001 | 0 | 0 | 1 | 0 | 0 | 1 | 1 | 1 | 1 | 5 | ^27^ |
| France | Rotily | 2000 | 0 | 1 | 0 | 0 | 1 | 1 | 1 | 1 | 1 | 6 | ^28^ |
| France | Rotily | 2000 | 0 | 0 | 1 | 1 | 0 | 1 | 1 | 1 | 1 | 6 | ^29^ |
| France | Verneuil | 2008 | 0 | 0 | 1 | 1 | 0 | 1 | 1 | 1 | 1 | 6 | ^30^ |
| France | Messiah | 2001 | 0 | 0 | 1 | 0 | 0 | 1 | 1 | 1 | 0 | 4 | ^27^ |
| France | Remy | 2021 | 0 | 0 | 1 | 0 | 0 | 1 | 1 | 1 | 0 | 4 | ^31^ |
| France | Marzo | 2009 | 1 | 0 | 1 | 1 | 0 | 1 | 1 | 1 | 1 | 7 | ^32^ |
| France | Roux | 2014 | 1 | 0 | 1 | 1 | 0 | 1 | 1 | 1 | 1 | 7 | ^33^ |
| Germany | Schulte | 2009 | 1 | 0 | 1 | 0 | 0 | 1 | 1 | 1 | 1 | 6 | ^34^ |
| Germany | Rotily | 2000 | 0 | 1 | 0 | 0 | 1 | 1 | 1 | 1 | 1 | 6 | ^28^ |
| Greece | Koulierakis | 2000 | 1 | 0 | 1 | 1 | 0 | 1 | 1 | 1 | 1 | 7 | ^35^ |
| Greece | Fotiadou | 2004 | 0 | 0 | 0 | 0 | 0 | 1 | 1 | 1 | 1 | 4 | ^36^ |
| Greece | Koulierakis | 2006 | 0 | 0 | 0 | 0 | 0 | 1 | 1 | 1 | 1 | 4 | ^37^ |
| Greece | Koulierakis | 2003 | 0 | 0 | 1 | 1 | 0 | 1 | 1 | 1 | 1 | 6 | ^38^ |
| Ireland | Crowley | 2019 | 0 | 0 | 1 | 1 | 0 | 1 | 1 | 1 | 0 | 5 | ^39^ |
| Ireland | Allwright | 2000 | 1 | 0 | 1 | 1 | 0 | 1 | 1 | 1 | 1 | 7 | ^40^ |
| Ireland | Drummond | 2014 | 1 | 0 | 1 | 0 | 0 | 1 | 1 | 1 | 1 | 6 | ^41^ |
| Ireland | Drummond | 2014 | 1 | 0 | 1 | 0 | 0 | 1 | 1 | 1 | 1 | 6 | ^41^ |
| Ireland | Long | 2001 | 1 | 0 | 1 | 1 | 0 | 1 | 1 | 1 | 1 | 7 | ^42^ |
| Italy | Scelza | 2022 | 0 | 0 | 0 | 1 | 0 | 1 | 1 | 1 | 1 | 5 | ^43^ |
| Italy | Marco | 2020 | 0 | 0 | 1 | 1 | 0 | 1 | 1 | 1 | 1 | 6 | ^44^ |
| Italy | Babudieri | 2005 | 1 | 0 | 1 | 1 | 0 | 1 | 1 | 1 | 1 | 7 | ^45^ |
| Italy | Rotily | 2000 | 0 | 1 | 0 | 0 | 1 | 1 | 1 | 1 | 1 | 6 | ^28^ |
| Italy | Fiore | 2021 | 1 | 0 | 1 | 1 | 0 | 1 | 1 | 1 | 1 | 7 | ^46^ |
| Italy | Izzo | 2021 | 0 | 0 | 0 | 1 | 0 | 1 | 1 | 1 | 0 | 4 | ^47^ |
| Italy | Ciccarese | 2020 | 0 | 0 | 1 | 1 | 0 | 1 | 1 | 1 | 0 | 5 | ^48^ |
| Luxembourg | Teyssier | 2023 | 1 | 0 | 0 | 1 | 0 | 1 | 1 | 1 | 1 | 6 | ^49^ |
| Netherlands | Rotily | 2000 | 0 | 0 | 0 | 0 | 0 | 1 | 1 | 1 | 1 | 4 | ^28^ |
| Northern Ireland | Danis | 2007 | 1 | 0 | 1 | 0 | 0 | 1 | 1 | 1 | 1 | 6 | ^50^ |
| Norway | Hannula | 2021 | 0 | 0 | 0 | 1 | 0 | 1 | 1 | 1 | 1 | 5 | ^51^ |
| Norway | Butken | 2020 | 1 | 0 | 1 | 1 | 0 | 1 | 1 | 1 | 1 | 7 | ^52^ |
| Portugal | Passadouro | 2004 | 0 | 0 | 1 | 1 | 0 | 1 | 1 | 1 | 1 | 6 | ^53^ |
| Portugal | Garcia | 2004 | 0 | 0 | 1 | 0 | 0 | 1 | 1 | 1 | 1 | 5 | ^54^ |
| Portugal | Barros | 2008 | 0 | 0 | 1 | 0 | 0 | 1 | 1 | 1 | 1 | 5 | ^55^ |
| Scotland | Arora | 2020 | 1 | 0 | 1 | 0 | 0 | 1 | 1 | 1 | 1 | 6 | ^56^ |
| Scotland | Taylor | 2012 | 1 | 0 | 1 | 1 | 0 | 1 | 1 | 1 | 1 | 7 | ^57^ |
| Scotland | Morrison | 2001 | 0 | 0 | 1 | 1 | 0 | 1 | 1 | 1 | 1 | 6 | ^58^ |
| Scotland | Rotily | 2000 | 0 | 1 | 1 | 0 | 1 | 1 | 1 | 1 | 1 | 7 | ^28^ |
| Spain | García-Guerrero | 2010 | 1 | 0 | 1 | 1 | 0 | 1 | 1 | 1 | 1 | 7 | ^59^ |
| Spain | Sanchez Recio | 2016 | 0 | 0 | 1 | 1 | 0 | 1 | 1 | 1 | 1 | 6 | ^60^ |
| Spain | Ferrer-Castro | 2012 | 0 | 0 | 1 | 0 | 0 | 1 | 1 | 1 | 1 | 5 | ^61^ |
| Spain | Brime | 2022 | 1 | 0 | 1 | 0 | 0 | 1 | 1 | 1 | 1 | 6 | ^62^ |
| Spain | Ferrer-Castro | 2012 | 0 | 0 | 1 | 0 | 0 | 1 | 1 | 1 | 1 | 5 | ^61^ |
| Spain | Murcia | 2009 | 0 | 0 | 1 | 1 | 0 | 1 | 1 | 1 | 1 | 6 | ^63^ |
| Spain | Cuadrado | 2018 | 0 | 0 | 1 | 1 | 0 | 1 | 1 | 1 | 1 | 6 | ^64^ |
| Spain | Martin | 2001 | 0 | 0 | 1 | 1 | 0 | 1 | 1 | 1 | 1 | 6 | ^65^ |
| Sweden | Rotily | 2000 | 0 | 0 | 0 | 0 | 0 | 1 | 1 | 1 | 1 | 4 | ^28^ |
| Switzerland | Baggio | 2020 | 0 | 0 | 1 | 0 | 0 | 1 | 1 | 0 | 0 | 3 | ^66^ |
| Switzerland | Wolff | 2012 | 0 | 0 | 1 | 1 | 0 | 1 | 1 | 1 | 0 | 5 | ^67^ |
| Switzerland | Pala | 2018 | 0 | 0 | 1 | 1 | 0 | 1 | 1 | 1 | 1 | 6 | ^68^ |
| Switzerland | Baggio | 2020 | 0 | 0 | 1 | 0 | 0 | 1 | 1 | 1 | 1 | 5 | ^66^ |
| **East and South East Asia** |  |  |  |  |  |  |  |  |  |  |  |  |  |
| Indonesia | Blogg | 2014 | 1 | 0 | 1 | 0 | 0 | 1 | 1 | 1 | 1 | 6 | ^69^ |
| Indonesia | Ministry of Health Republic of Indonesia | 2011 | 1 | 0 | 1 | 0 | 0 | 1 | 1 | 1 | 1 | 6 | ^70^ |
| Indonesia | Sembiring | 2018 | 0 | 0 | 1 | 0 | 0 | 1 | 1 | 1 | 1 | 5 | ^71^ |
| Taiwan | Lu | 2021 | 0 | 0 | 1 | 1 | 0 | 1 | 1 | 1 | 1 | 6 | ^72^ |
| Taiwan | Feng | 2012 | 0 | 0 | 1 | 0 | 0 | 1 | 1 | 1 | 1 | 5 | ^73^ |
| Taiwan | Lin | 2010 | 1 | 0 | 1 | 1 | 0 | 1 | 1 | 1 | 1 | 7 | ^74^ |
| Thailand | Harnpariphan | 2022 | 0 | 0 | 1 | 1 | 0 | 1 | 1 | 1 | 1 | 6 | ^75^ |
| **South Asia** |  |  |  |  |  |  |  |  |  |  |  |  |  |
| Afghanistan | John Hopkins University | 2011 | 0 | 0 | 1 | 0 | 0 | 1 | 1 | 1 | 1 | 5 | ^76^ |
| Afghanistan | John Hopkins University | 2011 | 0 | 0 | 1 | 0 | 0 | 1 | 1 | 1 | 1 | 5 | ^76^ |
| India | Choudhury | 2016 | 1 | 0 | 1 | 0 | 0 | 1 | 1 | 1 | 1 | 6 | ^77^ |
| India | National AIDS Control Organization | 2019 | 1 | 0 | 1 | 1 | 0 | 1 | 1 | 1 | 1 | 7 | ^78^ |
| India | National AIDS Control Organization | 2022 | 1 | 0 | 1 | 1 | 0 | 1 | 1 | 1 | 1 | 7 | ^79^ |
| India | Ramamoorthy | 2016 | 0 | 0 | 1 | 1 | 0 | 1 | 1 | 1 | 1 | 6 | ^80^ |
| Iran (Islamic Republic of) |  | 2009 | 1 | 0 | 1 | 0 | 0 | 1 | 1 | 1 | 0 | 5 | ^81^ |
| Iran (Islamic Republic of) | Moradi | 2019 | 1 | 0 | 1 | 1 | 0 | 1 | 1 | 1 | 0 | 6 | ^82^ |
| Iran (Islamic Republic of) | Seyedalinaghi | 2023 | 0 | 0 | 0 | 1 | 0 | 1 | 1 | 1 | 1 | 5 | ^83^ |
| Iran (Islamic Republic of) | Khajehkazemi | 2014 | 1 | 0 | 1 | 0 | 0 | 1 | 1 | 1 | 1 | 6 | ^84^ |
| Iran (Islamic Republic of) | Zamani | 2010 | 0 | 0 | 1 | 1 | 0 | 1 | 1 | 1 | 1 | 6 | ^85^ |
| Iran (Islamic Republic of) | Mirzazadeh | 2018 | 1 | 0 | 1 | 1 | 0 | 1 | 1 | 1 | 1 | 7 | ^86^ |
| Iran (Islamic Republic of) | Hariri | 2020 | 0 | 0 | 1 | 1 | 0 | 1 | 1 | 1 | 1 | 6 | ^87^ |
| Iran (Islamic Republic of) | Khajedaluee | 2016 | 1 | 0 | 1 | 0 | 0 | 1 | 1 | 1 | 0 | 5 | ^88^ |
| Iran (Islamic Republic of) | Ataei | 2011 | 0 | 0 | 0 | 1 | 0 | 1 | 1 | 1 | 1 | 5 | ^89^ |
| Iran (Islamic Republic of) | Mamani | 2016 | 0 | 0 | 1 | 0 | 0 | 1 | 1 | 1 | 0 | 4 | ^90^ |
| Iran (Islamic Republic of) | Shahesmaeili | 2022 | 1 | 0 | 1 | 0 | 0 | 1 | 1 | 1 | 0 | 5 | ^91^ |
| Iran (Islamic Republic of) | Mohtasham Amiri | 2021 | 0 | 0 | 1 | 1 | 0 | 1 | 1 | 1 | 0 | 5 | ^92^ |
| Iran (Islamic Republic of) | Hariri | 2021 | 0 | 0 | 1 | 1 | 0 | 1 | 1 | 1 | 1 | 6 | ^93^ |
| Iran (Islamic Republic of) | Navadeh | 2013 | 1 | 0 | 1 | 0 | 0 | 1 | 1 | 1 | 1 | 6 | ^94^ |
| Iran (Islamic Republic of) | Shahesmaeili | 2022 | 1 | 0 | 1 | 0 | 0 | 1 | 1 | 1 | 1 | 6 | ^91^ |
| Iran (Islamic Republic of) | Moradi | 2020 | 1 | 0 | 1 | 0 | 0 | 1 | 1 | 1 | 1 | 6 | ^95^ |
| Iran (Islamic Republic of) | Moradi | 2018 | 1 | 0 | 1 | 0 | 0 | 1 | 1 | 1 | 0 | 5 | ^96^ |
| Iran (Islamic Republic of) | Shahesmaeili | 2022 | 1 | 0 | 1 | 0 | 0 | 1 | 1 | 1 | 1 | 6 | ^91^ |
| Iran (Islamic Republic of) | Mirzazadeh | 2018 | 0 | 0 | 0 | 1 | 0 | 1 | 1 | 1 | 1 | 5 | ^86^ |
| Iran (Islamic Republic of) | Nokhodian | 2012 | 0 | 0 | 0 | 1 | 0 | 1 | 1 | 1 | 1 | 5 | ^97^ |
| Iran (Islamic Republic of) | SeyedAlinaghi | 2017 | 0 | 0 | 1 | 0 | 0 | 1 | 1 | 1 | 1 | 5 | ^98^ |
| Iran (Islamic Republic of) | Khezri | 2022 | 1 | 0 | 1 | 1 | 0 | 1 | 1 | 1 | 1 | 7 | ^99^ |
| Nepal | Shrestha | 2018 | 0 | 0 | 1 | 1 | 0 | 1 | 1 | 1 | 1 | 6 | ^100^ |
| Nepal | Shrestha | 2018 | 0 | 0 | 1 | 1 | 0 | 1 | 1 | 1 | 0 | 5 | ^101^ |
| Pakistan | Kazi | 2010 | 0 | 0 | 1 | 1 | 0 | 1 | 1 | 1 | 0 | 5 | ^102^ |
| Pakistan | Memon | 2012 | 0 | 0 | 1 | 0 | 0 | 1 | 1 | 1 | 1 | 5 | ^103^ |
| Pakistan | Butt | 2010 | 0 | 0 | 1 | 0 | 0 | 1 | 1 | 1 | 1 | 5 | ^104^ |
| Pakistan | Khan | 2019 | 0 | 0 | 0 | 0 | 0 | 1 | 1 | 1 | 1 | 4 | ^105^ |
| Sri Lanka | Niriella | 2015 | 0 | 0 | 1 | 0 | 0 | 1 | 1 | 1 | 1 | 5 | ^106^ |
| **Central Asia** |  |  |  |  |  |  |  |  |  |  |  |  |  |
| Kyrgyzstan | Moller | 2008 | 0 | 0 | 1 | 0 | 0 | 1 | 1 | 1 | 1 | 5 | ^107^ |
| Kyrgyzstan | Azbel | 2016 | 1 | 0 | 1 | 1 | 0 | 1 | 1 | 1 | 1 | 7 | ^108^ |
| Kyrgyzstan | Moller | 2008 | 0 | 0 | 1 | 0 | 0 | 1 | 1 | 1 | 1 | 5 | ^107^ |
| Tajikistan | Winetsky | 2014 | 1 | 0 | 1 | 1 | 0 | 1 | 1 | 1 | 1 | 7 | ^109^ |
| **Caribbean** |  |  |  |  |  |  |  |  |  |  |  |  |  |
| Commonwealth of Puerto Rico | Peña-Orellana | 2011 | 1 | 0 | 1 | 0 | 0 | 1 | 1 | 1 | 1 | 6 | ^110^ |
| **Latin America** |  |  |  |  |  |  |  |  |  |  |  |  |  |
| Brazil | Falquetto | 2013 | 0 | 0 | 1 | 0 | 0 | 1 | 1 | 1 | 1 | 5 | ^111^ |
| Brazil | Lopes | 2001 | 0 | 0 | 1 | 1 | 0 | 1 | 1 | 1 | 0 | 5 | ^112^ |
| Brazil | Rosa | 2012 | 0 | 0 | 0 | 1 | 0 | 1 | 1 | 1 | 1 | 5 | ^113^ |
| Brazil | Pompilio | 2011 | 1 | 0 | 1 | 0 | 0 | 1 | 1 | 1 | 1 | 6 | ^114^ |
| Brazil | Guimarães | 2001 | 0 | 0 | 1 | 1 | 0 | 1 | 1 | 1 | 1 | 6 | ^115^ |
| Brazil | Strazza | 2007 | 0 | 0 | 1 | 1 | 0 | 1 | 1 | 1 | 1 | 6 | ^116^ |
| Brazil | de Albuquerque | 2013 | 0 | 0 | 1 | 1 | 0 | 1 | 1 | 1 | 1 | 6 | ^117^ |
| Brazil | Miranda | 2000 | 0 | 0 | 0 | 1 | 0 | 1 | 1 | 1 | 1 | 5 | ^118^ |
| Brazil | Felisberto | 2019 | 0 | 0 | 0 | 1 | 0 | 1 | 1 | 1 | 1 | 5 | ^119^ |
| Brazil | El Maerrawi | 2015 | 0 | 0 | 1 | 1 | 0 | 1 | 1 | 1 | 1 | 6 | ^120^ |
| Brazil | Coelho | 2009 | 0 | 0 | 1 | 0 | 0 | 1 | 1 | 1 | 1 | 5 | ^121^ |
| Brazil | Massad | 1999 | 0 | 0 | 1 | 0 | 0 | 1 | 1 | 1 | 1 | 5 | ^122^ |
| Brazil | Felisberto | 2016 | 0 | 0 | 0 | 0 | 0 | 1 | 1 | 1 | 1 | 4 | ^123^ |
| Brazil | Santos | 2011 | 1 | 0 | 1 | 1 | 0 | 1 | 1 | 1 | 1 | 7 | ^124^ |
| Brazil | Strazza | 2004 | 0 | 0 | 1 | 1 | 0 | 1 | 1 | 1 | 1 | 6 | ^125^ |
| Colombia | Sanchez-Vanegas | 2021 | 0 | 0 | 1 | 1 | 0 | 1 | 1 | 1 | 1 | 6 | ^126^ |
| Colombia | Castillo | 2017 | 1 | 0 | 1 | 0 | 0 | 1 | 1 | 1 | 1 | 6 | ^127^ |
| Guatemala | Alvarez Rodriguez | 2013 | 1 | 0 | 1 | 1 | 0 | 1 | 1 | 1 | 1 | 7 | ^128^ |
| Mexico | Belaunzaran-Zamudio | 2017 | 1 | 0 | 1 | 1 | 0 | 1 | 1 | 1 | 0 | 6 | ^129^ |
| Mexico | Bautista-Arredondo | 2015 | 1 | 0 | 1 | 1 | 0 | 1 | 1 | 1 | 1 | 7 | ^130^ |
| Mexico | Alvarado-Esquivel | 2005 | 0 | 0 | 0 | 1 | 0 | 1 | 1 | 1 | 1 | 5 | ^131^ |
| Mexico | Bautista-Arredondo | 2015 | 1 | 0 | 1 | 1 | 0 | 1 | 1 | 1 | 1 | 7 | ^130^ |
| Mexico | M Gonzalez | 2011 | 0 | 0 | 1 | 1 | 0 | 1 | 1 | 1 | 0 | 5 | ^132^ |
| Venezuela (Bolivarian Republic of) | Monsalve-Castillo | 2009 | 0 | 0 | 1 | 0 | 0 | 1 | 1 | 1 | 1 | 5 | ^133^ |
| **North America** |  |  |  |  |  |  |  |  |  |  |  |  |  |
| Canada | Courtemanche | 2018 | 1 | 0 | 1 | 0 | 0 | 1 | 1 | 1 | 0 | 5 | ^134^ |
| Canada | Calzavara | 2003 | 1 | 0 | 1 | 0 | 0 | 1 | 1 | 1 | 1 | 6 | ^135^ |
| Canada | Martin | 2005 | 0 | 0 | 0 | 0 | 0 | 1 | 1 | 1 | 1 | 4 | ^136^ |
| Canada | Courtemanche | 2018 | 1 | 0 | 1 | 1 | 0 | 1 | 1 | 1 | 0 | 6 | ^134^ |
| Canada | Dussault | 2020 | 0 | 0 | 1 | 1 | 0 | 1 | 1 | 1 | 1 | 6 | ^137^ |
| Canada | Poulin | 2007 | 1 | 0 | 1 | 1 | 0 | 1 | 1 | 1 | 1 | 7 | ^138^ |
| Canada | Besney | 2018 | 0 | 0 | 0 | 1 | 0 | 1 | 1 | 1 | 1 | 5 | ^139^ |
| Canada | Calzavara | 2007 | 1 | 0 | 1 | 1 | 0 | 1 | 1 | 1 | 1 | 7 | ^140^ |
| Canada | Nolan | 2017 | 1 | 0 | 1 | 1 | 0 | 1 | 1 | 1 | 1 | 7 | ^141^ |
| Canada | Bonnycastle | 2011 | 0 | 0 | 1 | 0 | 0 | 1 | 1 | 1 | 1 | 5 | ^142^ |
| Canada | Poulin | 2007 | 1 | 0 | 1 | 1 | 0 | 1 | 1 | 1 | 1 | 7 | ^138^ |
| Canada | Calzavara | 2007 | 1 | 0 | 1 | 0 | 0 | 1 | 1 | 1 | 1 | 6 | ^140^ |
| United States of America | Begier | 2010 | 1 | 0 | 1 | 0 | 0 | 1 | 1 | 1 | 1 | 6 | ^143^ |
| United States of America | Kendrick | 2004 | 0 | 0 | 1 | 0 | 0 | 1 | 1 | 1 | 1 | 5 | ^144^ |
| United States of America | Swartz | 2004 | 1 | 0 | 1 | 0 | 0 | 1 | 1 | 1 | 1 | 6 | ^145^ |
| United States of America | Khan | 2005 | 0 | 0 | 1 | 1 | 0 | 1 | 1 | 1 | 1 | 6 | ^146^ |
| United States of America | Mullings | 2003 | 1 | 0 | 1 | 1 | 0 | 1 | 1 | 1 | 1 | 7 | ^147^ |
| United States of America | Kim | 2013 | 1 | 0 | 1 | 1 | 0 | 1 | 1 | 1 | 0 | 6 | ^148^ |
| United States of America | Trevino | 2013 | 0 | 0 | 0 | 1 | 0 | 1 | 1 | 1 | 1 | 5 | ^149^ |
| United States of America | Akiyama | 2017 | 0 | 0 | 1 | 1 | 0 | 1 | 1 | 1 | 1 | 6 | ^150^ |
| United States of America | Adams | 2013 | 0 | 0 | 1 | 0 | 0 | 1 | 1 | 1 | 1 | 5 | ^151^ |
| United States of America | Alvarez | 2014 | 0 | 0 | 1 | 1 | 0 | 1 | 1 | 1 | 1 | 6 | ^152^ |
| United States of America | Abiona | 2009 | 1 | 0 | 1 | 1 | 0 | 1 | 1 | 1 | 1 | 7 | ^153^ |
| United States of America | Brinkley-Rubinstein | 2020 | 1 | 0 | 1 | 0 | 0 | 1 | 1 | 1 | 1 | 6 | ^154^ |
| United States of America | MacGowan | 2009 | 1 | 0 | 1 | 0 | 0 | 1 | 1 | 1 | 1 | 6 | ^155^ |
| United States of America | Lally | 2006 | 0 | 0 | 0 | 0 | 0 | 1 | 1 | 1 | 1 | 4 | ^156^ |
| United States of America | Abiona | 2009 | 1 | 0 | 1 | 0 | 0 | 1 | 1 | 1 | 1 | 6 | ^157^ |
| United States of America | Tartaro | 2013 | 0 | 0 | 1 | 1 | 0 | 1 | 1 | 1 | 0 | 5 | ^158^ |
| United States of America | Keleekai | 2011 | 0 | 0 | 1 | 1 | 0 | 1 | 1 | 1 | 1 | 6 | ^159^ |
| United States of America | Beckwith | 2010 | 1 | 0 | 1 | 0 | 0 | 1 | 1 | 1 | 1 | 6 | ^160^ |
| United States of America | Nijhawan | 2016 | 0 | 0 | 1 | 1 | 0 | 1 | 1 | 1 | 1 | 6 | ^161^ |
| United States of America | Abiona | 2009 | 1 | 0 | 1 | 0 | 0 | 1 | 1 | 1 | 1 | 6 | ^157^ |
| United States of America | McClelland | 2002 | 0 | 0 | 1 | 1 | 0 | 1 | 1 | 1 | 1 | 6 | ^162^ |
| United States of America | Harrison | 2001 | 0 | 0 | 1 | 0 | 0 | 1 | 1 | 1 | 1 | 5 | ^163^ |
| United States of America | Alvarez | 2014 | 0 | 0 | 1 | 1 | 0 | 1 | 1 | 1 | 1 | 6 | ^152^ |
| United States of America | Beckwith | 2007 | 0 | 0 | 0 | 0 | 0 | 1 | 1 | 1 | 1 | 4 | ^164^ |
| United States of America | Simonsen | 2015 | 0 | 0 | 1 | 1 | 0 | 1 | 1 | 1 | 1 | 6 | ^165^ |
| United States of America | Arndt | 2002 | 1 | 0 | 1 | 0 | 0 | 1 | 1 | 1 | 1 | 6 | ^166^ |
| United States of America | Deb | 2022 | 1 | 0 | 1 | 0 | 0 | 1 | 1 | 1 | 1 | 6 | ^167^ |
| United States of America | Macalino | 2005 | 0 | 0 | 1 | 0 | 0 | 1 | 1 | 1 | 0 | 4 | ^168^ |
| United States of America | Mullings | 2001 | 1 | 0 | 1 | 1 | 0 | 1 | 1 | 1 | 1 | 7 | ^169^ |
| United States of America | Keleekai | 2011 | 0 | 0 | 1 | 1 | 0 | 1 | 1 | 1 | 1 | 6 | ^159^ |
| United States of America | Katyal | 2018 | 0 | 0 | 1 | 1 | 0 | 1 | 1 | 1 | 1 | 6 | ^170^ |
| United States of America | Fox | 2005 | 1 | 0 | 1 | 0 | 0 | 1 | 1 | 1 | 1 | 6 | ^171^ |
| United States of America | Rice | 2010 | 0 | 0 | 1 | 0 | 0 | 1 | 1 | 1 | 1 | 5 | ^172^ |
| United States of America | Abiona | 2009 | 1 | 0 | 1 | 1 | 0 | 1 | 1 | 1 | 1 | 7 | ^153^ |
| United States of America | Gates | 2017 | 1 | 0 | 1 | 1 | 0 | 1 | 1 | 1 | 1 | 7 | ^173^ |
| United States of America | Altice | 2005 | 0 | 0 | 1 | 1 | 0 | 1 | 1 | 1 | 1 | 6 | ^174^ |
| United States of America | Wenger | 2014 | 0 | 0 | 1 | 1 | 0 | 1 | 1 | 1 | 1 | 6 | ^175^ |
| **Australasia** |  |  |  |  |  |  |  |  |  |  |  |  |  |
| Australia | Bah | 2024 | 1 | 0 | 1 | 1 | 0 | 1 | 1 | 1 | 1 | 7 | ^176^ |
| Australia | Butler | 2017 | 1 | 0 | 1 | 0 | 0 | 1 | 1 | 1 | 0 | 5 | ^177^ |
| Australia | Indig | 2010 | 1 | 0 | 1 | 1 | 0 | 1 | 1 | 1 | 1 | 7 | ^178^ |
| Australia | Butler | 2017 | 1 | 0 | 1 | 1 | 0 | 1 | 1 | 1 | 1 | 7 | ^177^ |
| Australia | Loxley | 2001 | 0 | 0 | 0 | 1 | 0 | 1 | 1 | 1 | 1 | 5 | ^179^ |
| Australia | Indig | 2010 | 1 | 0 | 1 | 0 | 0 | 1 | 1 | 1 | 0 | 5 | ^178^ |
| Australia | Hellard | 2004 | 1 | 0 | 1 | 1 | 0 | 1 | 1 | 1 | 1 | 7 | ^180^ |
| Australia | Kinner | 2006 | 1 | 0 | 0 | 0 | 0 | 1 | 1 | 1 | 1 | 5 | ^181^ |
| Australia | Butler | 2017 | 1 | 0 | 1 | 0 | 0 | 1 | 1 | 1 | 0 | 5 | ^177^ |
| Australia | Hajarizadeh | 2021 | 1 | 0 | 1 | 1 | 0 | 1 | 1 | 1 | 1 | 7 | ^182^ |
| Australia | Nicholson | 2001 | 0 | 0 | 0 | 1 | 0 | 1 | 1 | 1 | 1 | 5 | ^183^ |
| Australia | Gilles | 2008 | 0 | 0 | 0 | 0 | 0 | 1 | 1 | 1 | 1 | 4 | ^184^ |
| Australia | Butler | 2017 | 1 | 0 | 1 | 1 | 0 | 1 | 1 | 1 | 0 | 6 | ^177^ |
| Australia | Kevin | 2013 | 1 | 0 | 1 | 1 | 0 | 1 | 1 | 1 | 1 | 7 | ^185^ |
| Australia | Reekie | 2014 | 1 | 0 | 1 | 0 | 0 | 1 | 1 | 1 | 1 | 6 | ^186^ |
| Australia | Hajarizadeh | 2021 | 1 | 0 | 1 | 1 | 0 | 1 | 1 | 1 | 1 | 7 | ^182^ |
| Australia | Keen | 2020 | 1 | 0 | 1 | 0 | 0 | 1 | 1 | 1 | 1 | 6 | ^187^ |
| Australia | Butler | 2017 | 1 | 0 | 1 | 0 | 0 | 1 | 1 | 1 | 0 | 5 | ^177^ |
| Australia | Butler | 2003 | 1 | 0 | 1 | 1 | 0 | 1 | 1 | 1 | 1 | 7 | ^188^ |
| Australia | Hajarizadeh | 2021 | 1 | 0 | 1 | 1 | 0 | 1 | 1 | 1 | 1 | 7 | ^182^ |
| Australia | Larney | 2013 | 1 | 0 | 1 | 1 | 0 | 1 | 1 | 1 | 1 | 7 | ^189^ |
| Australia | Hockings | 2002 | 1 | 0 | 1 | 1 | 0 | 1 | 1 | 1 | 1 | 7 | ^190^ |
| Australia | Hajarizadeh | 2021 | 1 | 0 | 1 | 1 | 0 | 1 | 1 | 1 | 1 | 7 | ^182^ |
| Australia | Hajarizadeh | 2021 | 1 | 0 | 1 | 1 | 0 | 1 | 1 | 1 | 1 | 7 | ^182^ |
| Australia | Australian Institute of Health and Welfare | 2019 | 1 | 0 | 1 | 1 | 0 | 1 | 1 | 1 | 1 | 7 | ^191^ |
| Australia | Australian Institute of Health and Welfare | 2019 | 1 | 0 | 1 | 1 | 0 | 1 | 1 | 0 | 0 | 5 | ^191^ |
| Australia | Indig | 2010 | 1 | 0 | 0 | 1 | 0 | 1 | 1 | 1 | 1 | 6 | ^178^ |
| **Sub Saharan Africa** |  |  |  |  |  |  |  |  |  |  |  |  |  |
| Benin | Hessou | 2017 | 1 | 0 | 1 | 0 | 0 | 1 | 1 | 1 | 1 | 6 | ^192^ |
| Burkina Faso | Bureau d’Appui en Santé Publique | 2017 | 1 | 0 | 1 | 1 | 0 | 1 | 1 | 1 | 1 | 7 | ^193^ |
| Cameroon | Kowo | 2021 | 0 | 0 | 1 | 1 | 0 | 1 | 1 | 1 | 1 | 6 | ^194^ |
| Ethiopia | Kassa | 2021 | 0 | 0 | 1 | 1 | 0 | 1 | 1 | 1 | 1 | 6 | ^195^ |
| Ghana | Adjei | 2008 | 1 | 0 | 1 | 1 | 0 | 1 | 1 | 1 | 1 | 7 | ^196^ |
| Ghana | Ghana AIDS Commission | 2013 | 1 | 0 | 1 | 0 | 0 | 1 | 1 | 1 | 0 | 5 | ^197^ |
| Ghana | Ghana AIDS Commission | 2013 |  | 0 | 1 | 0 | 0 | 1 | 1 | 1 | 0 | 4 | ^197^ |
| Lesotho | Akeke | 2014 | 0 | 0 | 0 | 0 | 0 | 1 | 1 | 1 | 1 | 4 | ^198^ |
| Nigeria | Adoga | 2009 | 1 | 0 | 1 | 1 | 0 | 1 | 1 | 1 | 1 | 7 | ^199^ |
| Nigeria | Okafor | 2020 | 0 | 0 | 0 | 0 | 0 | 1 | 1 | 1 | 1 | 4 | ^200^ |
| Congo | National Council for the Fight against AIDS | 2012 | 1 | 0 | 0 | 1 | 0 | 1 | 1 | 1 | 1 | 6 | ^201^ |
| South Africa | Telisinghe | 2014 | 0 | 0 | 1 | 1 | 0 | 1 | 1 | 1 | 1 | 6 | ^202^ |
| Togo | Ekouevi | 2013 | 1 | 0 | 1 | 1 | 0 | 1 | 1 | 1 | 1 | 7 | ^203^ |
| **Middle East & North Africa** |  |  |  |  |  |  |  |  |  |  |  |  |  |
| Cyprus | EMCDDA | 2024 | 1 | 0 | 0 | 0 | 0 | 1 | 1 | 1 | 1 | 5 | ^204^ |
| Lebanon | Mahfoud | 2010 | 0 | 0 | 1 | 0 | 0 | 1 | 1 | 1 | 0 | 4 | ^205^ |
| Lebanon | Ministry of Health Lebanon | 2008 | 0 | 0 | 1 | 1 | 0 | 1 | 1 | 1 | 1 | 6 | ^206^ |
| Türkiye | Sahin | 2018 | 0 | 0 | 1 | 0 | 0 | 1 | 1 | 1 | 1 | 5 | ^207^ |
| Türkiye | Keten | 2016 | 0 | 0 | 1 | 1 | 0 | 1 | 1 | 1 | 0 | 5 | ^208^ |

**References**

1. Azbel L, Wickersham JA, Wegman MP, et al. Burden of substance use disorders, mental illness, and correlates of infectious diseases among soon-to-be released prisoners in Azerbaijan. *Drug and Alcohol Dependence* 2015.

2. Ravlija J, Vasilj I, Marijanovic I, Vasilj M. Risk behaviour of prison inmates in relation to HIV/STI. *Psychiatria Danubina* 2014.

3. Popov G, Plochev K. Prevalence and correlates of hepatitis C virus infection among inmates of Bulgarian prisons. *Clinical Microbiology and Infection* 2011.

4. Gyarmathy VA, Neaigus A, Szamado S. HIV risk behavior history of prison inmates in Hungary. *AIDS Education and Prevention* 2003.

5. Tresó B, Barcsay E, Tarján A, et al. Prevalence and correlates of HCV, HVB, and HIV infection among prison inmates and staff, Hungary. *J Urban Health* 2012.

6. Kurcalte O SM, Manson E, Karadzhan J, Krastiÿš I, & Zalans O. Analysis of trends in the use of addictive substances in prisons in Latvia in 2022": Final report, 2023.

7. Narkauskaitė L, Juozulynas A, Mackiewicz Z, Venalis A, Utkuvienė J. Prevalence of psychoactive substances use in a Lithuanian women's prison revisited after 5 years. *Med Sci Monit* 2010.

8. Rasimaite B. DRID in prisons Lituhania. 2024.

9. Kiriazova T, Sereda Y. History of injection drug use mediates the effect of project start intervention in men who were released from prison in Ukraine. *Drug and Alcohol Dependence* 2017.

10. Azbel L, Wickersham JA, Grishaev Y, Dvoryak S, Altice FL. Burden of infectious diseases, substance use disorders, and mental illness among Ukrainian prisoners transitioning to the community. *PLoS One* 2013.

11. Balakireva O SV, Salabai N, Kryvoruk A. Analysis of HIV/AIDS Response in Penitentiary System of Ukraine: Ukrainian Institute for Social Research after Olexander Yaremenko; UNODC, 2012.

12. Silbernagl M, Slamanig R, Fischer G, Brandt L. Hepatitis C infection and psychiatric burden in two imprisoned cohorts: Young offenders and opioid-maintained prisoners. *Health Policy* 2018.

13. Busschots D, Kremer C, Bielen R, et al. A multicentre interventional study to assess blood-borne viral infections in Belgian prisons. *BMC Infectious Diseases* 2021.

14. Burek V, Horvat J, Butorac K, Mikulić R. Viral hepatitis B, C and HIV infection in Croatian prisons. *Epidemiology and Infection* 2010.

15. Vilibic-Cavlek T, Gjenero-Margan I, Retkovac B, et al. Sociodemographic characteristics and risk behaviors for HIV, hepatitis B and hepatitis C virus infection among Croatian male prisoners. *International Journal pf Prisoner Health* 2011; **7**(1): 28-31.

16. Christensen PB, Krarup HB, Niesters HGM, Norder H, Georgsen J. Prevalence and incidence of bloodborne viral infections among Danish prisoners. *European Journal of Epidemiology* 2000.

17. Boys A, Farrell M, Bebbington P, et al. Drug use and initiation in prison: results from a national prison survey in England and Wales. *Addiction* 2002.

18. Plugge E, Yudkin P, Douglas N. Changes in women's use of illicit drugs following imprisonment. *Addiction* 2009.

19. Mahto M, Zia S. Measuring the gap: from Home Office to the National Health Service in the provision of a one-stop shop sexual health service in a female prison in the UK. *Int J STD AIDS* 2008.

20. Jack K. Hepatitis C virus infection risk factors and test uptake in an English prison. *Gastrointestinal Nursing* 2020.

21. Aisyah DN, Shallcross L, Hayward A, et al. Hepatitis C among vulnerable populations: A seroprevalence study of homeless, people who inject drugs and prisoners in London. *J Viral Hepat* 2018.

22. Jack K, Smith SA, Lloyd J, Smith H, Thomson BJ. Hepatitis B and C management pathways in prison: An audit against UK NICE public health guidance (2013); 2013.

23. Weild AR, Gill ON, Bennett D, Livingstone SJ, Parry JV, Curran L. Prevalence of HIV, hepatitis B, and hepatitis C antibodies in prisoners in England and Wales: a national survey. *Commun Dis Public Health* 2000.

24. Rautanen M, Harald, K, & Tyni, S. Health and Wellbeing of Prisoners 2023 The Wattu IV Prison Population Study Finland, 2024.

25. Viitanen P, Vartiainen H, Aarnio J, et al. Hepatitis A, B, C and HIV infections among Finnish female prisoners--young females a risk group. *J Infect* 2011.

26. Jacomet C, Guyot-Lénat A, Bonny C, et al. Addressing the challenges of chronic viral infections and addiction in prisons: the PRODEPIST study. *European Journal of Public Health* 2016.

27. Messiah A, Escaffre N, Sannino N, Rotily M, Galinier-Pujol A. Sexuality in the age of AIDS in a vulnerable population: findings from a survey on prisoners. *POPULATION* 2001.

28. Rotily M, Weilandt C, Bird SM, et al. Surveillance of HIV infection and related risk behaviour in European prisons: A multicentre pilot study. *European journal of public health* 2001.

29. Rotily M, Delorme C, Galinier A, Escaffre N, Moatti JP. HIV risk behavior in prison and factors related to reincarceration among injecting drug users. *Presse Medicale* 2000.

30. Verneuil L, Vidal JS, Bekolo RZ, et al. Prevalence and risk factors of the whole spectrum of sexually transmitted diseases in male incoming prisoners in France. *European Journal of Clinical Microbiology & Infectious Diseases* 2009.

31. Remy AJ, Roy B, Hervet J. The 'prison zero hepatitits' project in france: a new pathway for hcv microelimination. *Heroin Addiction and Related Clinical Problems* 2021.

32. Marzo JN, Rotily M, Meroueh F, et al. Maintenance therapy and 3-year outcome of opioid-dependent prisoners: a prospective study in France (2003-06). *Addiction* 2009.

33. Roux P, Sagaon-Teyssier L, Lions C, Fugon L, Verger P, Carrieri MP. HCV seropositivity in inmates and in the general population: an averaging approach to establish priority prevention interventions. *BMJ Open* 2014.

34. Schulte B, Stover H, Thane K, Schreiter C, Gansefort D, Reimer J. Substitution treatment and HCV/HIV-infection in a sample of 31 German prisons for sentenced inmates. *International journal of prisoner health* 2009.

35. Koulierakis G, Gnardellis C, Agrafiotis D, Power KG. HIV risk behaviour correlates among injecting drug users in Greek prisons. *Addiction* 2000.

36. Fotiadou M, Livaditis M, Manou I, et al. Self-reported substance misuse in Greek male prisoners. *Eur Addict Res* 2004.

37. Koulierakis G. Drug use and related precautions prior to imprisonment, inside prison and intentions after release among Greek inmates. *Addiction Research & Theory* 2006.

38. Koulierakis G, Power KG, Gnardellis C, Agrafiotis D. HIV/AIDS related knowledge of inmates in Greek prisons. *Addiction Research & Theory* 2003.

39. Crowley D, Lambert JS, Betts-Symonds G, et al. The seroprevalence of untreated chronic hepatitis C virus (HCV) infection and associated risk factors in male Irish prisoners: a cross-sectional study, 2017. *Euro Surveill* 2019.

40. Allwright S, Bradley F, Long J, Barry J, Thornton L, Parry JV. Prevalence of antibodies to hepatitis B, hepatitis C, and HIV and risk factors in Irish prisoners: results of a national cross sectional survey. *Bmj* 2000.

41. Drummond A, Codd M, Donnelly N, et al. Study on the prevalence of drug use, including intravenous drug use, and blood-borne viruses among the Irish prisoner population. *Dublin: National Advisory Committee on Drugs and Alcohol* 2014.

42. Long J, Allwright S, Barry J, et al. Prevalence of antibodies to hepatitis B, hepatitis C, and HIV and risk factors in entrants to Irish prisons: a national cross sectional survey...including commentary by Bird SM. *BMJ: British Medical Journal (International Edition)* 2001.

43. Scelza G, Amato A, Pagano AM, et al. Effect of hepatitis C antiviral therapy on oral lichen planus and hyposalivation in inmates. *Annals of Gastroenterology* 2022.

44. Marco LD, Tullio P, Scalici F, et al. Screening and linkage to care of prisoners with HCV infection: the resist-HCV project. *Journal of Hepatology* 2020.

45. Babudieri S, Longo B, Sarmati L, et al. Correlates of HIV, HBV, and HCV infections in a prison inmate population: results from a multicentre study in Italy. *Journal of Medical Virology* 2005.

46. Fiore V, De Matteis G, Ranieri R, et al. HCV testing and treatment initiation in an Italian prison setting: A step-by-step model to micro-eliminate hepatitis C. *Int J Drug Policy* 2021.

47. Izzo C, Masarone M, Torre P, et al. Solving the Gap Between HCV Detection and Treatment in Prison HCV-RNA Testing and Treatment in a Cohort of Newly Arrived Convicts in Southern Italy. *Reviews on Recent Clinical Trials* 2022.

48. Ciccarese G, Drago F, Oddenino G, Crosetto S, Rebora A, Parodi A. Sexually transmitted infections in male prison inmates. Prevalence, level of knowledge and risky behaviours. *Infez Med* 2020.

49. Teyssier E, Kugener T, Seixas R, Berndt N., Seixas R., Kugener T., Origer A. PRS20: Luxembourg Results of the national quantitative study. 2023.

50. Danis K, Doherty L, McCartney M, McCarrol J, Kennedy H. Hepatitis and HIV in Northern Ireland prisons: a cross-sectional study. *Euro Surveill* 2007; **12**(1).

51. Hannula R, Soderholm J, Svendsen T, et al. Hepatitis C outreach project and cross-sectional epidemiology in high-risk populations in Trondheim, Norway. *Therapeutic Advances in Infectious Disease* 2021.

52. Bukten A, Lund IO, Kinner SA, et al. Factors associated with drug use in prison – results from the Norwegian offender mental health and addiction (NorMA) study. *HEALTH & JUSTICE* 2020.

53. Passadouro R. [Prevalence infections and risk factors due to HIV, Hepatitis B and C in a prison establishment in Leiria]. *Acta Medica Portuguesa* 2004.

54. Garcia A, Exposto F, Prieto E, Lopes M, Duarte A, da Silva RC. Association of Trichomonas vaginalis with sociodemographic factors and other STDs among females inmates in Lisbon. *International Journal of STD & AIDS* 2004.

55. Barros H, Ramos E, Lucas R. A survey of HIV and HCV among female prison inmates in Portugal. *Cent Eur J Public Health* 2008.

56. Arora G, Humphris G, Lahti S, Richards D, Freeman R. Depression, drugs and dental anxiety in prisons: A mediation model explaining dental decay experience. *Community Dent Oral Epidemiol* 2020.

57. Taylor A, Munro A, Allen E, et al. Low incidence of hepatitis C virus among prisoners in Scotland. [References]. *Addiction* 2013.

58. Morrison DS, Gilchrist G. Prison admission health screening as a measure of health needs. *Health Bull (Edinb)* 2001.

59. García-Guerrero J MMA, Sáiz de la Hoya Zamácola P, Vera-Remartínez EJ. Multi-centre study of the prevalence of latent tuberculosis infection amongst inmates in Spanish prisons. 2010.

60. Sánchez Recio R, Alonso Pérez de Ágreda JP, Santabárbara Serrano J. [Sexually transmitted infections in male prison inmates: risk of development of new diseases]. *Gac Sanit* 2016.

61. Ferrer-Castro V, Crespo-Leiro MR, García-Marcos LS, et al. [Evaluation of needle exchange program at Pereiro de Aguiar prison (Ourense, Spain): ten years of experience]. *Revista Espanola de Sanidad Penitenciaria* 2012.

62. Brime B, Llorens, N & Sanchez, E. SURVEY ON HEALTH AND DRUG USE IN THE INMATE POPULATION IN PENITENTIARY INSTITUTIONS (ESDIP), 2022.

63. Murcia J, Portilla J, Bedia M, et al. Chronic hepatitis C virus infection and associated liver disease among the inmates of a Spanish prison. *Enfermedades Infecciosas y Microbiologia Clinica* 2009.

64. Cuadrado A, Llerena S, Cobo C, et al. Microenvironment Eradication of Hepatitis C: A Novel Treatment Paradigm. *American Journal of Gastroenterology* 2018.

65. Martin V, Guerra JM, Cayla JA, Rodriguez JC, Blanco MD, Alcoba M. Incidence of tuberculosis and the importance of treatment of latent tuberculosis infection in a Spanish prison population. *Int J Tuberc Lung Dis* 2001.

66. Baggio S, Pala KC, Rieder JP, Tran NT, Wolff H, Getaz L. Infectious diseases in post-trial detention and comparisons with pre-trial detention: A study in Geneva, Switzerland. *Journal of Infection and Public Health* 2020.

67. Wolff H, Favrod-Coune T, Baroudi M, et al. Substitution treatment for all dependent opioid users is possible in jail: A case study of Switzerland. *Journal of General Internal Medicine* 2012.

68. Pala KC, Baggio S, Tran NT, Girardin F, Wolff H, Getaz L. Blood-borne and sexually transmitted infections: a cross-sectional study in a Swiss prison. *BMC Infectious Diseases* 2018.

69. Blogg S, Utomo, B,, Silitonga N, Hidayati DAN, Sattler G. Indonesian National Inmate Bio-Behavioral Survey for HIV and Syphilis Prevalence and Risk Behaviors in Prisons and Detention Centers, 2010. *SAGE OPEN* 2014.

70. Kemenkes R. Integrated Biological and Behavioural Survey 2011. *Jakarta: Kementerian Kesehatan RI* 2011.

71. Sembiring E, Ginting Y, Saragih RH. Factors associated with syphilis seropositive and Human Immunodeficiency Virus (HIV) infection among inmates at Lubuk Pakam prison, Indonesia. *1ST INT CONF ON TROP MED & INFECT DIS FAC OF MED UNIV SUMATERA UTARA IN CONJUNCTION WITH THE 23RD NATL CONGRESS OF THE INDONESIAN SOC OF TROP & INFECT DIS CONSULTANT AND THE 18TH ANNUAL MEETING OF INTERNAL MED DEPT FAC OF MED UNIV SUMATERA UTARA* 2018.

72. Lu MY, Chen CT, Shih YL, et al. Changing epidemiology and viral interplay of hepatitis B, C and D among injecting drug user-dominant prisoners in Taiwan. *Sci Rep* 2021.

73. Feng MC, Feng JY, Chen YH, Chang PY, Lu PL. Prevalence and knowledge of sexual transmitted infections, drug abuse, and AIDS among male inmates in a Taiwan prison. *Kaohsiung J Med Sci* 2012.

74. Lin CF, Twu SJ, Chen PH, Cheng JS, Wang JD. Prevalence and determinants of hepatitis B antigenemia in 15,007 inmates in Taiwan. *J Epidemiol* 2010.

75. Harnpariphan W, Han, W. M., Supanun R, Ubolyam S, et al. High Proportion of Blood-Borne and Sexually Transmitted Infections Among People Deprived of Liberty in a Central Male Prison in Thailand: A Cross-Sectional Study 2018-2019. *AIDS Research and Human Retroviruses* 2022.

76. Johns Hopkins University Bloomberg School of Public Health. Integrated Behavioral & Biological Surveillance (IBBS) in Afghanistan: Year 1 Report. *Johns Hopkins University Bloomberg School of Public Health* 2011.

77. Choudhury R, Singh N. Prevalence of HIV/AIDS in inmates of two district jails of central Uttar Pradesh, India. *Medico-Legal Update* 2016.

78. Organization NAC. HIV Sentinel Surveillance Plus 2019, Central Prison Sites, 2019.

79. National AIDS Control Organization. HIV Sentinel Surveillance Plus 2021, Central Prison Sites, 2022.

80. Ramamoorthy M, Venketeswaran A, Seenivasan P, et al. Risk factors and prevalence, hepatitis B virus and hepatitis C virus among prison inmates, Chennai, India, 2015. *International Journal of Infectious Diseases* 2016; **53**: 90.

81. National HIV bio­behavioral Surveillance Survey (BSS) in prisoners. 2009.

82. Moradi G, Jafari S, Zarei B, et al. Prevalence and Risk Factors for Hepatitis B and Hepatitis C Exposure in Iranian Prisoners: A National Study in 2016. *Hepatitis Monthly* 2019.

83. SeyedAlinaghi S, Farhoudi B, Shahmohamadi E, et al. Prevalence of and risk factors for HCV among incarcerated people at Great Tehran Prison: a cross-sectional study. *International journal of prisoner health* 2023.

84. Khajehkazemi R, Haghdoost A, Navadeh S, et al. Risk and vulnerability of key populations to HIV infection in Iran; Knowledge, attitude and practises of female sex workers, prison inmates and people who inject drugs. *Sexual Health* 2014.

85. Zamani S, Farnia M, Torknejad A, et al. Patterns of drug use and HIV-related risk behaviors among incarcerated people in a prison in Iran. *J Urban Health* 2010.

86. Mirzazadeh A, Shokoohi M, Navadeh S, et al. Underreporting in HIV-Related High-Risk Behaviors: Comparing the Results of Multiple Data Collection Methods in a Behavioral Survey of Prisoners in Iran. *PRISON JOURNAL* 2018.

87. Hariri S, Sharafkhah M, Alavi M, et al. A simple risk-based strategy for hepatitis C virus screening among incarcerated people in a low- to middle-income setting. *Harm Reduct J* 2020.

88. Khajedaluee M, Babaei A, Vakili R, et al. Sero-prevalence of bloodborne tumor viruses (HCV, HBV, HTLV-I and KSHV infections) and related risk factors among prisoners in Razavi Khorasan province, Iran, in 2008. *Hepatitis Monthly* 2016.

89. Ataei B, Khorvash F, Azadeh S, Nokhodian Z, Kassaian N, Babak A. The prevalence of high risk behaviors among women prisoners in Isfahan, Iran. *Journal of Isfahan Medical School* 2011.

90. Mamani M, Mahmudian H, Majzoobi MM, Poorolajal J. Prevalence and incidence rates of latent tuberculous infection in a large prison in Iran. *Int J Tuberc Lung Dis* 2016.

91. Shahesmaeili A, Karamouzian M, Tavakoli F, et al. HIV prevalence and continuum of care among incarcerated people in Iran from 2010 to 2017. *Harm Reduct J* 2022.

92. Mohtasham-Amiri Z, Rezvani SM, Ashoori F, Behboodi M, Toosi H, Jafari-Shakib R. Seroprevalence of Hepatitis C Virus among Prisoners in Lakan Prison, North of Iran, Is There Still a Concern? *Arch Iran Med* 2021.

93. Hariri S, Alavi M, Roshandel G, et al. An intervention to increase hepatitis C virus diagnosis and treatment uptake among people in custody in Iran. *Int J Drug Policy* 2021.

94. Navadeh S, Mirzazadeh A, Gouya MM, Farnia M, Alasvand R, Haghdoost AA. HIV prevalence and related risk behaviours among prisoners in Iran: results of the national biobehavioural survey, 2009. *Sex Transm Infect* 2013.

95. Moradi G, Darvishi S, Asaadi L, et al. Patterns of Drug Use and Related Factors Among Prisoners in Iran: Results from the National Survey in 2015. *Journal of Primary Prevention* 2020.

96. Moradi G, Gouya MM, Zavareh FA, et al. Prevalence and risk factors for HBV and HCV in prisoners in Iran: a national bio-behavioural surveillance survey in 2015. *Tropical Medicine & International Health* 2018.

97. Nokhodian Z, Yazdani MR, Yaran M, et al. Prevalence and risk factors of HIV, syphilis, hepatitis B and C among female prisoners in Isfahan, Iran. *Hepatitis Monthly* 2012.

98. Seyedalinaghi SA, Farhoudi B, Mohraz M, et al. Prevalence and Associated Factors of HIV Infection among Male Prisoners in Tehran, Iran. *Archives of Iranian Medicine* 2017.

99. Khezri M, Sharifi H, Mirzazadeh A, et al. A National Study of Suicidal Ideation and Suicide Attempt Among Incarcerated People in Iran. *International Journal of Mental Health and Addiction* 2023.

100. Shrestha G, Mulmi R, Yadav DK, et al. Health needs and risky behaviours among inmates in the largest prison of eastern Nepal. *Int J Prison Health* 2018.

101. Shrestha G. Prevalence of hypertension among incarcerated males of jhumka regional prison, Eastern Nepal. *Journal of Hypertension* 2018.

102. Kazi AM, Shah SA, Jenkins CA, Shepherd BE, Vermund SH. Risk factors and prevalence of tuberculosis, human immunodeficiency virus, syphilis, hepatitis B virus, and hepatitis C virus among prisoners in Pakistan. *International Journal pf Infectious Diseases* 2010.

103. Memon AR, Shafique K, Memon A, Draz AU, Rauf MUA, Afsar S. Hepatitis B and C prevalence among the high risk groups of Pakistani population. A cross sectional study. *Archives of Public Health* 2012.

104. Butt A, Jafri W, Janjua N, Pasha O. Seroprevalence and risk factors for hepatitis C infection among male prisoners in Karachi, Pakistan. *American Journal of Gastroenterology* 2010.

105. Khan MD, Wali A, Fatima R, Yaqoob A, Aziz S. Prevalence and associated risk factors of HIV in prisons in Balochistan, Pakistan: A cross-sectional study. *F1000Research* 2019.

106. Niriella MA, Hapangama A, Luke H, Pathmeswaran A, Kuruppuarachchi K, de Silva HJ. Prevalence of hepatitis B and hepatitis C infections and their relationship to injectable drug use in a cohort of Sri Lankan prison inmates. *Ceylon Medical Journal* 2015.

107. Moller LF, van den Bergh BJ, Karymbaeva S, Esenamanova A, Muratalieva R. Drug use in prisons in Kyrgyzstan: a study about the effect of health promotion among prisoners. *Int J Prison Health* 2008.

108. Azbel L, Polonsky M, Wegman M, et al. Intersecting epidemics of HIV, HCV, and syphilis among soon-to-be released prisoners in Kyrgyzstan: Implications for prevention and treatment. *International Journal of Drug Policy* 2016.

109. Winetsky DE, Almukhamedov O, Pulatov D, Vezhnina N, Dooronbekova A, Zhussupov B. Prevalence, risk factors and social context of active pulmonary tuberculosis among prison inmates in Tajikistan. *PLoS One* 2014.

110. Pena-Orellana M, Hernandez-Viver A, Caraballo-Correa G, Albizu-Garcia CE. Prevalence of HCV risk behaviors among prison inmates: Tattooing and injection drug use. *Journal of Health Care for the Poor and Underserved* 2011.

111. Falquetto TC, Endringer DC, Andrade TU, Lenz D. Hepatitis c in prisoners and non-prisoners in Colatina, Espirito santo, Brazil. *Brazilian Journal of Pharmaceutical Sciences* 2013.

112. Lopes F, Latorre MR, Campos Pignatari AC, Buchalla CM. [HIV, HPV, and syphilis prevalence in a women's penitentiary in the city of São Paulo, 1997-1998]. *Cad Saude Publica* 2001.

113. Rosa F, Carneiro M, Duro LN, et al. Prevalence of anti-HCV in an inmate population. *Rev Assoc Med Bras (1992)* 2012.

114. Pompilio MA, Pontes ERJC, Castro ARCM, et al. Prevalence and epidemiology of chronic hepatitis c among prisoners of Mato Grosso do Sul State, Brazil. *Journal of Venomous Animals and Toxins Including Tropical Diseases* 2011.

115. Guimarães T, Granato CF, Varella D, Ferraz ML, Castelo A, Kallás EG. High prevalence of hepatitis C infection in a Brazilian prison: identification of risk factors for infection. *The Brazilian Journal of Infectious Diseases* 2001.

116. Strazza L, Massad E, Azevedo RS, Carvalho HB. Behavior associated with HIV and HCV infection in female prison inmates in Sao Paulo, Brazil. *Cadernos de Saude Publica* 2007.

117. de Albuquerque ACC, da Silva DM, Rabelo DCC, et al. Seroprevalence and factors associated with human immunodeficiency virus (HIV) and syphilis in inmates in the state of Pernambuco, Brazil. *Ciencia & saude coletiva* 2014.

118. Miranda AE, Vargas PM, St Louis ME, Viana MC. Sexually transmitted diseases among female prisoners in Brazil - Prevalence and risk factors. *Sexually Transmitted Diseases* 2000.

119. Felisberto M, Saretto AA, Wopereis S, Machado MJ, Spada C. Prevalence of HCV infection in a prison population of the greater Florianopolis area. *Revista da Sociedade Brasileira de Medicina Tropical* 2019.

120. El Maerrawi I, Carvalho HB. Prevalence and risk factors associated with HIV infection, hepatitis and syphilis in a state prison of São Paulo. *Int J STD AIDS* 2015.

121. Coelho HC, de Oliveira SAN, Miguel JC, et al. Predictive markers for hepatitis C virus infection among Brazilian inmates. *Revista da Sociedade Brasileira de Medicina Tropical* 2009.

122. Massad E, Rozman M, Azevedo R, et al. Seroprevalence of HIV, HCV and syphilis in Brazilian prisoners: preponderance of parenteral transmission. *European journal of epidemiology* 1999; **15**: 439-45.

123. Felisberto M, Saretto AA, Wopereis S, Treitinger A, Machado MJ, Spada C. Prevalence of human immunodeficiency virus infection and associated risk factors among prison inmates in the city of Florianopolis. *Revista da Sociedade Brasileira de Medicina Tropical* 2016.

124. Santos BFO, de Santana NO, Franca AVC. Prevalence, genotypes and factors associated with HCV infection among prisoners in Northeastern Brazil. *World Journal of Gastroenterology* 2011.

125. Strazza L, Azevedo RS, Carvalho HB, Massad E. The vulnerability of Brazilian female prisoners to HIV infection. *Braz J Med Biol Res* 2004.

126. Sanchez-Vanegas G, Rodriguez-Vallejo D, Pinzon-Duran AC, Reina-Cifuentes MA, Monterrosa-Blanco A, Tiga-Segura JA. Prevalence of syphilis, hepatitis B and human immunodeficiency virus in the male prison population in Bogota, Colombia in 2019. [Spanish]. *Infectio* 2020.

127. Castillo RL, Noriega KJR, Briceno ML, Munoz NG, Pacheco JR. CONSUMPTION OF PSYCHOACTIVE SUBSTANCES BEFORE AND AFTER ADMISSION TO PRISON IN FOURTEEN PRISONS IN COLOMBIA. *REVISTA COLOMBIANA DE CIENCIAS SOCIALES* 2017.

128. Alvarez Rodriguez BE, Pinzon Z, Huaman BJ, et al. Prevalence of HIV, syphilis, drugs use and sexual risk behaviours among prisoners in Guatemala, 2012. *Sexually Transmitted Infections Conference: STI and AIDS World Congress* 2013.

129. Belaunzaran-Zamudio PF, Mosqueda-Gomez JL, Macias-Hernandez A, Sierra-Madero JG, Ahmed S, Beyrer C. Risk factors for prevalent hepatitis C virus-infection among inmates in a state prison system in Mexico. *PLoS One* 2017.

130. Bautista-Arredondo S, González A, Servan-Mori E, et al. A Cross-Sectional Study of Prisoners in Mexico City Comparing Prevalence of Transmissible Infections and Chronic Diseases with That in the General Population. *PLoS One* 2015.

131. Alvarado-Esquivel C, Sablon E, Martínez-García S, Estrada-Martínez S. Hepatitis virus and HIV infections in inmates of a state correctional facility in Mexico. *Epidemiology and Infection* 2005.

132. Gonzalez CAM, Ortiz BES, Aguilar MB, Gonzalez JDM. Risk factors and the seroprevalence of viral markers of hepatitis B (HVB) and hepatitis C (HCV) in high-risk groups in Chiapas. *Medwave* 2011.

133. Monsalve-Castillo F, Chacín-Bonilla L, Atencio RJ, et al. Low prevalence of hepatitis C virus infection in a prisoner population from Maracaibo, Venezuela. *Biomedica* 2009.

134. Courtemanche Y, Poulin C, Serhir B, Alary M. HIV and hepatitis C virus infections in Quebec's provincial detention centres: comparing prevalence and related risky behaviours between 2003 and 2014-2015. *Can J Public Health* 2018.

135. Calzavara LM, Burchell AN, Schlossberg J, et al. Prior opiate injection and incarceration history predict injection drug use among inmates. *Addiction* 2003.

136. Martin RE, Remple V, Gold F, Berkowitz J, Murphy W, Money D. Drug use and risk of bloodborne infections: A survey of female prisoners in British Columbia. *CANADIAN JOURNAL OF PUBLIC HEALTH-REVUE CANADIENNE DE SANTE PUBLIQUE* 2005.

137. Dussault C, Cox J, Klein M, Sebastiani G, Lebouche B, Kronfli N. Factors associated with on-demand HCV screening among Canadian provincial inmates. *Canadian Liver Journal* 2020.

138. Poulin C, Alary M, Lambert G, et al. Prevalence of HIV and hepatitis C virus infections among inmates of Quebec provincial prisons. [References]. *Canadian Medical Association Journal* 2007.

139. Besney JD, Angel C, Pyne D, Martell R, Keenan L, Ahmed R. Addressing Women’s Unmet Health Care Needs in a Canadian Remand Center. *Journal of Correctional Health Care* 2018.

140. Calzavara L, Ramuscak N, Burchell AN, et al. Prevalence of HIV and hepatitis C virus infections among inmates of Ontario remand facilities. *Cmaj* 2007.

141. Nolan AM, Stewart LA. Chronic Health Conditions Among Incoming Canadian Federally Sentenced Women. *J Correct Health Care* 2017.

142. Bonnycastle KD, Villebrun C. Injecting Risk Into Prison Sentences: A Quantitative Analysis of a Prisoner-Driven Survey to Measure HCV/HIV Seroprevalence, Risk Practices, and Viral Testing at One Canadian Male Federal Prison. *PRISON JOURNAL* 2011.

143. Begier EM, Bennani Y, Forgione L, et al. Undiagnosed HIV infection among New York City jail entrants, 2006: results of a blinded serosurvey. *J Acquir Immune Defic Syndr* 2010.

144. Kendrick SR, Kroc KA, Couture E, Weinstein RA. Comparison of point-of-care rapid HIV testing in three clinical venues. *Aids* 2004.

145. Swartz JA, Lurigio AJ, Weiner DA. CORRELATES OF HIV-RISK BEHAVIORS AMONG PRISON INMATES: IMPLICATIONS FOR TAILORED AIDS PREVENTION PROGRAMMING. *PRISON JOURNAL* 2004.

146. Khan AJ, Simard EP, Bower WA, et al. Ongoing transmission of hepatitis B virus infection among inmates at a state correctional facility. *American Journal of Public Health* 2005.

147. Mullings JL, Marquart JW, Hartley DJ. Exploring the effects of childhood sexual abuse and its impact on HIV/AIDS risk-taking behavior among women prisoners. *PRISON JOURNAL* 2003.

148. Kim AY, Nagami EH, Birch CE, Bowen MJ, Lauer GM, McGovern BH. A simple strategy to identify acute hepatitis C virus infection among newly incarcerated injection drug users. *Hepatology* 2013.

149. Trevino S. The relationship between age of first reported trauma and substance specific use in incarcerated women. *Dissertation Abstracts International: Section B: The Sciences and Engineering* 2013.

150. Akiyama MJ, Kaba F, Rosner Z, et al. Correlates of hepatitis C virus infection in the targeted testing program of the New York city jail system: Epidemiologic patterns and priorities for action. *Public Health Reports* 2017.

151. Adams LM, Kendall S, Smith A, Quigley E, Stuewig JB, Tangney JP. HIV risk behaviors of male and female jail inmates prior to incarceration and one year post-release. *AIDS Behav* 2013.

152. Alvarez KJ, Befus M, Herzig CTA, Larson E. Prevalence and correlates of hepatitis C virus infection among inmates at two New York State correctional facilities. *Journal of Infection and Public Health* 2014.

153. Abiona TC, Adefuye AS, Balogun JA, Sloan PE. Gender differences in HIV risk behaviors of inmates. *J Womens Health (Larchmt)* 2009.

154. Brinkley-Rubinstein L, Crowley C, Montgomery MC, et al. Interest and Knowledge of HIV Pre-Exposure Prophylaxis in a Unified Jail and Prison Setting. *Journal of Correctional Health Care* 2020.

155. MacGowan R, Margolis A, Richardson-Moore A, et al. Voluntary Rapid Human Immunodeficiency Virus (HIV) Testing in Jails. *Sexually Transmitted Diseases* 2009.

156. Lally M, Gaitanis M, Vallabhaneni S, et al. Willingness to receive an HIV vaccine among incarcerated persons. *Prev Med* 2006.

157. Abiona TC, Balogun JA, Adefuye AS, Sloan PE. Pre-incarceration HIV risk behaviours of male and female inmates. [References]. *International journal of prisoner health* 2009.

158. Tartaro C, Levy MP. An Evaluation of an HIV Testing Program in the Jail Setting: Results and Recommendations. *PRISON JOURNAL* 2013.

159. Keleekai NL. Patterns and predictors of HIV, sexually transmitted infections, and staphylococcus aureus co-infection among New York state prison inmates. *Dissertation Abstracts International: Section B: The Sciences and Engineering* 2012.

160. Beckwith CG, Liu T, Bazerman LB, et al. HIV risk behavior before and after HIV counseling and testing in jail: a pilot study. *J Acquir Immune Defic Syndr* 2010.

161. Nijhawan AE, Iroh PA, Brown LS, Winetsky D, Porsa E. Cost analysis of tuberculin skin test and the QuantiFERON-TB Gold In-tube test for tuberculosis screening in a correctional setting in Dallas, Texas, USA. *BMC Infect Dis* 2016.

162. McClelland GM, Teplin LA, Abram KM, Jacobs N. HIV and AIDS risk behaviors among female jail detainees: Implications for public heath policy. [References]. *American journal of public health* 2002.

163. Harrison LD, Bachman T, Freeman C, Inciardi JA. The acceptability of the female condom among US women at high risk from HIV. [References]. *Culture, health & sexuality* 2001.

164. Beckwith CG, Atunah-Jay S, Cohen J, et al. Feasibility and Acceptability of Rapid HIV Testing in Jail. [References]. *AIDS Patient Care and STDs* 2007.

165. Simonsen KA, Shaikh RA, Earley M, et al. Rapid HIV Screening in an Urban Jail: How Testing at Exit With Linkage to Community Care Can Address Perceived Barriers. *J Prim Prev* 2015.

166. Arndt S, Turvey CL, Flaum M. Older offenders, substance abuse, and treatment. *American Journal of Geriatric Psychiatry* 2002.

167. Deb LC, Hove H, Miller TK, et al. Epidemiology of Hepatitis C virus infection among incarcerated populations in North Dakota. *PLoS One* 2022.

168. Macalino GE, Dhawan D, Rich JD. A missed opportunity: hepatitis C screening of prisoners. *Am J Public Health* 2005.

169. Mullings JL, Marquart JW, Diamond PM. Cumulative continuity and injection drug use among women: A test of the downward spiral framework. [References]. *Deviant Behavior* 2001.

170. Katyal M, Leibowitz R, Venters H. IGRA-Based Screening for Latent Tuberculosis Infection in Persons Newly Incarcerated in New York City Jails. *J Correct Health Care* 2018.

171. Fox RK, Currie SL, Evans J, et al. Hepatitis C virus infection among prisoners in the California State correctional system. *Clinical Infectious Diseases* 2005.

172. Rice DK. Design, implementation, and evaluation of a jail-based HIV screening program. *Dissertation Abstracts International: Section B: The Sciences and Engineering* 2011.

173. Gates ML, Turney A, Ferguson E, Walker V, Staples-Horne M. Associations among Substance Use, Mental Health Disorders, and Self-Harm in a Prison Population: Examining Group Risk for Suicide Attempt. *Int J Environ Res Public Health* 2017.

174. Altice FL, Marinovich A, Khoshnood K, Blankenship KM, Springer SA, Selwyn PA. Correlates of HIV infection among incarcerated women: implications for improving detection of HIV infection. *J Urban Health* 2005.

175. Wenger PJ, Rottnek F, Parker T, Crippin JS. Assessment of hepatitis C risk factors and infection prevalence in a jail population. *American journal of public health* 2014; **104**(9): 1722-7.

176. Bah R, Sheehan Y, Li X, et al. Prevalence of blood-borne virus infections and uptake of hepatitis C testing and treatment in Australian prisons: the AusHep study. *The Lancet Regional Health–Western Pacific* 2024; **53**.

177. Butler TS, M. National Prison Entrants’ Bloodborne Virus and Risk Behaviour Survey Report: Kirby Institute 2017.

178. Indig D, Topp L, Ross B, et al. 2009 NSW Inmate Health Survey: Key Findings Report. Sydney: Justice Health, 2010.

179. Loxley W. Drug use, intoxication and offence type in two groups of alleged offenders in Perth: A pilot study. *Australian and New Zealand Journal of Criminology* 2001.

180. Hellard ME, Hocking JS, Crofts N. The prevalence and the risk behaviours associated with the transmission of hepatitis C virus in Australian correctional facilities. *Epidemiol Infect* 2004.

181. Kinner SA. The post-release experience of prisoners in Queensland. 2006.

182. Hajarizadeh B, Grebely J, Byrne M, et al. Evaluation of hepatitis C treatment-as-prevention within Australian prisons (SToP-C): a prospective cohort study. *LANCET GASTROENTEROLOGY & HEPATOLOGY* 2021.

183. Nicholson J, Almond L, Rizvi N, Fairley CK. Low prevalence of STIs among women in prison, but bacterial vaginosis is common. *Australian and New Zealand journal of public health* 2003.

184. Gilles M, Swingler E, Craven C, Larson A. Prison health and public health responses at a regional prison in Western Australia. *Australia and New Zealand Journal of Public Health* 2008.

185. Kevin M. Drug Use in the Inmate Population–prevalence, nature and context. *DUIP NSW–6th Biennial data collection 2009‑10: Overview and series trend* 2013.

186. Reekie JM, Levy MH, Richards AH, et al. Trends in prevalence of HIV infection, hepatitis B and hepatitis C among Australian prisoners - 2004, 2007, 2010. *Medical Journal of Australia* 2014.

187. Keen C, Kinner SA, Borschmann R, Young JT. Comparing the predictive capability of self-report and medically-verified non-fatal overdose in adults released from prison: A prospective data linkage study. *Drug and Alcohol Dependence* 2020.

188. Butler T, Levy M, Dolan K, Kaldor J. Drug use and its correlates in an Australian prisoner population. *Addiction Research & Theory* 2003.

189. Larney S, Monkley DL, Indig D, Hampton SE. A cross-sectional study of susceptibility to vaccine-preventable diseases among prison entrants in New South Wales. *Med J Aust* 2013.

190. Hockings BA, Young, M., Falconer, A., and O'Rourke, P.K. Queensland Women Prisoners' Health Survey. Brisbane: Department of Corrective Services, 2002.

191. Welfare AIoHa. The health of Australia’s prisoners 2018. Canberra: AIHW, 2019.

192. Hessou S, Dougnon VT, Glele-Ahanhanzo Y, et al. A behavioral and serological survey on HIV prevalence among prisoners in Benin. *Journal of Public Health in Africa* 2017.

193. Catraye DJ, Ky-Ba, A & Tavi-Ouattarra, A.Y. ENQUETE BIO COMPORTEMENTALE DU VIH-SIDA EN MILIEU CARCERAL AUPRES DES DETENUS HOMMES ET FEMMES AU BURKINA FASO. 2017.

194. Kowo MP, Andoulo FA, Sizimboue DT, et al. Seroprevalence of hepatitis B and associated factors among inmates: a cross sectional study in the Douala New Bell Prison, Cameroon. *Pan African Medical Journal* 2021.

195. Kassa Y, Million Y, Biset S, Moges F. Hepatitis b and hepatitis c viral infections and associated factors among prisoners in northeast ethiopia. *Journal of Blood Medicine* 2021.

196. Adjei AA, Armah HB, Gbagbo F, et al. Correlates of HIV, HBV, HCV and syphilis infections among prison inmates and officers in Ghana: A national multicenter study. *BMC Infectious Diseases* 2008.

197. Commission GA. National Health and HIV Survey of Prison Inmates in Ghana. 2013.

198. Akeke VA, Mokgatle M, Oguntibeju OO. Prevalence of risk factors for transmission of HIV and blood-borne viruses in a prison population. *AFRICAN JOURNAL OF MICROBIOLOGY RESEARCH* 2014.

199. Adoga MP, Banwat EB, Forbi JC, et al. Human immunonodeficiency virus, hepatitis B virus and hepatitis C virus: sero-prevalence, co-infection and risk factors among prison inmates in Nasarawa State, Nigeria. *The Journal of Infection in Developing Countries* 2009.

200. Okafor IM, Ugwu SO, Okoroiwu HU. Hepatitis C virus infection and its associated factors among prisoners in a Nigerian prison. *BMC Gastroenterol* 2020.

201. AIDS NCftFa. BEHAVIORAL SURVEY COUPLED TO HIV SEROLOGY AMONG PROFESSIONALS SEX, MEN HAVING SEX SEX WITH MEN AND INMATES IN REPUBLIC OF CONGO FINAL REPORT. In: AIDS NCftFa, editor.; 2012.

202. Telisinghe L, Fielding KL, Malden JL, et al. High tuberculosis prevalence in a South African prison: the need for routine tuberculosis screening. *PLoS One* 2014.

203. Ekouevi DK, D'Almeida S, Salou M, et al. HIV seroprevalence among inmates in Togo. *Med Mal Infect* 2013.

204. European Union Drug Agency. EUDA Prison Data 2024. Lisbon; 2024.

205. Mahfoud Z, Kassak K, Kreidieh K, Shamra S, Ramia S. Prevalence of antibodies to human immunodeficiency virus (HIV), hepatitis B and hepatitis C and risk factors in prisoners in Lebanon. *The Journal of Infection in Developing Countries* 2010.

206. Ministry of Health Lebanon. AN INTEGRATED BIO-BEHAVIORAL SURVEILLANCE STUDY AMONG MOST AT RISK POPULATIONS IN LEBANON: FEMALE SEX WORKERS, INJECTING DRUG USERS, MEN WHO HAVE SEX WITH MEN, AND PRISONERS. 2008.

207. Sahin AR, Sahin AM, Gunduz A, Aktemur A, Kes-Uzun N. HIV seropositivity in a penal institution in Turkey: A cross-sectional study. [Turkish]. *Klimik Dergisi* 2018.

208. Keten D, Ova ME, Keten HS, et al. The prevalence of hepatitis B and C among prisoners in Kahramanmaras, Turkey. *Jundishapur Journal of Microbiology* 2016.
