# Supplementary material for: The global epidemiology of injecting drug use, HIV, viral hepatitis and tuberculosis among people who are incarcerated: a multistage systematic review
Source: Int J Drug Policy. Author manuscript; Available in PMC 2026 Apr 8. (PMC13058553; doi:10.1016/j.drugpo.2025.105062)
Supplement: 2 [file NIHMS2157186-supplement-2.docx]

## Appendix 15.2: Study Characteristics of included papers for HIV estimates

| **Country** | **Author** | **Year of Publication** | **Geographic area** | **Female data** | **Male data** | **Mixed data** | **Risk of Bias Total Score** | **Reference** |
| --- | --- | --- | --- | --- | --- | --- | --- | --- |
| **Eastern Europe** |  |  |  |  |  |  |  |  |
| Armenia | Weilandt | 2007 | National | No | Yes | No | 7 | ^1^ |
| Azerbaijan | Kasumov | 2008 | City | No | Yes | No | 5 | ^2^ |
| Azerbaijan | Azbel | 2015 | National | Yes | Yes | No | 7 | ^3^ |
| Azerbaijan | Čakalo | 2012 | National | No | Yes | No | 7 | ^4^ |
| Azerbaijan | Handanagic | 2015 | City | No | Yes | No | 6 | ^5^ |
| Bosnia & Herzegovina | Ravlija | 2014 | National | No | No | Yes | 6 | ^6^ |
| Bulgaria | Popov | 2011 | Facility | No | No | Yes | 5 | ^7^ |
| Bulgaria | Panayotov | 2022 | National | No | No | Yes | 5 | ^8^ |
| Estonia | Kivimets | 2014 | National | Yes | Yes | No | 6 | ^9^ |
| Hungary | Vanya | 2017 | Subnational | No | No | Yes | 6 | ^10^ |
| Hungary | Treso | 2012 | National | No | No | Yes | 6 | ^11^ |
| Ukraine | Azbel | 2013 | National | Yes | Yes | No | 7 | ^12^ |
| Ukraine | Balakireva | 2012 | Subnational | Yes | Yes | No | 8 | ^13^ |
| **Western Europe** |  |  |  |  |  |  |  |  |
| Austria | Silbernagl | 2018 | Subnational | No | Yes | No | 5 | ^14^ |
| Austria | Silbernagl | 2018 | Subnational | No | Yes | No | 5 | ^14^ |
| Belgium | Todts | 2008 | National | No | No | Yes | 5 | ^15^ |
| Belgium | Busschotts | 2021 | Subnational | No | No | Yes | 6 | ^16^ |
| Belgium | Todts | 2008 | National | No | No | Yes | 5 | ^15^ |
| Croatia | Burek | 2009 | National | Yes | Yes | No | 6 | ^17^ |
| Croatia | Vilibic-Cavlek | 2011 | Subnational | No | Yes | No | 6 | ^18^ |
| Denmark | Christensen | 2000 | Facility | No | Yes | No | 5 | ^19^ |
| England and Wales | Morey | 2018 | Facility | No | No | Yes | 5 | ^20^ |
| England and Wales | Mahto | 2008 | Facility | Yes | No | No | 5 | ^21^ |
| England and Wales | Aisyah | 2017 | City | No | No | Yes | 5 | ^22^ |
| England and Wales | Weild | 2000 | Multinational | Yes | Yes | No | 6 | ^23^ |
| Finland | Rautanen | 2023 | National | Yes | Yes | No | 5 | ^24^ |
| Finland | Viitanen | 2011 | Facility | Yes | Yes | No | 5 | ^25^ |
| Finland | Viitanen | 2011 | Subnational | Yes | Yes | No | 7 | ^25^ |
| France | Jacomet | 2016 | Subnational | No | No | Yes | 7 | ^26^ |
| France | Izquierdo | 2019 | Facility | No | No | Yes | 6 | ^27^ |
| France | Semaille | 2013 | National | No | No | Yes | 5 | ^28^ |
| France | Reynaud-Maurupt | 2005 | Subnational | No | No | Yes | 5 | ^29^ |
| France | Lelievre | 2020 | Facility | No | Yes | No | 5 | ^30^ |
| France | Rotily | 2000 | Facility | No | Yes | No | 6 | ^31^ |
| France | Perrodeau | 2016 | Facility | No | No | Yes | 4 | ^32^ |
| France | Abel | 2018 | Facility | No | No | Yes | 4 | ^33^ |
| France | Perrodeau | 2016 | Facility | No | No | Yes | 4 | ^32^ |
| Germany | Schulte | 2009 | National | No | No | Yes | 6 | ^34^ |
| Germany | Rotily | 2000 | Facility | No | Yes | No | 6 | ^31^ |
| Germany | Lehmann | 2004 | Facility | No | Yes | No | 5 | ^35^ |
| Greece | Fotiadou | 2004 | Facility | No | Yes | No | 4 | ^36^ |
| Ireland | Wright | 2006 | National | Yes | No | No | 6 | ^37^ |
| Ireland | NACDA | 2011 | National | No | No | Yes | 5 | ^38^ |
| Ireland | Bannan | 2016 | Facility | No | Yes | Yes | 4 | ^39^ |
| Ireland | Crowley | 2019 | Facility | No | Yes | No | 6 | ^40^ |
| Ireland | Allwright | 2000 | National | Yes | Yes | No | 7 | ^41^ |
| Ireland | Bannan | 2016 | Facility | No | Yes | Yes | 5 | ^39^ |
| Ireland | Drummond | 2014 | National | Yes | Yes | No | 6 | ^42^ |
| Ireland | Drummond | 2014 | National | Yes | Yes | No | 6 | ^42^ |
| Ireland | Bannan | 2016 | Facility | No | Yes | Yes | 5 | ^39^ |
| Ireland | Wright | 2006 | National | Yes | No | No | 6 | ^37^ |
| Italy | Stasi | 2016 | Subnational | No | No | Yes | 7 | ^43^ |
| Italy | Babudieri | 2005 | National | Yes | Yes | No | 7 | ^44^ |
| Italy | Sagnelli | 2012 | National | No | No | Yes | 5 | ^45^ |
| Italy | Giuliani | 2020 | City | No | No | Yes | 4 | ^46^ |
| Italy | Ranieri | 2015 | Facility | No | No | Yes | 5 | ^47^ |
| Italy | Giuliani | 2020 | City | No | No | Yes | 4 | ^46^ |
| Italy | Rotily | 2000 | Facility | No | Yes | No | 6 | ^31^ |
| Italy | Ranieri | 2015 | Facility | No | No | Yes | 5 | ^47^ |
| Italy | Monarca | 2015 | National | No | No | Yes | 5 | ^48^ |
| Italy | Voller | 2011 | Subnational | No | Yes | No | 6 | ^49^ |
| Italy | Ciccarese | 2020 | Facility | No | Yes | No | 6 | ^50^ |
| Malta | Muscat | 2022 | Facility | No | No | Yes | 4 | ^51^ |
| Netherlands | Rotily | 2000 | Facility | No | Yes | No | 4 | ^31^ |
| North Macedonia | Jovanovska | 2014 | Subnational | No | No | Yes | 6 | ^52^ |
| Northern Ireland | Danis | 2007 | Subnational | No | No | Yes | 6 | ^53^ |
| Portugal | da Silva Marques | 2010 | Facility | No | Yes | No | 5 | ^54^ |
| Portugal | Passadouro | 2004 | Facility | Yes | Yes | No | 6 | ^55^ |
| Portugal | Garcia | 2004 | Facility | Yes | No | No | 5 | ^56^ |
| Portugal | Barros | 2008 | Facility | Yes | No | No | 5 | ^57^ |
| Scotland | Peters | 2016 | Facility | No | No | Yes | 5 | ^58^ |
| Scotland | Rotily | 2000 | Facility | No | Yes | No | 7 | ^59^ |
| Spain | Gonzalez | 2008 | Facility | Yes | No | No | 6 | ^60^ |
| Spain | García-Guerrero | 2010 | National | No | No | Yes | 7 | ^61^ |
| Spain | Marco | 2012 | Subnational | No | No | Yes | 7 | ^62^ |
| Spain | Olivan | 2001 | Facility | No | No | Yes | 6 | ^63^ |
| Spain | Vicente-Alcalde | 2020 | Subnational | No | No | Yes | 6 | ^64^ |
| Spain | Ferrer-Castro | 2012 | Facility | No | No | Yes | 5 | ^65^ |
| Spain | Murcia | 2009 | Facility | No | No | Yes | 6 | ^66^ |
| Spain | Cuadrado | 2018 | Facility | No | No | Yes | 6 | ^67^ |
| Spain | Marcos | 2022 | Facility | No | Yes | No | 5 | ^68^ |
| Spain | Serroukh | 2022 | Facility | Yes | Yes | No | 5 | ^69^ |
| Spain | Mourino | 2013 | City | No | Yes | No | 5 | ^70^ |
| Spain | Martin | 2001 | Facility | No | No | Yes | 5 | ^71^ |
| Sweden | Rotily | 2000 | Facility | No | Yes | No | 4 | ^31^ |
| Sweden | Gahrton | 2019 | Subnational | Yes | Yes | No | 6 | ^72^ |
| Switzerland | Pala | 2018 | Facility | No | Yes | No | 6 | ^73^ |
| Switzerland | Moschetti | 2015 | Subnational | Yes | Yes | No | 7 | ^74^ |
| **East and South East Asia** |  |  |  |  |  |  |  |  |
| Indonesia | Blogg | 2014 | National | Yes | Yes | No | 6 | ^75^ |
| Indonesia | Ministry of Health Republic of Indonesia | 2011 | National | No | No | Yes | 6 | ^76^ |
| Indonesia | Arends | 2019 | Facility | Yes | No | No | 5 | ^77^ |
| Indonesia | Sembiring | 2018 | Facility | No | No | Yes | 5 | ^78^ |
| Indonesia | Rey | 2018 | Facility | No | No | Yes | 4 | ^79^ |
| Myanmar | Mwe Nom | 2020 | Facility | Yes | Yes | No | 4 | ^80^ |
| Philippines | Simbulan | 2001 | Facility | Yes | No | No | 4 | ^81^ |
| South Korea | Choi | 2021 | National | No | No | Yes | 6 | ^82^ |
| Taiwan | Lu | 2021 | Facility | Yes | Yes | No | 6 | ^83^ |
| Thailand | Morasert | 2018 | Facility | No | No | Yes | 5 | ^84^ |
| **South Asia** |  |  |  |  |  |  |  |  |
| Afghanistan | John Hopkins University | 2012 | Facility | No | No | Yes | 5 | ^85^ |
| Afghanistan | John Hopkins University | 2011 | Facility | No | No | Yes | 5 | ^86^ |
| Afghanistan | John Hopkins University | 2012 | Facility | No | No | Yes | 5 | ^85^ |
| Afghanistan | John Hopkins University | 2011 | Facility | No | No | Yes | 5 | ^86^ |
| India | Kosambiya | 2022 | Facility | No | No | Yes | 6 | ^87^ |
| India | Sabharwal | 2012 | Facility | No | No | Yes | 5 | ^88^ |
| India | Choudhury | 2016 | Subnational | Yes | Yes | No | 5 | ^89^ |
| India | Tyagi | 2018 | Facility | No | No | Yes | 5 | ^90^ |
| India | National AIDS Control Organization | 2019 | National | No | Yes | No | 7 | ^91^ |
| India | National AIDS Control Organization | 2022 | National | No | Yes | No | 7 | ^92^ |
| Iran (Islamic Republic of) | Ziaee | 2014 | Subnational | No | No | Yes | 7 | ^93^ |
| Iran (Islamic Republic of) | Haghdoost | 2013 | National | No | No | Yes | 6 | ^94^ |
| Iran (Islamic Republic of) | Shahesmaeili | 2022 | National | Yes | Yes | No | 5 | ^95^ |
| Iran (Islamic Republic of) | Shahesmaeili | 2022 | National | Yes | Yes | No | 6 | ^95^ |
| Iran (Islamic Republic of) | Khademi | 2019 | Subnational | No | Yes | No | 7 | ^96^ |
| Iran (Islamic Republic of) | Shahesmaeili | 2022 | National | Yes | Yes | No | 6 | ^95^ |
| Iran (Islamic Republic of) | Nokhodian | 2012 | Facility | Yes | No | No | 5 | ^97^ |
| Iran (Islamic Republic of) | SeyedAlinaghi | 2017 | Facility | No | Yes | No | 4 | ^98^ |
| Iran (Islamic Republic of) | Shahbazi | 2014 | National | No | No | Yes | 5 | ^99^ |
| Pakistan | Wali | 2019 | Facility | No | No | Yes | 6 | ^100^ |
| Pakistan | Nafees | 2011 | City | Yes | Yes | No | 6 | ^101^ |
| Pakistan | Kazi | 2010 | Facility | No | Yes | No | 6 | ^102^ |
| Pakistan | Pervaiz | 2012 | Subnational | Yes | Yes | No | 7 | ^103^ |
| Pakistan | Khan | 2019 | Facility | No | No | Yes | 4 | ^104^ |
| Pakistan | Safdar | 2009 | Subnational | No | No | Yes | 6 | ^105^ |
| Pakistan | Khan | 2019 | Subnational | No | No | Yes | 6 | ^104^ |
| Pakistan | Shah | 2013 | Facility | No | Yes | No | 5 | ^106^ |
| **Central Asia** |  |  |  |  |  |  |  |  |
| Kyrgyzstan | Azbel | 2016 | National | No | No | Yes | 7 | ^107^ |
| Tajikistan | Ministry of Health and Social Protection | 2020 |  | No | No | Yes | 5 | ^108^ |
| **Caribbean** |  |  |  |  |  |  |  |  |
| Antigua & Barbuda | Boisson | 2009 | Facility | No | Yes | No | 4 | ^109^ |
| Dominica | Boisson | 2009 | Facility | No | Yes | No | 4 | ^109^ |
| Grenada | Boisson | 2009 | Facility | No | Yes | No | 4 | ^109^ |
| Jamaica | Andrinopolous | 2010 | Facility | No | Yes | No | 5 | ^110^ |
| Saint Kitts & Nevis | Boisson | 2009 | Facility | No | Yes | No | 4 | ^109^ |
| Saint Lucia | Boisson | 2009 | Facility | No | Yes | No | 5 | ^109^ |
| Saint Vincent & the Grenadines | Boisson | 2009 | Facility | No | Yes | No | 5 | ^109^ |
| **Latin America** |  |  |  |  |  |  |  |  |
| Argentina | Adaszko | 2017 | National | Yes | Yes | No | 7 | ^111^ |
| Argentina | Mendizabal | 2020 | Subnational | No | No | Yes | 7 | ^112^ |
| Belize | Gough | 2009 | Facility | No | Yes | No | 6 | ^113^ |
| Bolivia (Plurinational State of) | Villarroel-Torrico | 2018 | Facility | Yes | No | No | 6 | ^114^ |
| Bolivia (Plurinational State of) | Lambert | 2005 | Subnational | Yes | Yes | No | 5 | ^115^ |
| Brazil | Vale | 2016 | Facility | Yes | Yes | No | 6 | ^116^ |
| Brazil | Machado | 2019 | Facility | No | No | Yes | 5 | ^117^ |
| Brazil | Lopes | 2001 | Facility | Yes | No | No | 5 | ^118^ |
| Brazil | Leal | 2022 | Subnational | Yes | No | No | 7 | ^119^ |
| Brazil | Pompilio | 2011 | Subnational | No | No | Yes | 6 | ^120^ |
| Brazil | Guimarães | 2001 | Facility | No | Yes | No | 6 | ^121^ |
| Brazil | Benedetti | 2020 | Subnational | Yes | No | No | 6 | ^122^ |
| Brazil | Strazza | 2007 | Facility | Yes | No | No | 6 | ^123^ |
| Brazil | de Albuquerque | 2013 | Facility | No | Yes | No | 6 | ^124^ |
| Brazil | Ronchi | 2017 | Facility | No | Yes | No | 5 | ^125^ |
| Brazil | Sgarbi | 2015 | Subnational | Yes | Yes | No | 7 | ^126^ |
| Brazil | Valenca | 2015 | Facility | No | No | Yes | 6 | ^127^ |
| Brazil | Ferreto | 2021 | Subnational | No | Yes | No | 7 | ^128^ |
| Brazil | dos Santos Bet | 2018 | Subnational | No | No | Yes | 7 | ^129^ |
| Brazil | Catalan-Soares | 2000 | City | No | Yes | No | 4 | ^130^ |
| Brazil | do Nascimento | 2020 | City | No | No | Yes | 5 | ^131^ |
| Brazil | El Maerrawi | 2015 | Facility | No | Yes | No | 6 | ^132^ |
| Brazil | Prellwitz | 2013 | Facility | No | No | Yes | 5 | ^133^ |
| Brazil | Massad | 1999 | Facility | No | No | Yes | 5 | ^134^ |
| Brazil | Leite | 2022 | Facility | No | Yes | No | 5 | ^135^ |
| Brazil | Soares | 2022 | Subnational | No | No | Yes | 6 | ^136^ |
| Brazil | Miranda | 2025 | Facility | Yes | No | No | 5 | ^137^ |
| Brazil | Coelho | 2007 | Facility | No | Yes | No | 6 | ^138^ |
| Brazil | Pelissari | 2018 | Facility | No | Yes | No | 4 | ^139^ |
| Brazil | Marins | 2000 | Subnational | No | Yes | No | 6 | ^140^ |
| Brazil | Moura | 2019 | Facility | No | No | Yes | 5 | ^141^ |
| Brazil | Sousa | 2017 | Subnational | Yes | Yes | No | 6 | ^142^ |
| Brazil | Felisberto | 2016 | Facility | No | Yes | No | 4 | ^143^ |
| Colombia | Sanchez-Vanegas | 2021 | Facility | No | Yes | No | 6 | ^144^ |
| Guatemala | Alvarez Rodriguez | 2013 | Subnational | No | No | Yes | 7 | ^145^ |
| Mexico | Belaunzaran-Zamudio | 2017 | Subnational | No | No | Yes | 5 | ^146^ |
| Mexico | Silverman-Retana | 2017 | City | No | No | Yes | 5 | ^147^ |
| Mexico | Bautista-Arredondo | 2015 | Subnational | Yes | Yes | Yes | 7 | ^148^ |
| Mexico | Bautista-Arredondo | 2015 | Subnational | Yes | Yes | Yes | 7 | ^148^ |
| Mexico | Alvarado-Esquivel | 2005 | Facility | No | No | Yes | 5 | ^149^ |
| Peru | Garaycochea | 2013 | Facility | Yes | No | No | 4 | ^150^ |
| Peru | Cyrus | 2021 | Facility | Yes | No | No | 6 | ^151^ |
| Uruguay | Troya | 2010 | Facility | No | Yes | No | 6 | ^152^ |
| Venezuela (Bolivarian Republic of) | Gil | 2022 | National | Yes | Yes | No | 6 | ^153^ |
| Venezuela (Bolivarian Republic of) | Tremarias | 2008 | National | No | No | Yes | 5 | ^154^ |
| Venezuela (Bolivarian Republic of) | Alcivar | 2020 | Facility | Yes | No | No | 5 | ^155^ |
| **North America** |  |  |  |  |  |  |  |  |
| Canada | Courtemanche | 2018 | Subnational | Yes | Yes | No | 4 | ^156^ |
| Canada | Poulin | 2007 | Subnational | No | Yes | No | 6 | ^157^ |
| Canada | Courtemanche | 2018 | Subnational | Yes | Yes | No | 6 | ^156^ |
| Canada | Calzavara | 2007 | Subnational | No | No | Yes | 7 | ^158^ |
| Canada | Ford | 2000 | Facility | No | Yes | No | 5 | ^159^ |
| Canada | Poulin | 2007 | Subnational | No | Yes | No | 7 | ^157^ |
| Canada | Calzavara | 2007 | Subnational | No | No | Yes | 6 | ^158^ |
| United States of America | Begier | 2010 | Subnational | Yes | Yes | No | 6 | ^160^ |
| United States of America | Kendrick | 2004 | Facility | Yes | No | No | 5 | ^161^ |
| United States of America | Bauserman | 2001 | Subnational | Yes | Yes | No | 7 | ^162^ |
| United States of America | Klein | 2002 | Subnational | No | No | Yes | 6 | ^163^ |
| United States of America | Kavasery | 2009 | Facility | No | Yes | No | 5 | ^164^ |
| United States of America | de Ravello | 2005 | Subnational | Yes | No | No | 7 | ^165^ |
| United States of America | Akiyama | 2017 | City | No | No | Yes | 6 | ^166^ |
| United States of America | Rowell-Cunsolo | 2016 | Subnational | No | No | Yes | 7 | ^167^ |
| United States of America | Smith | 2016 | Subnational | No | No | Yes | 6 | ^168^ |
| United States of America | Solomon | 2004 | Subnational | No | No | Yes | 6 | ^169^ |
| United States of America | Alvarez | 2014 | Facility | Yes | Yes | No | 6 | ^170^ |
| United States of America | de Voux | 2012 | Subnational | No | No | Yes | 8 | ^171^ |
| United States of America | MacGowan | 2009 | Subnational | Yes | Yes | No | 6 | ^172^ |
| United States of America | Krebs | 2006 | Subnational | No | Yes | No | 6 | ^173^ |
| United States of America | Sampson | 2008 | Subnational | Yes | Yes | No | 7 | ^174^ |
| United States of America | Tartaro | 2013 | Facility | No | No | Yes | 5 | ^175^ |
| United States of America | Sieck | 2011 | Facility | No | No | Yes | 5 | ^176^ |
| United States of America | Feld | 2023 | Subnational | Yes | Yes | No | 6 | ^177^ |
| United States of America | Cocoros | 2014 | Facility | No | No | Yes | 4 | ^178^ |
| United States of America | Strick | 2011 | Subnational | No | Yes | No | 6 | ^179^ |
| United States of America | Keleekai | 2011 | Facility | Yes | Yes | No | 6 | ^180^ |
| United States of America | Scott | 2011 | Subnational | Yes | Yes | Yes | 5 | ^181^ |
| United States of America | Leukefeld | 2002 | Subnational | No | Yes | No | 6 | ^182^ |
| United States of America | Javanbakht | 2014 | Facility | Yes | No | No | 5 | ^183^ |
| United States of America | Ruiz | 2002 | Subnational | Yes | Yes | No | 5 | ^184^ |
| United States of America | Nijhawan | 2016 | Facility | No | No | Yes | 6 | ^185^ |
| United States of America | Spaulding | 2015 | Facility | No | No | Yes | 5 | ^186^ |
| United States of America | Beckwith | 2012 | Subnational | No | No | Yes | 6 | ^187^ |
| United States of America | Weant | 2012 | Subnational | No | No | Yes | 4 | ^188^ |
| United States of America | Rosen | 2016 | Subnational | No | No | Yes | 6 | ^189^ |
| United States of America | Spaulding | 2011 | Facility | No | No | Yes | 6 | ^190^ |
| United States of America | Desai | 2021 | Facility | Yes | No | No | 5 | ^191^ |
| United States of America | Maruschak | 2023 | National | No | No | Yes | 6 | ^192^ |
| United States of America | Beckwith | 2011 | Subnational | No | No | Yes | 6 | ^193^ |
| United States of America | Wohl | 2013 | Subnational | No | No | Yes | 6 | ^194^ |
| United States of America | Bailargeon | 2003 | Subnational | Yes | Yes | No | 6 | ^195^ |
| United States of America | Ruiz | 2002 | Subnational | Yes | Yes | No | 5 | ^184^ |
| United States of America | Rosen | 2009 | Subnational | Yes | Yes | No | 6 | ^196^ |
| United States of America | Macalino | 2004 | Facility | No | Yes | No | 5 | ^197^ |
| United States of America | Carvajal | 2005 | Facility | Yes | No | No | 5 | ^198^ |
| United States of America | Baillargeon | 2017 | Subnational | Yes | Yes | No | 6 | ^199^ |
| United States of America | Kavasery | 2009 | Facility | Yes | No | No | 5 | ^200^ |
| United States of America | Harrison | 2001 | Facility | Yes | No | No | 5 | ^201^ |
| United States of America | Scott | 2011 | Subnational | Yes | Yes | Yes | 6 | ^181^ |
| United States of America | Spaulding | 2013 | National | No | No | Yes | 5 | ^202^ |
| United States of America | Seth | 2015 | National | Yes | Yes | No | 6 | ^203^ |
| United States of America | Peter | 2013 | Facility | Yes | Yes | No | 4 | ^204^ |
| United States of America | Alvarez | 2014 | Facility | Yes | Yes | No | 6 | ^170^ |
| United States of America | Spaulding | 2015 | Facility | No | No | Yes | 5 | ^186^ |
| United States of America | Beckwith | 2007 | Facility | No | Yes | No | 4 | ^205^ |
| United States of America | Simonsen | 2015 | Facility | No | No | Yes | 6 | ^206^ |
| United States of America | Arriola | 2001 | Subnational | Yes | Yes | No | 6 | ^207^ |
| United States of America | Chin | 2022 | Subnational | No | No | Yes | 6 | ^208^ |
| United States of America | Taussig | 2006 | Subnational | No | Yes | No | 7 | ^209^ |
| United States of America | Baillargeon | 2000 | Subnational | Yes | Yes | No | 6 | ^210^ |
| United States of America | Lucas | 2016 | Subnational | Yes | Yes | No | 5 | ^211^ |
| United States of America | Strick | 2011 | Subnational | No | Yes | No | 6 | ^179^ |
| United States of America | Strick | 2011 | Subnational | No | Yes | No | 6 | ^179^ |
| United States of America | Chin | 2021 | Subnational | No | No | Yes | 7 | ^212^ |
| United States of America | Keleekai | 2011 | Facility | Yes | Yes | No | 6 | ^180^ |
| United States of America | Katyal | 2018 | City | Yes | No | No | 6 | ^213^ |
| United States of America | Baillargeon | 2008 | Subnational | Yes | Yes | No | 6 | ^214^ |
| United States of America | de la Flor | 2017 | Facility | No | No | Yes | 5 | ^215^ |
| United States of America | Rice | 2010 | Facility | Yes | Yes | No | 5 | ^216^ |
| United States of America | Baillargeon | 2003 | Subnational | Yes | Yes | No | 6 | ^195^ |
| United States of America | Peter | 2013 | Facility | Yes | Yes | No | 4 | ^204^ |
| United States of America | Altice | 2005 | Facility | Yes | No | No | 6 | ^217^ |
| United States of America | Wenger | 2014 | Facility | No | No | Yes | 6 | ^218^ |
| **Pacific Island States & Terr·** |  |  |  |  |  |  |  |  |
| Fiji | Kinner | 2015 | Subnational | No | No | Yes | 5 | ^219^ |
| **Australasia** |  |  |  |  |  |  |  |  |
| Australia | Bah | 2024 | National | Yes | Yes | No | 7 | ^220^ |
| Australia | Indig | 2010 | Subnational | No | Yes | No | 7 | ^221^ |
| Australia | Butler | 2017 | National | Yes | Yes | No | 6 | ^222^ |
| Australia | Butler | 2007 | Subnational | Yes | Yes | No | 6 | ^223^ |
| Australia | Gilles | 2008 | Facility | No | No | Yes | 3 | ^224^ |
| Australia | Watkins | 2009 | Subnational | No | No | Yes | 5 | ^225^ |
| Australia | Justice Health | 2017 | Subnational | Yes | Yes | No | 7 | ^226^ |
| Australia | Reekie | 2014 | Subnational | No | No | Yes | 5 | ^227^ |
| Australia | Justice Health | 2017 | Subnational | Yes | Yes | No | 7 | ^226^ |
| Australia | Stoove | 2011 | Facility | No | No | Yes | 4 | ^228^ |
| Australia | Indig | 2010 | Subnational | No | Yes | No | 5 | ^221^ |
| New Zealand | Lythgoe | 2022 | National | No | No | Yes | 6 | ^229^ |
| **Sub Saharan Africa** |  |  |  |  |  |  |  |  |
| Benin | Hessou | 2017 | National | No | No | Yes | 6 | ^230^ |
| Burkina Faso | Bureau d’Appui en Santé Publique | 2017 | National | Yes | Yes | No | 7 | ^231^ |
| Burkina Faso | Diendere | 2011 | Facility | No | No | Yes | 6 | ^232^ |
| Burkina Faso | Ba | 2017 | National | No | No | Yes | 7 | ^233^ |
| Cameroon | Noeske | 2006 | Facility | No | No | Yes | 5 | ^234^ |
| Cote d'Ivoire | Angora |  | Facility | No | No | Yes | 5 | ^235^ |
| Cote d'Ivoire | Receveur | 2015 | Facility | Yes | Yes | No | 6 | ^236^ |
| Democratic Republic of the Congo | Kayomo | 2018 | Facility | No | No | Yes | 5 | ^237^ |
| Democratic Republic of the Congo | Mashako | 2012 | Facility | No | No | Yes | 5 | ^238^ |
| Ethiopia | Sahle | 2023 | Facility | Yes | Yes | No | 6 | ^239^ |
| Ethiopia | Sahle | 2023 | Facility | Yes | Yes | No | 6 | ^239^ |
| Ethiopia | Sahle | 2019 | Facility | No | No | Yes | 6 | ^240^ |
| Ethiopia | Kedebe | 2017 | Facility | No | No | Yes | 5 | ^241^ |
| Ethiopia | Sahle | 2023 | Facility | Yes | Yes | No | 6 | ^239^ |
| Ghana | Adjei | 2008 | National | Yes | Yes | No | 7 | ^242^ |
| Ghana | Ghana AIDS Commission | 2013 | National | Yes | Yes | No | 6 | ^243^ |
| Ghana | Adjei | 2006 | National | No | No | Yes | 6 | ^244^ |
| Ghana | Ghana AIDS Commission | 2013 |  | Yes | Yes | No | 5 | ^243^ |
| Kenya | Ministry of Health Kenya | 2012 | National | No | No | Yes | 6 | ^245^ |
| Liberia | Jones | 2019 | Subnational | No | No | Yes | 6 | ^246^ |
| Madagascar | Fenomanana | 2021 | Facility | No | No | Yes | 4 | ^247^ |
| Malawi | Mangochi | 2022 | Facility | No | No | Yes | 6 | ^248^ |
| Malawi | Banerjee | 2000 | Facility | No | No | Yes | 5 | ^249^ |
| Malawi | Chimphambano | 2007 | Facility | Yes | Yes | No | 6 | ^250^ |
| Malawi | Mallewa | 2023 | National | No | No | Yes | 6 | ^251^ |
| Nigeria | Chigbu | 2010 | Facility | No | Yes | No | 5 | ^252^ |
| Nigeria | Bashorun | 2015 | Facility | No | No | Yes | 4 | ^253^ |
| Nigeria | Muhammed | 2012 | National | Yes | Yes | No | 6 | ^254^ |
| Nigeria | Lawrence | 2021 | Facility | Yes | Yes | No | 5 | ^255^ |
| Nigeria | Adoga | 2009 | Subnational | No | Yes | No | 7 | ^256^ |
| Nigeria | Onoja | 2016 | City | No | No | Yes | 4 | ^257^ |
| Nigeria | Dada | 2006 | Subnational | No | Yes | No | 7 | ^258^ |
| Nigeria | Abba | 2011 | Facility | No | Yes | No | 5 | ^259^ |
| Congo | National Council for the Fight against AIDS | 2012 | National | No | No | Yes | 6 | ^260^ |
| Senegal | Ministry of Health and Social Action | 2020 | National | Yes | Yes | No | 7 | ^261^ |
| Senegal | Jaquet | 2016 | Facility | No | Yes | No | 6 | ^262^ |
| Sierra Leone | Ampofo | 2021 | National | Yes | Yes | No | 7 | ^263^ |
| Sierra Leone | Sesay | 2012 | National | No | No | Yes | 6 | ^264^ |
| South Africa | The Aurum Institute NICD | 2022 | Facility | Yes | Yes | No | 4 | ^265^ |
| South Africa | The Aurum Institute NICD | 2020 | Facility | Yes | Yes | No | 4 | ^265^ |
| South Africa | Skiti | 2013 | Facility | No | No | Yes | 5 | ^266^ |
| South Africa | Telisinghe | 2014 | Facility | No | Yes | No | 6 | ^267^ |
| South Africa | Stevenson | 2020 | Subnational | No | No | Yes | 5 | ^268^ |
| Swaziland | Dlamini | 2012 | National | Yes | Yes | No | 8 | ^269^ |
| United Republic of Tanzania | Angolwisye | 2011 | Subnational | No | No | Yes | 7 | ^270^ |
| United Republic of Tanzania | Mutayoba | 2014 | Subnational | Yes | Yes | No | 8 | ^271^ |
| United Republic of Tanzania | Steiner | 2015 | Facility | No | Yes | No | 6 | ^272^ |
| United Republic of Tanzania | Dahoma | 2009 | Subnational | No | No | Yes | 6 | ^273^ |
| Togo | Jaquet | 2016 | Facility | No | Yes | No | 6 | ^262^ |
| Togo | Ekouevi | 2013 | National | No | No | Yes | 7 | ^274^ |
| Togo | Akakpo | 2013 | Facility | No | Yes | No | 5 | ^275^ |
| Uganda | United Nations Office on Drugs and Crime | 2008 | National | Yes | Yes | No | 8 | ^276^ |
| Uganda | Kinaalwa | 2015 | Subnational | No | No | Yes | 6 | ^277^ |
| Zambia | Harris | 2014 | Facility | No | No | Yes | 6 | ^278^ |
| Zambia | Simooya | 2001 | Subnational | Yes | Yes | No | 7 | ^279^ |
| Zambia | Simooya | 2014 | National | Yes | Yes | No | 6 | ^280^ |
| Zambia | Maggard | 2015 | National | No | No | Yes | 6 | ^281^ |
| Zambia | Kagujje | 2021 | National | Yes | Yes | No | 7 | ^282^ |
| **Middle East & North Africa** |  |  |  |  |  |  |  |  |
| Egypt | Mohamed | 2013 | Facility | No | No | Yes | 5 | ^283^ |
| Lebanon | Ministry of Health Lebanon | 2008 | Facility | No | Yes | No | 6 | ^284^ |
| Libya | Ziglam | 2012 | Subnational | No | Yes | No | 6 | ^285^ |
| Morocco | El Ghrari | 2007 | Facility | Yes | No | No | 6 | ^286^ |
| Sudan | Sudan HIV Epidemiology Analysis Group | 2013 | National | No | No | Yes | 6 | ^287^ |
| Syrian Arab Republic | Kobeissi | 2014 | Facility | No | No | Yes | 5 | ^288^ |
| Türkiye | Sahin | 2018 | Facility | No | Yes | No | 5 | ^289^ |
| Türkiye | Balci | 2012 | Subnational | No | No | Yes | 6 | ^290^ |
| Türkiye | Keten | 2016 | Facility | No | No | Yes | 6 | ^291^ |

**References**

1. Weilandt C, Stöver H, Eckert J, Grigoryan G. Anonymous survey on infectious diseases and related risk behaviour among Armenian prisoners and prison staff. *International Journal of Prisoner Health* 2007.

2. V. Kasumov AK, D. Makhmudova, F. Juzbashov, S. Hasiev, S. Babazade, R. Sultanova, G. Kasumova, N. Kerimova. Prevalence of HIV, hepatitis and syphilis, and behavioural risk factors among most-at-risk groups in the Republic of Azerbaijan. In: Ministry of Health of the Republic of Azerbaijan Republican AIDS Centre, editor.; 2008.

3. Azbel L, Wickersham JA, Wegman MP, et al. Burden of substance use disorders, mental illness, and correlates of infectious diseases among soon-to-be released prisoners in Azerbaijan. *Drug and Alcohol Dependence* 2015.

4. Jurja-Ivana Čakalo SH. The report on results of a surveillance survey on knowledge, risks and prevalence of HIV and sexually and parenterally transmitted infections in most-at-risk populations in Azerbaijan. 2012.

5. Handanagic S. Report on the Integrated Bio-behavioural Surveillance Surveys among Key Populations in Azerbaijan, 2015. In: Ministry of Health of Republic of Azerbaijan WHO, WHO Collaborating Centre for HIV Surveillance, Zagreb Croatia, editor.; 2015.

6. Ravlija J, Vasilj I, Marijanovic I, Vasilj M. Risk behaviour of prison inmates in relation to HIV/STI. *Psychiatria Danubina* 2014.

7. Popov G, Plochev K. Prevalence and correlates of hepatitis C virus infection among inmates of Bulgarian prisons. *Clinical Microbiology and Infection* 2011.

8. Panayotov AS, G; Petkova, I; Ivanova, T; Metodieva, I; Chipeva, S. Annual Report on the problems related to drugs and drug addiction in Bulgaria: National Focus Center on Drugs and Addiction, 2022.

9. Kivimets K, Uuskula A. HIV testing and counselling in Estonian prisons, 2012 to 2013: aims, processes and impacts. *Euro Surveill* 2014.

10. Vanya M, Szili K, Magori K, Krisztina V. Skin diseases and sexually transmitted infection in a Hungarian prison. *Reviews and Research in Medical Microbiology* 2017.

11. Tresó B, Barcsay E, Tarján A, et al. Prevalence and correlates of HCV, HVB, and HIV infection among prison inmates and staff, Hungary. *J Urban Health* 2012.

12. Azbel L, Wickersham JA, Grishaev Y, Dvoryak S, Altice FL. Burden of infectious diseases, substance use disorders, and mental illness among Ukrainian prisoners transitioning to the community. *PLoS One* 2013.

13. Balakireva O SV, Salabai N, Kryvoruk A. Analysis of HIV/AIDS Response in Penitentiary System of Ukraine: Ukrainian Institute for Social Research after Olexander Yaremenko; UNODC, 2012.

14. Silbernagl M, Slamanig R, Fischer G, Brandt L. Hepatitis C infection and psychiatric burden in two imprisoned cohorts: Young offenders and opioid-maintained prisoners. *Health Policy* 2018.

15. Todts S, Glibert P, Van Malderen S, Van Huyck C, Saliez V, Hogge M. Usage de drogues dans les prisons belges: monitoring des risques sanitaires. *Bruxelles: SPF Justice* 2008.

16. Busschots D, Kremer C, Bielen R, et al. A multicentre interventional study to assess blood-borne viral infections in Belgian prisons. *BMC Infectious Diseases* 2021.

17. Burek V, Horvat J, Susic E, Mikulic R. Prevalence of hepatitis B and C among prison population in Croatia. [Croatian]. *Acta Medica Croatica* 2009.

18. Vilibic-Cavlek T, Gjenero-Margan I, Retkovac B, et al. Sociodemographic characteristics and risk behaviors for HIV, hepatitis B and hepatitis C virus infection among Croatian male prisoners. *International Journal pf Prisoner Health* 2011; **7**(1): 28-31.

19. Christensen PB, Krarup HB, Niesters HGM, Norder H, Georgsen J. Prevalence and incidence of bloodborne viral infections among Danish prisoners. *European Journal of Epidemiology* 2000.

20. Morey S, Hamoodi A, Jones D, et al. Increased diagnosis and treatment of hepatitis C in prison by universal offer of testing and use of telemedicine. *J Viral Hepat* 2019.

21. Mahto M, Zia S. Measuring the gap: from Home Office to the National Health Service in the provision of a one-stop shop sexual health service in a female prison in the UK. *Int J STD AIDS* 2008.

22. Aisyah DN, Shallcross L, Hayward A, et al. Hepatitis C among vulnerable populations: A seroprevalence study of homeless, people who inject drugs and prisoners in London. *J Viral Hepat* 2018.

23. Weild AR, Gill ON, Bennett D, Livingstone SJ, Parry JV, Curran L. Prevalence of HIV, hepatitis B, and hepatitis C antibodies in prisoners in England and Wales: a national survey. *Commun Dis Public Health* 2000.

24. Rautanen M, Harald, K, & Tyni, S. The Health and Wellbeing of Finnish Prisoners 2023 (Wattu IV). *Finnish institute for health and welfare (THL) Report 007/2023 256 pages Helsinki 2023* 2023.

25. Viitanen P, Vartiainen H, Aarnio J, et al. Hepatitis A, B, C and HIV infections among Finnish female prisoners--young females a risk group. *J Infect* 2011.

26. Jacomet C, Guyot-Lénat A, Bonny C, et al. Addressing the challenges of chronic viral infections and addiction in prisons: the PRODEPIST study. *European Journal of Public Health* 2016.

27. Izquierdo L, Mellon G, Buchaillet C, et al. Prevalence of hepatitis E virus and reassessment of HIV and other hepatitis virus seroprevalences among French prison inmates. *PLoS One* 2019.

28. Semaille C, Le Strat Y, Chiron E, et al. Prevalence of human immunodeficiency virus and hepatitis C virus among French prison inmates in 2010: a challenge for public health policy. *Euro surveillance : bulletin Europeen sur les maladies transmissibles = European communicable disease bulletin* 2013.

29. Reynaud-Maurupt C, Caer Y, Escaffre N, et al. High-dose buprenorphine substitution during incarceration - Management of opiate addicts. *Presse Medicale* 2005.

30. Lelievre C, Prissette G, Reuche AA, et al. Detection of sexually transmitted infections at the Amiens prison. State of play from February 2019 to May 2019. *Revue de Medecine Legale* 2020.

31. Rotily M, Weilandt C, Bird SM, et al. Surveillance of HIV infection and related risk behaviour in European prisons: A multicentre pilot study. *European journal of public health* 2001.

32. Perrodeau F, Pillot-Debelleix M, Vergniol J, et al. Optimizing hepatitis B vaccination in prison. *Med Mal Infect* 2016.

33. Abel S, Cuzin L, Da Cunha S, et al. Reaching the WHO target of testing persons in jails in prisons will need diverse efforts and resources. *PLoS One* 2018.

34. Schulte B, Stover H, Thane K, Schreiter C, Gansefort D, Reimer J. Substitution treatment and HCV/HIV-infection in a sample of 31 German prisons for sentenced inmates. *International journal of prisoner health* 2009.

35. Lehmann M, Meyer MF, Monazahian M, Tillmann HL, Manns MP, Wedemeyer H. High rate of spontaneous clearance of acute hepatitis C virus genotype 3 infection. *J Med Virol* 2004.

36. Fotiadou M, Livaditis M, Manou I, et al. Self-reported substance misuse in Greek male prisoners. *Eur Addict Res* 2004.

37. Wright B, Duffy D, Curtin K, Linehan S, Monks S, Kennedy HG. Psychiatric morbidity among women prisoners newly committed and amongst remanded and sentenced women in the Irish prison system. *Irish journal of psychological medicine* 2006.

38. National Advisory Committee on Drugs and Alcohol. Drugs and health in Irish prisons 2011. A report for prisoners., 2014.

39. Bannan CL, Lynch PA, Conroy EP, et al. Point-of-care testing for HIV in an Irish prison setting: results from three major Irish prisons. *Int J STD AIDS* 2016.

40. Crowley D, Lambert JS, Betts-Symonds G, et al. The seroprevalence of untreated chronic hepatitis C virus (HCV) infection and associated risk factors in male Irish prisoners: a cross-sectional study, 2017. *Euro Surveill* 2019.

41. Allwright S, Bradley F, Long J, Barry J, Thornton L, Parry JV. Prevalence of antibodies to hepatitis B, hepatitis C, and HIV and risk factors in Irish prisoners: results of a national cross sectional survey. *Bmj* 2000.

42. Drummond A, Codd M, Donnelly N, et al. Study on the prevalence of drug use, including intravenous drug use, and blood-borne viruses among the Irish prisoner population. *Dublin: National Advisory Committee on Drugs and Alcohol* 2014.

43. Stasi C, Silvestri C, Fanti E, Di Fiandra T, Voller F. Prevalence and features of chronic viral hepatitis and HIV coinfection in Italian prisons. *Eur J Intern Med* 2016.

44. Babudieri S, Longo B, Sarmati L, et al. Correlates of HIV, HBV, and HCV infections in a prison inmate population: results from a multicentre study in Italy. *Journal of Medical Virology* 2005.

45. Sagnelli E, Starnini G, Sagnelli C, et al. Blood born viral infections, sexually transmitted diseases and latent tuberculosis in italian prisons: a preliminary report of a large multicenter study. *European Review for Medical and Pharmacological Sciences* 2012.

46. Giuliani R, Casigliani V, Fornili M, et al. HCV micro-elimination in two prisons in Milan, Italy: A model of care. *Journal of Viral Hepatitis* 2020.

47. Ranieri R, Sommella J, D'Angelo C, et al. Antiretroviral therapy in inmates: Between guidelines and reality of Italian correctional facilities. [Italian]. *Infezioni in Medicina* 2015.

48. Monarca R, Madeddu G, Ranieri R, et al. HIV treatment and care among Italian inmates: a one-month point survey. *BMC Infectious Diseases* 2015.

49. Voller F, Silvestri C, Orsini C, Aversa L, Da Frè M, Cipriani F. [The health conditions of prison inmates in Tuscany]. *Epidemiol Prev* 2011.

50. Ciccarese G, Drago F, Oddenino G, Crosetto S, Rebora A, Parodi A. Sexually transmitted infections in male prison inmates. Prevalence, level of knowledge and risky behaviours. *Infez Med* 2020.

51. Muscat K, Cremona C, Fenech TM, Abela M, Padovese V. Sexually transmitted infections epidemiology and risk assessment at the main correctional facility in Malta (2017-2019). *Journal of the European Academy of Dermatology and Venereology* 2022.

52. Jovanovska T, Kocic B, Stojcevska VP. Prevalence, attitudes and knowledge about HIV HBV and HCV infections among inmates in prisons Prilep and Bitola--a pilot study. *Coll Antropol* 2014.

53. Danis K, Doherty L, McCartney M, McCarrol J, Kennedy H. Hepatitis and HIV in Northern Ireland prisons: a cross-sectional study. *Euro Surveill* 2007; **12**(1).

54. da Silva Marques NM, Margalho R, Melo MJ, da Cunha JGS, Melico-Silvestre AA. Seroepidemiological survey of transmissible infectious diseases in a Portuguese prison establishment. *Brazilian Journal of Infectious Diseases* 2011.

55. Passadouro R. [Prevalence infections and risk factors due to HIV, Hepatitis B and C in a prison establishment in Leiria]. *Acta Medica Portuguesa* 2004.

56. Garcia A, Exposto F, Prieto E, Lopes M, Duarte A, da Silva RC. Association of Trichomonas vaginalis with sociodemographic factors and other STDs among females inmates in Lisbon. *International Journal of STD & AIDS* 2004.

57. Barros H, Ramos E, Lucas R. A survey of HIV and HCV among female prison inmates in Portugal. *Cent Eur J Public Health* 2008.

58. Peters SE, Bissett B, Cassells Y, Paton J, Aitken C. HIV testing and care in prisoners: The first year results of opt-out BBV testing in Glasgow, UK. *Journal of the International AIDS Society* 2016.

59. Rotily M, Delorme C, Galinier A, Escaffre N, Moatti JP. HIV risk behavior in prison and factors related to reincarceration among injecting drug users. *Presse Medicale* 2000.

60. Gonzalez C, Canals J, Ortiz M, et al. Prevalence and determinants of high-risk human papillomavirus (HPV) infection and cervical cytological abnormalities in imprisoned women. *Epidemiology and Infection* 2008.

61. García-Guerrero J MMA, Sáiz de la Hoya Zamácola P, Vera-Remartínez EJ. Multi-centre study of the prevalence of latent tuberculosis infection amongst inmates in Spanish prisons. 2010.

62. Marco A, Saiz de la Hoya P, Garcia-Guerrero J, Grupo P. [Multi-centre study of the prevalence of infection from HIV and associated factors in Spanish prisons]. [Spanish]. *Revista espanola de sanidad penitenciaria* 2012.

63. Olivan G. The health profile of Spanish incarcerated delinquent youths. [References]. *Journal of Adolescent Health* 2001.

64. Vicente-Alcalde N, Tuells J, Egoavil CM, Ruescas-Escolano E, Altavilla C, Caballero P. Immunization Coverage of Inmates in Spanish Prisons. *International Journal of Environmental Research and Public Health* 2020.

65. Ferrer-Castro V, Crespo-Leiro MR, García-Marcos LS, et al. [Evaluation of needle exchange program at Pereiro de Aguiar prison (Ourense, Spain): ten years of experience]. *Revista Espanola de Sanidad Penitenciaria* 2012.

66. Murcia J, Portilla J, Bedia M, et al. Chronic hepatitis C virus infection and associated liver disease among the inmates of a Spanish prison. *Enfermedades Infecciosas y Microbiologia Clinica* 2009.

67. Cuadrado A, Llerena S, Cobo C, et al. Microenvironment Eradication of Hepatitis C: A Novel Treatment Paradigm. *American Journal of Gastroenterology* 2018.

68. Marco A, Gallego C, Perez-Caceres V, et al. Public Health response to an outbreak of SARS-CoV2 infection in a Barcelona prison. *Epidemiology and Infection* 2021.

69. Serroukh SC-Y. Consumo de sustancias, tratamiento con psicofármacos y patología infecciosa en personas redusas del centro penitenciario Puig de las Basses. *Metas de Enfermería* 2022.

70. Mourino AM, Gallego Castellvi C, Garcia De Olalla P, et al. Late diagnosis of HIV infection among prisoners. *AIDS Reviews* 2013.

71. Martin V, Guerra JM, Cayla JA, Rodriguez JC, Blanco MD, Alcoba M. Incidence of tuberculosis and the importance of treatment of latent tuberculosis infection in a Spanish prison population. *Int J Tuberc Lung Dis* 2001.

72. Gahrton C, Westman G, Lindahl K, et al. Prevalence of Viremic hepatitis C, hepatitis B, and HIV infection, and vaccination status among prisoners in Stockholm County. *BMC Infectious Diseases* 2019.

73. Pala KC, Baggio S, Tran NT, Girardin F, Wolff H, Getaz L. Blood-borne and sexually transmitted infections: a cross-sectional study in a Swiss prison. *BMC Infectious Diseases* 2018.

74. Moschetti K, Stadelmann P, Wangmo T, et al. Disease profiles of detainees in the Canton of Vaud in Switzerland: gender and age differences in substance abuse, mental health and chronic health conditions. *BMC Public Health* 2015.

75. Blogg S, Utomo, B,, Silitonga N, Hidayati DAN, Sattler G. Indonesian National Inmate Bio-Behavioral Survey for HIV and Syphilis Prevalence and Risk Behaviors in Prisons and Detention Centers, 2010. *SAGE OPEN* 2014.

76. Ministry of Health Republic of Indonesia. IBBS 2011 Integrated Bioloigcal and Behavioral Survey, 2011.

77. Arends RM, Nelwan EJ, Soediro R, et al. Associations between impulsivity, risk behavior and HIV, HBV, HCV and syphilis seroprevalence among female prisoners in Indonesia: A cross-sectional study. *PLoS One* 2019.

78. Sembiring E, Ginting Y, Saragih RH. Factors associated with syphilis seropositive and Human Immunodeficiency Virus (HIV) infection among inmates at Lubuk Pakam prison, Indonesia. *1ST INT CONF ON TROP MED & INFECT DIS FAC OF MED UNIV SUMATERA UTARA IN CONJUNCTION WITH THE 23RD NATL CONGRESS OF THE INDONESIAN SOC OF TROP & INFECT DIS CONSULTANT AND THE 18TH ANNUAL MEETING OF INTERNAL MED DEPT FAC OF MED UNIV SUMATERA UTARA* 2018.

79. Rey I, Saragih R, Effendi-Ys R, Sembiring J, Siregar G, Zain L. Profile of hepatitis B and C virus infection in prisoners in Lubuk Pakam correctional facilities. IOP Conference Series: Earth and Environmental Science; 2018: IOP Publishing; 2018. p. 012033.

80. Mwe Nom NA, Kyaw KWY, Kumar AMV, et al. HIV care cascade among prisoners of the Mandalay Central Prison in Myanmar: 2011-2018. *Tropical Medicine and Infectious Disease* 2020.

81. Simbulan NP, Aguilar AS, Flanigan T, Cu-Uvin S. High-risk behaviors and the prevalence of sexually transmitted diseases among women prisoners at the women state penitentiary in Metro Manila. *Social Science & Medicine* 2001.

82. Choi S, Lee E, Bang JH. High Prevalence of Human Immunodeficiency Virus Infection among Inmates in Korean Correctional Facilities. *J Korean Med Sci* 2021.

83. Lu MY, Chen CT, Shih YL, et al. Changing epidemiology and viral interplay of hepatitis B, C and D among injecting drug user-dominant prisoners in Taiwan. *Sci Rep* 2021.

84. Morasert T, Worapas W, Kaewmahit R, Uphala W. Prevalence and risk factors associated with tuberculosis disease in Suratthani Central Prison, Thailand. *International Journal of Tuberculosis and Lung Disease* 2018.

85. Johns Hopkins University Bloomberg School of Public Health. Integrated Biological & Behavioral Surveillance (IBBS) in Selected Cities of Afghanistan Findings of 2012 IBBS survey and comparison to 2009 IBBS

survey. *National AIDS Control Program (NACP) Ministry of Public Health, Afghanistan* 2012.

86. Johns Hopkins University Bloomberg School of Public Health. Integrated Behavioral & Biological Surveillance (IBBS) in Afghanistan: Year 1 Report. *Johns Hopkins University Bloomberg School of Public Health* 2011.

87. Kosambiya JK, Vadgama P, Samudyatha UC, Rathod D, Buch R, Damor R. Active case finding of pulmonary tuberculosis and HIV infection among prisoners of South Gujarat: A cross sectional study. *Indian Journal of Tuberculosis* 2022.

88. Sabharwal ER, Mathur DR, Mehta P. HIV seroprevalence among prison inmates in Rajasthan, India. *Journal of Clinical and Diagnostic Research* 2012.

89. Choudhury R, Singh N. Prevalence of HIV/AIDS in inmates of two district jails of central Uttar Pradesh, India. *Medico-Legal Update* 2016.

90. Tyagi SK, Sovani V, Dias NP, Tyagi D, Saxena S. Prevalence and risk factors of HCV infection in a prison setting in Uttar Pradesh, India. *Indian Journal of Public Health Research and Development* 2018.

91. Organization NAC. HIV Sentinel Surveillance Plus 2019, Central Prison Sites, 2019.

92. National AIDS Control Organization. HIV Sentinel Surveillance Plus 2021, Central Prison Sites, 2022.

93. Ziaee M, Sharifzadeh G, Namaee MH, Fereidouni M. Prevalence of HIV and Hepatitis B, C, D Infections and Their Associated Risk Factors among Prisoners in Southern Khorasan Province, Iran. *Iranian Journal of Public Health* 2014.

94. Haghdoost AA, Mirzazadeh A, Shokoohi M, Sedaghat A, Gouya MM. HIV trend among Iranian prisoners in 1990s and 2000s; analysis of aggregated data from HIV sentinel sero-surveys. *Harm Reduct J* 2013.

95. Shahesmaeili A, Karamouzian M, Tavakoli F, et al. HIV prevalence and continuum of care among incarcerated people in Iran from 2010 to 2017. *Harm Reduct J* 2022.

96. Khademi N, Shakiba E, Khodadost M, Khoramdad M. Seroprevalence and related risk behaviors of hepatitis C, hepatitis B and HIV infections among Male prisoners in Kermanshah, Iran. *Archives of Iranian Medicine* 2019.

97. Nokhodian Z, Yazdani MR, Yaran M, et al. Prevalence and risk factors of HIV, syphilis, hepatitis B and C among female prisoners in Isfahan, Iran. *Hepatitis Monthly* 2012.

98. Seyedalinaghi SA, Farhoudi B, Mohraz M, et al. Prevalence and Associated Factors of HIV Infection among Male Prisoners in Tehran, Iran. *Archives of Iranian Medicine* 2017.

99. Shahbazi M, Farnia M, Rahmani K, Moradi G. Trend of HIV/AIDS Prevalence and Related Interventions Administered in Prisons of Iran -13 Years' Experience. *Iranian Journal of Public Health* 2014.

100. Wali A, Khan D, Safdar N, et al. Prevalence of tuberculosis, HIV/AIDS, and hepatitis; in a prison of Balochistan: a cross-sectional survey. *BMC Public Health* 2019.

101. Nafees M, Qasim A, Jafferi G, Anwar MS, Muazzam M. HIV infection, HIV/HCV and HIV/HBV co-infections among jail inmates of Lahore. *Pakistan Journal of Medical Sciences* 2011.

102. Kazi AM, Shah SA, Jenkins CA, Shepherd BE, Vermund SH. Risk factors and prevalence of tuberculosis, human immunodeficiency virus, syphilis, hepatitis B virus, and hepatitis C virus among prisoners in Pakistan. *International Journal pf Infectious Diseases* 2010.

103. Pervaiz A, Ghafoor T, Asghar RJ. Screening of prisoners for Human Immunodeficiency Virus (HIV), Hepatitis C (HCV) and B (HBV) in Punjab Province, Pakistan, 2009. *International Journal of Infectious Diseases* 2012.

104. Khan MD, Wali A, Fatima R, Yaqoob A, Aziz S. Prevalence and associated risk factors of HIV in prisons in Balochistan, Pakistan: A cross-sectional study. *F1000Research* 2019.

105. Safdar S, Mehmood A, Abbas SQ. Prevalence of HIV/AIDS among jail inmates in Sindh. *J Pak Med Assoc* 2009.

106. Shah SSA, Ali M, Ahmad M, Hamadan U. Screening of jail inmates for HIV and tuberculosis. *Pakistan Journal of Medical and Health Sciences* 2013.

107. Azbel L, Polonsky M, Wegman M, et al. Intersecting epidemics of HIV, HCV, and syphilis among soon-to-be released prisoners in Kyrgyzstan: Implications for prevention and treatment. *International Journal of Drug Policy* 2016.

108. Ministry of Health and Social Protection The Republic of Tajikistan. Reference on the implementation of the National Epidemic Control Program human immunodeficiency syndrome in the Republic of Tajikistan for 2017 2020, 2019.

109. Boisson EV, Trotman C. HIV seroprevalence among male prison inmates in the six countries of the Organization of Eastern Caribbean states in the Caribbean (OECS). *West Indian Med J* 2009.

110. Andrinopoulos K, Kerrigan D, Figueroa JP, et al. Establishment of an HIV/sexually transmitted disease programme and prevalence of infection among incarcerated men in Jamaica. *Int J STD AIDS* 2010.

111. Adaszko D, Sotelo JA, Orlando M, Angelerei P. Estudio de prevalencia de VIH, sífilis, hepatitis virales y tuberculosis en personas en contextos de encierro en unidades del Servicio Penitenciario Federal. *Buenos Aires, Ministerio de Salud* 2017.

112. Mendizabal M, Testa P, Rojas M, et al. Pilot study using the ECHO model to enhance linkage to care for patients with hepatitis C in the custodial setting. *Journal of Viral Hepatitis* 2020.

113. Gough E, Edwards P. HIV seroprevalence and associated risk factors among male inmates at the Belize Central Prison. *Rev Panam Salud Publica* 2009.

114. Villarroel-Torrico M, Montaño K, Flores-Arispe P, et al. Syphilis, human immunodeficiency virus, herpes genital and hepatitis B in a women's prison in Cochabamba, Bolivia: prevalence and risk factors. *Revista Espanola de Sanidad Penitenciaria* 2018.

115. Lambert ML, Torrico F, Billot C, Mazina D, Marleen B, Van der Stuyft P. Street youths are the only high-risk group for HIV in a low-prevalence South American country. *Sex Transm Dis* 2005; **32**(4): 240-2.

116. Vale EP, Carvalho LD, Pereira FCD. HIV seroprevalence in prisoners in Amapascritores. *REVISTA DE EPIDEMIOLOGIA E CONTROLE DE INFECCAO* 2016.

117. Machado F, Becker D, de Oliveira CF, Possuelo LG, Renner JDP. Seroprevalence of HIV, hepatitis B and C and syphilis infection in prisoners of the central region of Rio Grande do Sul, Brazil. *O Mundo da Saúde* 2019.

118. Lopes F, Latorre MR, Campos Pignatari AC, Buchalla CM. [HIV, HPV, and syphilis prevalence in a women's penitentiary in the city of São Paulo, 1997-1998]. *Cad Saude Publica* 2001.

119. Leal M, Kerr L, Mota RMS, Neto RDP, Seal D, Kendall C. Health of female prisoners in Brazil. *Ciencia & saude coletiva* 2022.

120. Pompilio MA, Pontes ERJC, Castro ARCM, et al. Prevalence and epidemiology of chronic hepatitis c among prisoners of Mato Grosso do Sul State, Brazil. *Journal of Venomous Animals and Toxins Including Tropical Diseases* 2011.

121. Guimarães T, Granato CF, Varella D, Ferraz ML, Castelo A, Kallás EG. High prevalence of hepatitis C infection in a Brazilian prison: identification of risk factors for infection. *The Brazilian Journal of Infectious Diseases* 2001.

122. Benedetti MSG, Nogami ASA, da Costa BB, et al. Sexually transmitted infections in women deprived of liberty in Roraima, Brazil. *Revista de Saude Publica* 2020.

123. Strazza L, Massad E, Azevedo RS, Carvalho HB. Behavior associated with HIV and HCV infection in female prison inmates in Sao Paulo, Brazil. *Cadernos de Saude Publica* 2007.

124. de Albuquerque ACC, da Silva DM, Rabelo DCC, et al. Seroprevalence and factors associated with human immunodeficiency virus (HIV) and syphilis in inmates in the state of Pernambuco, Brazil. *Ciencia & saude coletiva* 2014.

125. Ronchi BR, Rios GM, Knoll RK, Cardoso C. Prevalence of HIV, sifilis, hepatitis B and hepatitis c in the inmates of the penitentiary complex of vale do Itajai-SC. *Sexually Transmitted Infections* 2017.

126. Sgarbi RVE, Carbone ADS, Paiao DSG, et al. A Cross-Sectional Survey of HIV Testing and Prevalence in Twelve Brazilian Correctional Facilities. *PLoS One* 2015.

127. Valença MS, Scaini JL, Abileira FS, Gonçalves CV, von Groll A, Silva PE. Prevalence of tuberculosis in prisons: risk factors and molecular epidemiology. *International Journal of Tuberculosis and Lung Disease* 2015.

128. Defante Ferreto LE, Guedes S, Braz Pauli F, et al. Seroprevalence and associated factors of HIV and Hepatitis C in Brazilian high-security prisons: A state-wide epidemiological study. *PLoS One* 2021.

129. dos Santos Bet GM, de Souza GHdA, Croda J, et al. Treatment outcomes of brazilian inmates with treponema pallidum and human immunodeficiency virus infection: A prospective cohort study. *The American Journal of Tropical Medicine and Hygiene* 2018; **98**(6): 1603.

130. Catalan-Soares BC, Almeida RT, Carneiro-Proietti AB. Prevalence of HIV-1/2, HTLV-I/II, hepatitis B virus (HBV), hepatitis C virus (HCV), Treponema pallidum and Trypanosoma cruzi among prison inmates at Manhuacu, Minas Gerais State, Brazil. *Revista da Sociedade Brasileira de Medicina Tropical* 2000.

131. do Nascimento CT, Pena DZ, Giuffrida R, et al. Prevalence and epidemiological characteristics of inmates diagnosed with infectious diseases living in a region with a high number of prisons in Sao Paulo state, Brazil. *BMJ Open* 2020.

132. El Maerrawi I, Carvalho HB. Prevalence and risk factors associated with HIV infection, hepatitis and syphilis in a state prison of São Paulo. *Int J STD AIDS* 2015.

133. Prellwitz IM, Alves BM, Ikeda ML, et al. HIV behind bars: human immunodeficiency virus cluster analysis and drug resistance in a reference correctional unit from southern Brazil. *PLoS One* 2013.

134. Massad E, Rozman M, Azevedo R, et al. Seroprevalence of HIV, HCV and syphilis in Brazilian prisoners: preponderance of parenteral transmission. *European journal of epidemiology* 1999; **15**: 439-45.

135. Leite AGD, Damasceno LM, Conceicao SC, Motta PFC. Rapid tests for HIV, syphilis, and chronic hepatitis in a prison population in a prison complex in Salvador (BA), Brazil. *Ciencia & saude coletiva* 2022.

136. Soares Epifania P, Santos Passos Costa J, Costa Barros KC, Santos de Freitas K, Sampaio Maciel G, da Silva Santos Passos S. Doenças infectocontagiosas em indivíduos privados de liberdade. *Enfermagem Brasil* 2022.

137. Miranda AE, Vargas, P.M., Louis, M.E.S. & Viana, M.C. Sexually transmitted diseases among female prisoners in Brazil: prevalence and risk factors. *Sexually transmitted diseases* 2000; **27**(9): 491-5.

138. Coelho HC, Perdoná GC, Neves FR, Passos AD. HIV prevalence and risk factors in a Brazilian penitentiary. *Cad Saude Publica* 2007.

139. Pelissari DM, Kuhleis DC, Bartholomay P, et al. Prevalence and screening of active tuberculosis in a prison in the South of Brazil. *International Journal of Tuberculosis and Lung Disease* 2018.

140. Marins JR, Page-Shafer K, De Azevedo Barros MB, Hudes ES, Chen S, Hearst N. Seroprevalence and risk factors for HIV infection among incarcerated men in Sorocaba, Brazil. *AIDS and behavior* 2000.

141. Moura RJ, Romero GAS. HIV prevalence in recently incarcerated adult males in the Federal District, Brasilia, Brazil. *Rev Soc Bras Med Trop* 2020.

142. Sousa KAA, Araujo TME, Teles SA, Rangel EML, Nery IS. Factors associated with HIV prevalence in a prison population. *Revista da Escola de Enfermagem da U S P* 2017.

143. Felisberto M, Saretto AA, Wopereis S, Treitinger A, Machado MJ, Spada C. Prevalence of human immunodeficiency virus infection and associated risk factors among prison inmates in the city of Florianopolis. *Revista da Sociedade Brasileira de Medicina Tropical* 2016.

144. Sanchez-Vanegas G, Rodriguez-Vallejo D, Pinzon-Duran AC, Reina-Cifuentes MA, Monterrosa-Blanco A, Tiga-Segura JA. Prevalence of syphilis, hepatitis B and human immunodeficiency virus in the male prison population in Bogota, Colombia in 2019. [Spanish]. *Infectio* 2020.

145. Alvarez Rodriguez BE, Pinzon Z, Huaman BJ, et al. Prevalence of HIV, syphilis, drugs use and sexual risk behaviours among prisoners in Guatemala, 2012. *Sexually Transmitted Infections Conference: STI and AIDS World Congress* 2013.

146. Belaunzaran-Zamudio PF, Mosqueda-Gomez JL, Macias-Hernandez A, Rodríguez-Ramírez S, Sierra-Madero J, Beyrer C. Burden of HIV, Syphilis, and Hepatitis B and C Among Inmates in a Prison State System in Mexico. *AIDS Res Hum Retroviruses* 2017.

147. Silverman-Retana O, Servan-Mori E, McCoy SI, Larney S, Bautista-Arredondo S. Hepatitis C antibody prevalence among Mexico City prisoners injecting legal and illegal substances. *Drug and Alcohol Dependence* 2017.

148. Bautista-Arredondo S, González A, Servan-Mori E, et al. A Cross-Sectional Study of Prisoners in Mexico City Comparing Prevalence of Transmissible Infections and Chronic Diseases with That in the General Population. *PLoS One* 2015.

149. Alvarado-Esquivel C, Sablon E, Martínez-García S, Estrada-Martínez S. Hepatitis virus and HIV infections in inmates of a state correctional facility in Mexico. *Epidemiology and Infection* 2005.

150. Garaycochea MC, Pino R, Chavez I, et al. Sexually transmitted infections in women living in a prison in Lima, Peru. *Revista Peruana de Medicina Experimental y Salud Publica* 2013.

151. Cyrus E, Sanchez J, Madhivanan P, et al. Prevalence of Intimate Partner Violence, Substance Use Disorders and Depression among Incarcerated Women in Lima, Perú. *Int J Environ Res Public Health* 2021.

152. Troya MM, Vila RB. HIV infection and associated risk behaviours in a prison in Montevideo, Uruguay. *Rev Esp Sanid Penit* 2010; **12**(1): 21-8.

153. Gil YMF. Estudio de prevalencia de VIH en población de Personas Privada de Libertad en 30 establecimientos penitenciarios de la República Bolivariana de Venezuela, 2022. In: Salud MdPPpl, editor.; 2022.

154. Posada A, Díaz Tremarias M. [HIV, hepatitis B and syphilis infection in inmates of Venezuela's prisons, 1998-2001]. *Rev Esp Sanid Penit* 2008; **10**(3): 73-9.

155. Alcivar JC, Zambrano MM, Madronero MG, et al. Sexually transmitted infections in inmates in Merida Venezuela. *INVESTIGACION CLINICA* 2020.

156. Courtemanche Y, Poulin C, Serhir B, Alary M. HIV and hepatitis C virus infections in Quebec's provincial detention centres: comparing prevalence and related risky behaviours between 2003 and 2014-2015. *Can J Public Health* 2018.

157. Poulin C, Alary M, Lambert G, et al. Prevalence of HIV and hepatitis C virus infections among inmates of Quebec provincial prisons. *Cmaj* 2007.

158. Calzavara L, Ramuscak N, Burchell AN, et al. Prevalence of HIV and hepatitis C virus infections among inmates of Ontario remand facilities. *Cmaj* 2007.

159. Ford PM, Pearson M, Sankar-Mistry P, Stevenson T, Bell D, Austin J. HIV, hepatitis C and risk behaviour in a Canadian medium-security federal penitentiary. Queen's University HIV Prison Study Group. *Qjm* 2000.

160. Begier EM, Bennani Y, Forgione L, et al. Undiagnosed HIV infection among New York City jail entrants, 2006: results of a blinded serosurvey. *J Acquir Immune Defic Syndr* 2010.

161. Kendrick SR, Kroc KA, Couture E, Weinstein RA. Comparison of point-of-care rapid HIV testing in three clinical venues. *Aids* 2004.

162. Bauserman RL, Ward MA, Eldred L, Swetz A. Increasing voluntary HIV testing by offering oral tests in incarcerated populations. *Am J Public Health* 2001.

163. Klein SJ, O'Connell DA, Devore BS, Wright LN, Birkhead GS. Building an HIV continuum for inmates: New York state's criminal justice initiative. [References]. *AIDS Education and Prevention* 2002.

164. Kavasery R, Maru DS, Sylla LN, Smith D, Altice FL. A prospective controlled trial of routine opt-out HIV testing in a men's jail. *PLoS One* 2009.

165. de Ravello L, Brantley MD, Lamarre M, Qayad MG, Aubert H, Beck-Sague C. Sexually transmitted infections and other health conditions of women entering prison in Georgia, 1998-1999. *Sex Transm Dis* 2005.

166. Akiyama MJ, Kaba F, Rosner Z, et al. Correlates of hepatitis C virus infection in the targeted testing program of the New York city jail system: Epidemiologic patterns and priorities for action. *Public Health Reports* 2017.

167. Rowell-Cunsolo TL, Szeto B, Sampong SA, Larson EL. Predictors of sexual behaviour among men and women in New York City area prisons. *CULTURE HEALTH & SEXUALITY* 2016.

168. Smith L, Moncur BL, Anderson B, et al. HIV prevalence and care in the New York state department of corrections. *Topics in Antiviral Medicine* 2016.

169. Solomon L, Flynn C, Muck K, Vertefeuille J. Prevalence of HIV, syphilis, hepatitis B, and hepatitis C among entrants to Maryland correctional facilities. *Journal of Urban Health* 2004.

170. Alvarez KJ, Befus M, Herzig CTA, Larson E. Prevalence and correlates of hepatitis C virus infection among inmates at two New York State correctional facilities. *Journal of Infection and Public Health* 2014.

171. de Voux A, Spaulding AC, Beckwith C, et al. Early identification of HIV: empirical support for jail-based screening. *PLoS One* 2012; **7**(5): e37603.

172. MacGowan R, Margolis A, Richardson-Moore A, et al. Voluntary Rapid Human Immunodeficiency Virus (HIV) Testing in Jails. *Sexually Transmitted Diseases* 2009.

173. Krebs CP. INMATE FACTORS ASSOCIATED WITH HIV TRANSMISSION IN PRISON. *CRIMINOLOGY & PUBLIC POLICY* 2006.

174. Sampson LA. Screening for syphilis and HIV in North Carolina jails. *Dissertation Abstracts International: Section B: The Sciences and Engineering* 2009.

175. Tartaro C, Levy MP. An Evaluation of an HIV Testing Program in the Jail Setting: Results and Recommendations. *PRISON JOURNAL* 2013.

176. Sieck CJ, Dembe AE. Results of a pilot study of pre-release STD testing and inmates' risk behaviors in an Ohio prison. *J Urban Health* 2011.

177. Feld S, Steele J, Klinedinst S, et al. Implementing Opt-Out HIV Testing in the Alameda County Jails. *Journal of correctional health care : the official journal of the National Commission on Correctional Health Care* 2023.

178. Cocoros N, Nettle E, Church D, et al. Screening for hepatitis C as a prevention enhancement (SHAPE) for HIV: An integration pilot initiative in a massachusetts county correctional facility. *Public Health Reports* 2014.

179. Strick LB, MacGowan, R.J., Margolis & Belcher, L. HIV screening of male inmates during prison intake medical evaluation--Washington, 2006-2010. *MMWR Morb Mortal Wkly Rep* 2011.

180. Keleekai NL. Patterns and predictors of HIV, sexually transmitted infections, and staphylococcus aureus co-infection among New York state prison inmates. *Dissertation Abstracts International: Section B: The Sciences and Engineering* 2012.

181. Scott J, Sampson LA, Clymore JM, Moore PR, Leone PA. Integrated HIV, syphilis, and other STI testing in North Carolina county jails. *Sexually Transmitted Infections* 2011.

182. Leukefeld CG, Staton M, Hiller ML, et al. A descriptive profile of health problems, health services utilization, and HIV serostatus among incarcerated male drug abusers. *J Behav Health Serv Res* 2002.

183. Javanbakht M, Boudov M, Anderson LJ, et al. Sexually transmitted infections among incarcerated women: Findings from a decade of screening in a Los Angeles County jail, 2002-2012. [References]. *American journal of public health* 2014.

184. Ruiz JD, Molitor F, Plagenhoef JA. Trends in hepatitis C and HIV infection among inmates entering prisons in California, 1994 versus 1999. *Aids* 2002.

185. Nijhawan AE, Iroh PA, Brown LS, Winetsky D, Porsa E. Cost analysis of tuberculin skin test and the QuantiFERON-TB Gold In-tube test for tuberculosis screening in a correctional setting in Dallas, Texas, USA. *BMC Infect Dis* 2016.

186. Spaulding AC, Kim MJ, Corpening KT, Carpenter T, Watlington P, Bowden CJ. Establishing an HIV Screening Program Led by Staff Nurses in a County Jail. *J Public Health Manag Pract* 2015.

187. Beckwith CG, Nunn A, Baucom S, et al. Rapid HIV testing in large urban jails. *Am J Public Health* 2012.

188. Weant TE, Turner AN, Murphy-Weiss M, Murray DM, Wang SH. Can social history variables predict prison inmates' risk for latent tuberculosis infection? *Tuberc Res Treat* 2012; **2012**: 132406.

189. Rosen DL, Wohl DA, Golin CE, et al. Comparing HIV Case Detection in Prison During Opt-In vs. Opt-Out Testing Policies. *J Acquir Immune Defic Syndr* 2016.

190. Spaulding AC, Seals RM, McCallum VA, Perez SD, Brzozowski AK, Steenland NK. Prisoner survival inside and outside of the institution: implications for health-care planning. *Am J Epidemiol* 2011.

191. Desai J, Nijhawan A, Krakower D, Harris BL, Taherzadeh D. Hiv/sti testing and prep eligibility among women incarcerated in an urban county jail. *Topics in Antiviral Medicine* 2021.

192. Maruschak LM. HIV in Prisons, 2021 – Statistical Tables: Bureau of Justice Statistics, 2023.

193. Beckwith CG, Bazerman L, Cornwall AH, et al. An evaluation of a routine opt-out rapid HIV testing program in a Rhode Island jail. *AIDS Educ Prev* 2011.

194. Wohl DA, Golin C, Rosen DL, May JM, White BL. Detection of undiagnosed HIV among state prison entrants. *Jama* 2013.

195. Baillargeon J, Wu H, Kelley MJ, Grady J, Linthicum L, Dunn K. Hepatitis C seroprevalence among newly incarcerated inmates in the Texas correctional system. *Public Health* 2003.

196. Rosen DL, Schoenbach VJ, Wohl DA, White BL, Stewart PW, Golin CE. Characteristics and behaviors associated with HIV infection among inmates in the North Carolina prison system. *Am J Public Health* 2009.

197. Macalino GE, Vlahov D, Sanford-Colby S, et al. Prevalence and Incidence of HIV, Hepatitis B Virus, and Hepatitis C Virus Infections Among Males in Rhode Island Prisons. [References]. *American journal of public health* 2004.

198. Carvajal RI, Ross MW, Byrd T, Shelton A. HIV Counseling and Testing Program for Female Inmates: Analysis of Data From the Harris County Jail. [References]. *Journal of Correctional Health Care* 2005.

199. Baillargeon J, Pulvino JS, Leonardson JE, et al. The changing epidemiology of HIV in the criminal justice system. *Int J STD AIDS* 2017.

200. Kavasery R, Maru DS, Cornman-Homonoff J, Sylla LN, Smith D, Altice FL. Routine opt-out HIV testing strategies in a female jail setting: a prospective controlled trial. *PLoS One* 2009.

201. Harrison LD, Bachman T, Freeman C, Inciardi JA. The acceptability of the female condom among US women at high risk from HIV. [References]. *Culture, health & sexuality* 2001.

202. Spaulding A, Booker C, Freeman S, et al. Jails, HIV Testing, and Linkage to Care Services: An Overview of the EnhanceLink Initiative. *AIDS & Behavior* 2013.

203. Seth P, Figueroa A, Wang G, Reid L, Belcher L. HIV Testing, HIV Positivity, and Linkage and Referral Services in Correctional Facilities in the United States, 2009-2013. *Sex Transm Dis* 2015.

204. Peter P. Impact of Opt-Out and Opt-in HIV testing and education program on discovering HIV in jail populations. *Dissertation Abstracts International: Section B: The Sciences and Engineering* 2013.

205. Beckwith CG, Atunah-Jay S, Cohen J, et al. Feasibility and Acceptability of Rapid HIV Testing in Jail. [References]. *AIDS Patient Care and STDs* 2007.

206. Simonsen KA, Shaikh RA, Earley M, et al. Rapid HIV Screening in an Urban Jail: How Testing at Exit With Linkage to Community Care Can Address Perceived Barriers. *J Prim Prev* 2015.

207. Arriola KR, Braithwaite RL, Kennedy S, et al. A collaborative effort to enhance HIV/STI screening in five county jails. *Public Health Rep* 2001.

208. Chin ET, Leidner D, Zhang Y, et al. Effectiveness of Coronavirus Disease 2019 (COVID-19) Vaccines Among Incarcerated People in California State Prisons: Retrospective Cohort Study. *Clin Infect Dis* 2022.

209. Taussig J. HIV Transmission Among Male Inmates in a State Prison System -- Georgia, 1992-2005. (cover story). *MMWR: Morbidity & Mortality Weekly Report* 2006.

210. Baillargeon J, Black SA, Pulvino J, Dunn K. The disease profile of Texas prison inmates. *Ann Epidemiol* 2000.

211. Lucas KD, Eckert V, Behrends CN, Wheeler C, MacGowan RJ, Mohle-Boetani JC. Evaluation of Routine HIV Opt-Out Screening and Continuum of Care Services Following Entry into Eight Prison Reception Centers--California, 2012. *MMWR Morb Mortal Wkly Rep* 2016.

212. Chin ET, Ryckman T, Prince L, et al. COVID-19 in the California State Prison System: an Observational Study of Decarceration, Ongoing Risks, and Risk Factors. *J Gen Intern Med* 2021.

213. Katyal M, Leibowitz R, Venters H. IGRA-Based Screening for Latent Tuberculosis Infection in Persons Newly Incarcerated in New York City Jails. *J Correct Health Care* 2018.

214. Baillargeon JG, Paar DP, Wu H, et al. Psychiatric disorders, HIV infection and HIV/hepatitis co-infection in the correctional setting. [References]. *AIDS Care* 2008.

215. de la Flor C, Porsa E, Nijhawan AE. Opt-out HIV and Hepatitis C Testing at the Dallas County Jail: Uptake, Prevalence, and Demographic Characteristics of Testers. *Public Health Rep* 2017.

216. Rice DK. Design, implementation, and evaluation of a jail-based HIV screening program. *Dissertation Abstracts International: Section B: The Sciences and Engineering* 2011.

217. Altice FL, Marinovich A, Khoshnood K, Blankenship KM, Springer SA, Selwyn PA. Correlates of HIV infection among incarcerated women: implications for improving detection of HIV infection. *J Urban Health* 2005.

218. Wenger PJ, Rottnek F, Parker T, Crippin JS. Assessment of hepatitis C risk factors and infection prevalence in a jail population. *American journal of public health* 2014; **104**(9): 1722-7.

219. Kinner SA, Winter R, Saxton K. A longitudinal study of health outcomes for people released from prison in Fiji: the HIP-Fiji project. *Australas Psychiatry* 2015.

220. Bah R, Sheehan Y, Li X, et al. Prevalence of blood-borne virus infections and uptake of hepatitis C testing and treatment in Australian prisons: the AusHep study. *The Lancet Regional Health–Western Pacific* 2024; **53**.

221. Indig D, Topp L, Ross B, et al. 2009 NSW Inmate Health Survey: Key Findings Report. Sydney: Justice Health, 2010.

222. Butler TS, M. National Prison Entrants’ Bloodborne Virus and Risk Behaviour Survey Report: Kirby Institute 2017.

223. Butler T, Boonwaat L, Hailstone S, et al. The 2004 Australian prison entrants' blood-borne virus and risk behaviour survey. *Aust N Z J Public Health* 2007.

224. Gilles M, Swingler E, Craven C, Larson A. Prison health and public health responses at a regional prison in Western Australia. *Australia and New Zealand Journal of Public Health* 2008.

225. Watkins RE, Mak DB, Connelly C. Testing for sexually transmitted infections and blood borne viruses on admission to Western Australian prisons. *BMC Public Health* 2009.

226. Health J. National Patient Health Survey. 2017.

227. Reekie JM, Levy MH, Richards AH, et al. Trends in prevalence of HIV infection, hepatitis B and hepatitis C among Australian prisoners - 2004, 2007, 2010. *Medical Journal of Australia* 2014.

228. Stoové M, Kirwan A. External component of the evaluation of drug policies and services and their subsequent effects on prisoners and staff within the Alexander Maconochie Centre. 2011.

229. Lythgoe J, Kolodziej, J & Hollingshead, B. People living with HIV in prisons. 2022.

230. Hessou S, Dougnon VT, Glele-Ahanhanzo Y, et al. A behavioral and serological survey on HIV prevalence among prisoners in Benin. *Journal of Public Health in Africa* 2017.

231. Catraye DJ, Ky-Ba, A & Tavi-Ouattarra, A.Y. ENQUETE BIO COMPORTEMENTALE DU VIH-SIDA EN MILIEU CARCERAL AUPRES DES DETENUS HOMMES ET FEMMES AU BURKINA FASO. 2017.

232. Diendéré EA, Tiéno H, Bognounou R, et al. Prevalence and risk factors associated with infection by human immunodeficiency virus, hepatitis B virus, syphilis and bacillary pulmonary tuberculosis in prisons in Burkina Faso. *Medecine Tropicale : revue du Corps de sante colonial* 2011.

233. Ba AK, Sanou M, Diallo I, et al. Bio-behavioural HIV survey in prisons on men and women in Burkina Faso. *AUSTRALASIAN MEDICAL JOURNAL* 2017.

234. Noeske J, Kuaban C, Amougou G, Piubello A, Pouillot R. Pulmonary tuberculosis in the Central Prison of Douala, Cameroon. *East African Medical Journal* 2006.

235. Angora B, Assemien J, Laurent A, et al. HIV in prison in low income countries. *Aids* 2011.

236. Receveur MC, Seri B, Koffi A, et al. Prevalence of pulmonary tuberculosis among prison inmates: A cross-sectional survey at the Correctional and Detention Facility of Abidjan, Cote d'Ivoire. *Tropical Medicine and International Health* 2017.

237. Kayomo MK, Hasker E, Aloni M, et al. Outbreak of tuberculosis and multidrug-resistant tuberculosis, Mbuji-Mayi central prison, democratic Republic of the Congo. *Emerging Infectious Diseases* 2018.

238. Mashako KY, Sebahire, V & Murhabazi, V. HIV care and prevention in prison in a country in conflict: community approach in SOFEDI, Bukavu, DR Congo. XIX International AIDS Conference. Washington, D.C.; 2012.

239. Sahle ET, Amogne W, Manyazewal T, et al. Prevalence of and risk factors for Human Immunodeficiency Virus (HIV) infection in entrants and residents of an Ethiopian prison. *PLoS One* 2023.

240. Sahle ET, Blumenthal J, Jain S, et al. Bacteriologically-confirmed pulmonary tuberculosis in an Ethiopian prison: Prevalence from screening of entrant and resident prisoners. *PLoS One* 2019.

241. Kebede W, Abdissa A, Seid Y, Mekonnen Z. Seroprevalence and risk factors of hepatitis B, hepatitis C and HIV infections among prisoners in Jimma Town, Southwest Ethiopia. *Asian Pacific Journal of Tropical Disease* 2017.

242. Adjei AA, Armah HB, Gbagbo F, et al. Correlates of HIV, HBV, HCV and syphilis infections among prison inmates and officers in Ghana: A national multicenter study. *BMC Infectious Diseases* 2008.

243. Commission GA. National Health and HIV Survey of Prison Inmates in Ghana. 2013.

244. Adjei AA, Armah HB, Gbagbo F, et al. Prevalence of human immunodeficiency virus, hepatitis B virus, hepatitis C virus and syphilis among prison inmates and officers at Nsawan and Accra, Ghana. *Journal of Medical Microbiology* 2006.

245. Ministry of Health Kenya. MARPs SURVEILLANCE REPORT. *Ministry of Health, National AIDS & STI Control Programme-NASCOP* 2012.

246. Jones MJ. INTEGRATED BIO-BEHAVIOURAL SURVEILLANCE SURVEY REPORT OF KEY POPULATIONS IN LIBERIA (IBBSS, 2018). In: Health Mo, editor.; 2019.

247. Fenomanana J, Randriatsarafara FM, Ranampy FF, Randriamanantany ZA. A behavioural and HIV serological survey among detainees of Ankazondrano jail in Fianarantsoa, Madagascar. *Afr J AIDS Res* 2021.

248. Mangochi P, Bossard C, Catacutan C, et al. TB screening, prevention and treatment cascade in a Malawi prison. *The International Journal of Tuberculosis and Lung Disease* 2022; **26**(10): 956-62.

249. Banerjee A, Harries AD, Mphasa N, Yadid AE, Nyirenda T, Salaniponi FM. Prevalence of HIV, sexually transmitted disease and tuberculosis amongst new prisoners in a district prison, Malawi. *Trop Doct* 2000.

250. Chimphambano C, Komolafe I, Muula A. Prevalence of HIV, HepBsAg and Hep C antibodies among inmates in Chichiri prison, Blantyre, Malawi. *Malawi Medical Journal* 2007; **19**(3): 107-10.

251. Mallewa J, Kaombe, T & Simbeye, J. Access to preventive, testing, treatment services and retention in care for HIV key populations groups in Malawi. 2023.

252. Chigbu LN, Iroegbu CU. Incidence and spread of Mycobacterium tuberculosis-associated infection among Aba Federal prison inmates in Nigeria. *Journal of Health, Population and Nutrition* 2010.

253. Bashorun A. HIV prevalence and associated factors amongst prison inmates in Kuje federal prison, Federal Capital Territory, Abuja, Nigeria, 2013. *Pan African Medical Journal* 2015.

254. Muhammed OT, Akpa OM, Atilola GO, Komolafe IOO. Seroprevalence of HIV/AIDS and HIV risk factors among prison inmates in Ogun State, Nigeria. *HIV & AIDS Review* 2012; **11**(1): 25-30.

255. Lawrence QO, Amadi ANC, Okosa C, Ikpi PO, Chukwuemeka BC. Co-infection of Trichomonas vaginalis and HIV infection and its risk factors among prison inmates in Umuahia, Abia State, South Eastern Nigeria. *JOURNAL OF BASIC AND APPLIED ZOOLOGY* 2021.

256. Adoga MP, Banwat EB, Forbi JC, et al. Human immunonodeficiency virus, hepatitis B virus and hepatitis C virus: sero-prevalence, co-infection and risk factors among prison inmates in Nasarawa State, Nigeria. *The Journal of Infection in Developing Countries* 2009.

257. Onoja A, Mohammed SB, Ya'aba Y, Liman M, Njab J. Seroprevalence of HIV among the people of lake Chad basin of borno state, nigeria. *Journal of Phytomedicine and Therapeutics* 2016.

258. Dada MO, Akanmu AS, Esan OA. Seroprevalence of HIV among male prisoners in Lagos State, Nigeria. *Niger Postgrad Med J* 2006.

259. Abba OJ, Ibraheem IS, Idoko J. Prevalence and risk factors for HIV/AIDS among male inmates in Jos Prison, Plateau State, Nigeria. *Nigerian Journal of Parasitology* 2011.

260. AIDS NCftFa. BEHAVIORAL SURVEY COUPLED TO HIV SEROLOGY AMONG PROFESSIONALS SEX, MEN HAVING SEX SEX WITH MEN AND INMATES IN REPUBLIC OF CONGO FINAL REPORT. In: AIDS NCftFa, editor.; 2012.

261. Agency for the Promotion of Population Activities Senegal. ENQU bÊTE NATIO NALE DE SURVEILLANCE COMBINEE DES IST ET DU VIH/SIDA (ENSC 2019) COMPOSANTE COMPORTEMENTALE. In: SOCIALE MDLSEDLA, editor.; 2020.

262. Jaquet A, Wandeler G, Tine J, et al. HIV infection, viral hepatitis and liver fibrosis among prison inmates in West Africa. *BMC Infectious Diseases* 2016.

263. Ampofo WK. Sierra Leone Integrated Bio-Behavioural Survey and Size Estimation Among Female Sex Workers (FSWs), Men who Have Sex with Men (MSM), Persons who Inject Drugs (PWID), Transgender (TG) and People in Close Settings (PCS). In: Secretariat NHA, editor.; 2021.

264. Sesay M. Seroprevalence Study for Key Populations Sierra Leone. 2012.

265. The Aurum Institute NICD. Socio-behavioural and structural factors driving HIV/AIDS, STIs and Hepatitis B & C infections among inmates in Correctional Facilities, Johannesburg, 2020.

266. V Skiti EG, P Gribble, H Hausler. Screening and testing for tuberculosis and HIV in correctional facilities

in the Western Cape, South Africa. 44th World Conference on Lung Health of the International Union Against Tuberculosis; 2013.

267. Telisinghe L, Fielding KL, Malden JL, et al. High tuberculosis prevalence in a South African prison: the need for routine tuberculosis screening. *PLoS One* 2014.

268. Stevenson KA, Podewils LJ, Zishiri VK, Castro KG, Charalambous S. HIV prevalence and the cascade of care in five South African correctional facilities. *PLoS One* 2020.

269. Dlamini P, Dlamini P, Mnisi Z, Hariga F. A situational assessment on TB, HIV, syphilis, hepatitis C and hepatitis B infections and associated risk behaviours among prisoners and prison officers in Swaziland; 2012.

270. J Angolwisye FK, F Nichombe, M Minja, A Rachow, H Machibia, M Pletschette, P Clowes. First survey on TB and HIV prevalence in the prisons of the Mbeya region in Tanzania. *42nd World Conference on Lung Health of the International Union Against Tuberculosis and Lung Disease* 2011; (157).

271. Mutayoba B, Ngowi B, Kohi W. HIV prevalence and related risk factors in prison settings: findings from a rapid situational assessment in mainland Tanzania. 20th International AIDS Conference, Melbourne, Australia; 2014; 2014.

272. Steiner A, Mangu C, van den Hombergh J, et al. Screening for pulmonary tuberculosis in a tanzanian prison and computer-aided interpretation of chest X-rays. *Public Health Action* 2015.

273. M. Dahoma EM, A. Othman, A. Seha, A. Abdullah. Predisposing sexual and drug related risk factors among prisoners in Zanzibar. International Aids Society (IAS) 2009. Cape Town; 2009.

274. Ekouevi DK, D'Almeida S, Salou M, et al. HIV seroprevalence among inmates in Togo. *Med Mal Infect* 2013.

275. Akakpo AS, Ekouevi DK, Toure AM, et al. Skin disease and HIV infection among inmates in Lome, Togo: a study of 194 prisoners. *Medecine et sante tropicales* 2014.

276. United Nations Office on Drugs and Crime. A Rapid Situation Assessment of HIV/STI/TB and Drug Abuse among Prisoners in Uganda Prisons Service, 2009.

277. Kinaalwa GN, F. Integrated Legal Aid in HIV Programming in Prison Rehabilitation Services

for Better Access and Quality HIV Care - Mityana

Uganda Charity Experience. 18th International Conference on AIDS and STI's in Africa; 2015.

278. Harris JB, Siyambango M, Levitan EB, et al. Derivation of a tuberculosis screening rule for sub-Saharan African prisons. *Int J Tuberc Lung Dis* 2014.

279. Simooya OO, Sanjobo NE, Kaetano L, et al. 'Behind walls': a study of HIV risk behaviours and seroprevalence in prisons in Zambia. *Aids* 2001.

280. Simooya OO, Sanjobo N, Mulenga C, et al. Aggressive awareness campaigns may not be enough for HIV prevention in prisons-studies in Zambia suggest time for evidence based interventions. *Open Infectious Diseases Journal* 2014.

281. Maggard KR, Hatwiinda S, Harris JB, et al. Screening for tuberculosis and testing for human immunodeficiency virus in Zambian prisons. *Bulletin of the World Health Organization* 2015.

282. Kagujje M, Somwe P, Hatwiinda S, et al. Cross-sectional assessment of tuberculosis and HIV prevalence in 13 correctional facilities in Zambia. *BMJ Open* 2021.

283. Mohamed HI, Saad ZM, Abd-Elreheem EM, et al. Hepatitis C, hepatitis B and HIV infection among Egyptian prisoners: seroprevalence, risk factors and related chronic liver diseases. *J Infect Public Health* 2013.

284. Ministry of Health Lebanon. AN INTEGRATED BIO-BEHAVIORAL SURVEILLANCE STUDY AMONG MOST AT RISK POPULATIONS IN LEBANON: FEMALE SEX WORKERS, INJECTING DRUG USERS, MEN WHO HAVE SEX WITH MEN, AND PRISONERS. 2008.

285. Ziglam H, Zorgani AA, Balouz A, Abudhe AH, Elahmer O. Prevalence of antibodies to human immunodeficiency virus, hepatitis B, and hepatitis C in prisoners in Libya. *Libyan Journal of Medicine* 2012.

286. El Ghrari K, Terrab Z, Benchikhi H, Lakhdar H, Jroundi I, Bennani M. Prevalence of syphilis and HIV infection in female prisoners in Morocco. [French]. *Eastern Mediterranean Health Journal* 2007.

287. Group SHEA. Epidemiology of HIV in Sudan Staging and Analysis. In: Group SHEA, editor.; 2013.

288. Kobeissi L. The Integrated Bio-Behavioral Survey (IBBS) in Syria: 2013-2014. In: United Nations Development Program, editor.; 2014.

289. Sahin AR, Sahin AM, Gunduz A, Aktemur A, Kes-Uzun N. HIV seropositivity in a penal institution in Turkey: A cross-sectional study. [Turkish]. *Klimik Dergisi* 2018.

290. Balci E, Turker K, Senol V, Gunay O. Screening Indicators of Hepatitis A, Hepatitis B, Hepatitis C and HIV infections in Prisoners. *Viral Hepatitis Journal* 2012.

291. Keten D, Ova ME, Keten HS, et al. The prevalence of hepatitis B and C among prisoners in Kahramanmaras, Turkey. *Jundishapur Journal of Microbiology* 2016.
